# Characterization of piRNAs across postnatal development in mouse brain

**DOI:** 10.1038/srep25039

**Published:** 2016-04-26

**Authors:** Yanal Ghosheh, Loqmane Seridi, Taewoo Ryu, Hazuki Takahashi, Valerio Orlando, Piero Carninci, Timothy Ravasi

**Affiliations:** 1 Division of Applied Mathematics and Computer Sciences, King Abdullah University of Science and Technology, Thuwal 23955-6900, Kingdom of Saudi Arabia; 2 KAUST Environmental Epigenetic Program (KEEP), Division of Biological and Environmental Sciences & Engineering, King Abdullah University of Science and Technology, Thuwal 23955-6900, Kingdom of Saudi Arabia; 3 RIKEN Center for Life Science Technologies, Division of Genomic Technologies, 1-7-22 Suehiro-cho, Tsurumi-ku, Yokohama, Kanagawa 230-0045, Japan; 4 Department of Medicine, Division of Genetic, University of California, San Diego. 9500 Gilman Drive La Jolla, California 92093-0688, USA

## Abstract

PIWI-interacting RNAs (piRNAs) are responsible for maintaining the genome stability by silencing retrotransposons in germline tissues– where piRNAs were first discovered and thought to be restricted. Recently, novel functions were reported for piRNAs in germline and somatic cells. Using deep sequencing of small RNAs and CAGE of postnatal development of mouse brain, we identified piRNAs only in adult mouse brain. These piRNAs have similar sequence length as those of MILI-bound piRNAs. In addition, we predicted novel candidate regulators and putative targets of adult brain piRNAs.

PIWI-interacting RNAs (piRNAs) are short non-coding RNAs that protect the genome integrity by silencing transposable elements (TEs)^1^,^2^,^3^,^4^,^5^,^6^. They are scattered in genomic clusters that can span up to hundreds of kilobases, and sometimes overlap^7^. These piRNA clusters are transcribed as long single-stranded transcripts (piRNA precursors) by RNA POL II^8^. In postnatal testes, these precursors undergo primary processing^9^ in which they are exported from the nucleus into the cytoplasm where they are finally made into mature piRNAs with Uracil predominantly at their 5′ end^7^,^10^. Mature piRNAs then associate in a length-dependent manner with PIWI-like family proteins, such as MILI, MIWI and MIWI2 to create a mature piRNA-induced silencing complex (piRISC)^7^. Guided by the piRNA, piRISC seeks out complementary target sequences and effectively silences them through either post-transcriptional gene silencing^11^,^12^,^13^ or by inducing DNA methylation^14^,^15^.

In mouse, piRNAs can be subdivided into two groups according to the time of their peak expression; pre-pachytene and pachytene piRNAs. Pre-pachytene piRNAs are most abundant prior to the pachytene stage in meiosis but maintain a basal expression level even during later stages^8^. They are known to target TEs and cleave them using the slicer activity of the PIWI domain^16^. Whereas pachytene piRNAs reach their peak expression during the pachytene stage of meiosis and are more abundant^8^. They are primarily derived from intergenic regions and are reported to control expression of protein-coding genes^17^. However, their functions are yet to be fully determined.

Although piRNAs were previously thought to be exclusively found in germ cells, recent studies reported them in somatic cells such as follicle cells in fruit fly ovaries, principal/basal cells in macaque epididymis and tissues such as sea slug central nervous system, and rat cerebral cortex^14^,^18^,^19^,^20^,^21^. A recent effort to identify piRNAs in the mouse brain fell short when the reported sequences were compared with known gene annotation. It has been revealed that snoRNAs and other abundant RNAs were misclassified as piRNAs^14^,^22^. Saxena and colleagues observed a 1.9 increase in total piRNA of Mecp2 knockout mouse cerebellum when compared to wild type mouse. It also suggested that this increase might cause gene-specific mis-regulation in Rett syndrome, which is often associated with mutations in Mecp2 gene. However, the authors admitted that more in-depth analysis was required to corroborate their preliminary results^23^. Here we identified piRNAs in adult mouse brain that exhibit the hallmarks of piRNA such as length (24–31 bp) and 1U bias. Moreover, we demonstrate that these piRNAs are similar to MILI-bound piRNAs with regards to their length. Finally, we predicted novel candidate regulators and potential targets of piRNAs in adult mouse brain.

## Results

### piRNAs are expressed in mouse brain

**Figure 1:**
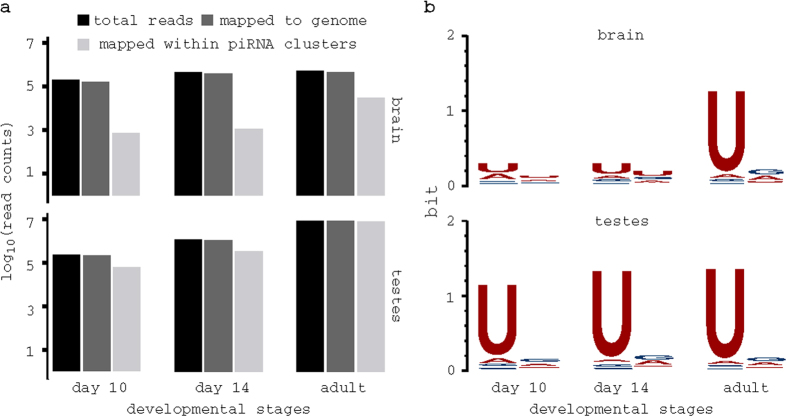
Sequencing of piRNA in brain and testes. (**a**) Bar plot shows the number of sequenced reads; reads that mapped to genome; and reads mapped within annotated piRNA clusters. (**b**) Sequence bias of reads obtained in each stage and tissue; unlike reads at adult stage in brain, reads at 10 dpp and 14 dpp lack significant 1U bias, all reads from testes samples showed significant 1U bias. Sequence logo was obtained using Weblogo^68^.

To investigate whether piRNAs were present in mouse brain, we deep-sequenced small RNAs (enriching for piRNAs based on their size) from brain and testes tissues. The samples were taken at 10 days post-partum (dpp), 14 dpp, and adult stages. We obtained 199,003; 457,461; 529,081 and 223,341; 1,177,846; 8,359,064 reads for 10 dpp; 14 dpp; adult stages in brain and testes, respectively. Of these reads 161,869 (81%); 390,929 (85%); 453,456 (86%) and 213,740 (96%); 1,132,436 (96%); 8,302,184 (99%) mapped to the UCSC mouse genome release 9 (mm9) for brain and testes, respectively (Fig. 1a). We focused on previously defined 214 piRNA clusters^8^. Out of the mapped reads, 741 (0.46%) and 1,106 (0.28%) reads mapped within piRNA clusters at 10 dpp and 14 dpp in brain, respectively, in contrast to 31,398 (7%) reads in adult brain (Fig. 1a). As for testes samples, 58,835 (28%); 340,181 (30%) and 8,234,258 (99%) mapped within piRNA clusters for 10 dpp; 14 dpp and adult stages, respectively. This suggests that piRNAs are unlikely to be present at 10 and 14 dpp in brain. In fact, only reads from these two stages lack significant 1U bias characteristic of primary piRNAs (Fig. 1b).

The expression of piRNAs in the central nervous system of adult mouse has been reported by Lee and colleagues^22^. However, other reports^14^ noted that those piRNAs were partial snoRNA. Saxena and colleagues^23^ also identified piRNA in mouse cerebellum. However, many of their most abundant piRNAs were previously reported by Lee *et al.*^22^. To avoid such a misclassification, we compared adult brain reads that mapped within piRNA clusters against annotated non-coding RNAs^24^,^25^,^26^ using BLAST^27^. Only 1.1% of the reads had perfect matches. This result, coupled with the facts that adult brain reads map within annotated piRNA clusters and show significant 1U bias, suggests that those reads are bona fide piRNAs.

### piRNA clusters expressed in brain are not tissue specific

**Figure 2:**
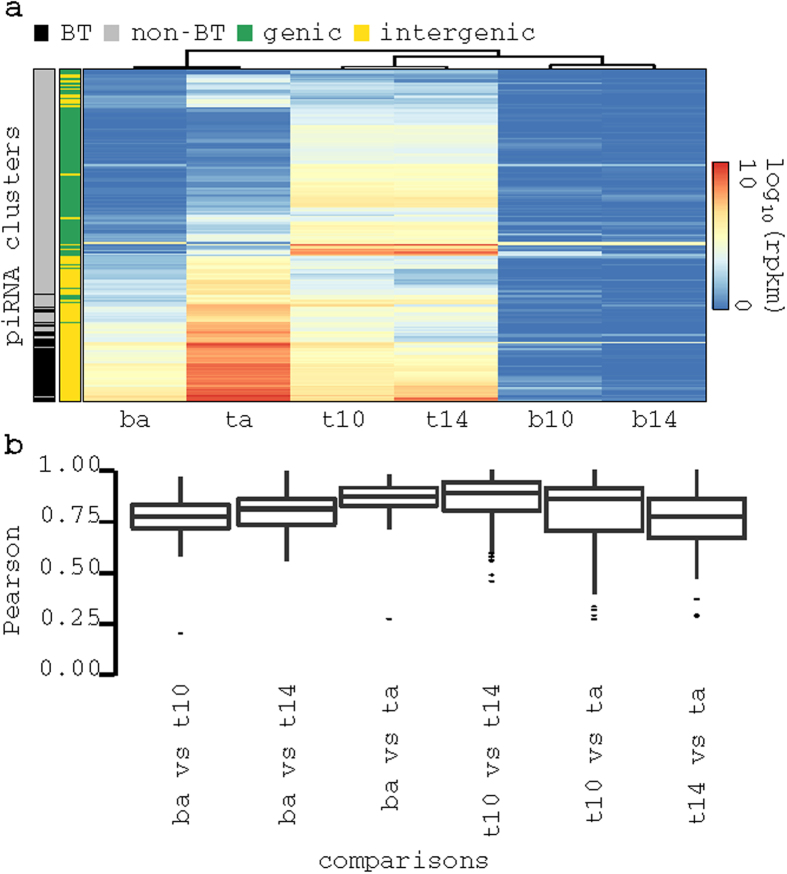
Expression profile of piRNA clusters in brain and testes clusters. (**a**) Heatmap showing expression profiles of piRNA clusters (rows); brain adult sample cluster with testes adult sample (based on Pearson correlation), suggesting they share expressed piRNA clusters; annotation at the left of heatmap shows that most piRNA clusters expressed in adult brain (BT clusters) are intergenic (**b**) boxplot showing distributions of coverage correlation that was computed along the length of expressed piRNA between all samples; high correlation indicate that piRNAs production along the length of piRNAS clusters is similar across all conditions.

piRNA clusters exhibit distinct expression profiles across developmental stages in both tissues (Fig. 2a). For instance, 47 piRNA clusters were expressed (see Materials and Methods and Fig. S1) in the adult stage in brain and 139, 154, and 115 piRNA clusters were expressed in testes at 10 dpp, 14 dpp and adult stages, respectively.

Adult brain piRNAs were mostly produced by intergenic piRNA clusters (p-value < 0.002, one tailed fisher exact test) which dominate piRNA production in pachytene stage of spermatogenesis^8^ (Fig. 2a). Since all piRNA clusters expressed in adult brain (henceforth denoted as BT clusters) were expressed in adult testes, we sought to determine whether there were any tissue-specific piRNA-producing regions within the clusters. We examined the coverage of piRNAs along the length of BT clusters in both tissues at adult stage and did not find tissue-specific piRNA-producing regions (median Pearson correlation 0.87; Fig. 2b). Therefore, we expected BT clusters to produce similar piRNA populations in both tissues. Indeed, out of 14,978 unique piRNA sequences mapped within BT clusters 13,253 (88.5%) were also present in adult testes (exact sequence match). Whereas only 2,692 (18.0%) and 6,417 (42.8%) were present in testes at 10 dpp and 14 dpp. Overall, this suggests that BT clusters produce a subpopulation of adult testes piRNAs.

### piRNAs in adult brain are similar to MILI-bound piRNAs

**Figure 3:**
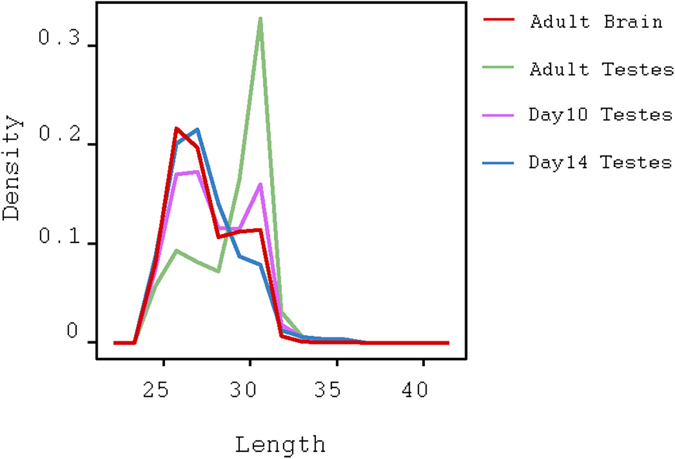
piRNAs in adult brain are similar be MILI-bound. (**a**) Length distribution of piRNAs at adult brain reveals a peak at 26~27 bases which is commonly associated with MILI-binding. Consistent with current knowledge, A high peak at 29~31 bases in adult testes and a smaller peak at 26~27 bases are associated with MIWI- and MILI-binding, respectively.

Adult testes piRNAs are composed of both MIWI- and MILI-bound piRNAs^28^, each of those proteins binds to a distinct sequence length of piRNAs^7^. Since adult brain piRNAs are a subpopulation of adult testes piRNAs, we sought to determine which of those two proteins might bind adult brain piRNAs. The length distribution of adult brain piRNAs peaks at 26~27 bps, which is the nominal length for MILI-bound piRNAs^7^. Similarly to the length distribution of piRNAs in the adult brain, the length distribution of testes piRNAs at 14 dpp also peaks at 26~27 bps. This has been attributed to the presence of MILI-bound piRNAs^29^. Whereas, in adult testes, the distribution peaks at 26~27 and 29~30 bps; the first peak corresponds to MILI-bound piRNAs and the second peak corresponds to MIWI-bound piRNAs^7^ (Fig. 3). These peaks and their associations to PIWI-like proteins are consistent with publicly available immuno-precipitation (IP) data (Supplementary Fig. S2). Although adult brain piRNAs are similar to MILI-bound piRNAs with regards to their length, we could not detect an appreciable Mili expression (normalized expression ≤0.5) in adult brain based on our CAGE expression data nor through independent sources^30^,^31^.

### ZIC2 and MEIS1 may regulate transcription of piRNA clusters in adult brain

**Figure 4:**
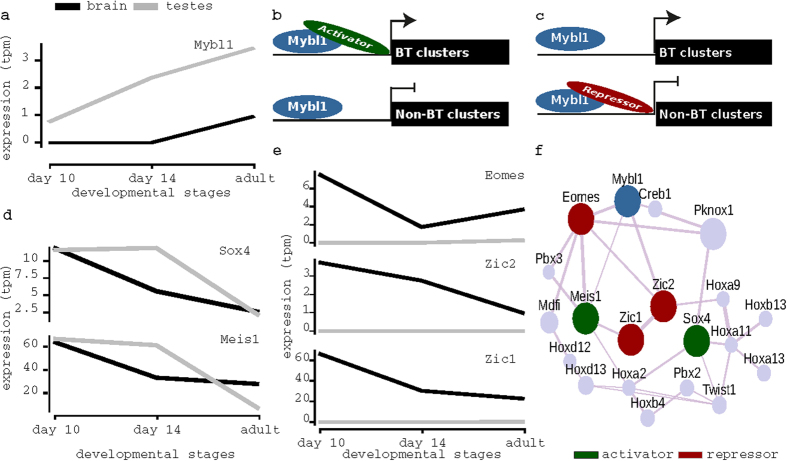
Identification of candidate regulators of BT piRNA clusters. (**a**) Expression profile of Mybl1. (**b**) Diagram depicting the first possible scenarios in which BT clusters are expressed due to an activator TF. (**c**) Similar to (**b**) but for second scenario, in which non-BT clusters are silenced in adult brain due to a repressor TF. (**d**) Expression profile of candidate TFs that fit first scenario. (**e**) Expression profile of candidate TFs that fit the second scenario. (**f**) Co-expression network obtained from GeneMANIA^63^ showing that many candidates co-express with Mybl1; TFs were classified as activator or a repressor based on our suggested scenarios as their assumed function in the scenarios does not contradict with known literature about their function.

MYBL1 is necessary for the transcription of pachytene piRNA clusters in mouse testes^8^. In fact, MYBL1 binds to the promoters of 106 piRNA clusters in adult testes, including 46 BT piRNA clusters. However, our CAGE expression data shows that Mybl1 is lowly expressed (see Materials and Methods) in adult brain (Fig. 4a). This is consistent with data obtained from other studies^31^ as well as Allen Brain Atlas^30^. Moreover, only a subset of its target piRNA clusters was expressed in adult brain. Thus, we suspect that other factors, alongside MYBL1, control the expression of this subset. Here, we considered two possible scenarios: either a transcription factor (TF) is activating only BT clusters (Fig. 4b) or a TF repressing piRNA clusters not expressed in adult brain but expressed in adult testes (non-BT clusters) (Fig. 4c).

To determine the likely scenario, we used DREME^32^ in conjunction with TOMTOM^33^ to identify discriminatory transcription factor binding sites (TFBFs) between the promoters of BT and non-BT clusters. Initially, we identified 162 discriminatory TFs. Using CAGE to determine their expression profiles and UniProt^34^ to determine their functional annotation (repression or activation), we filtered them to five candidate TFs (see Materials and Methods and Supplementary Table S1): two were unique to BT clusters, namely, MEIS1 and SOX4; three were unique to non-BT clusters, namely, ZIC1, ZIC2 and EOMES (Fig. 4d,e). All TFs were implicated in neuron differentiation and development^35^,^36^,^37^,^38^.

Although ZIC2 can act as both repressor and activator, as a repressor it fits the second scenario. Indeed, based on public Chromatin Immunoprecipitation Sequencing (ChIP-Seq) data, ZIC2 binds the promoters of non-BT clusters in adult cerebellum^39^. Unfortunately, we did not find data for the other TFs related to their binding in adult brain. Interestingly, Zic2, Eomes and Meis1 co-express with Mybl1 (Fig. 4f). Therefore, we predict that MEIS1, EOMES and specifically ZIC2 are likely candidates for the regulation of piRNA cluster transcription.

### Prediction of targets of adult brain piRNAs

In addition to the well-established role of piRNAs in TE-silencing, several studies have suggested that piRNAs may be involved in the regulation of genes in several species^14^,^17^,^40^,^41^. In order to investigate whether adult brain piRNAs were involved in mRNA deadenylation, we adapted the methodology described by^17^ and took into account only the top-scoring predicted gene target for each piRNA according to miRanda^42^ (Supplementary Table S2). Considering that putative gene targets should be down-regulated when compared to non-target genes if adult brain piRNAs were involved in mRNA deadenylation, we performed Mann Whitney test on the non-zero fold change, as calculated by GFOLD^43^, of the genes between 14 dpp and adult stages in brain (see Materials and Methods). Although there was no significant divergence in differential expression between predicted target genes and non-target genes (p-value 0.5122) (Supplementary Fig. S3), we cannot deny the possibility that a few adult brain piRNAs may be involved in small-scale regulation of target mRNAs.

A more recent study reported an alternative approach to identify piRNA targets^44^. This approach relied on a specific signature of partial complementarity of piRNAs to their putative targets (see Materials and Methods). Using this specific signature on our adult brain piRNAs, we were able to identify 41 potential mRNA targets of piRNAs (Supplementary Table S3). Using CPDB^45^, Gene Ontology (GO) enrichment for the putative mRNA targets revealed they were significantly (p-value ≤ 0.001) associated with pigmentation. Moreover, these putative targets were significantly (p-value ≤ 0.001) associated with Cholinergic synapse pathway. Applying the same signature on repeat elements, we identified 7,565 putative targets (Supplementary Table S4). Of which, 3,081 were short interspersed nucleotide elements (SINEs); 1,151 were long terminal repeats (LTRs); 450 were long interspersed nucleotide elements (LINEs). GO enrichment for repeat element targets revealed their significant (p-value ≤ 0.001) association with cardiac neural crest cell development involved in outflow tract morphogenesis based on GREAT^46^. In conclusion, these *in silico* predictions of putative targets should help guide future *in vitro* experimental validation.

## Discussion

Here, we characterize piRNA in mouse brain throughout postnatal development and show that piRNAs are only present in the adult stage of brain development. These piRNAs display significant 1U bias and are found in an intergenic subset of previously annotated piRNA clusters^8^.

piRNAs bind to PIWI-like proteins according to their sequence length^7^. The sequence length of adult brain piRNAs peaks at 26~27 bases, which is a characteristic of piRNAs that bind MILI. However, the expression of PIWI-like genes– including Mili– were absent in adult brain. Since piRNAs function as part of a complex with PIWI-like proteins, it is unclear how these piRNAs function in the adult brain. One hypothesis is that piRNAs associate with a different protein; another is that Mili is expressed in a unique cell type that is hard to detect using whole brain sequencing.

MYBL1 is a key regulator of adult testes piRNA clusters^8^. In the brain, we showed that Mybl1 was solely expressed in adult stage based on our CAGE expression data. However, despite its expression, only a subset of adult testes piRNA clusters was expressed. This suggests that TFs other than MYBL1 may be implicated in the regulation of piRNA clusters in the brain. Consequently, we identified five TFs that may regulate piRNA clusters’ expression alongside MYBL1. Three of them co-express with Mybl1. One candidate is ZIC2, which is involved in neurogenesis^36^,^47^. Zic2 is widely expressed in adult brain^48^ and binds promoters of non-BT piRNA clusters in adult cerebellum^39^. Thus, we suspect that ZIC2 may repress those piRNA clusters. Another candidate is MEIS1, an activator TFs, which has binding sites only in promoters of piRNA clusters expressed in adult brain. In conclusion, we predicted novel candidate regulators of piRNA clusters for future validation.

To determine whether piRNAs in adult brain were involved in mRNA deadenylation, we used miRanda^42^ to identify piRNA targets as previously described^17^. However, these predicted targets were not more likely to be down-regulated when compared to non-target genes (Mann-Whitney test; p-value 0.5122).

A more recent study reported a specific targeting signature of adult testes piRNAs^44^. Based on this signature, we predicted 41 candidate mRNA targets and 7,565 repeat element targets. GO enrichment of candidate mRNA and repeat element targets were associated with pigmentation, and cardiac neural crest development, respectively. In mouse embryo, cardiac neural crest stem cells were shown to be able to differentiate into pigment cells^49^. Furthermore, the predicted mRNA targets were also associated with cholinergic synapse pathway.

In adult testes, L1 elements are repressed through multiple mechanisms, including piRNAs^50^. Furthermore, L1 elements are derepressed in mice with mutant MILI^11^. Although L1 elements are active during brain development^51^, how they are controlled in brain and whether piRNAs play a role in their regulation is still unclear^52^. Therefore, future functional analysis is required.

In conclusion, we described the developmental expression of piRNA in postnatal mouse brain. We showed that these piRNAs are similar to MILI-bound piRNAs in terms of their length and suggested new candidate regulators of those piRNAs. Although a deeper investigation into piRNAs in the brain is required, we believe the data and results described here provide new insights and a valuable resource for the small-RNA community.

## Materials and Methods

### RNA Extraction

Total RNA was extracted using RNA-Bee (AMS Biotechnology) according to the manufacturer’s instructions, from pools of whole brain and pools of testis of male C57Bl/6 mice, sacrificed through cervical displacement, at 10 dpp, 14 dpp and adult, in accordance with the approved guidelines. All experimental protocols were approved by the Roslin Institute. Quality and quantity of the total RNA was measured by Nanodrop spectrophotometer and Bioanalyzer RNA chips (Agilent). For sequencing library preparation, low molecular weight RNA (<40 nucleotides long) was isolated from the total RNA using a FlashPAGE fractionator (Life Technologies).

### Preparation of RNA-Seq library

50 ng of fractionated small RNA from brain and testes at 10 dpp, 14 dpp and adult was tagged and used to generate cDNA libraries according to Kawano *et al.*^53^. Briefly, adenylated 3′ adapters were ligated to 3′ end of small RNAs using a truncated RNA ligase enzyme followed by 5′ adaptor ligation to 5′-monophosphate ends using RNA ligase enzyme, ensuring specific ligation of non-degraded small RNAs. cDNA was prepared using a primer specific to the 3′ adaptor in the presence of dimer eliminator and amplified for 14 PCR cycles using a special forward primer targeting the 5′ adaptor containing additional sequence for sequencing and a reverse primer targeting the 3′ adaptor. The amplified libraries which contained piRNA and sequencing linkers were run on a 6% polyacrylamide gel and then the 80–84 bp bands (which correspond to inserts of 26–32 nucleotides cDNAs) were extracted by gel extraction protocols (QIAGEN). Libraries were sequenced after quality check on a Bioanalyzer DNA 1,000 chip (Agilent).

### Preprocessing of RNA-Seq tags

Multiplexed barcode sequencing for three pools during three postnatal developmental stages from two tissues was performed on RNA-Seq tags using Illumina GA-IIX (40 bp single end tags), where the barcode and the adapter were ligated to the 5′ end and 3′ end, respectively. In order to retain high quality RNA-Seq reads, we trimmed all bases, including bases for barcode and 3′ adapter, which had a quality score ≤15 as well as all subsequent bases for each read. In order to extract the endogenous sequences from the RNA-Seq reads, we stripped the four base 5′ barcodes as well as at least one base of 3′ adapter. Any read, which was not stripped, was discarded, resulting in endogenous sequences with a maximum length of 35 bases. Since piRNA size ranges from 26 to 31 bases^1^, we discarded all sequences whose length was ≤24 bases. Finally, for each developmental stage in each tissue we created a single dataset by concatenating all the sequences from the three pools together. We retained duplicate reads. Subsequently, we aligned the sequences on the non-repeat-masked UCSC release 9 of the mouse genome (MM9)^54^ using bowtie2 v2.2.5^55^ using the sensitive preset option and allowed a maximum 100 alignments. All the reads that aligned to the genome were retained and used for subsequent analysis.

### Comparison against non-coding RNA

We compared adult brain reads that mapped within annotated piRNA clusters to known non-coding RNAs. These non-coding RNAs include NONCODE v3.0 snoRNA^24^, UCSC tRNA^25^, miRBase v21 miRNA^26^ and the NCBI complete ribosomal DNA unit. The comparison was performed using NCBI BLASTN v2.2.31+^27^. Except for “-task blastn”, Default parameters were used.

### piRNA Cluster expression

The expression of each piRNA cluster was based on the number of reads mapped within the piRNA cluster keeping multi-mapping reads. We used RPKM (Reads Per Kilobase per Million mapped reads) for normalization. In order to distinguish between expressed and non-expressed piRNA clusters, we observe the following criteria: a piRNA cluster must have at least 100 mapped reads; the RPKM expression level of piRNA cluster must be at least 10; the reads must map within the entire piRNA cluster and not concentrated in a small region, to reduce the effect of PCR duplicates, this was done by requiring that the maximum depth of the reads divided by the total number of mapped reads per piRNA cluster be less than 0.9. Based on these criteria, we designate clusters as expressed or not for each developmental stage in each tissue independently (Supplementary Fig. S1).

### Preparation of CAGEscan library

CAGEscan is an enhancement upon CAGE (Cap Analysis of Gene Expression) which uses paired-end sequencing approach to sequence an additional random block in the 3′ direction of the same transcript. The preparation of the CAGEscan library was adapted from published protocol^56^ and modified to work with Illumina GA-IIX 36 cycles paired end CAGEscan read sequencer. The first-strand cDNAs were created using 500 μg brain and testes RNA of male C57Bl/6 mice, sacrificed through cervical displacement, with 2 μl of 0.66 M D-threalose, 3.3 M D-sorbitol^57^, 100 μM template switching oligonucleotide (5′-TAGTCGAACTGAAGGTCTCCAGCArGrGrG), 10 μM random reverse-transcription primer with a random pentadecamer tail (5′-GTACCAGCAGTAGTCGAACTGAAGGTCTCCTCTN15). The mixed solution was reduced in volume to 2 μl in a centrifugal evaporator at room temperature then heated for denaturizing at 65 °C for 10 min and transferred quickly on an ice-water mix. Reverse transcription was accomplished in a volume of 10 μl with the following components: 1.25 × first-strand buffer, 650 μM dNTPs, 1.3 mM DTT, 925 mM betain and 200 units SuperScript II, and the reaction was incubated at 22 °C for 10 min, 50 °C for 30 min, 75 °C for 15 min. Synthesized cDNAs were purified with Agencourt RNAClean XP kit following the manufacturer’s instructions. The purified cDNAs were eluted to 40 μl water then synthesized the second strand cDNA and the resulting product by semisuppressive PCR^58^. The determination of the optimal PCR cycle number in pilot reactions was used quantitative PCR: 0.15 × purified cDNA, 1 × SYBR Premix Ex Taq (TaKaRa), 100 mM semisuppressive forward primer (5′- TAGTCGAACTGAAGGTCTCCAGC) and 100 mM semisuppressive reverse primer (5′-TGACGTCGTCTAGTCGAACTGAAGGTCTCCGAACC) with a StepOne Real-Time PCR system. The thermal cycling program was: 5 min 95 °C and 40 × (15 s at 95 °C, 10 s at 65 °C, 2 min at 68 °C). After determination, PCR was performed on a large-scale hot-start PCR with 3 μl of purified first-strand cDNA as template in a final volume of 50 μl using a reaction mixture containing 1.25 U TaKaRa Ex Taq HS (TaKaRa), 200 μM each dNTP mixture (TaKaRa), 1 × Ex Taq buffer (TaKaRa), 100 mM semisuppressive forward primer and 100 mM semisuppressive reverse primer with the following thermal cycling program: 5 min 95 °C and 17–19 cycles × (15s at 95 °C, 10 s at 65 °C, 2 min at 68 °C). The amplified DNA was purified with Agencourt AMPure XP kit then measured the concentration by NanoDrop 1000.

To create adaptor sequences for Illumina GA-IIX sequencer, semisuppressive PCR cDNA was amplified using 20 ng DNA in a final volume of 50 μl with a mixture containing 1.25 U Ex Taq HS (TaKaRa), 200 μM each dNTP mixture (TaKaRa), 1 × Ex Taq buffer (TaKaRa), 200 mM forward primer (5′–AATGATACGGCGACCACCGAGATCTACACTAGTCGAACTGAAGG) and 200 mM reverse primer (5′–CAAGCAGAAGACGGCATACGAGATCGGTCTCGGCATTCCTGCTGAACCGCTCTTCCGATCT) following thermal cycling program: 1 min 95 °C, 15 s at 95 °C, 10 s at 55 °C, 2 min at 68 °C and 6 cycles × (15 s at 95 °C, 10 s at 65 °C, 2 min at 68 °C). The amplified DNA was purified with Agencourt AMPure XP kit then measured the concentration by Agilent High Sensitivity DNA kit and adjusted 16 pM final concentration (from 200 bp–700 bp) on GAIIx. CAGEscan tags were amplified with Cluster generation kit with modified sequence primer (5′–TAGTCGAACTGAAGGTCTCCAGCA) following Cluster generation protocol (Illumina) then generated clusters were sequenced with 36 cycle paired end CAGEscan read sequencing kit by Illumina GA-IIX.

### Preprocessing of CAGEscan tags

Multiplexed barcode sequencing for three pools during three postnatal developmental stages from two tissues was performed in CAGEscan tags using Illumina GA-IIX (105 bp paired end tags), where the adapter followed by six bases of barcode and three consecutive guanine bases were ligated to the 5′ end of the forward mate while only the adapter was ligated to the 5′ end of the reverse mate. After removing any partial 5′ and 3′ adapters for both mates, we trimmed all bases which had a quality score ≤15 as well as all subsequent bases. Afterwards, we removed the nine bases comprising the barcode and the three guanine bases from the 5′ end of the forward mate. We then discarded any read whose length was ≤15 bases.

We mapped the remaining paired-end sequences onto MM9 using bowtie2 v2.2.5^55^, using the sensitive preset option, in addition to Phred64 quality scores. We considered dove-tail sequences to be discordant pairs and we retained non-discordant sequences.

### Gene expression analysis

We used the R package CAGEr v1.4.1^59^, allowing any mapping quality, to retrieve CTSSs. Then we normalized the expression of each of the CTSS according to power-law normalization^60^ in transcripts per million (tpm). To obtain expression value for each gene, we summed the tpm for each unique CTSS within the one Kb upstream and 0.5 Kb downstream of all its transcription starting sites. Any gene whose normalized expression value was ≥0.5 was designated as expressed. Differential gene expression was performed using GFOLD^43^ v1.1.2 using 0.01 as cutoff for the False Discovery Rate (FDR).

### Prediction of discriminatory regulatory TFs

In order to investigate the TFs that may be regulating the intergenic piRNA clusters expressed in adult brain, we used DREME v4.10.1^32^, using default parameters, to find significantly overrepresented (E-value ≤0.05) discriminatory motifs that are unique to piRNA clusters expressed in adult brain and motifs that are unique to piRNA clusters expressed in adult testes excluding those expressed in adult brain. Then we compared the motifs against mouse UniPROBE^61^ and JASPAR^62^ vertebrate using TOMTOM^33^, with default parameters, to identify the TFs with binding sites most similar to those specific motifs. Using a normalized expression cutoff of 0.5 tpm, we required that all TFs be higher than this threshold in adult brain. Furthermore, TFs whose binding sites were overrepresented in promoters of adult brain piRNA clusters were required to be expressed above this threshold for all testes developmental stages. Whereas, TFs whose binding sites are overrepresented in the subset of piRNA clusters expressed in adult testes but not in adult brain were required to be expressed no more than that threshold on all testes developmental stages. Finally, we retained only mouse TFs whose expression patterns are consistent with the two proposed scenarios as mentioned above. Finally, we used UniProt^34^ to determine whether a TF was an activator or a repressor. We used GeneMANIA^63^ database to visualize co-expression between the candidate TFs.

### piRNA Target Prediction

In order to determine whether piRNAs in adult brain were involved in mRNA deadenylation as shown previously in testes, we adopted a similar methodology to that described by Gou *et al.*^17^. Briefly, we considered all expressed (normalized CAGE expression ≥0.5) genes at either 14 dpp or adult stages in brain as potential gene targets of any adult brain piRNA. Using miRanda^42^ v3.3a to match piRNAs to their targets, we set a more stringent score threshold, 160, for reporting hits in contrast to 150 used originally by Gou *et al.*^17^. Then for each piRNA, we considered only the top-scoring gene target. The Differential expression was obtained from the generalized fold change as given by GFOLD^43^ for each gene between 14 dpp and adult stages in brain. To determine whether predicted target genes were more likely to be down-regulated than non-target genes, we selected all the genes that exhibited any differential expression, up or down. The distribution of non-zero GFOLD values of target genes was then compared to the distribution of non-zero GFOLD values of non-target genes using Mann-Whitney test.

Alternatively, we also used the method introduced by Goh *et al.*^44^ that takes advantage of unique signatures of piRNA targeting. This signature relies on the divergent partial complementarity of sense piRNAs and guide piRNAs. Sense piRNAs were aligned to the genome using bowtie v1.1.1^64^ with parameters “–a –v 0”. Guide piRNAs were mapped also using bowtie v1.1.1^64^ with parameters “-a -n 0 -l 10 -e 160”. We used SAMtools^65^ to ensure partial complementarity. Since this method applies to both mRNA and repeats, we also investigated any transposable element, whose length was 75% of the consensus length as determined by HOMER^66^. Using BEDTools^67^, any mRNA or repeat element which overlapped the piRNA targeting signature was designated as potential target of piRNAs.

### Coverage of piRNAs along the length of piRNA clusters

Each expressed piRNA cluster was divided into non-overlapping windows of 100 bases. Coverage was determined based on the percentage of total reads that mapped within each window for each piRNA cluster to the total reads that mapped within the entire piRNA cluster. This was done for all expressed piRNA clusters in each developmental stage for each tissue independently. We then tested the Pearson correlation of the coverage, as previously defined, of the same expressed piRNA cluster across two samples for each of the expressed piRNA clusters common to both samples. For the correlation distribution, we only retained significant (p-value ≤ 0.001) correlations.

## Additional Information

**Accession Codes**: The data is available under the Bioproject ID SRP073223.

**How to cite this article**: Ghosheh, Y. *et al.* Characterization of piRNAs across postnatal development in mouse brain. *Sci. Rep.* **6**, 25039; doi: 10.1038/srep25039 (2016).

## Supplementary Material

Supplementary Information

Supplementary Table S2

Supplementary Table S3

Supplementary Table S4
