# Supplementary material for: Characterization of piRNAs across postnatal development in mouse brain
Source: Sci Rep. 2016 Apr 26;6:25039. doi: 10.1038/srep25039 (PMC4844963; doi:10.1038/srep25039)
Supplement: Supplementary Information [file srep25039-s1.pdf]

Supplemental information for "Characterization of piRNAs across postnatal development in mouse brain".

Yanal Ghosheh[a,b], Loqmane Seridi[a,b], Taewoo Ryu[a,b], Hazuki Takahashi[c], Valerio Orlando[b], Piero Carninci[c] and Timothy Ravasi[a,b,d,*]

[a] Division of Applied Mathematics and Computer Sciences, King Abdullah University of Science and Technology, Thuwal 23955-6900, Kingdom of Saudi Arabia.

[b] KAUST Environmental Epigenetic Program (KEEP), Division of Biological and Environmental Sciences & Engineering, King Abdullah University of Science and Technology, Thuwal 23955-6900, Kingdom of Saudi Arabia.

[c] RIKEN Center for Life Science Technologies, Division of Genomic Technologies, 1-7-22 Suehiro-cho, Tsurumi-ku,Yokohama, Kanagawa 230-0045, Japan.

[d] Department of Medicine, Division of Genetic, University of California, San Diego. 9500 Gilman Drive La Jolla, California 92093-0688, USA.

[*]Corresponding Author: Timothy Ravasi (timothy.ravasi@kaust.edu.sa). King Abdullah University of Science and Technology. Thuwal, Kingdom of Saudi Arabia.

Supplemental Figures

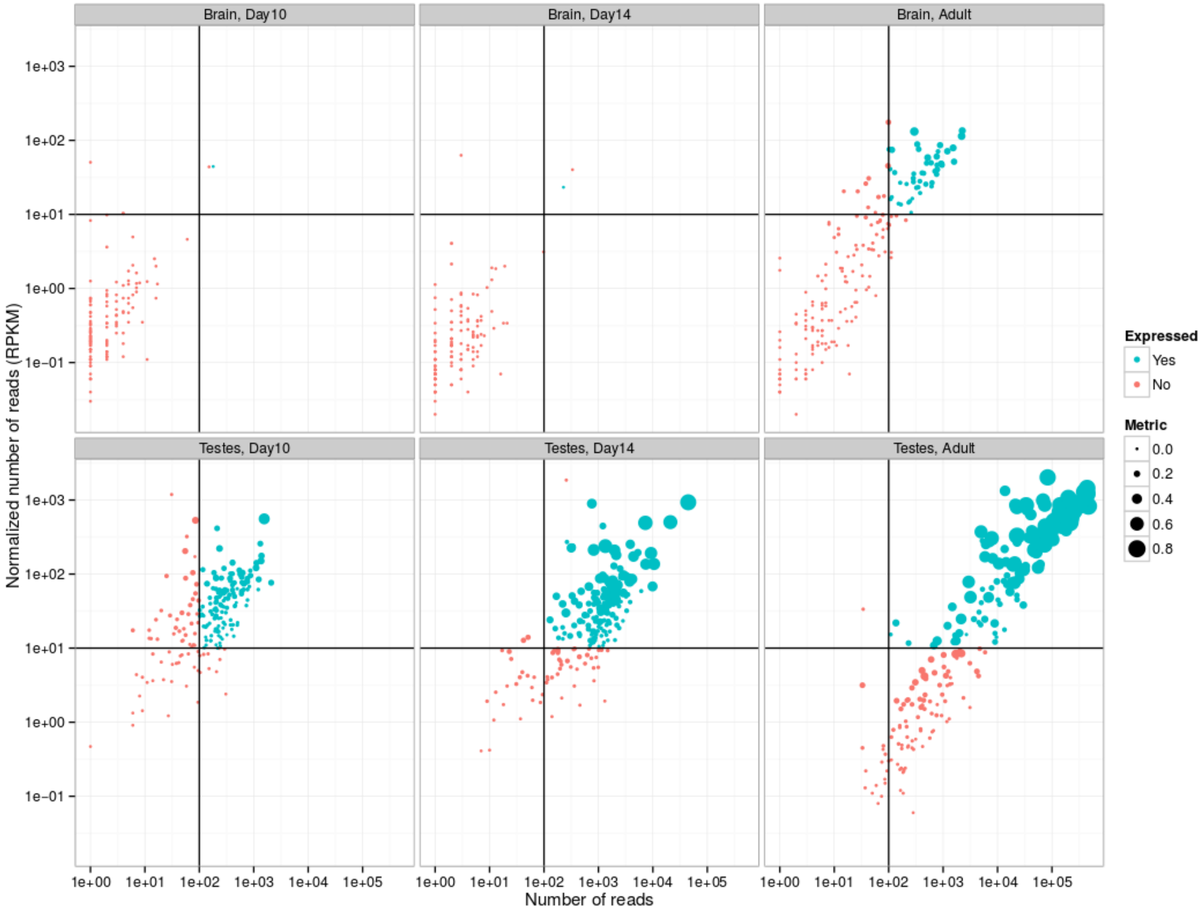

Figure S1. Filtering non-expressed piRNA clusters**.** scatter point shows expressed piRNA clusters (blue): pass threshold of 10 rpkm (horizontal); 100 reads (vertical); have more uniform-like coverage of reads (metric; see methods).

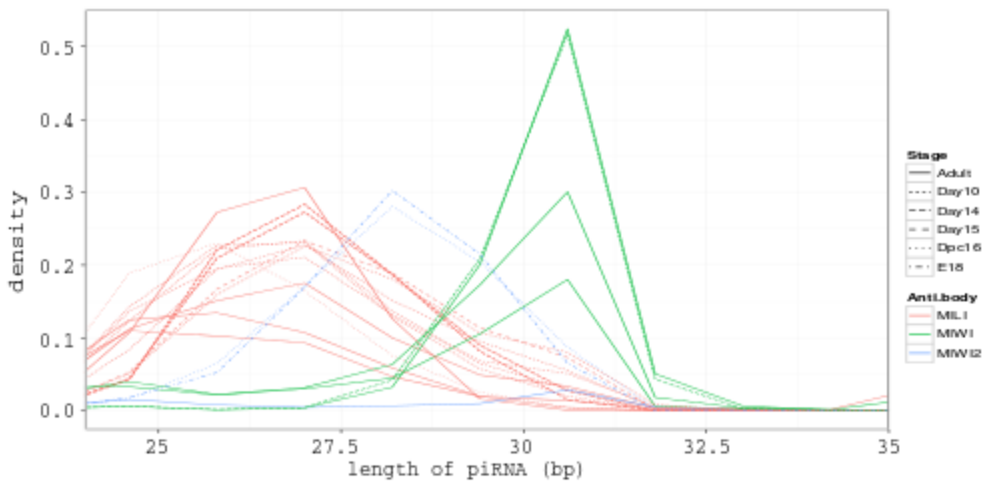

Figure S2. Piwi-like genes bind piRNAs of specific sequence lengths. lineplot shows sequence length distribution of piRNAs obtained from publicly available PIWI-like proteins IP datasets. piRANs of lengths 26~27bp, 28~29bp and 29~31bp associate to MILI, MIWI2 and to MIWI. These data sets were obtained from the following NCBI GEO series: GSE7414, GSE12757, GSE16023, GSE18825, GSE19172, GSE26251, GSE27623, GSE32184, GSE39203.

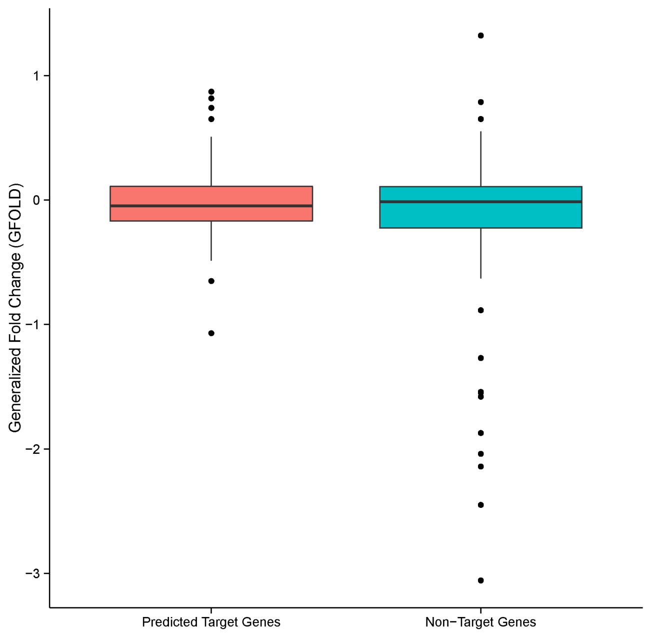

Figure S3: miRanda-predicted target genes do not exhibit more down regulation than non-target genes (p-value 0. 5122). The similarity between the two distributions suggests that piRNAs in adult brain may not be implicated in massive mRNA deadenylation.

Supplementary Tables

Motif	Over-representation	Over-represented Motif E-value	TF Match P-value	TF
	BT Clusters' Promoters	4.9e-003	0.00497752	Eomes
	Non BT Clusters' Promoters	2.0e-002	0.00858316	Meis1
	Non BT Clusters' Promoters	4.5e-002	0.014478	Sox4
	BT Clusters' Promoters	3.7e-008<br>1.4e-004	0.00616759<br>0.0152929	Zic1
	BT Clusters' Promoters	3.7e-008	0.00909189	Zic2

Supplementary Table S1: Discriminatory motifs that distinguish between BT clusters' promters and non-BT clusters' promoters. These motifs were compared against

JASPAR vertebrate and UniPROBE mouse to find their associated candidate TFs. These TFs may regulate the transcription of BT piRNA clusters.
