# Supplementary material for: Characterization of piRNAs across postnatal development in mouse brain
Source: Sci Rep. 2016 Apr 26;6:25039. doi: 10.1038/srep25039 (PMC4844963; doi:10.1038/srep25039)
Supplement: Supplementary Table S2 [file srep25039-s2.pdf]

Gene	Day10_Expression	Day14_Expression	Adult_Expression	Average_miRanda_Score
Kcnq1ot1	1.64268	0.761137	1.91589	811.429
Xist	0	2.12644	0	1235.87
Slc8a1	11.9343	5.67051	6.29308	525.519
Aak1	6.55296	4.30521	3.83179	473.717
Strbp	44.4389	64.2434	45.5366	510.174
Plagl1	0.923247	0.761137	0.615324	1490.79
Fut9	11.423	9.46325	10.3105	465.385
Gucy1a2	7.80295	5.44691	7.02143	539.848
Lpp	3.15698	2.50701	4.35583	478.719
Synj2bp	21.9323	20.8676	13.8678	530.633
Gskip	1.5143	1.08938	1.71311	945.897
Ino80d	3.61965	2.12644	6.14615	476.731
Fzd3	9.15611	3.57917	5.81987	458.76
Ubn2	2.76974	0	1.57327	562.542
Tmem167	8.97431	6.74647	5.74375	415.875
Scai	8.40663	4.89934	4.19134	574.304
Gm16712	0.591053	0	0.615324	907.818
Fam163b	1.18211	0	0.615324	1098.09
Bicd1	16.4015	14.6784	11.9963	737.409
Homer2	5.06062	2.88758	1.57327	464.857
Nufip2	37.9943	24.7294	21.8818	511.55
Nfia	33.7869	10.5856	6.99141	1002.8
Hook3	22.389	20.1806	19.1753	496.35
Camk4	26.2621	14.5017	10.3891	480.85
Zyg11b	9.29864	2.81803	2.80392	544.368
Pou2f1	4.28404	2.50701	2.87384	450.105
Ntrk2	2.78026	2.55933	3.97891	603.316
Nav1	11.6106	3.59639	1.23065	986
Kcna2	1.58764	1.08938	4.01321	475.579
Gm16386	7.85108	4.15543	2.25852	1014.11
Zfp871	20.3847	14.8321	13.7222	560.444
Prlr	1.77316	0	1.57327	499.667
Peg10	2.06356	1.01984	0	722
Zfp781	18.2344	10.8602	12.124	943.059
Zfp26	6.16217	3.82718	0.957947	846.824
Tet1	0.923247	0.708814	1.84597	434.882
Map3k2	1.419	0	0.615324	435.824
Kctd14	0	0	0.957947	1954.41
Clmn	0	0.380568	1.30057	448.118
Usp15	55.4087	47.0682	48.083	450.125
Tyw3	5.03563	4.94863	7.03834	670.5
Tnrc6b	107.529	67.5604	66.5085	525.875
Steap2	3.0286	1.46995	1.84597	460.062
Slc1a2	28.0969	36.0927	29.33	459.625
Rc3h1	5.60504	2.02899	3.14654	486.812
Gm5089	7.72962	2.55933	3.54501	1068.31

Enah	4.04979	3.1986	3.68882	476.625
C1qtnf1	0.332194	1.08938	0.685246	1001
Slc4a8	18.0505	17.322	17.481	533.6
Itga4	2.10535	1.46995	0	495.333
Fam63b	0.332194	0.380568	0.615324	384.333
Bmpr2	18.7588	11.3699	19.7716	560.267
A230073K19Rik	162.163	109.434	154.728	628.067
Xpo7	2.76974	1.7982	1.55191	433.929
Tmem170b	0.332194	0.708814	0.957947	390.286
Tead1	0.923247	0.380568	1.10507	389
Sox11	1.93035	1.08938	0.342623	596.857
Pgap1	16.4027	8.99475	4.25161	423.071
Lrrc58	0.923247	2.12644	0.615324	431.643
Kcnb1	1.77316	0.380568	4.51703	475.571
Csrnp3	14.8219	6.03385	4.69845	527.429
Celf2	292.168	148.584	151.231	491.429
Zfp935	12.1827	7.64667	9.03828	687.308
Trub2	6.45828	5.32504	4.32154	1581.31
Rora	2.82111	1.46995	2.94376	414.231
Mesdc2	22.3657	10.9542	14.3007	512.154
Gm14436	10.9658	5.49923	6.16022	683.077
Dnajc28	2.51088	3.97696	5.31378	673.154
Dcx	67.561	26.2156	8.47283	429.462
Clock	23.1497	14.1818	13.2742	425.462
Zbtb44	1.18211	0	1.23065	579
Tug1	46.585	26.2756	19.886	371.417
Ttc14	2.76974	0.708814	1.30057	486.167
Trove2	5.70095	1.46995	2.09731	424.583
Tmod2	14.8838	11.6716	16.9444	501.5
Tbrg3	1.84649	2.12644	0.957947	407.917
Rufy2	11.2163	8.32119	9.72793	364.083
N4bp2l2	79.4543	33.3626	50.7375	583.833
Itsn1	4.35799	1.14171	3.3462	493.083
Hnrnpd	18.7452	8.33449	10.568	502.083
Hif3a	0	0	0.866667	617.083
Gpc6	5.47667	3.21582	1.57327	676.75
Gm19461	0	0	0.615324	945
Fam210a	6.01603	4.35753	5.71149	410
Fam199x	0.332194	0.380568	2.43994	471.583
Dnajc27	3.61965	4.58832	7.54528	515.583
Cadm2	5.14447	3.26815	0.615324	514.333
Zfp148	17.9177	5.34226	7.87948	481
Ubxn7	26.1734	18.6412	14.2722	461.455
Trdmt1	3.15698	2.10922	1.02787	724.636
Slc5a3	2.89812	0.380568	4.28956	549.364
Slc4a5	0.591053	0	0.957947	595
Slc24a2	18.8156	26.4732	22.5604	384.636

Sh3gl2	6.96901	5.01402	6.2018	889.364
Polr3e	1.64268	1.70074	0.615324	2744.18
Pappa	6.42198	4.62714	4.38194	535.091
Kantr	24.7121	10.7968	7.03671	719.455
Fgf9	7.49607	2.17876	4.35583	1291.27
Cdr1	27.9615	39.4039	57.2086	1001.64
Ankrd45	10.3003	8.27641	15.1691	670.182
Zdhhc21	4.53863	4.26008	1.57327	488.3
Wdr37	42.8256	19.6107	23.6866	735.1
Ttbk2	0.591053	0.761137	0.615324	390.4
Suclg2	0.591053	0.380568	1.10507	772.1
Smad2	12.7683	4.536	3.89925	420.7
Shisa7	0.923247	1.7982	0.957947	379
Rnf150	3.0286	2.83525	0.957947	390.4
Rgs17	7.59222	5.24481	4.09586	519.9
Rfng	0.332194	0	0.957947	850.5
Psd3	92.1432	75.7344	87.5378	476.4
Prex2	5.1992	4.61623	6.96425	411.4
Pank3	9.68527	9.06429	7.80956	489.6
Mid1	1.05163	0.380568	1.23065	855.2
Map3k9	0	0.708814	0.957947	388.7
Malat1	12896.7	23052.8	16667.6	410.5
Kdm7a	0.591053	0.761137	0	521.1
Hecw2	0	0	0.615324	451.6
Gabpa	13.2751	6.4358	5.4609	709.6
Fmn1	5.4024	4.92969	6.56995	451.1
Fam160a2	6.58987	2.12644	3.7956	439.1
Edaradd	0.591053	0.708814	0	484.8
Col19a1	2.98681	1.46995	3.39788	539.8
1700025G04Rik	3.95185	0.708814	3.63629	344.2
Zkscan8	3.39388	0.380568	0.615324	429.556
Zfp97	5.06807	5.17476	4.78973	563.222
Zfp600	0.591053	0	0.957947	607.778
Tsc22d2	0	0	1.30057	541.444
Thsd7a	2.10535	0.708814	1.91589	431.111
Tanc2	6.46273	6.38652	6.02038	475.222
Stxbp5l	9.37572	6.54022	10.2346	518.333
Stx8	8.17194	10.1328	12.0879	2425.44
Ssbp2	31.0127	23.8657	25.6962	414.444
Slc7a11	17.3313	11.894	13.5003	463.111
Setbp1	0.591053	1.08938	0.685246	535.111
Serbp1	77.614	45.8874	44.5319	458
Rgs8	6.90976	8.29819	8.22206	451.778
Rfesd	2.10535	2.88758	1.57327	475.778
Ptbp3	1.5143	0.708814	0.615324	560.667
Myo5a	0.996582	1.41763	2.40564	378.889
Msi2	5.68141	3.11837	4.30727	907.444

Lnp	20.0556	5.27687	11.1676	503.778
Kalrn	20.151	11.953	17.4876	343
Gm14440	25.017	16.6799	19.6952	774.889
Fgd4	46.8963	23.1599	20.777	378.222
Dnal1	22.1745	17.2689	18.5021	415.222
Cmah	0	0.708814	0	424.778
Cbx5	105.613	88.9472	71.6187	380.222
Braf	2.43755	2.12644	3.48916	448.333
Acvr1c	0.332194	0	0.615324	430.889
A830018L16Rik	13.7676	9.79752	10.5622	430.333
5930403L14Rik	0.591053	0	0.615324	517.667
2610507I01Rik	0.591053	1.85052	4.35583	867.333
Zbtb41	4.60782	3.36941	1.48199	465.5
Vldlr	2.23373	1.7982	0.615324	403.125
Trim66	2.43755	3.21582	3.05526	386.25
Tnfrsf22	1.5143	0.761137	0	791.25
Tmppe	1.71601	2.17876	0.342623	410.125
Tfcp2l1	0.591053	0.708814	0.615324	482.5
St8sia1	2.36421	1.46995	2.18859	634.375
Ssr1	57.7321	40.3475	37.2979	382
Slc30a7	4.99748	5.85617	5.81514	402.5
Sbno1	2.17869	3.05951	2.18859	478.625
Rab3c	49.5344	46.6539	41.5709	407
Qk	14.2893	11.5614	13.4139	403.25
Prkg1	8.58498	4.20775	5.20956	601
Ppp1r9a	4.85313	3.97696	4.44711	375.875
Phc3	7.11661	5.46576	7.40627	441.375
Pfkfb2	5.62761	5.90079	9.20001	738.625
Pde4d	0.591053	1.41763	0.615324	365.125
Nin	1.77316	2.12644	4.92259	380.5
Ncl	251.067	198.481	141.741	445.125
Mylk4	0	0.708814	0.615324	1011.62
Mrps25	112.493	88.7203	67.5191	402.5
Med16	1.16015	0	2.53122	628.25
Mapre1	70.5482	57.7055	34.6115	440
Hjurp	14.5609	7.93331	12.3509	400.875
Gm3414	4.5429	2.81803	2.33572	394.125
Gfap	6.31606	8.99126	8.81022	1099.62
Gas7	19.9281	13.645	18.6902	358.625
Fubp1	31.8986	19.5383	17.6917	610.125
Fam126b	5.77638	3.90741	8.65036	460.25
Dclk1	76.5562	61.9166	52.9008	437.375
Ctdspl2	47.9911	40.2497	27.5808	364.75
Creb1	13.2547	9.16945	7.2653	446.75
Cdk7	16.7497	8.09794	10.072	455.875
Cd300a	0.591053	0	0.615324	677.375
Ccdc50	14.2448	11.1672	11.6572	440.875

Agps	3.23032	3.21582	1.57327	483.25
Add2	16.7336	8.13483	8.20917	419.25
Adam22	4.50173	2.83525	2.87384	511
Acap2	14.0796	11.9495	10.6396	480.625
6720489N17Rik	5.73552	2.40956	1.7204	879.5
Zfp677	3.41584	0.708814	2.43994	471.143
Wnk3	9.15407	5.70561	9.98502	501.429
Ube2w	6.02348	5.70942	7.1372	372.286
Ttc39b	0.332194	0	2.33572	587.429
Syn3	3.52436	3.11837	4.48557	410.286
Srprb	4.08023	1.7982	3.05526	746.857
Shisa6	0	0.708814	0	340.857
Rnf152	1.18211	0.380568	0.615324	407.286
Rbms3	15.2597	5.91802	8.19983	425.571
Ralgps1	5.31371	1.46995	2.32844	414.571
Rab27b	37.97	34.9577	30.6835	405.286
Prrg3	6.77064	1.46995	0.685246	459.714
Prkca	3.28746	3.49894	6.9429	408.857
Pogk	2.21177	2.80081	4.77293	451.857
Plxna2	10.4865	4.03173	6.04193	434.571
Plaa	3.72607	1.41763	1.98581	359.857
Pikfyve	3.98493	1.85052	3.3066	406.714
Pigm	1.5143	0	1.23065	380.143
Pigk	8.80156	4.13054	6.5175	2458.57
Pde1c	6.98045	3.97696	7.83092	457.143
Pcdh10	128.522	93.4852	66.0644	600.714
Myo15	1.5143	0.380568	1.23065	344.571
Mrpl11	14.8205	8.07899	7.77386	636.857
Lrtm1	0	0	0.615324	385.714
Lin7a	20.8001	12.3199	11.4627	349.429
Irgq	3.54632	1.41763	2.18859	458.571
Inpp4b	17.2001	18.7322	20.7579	532
Igf1r	1.25544	1.14171	1.30057	386.714
Hif1an	70.7511	37.2004	33.4341	370.714
Gxylt1	0.591053	0	1.23065	414.429
Gpatch11	78.9671	65.4441	61.2853	1012.57
Gnao1	18.1493	10.5416	4.52432	1326.86
Fign	3.96236	3.11837	3.47236	432.571
Fbxo32	0.591053	1.41763	2.53122	361.714
Fbxl20	0.332194	0	0.615324	643.714
Faxc	9.52231	5.13966	8.9015	392
Fam198b	0.591053	0.380568	1.23065	478
Ept1	6.54961	3.17069	4.66226	469
Dusp11	13.875	8.28461	6.37521	456.571
Dcp2	3.39295	1.32017	4.57743	497.143
D430019H16Rik	6.4766	3.1986	0.685246	431.143
Cux1	62.2535	37.5539	34.7249	408.857

Cep97	3.57303	2.98884	4.77509	432.286
Car8	7.1189	5.67466	7.47167	507.857
BB014433	0	0	0.615324	1303.86
Asxl3	3.43413	1.08938	0.685246	455.571
Arrb1	5.07082	2.87035	5.33878	469.143
A830082N09Rik	4.46957	4.02928	8.24271	501.143
1700001L05Rik	1.5143	1.14171	0.342623	485.286
1200014J11Rik	8.22506	4.58832	4.33903	417.286
Zfp558	1.25544	0.708814	0.615324	671
Zfp113	2.10535	1.41763	1.91589	503.833
Whsc1	70.7058	36.4691	52.1135	368.5
Trps1	6.84787	3.87951	2.53122	420.5
Trim9	13.2151	6.18442	4.86694	356.833
Tns3	3.28746	1.08938	0	447.833
Tfdp2	3.2655	1.7982	2.18859	422.167
Tbl1xr1	2.51088	1.4004	2.60114	526.167
Tbc1d24	7.16395	7.2245	6.22808	679.167
Syt14	3.74803	0.761137	2.18859	370.333
Sv2c	11.2221	6.21049	7.70203	835
Specc1	6.96662	7.88682	7.82223	349.667
Socs7	0.332194	1.08938	0.342623	363.667
Sestd1	3.8947	1.08938	0	395.167
Sema5a	1.5143	0.380568	0.957947	394.5
Samd8	0.591053	0	0.957947	392.167
Runx1t1	9.86238	9.09224	6.22316	389.5
Rorb	7.91695	8.4148	8.5176	588.5
Rlim	40.2239	28.283	23.086	368.667
Rfx3	10.6001	8.51296	2.53122	445.167
Pten	0.332194	0.708814	0.342623	535.667
Prkcb	12.9228	8.89353	6.88648	448.833
Pqlc1	0.664388	0.761137	0	472.167
Poldip3	6.90501	3.59639	5.60803	605.333
Pes1	17.3764	14.4433	12.1015	1471.83
Pcnx	0.923247	0.708814	0.342623	373
Pcdh15	9.90196	5.37736	4.37719	389
Pak3	40.0967	20.5631	20.5151	425.167
Nova1	8.60117	6.76405	6.2841	481.5
Mrpl15	18.1798	7.61876	9.01378	444.333
Mlec	2.67445	2.23109	0.957947	386
Med13	10.3545	3.97696	7.38694	338.833
Mecp2	3.85655	3.85509	4.25526	583.5
Lyrm9	7.0673	3.21998	5.75989	371.833
Lifr	3.54632	2.12644	2.40564	508.667
Klhl24	13.0949	11.8625	10.2051	600.333
Hnrnpr	10.4204	7.8698	9.2404	420.667
Emc8	11.2938	5.82748	7.7939	364.833
Elf2	4.21071	0	3.83179	395.167

Dock9	40.248	20.2073	18.158	821.167
Dlg2	192.079	155.896	94.3163	311.167
Crebrf	8.17275	11.089	14.7244	455
Chic1	5.16704	6.41442	6.44021	479.167
Cdyl2	6.17414	2.70219	3.54501	419.5
Cdc40	20.5237	15.0883	20.4567	640.333
Cbfa2t3	1.25544	0.380568	3.29367	343
Camk2d	14.7786	10.7213	17.7991	373.833
Camk2a	80.0514	85.3947	109.453	340.833
Bcl2l13	2.10535	2.17876	1.02787	387.667
Bcl11b	37.8165	25.558	29.0177	449.167
AW549877	16.2295	12.0176	12.2624	397.833
Asb1	1.5143	0.708814	0	393.167
Arhgef12	9.14085	3.54407	6.6189	373.167
Arhgap11a	17.6666	4.9617	6.6357	1441.83
Aptx	7.12205	8.74375	3.451	1742.83
Amer1	9.17393	1.7982	2.53122	474.667
4931428F04Rik	7.82826	2.55933	2.74827	1294.83
2900097C17Rik	3.00664	2.50701	4.44711	360.5
Zfp953	3.03972	0.380568	2.33572	405
Zfp937	3.2655	3.59639	3.41924	470.4
Zfp706	8.68115	3.82718	3.93544	373
Zfp266	10.6986	5.01402	7.71886	402.2
Xpr1	15.2374	9.30616	10.9443	409.4
Wbscr25	0.664388	0	0.615324	392.4
Uggt1	11.8255	6.75989	8.53696	337.8
Trub1	22.4482	14.0737	11.3842	439
Trp53bp1	22.8417	13.3736	13.1606	346.4
Tpgs2	3.42268	3.97696	4.06177	474.4
Tomm20	7.61932	5.60816	8.27496	403.8
Tmtc1	2.43755	0	0.615324	406.2
Thsd4	6.24487	3.83787	5.19982	340.6
Tardbp	24.9806	16.3598	16.1572	373.4
Syncrip	51.2169	16.7917	32.4563	480
Stx2	0.332194	0.380568	0.615324	698.2
Stox2	8.51164	4.63345	4.03457	444
Srgap3	49.4577	27.818	41.0316	371.4
Spg21	2.23373	0	1.23065	798.2
Snx27	1.7512	0.380568	3.90899	408
Slitrk2	8.15462	3.59639	7.87872	594.8
Slc38a9	4.79966	2.50701	1.84597	375
Slc30a4	0.591053	1.08938	0.957947	409.6
Seh1l	18.1437	16.069	11.9644	534.4
Rnmt	21.3005	15.5017	17.0534	315.4
Rnf130	1.84649	1.08938	0.685246	407.2
Rgs9	22.8444	13.8967	23.3028	607.6
Rbfox3	19.9045	19.865	10.4172	209.6

Raph1	0.996582	1.46995	0	348
Rap1gap2	10.46	7.19659	11.8981	334.2
Rabgap1l	84.2682	49.542	63.76	408.6
Pyurf	4.74462	3.60359	2.16724	340.6
Ptpn2	6.35599	3.42939	4.74136	466.8
Prickle2	48.3032	28.6538	33.9566	343.8
Plcxd2	13.8475	5.44936	6.54049	442.2
Pde7a	2.43755	0.708814	0.957947	437.4
Pde10a	3.0286	3.59639	3.76186	413.8
Pcdh19	4.46747	0	1.23065	434.2
Orc4	127.732	108.545	88.2403	310.4
Ogfrl1	0.923247	0.761137	0	472
Nr6a1	2.82479	2.88758	7.45381	469.2
Ncan	11.9967	5.33596	2.94257	531
Nab1	2.17869	1.46995	0.685246	438.6
Mrpl19	7.46477	6.81847	5.10924	469.4
Mrpl17	15.9981	9.65711	8.24743	475
Miat	10.0845	3.90741	2.53122	432.6
Mgat4a	6.54658	5.83895	4.71981	377.4
Magt1	3.76632	0.761137	3.40244	505.2
Lrp6	2.64978	0.708814	0.957947	363.4
Lcorl	6.78651	3.21582	5.13964	416.2
Kcnd3	4.96532	1.7982	5.26521	375.6
Kcna1	0.591053	1.08938	0	341.2
Il17rd	1.419	0	0.615324	401
Ifnar1	3.48917	4.58832	2.43994	428.2
Gpr146	0.664388	0.380568	0.957947	661.8
Gpalpp1	29.2769	27.3613	25.636	437.4
Gna13	0.332194	0.380568	0.957947	438.2
Gmnc	1.97487	0.380568	1.57327	373.2
Gm608	0.923247	1.08938	0.957947	380.8
Gm20554	0	0.708814	0	405.8
Fam212b	2.56593	1.14171	6.85086	370.8
Fam168a	48.5452	32.1384	29.6525	337.2
Exoc8	0.923247	1.08938	0.957947	337.2
Etnk1	2.10535	2.12644	3.48916	402
Elac1	0	0	1.57327	371
E330033B04Rik	13.7006	3.57917	6.65726	301.8
Dpy19l3	0.923247	1.41763	2.18859	492.8
Dcc	7.39953	6.81221	0.957947	309.6
Dcaf17	13.9062	8.29363	7.99761	538.4
Dcaf10	0	0.380568	2.53122	439.4
Ctdsp2	2.17869	0.708814	2.18859	493.6
Cpsf6	16.9499	11.2599	6.72905	570.6
Cpne5	2.69641	1.7982	1.23065	437.4
Commd7	6.86594	2.23109	8.87111	402.2
Cnot6l	1.18211	2.79012	2.09731	530

Ccdc127	15.1199	17.2835	15.2183	369
C77370	2.36421	1.72865	0.957947	435.6
Bbx	4.37125	2.17876	6.51013	437.6
Atxn1	40.022	19.7828	23.6576	404
Atrn	0.664388	0	0.615324	501
Asph	28.5212	24.2988	27.085	411.8
Arhgap19	3.98493	2.55933	0.342623	969.2
Apbb2	19.3604	11.0578	16.9162	374.2
Ap3s2	26.812	14.6485	17.8643	407
Ankrd33b	0	0.708814	0.615324	371
Anapc1	15.7534	13.3597	7.73551	307.4
Amotl1	0.591053	0.708814	0.615324	373.4
Ago2	0.591053	0	1.10507	436.6
Aff4	7.06491	5.34607	5.00678	405.8
Abl2	2.10535	0.708814	0	602
A230056J06Rik	6.22077	3.52684	4.03457	440.4
9430037G07Rik	0.591053	0.380568	0.615324	380.4
5031426D15Rik	15.506	6.43165	10.9432	376.6
1500004A13Rik	77.9838	60.8457	55.7173	412.8
Zfp951	4.21071	4.13054	1.91589	570.75
Zfp945	27.2824	17.4519	19.0032	344.5
Zfp882	7.36559	4.44807	5.26521	386
Zfp811	6.7033	3.21582	2.18859	428.25
Zfp563	2.60111	1.41763	0.957947	389.25
Zfp398	1.25544	2.17876	0.866667	300
Zfp329	4.0357	5.49367	3.93544	376.75
Ythdf3	4.02518	1.7982	3.14654	415.75
Wisp1	0.591053	0.708814	0.615324	346
Vps26b	9.01037	3.54407	2.25852	392.25
Vma21	11.475	4.599	10.1925	470.75
Usp45	13.2761	11.1834	10.6056	428.25
Unc5d	0.664388	0	0.615324	503.75
Unc5c	16.7501	6.21952	6.20636	346.25
Uhrf1bp1	0.591053	1.08938	1.57327	878.75
Ubr1	29.2926	17.5746	14.1623	338.25
U2surp	50.1149	46.2741	47.533	461.25
Trim2	40.539	20.3935	22.2985	463
Traf3	0.332194	0.380568	0.957947	385
Tmx4	13.0047	6.21182	14.471	420.5
Tmx3	11.6061	6.00595	8.22526	343.75
Tm9sf3	3.11245	0.380568	3.74506	422.75
Thrb	5.87168	8.13239	6.32681	343.5
Tet3	1.5143	1.7982	0.615324	382
Tcf4	93.0792	51.0389	40.3175	418.25
Tceanc2	9.74512	1.41763	3.7956	381
Sypl	2.17869	2.55933	1.98581	430.75
Styx	1.64268	0.708814	0.615324	384.5

Stx17	5.60179	3.03044	5.89291	468.75
St8sia6	0	0	0.957947	419.75
Ss18l1	6.60598	5.61554	5.94011	262.75
Srrm4	8.30965	4.7381	6.92078	393.25
Spry4	1.64268	0.708814	0	341.75
Spock1	0	0.380568	0.615324	425.75
Sox6	22.2683	15.6559	11.3545	344
Smcr8	3.29858	2.50701	5.45243	424.75
Slc7a14	5.22116	3.17069	5.86373	379.5
Slc46a3	1.18211	1.08938	0	504
Slc35f1	6.08429	2.55933	1.94962	430.75
Sh3pxd2b	1.7512	0.708814	0.957947	259.5
Sft2d2	0.591053	0.708814	1.91589	422.25
Sept6	3.0286	3.97696	1.84597	414.5
Sept11	34.6978	43.6951	23.935	383.75
Sephs1	1.419	0	1.10507	342.5
Sec14l3	6.36798	0	0.615324	343.75
Scn8a	8.75151	4.68577	9.57658	337.25
Satb1	12.63	7.24511	4.97115	421.25
Runx2	2.95527	0	0.615324	376
Rreb1	1.5143	1.08938	3.07662	484
Rpusd2	0	0	0.615324	422.25
Rps6ka3	3.44465	1.7982	1.98581	382.25
Rnf24	1.18211	0	0.957947	541.25
Rnf165	0	2.17876	0.957947	1073.75
Rnd1	4.84382	2.50701	0.615324	350.5
Rgs7bp	3.28746	1.41763	1.91589	426.25
Rcan3	0.664388	0.708814	0	426.5
Rbm33	5.32968	2.48979	4.36856	418.75
Ralgps2	16.2354	8.43491	6.34978	342.75
Rad51d	4.79966	2.23109	3.41924	419.25
Rab8b	38.6782	34.6294	28.8159	542.5
Pvrl1	0.332194	1.08938	0	382.5
Ptprb	1.84649	1.32017	1.82461	333.75
Prrc2b	2.30707	1.72865	1.30057	375.5
Prr14l	12.5347	5.9085	9.28787	413.5
Plekhb2	39.8757	25.5469	36.3842	385
Plekha8	1.18211	0	1.30057	335.75
Plekha3	0.332194	0.380568	1.57327	587
Pik3ca	5.46615	6.3621	8.06913	463
Pi15	3.15698	1.01984	0	384.75
Phf6	24.0946	10.1227	6.67019	632.5
Phf14	10.4206	4.43854	4.04883	630.25
Pgpep1	1.84649	1.85052	1.23065	369.5
Pgm5	1.18211	0	1.23065	424.5
Pgm2l1	16.5199	10.1781	6.70563	421.75
Peg13	31.7239	18.5403	33.286	424.25

Pdzd2	9.89546	3.54407	3.90899	419.75
Pdcd6ip	0.923247	1.46995	1.48199	382.75
Pbx1	11.5886	3.21582	4.03457	415.5
Pard3b	1.419	1.41763	2.06302	335.5
Paqr8	3.43413	3.57917	2.60114	375.75
Pank1	1.84649	0.761137	4.10449	373.75
Palm2	2.17869	0.380568	0.957947	423.5
Pafah1b1	13.699	11.5339	12.3987	379.25
P2rx7	6.08181	3.59639	3.32796	467.5
Osbpl3	3.28746	1.08938	2.80392	373
Nwd1	8.98241	6.97346	9.75913	292.5
Nufip1	4.46957	2.55933	2.18859	586.25
Nucks1	13.3617	5.92874	6.12403	674.5
Nr2e1	0.923247	0.708814	1.64319	1013
Nqo2	6.45432	3.85509	4.9748	294.5
Nkain3	4.35738	1.08938	2.05574	346.75
Nfib	24.7008	11.7288	4.44711	378.5
Nap1l1	26.4614	18.5163	18.1138	375.5
Mysm1	35.6216	24.4934	25.5355	461.5
Mxi1	9.04463	6.73881	7.18475	298.75
Mxd1	0.996582	1.52227	0.615324	459.25
Mpeg1	0.591053	1.41763	1.94962	387.5
Mlph	0	0	0.957947	1373
Med28	4.37427	1.85052	3.97891	464.25
Mbp	232.175	385.041	255.836	338.25
Mapt	37.2094	10.5699	10.1635	461
Man1a2	20.7566	15.8914	21.9128	496.25
Lsm11	3.2655	0.708814	0	300.25
LOC102634431	1.77316	1.7982	2.18859	530
Lmbr1	2.82479	2.40956	1.23065	342.75
Lig3	5.69247	1.78097	5.11772	344.75
Larp1	1.5143	0.380568	2.78256	392
Kras	4.53146	1.7982	3.20239	428
Kdm5a	2.10535	1.08938	2.4613	343.25
Kctd12	0	0.380568	0.957947	420.5
Katnal1	7.38091	7.9016	16.3074	375
Itih5	8.67372	2.88758	5.42661	380.75
Insr	1.84649	0	1.98581	379.25
Ids	12.13	8.49573	12.263	382.25
Hlf	4.85313	1.46995	4.99251	464.25
Hhip	7.45489	12.2574	9.15555	421.25
Gyk	5.11199	1.46995	5.01406	384.25
Gspt1	2.56593	0.380568	0.957947	347.5
Grin3a	15.3992	3.92464	3.14654	507.5
Gm12992	0.332194	0.708814	0.342623	337.25
Gas2l3	2.17869	0.708814	0.342623	392.5
Ftx	19.3046	12.279	22.0665	351.75

Fsd1l	35.6867	13.6595	21.5535	664.25
Frrs1l	3.82137	2.17876	2.71264	338.5
Foxp1	5.92672	2.08131	1.02787	380.75
Foxk1	0.923247	0.761137	0	299.25
Fosl2	3.23032	0.380568	3.05526	501.25
Fn3k	3.69299	7.14046	8.40808	382.25
Fbxo42	1.84649	0.708814	1.57327	342.5
Esrrg	2.60111	2.12644	2.4613	345
Epm2aip1	7.21735	5.92874	3.55909	338.5
Elmod2	10.8665	4.66855	2.87384	423
Eif5a2	3.31417	2.37395	3.51072	602.25
Dynlt1a	4.51759	1.08938	0.342623	343.75
Dnlz	5.29752	1.46995	0.957947	383.25
Dennd5b	0.591053	0.380568	2.4613	376.25
Dennd1b	1.18211	2.12644	1.89454	391.25
Dcaf7	16.0214	4.536	4.25161	461.25
Dap3	12.4328	7.34545	7.51726	384.25
D830031N03Rik	1.84649	0	1.30057	382
D630041G03Rik	3.10194	0	0.615324	456.25
Crtc1	19.1389	11.712	17.3299	417.75
Cntn2	1.77316	0	1.55191	386.5
Cnr1	3.61965	1.7982	4.78973	415
Clic5	0	0.708814	0.615324	376.5
Chrdl1	1.38382	1.7982	1.97174	375.25
Chid1	9.83622	8.65698	8.06913	347
Celf1	3.43413	3.59639	1.57327	596.75
Cdv3	3.0286	1.7982	1.91589	377.75
Ccnd2	7.24709	1.4004	3.69194	383
Ccdc116	0.591053	2.12644	0	390.25
Cbfa2t2	1.85701	1.46995	0.615324	415.5
Camk1d	3.30365	0.708814	1.73333	460.5
Cacul1	0	1.41763	0.342623	462.75
Cacna1c	4.21071	1.08938	0.957947	380.25
Bri3bp	0.996582	0	0.685246	510.25
Bcas3os1	0	0	1.23065	1732.25
B3galt5	0.664388	1.08938	2.78256	544
Arl5a	1.77316	3.14628	0.685246	421.75
Arhgap20	0.591053	1.7982	0.615324	372
AI593442	4.21071	5.62538	2.53122	505.25
Acad9	4.63011	2.7378	2.67834	380
A830010M20Rik	24.7372	22.8121	24.3021	502.5
A430105I19Rik	1.05163	1.41763	0.957947	301.5
9830147E19Rik	4.64932	1.41763	1.23065	540.25
4933432I09Rik	0.923247	0.708814	0	761.75
Zzz3	6.3873	5.85617	5.53538	286
Zxdb	0.664388	1.08938	0.615324	340.333
Znrf3	1.5143	0	1.84597	491.333

Zmiz1	9.35368	3.21582	6.09759	391.333
Zfp84	13.4913	5.82409	9.45283	444.333
Zfp748	21.885	8.71792	11.7726	397.667
Zfp719	8.98841	3.59639	2.95104	347.333
Zfp462	23.0611	16.3373	18.2708	556.333
Zfp446	0.591053	0	0.615324	341
Zfp316	2.72857	0	1.23065	392.333
Zfp275	3.28746	1.08938	0	448.667
Zfp174	2.36421	1.72865	0.685246	397.667
Zfp111	3.17527	0.380568	1.7204	290.667
Zdhhc15	3.3608	4.20775	1.84597	287.667
Zcchc8	19.1235	12.3576	8.35108	440.333
Zbtb4	1.18211	0.708814	0	390.667
Zak	0.923247	0.380568	1.30057	348
Yy2	0	0	1.57327	659.667
Ypel2	3.57848	2.17876	4.82819	387
Wwp1	0	0	0.957947	293.333
Wipf2	6.32626	6.49116	5.71225	445
Wdr7	8.43044	5.88408	4.50751	334.333
Wdr43	12.8046	8.68788	13.8034	335.667
Wdr13	15.6448	8.99098	10.5306	350
Wars2	10.174	5.01783	5.7425	341.333
Vapb	5.46405	1.7982	3.57931	396
Usp9x	3.0286	2.17876	1.82461	399.667
Usp31	3.13357	1.61281	2.93983	443.667
Ufm1	11.2723	8.32107	8.87306	548.667
Ube2v2	7.1602	3.97696	1.57327	389.667
Ube2g1	11.836	3.49894	5.31378	343.333
Tyms	3.83188	1.41763	3.14654	604
Txndc9	29.2637	13.427	15.4935	337
Tub	0	0	0.685246	349.667
Trp53inp1	0.664388	1.46995	0.615324	446
Tor1aip2	11.6476	9.6776	13.4549	336
Tnpo2	3.48917	1.08938	3.29367	342.333
Tmem88b	4.86639	5.62782	13.332	464
Tmem63a	0.923247	0.708814	3.41924	287.333
Tmem18	3.87851	4.88096	1.84597	391.667
Tmem106b	32.7308	23.6864	22.7328	396.333
Thoc2	43.7908	23.4519	28.1747	494.333
Thg1l	4.7798	2.17876	3.76186	340.333
Tceanc	0	1.08938	0	389.667
Tbc1d4	0	0	0.957947	342
Tbc1d32	23.4264	16.3758	11.2002	347
Tbc1d30	2.43755	0.380568	3.14654	395.667
Taf1	25.8874	16.5017	9.23122	390.667
Tacc1	5.00348	6.22904	6.79577	395.667
Stxbp6	3.0286	1.32017	2.80392	282

Stxbp5	0.923247	0.380568	1.20929	389.667
Strn	2.76974	3.26815	3.55909	394.333
Strip2	5.83092	6.41687	6.41492	503.667
Stk35	0	0	0.615324	443.667
Stat1	1.32878	1.7982	5.82488	446.667
Stam2	6.47752	8.64928	5.83782	288
St6galnac3	5.73613	0.761137	6.51741	444.667
St3gal1	5.23892	4.73471	1.84597	455.333
Sspn	0.591053	1.4004	0.615324	461
Ssh2	6.90712	3.59639	6.29308	445
Srpk2	17.1051	11.0974	13.5433	349
Srgap1	4.61624	0.708814	1.82461	447
Spock2	39.7314	27.1435	41.0849	393.333
Spin1	12.9156	5.98153	3.02097	355
Sorcs1	1.84649	1.41763	2.18859	340.333
Smarcal1	8.30206	7.98261	8.17214	334.333
Slc9a5	2.36421	1.7982	0.342623	393
Slc7a2	1.64268	1.08938	1.7204	395.333
Slc43a2	4.09345	1.08938	5.13964	386.333
Slc31a1	7.11871	5.77896	1.98581	393.333
Slc2a9	1.25544	0	0.615324	342.667
Slc2a13	0	1.08938	0.342623	345.667
Slc25a44	11.1712	7.22097	7.77195	613.333
Slc23a2	10.8922	4.40294	6.03346	472
Skil	13.0941	7.72137	5.42216	398.333
Shroom2	35.7297	18.0285	12.2081	388.667
Sh3bgrl2	2.59777	4.05027	4.33447	458
Sgk3	3.82137	4.23566	3.19966	341
Setd7	9.83707	10.0793	10.4846	333.667
Serp1	6.22077	3.42939	1.94962	343.667
Sema3a	6.9402	5.01402	4.44711	286.667
Scimp	0	0	0.615324	689
Scd1	18.0968	7.97768	13.2262	396.333
Scamp1	27.8712	20.0664	25.9164	398.333
Sbk3	0	0	0.615324	557
Rsf1	2.84308	3.41101	2.80392	342
Rps6ka2	1.38382	1.08938	1.98581	569
Rps15a	1.85701	2.28002	1.4477	345
Rnf4	17.4553	15.2829	11.8048	336.333
Rnf170	2.30707	1.46995	0.957947	289.667
Rnf169	0	0.380568	0.615324	290.333
Rmnd5a	1.18211	0.708814	0.342623	391
Rictor	16.6118	8.03383	10.8237	450
Rhoh	0.591053	0.708814	0.342623	396.333
Reep3	3.96236	3.95974	1.57327	714.667
Rcan2	20.8263	22.8231	39.957	395.333
Rbpj	8.37061	8.1701	6.82188	392.333

Ranbp10	1.64268	2.17876	1.94962	396.667
Rab11b	5.75442	3.83787	5.35669	335.667
Pum2	10.8481	10.7396	4.18678	340.333
Ptrh2	7.86406	2.81803	4.57685	348
Ptprt	0.332194	0.761137	0.342623	337.667
Ptpn4	4.21071	0.708814	1.82461	392.667
Ptp4a3	0.923247	0	1.57327	350.333
Prrx1	3.15698	1.7982	0.957947	352.667
Prps1l3	9.18252	4.94447	8.93326	395
Prpf19	13.2123	8.47735	12.2606	446.667
Prkd3	3.28746	0	4.17441	336
Prkaa2	4.35738	3.32047	2.06302	343.667
Preb	3.61965	3.70104	2.80392	391.333
Prdm16	0.923247	0	0.615324	392
Ppargc1a	9.98202	11.2259	12.9361	287
Pofut1	0.664388	1.01984	0.615324	447.667
Plekho2	2.5214	0.708814	3.72255	447
Plekha2	0.332194	1.32017	2.78256	280.667
Pkib	10.2277	2.81803	8.84689	388
Pja2	228.11	189.085	202.026	445
Pik3r1	53.3897	29.3545	39.47	397
Pign	9.63718	3.49894	4.43304	338
Phex	1.18211	0	0.615324	403
Phactr2	6.72159	4.86424	2.43994	452
Pds5b	16.8546	10.4113	10.5704	299
Pdpn	8.68082	3.41101	1.64319	342.333
Pdk1	1.18211	0.380568	1.23065	338.667
Pdik1l	6.48166	3.49894	4.75157	286
Pcyt1b	12.4857	7.75182	9.08293	396.667
Pcmtd1	25.4589	22.8051	43.1844	559.667
Pank2	0.591053	2.50701	0.957947	509.333
Otud7b	0.664388	0.708814	0	400.333
Osbpl6	5.00348	6.1034	4.10449	451.333
Oprk1	0.996582	1.7982	0.615324	336
Opcml	55.5969	36.9908	34.9366	391
Ociad1	59.9747	36.9553	53.1246	392.667
Nxpe3	1.16015	3.49894	4.10449	410
Nup50	10.9502	6.12281	5.82747	285
Nt5dc3	0.591053	1.32017	0	397
Nt5c2	4.80176	1.7982	3.76186	336.667
Nr3c1	8.29094	7.58482	2.80392	389.667
Npsr1	1.5143	0.380568	1.57327	393.333
Npr3	6.21721	5.39703	4.03821	289.667
Nkain2	18.7164	14.8123	21.7265	392.333
Nipal2	0.923247	0	1.57327	344
Nid1	4.16678	1.14171	0	394
Nfasc	23.1772	32.494	10.272	338.333

Nars2	2.51088	1.08938	1.57327	344.667
Myo6	8.02306	4.30765	4.71981	393
Mul1	5.62971	3.26815	3.14654	337
Mtf1	8.42806	5.85617	10.647	396.333
Mon2	1.18211	0.708814	1.57327	345
Mmgt1	25.774	15.6361	11.1903	333.333
Mme	5.72501	1.7982	4.8383	385.333
Mid2	0.923247	0.708814	2.18859	333.667
Mical2	0.923247	1.08938	0.615324	295.667
Mgat3	1.25544	3.97696	1.57327	292.667
Mga	14.6883	11.3638	13.2478	392.667
Megf10	1.18211	1.7982	2.80392	334
Mau2	0	0	0.615324	341.667
Map4	7.00776	1.46995	5.53083	614.333
Map1b	78.6872	48.1541	39.7569	448
Mal2	5.44419	6.63755	5.94147	346
Lypd6	4.15356	2.43746	0.615324	382
Lrrtm2	25.9674	18.8528	26.0062	342
Lrrc75b	0	0.708814	0	508.667
Lrrc39	0	1.72865	0	340.667
Lrch3	0.591053	0	1.91589	450
Lipt2	0.591053	0	0.615324	329.333
Lbh	9.34212	10.4019	9.58822	386.333
Larp4	26.2225	20.7135	14.3593	450.667
Klhl18	5.43781	2.99222	3.92193	390.333
Klf8	1.18211	1.7982	1.57327	394
Klf13	2.23373	0.380568	0.615324	453.667
Kif3c	1.77316	1.4004	0.342623	449.667
Kif3b	7.52813	3.74997	0.615324	396.333
Kif1b	2.76974	1.08938	1.30057	382
Kdsr	6.77664	4.58832	1.82461	455.333
Kctd12b	3.15698	3.54407	0	287.333
Iws1	2.17869	2.50701	1.57327	560.667
Ivns1abp	12.8683	9.87676	4.45076	237.333
Itpripl1	3.5024	1.32017	0	388.333
Itch	18.5173	6.34488	10.0378	288.667
Irs1	0	0	0.685246	451.333
Ireb2	0	1.41763	1.64319	412.667
Iqce	9.19426	5.82056	3.57931	458
Intu	0	1.08938	1.23065	384.333
Igsf3	6.57282	1.08938	2.53122	282.667
Ighmbp2	2.36421	1.46995	1.84597	505.667
Igf1	20.3874	8.10797	5.97201	393.333
Ifit2	5.51245	2.02899	4.62853	447
Ifih1	0	0	0.957947	345.667
Hspbap1	2.44806	1.08938	3.86552	336.333
Hspa13	4.94002	1.14171	1.57327	397

Hs2st1	5.47635	2.02899	1.91589	402.333
Hpcal4	6.77664	3.64871	2.51441	452
Homez	0	1.08938	0.957947	400
Hip1	3.2655	2.17876	1.57327	400.333
Hdgfrp3	5.93994	3.21582	4.32154	388.333
Gucy2e	0	0	0.615324	350.333
Gria2	273.449	184.199	223.817	344
Grb10	2.80283	0.380568	2.09731	292.333
Gprc5b	0	0.380568	0.685246	346.333
Gnai1	7.06694	4.44807	4.26455	338.667
Gmeb2	1.7512	1.41763	2.74827	340.667
Gm9079	1.18211	0.708814	1.10507	398.333
Gm21276	0	0	0.615324	341.333
Gm20597	0	0.708814	0	345.667
Gm18853	0.591053	0.708814	4.17441	454.333
Gm17066	20.6239	8.55809	16.6979	454.333
Gm16702	0.591053	0	0.615324	335
Gm16523	3.54632	0.761137	2.33572	391.333
Gm11190	2.43755	1.41763	0	495.667
Glcci1	7.31264	1.08938	9.881	344.667
Galntl6	28.5526	15.1754	13.7684	337
Gabrb2	3.61965	6.26465	5.66095	334.667
G3bp2	41.604	32.6039	41.0826	447
G2e3	11.794	9.55684	4.77566	450
Fundc2	1.97487	1.14171	0.866667	393.667
Fstl1	11.516	7.08879	5.71225	395
Frmd4a	42.3651	20.8523	12.3207	341.667
Foxred2	0.332194	0.708814	1.23065	341
Fnbp1	19.4129	6.96918	12.8998	406.333
Fgf1	3.52436	2.54211	5.58648	335.667
Fgf12	61.7844	47.7633	70.5742	283.333
Fam78b	2.76974	0.708814	0.685246	292
Fam53c	2.36421	1.41763	0.615324	591
Fam206a	7.94762	2.12644	5.2225	290
Fam120c	5.05852	1.41763	3.43232	285.667
Exph5	2.36421	3.21582	12.0875	394.333
Evi2a-evi2b	5.00409	9.75464	34.9572	281.333
Ero1l	2.10535	1.41763	2.18859	439.333
Ermap	0.923247	0	1.23065	346
Ercc4	2.85359	1.46995	0.957947	445.333
Erc1	3.17527	3.92464	0.342623	339
Entpd1	6.2779	5.70942	3.02097	351.333
Emc1	4.01709	3.17069	4.09586	348.333
Elovl6	4.57598	2.88758	2.92969	341.333
E330020D12Rik	0.923247	1.32017	0.615324	288.333
Dynll2	6.00006	3.57917	5.80353	346.333
Dtx4	30.8803	20.6372	20.3555	389

Dnajc1	0.591053	0	0.615324	337.667
Diexf	6.90501	8.71933	5.13964	345
Dido1	24.205	14.2079	14.974	347.333
Diap2	9.93056	3.49894	8.93211	507
Dcun1d3	4.57598	0.380568	1.23065	502.333
Dach2	0	0.708814	0	343
D630045J12Rik	0.332194	0	0.615324	344.333
D430041D05Rik	3.43413	1.7982	1.30057	564.667
Cyp26b1	2.06418	0.708814	0.342623	331.333
Cyld	4.8751	8.03796	9.20596	391
Cxxc4	4.99203	0.380568	1.23065	341.667
Cxcl12	5.00409	5.64423	1.94962	398.667
Ctps2	18.1004	7.75182	11.9006	387.667
Crk	2.95527	0.708814	0.342623	399.333
Cpeb3	4.83484	1.46995	3.83179	611.333
Coa5	9.61761	3.26815	5.88054	445.667
Cngb3	0.827951	0	0.615324	459.667
Chst11	0.591053	0	1.57327	447
Chml	9.86028	6.1034	5.33513	335
Chd5	47.5732	33.293	34.8681	347.667
Cfl2	9.42767	6.36929	5.17973	387.667
Cep83os	3.87851	2.81803	4.23026	456.333
Cecr2	0	0.708814	0	338.667
Cds2	6.22077	7.65437	9.96367	678
Cdk19	7.20677	3.95974	9.79419	342.667
Cdh4	0	0	1.02787	387.667
Cdc27	15.2103	8.01001	7.66842	286
Cdc14b	1.18211	0	1.57327	458.333
Cd93	4.33909	2.17876	0.957947	333.333
Cd2ap	0.591053	0	0.615324	458
Ccser2	18.7927	5.72283	8.29912	292.667
Ccdc85b	2.10535	2.08131	1.30057	399.333
Ccdc71	0.591053	1.08938	0.342623	519.667
Ccbe1	1.77316	1.41763	1.84597	340.667
Cask	3.3608	3.92464	1.4477	509.667
Cacnb4	22.3185	22.0925	26.517	494.667
Cacna1e	27.6176	15.8766	14.5283	396.667
C3ar1	0.923247	0	1.30057	395
Btbd9	3.1702	1.7982	2.18859	559
Bsn	1.5143	1.41763	0.615324	349.333
Blcap	4.9998	1.01984	3.81771	352.333
Birc5	3.3608	1.32017	0	387.333
Bhlhe41	34.4101	24.3083	53.0622	395.667
Bcl2	3.18882	2.48979	1.84597	442.333
Bcat1	8.65831	5.49923	10.1464	389
Bbs1	7.16387	2.88758	4.44711	336
Bag3	0	0.708814	1.7204	516.667

Baalc	3.78322	2.12644	3.25019	396.333
Azi2	5.55638	4.30521	0.615324	341
Atp8a1	7.23721	4.17214	3.46781	351.333
Atp2b4	32.9581	24.8847	19.8829	394.333
Atf2	38.6684	25.7653	27.7765	457
Atad2b	0.923247	1.14171	0.685246	392.667
Arl11	0	0	1.84597	440.333
Arih1	1.91983	0.761137	1.23065	451
Arid5a	2.00671	1.32017	0.615324	499.333
Arhgap28	0.591053	0.708814	0.342623	440.333
Arhgap25	1.5143	2.50701	2.67834	235.667
Ap3m1	11.1069	9.74114	6.72599	510.333
Antxr1	0.332194	0	0.957947	338.333
Ano5	3.3608	0.708814	1.7204	509.333
Ankfy1	15.6577	11.5593	15.1643	455
Alg10b	0	0	0.615324	394
Aldh5a1	0.591053	0.380568	1.23065	340.667
Akap5	27.3618	22.7774	17.0409	333.333
Akap11	15.7155	12.2358	19.3003	388.333
Aebp2	1.7512	0.380568	1.30057	509.333
Adamts5	0.591053	0	0.615324	287
Actr1a	55.9505	36.9795	31.4559	282.333
Abhd13	0.827951	0.380568	1.91589	446.333
Abca5	5.73552	1.85052	2.87384	503
Aasdhppt	33.0381	28.5901	17.6403	464
9330158H04Rik	3.59769	1.41763	1.57327	394
6330416G13Rik	0.591053	0.708814	0	435.333
5330413P13Rik	0	0.708814	1.57327	240
4833422C13Rik	0.332194	1.46995	0.342623	345.333
2810417H13Rik	3.24628	1.32017	0	415.667
2610002M06Rik	10.9864	4.82864	4.77566	555.333
2510009E07Rik	2.10535	1.08938	1.64319	493.667
2310022B05Rik	1.5143	0	0.957947	340.667
2010111I01Rik	10.8728	8.99342	9.34778	334
Zzef1	2.43755	0.708814	0.342623	500.5
Zwint	240.28	195.482	190.329	261
Zscan26	17.3481	17.0921	18.9952	418
Zrsr2	22.7825	11.6385	16.5296	339
Zmym2	8.98748	5.75793	6.81459	586
Zfyve20	14.7846	11.511	14.3236	338.5
Zfx	5.53948	2.79012	2.18859	517
Zfp955b	11.4535	8.16749	5.78672	341.5
Zfp9	1.84649	1.08938	0.342623	340
Zfp866	12.1878	5.27991	6.19318	501
Zfp850	2.23373	0.708814	1.84597	421
Zfp831	0	0	0.615324	415.5
Zfp799	8.47703	6.43545	11.5476	436

Zfp78	0.923247	1.32017	0.866667	412.5
Zfp773	4.37093	2.12644	0.615324	342
Zfp738	14.1801	7.92978	11.5453	427.5
Zfp68	12.64	11.5167	5.13577	340.5
Zfp521	13.3782	8.95695	7.5193	432.5
Zfp518a	2.10535	0.380568	0.957947	499
Zfp488	0.591053	1.7982	0.342623	414.5
Zfp422	5.82543	2.55933	3.25019	334.5
Zfp354c	7.31326	1.7982	1.64319	339
Zfp281	0.664388	0.708814	0	262.5
Zfp280b	0.332194	0.380568	0.685246	344.5
Zfp236	1.25544	0.708814	0.957947	497
Zfp229	4.13737	3.49894	0.957947	345
Zfp120	11.8335	7.06461	5.94183	662.5
Zfp11	1.77316	1.46995	0.342623	340
Zfhx3	0	1.08938	0	1328
Zfa-ps	0.332194	0.708814	0	497
Zeb2	41.8931	22.9046	29.2447	504.5
Zbtb43	11.3264	15.7415	15.2436	430.5
Zbtb26	5.31371	2.50701	1.23065	425
Yod1	1.5143	2.12644	0	337
Ykt6	13.4716	10.3429	3.97891	347.5
Yipf6	19.7265	10.2745	15.4246	340.5
Xpnpep3	4.83484	3.59639	5.41234	506
Xkr4	2.10535	1.41763	0.615324	263.5
Xirp1	0	0.380568	0.615324	432.5
Xiap	10.6551	5.6777	5.40506	411.5
Xaf1	1.64268	1.08938	2.09731	686
Wtap	0.996582	0.380568	0.957947	425.5
Wdr73	1.5143	5.85617	1.30057	191
Wdr33	62.713	48.3447	36.8582	343
Wdfy3	4.41242	1.46995	2.74827	421.5
Wash	1.5143	0.708814	2.53122	337.5
Vti1a	17.8037	11.6447	14.2969	257.5
Vps37a	3.0286	1.46995	0.957947	422
Vps26a	20.4611	15.4008	13.7858	260.5
Vav3	0.923247	2.17876	1.84597	329
Vamp3	4.61414	3.49894	2.09731	337.5
Uvssa	3.15698	1.7982	1.57327	349
Uty	25.454	11.8419	18.5423	337
Usp48	5.39071	3.64871	3.83179	340
Unc80	7.38388	3.97696	8.15332	349
Unc13a	3.88871	1.46995	1.57327	423.5
Unc119b	0.332194	0	1.57327	339
Ubxn8	3.92989	2.10922	3.12974	417
Ubr2	3.48917	2.7378	2.53122	344
Ube2r2	5.39071	2.23109	4.44711	337.5

Ube2q2	6.18558	3.52684	5.26541	339.5
Ube2h	8.17368	9.09939	4.35583	346
Txnl4a	61.0885	43.6909	39.6711	337.5
Tulp4	140.145	88.22	208.202	341
Tubgcp4	2.56593	2.17876	1.30057	338.5
Tube1	2.82479	0	1.7204	349
Ttc19	0.923247	2.55933	3.81771	430
Ttbk1	0.664388	0	1.57327	253.5
Tsr2	20.2387	17.7794	16.5542	341.5
Tspyl5	0	0.380568	0.615324	336.5
Tspan7	76.8578	64.2788	78.7865	331.5
Tspan2	9.12791	8.64932	2.40564	354.5
Tspan15	0	0	0.615324	507
Trmt2b	1.77316	0	1.57327	416
Trio	0.332194	0	0.685246	333
Trim44	39.2531	22.0738	33.0774	421
Trim30a	0	0.708814	0.615324	260.5
Traf6	0.664388	0.380568	0.685246	430.5
Tppp	8.34495	12.2558	12.6285	424.5
Tpmt	6.07381	3.59639	2.4613	338.5
Tpd52	3.00664	1.72865	4.01321	335.5
Tom1l2	17.9023	15.6599	19.3904	434
Tollip	20.577	10.7322	10.5216	414.5
Tmod3	26.4136	16.4706	17.5004	331
Tmf1	2.95527	1.72865	1.64319	664.5
Tmem87b	5.58308	6.45983	6.38337	331.5
Tmem71	0.591053	0.708814	0	338.5
Tmem263	1.7512	0.761137	0.957947	423
Tmem164	3.87851	1.46995	1.57327	347.5
Tmem151b	4.63011	2.02899	2.25852	416.5
Tmem151a	2.34225	0	1.30057	267.5
Tlk2	0.332194	2.17876	0.342623	502
Tia1	25.8026	17.2753	27.2065	509.5
Tgoln1	47.0314	41.2857	50.1546	346
Tfap2b	5.05852	2.17876	1.57327	342.5
Tfam	8.66883	5.95312	3.81043	510
Tef	2.51088	6.41442	9.78224	269
Tdrp	1.05163	0.708814	0	339.5
Tctn1	0	1.41763	0.342623	265.5
Tcf7l2	33.201	10.9358	9.56995	270
Tbl2	0.996582	2.08131	0.615324	418.5
Tbkbp1	3.74803	2.81803	2.33572	431.5
Szrd1	14.2238	6.12225	8.61053	418.5
Sytl5	4.59795	3.54407	5.06243	419.5
Sytl4	0	1.08938	0.615324	505
Syt7	18.2394	7.71621	14.215	339.5
Syt6	2.30707	2.17876	0.685246	339.5

Syne3	0.923247	0.708814	0	332.5
Sugt1	60.1904	42.1153	45.3223	345
Stxbp4	5.24006	1.08938	3.00358	344.5
Stk24	0.664388	0.708814	0	265
Stk16	2.08339	1.08938	0.957947	342.5
Ssfa2	2.95527	0.380568	1.37049	272
Srr	44.167	29.062	23.3137	332
Srgap2	17.8084	12.5115	9.65913	337
Spon1	10.6903	7.30909	3.97891	342
Spag9	116.629	75.7723	102.601	501.5
Snx30	0.996582	0	0.615324	346.5
Sntg1	0.591053	0	0.615324	336.5
Snhg11	59.6379	49.5254	58.1647	774
Snap91	7.64274	3.21582	5.97201	188.5
Sms	2.08339	4.61623	2.25852	342
Smim13	1.7512	3.54407	0.957947	499
Smg5	4.16408	3.73925	3.52289	260.5
Smg1	2.10535	2.12644	1.64319	334
Smad5	12.8437	6.89625	0.685246	340
Slx1b	0	1.08938	2.59721	333.5
Slc7a1	6.01492	0	1.82461	497
Slc6a6	0.591053	1.46995	1.30057	429
Slc5a7	0.591053	0.380568	0.615324	438.5
Slc39a14	2.36421	1.41763	1.57327	190
Slc36a1	1.16015	0.708814	0.615324	413
Slc35d1	8.54728	2.34001	4.27728	515.5
Slc35a5	5.0167	8.53928	5.78141	487
Slc30a9	0.591053	0.708814	0	337.5
Slc30a10	0.923247	0.708814	0.342623	499.5
Slc25a37	2.10535	2.12644	1.91589	343.5
Slc25a36	1.5143	2.83525	0.866667	258
Slc25a10	0	0	1.23065	346.5
Slc22a23	0	0	0.615324	345
Slc16a1	2.39637	1.08938	3.90899	347.5
Slc14a1	3.74803	1.41763	2.80392	413
Shc4	0.591053	0	0.615324	407
Sh3glb1	6.03524	3.64871	5.65526	495.5
Sgms2	0	0	0.957947	328
Sgcb	3.79941	3.31356	1.79032	429
Sfxn2	1.5143	1.4004	1.57327	329.5
Sfmbt2	2.69641	3.11837	0.957947	336
Sfmbt1	0.591053	0.761137	2.53122	427.5
Sertad2	2.17869	0	1.30057	503.5
Senp8	1.18211	2.12644	1.23065	341.5
Sema3e	1.26596	0.708814	1.91589	339
Sema3d	3.03972	2.7378	4.03457	420
Sec63	3.19216	1.46995	0.957947	504

Sec14l1	9.01814	6.84976	9.66399	430.5
Sdad1	4.61414	2.89002	4.16033	338.5
Scn9a	3.0286	2.02899	1.23065	416
Scn2b	59.4223	74.8425	93.6466	502
Scn1a	10.4888	11.4383	15.459	344
Scin	0.332194	0.708814	0	514
Scfd2	0.923247	1.7982	0.342623	259.5
Sbf2	1.18211	0.761137	0.615324	346.5
Sash1	1.5143	2.88758	2.33572	1057.5
Runx1	0	0.380568	0.615324	595
Rprd1b	2.17869	0.708814	0.615324	334
Rnpc3	6.56915	5.06634	5.48226	344.5
Rnf38	2.10535	1.08938	0.957947	258
Ric3	11.4434	6.20113	12.4048	507.5
Rhbdd2	0.827951	3.72203	2.40564	336
Rgp1	1.84649	1.7982	1.57327	406.5
Rfxank	2.36421	2.10922	3.44079	340.5
Reps2	1.58764	1.7982	1.91589	506
Reep1	11.9596	9.78008	3.28392	344.5
Rdh13	1.18211	1.08938	1.64319	337.5
Rbfox2	78.546	55.8001	45.1684	360
Rbbp6	13.2529	10.6745	10.921	337
Rapgef4	16.023	14.5468	23.6392	348
Rapgef3	0.591053	0	0.615324	417
Rap2b	1.64268	0.708814	0.342623	441
Ralgapa2	1.5143	1.08938	1.30057	425
Ralgapa1	0	0	0.615324	349.5
Rag1	3.61755	0.380568	1.23065	505
Rad54l2	0.591053	1.46995	1.23065	431
Rad1	0.923247	1.85052	2.4613	256
Rabep1	2.67445	1.41763	0	337.5
Rab9b	7.12418	8.08422	11.0202	350.5
Rab6a	2.10535	2.17876	1.37049	262.5
Rab2b	15.5298	14.0516	10.3053	266
Rab26	1.25544	0.380568	1.30057	178
Rab11fip2	16.6924	11.1812	13.0832	421
R3hcc1l	4.51884	4.92725	4.55861	341.5
Ptrhd1	5.41716	7.8898	6.11417	424.5
Ptpra	1.25544	0.380568	0.957947	343
Pter	0.591053	0.761137	1.91589	424
Ptchd2	0.332194	0.380568	0.615324	272
Ptchd1	0	2.17876	1.84597	427.5
Prtg	0	0.708814	0	582
Prrt2	35.1437	23.6938	27.6531	345
Prrg1	4.8751	0.380568	2.16724	422
Pros1	0.591053	2.10922	1.77293	417.5
Prokr2	4.7798	3.85509	0	349.5

Prkg2	0	0.380568	0.615324	264
Prkci	3.07997	0.708814	5.98419	334.5
Prex1	0.332194	0	1.4477	268
Prdm2	5.08793	2.17876	3.90171	511.5
Prdm15	2.43755	1.41763	3.74051	341.5
Pptc7	0.332194	0.708814	2.01955	520
Ppp2r5e	0.664388	0.708814	0.342623	334.5
Ppp2r2c	9.51935	5.24481	4.89339	337.5
Ppp2r1b	2.73908	1.14171	0.342623	407
Ppp1r3a	0.827951	0.708814	0	350
Ppp1r16b	0.332194	1.01984	2.25852	341
Ppm1h	1.05163	0	0.615324	412.5
Ppm1e	5.13396	2.17876	2.25852	426
Ppil3	7.55321	5.01402	6.49586	517
Ppih	4.60814	2.02899	1.94962	340
Polr3h	4.72843	1.46995	2.38825	420
Pnma2	10.648	7.34006	3.65378	420
Pmpca	21.3174	16.8477	11.7492	355.5
Plekhm3	4.42294	2.88758	6.31463	414
Plekhm1	6.43845	1.08938	2.25852	415.5
Plce1	0.591053	2.12644	1.84597	337.5
Plagl2	0.332194	0	0.615324	336
Pkn2	0.591053	0.708814	0.615324	350.5
Pitpnm3	0	0	0.615324	348.5
Pin1	21.8649	14.9535	17.0652	431
Phtf1os	1.77316	0.708814	0.615324	423
Phldb2	3.65274	0	0.957947	333
Phf8	0.332194	0.761137	0.957947	505
Phf21a	15.9335	4.30521	17.7603	338
Phc1	0	0.708814	0.957947	340
Pex13	4.30865	3.14628	4.54957	345
Pdxk	5.03321	4.58528	6.64604	509
Pde6a	0	0	0.615324	604.5
Pde4dip	40.1331	28.2912	19.8259	260
Pcyox1	27.697	17.3273	27.3257	438
Pclo	3.23032	1.46995	4.35583	499.5
Pcbp2	0	0.761137	1.02787	414
Pate2	0	0.708814	0	340.5
Parvb	2.5214	1.7982	0.615324	412.5
Pard3	1.18211	0	1.02787	269
Pappa2	0.923247	1.46995	0	346.5
Papolg	38.2876	19.3838	18.854	425
Pak2	0.591053	2.88758	0.957947	334.5
Pafah2	1.16015	1.01984	0.957947	338
Orc3	11.7798	7.40635	9.77326	435
Olfr29-ps1	0	0.708814	0	414.5
Ocln	3.3608	1.41763	3.56637	348

Nxph1	6.90712	4.9617	3.02097	332.5
Nutf2	29.3267	19.4349	23.021	342
Nudt4	5.72501	3.4573	1.91589	416
Nudt13	6.14409	7.48737	6.80438	257
Nptx1	1.5143	1.52227	3.28638	506
Nol7	33.7645	25.2016	16.0639	341.5
Nol4l	1.7512	0.761137	0	343.5
Nmt2	20.6928	10.6569	10.6735	417
Nmnat2	44.9503	42.4657	36.2672	491.5
Nkap	4.6845	5.9364	3.09089	327.5
Nifk	21.6991	14.6886	12.3118	428
Nfix	1.58764	0.380568	1.57327	418.5
Nfic	2.43755	0.708814	1.91589	335
Nfatc2	0.591053	0.380568	0.615324	328
Neto2	5.78005	3.21582	1.23065	346.5
Nedd4	124.641	82.1603	58.4521	345
Ndst1	3.10194	1.41763	3.48916	490
Ncor1	9.06174	1.41763	9.50974	186.5
Ncam2	16.6936	8.31388	11.2663	421
Nav2	13.9172	3.21582	7.35151	257.5
N4bp1	0	0	0.615324	423.5
Myt1	15.0396	9.76358	12.9147	337
Myo9a	11.8089	2.99222	1.64319	411
Mtus2	1.25544	2.88758	3.39788	415
Mttp	17.4978	9.48428	16.5964	189.5
Mtif3	20.143	11.1349	13.5987	497
Mrpl35	27.9293	24.2891	20.5449	429.5
Mroh1	1.84649	0	1.82461	353
Mplkip	7.49817	2.50701	0.957947	263.5
Mob3b	2.82479	2.88758	0.957947	424.5
Moap1	3.95185	2.50701	2.25852	407.5
Mmp24	1.38382	0.708814	0.957947	343.5
Mitf	2.10535	2.12644	1.30057	411.5
Mink1	3.65181	3.26815	4.53396	337
Mier1	26.3994	11.6141	17.6347	507
Mief1	10.1459	6.25977	5.75184	427.5
Mfap3l	14.1443	7.19278	15.8767	426
Mesdc1	0.591053	0	0.957947	497
Med22	3.0286	1.08938	3.29367	586.5
Med17	2.43755	1.41763	0.957947	356.5
Med12l	3.74803	1.52227	0.957947	431.5
Mdga1	0.664388	0	0.615324	332.5
Mcc	0.591053	0	0.615324	512.5
Mcat	0.591053	0	0.615324	501.5
Mbtd1	0.827951	1.32017	0	337
Mbnl2	90.2924	112.458	109.391	499
Marcks	47.8113	19.4742	7.95593	335

March6	1.77316	2.60827	0.615324	336
Mapkbp1	9.24727	3.83787	3.76186	262.5
Mapk9	8.0432	5.37045	3.12518	412
Mapk1ip1l	7.95664	5.60816	2.18859	419
Mapk1	10.4426	12.3229	6.45315	424.5
Mapk10	44.4468	27.644	46.4199	338
Map4k2	5.39281	1.08938	3.02097	350.5
Map3k7	1.419	0	2.53122	254
Maml2	3.10194	3.21582	2.25852	336
Malt1	0	0.708814	0	339.5
Lztfl1	12.475	9.03992	3.97891	264
Lynx1	37.7722	41.4556	43.4281	349
Luzp2	40.9103	34.8206	34.5281	426
Luzp1	14.0239	12.4495	15.2858	343.5
Luc7l	22.3005	12.8801	12.1191	425.5
Luc7l3	50.5324	30.8474	41.463	342.5
Lrrc61	1.5143	2.50701	1.23065	426
Lrp4	0.923247	0	1.57327	348.5
Lppr4	5.56922	4.20775	2.53122	332.5
Lpgat1	1.25544	0.708814	0	342.5
Lnpep	14.7129	12.9022	12.4691	423
Lins	4.53146	2.50701	2.16724	352.5
Lin54	0.591053	1.41763	1.30057	343
Leprot	5.51807	4.26008	4.22753	183
Lemd3	0.332194	0	1.4477	416.5
Lancl1	7.98755	3.1986	6.05411	329.5
Lamp2	16.9368	10.5793	12.2092	351
L2hgdh	0	0	1.10507	340
Krr1	15.4239	13.8758	16.3637	335
Krit1	5.64794	4.07822	6.23743	500.5
Krba1	6.54993	2.17876	3.14654	505.5
Kpna3	15.7608	13.8242	5.12842	411.5
Klhl42	1.5143	0.708814	0.342623	421
Klhdc10	10.76	4.06866	5.85918	343.5
Klf10	11.9034	10.246	9.82479	257.5
Kirrel3	17.1973	7.90643	7.95256	345
Kif5a	51.4011	41.3319	79.6581	177
Kif2a	15.8234	7.07811	7.73691	344
Kdm5c	8.95136	4.97957	3.46781	495.5
Kcnv1	2.56593	2.50701	2.53122	423.5
Kcnn3	1.5143	0.708814	2.82547	332.5
Kcnma1	9.19222	3.37279	5.56512	355
Kcnh1	0	0.708814	2.53122	261.5
Kcnc4	1.84649	0.708814	0.615324	323
Kcnc2	13.9164	6.89323	15.1854	495.5
Kcnb2	12.7031	7.5038	8.16061	334
Kbtbd8	1.84649	0.708814	0	335.5

Kbtbd11	5.59453	3.59639	3.83179	289
Jup	0.591053	0.380568	0.615324	259.5
Jrk	0.591053	1.01984	0.957947	505
Josd1	0	0	0.957947	339.5
Jade2	0.591053	0	3.48916	417.5
Itga8	0.591053	0.708814	1.30057	347
Iqsec3	0.591053	0.708814	0	345.5
Inpp4a	10.6755	6.15572	13.556	261.5
Impad1	4.5429	0	0.615324	417
Ikzf5	15.6124	10.1904	11.2873	742.5
Ikbkg	7.40217	5.40084	7.60554	412
Ikbkap	10.6333	7.82463	4.84558	267.5
Ifitm10	0.591053	2.40956	6.51429	251
Ick	5.98387	2.50701	3.99185	178.5
Htr1a	10.4865	4.96889	9.78641	421
Hspa14	38.2413	27.5511	27.8754	431
Hsd17b7	10.9306	7.51829	4.76719	431
Hmgcll1	3.0286	2.50701	1.84597	424.5
Hist2h2be	6.5213	1.08938	2.80757	251
Hist1h2be	11.934	7.07091	5.60784	428
Hhat	0.591053	0	0.615324	724.5
Heyl	0.923247	1.08938	0.342623	352.5
Hcn1	0	0.380568	0.957947	590.5
Haus2	10.5013	6.88035	7.93441	431
Gtpbp10	2.01006	4.15543	1.57327	348
Gtf3c4	0.591053	0	1.48199	426.5
Gtf3c2	2.93331	1.41763	1.91589	179
Grin2b	68.3694	37.4646	39.6996	257.5
Grem2	1.419	0.708814	0.615324	336.5
Gpr61	8.48476	12.7428	8.32056	336.5
Gpr158	8.38046	8.13239	7.72397	333.5
Gpr116	5.39071	3.21582	2.53122	491.5
Gpd1l	3.61965	7.5038	4.52432	333.5
Gosr1	10.1007	6.63755	9.79059	360
Gopc	6.3894	0.708814	3.28638	344
Golph3l	11.3093	4.25288	6.35348	343.5
Gnb4	6.3813	3.21582	0.685246	490
Gnaq	6.14105	1.7982	5.60784	430.5
Gmps	59.4907	47.4194	55.6483	413.5
Gm6194	1.84649	1.41763	1.23065	350
Gm2a	49.3614	30.3658	21.1447	501
Gm20604	7.20677	2.88758	7.28279	342
Gm20125	0.332194	0.708814	0	266
Gm19757	1.58764	0	1.64319	341.5
Gm15941	1.18211	1.41763	0	337.5
Gm10778	2.80283	2.79012	3.48916	425
Gm10408	0.332194	0	0.957947	744

Gm10390	0	0.708814	0	338.5
Gltscr1l	0.332194	0	0.685246	334
Glce	17.5923	13.0941	11.3965	342
Gfpt1	32.2866	16.298	21.4605	496.5
Gfod1	0.591053	0.708814	1.30057	437
Gemin5	1.97487	2.88758	1.57327	337.5
Gcnt2	13.3401	9.40326	8.8243	337
Gbp9	0.923247	1.08938	4.42456	342.5
Gbp7	1.97487	0.708814	0.615324	339
Galnt6	1.25544	0	1.73333	351
Galnt4	1.5143	0.708814	0.615324	774
Galnt16	5.62971	1.41763	2.33572	429
Gabrg2	78.5848	65.0395	57.0601	419
Gabra1	13.5097	7.07091	8.42483	405.5
Frmpd4	13.6371	8.16029	12.997	335
Frmd7	3.15698	0	1.23065	344
Foxp2	17.6826	4.28798	7.75827	423.5
Fndc3b	1.25544	0.708814	1.57327	422.5
Fmod	3.82137	2.83525	2.01955	340.5
Fhl1	6.90501	4.88096	4.03457	183
Fgd6	1.5143	2.50701	0.957947	418
Fgd5	3.74803	1.7982	2.4613	260.5
Fech	7.73086	4.89934	5.06608	523
Fdx1	0	0	0.615324	423
Fbxw8	0.591053	0	1.30057	418.5
Fbxo8	3.39388	3.97696	1.57327	339
Fbxo45	1.25544	3.72973	0.615324	420.5
Fbxo28	0	0	0.957947	350.5
Fam73a	6.50968	4.35753	2.16724	370
Fam60a	2.43755	0.708814	0	266.5
Fam49a	72.2646	46.8882	30.4324	342
Fam227a	3.0286	1.46995	0.957947	429
Fam178a	20.0883	15.5065	16.7801	419.5
Faim2	6.87584	9.96961	12.4295	330
Exog	7.3253	4.61867	2.90757	335
Etv6	1.58764	2.81803	3.02097	257.5
Etv3	1.87958	2.23109	4.50296	345
Erlec1	8.77795	11.9291	9.75261	418.5
Eri1	1.77316	0.708814	0.615324	495.5
Epas1	4.2726	2.10922	2.67834	510
Eny2	16.6724	12.5245	14.2385	409
Enpp4	0.996582	3.54407	2.16724	577
Emx2os	1.5143	0.708814	0.685246	527.5
Elovl5	13.6783	7.48923	8.86334	254.5
Elavl3	1.18211	0.708814	0.615324	409.5
Eif5b	40.8181	27.1785	38.7623	514.5
Eif5	1.58764	0.380568	0.957947	504

Eif1ad	14.5076	7.52518	7.31702	334.5
Egr3	2.43755	0.380568	2.32844	259
Efr3b	2.01006	1.14171	4.36038	494
Efr3a	1.5143	2.55933	5.70286	582
Efna5	2.01006	0.380568	0.957947	340
Edem3	1.5143	0.761137	0	347
Ebf1	13.1537	7.4687	5.20956	499.5
E2f3	0.923247	0.708814	2.25852	262.5
Dzip1l	1.18211	0.708814	0.615324	349.5
Dzank1	31.2073	27.5227	38.3356	258.5
Dusp22	2.51088	0.380568	2.53122	343.5
Dtna	68.3619	45.9102	57.4652	414
Dpysl5	12.251	6.26465	0.685246	343
Dnmt3a	4.68957	3.97696	0.957947	344
Dnajc5	15.1692	9.72032	8.01823	416.5
Dnajc18	36.6237	29.0549	26.2294	652.5
Dnaja2	26.2971	17.0258	21.7411	341
Dmxl1	0.591053	0.708814	2.25852	343
Dlx6os1	25.8338	15.7253	28.865	335
Dip2b	3.95185	4.86424	4.99251	340.5
Dhx33	1.5143	0.708814	0.957947	338
Dgkb	46.318	35.3689	72.7496	425.5
Dgcr2	2.51088	1.72865	3.05526	344.5
Dennd6b	1.77316	0	1.7204	346
Dennd4c	4.28194	0.761137	0.615324	426.5
Dennd2d	0	0	0.615324	331
Ddx6	33.4148	22.2975	21.8408	265
Ddx51	2.10535	1.46995	0.957947	417.5
Ddit4l	0.332194	1.46995	3.90899	421
Ddah1	1.77316	3.59639	0.957947	429
Dcp1a	10.9553	2.73292	5.33878	340
Dck	1.5143	1.7982	0.685246	414.5
Dcdc2a	0.664388	1.08938	0.957947	342.5
D16Ertd472e	0.332194	0.708814	3.14654	333.5
Cyth4	6.99734	6.63383	3.00358	333.5
Cycs	72.2381	76.9072	55.0193	345
Cyb5b	15.9251	11.9996	10.959	335.5
Cyb561d1	0.591053	0.380568	2.09731	428.5
Ctif	0	0.708814	0	341.5
Csnk1g1	9.38552	6.64521	6.57759	502
Csmd1	6.07107	7.14765	3.70621	341
Crybg3	7.77322	2.50701	4.03457	256.5
Crnkl1	9.55804	8.48679	8.71625	337
Crkl	0.332194	2.17876	1.30057	335
Creg2	0	0.708814	0.957947	429
Cpsf2	8.1309	2.48979	2.23716	343
Cpox	0.827951	0	1.30057	601.5

Cpne4	2.43755	0.708814	3.05526	509.5
Cplx2	235.641	179.418	197.896	1114
Cpeb4	8.38887	4.68577	9.82494	423
Cpd	1.25544	0.380568	0.615324	506
Col8a1	2.93331	1.70074	1.94962	430.5
Cobl	1.5143	0.708814	2.4613	334.5
Cntnap2	49.6758	31.3263	30.655	333.5
Cnksr2	9.78531	5.06634	7.28164	268
Cln8	0.923247	0.380568	0.957947	427
Clec5a	0.332194	0	1.23065	502.5
Cisd2	9.34056	9.71428	8.06051	586.5
Chtf8	3.11245	2.28341	3.50154	414.5
Chrna4	2.36421	1.46995	2.60114	333.5
Chrm1	4.88561	2.83525	2.80392	340
Chm	27.7324	19.6816	19.6243	264
Chl1	104.128	40.9464	37.0309	343
Chdh	0	0	1.30057	336.5
Chd9	4.35231	5.52023	3.48916	343
Chd6	73.6653	46.6095	52.8	352.5
Chd2	45.2888	30.4413	37.5676	440.5
Champ1	1.58764	2.64035	0.685246	262
Cgn	2.10535	1.08938	0	415.5
Cflar	7.25043	3.59639	5.04108	335
Cers1	0.332194	0.708814	0	411
Cerk	1.84649	0.708814	0.685246	424
Cenpo	2.89812	1.7982	2.18859	337
Cenpf	17.2082	1.08938	0.615324	343.5
Cdip1	18.2248	17.5134	21.9618	263
Cdc42se2	55.4673	41.9675	33.5842	429.5
Cd2bp2	12.3195	5.22759	9.70845	344.5
Ccnj	0.591053	0	0.615324	413
Ccdc93	9.28245	1.41763	5.73361	491.5
Ccdc85a	8.54396	3.97696	2.4613	349.5
Ccdc6	0.591053	1.85052	1.30057	276
Ccdc167	9.24067	5.77896	7.49844	346
Cbx4	1.58764	0.380568	2.25852	269
Casp9	0.591053	1.85052	1.30057	424.5
Casp2	1.77316	2.17876	0.615324	497.5
Car5b	0	0.708814	2.33572	422.5
Capza1	9.95497	5.7584	9.43016	345.5
Cacfd1	0.923247	2.17876	0.615324	346.5
C330018D20Rik	2.17869	0.708814	0.685246	328.5
Btf3l4	38.5538	35.6499	29.2165	347
Brip1	1.5143	2.17876	0	349.5
Bmpr1b	17.2922	10.7407	16.5464	424
Bmi1	5.42497	2.08131	4.17806	342.5
Bcl2l11	2.93331	1.72865	1.30057	347

BC031361	0	0.708814	0	350.5
BC030336	2.60111	3.21582	1.23065	346.5
Bahcc1	1.7512	0	1.23065	338.5
B3glct	16.0274	3.64871	4.90267	256
B3galt1	1.5143	0.708814	0.615324	358.5
Atxn7l3	0	0.708814	0.957947	347
Atxn7	0	0.708814	0.342623	425
Atp6v1a	30.2011	21.7246	17.6705	341
Atp11c	6.54238	3.4573	3.81498	343
Atmin	0.591053	0.380568	0.957947	181.5
Atl2	29.5986	23.1677	24.4393	345
Atg2b	1.5143	1.08938	0.342623	336
Atg16l1	3.74803	4.30521	4.10449	260
Ate1	55.201	37.2194	56.125	335
Astn1	39.8986	23.7493	25.7229	420
Asb15	0.591053	0.380568	0.615324	417
Arpp21	233.657	181.704	145.517	411.5
Arpp19	30.8018	16.6489	18.998	501.5
Arntl2	0.591053	0.708814	0.615324	361.5
Arl14ep	14.2949	7.07811	14.2758	435
Arid3b	0.591053	0.380568	0.615324	419.5
Arid2	7.69988	4.25288	4.03457	590
Arhgef7	5.39281	9.27106	3.92193	428
Arhgap31	4.41242	4.20775	0.685246	337
Arfip2	4.7777	2.84245	2.95104	266.5
Arf4	32.6875	23.9898	19.0201	332
Arf3	7.99229	10.5342	3.93544	333
Appl1	33.3177	14.0048	19.9841	423
Apc	8.88199	4.92725	0.685246	431
Apc2	23.7873	8.89597	4.01321	423.5
Apba1	3.0286	0.761137	0.342623	262
Ap5m1	8.82274	2.50701	3.46781	260
Ap4e1	11.5654	4.91657	8.02193	264
Ap2b1	22.6751	13.5132	14.0901	339
Ano6	35.4427	15.6717	23.7799	339
Ankrd29	2.56593	3.82718	1.82461	421
Ankrd10	0.996582	0	0.615324	345.5
Ankle2	1.5143	0	1.30057	272.5
Alx4	3.54632	1.08938	1.30057	414.5
Alkbh5	0	1.41763	0.685246	345
Alg11	22.0163	17.201	21.7004	427.5
Aldh1l2	0.591053	0	1.23065	345.5
Akap13	0	0	1.30057	413
Ak4	1.25544	0.708814	1.64319	411
Ahcyl2	5.85339	1.08938	4.10449	427
Ago3	4.98151	2.81803	4.99251	343.5
Ago1	0.332194	0.708814	0	413

Agl	8.31065	2.98884	6.11545	509
Aff3	0.332194	0.761137	1.30057	335.5
Aff1	1.5143	0.380568	1.91589	498.5
Afap1	8.13849	4.94447	0.342623	582
Adora1	1.25544	2.17876	2.25852	429.5
Adcy9	0	0	0.957947	419
Adam19	9.75922	10.1323	2.71264	345
Acvr2a	2.69641	1.46995	1.57327	429
Abt1	8.43803	3.17069	1.91589	347
Abcc9	0.923247	1.7982	0.957947	414.5
A730017L22Rik	13.0067	3.21582	6.47394	256
A530054K11Rik	8.32067	7.74909	8.01713	339.5
A330050F15Rik	0.923247	0	0.615324	260
A330049N07Rik	0	0.708814	5.35224	506.5
6330403A02Rik	0.591053	0.380568	0.615324	522
5530601H04Rik	6.47962	6.46336	6.1507	345.5
4930525G20Rik	0	0	0.615324	341
3110052M02Rik	4.80176	2.88758	5.4609	504
2700054A10Rik	1.25544	1.41763	0.615324	342
2610005L07Rik	38.4651	32.1171	31.9493	336.5
1810041L15Rik	4.20861	2.17876	1.57327	587
1810026B05Rik	0.591053	1.7982	2.53122	420
1810013L24Rik	7.8084	2.88758	4.17441	520
1110002L01Rik	3.3608	4.25288	4.30727	332.5
Zswim4	0.332194	1.08938	0.615324	346
Zscan22	1.5143	2.37395	2.40564	330
Zscan12	2.69641	0	0.615324	499
Zranb2	216.94	160.142	179.588	510
Znrf1	5.18533	9.43534	5.878	334
Zmynd8	17.4691	14.8946	17.8787	347
Zmpste24	24.5045	18.766	18.0727	335
Zkscan1	26.206	16.5438	13.4086	500
Zik1	6.78715	3.90741	3.47236	335
Zic4	11.0144	3.21582	0.342623	174
Zgrf1	2.10535	0.708814	0.615324	341
Zfyve27	1.5143	0	1.23065	341
Zfpm2	3.61965	1.46995	1.57327	358
Zfp961	4.7798	3.80996	3.29367	343
Zfp938	19.3916	13.3379	14.2751	179
Zfp91Cntf	0	0.708814	0.342623	331
Zfp91	0	0.708814	0.342623	340
Zfp874b	6.09029	4.22447	5.99902	335
Zfp874a	4.05827	5.83895	5.33513	359
Zfp870	7.30844	1.41763	5.20956	368
Zfp830	10.6788	10.6197	3.76186	337
Zfp827	11.1256	9.60183	5.89481	348
Zfp800	1.7512	0.380568	2.53122	1028

Zfp784	2.69641	0.708814	2.18859	342
Zfp763	6.73546	1.85052	3.76186	337
Zfp747	0	0.761137	0.615324	343
Zfp746	0.591053	0	0.615324	492
Zfp709	5.03866	4.63345	6.32681	178
Zfp703	1.71601	1.01984	1.10507	336
Zfp697	4.28404	3.97696	2.18859	497
Zfp654	17.497	6.71476	17.2349	508
Zfp652	30.1032	23.5997	28.4715	347
Zfp644	3.98401	1.7982	4.59424	170
Zfp619	3.88964	1.4004	1.91589	330
Zfp612	5.41952	4.91657	6.39357	340
Zfp609	0.923247	0.761137	0.342623	341
Zfp599	1.18211	1.08938	0.342623	345
Zfp595	2.23373	1.70074	1.10507	341
Zfp593	0.332194	0	0.615324	179
Zfp58	9.39797	6.56499	3.50154	348
Zfp575	0.332194	0	0.957947	527
Zfp54	2.23373	1.01984	1.84597	194
Zfp532	0	1.46995	0.615324	345
Zfp516	0.664388	1.41763	0.615324	348
Zfp512	38.971	24.1436	23.7585	340
Zfp467	1.91983	2.88758	1.55647	338
Zfp458	3.71759	2.9399	5.71149	329
Zfp445	12.3935	8.50906	12.3909	337
Zfp444	3.31962	0.708814	0.615324	498
Zfp397	9.29719	2.17876	3.43351	497
Zfp386	21.9517	17.5755	10.1995	352
Zfp384	4.80176	1.7982	2.87384	351
Zfp36l1	12.1195	6.88339	1.91589	349
Zfp367	0	0.708814	0.342623	345
Zfp366	1.25544	3.34916	1.48199	338
Zfp365	3.54632	2.50701	11.5976	498
Zfp334	0.591053	1.4004	1.48199	345
Zfp324	0.591053	1.41763	0	349
Zfp322a	23.3962	20.0006	18.0094	348
Zfp282	2.10535	1.7982	2.4613	177
Zfp273	4.94885	4.51877	2.16724	176
Zfp260	24.8974	10.2964	13.4137	507
Zfp235	2.95527	1.72865	3.32796	185
Zfp191	8.74006	4.20775	4.97115	326
Zfp169	2.10535	1.78097	2.95104	508
Zfp157	3.9404	3.49894	2.78256	334
Zfp142	4.8751	0.761137	1.30057	338
Zfp106	10.7133	11.6837	12.5333	329
Zfhx4	12.9244	7.63714	10.2538	343
Zfc3h1	2.51088	0.380568	3.07662	177

Zer1	0.664388	1.41763	2.18859	351
Zeb1	28.9544	17.9122	21.6723	324
Zdhhc8	0.923247	0	2.78256	348
Zdhhc20	18.8984	11.0172	18.8792	350
Zbtb7c	1.18211	0.761137	0.685246	335
Zbtb6	5.64957	1.85052	3.12518	510
Zbtb40	0.591053	1.08938	1.57327	340
Zbtb39	0.591053	0	1.64319	500
Zbtb37	1.18211	1.41763	0	347
Zbtb33	1.97487	2.12644	0	180
Zbtb18	82.6921	48.5242	29.6983	341
Zbtb16	0.591053	0.708814	1.67692	337
Ywhaz	85.4394	54.5473	56.7021	179
Ywhaq	31.1419	23.7341	22.4372	499
Ythdf2	7.52487	1.7982	3.74051	180
Ythdc2	0	0.708814	3.29367	337
Ypel1	0.664388	0.380568	0.615324	337
Yeats4	7.58052	3.11837	3.05526	179
Ybey	4.71364	1.08938	3.81771	493
Yae1d1	20.4311	6.84731	11.6966	353
Xylt2	1.77316	0.708814	1.82461	183
Xrcc6bp1	0	0	0.685246	189
Xrcc2	4.37125	2.79012	5.57657	489
Xndc1	22.2863	10.4724	13.7726	337
Wscd2	0.332194	0	0.615324	338
Wnk1	3.448	3.1986	2.09731	329
Wipf1	4.5429	1.41763	3.76186	357
Whsc1l1	15.0209	9.31922	9.79651	340
Wfs1	2.08339	0	2.09731	356
Wfikkn2	0	0.708814	0	341
Wdr82	4.59795	4.86424	2.18859	333
Wdr81	0.332194	0	0.615324	342
Wdr76	10.1272	5.58977	11.2951	336
Wdr4	0.923247	2.02899	3.07662	334
Wdr18	8.7437	6.29097	3.41062	337
Wdr11	5.78005	2.9399	4.18169	329
Wdfy2	0.332194	0	0.615324	178
Wbp4	25.02	18.0336	18.2206	180
Wasl	5.46615	3.82718	6.47906	348
Wapal	4.05827	1.08938	0.957947	337
Wac	8.12916	5.96343	8.18056	356
Vstm2a	17.8291	14.239	14.2166	195
Vopp1	0.332194	1.08938	1.30057	339
Vkorc1l1	0.664388	0	0.957947	500
Vipr2	2.10535	0	2.53122	344
Vipr1	0	0.380568	1.23065	348
Vhl	2.08339	0.761137	0.615324	511

Vezt	7.29062	5.13013	1.57327	337
Vcl	3.48917	1.08938	3.56637	342
Vat1l	46.0665	26.0994	20.0597	329
Vamp4	16.5679	7.24403	6.12876	187
Vamp2	4.8997	3.8223	4.45076	183
Utrn	0.923247	0.761137	1.23065	351
Utp23	4.03905	2.40956	2.18859	330
Usp8	136.946	106.388	103.915	182
Usp7	2.51088	0	1.23065	694
Usp49	16.9252	16.6549	18.1599	341
Usp46	18.4353	10.8462	10.1398	344
Usp43	0.332194	0	0.615324	353
Usp36	1.18211	0.761137	1.64319	334
Usp22	4.76059	1.7982	0.957947	346
Usp14	21.0242	10.6955	19.1121	510
Usp12	4.20861	2.12644	1.48199	344
Upk1b	0	0.380568	0.615324	190
Umodl1	0	0.708814	0	337
Ulk2	0.591053	0	1.30057	344
Uimc1	5.03563	4.80189	5.73361	175
Uhrf1bp1l	0.923247	5.12243	1.82461	509
Ugcg	1.32878	2.7378	0.615324	185
Uevld	2.67445	1.85052	0.342623	338
Ubxn2a	5.07723	2.32163	4.75354	180
Ubxn10	0.923247	0.708814	0.615324	339
Ubtf	8.76203	3.49894	3.14654	505
Ubtd2	0	0.761137	0.957947	351
Ublcp1	5.64266	2.10922	6.92455	333
Ubl4	9.56738	8.49052	5.35669	336
Ube3b	1.84649	2.02899	0.342623	185
Ube3a	5.79328	4.30521	2.95104	342
Ube2z	2.43755	1.41763	1.23065	494
Ube2n	75.416	57.4878	45.945	506
Ube2k	116.021	83.4344	72.0982	496
Ube2j2	7.69988	3.14628	2.18859	176
Ube2j1	18.2751	15.3923	20.481	358
Ube2e2	6.68134	3.94251	2.85248	335
Ubash3b	0.923247	1.85052	0.615324	343
Ubap2l	29.5305	27.6464	18.4435	355
Ubac2	1.25544	0.761137	1.57327	330
Ubac1	0.332194	2.50701	0.615324	338
Uba5	3.78322	0.761137	3.05526	172
Tyw5	15.8735	10.9362	9.72565	333
Tyw1	2.10535	1.7982	0.957947	347
Txlng	13.6341	8.69558	9.29269	343
Txlna	7.21432	2.9399	7.38973	496
Twistnb	3.28746	1.08938	1.57327	345

Twf1	9.28659	7.61876	4.89339	349
Tufm	8.00801	2.40956	2.18859	547
Tubb5	115.648	57.7967	26.7869	321
Ttpa	5.00348	5.01402	0.957947	350
Ttll12	0.923247	1.41763	0.615324	495
Tti2	1.18211	1.08938	2.31436	328
Ttc9c	21.0505	13.9174	10.127	350
Ttc39a	0.591053	0.380568	0.615324	189
Ttc23	4.93014	2.7378	2.53122	339
Ttc17	7.8058	1.41763	4.6409	184
Tspyl1	15.0369	7.26298	15.1311	340
Tspan9	0.591053	2.10922	0.615324	335
Tspan12	4.16408	2.88758	2.74827	351
Tspan11	0	1.08938	1.57327	343
Tshz1	7.10883	5.36014	10.8741	353
Tsc1	1.58764	0.380568	2.53122	329
Trpm2	3.15698	0	1.84597	338
Trp53inp2	4.28404	1.90284	5.82488	345
Trp53cor1	0.332194	0	0.615324	513
Trnp1	0	0.761137	1.20929	334
Trmt6	19.7195	12.4244	17.4259	339
Trmt61b	12.1694	5.62538	6.202	184
Trmt12	2.67445	1.08938	1.57327	186
Trmt10b	9.48271	7.04716	9.50434	340
Trmt10a	21.5578	9.80688	17.1325	1239
Trip4	16.7145	6.74267	10.1467	331
Trip12	15.4989	6.97346	12.6976	334
Trim62	0.332194	0.380568	1.79032	324
Trim56	1.05163	0.761137	0.342623	720
Trim36	1.32878	1.7982	0.342623	353
Trim35	9.84881	5.47072	11.3374	344
Trim30e-ps1	1.18211	0.708814	0	508
Trim24	3.2655	1.7982	2.18859	340
Trim21	0.923247	0.708814	0.957947	332
Trim12c	2.43755	1.41763	1.57327	964
Trhr	6.31396	5.32504	3.48916	493
Trhde	4.46957	1.7982	5.67776	323
Trappc13	39.5699	39.0149	23.2155	350
Trappc10	3.0286	0	1.84597	171
Trak2	15.5068	11.8248	13.25	341
Traf3ip1	1.91983	2.55933	3.02097	330
Tra2b	32.4928	17.8109	23.5434	344
Tpm2	8.50791	1.08938	0.342623	676
Tpm1	55.4617	33.6879	38.5145	185
Tomm70a	4.48576	1.85052	2.53122	335
Tnrc6c	2.43755	0.708814	1.57327	336
Tnrc6a	18.5199	11.2214	15.0662	182

Tnfaip8l1	0	0.708814	0	495
Tnfaip8	5.40394	2.17876	1.23065	178
Tmtc3	4.74462	2.80081	2.25852	497
Tmem65	4.03905	1.46995	0.957947	180
Tmem55b	0	0.708814	0.342623	181
Tmem50b	9.50104	10.4107	9.0372	331
Tmem47	16.7061	18.0915	20.5535	489
Tmem44	0.591053	1.08938	0	339
Tmem43	13.6959	10.1415	11.7088	495
Tmem33	10.9386	10.5381	8.21991	522
Tmem229b	3.19216	1.08938	0.615324	178
Tmem19	2.93331	2.88758	4.16033	186
Tmem179	1.5143	0.380568	1.30057	357
Tmem177	1.25544	3.17069	1.7204	352
Tmem167b	6.4766	2.79012	5.84644	185
Tmem161a	0.923247	0	2.95104	181
Tmem150c	4.5429	2.83525	1.10507	671
Tmem135	6.3823	4.20775	4.29745	332
Tmem129	3.0286	2.55933	2.95104	324
Tmem127	4.61624	0	0.615324	491
Tmem110	0.591053	0	0.685246	349
Tmem107	2.89812	1.7982	1.7204	180
Tmem104	3.28746	1.08938	0.615324	332
Tmeff1	2.10535	1.7982	1.57327	338
Tmed7	1.97487	1.46995	2.74827	182
Tmed4	33.5242	34.5911	44.9414	332
Tmed2	1.25544	1.14171	0.685246	185
Tmed1	3.23032	2.88758	2.09731	341
Tmco1	36.1723	22.1153	27.3586	191
Tmcc3	0.591053	1.41763	2.80392	180
Tmc7	1.5143	1.7982	1.57327	342
Tma7	9.29104	9.92428	3.32796	324
Tm9sf4	12.3943	8.82592	7.09002	504
Tln2	4.35231	0.761137	3.41924	353
Tlk1	6.78986	2.50701	1.64319	357
Tle3	2.10535	0.708814	0	510
Timp3	7.8421	5.6642	4.82726	347
Timp2	0.591053	0.380568	0.615324	499
Timm22	2.47063	3.11837	2.51441	334
Thumpd3	12.8597	7.37845	8.88431	352
Thsd7b	2.13751	2.70219	0.615324	179
Them4	5.35164	5.27272	3.05526	499
Tgtp2	0.591053	1.08938	11.1594	190
Tgoln2	54.4309	48.3287	63.5069	180
Tenm3	1.91983	2.50701	0.615324	499
Tecpr2	0.591053	0	0.685246	181
Tdrd3	4.40643	0.380568	0.866667	341

Tdrd1	0	0	1.10507	335
Tcea1	15.6771	9.7387	10.9806	353
Tbc1d5	30.5442	17.6205	26.6789	339
Tbc1d23	23.7581	12.9153	10.7802	331
Tbc1d16	0	0	0.615324	345
Tbc1d14	7.18669	4.16611	4.20814	332
Tapt1	1.25544	1.14171	1.02787	183
Tapbp	2.76974	1.7982	2.25852	335
Taf9b	30.5596	17.7643	20.7129	342
Taf7	13.1827	12.8965	7.74443	345
Tada1	4.69588	1.46995	3.14654	333
Tacr1	0.923247	0.708814	0	339
Tab3	0	0	1.64319	352
Tab1	5.04583	0.761137	2.40564	334
Syt9	0.591053	0.708814	2.06302	339
Syt16	2.10535	0.708814	1.48199	347
Syt1	79.8698	70.0984	90.4414	513
Syt12	0.923247	0.708814	1.64319	339
Sys1	0	0	0.615324	361
Synpo	4.11541	7.70365	8.40699	340
Synpo2	0.591053	0	0.615324	348
Synj2	10.5798	11.5004	12.2904	339
Syngr1	9.33836	7.92978	10.9774	340
Svil	0	0.708814	1.23065	331
Sv2b	124.741	129.561	116.173	187
Susd2	2.10535	1.41763	0	180
Sulf2	5.13396	3.92464	2.25852	183
Sucla2	91.9176	82.8119	73.7109	505
Sub1	58.9738	55.2142	40.9093	504
Stxbp1	52.8266	36.3676	37.532	331
Stx7	19.1491	7.00856	8.57003	348
Stx12	15.6779	8.29665	8.63731	498
Strip1	2.67445	2.10922	1.57327	173
Stmn2	168.498	104.166	31.2056	337
Stk4	18.7475	15.048	12.4307	662
Stk39	1.25544	1.14171	2.18859	176
Stk11	1.77316	1.41763	3.48916	188
Stk10	0.664388	0	0.615324	349
Stim1	0.591053	0	1.30057	353
Stc1	1.7512	1.08938	0.342623	348
Stat6	2.65523	1.46995	0.957947	345
Stat5a	0	0.380568	0.615324	340
Stat3	5.68383	2.17876	4.19151	329
Stat2	4.47589	6.41835	3.81498	344
Stard7	6.53941	0.380568	2.87384	487
St6galnac2	1.18211	0	0.957947	349
St3gal4	0.923247	1.41763	0.342623	349

St3gal2	1.18211	0	0.957947	500
Ssbp1	27.0605	15.9102	15.4474	372
Srxn1	1.5143	0	1.57327	361
Srsf3	15.5163	8.40681	10.5721	340
Srsf12	3.79941	0.708814	1.7204	335
Srsf10	45.5379	25.3325	28.3097	500
Srp72	7.09226	3.49894	6.10123	345
Sri	19.6945	19.2887	14.1769	178
Srebf2	12.603	7.85343	7.1813	175
Src	1.18211	0	0.685246	340
Spty2d1	2.95527	5.39703	2.94257	337
Sptssb	2.36421	1.08938	0.866667	348
Sprn	0.591053	2.17876	2.53122	335
Spink8	2.10535	0.708814	1.23065	340
Spink10	2.10535	2.7378	2.80392	505
Spice1	1.97487	1.08938	2.87384	490
Spdl1	0.923247	0.708814	1.23065	174
Spcs3	16.0126	9.32581	15.5727	500
Spcs2	44.6757	39.4059	32.6693	336
Spata2	0	2.50701	0.342623	346
Spag16	0	0	2.18859	182
Sox9	0.591053	0.708814	0.615324	1020
Sox13	0	0	0.957947	496
Sorbs1	25.5399	16.2171	11.1663	491
Son	234.647	211.778	187.685	334
Socs6	0.664388	0.380568	1.82461	185
Soat1	16.8601	12.9793	18.4922	181
Snapin	8.53765	10.9597	15.3396	500
Smg7	14.3799	11.6664	9.71607	175
Smg6	30.4535	10.853	20.4591	338
Smco1	0	0	0.957947	398
Smarcd1	0.664388	1.46995	0.957947	342
Slu7	32.8924	27.9608	31.1834	342
Slmap	6.7033	5.01402	12.2994	185
Slitrk5	6.98045	4.7381	2.18859	191
Slitrk1	25.6951	18.522	25.2174	501
Slit2	2.62105	0.380568	0.866667	331
Slfn5	1.18211	1.72865	1.57327	350
Slco3a1	0.332194	1.7982	0	331
Slc8a2	1.5143	2.40956	0.615324	498
Slc7a5	2.43755	0	0.615324	334
Slc6a17	5.06062	3.17069	4.17441	348
Slc6a11	5.10989	1.46995	2.18859	490
Slc45a4	0	0.708814	1.84597	366
Slc44a2	3.61965	0.380568	2.07596	333
Slc41a2	6.73546	1.7982	4.16033	327
Slc39a9	7.57928	3.42939	1.57327	521

Slc39a2	1.77316	0.708814	2.80392	348
Slc39a10	28.7495	17.0826	24.5525	524
Slc38a2	4.77707	2.02899	5.75496	342
Slc38a1	62.8279	37.7248	51.712	343
Slc37a3	6.43061	3.17069	9.85503	332
Slc35e3	1.25544	0	0.957947	324
Slc35c2	2.23373	0	2.43994	179
Slc35b4	3.15698	1.08938	1.57327	344
Slc35a4	5.0167	4.25704	4.32035	334
Slc35a2	4.37427	1.32017	2.53122	363
Slc2a3	102.108	102.243	66.877	344
Slc25a46	0	1.08938	1.02787	176
Slc22a15	1.18211	0.708814	0.342623	490
Slc19a3	0.332194	1.41763	0	188
Slc17a7	2.5214	3.1986	1.23065	329
Slc17a5	4.70436	3.54407	2.80392	348
Slc15a2	7.58262	2.10922	4.28591	341
Slc12a5	18.7304	11.7849	16.4165	342
Skp2	3.54632	0.708814	0.615324	362
Ski	1.32878	0.761137	0.685246	357
Sik1	0.664388	0.708814	0	189
Sidt2	0.332194	0.761137	1.57327	347
Siae	2.36421	1.08938	1.57327	341
Shisa9	0.923247	0	0.615324	342
Shisa5	13.2145	9.36091	15.1068	504
Shank1	29.4324	12.9646	17.2799	346
Sh3rf2	0	0	1.57327	333
Sh3kbp1	10.4675	3.44662	7.32823	347
Sgpp2	4.13737	1.08938	4.44711	498
Sgpl1	6.36533	1.14171	3.21646	481
Sgol1	1.58764	0.708814	0	329
Sf3b1	35.4885	20.1445	25.572	344
Setx	6.18558	5.44691	0.615324	344
Sertm1	0.923247	1.08938	0	510
Serpini1	36.4838	29.4399	44.2746	346
Serpine2	25.1283	20.9359	15.3512	480
Serpinb9	0	0	0.615324	354
Serpinb8	0	0.761137	2.43994	502
Serpinb11	0	0	1.23065	183
Serinc5	0.591053	0.380568	0.957947	346
Serinc3	18.5989	9.06845	14.3283	494
Serf2	7.30687	5.42277	5.7653	354
Sept3	6.13054	3.82718	3.97891	177
Sept10	3.57303	4.54668	1.82461	340
Sepn1	0	0	1.23065	335
Senp2	4.15356	1.46995	3.46781	498
Sema3c	4.41242	1.08938	1.23065	358

Selt	16.4462	12.7678	15.1301	336
Secisbp2l	5.31948	2.17876	2.53122	496
Sec61a1	14.5062	5.86985	1.89454	517
Sec16a	18.3554	13.2805	13.7253	340
Sdhaf2	10.2895	7.12324	5.80353	205
Sdf4	16.6695	12.8494	14.8591	335
Scrt1	17.1724	16.4061	56.9718	501
Scpep1	1.5143	0.380568	0.615324	505
Scoc	36.6926	29.1064	23.3715	527
Sco1	0.332194	0	1.64319	345
Scn3a	31.3061	19.7159	19.6277	334
Scml4	2.69641	1.7982	0.342623	350
Scd2	105.027	75.4519	70.3926	339
Sccpdh	14.9634	7.5922	10.319	184
Scarna8	2.36421	0.708814	0.342623	180
Scaf11	5.05852	2.88758	3.88763	501
Satb2	2.34225	0	1.23065	340
Sass6	5.0146	1.7982	2.33572	500
Sap18	10.2041	8.34413	10.4887	493
Samhd1	4.33909	1.08938	0.615324	505
Samd5	5.51752	6.05108	1.57327	177
Samd4b	1.77316	0.708814	1.02787	335
Samd3	0.591053	0.708814	0.615324	339
Sall1	2.36421	1.08938	0.615324	365
Rwdd4a	5.27285	5.48629	2.4613	177
Rusc1	8.5786	6.99788	8.9017	346
Rufy3	79.9153	43.3364	46.6356	357
Rtn4	70.4446	65.6031	93.9257	350
Rtkn	0.591053	0	0.615324	181
Rtf1	12.2669	7.19659	7.29959	341
Rsph4a	2.69641	1.08938	2.34866	836
Rsbn1l	1.77316	2.02899	1.23065	487
Rsbn1	0.332194	0.708814	0.342623	328
Rrnad1	4.16953	2.50701	3.29367	343
Rps6kb1	43.4777	23.2764	24.8448	530
Rps27	1.97487	0.380568	2.53122	170
Rprd2	17.3809	10.7914	7.71476	326
Rprd1a	16.5071	16.053	14.5916	490
Rpp14	9.17468	7.19232	5.81626	504
Rpn1	26.3301	16.0815	14.6425	507
Rorc	0	0	0.957947	340
Rock2	0.591053	0.708814	2.53122	339
Robo2	25.9306	16.4088	9.81401	507
Rnf43	2.43755	1.41763	6.00631	345
Rnf41	10.6407	2.9399	6.86569	346
Rnf40	4.73862	4.74299	4.44318	340
Rnf19a	4.37427	2.88758	2.18859	185

Rnf146	21.2449	25.2237	24.7271	335
Rnf145	1.5143	1.41763	0.615324	337
Rnf128	0.591053	2.12644	1.57327	324
Rnf122	2.43755	2.10922	1.23065	352
Rnf114	0	0.708814	0.615324	340
Rnd3	64.0707	42.9378	23.4937	188
Rnasel	8.26687	2.50701	2.67834	332
Rlf	3.54632	2.50701	4.71981	355
Riok3	3.33883	6.29859	3.02097	332
Rin2	2.82479	1.4004	0	183
Rims2	9.1892	4.7381	8.01348	488
Rhoq	1.5143	0	0.685246	347
Rhof	2.93331	1.7982	1.23065	353
Rhno1	1.77316	1.46995	1.91589	327
Rheb	17.976	17.5265	13.2292	508
Rgs3	0	0	0.615324	336
Rfx4	3.85321	4.07822	3.2852	183
Rfc1	21.5903	10.8702	13.9251	350
Rev3l	3.98401	2.08131	3.14654	353
Rell1	1.25544	1.08938	1.48199	490
Recql5	11.2182	5.70561	7.77451	326
Rdh1	0.591053	0	0.615324	539
Rdh10	0.332194	0	1.23065	184
Rbx1	20.4116	16.5588	19.78	182
Rbm41	1.25544	0.761137	1.57327	337
Rbm25	34.1053	21.7994	20.868	357
Rbm19	3.03912	2.50701	3.12518	344
Rbbp5	9.96788	5.73247	4.37719	358
Rbbp4	6.10858	3.97696	2.32844	348
Rbak	3.96236	1.46995	1.30057	342
Rassf4	10.2267	6.17458	6.13509	178
Rassf2	0.332194	2.81803	0.342623	330
Rassf1	6.39781	1.46995	1.84597	352
Rasgef1b	6.77725	4.87492	5.71529	343
Rapgef6	16.5329	6.15572	10.4784	334
Rapgef2	23.5021	13.5231	22.6218	337
Rap2a	6.87674	2.17876	3.14654	486
Ranbp3	5.59453	3.80996	3.37653	336
Ralgapb	0.332194	0	1.82461	348
Rala	6.88213	4.26008	3.83179	341
Rad51b	1.18211	0.708814	0.615324	178
Rad23a	12.8564	10.0707	13.5464	335
Rad17	3.15698	1.46995	1.30057	181
Rab5b	14.6495	6.66659	12.7128	330
Rab4a	7.8956	4.84702	5.01406	182
Rab43	1.77316	1.7982	0	330
Rab3gap2	5.85136	5.00095	5.75861	351

Rab3a	19.7669	11.089	15.366	173
Rab37	0	0	3.05526	343
Rab33b	2.51088	1.14171	2.80392	502
Rab32	0.332194	0.708814	0	495
Rab31	14.4978	11.6116	7.63173	350
Rab30	11.3813	9.28478	7.70262	334
Rab27a	6.77453	6.03385	5.91685	492
Rab23	5.00348	1.08938	0.957947	339
Rab12	5.53948	2.17876	1.30057	349
Rab11fip3	2.10535	0.380568	1.23065	347
Qser1	0	0.380568	1.30057	351
Qpct	8.43044	4.92725	4.9748	333
Qdpr	1.58764	2.55933	10.0823	342
Pxylp1	0.923247	0.708814	2.21015	329
Pxdn	4.67128	5.32504	1.48199	336
Pxdc1	0	0.708814	0.342623	331
Pvrl4	0	0.708814	0	196
Pus10	2.69641	1.46995	4.52432	350
Pttg1ip	19.7863	10.9616	16.1259	342
Pttg1	4.0901	2.55933	2.8008	185
Ptpro	5.53948	2.99222	1.91589	336
Ptpre	0	0	0.615324	332
Ptprd	33.4134	30.7211	22.8466	505
Ptpn3	0	0	1.64319	322
Ptgr2	23.4909	17.7421	15.5418	187
Ptgir	0.591053	0.708814	1.91589	337
Ptger1	0.591053	0	1.23065	171
Ptcd3	17.8356	14.4041	14.0699	184
Psmf1	7.32651	3.59639	3.89925	336
Psme3	9.25624	9.20502	5.84238	347
Psd	3.45873	0.708814	1.91589	339
Prune2	0.591053	0.708814	0	335
Prss35	6.2407	1.72865	0	512
Prr5l	0.591053	0.708814	4.10449	344
Prpf4b	80.4695	61.859	50.5312	185
Prpf4	9.5031	3.54407	7.58101	498
Prpf40a	29.4338	20.7825	15.1631	183
Prpf31	16.7102	11.4511	12.3176	345
Prpf18	50.8604	38.077	38.7224	519
Prob1	1.18211	0	0.957947	189
Prkx	0.591053	1.41763	0.342623	343
Prkcz	16.5083	5.91802	11.3072	169
Prkce	0.332194	0.708814	0.615324	483
Prkar2a	2.17869	2.10922	2.25852	508
Prkaa1	11.1324	8.22598	10.8659	325
Prelp	6.99432	5.55583	10.4909	340
Prdx6b	4.29517	2.89002	4.22148	346

Ppp6r3	24.1138	8.33718	14.8848	336
Ppp3ca	1.25544	1.52227	1.37049	351
Ppp2r5c	20.1426	5.39459	9.86329	336
Ppp2r3a	6.77664	7.19278	6.78283	354
Ppp2r2b	28.6037	14.7392	21.4611	337
Ppp2r2a	5.46615	3.64871	6.02058	335
Ppp1r26	0.591053	0.708814	0	486
Ppp1r2	6.18558	2.17876	5.9291	183
Ppp1r10	11.7483	10.255	12.0053	352
Ppm1k	5.75809	3.40149	6.9086	335
Ppm1a	2.43755	0.708814	1.30057	345
Ppig	15.9437	13.7784	16.041	349
Ppfibp1	22.9758	20.7456	12.0275	333
Pou6f1	1.77316	1.7982	1.7204	345
Pou3f2	2.80283	2.55933	0.342623	500
Pou2f2	0	0	0.615324	519
Pou2af1	0	0	0.615324	499
Pomt1	4.31713	3.11837	1.57327	185
Polr3g	4.50173	1.46995	0.342623	347
Polr3f	3.33883	2.12644	2.78256	351
Polr2l	1.91983	1.41763	1.84597	331
Podxl	0.591053	1.41763	0.615324	362
Pno1	9.56227	11.0151	7.15856	363
Pnkd	9.52948	5.98872	7.77882	353
Plxdc2	5.13396	1.90284	0.957947	518
Plxdc1	0.332194	0	0.615324	359
Plscr4	0.923247	0.380568	0.615324	504
Pls1	2.82479	2.17876	8.67258	531
Plp1	107.384	201.454	141.014	351
Plekhg1	7.5715	2.79012	4.10449	348
Plek	5.01883	1.7982	6.42282	341
Pld5	3.92989	1.08938	7.62738	360
Plch1	2.17869	2.43746	3.29367	342
Plcd1	1.5143	1.7982	0.342623	192
Plag1	0.923247	0	0.615324	333
Pla2g6	0.332194	0.380568	0.615324	181
Pla2g5	0	0.708814	0	340
Pla2g16	1.77316	3.26815	4.25161	493
Pkp4	1.18211	0.380568	0.957947	184
Pkm	70.5132	78.1092	79.1385	185
Pitpnc1	3.98493	2.9399	3.08154	502
Pip4k2c	3.61965	4.61623	4.33447	347
Pip4k2b	2.43755	1.08938	0.342623	497
Pik3cd	0	0.708814	0.615324	181
Pik3c2a	9.10553	10.5392	7.47322	338
Pigh	5.7848	1.41763	1.48199	336
Pias2	2.10535	1.41763	2.87384	352

Pianp	3.0265	2.17876	1.91589	342
Phtf2	7.49506	1.70074	2.51441	343
Phlda1	0.591053	0.708814	0	329
Phf5a	12.5825	3.82718	8.26879	333
Phactr3	14.5283	6.66244	15.6682	342
Phactr1	18.7624	17.1588	21.4692	518
Pgrmc2	1.32878	1.08938	1.57327	195
Pggt1b	6.47628	2.66059	6.01003	355
Pgbd5	1.5143	2.12644	1.57327	355
Pfn2	41.4727	30.6673	23.7003	343
Per3	3.10194	0.708814	0.957947	340
Per2	1.5143	0	2.40564	345
Peo1	18.023	7.60506	5.63845	511
Peli2	8.4863	3.75276	6.39932	181
Pdpk1	12.8326	9.53279	10.272	346
Pdp1	6.00006	10.5419	16.2614	347
Pdlim5	4.88896	1.46995	1.23065	514
Pdgfra	50.7393	39.6981	20.4624	343
Pde7b	5.11199	3.21582	7.41109	337
Pde5a	0.591053	1.08938	0.615324	327
Pde4b	25.4537	17.1061	29.948	334
Pdcl	36.8625	15.3224	15.4851	350
Pdcl3	14.5594	14.8323	8.68096	333
Pdcd7	0	1.41763	0.615324	193
Pcyt1a	8.7181	5.73732	5.08399	502
Pcdhb3	1.77316	2.43746	2.18859	340
Pcdhb18	7.75493	4.68577	5.51656	487
Pcdh9	161.472	141.427	111.779	491
Pcdh7	21.5371	18.0453	19.6321	340
Pcdh17	18.3104	14.9183	9.77748	504
Pcdh11x	20.4877	15.2003	7.21861	681
Paxip1	2.10535	0.708814	0.615324	337
Parp11	20.1174	9.87366	10.9549	338
Paqr7	1.25544	2.12644	1.57327	361
Pan3	0.332194	1.08938	0.342623	330
Pan2	1.84649	2.17876	3.02097	182
Palld	1.18211	0.708814	0.342623	352
Pak7	41.1916	19.8829	13.874	337
Paip1	6.08391	0.708814	1.64319	326
Pafah1b2	106.627	83.5938	87.3497	341
Pabpc5	1.77316	0.708814	0	180
P4hb	5.00348	0	2.87384	349
P4ha1	13.5674	10.343	5.93691	339
P2rx3	1.5143	1.7982	1.57327	335
Oxsr1	0.664388	0.380568	1.82461	328
Oxgr1	0	0	0.615324	344
Otud6b	37.423	21.5741	23.2017	342

Otud5	2.10535	2.83525	2.18859	499
Otud4	0.664388	1.85052	2.18859	333
Osbpl8	1.97487	1.46995	0.957947	329
Osbpl5	1.5143	1.70074	0	346
Olig1	1.64268	0.708814	0.342623	182
Olfm3	56.3156	47.0394	52.7593	341
Ogt	37.3626	21.9205	26.5214	338
Ogn	0.591053	0.761137	0	336
Ogfod1	27.4811	23.1523	36.2052	187
Oas2	0.591053	0	0.615324	328
Oas1d	0	0	0.615324	327
Nxph4	0	0	0.615324	171
Nxpe4	1.18211	1.72865	1.23065	348
Nxf1	21.326	18.0336	18.3764	183
Nus1	4.8751	3.54407	3.63629	339
Nup98	4.28194	0.708814	5.35669	338
Nup88	17.1996	12.9554	12.1315	349
Nudt16l1	2.36421	1.46995	4.25161	340
Nudt10	1.91983	0	0.685246	484
Nubp1	1.84649	0.761137	1.84597	498
Nub1	37.0404	27.7607	32.7616	356
Nts	32.8129	19.5924	10.0374	359
Ntrk3	16.4066	9.57653	14.6248	335
Ntng1	23.5531	15.2178	25.8083	184
Nsun6	4.873	2.50701	4.44711	367
Nsmf	4.00689	0.708814	1.20929	345
Nsf	56.8008	74.5861	66.8827	190
Nsa2	26.6233	19.9413	9.93422	343
Nrxn3	28.4558	16.2648	27.3328	338
Nrxn1	32.8051	15.0302	14.974	349
Nrp1	10.1904	7.45867	2.60114	330
Nrip3	12.3062	13.7004	15.2083	340
Nrg3	0.923247	3.59639	1.91589	344
Nrcam	81.6532	50.1553	53.8012	342
Nrbp2	1.58764	1.46995	2.4613	659
Nr2c1	2.69641	3.49894	1.91589	178
Nr1h5	0	0.708814	0	364
Npy1r	6.9402	2.87035	6.07623	348
Nphs1	0.591053	0.380568	0.615324	867
Npepps	2.69641	0.708814	0.957947	177
Npcd	10.2763	10.0984	6.99874	526
Npat	3.83459	4.58832	5.43879	497
Nov	10.4582	9.56451	5.48226	323
Nolc1	22.3354	8.98406	16.794	342
Nol6	23.0828	12.6328	9.39515	185
Noc3l	36.7235	22.3047	19.7726	339
Nnt	2.39576	2.17876	4.48461	345

Nlrp5-ps	0.332194	1.08938	0.342623	328
Nln	8.20281	4.15543	2.4613	182
Nlgn2	1.18211	0	0.615324	335
Nlgn1	51.8446	35.9608	44.8251	348
Nkx6-2	0	0.380568	0.615324	342
Nktr	7.80305	3.49894	5.59956	185
Nip7	24.1796	9.46869	7.56248	532
Nhsl1	4.70646	1.41763	2.53122	335
Ngfr	0.923247	0	0.615324	332
Nfatc3	0.591053	0.708814	0	335
Nek7	0.923247	1.08938	1.30057	338
Nek1	11.5128	4.9617	7.86635	345
Nedd4l	27.4849	19.9857	22.6725	346
Nedd1	5.10989	0.708814	1.55647	342
Necap1	22.1517	24.07	25.7879	494
Necab1	57.9103	42.439	31.0015	365
Nebl	13.6687	24.0916	8.99269	348
Ndst3	24.5613	18.5705	16.3101	340
Ndrg2	40.4506	30.7619	29.7773	176
Ncoa3	11.1302	6.62799	4.84558	326
Ncoa1	0.923247	0.380568	0.685246	175
Nckipsd	1.18211	0.708814	0	344
Nck2	0.923247	0	0.957947	187
Nceh1	10.9512	6.88968	3.93544	504
Ncdn	39.5872	37.1407	35.0752	180
Ncapg2	4.75513	1.08938	0.615324	336
Ncam1	130.149	73.1811	45.8917	356
Nbr1	26.6814	19.3296	21.9764	354
Nbeal1	8.27739	4.91657	5.65094	334
Nbea	0.923247	0	1.23065	344
Nat8l	0	0	0.615324	182
Narf	11.6431	4.50039	5.9373	335
Napg	31.0534	25.7713	28.1359	184
Napepld	2.36421	1.08938	0.615324	342
Napb	72.4415	83.0882	85.8703	343
Napa	30.7438	31.2188	35.836	324
Nap1l3	9.78327	2.12644	5.31378	351
Naip6	0	0.708814	0.342623	360
Nabp1	2.23373	0	0.957947	361
N28178	6.68134	2.55933	11.467	321
Myl12b	32.1609	30.3337	23.478	351
Myh10	92.083	45.6132	43.4884	328
Mxra8	0.591053	0	0.615324	186
Mxra7	0	1.70074	0	187
Mut	8.91507	2.81803	5.4952	343
Mum1l1	2.18921	2.02899	0.615324	512
Mum1	3.24083	0.708814	1.67692	498

Mturn	6.77064	2.17876	3.12518	179
Mtmr6	31.4435	27.6643	21.134	491
Mtmr4	4.05827	2.88758	3.81771	342
Mtmr14	2.69641	0.708814	0	346
Mtm1	9.36915	8.73887	9.87649	181
Mthfsd	6.16152	4.46645	6.23553	339
Mthfr	3.28746	0.380568	3.12974	352
Mtfr1	1.05163	1.46995	3.81771	340
Mterf4	3.07997	1.85052	2.32844	337
Mtcp1	3.17527	4.2989	5.71514	181
Msantd4	22.6494	16.5586	32.9667	174
Mrgprf	0.591053	0	0.615324	344
Mras	2.76974	5.27272	5.17393	345
Mpv17l	12.9062	8.4155	5.06243	495
Mprip	0.332194	1.14171	0.342623	357
Mpp5	21.4407	14.4386	28.5397	498
Mpp2	9.34345	3.59639	1.57327	344
Moxd1	0.332194	1.08938	1.30057	492
Mon1b	2.30707	3.49894	2.67834	337
Mobp	19.856	30.2529	26.7135	326
Mmp19	0	1.41763	0	333
Mmp13	5.44126	0.708814	0	346
Mmaa	3.28746	3.92464	6.31463	184
Mlxip	0.923247	0	0.615324	354
Mlst8	1.5143	1.46995	0	348
Mllt6	0.591053	0.761137	0.615324	365
Mllt3	11.1157	7.9016	4.52432	494
Mllt11	231.167	131.401	66.3083	353
Mkrn2	3.33883	1.4004	1.84597	336
Mkln1	39.8091	39.9245	37.3085	649
Mir5123	0	0	1.30057	486
Mir468	0	0	0.615324	358
Mif	29.2373	12.4517	11.4259	225
Mien1	18.8837	13.9982	13.6771	188
Mical3	17.6076	11.6274	11.1076	502
Mfsd6	1.18211	0.708814	0.957947	333
Mfap5	2.56593	1.32017	0	178
Mfap3	11.3188	5.83125	6.21417	492
Mfap1a	72.077	49.0659	44.8955	341
Mettl7a2	0.332194	0	0.685246	349
Mettl2	0.332194	0	1.23065	360
Mettl21a	7.66734	7.00984	8.40893	331
Met	1.25544	1.08938	0	342
Meis2	36.8219	12.9997	24.3348	343
Meis1	64.2043	33.1861	27.7095	353
Mef2c	36.8838	29.9888	22.4968	345
Mef2a	1.84649	3.82718	2.50986	175

Med26	0.332194	0	0.615324	496
Mecom	12.7439	5.22759	7.57958	653
Mdm4	34.3896	22.038	26.9972	328
Mdm2	8.70434	3.64871	3.02097	502
Mdh2	65.7442	49.6565	40.7279	346
Mctp1	2.76974	1.08938	1.57327	335
Mcph1	5.22116	2.37395	4.87931	492
Mcm4	4.9214	2.48979	2.60114	187
Mbnl1	6.08694	3.39378	6.8916	506
Matr3	91.7689	60.1405	54.5292	345
Mars2	1.18211	1.08938	0.957947	513
Marf1	3.0286	2.17876	2.78256	343
March1	31.7015	17.6003	18.0021	341
Mapkapk2	0	2.02899	0.615324	337
Mapk8ip3	42.8272	25.7883	45.1236	343
Mapk8	19.5256	12.6593	8.36103	501
Map6	29.5338	13.7608	6.61435	506
Map3k13	2.95527	1.46995	1.23065	339
Map2	45.4493	20.4736	27.2076	348
Map1a	48.7739	35.8021	38.2841	337
Man2a1	0.332194	0	1.57327	331
Maml3	0.591053	0	1.23065	342
Mak	0.332194	0	1.02787	330
Magi1	17.5342	7.33564	11.5016	184
Mageb16-ps1	2.23373	1.08938	0.615324	341
Macrod2	16.2494	9.90895	11.2212	332
M6pr	9.77993	8.37203	5.61363	333
Lyz2	2.06418	0.380568	0.957947	343
Lysmd4	4.59427	1.7982	2.16724	326
Ly6e	5.03866	6.23674	5.60784	351
Luc7l2	3.0286	0.708814	3.14654	348
Lsm6	16.2517	6.82202	8.47221	352
Lsm1	10.0953	4.78703	3.39788	335
Lrrtm4	28.6855	20.7939	13.0358	341
Lrrtm3	28.9165	17.9733	13.1605	342
Lrrn4cl	0	0.708814	0	344
Lrrfip2	1.18211	1.08938	1.7204	335
Lrrcc1	7.44493	3.26815	3.23802	500
Lrrc8a	1.84649	3.03044	1.84597	339
Lrrc59	6.0572	1.14171	0.685246	331
Lrrc40	11.4267	7.90113	9.12757	503
Lrp1	24.3608	13.3551	18.812	336
Lrp12	2.51088	1.08938	0.685246	185
Lrit2	0.591053	0.708814	0	340
Lrba	1.25544	0	3.69194	496
Lppr5	9.92093	5.44691	3.14654	502
Lppr1	9.95643	1.46995	2.18859	494

Lpl	6.47325	2.12644	0	338
Lpin1	2.85663	2.70219	4.06633	667
Lpar1	2.34225	1.72865	2.18859	489
Lonrf2	12.6631	8.82398	9.24413	343
Lmod3	3.54632	2.88758	0	190
Lmo4	18.1361	16.4992	12.1867	351
Lmo3	1.77316	0.708814	0.342623	343
Lmln	0	0.761137	0	352
Lix1l	2.23373	0.380568	3.14654	341
Lipm	0	0.708814	0	329
Lincpint	2.36421	5.01402	9.43962	350
Lin28b	1.18211	2.12644	2.87384	188
Limk2	10.0746	7.60845	8.68445	492
Limd1	0.332194	0	0.615324	495
Lima1	13.16	6.66193	6.2018	324
Lhx9	3.87851	0.708814	1.02787	348
Lgr4	2.10535	1.08938	1.57327	505
Lgalsl	20.1525	9.06706	10.0404	332
Leprotl1	3.15698	0	3.02097	332
Lepr	1.25544	1.08938	0	180
Lef1	2.85359	5.66901	6.2317	179
Ldoc1	0	1.41763	0.342623	338
Ldlrad3	0.996582	0.761137	0.342623	178
Lcp1	1.18211	0.708814	0.615324	349
Lclat1	8.13001	3.52684	6.30489	340
Lca5	6.08875	2.56178	2.50986	338
Laptm5	0.664388	1.01984	2.53122	1771
Lancl2	3.95185	3.92464	0.957947	341
Lamtor3	15.0975	10.6355	10.1184	180
l7Rn6	15.0005	8.18167	12.338	336
Ksr2	0.591053	1.08938	1.57327	492
Kpnb1	1.25544	0	0.866667	329
Kpna6	16.5377	10.4724	9.61812	342
Kpna1	34.4507	23.7082	40.681	339
Knop1	18.4521	14.2827	8.7822	497
Kndc1	2.10535	1.85052	0.685246	190
Klhl29	0.827951	1.46995	0.342623	339
Klhl28	14.3128	5.15804	10.1593	183
Klhl23	15.9227	7.09914	2.95104	346
Klhl20	2.85663	2.50701	2.53122	501
Klhl17	1.5143	0	0.615324	326
Klhl15	0	0.380568	2.80392	336
Klhdc7a	0	0	1.84597	333
Klf12	2.10535	0.708814	0.615324	350
Kif5c	19.7464	10.879	19.0533	341
Kif3a	34.9238	23.4795	15.0438	332
Kif26b	2.34225	0.380568	0.615324	186

Kif24	3.30365	0	0.685246	355
Kif21b	4.46957	0.761137	0.342623	522
Kif16b	1.25544	0.708814	0	510
Kidins220	50.4585	28.7835	26.5168	342
Khdrbs3	6.45464	6.9658	7.98238	356
Khdrbs1	11.9937	7.75598	3.41924	498
Kdm4a	47.5442	31.1551	25.7018	335
Kdm2a	1.5143	0	1.91589	352
Kdm1b	5.57206	1.08938	0.615324	346
Kdelr2	0.664388	0.761137	0	194
Kdelc2	1.99316	1.14171	1.91589	338
Kctd5	8.16256	6.43165	8.28618	337
Kctd13	1.5143	0	0.685246	344
Kctd10	1.77316	0	2.80392	509
Kcns3	0.332194	0.380568	1.23065	337
Kcnk2	16.7614	11.3031	6.6189	351
Kcnj9	9.17026	3.04882	11.953	510
Kcnj6	12.1262	10.5688	10.4986	516
Kcnj2	0.923247	0.708814	0	337
Kcnj12	0	0	0.615324	188
Kcnj10	3.61965	4.536	3.02097	341
Kcng4	0.664388	0	0.615324	339
Kcnd2	12.4466	12.4376	15.5588	501
Kcnc3	1.77316	0	2.4613	322
Kazn	2.43755	0.708814	1.30057	341
Kat7	7.39775	3.92464	6.25377	511
Kank1	2.69641	0.761137	1.84597	176
Jmjd4	1.5143	1.08938	0.342623	334
Jarid2	45.3482	23.8393	17.7282	342
Jam2	5.52119	4.30521	3.14654	516
Jakmip3	2.60111	2.55933	3.14654	493
Jade3	0.664388	1.41763	0.615324	514
Itpr2	9.9246	4.3403	2.53122	339
Itpr1	7.87441	10.2303	15.0305	499
Itpkb	0.591053	0.708814	2.16724	363
Itm2b	56.4382	31.0177	56.6769	344
Itk	0	0.708814	0	518
Itih2	2.69641	1.41763	0.615324	190
Itgb1	14.8626	6.33419	2.31436	338
Isy1	25.7916	20.5065	16.4727	179
Ist1	28.0587	15.4522	18.8906	346
Iqsec1	4.41242	3.21582	5.67776	347
Iqgap1	17.5985	6.46336	5.88073	517
Iqcg	1.7512	0	1.30057	348
Ipo9	15.9504	15.0415	13.0637	335
Ipo4	6.31606	3.59639	4.59424	497
Ipcef1	11.67	15.0119	16.3891	338

Ip6k1	0	2.17876	0.685246	349
Ints12	6.24202	3.92464	2.16724	325
Ints10	14.9298	12.8244	10.5645	197
Inpp5e	2.43755	2.83525	2.53122	480
Inpp5b	2.69641	0.708814	0.957947	184
Ino80c	4.05827	1.7982	2.50986	342
Ing3	12.7688	17.1646	7.51545	334
Impact	66.5208	64.8043	92.9104	179
Impa1	16.075	14.108	14.2013	500
Imp4	1.77316	1.32017	1.84597	180
Immt	16.9725	14.2343	13.977	330
Il33	6.57289	3.21582	11.464	179
Il1rap	6.27247	9.73042	11.3971	510
Ikzf4	0.591053	1.08938	1.84597	340
Ikbke	0	0	1.84597	343
Ikbkb	6.75322	4.19053	3.29367	327
Iigp1	0	0	2.21015	357
Igsf6	2.23373	3.14628	0.615324	506
Igf2	4.14757	1.46995	0.615324	504
Ift80	7.28351	4.74957	6.66943	347
Ifi203	8.39945	6.03385	0.615324	833
Ier5	2.95527	2.02899	2.80392	347
Idi1	19.4423	14.3304	14.2456	338
Ica1l	2.10535	1.08938	3.48916	340
Ibtk	3.52436	0.761137	2.16724	349
Iah1	2.17869	3.08276	3.05526	185
Hykk	0.591053	0.708814	0.957947	334
Hus1	3.98493	0.708814	3.63629	514
Hunk	0.827951	1.89946	1.30057	333
Hspa12a	9.50467	4.45879	5.48226	344
Hsf2	6.32658	3.59639	2.51441	338
Hsdl1	3.69089	3.26815	3.90899	325
Hs6st2	4.48576	1.7982	1.91589	499
Hrasls	4.83484	3.21582	6.73446	494
Hps3	4.24379	2.16154	2.69128	176
Hp1bp3	38.4384	22.643	30.5601	328
Hoxc13	1.26596	1.01984	0.342623	336
Hnrnpdl	59.5594	37.561	37.3224	329
Hn1l	3.72515	2.17876	1.84597	340
Hmgn3	165.26	130.033	98.4936	185
Hmgcr	7.96894	4.64064	3.29367	349
Hmg20a	73.3488	42.8212	46.6214	493
Hipk1	1.7512	2.12644	3.96211	328
Hif1a	5.16704	4.23566	2.80392	357
Hiat1	3.0286	0	0.615324	357
Hfe	0	0	0.615324	187
Herpud1	5.84558	3.03044	6.49216	342

Hecw1	15.9981	15.0604	6.7952	350
Heca	0.332194	1.41763	0	328
Heatr6	0.996582	1.7982	4.93686	330
Heatr1	1.58764	2.10922	1.57327	168
Hdx	10.3614	6.8404	9.98836	342
Hdlbp	26.921	20.6862	13.5474	340
Hcrtr2	1.18211	0.708814	0	333
Hbs1l	14.2267	7.40635	10.0587	184
Hapln2	0.591053	2.10922	5.95521	361
Hadha	45.6133	36.7025	24.2819	344
H2afj	4.90818	3.97696	4.32154	349
H19	23.0831	12.9694	1.23065	484
Gvin1	3.19216	2.55933	9.77346	344
Gtpbp1	1.25544	2.40956	0.957947	329
Gtf3c1	7.67638	8.91238	8.51985	181
Gtf2h5	21.6025	14.8556	11.485	332
Gsr	0.923247	0	1.84597	328
Gspt2	1.25544	2.50701	3.94329	183
Grsf1	11.8778	7.61876	14.1005	332
Grpel1	45.9192	29.3716	28.2661	349
Grip1	6.03524	1.46995	4.37719	169
Grik5	1.77316	1.08938	2.53122	168
Grik2	5.64965	4.30521	3.29367	332
Gria4	45.1814	40.0595	38.492	336
Gria1	135.074	84.8834	125.104	354
Greb1l	1.5143	0	2.18859	344
Gpr88	5.06062	1.08938	2.43994	351
Gpr26	38.0691	11.9668	22.3858	336
Gpr183	0	0	0.615324	184
Gpr17	15.9503	12.1216	0	348
Gpr155	5.61913	2.55933	2.69128	350
Gpr126	0	0	0.957947	346
Gpr101	1.16015	0.708814	1.10507	339
Gpm6b	504.18	403.875	419.369	184
Gpm6a	322.55	272.761	190.649	348
Gpd2	8.04595	3.11837	5.31378	500
Gpc1	0.591053	0	0.957947	494
Gpbp1	34.3254	26.0554	28.7894	331
Gpatch2l	3.52436	1.32017	2.18859	334
Gpaa1	1.77316	0	0.866667	189
Golph3	5.00173	4.63761	4.33307	188
Golm1	9.04695	2.98884	2.90757	485
Golga1	21.3365	13.4441	14.1312	351
Gnpnat1	6.89357	2.66059	4.06906	494
Gnl3l	79.079	71.267	79.2942	343
Gne	5.37085	1.08938	3.12518	344
Gnas	31.1472	24.0817	29.843	186

Gnal	18.4252	5.06634	8.73757	491
Gnai3	8.24965	8.49573	8.00226	331
Gmfb	79.0087	52.12	51.3872	492
Gm9776	1.18211	1.41763	0	348
Gm8898	22.0404	15.9329	13.173	333
Gm8615	2.36421	1.7982	1.64319	341
Gm7854	0.591053	0	0.615324	172
Gm5464	0	0	0.615324	499
Gm5113	3.28746	2.43746	1.57327	344
Gm4981	0	0	0.615324	188
Gm4944	2.43755	2.12644	1.23065	330
Gm4724	50.3501	37.5251	34.3871	179
Gm21119	5.77638	1.46995	12.726	333
Gm20337	0	0	0.957947	353
Gm20199	2.76974	2.50701	5.51656	504
Gm19522	0.923247	0	2.18859	513
Gm16023	2.10535	1.7982	0.615324	344
Gm15800	2.67445	1.08938	1.30057	351
Gm14405	1.32878	0	1.71311	1635
Gm13251	0	1.41763	1.23065	691
Gm10653	0	0.708814	0	183
Gm10649	2.10535	2.12644	1.57327	180
Gm10406	0.332194	0.708814	0.685246	339
Gm10336	1.97487	1.7982	2.67834	343
Gm10033	15.4938	9.16341	13.8294	321
Glul	72.6579	59.8381	96.8042	335
Gltp	5.60565	3.90741	1.64319	327
Glrx5	1.18211	1.41763	1.57327	195
Glrx3	27.4124	16.9441	16.3409	337
Glrx	14.8788	13.5279	7.68394	330
Glrx2	7.23931	3.1986	4.37719	357
Glb1l2	0.332194	0	0.957947	346
Gla	1.38382	1.7982	2.43994	346
Gjc3	8.5786	7.62944	10.5093	330
Git2	2.76974	3.49894	1.30057	182
Gimap8	0.591053	0	1.23065	365
Gimap6	9.80375	4.36145	2.96398	336
Gga2	0	0.708814	0.615324	342
Gfra1	1.7512	1.08938	3.07662	357
Gemin8	0.996582	0.380568	0.957947	683
Gdpd5	0	1.72865	0.342623	340
Gdpd1	12.2241	8.07352	8.36574	507
Gdap2	3.67103	5.70805	3.88763	180
Gdap1	22.5747	10.7262	16.0644	331
Gda	17.6731	17.1237	11.6978	660
Gcnt4	0.664388	1.08938	0.342623	335
Gbp6	0.591053	0	0.957947	361

Gbp4	0.664388	1.41763	0	510
Gatsl3	0	0	0.615324	346
Gatsl2	3.15698	1.08938	0.615324	340
Gatad2a	0	0.761137	2.53122	500
Ganab	11.1841	7.20954	7.37153	165
Gal3st3	13.7841	8.40592	7.66187	177
Gad2	8.06726	9.03992	5.04108	348
Gabrb3	17.6249	9.92815	8.77281	350
Gaa	5.7658	8.78234	11.2282	335
Fyttd1	20.0418	12.2654	13.1879	326
Fyn	2.51088	1.4004	3.29367	350
Fyb	0	0.708814	0	333
Fuom	2.44806	3.9794	3.86552	336
Ftsj1	1.25544	1.7982	4.01264	180
Fry	23.7067	10.3914	23.0041	330
Frs2	15.8294	12.1159	11.7915	339
Fpgt	4.57598	1.08938	4.48084	334
Foxn2	1.84649	1.14171	0	336
Foxl1	1.419	2.02899	0.342623	183
Foxj2	0	0.708814	0.685246	178
Fnip2	0	0.708814	0	666
Fmnl2	3.54632	0.708814	1.23065	339
Fmn2	8.14224	1.70074	7.87	486
Flt1	1.18211	0	0.615324	504
Flrt2	3.41584	2.17876	2.17179	489
Flrt1	0.332194	3.21582	1.98581	333
Fkbp5	0.332194	0.708814	0.957947	352
Fkbp10	2.60111	0.708814	0	346
Fgfr1op2	22.4426	23.7493	33.6612	346
Fgf14	77.3612	63.4631	69.1356	342
Fgf10	2.76974	1.7982	2.53122	361
Fez2	11.9042	5.19968	7.4372	183
Fem1b	4.00689	0	2.87384	344
Fcho1	8.78933	3.04116	5.2475	182
Fbxw2	8.25278	5.62538	8.06913	332
Fbxo3	16.4087	14.6692	11.6852	340
Fbxo31	0.996582	1.7982	2.87384	341
Fbxo27	0	0	1.57327	340
Fbxl22	0.591053	0	0.615324	341
Fbxl16	0	0.708814	0	335
Fbxl14	0	0	0.615324	342
Fbf1	15.0988	6.3621	10.2581	330
Farp1	3.91067	1.08938	1.57327	177
Far2	22.7496	12.7062	8.70895	502
Fam84b	0	0	0.615324	185
Fam49b	3.94975	2.23109	5.02427	345
Fam45a	17.9745	15.3796	19.7739	340

Fam3a	7.93678	4.58832	4.99251	348
Fam228b	0	0	0.615324	360
Fam228a	0	0	1.57327	330
Fam19a2	5.52291	4.48783	3.70502	344
Fam175a	0.923247	1.08938	2.13294	331
Fam172a	29.6865	19.2596	17.4181	345
Fam169a	3.95185	0.380568	3.41924	336
Fam168b	0.591053	1.08938	1.84597	323
Fam160b2	0.923247	2.81803	1.82461	352
Fam160b1	1.25544	0.708814	0.615324	512
Fam159b	0	0.380568	0.615324	358
Fam149a	0.923247	0.708814	0.615324	353
Fam133b	58.0187	38.5485	43.0107	504
Fam131b	0.332194	0.708814	0.685246	497
Fam124b	0.591053	0	0.615324	193
Fam122b	2.95527	1.41763	3.69194	347
Fam120b	10.6636	4.88096	10.2182	321
Fam102b	0	0.708814	0.342623	170
Fads3	1.5143	2.17876	0.342623	339
Fadd	0.591053	0.380568	0.615324	494
Eya1	3.0286	0	0.615324	186
Exosc4	3.15698	0	0.615324	180
Exoc2	10.9885	7.77353	13.5908	175
Ewsr1	19.7819	16.0701	21.9448	506
Evl	6.90712	2.88758	1.57327	175
Evc	0.332194	0.380568	0.615324	347
Etohi1	15.6009	13.7535	13.7751	515
Etf1	15.5854	13.6243	11.4302	332
Errfi1	0.923247	1.08938	0	344
Ermn	31.8501	52.6838	127.5	344
Eri2	3.00664	1.41763	0.957947	339
Erbb2ip	5.44419	4.61623	6.34893	348
Ephb1	13.9944	5.1676	5.08399	354
Epha5	7.31054	0.761137	2.53122	339
Epc2	7.58537	2.50701	3.7956	343
Epb4.2	0	0	0.615324	353
Epb4.1l1	3.19216	2.79012	3.41924	514
Entpd7	1.64268	0.708814	2.09731	378
Entpd5	3.28746	2.83525	4.10449	344
Enpp6	5.29752	4.23566	1.64319	332
Enox2	1.58764	0.708814	0.615324	349
Engase	1.25544	0.708814	0	181
Endod1	1.84649	1.08938	0	333
Emp2	4.72265	1.14171	0.342623	176
Emp1	1.64268	1.7982	0.615324	502
Eltd1	6.87247	6.61205	6.48724	337
Elp4	3.49969	3.21582	2.58706	489

Elmsan1	0	0.761137	0.615324	328
Ell2	0.664388	0	1.23065	339
Elk3	6.3894	1.85052	0.685246	343
Elavl2	63.7417	33.9555	30.142	340
Elavl1	0	0	0.615324	682
Eif4g2	14.1022	4.93816	4.33903	343
Eif4ebp2	0.332194	1.41763	0.342623	326
Eif4e2	40.1464	20.6969	13.7754	498
Eif4b	11.1803	2.17876	2.40564	510
Eif3j2	2.58422	2.87035	0.342623	359
Eid2b	2.10535	1.72865	0	335
Eid1	69.0444	32.5016	31.2605	347
Ehd4	0	0.708814	0	365
Ehd3	1.77316	0.761137	1.57327	348
Ehbp1	5.46615	3.64871	1.91589	368
Egfr	3.06168	0	0.957947	181
Egfem1	3.28746	1.85052	3.14654	518
Efcab2	5.02808	5.1676	4.65441	340
Eef2k	2.69641	0.708814	0.615324	348
Eef1a1	11.547	5.67466	5.71225	333
Edrf1	3.8947	1.7982	1.23065	353
Edil3	10.4696	5.39459	13.6732	509
Ect2	1.77316	0.380568	0.615324	515
Eci2	8.05582	3.54407	4.87931	171
E130311K13Rik	3.24628	4.8963	1.91589	200
E130307A14Rik	6.2941	0.761137	5.04108	490
Dyrk1a	3.54632	1.08938	3.39788	503
Dynll1	63.4819	44.1952	32.2682	656
Dut	7.50775	2.48979	1.37049	323
Dusp8	1.25544	0.380568	0.615324	346
Dusp27	0	0	0.615324	354
Dusp18	3.52436	2.12644	1.91589	349
Dusp16	0	0	0.685246	341
Duox1	0	0.708814	0	356
Dtl	1.84649	0.708814	0	178
Dtd2	2.53549	2.02899	1.98581	499
Drp2	5.66786	3.87951	5.09692	336
Drd1a	3.61965	3.21582	1.91589	348
Dpy19l1	0.332194	0.708814	1.91589	192
Dpp8	8.78459	2.99222	4.59424	333
Dpm1	18.7305	15.4614	19.8179	335
Dph6	22.8372	17.4502	17.6904	360
Dpf3	3.44465	0.380568	1.57327	499
Dock3	0.332194	0.761137	1.82461	330
Dock11	2.67445	1.08938	0.685246	189
Dnm3	21.8995	11.4251	14.9416	338
Dnm2	17.6988	12.0322	14.4006	336

Dnajc3	2.65523	1.41763	0	349
Dnajc16	3.65274	4.82864	4.92279	345
Dnajb5	5.16494	5.34226	1.91589	356
Dnajb4	39.3639	29.5988	38.5225	324
Dnaja4	3.82137	3.52684	5.80808	333
Dnaja1	221.396	145.055	112.334	337
Dlx5	1.7512	0.708814	1.57327	180
Dlst	5.52119	5.04912	2.60114	345
Dlg3	4.41242	8.27525	2.50986	334
Dleu7	1.05163	0.761137	1.10507	190
Dlc1	19.6415	8.77166	9.452	509
Dlat	9.18623	7.54921	3.12518	333
Dixdc1	3.94975	1.41763	3.12462	348
Dio2	23.8168	25.6674	12.9333	347
Dicer1	0.332194	0.380568	0.685246	334
Diablo	2.3804	3.95974	3.41612	342
Dhrs9	0	0	0.615324	353
Dhfr	3.72607	0	0.615324	346
Dgkg	2.21177	1.46995	3.12974	192
Dgke	2.10535	1.72865	0.615324	342
Derl2	11.1737	5.808	9.7425	341
Derl1	4.15356	1.08938	4.90123	338
Depdc5	10.5274	6.96227	7.22868	342
Dennd6a	2.26254	1.41763	1.91589	500
Degs2	0	0	0.615324	499
Dedd	2.58422	1.46995	1.57327	335
Decr2	2.17869	1.14171	1.20929	490
Ddx55	3.0286	5.55583	4.71981	181
Ddx3y	38.0174	27.3335	36.26	662
Ddx3x	29.8942	21.472	26.7146	493
Ddx19b	6.21866	1.46995	3.41924	322
Ddx11	2.82479	0.761137	0.342623	498
Ddi2	0.591053	0.380568	0.615324	491
Dcun1d4	17.1097	15.1048	13.1342	521
Dcun1d2	4.33909	2.48979	1.57327	333
Dctn4	27.2948	13.789	20.4492	501
Dclre1c	13.2472	14.5105	11.8148	338
Dclk3	1.97487	0	1.82461	187
Dclk2	0	0	0.615324	323
Dbndd2	6.49956	7.03813	8.52905	338
Dbndd1	1.25544	0.708814	0	354
Dbhos	0.591053	0.708814	0.615324	340
Dazap2	20.1904	16.2683	18.4032	340
Dapp1	0.591053	0.708814	1.23065	185
Dapl1	0	0.708814	0	185
Dagla	0.591053	0.380568	1.23065	529
Dad1	79.1878	53.1424	43.118	342

Dab2ip	2.69641	2.08131	4.37719	489
Dab1	6.89628	5.70561	10.1065	359
D7Ertd715e	125.553	89.2302	77.6616	343
D630039A03Rik	0	0.708814	0	180
D3Ertd254e	22.855	11.7678	15.6151	677
D2hgdh	2.10535	0.708814	3.07662	343
D230025D16Rik	2.5214	1.41763	2.78256	487
D10Wsu102e	8.20281	6.00595	4.13822	334
D10Bwg1379e	6.61759	2.88758	6.2848	338
D030025E07Rik	0.591053	0.708814	0	340
Cyp51	26.7261	11.9154	8.94072	485
Cyp3a11	0	0	0.615324	178
Cyp2s1	0	0.380568	1.57327	339
Cyp2j6	4.9214	6.59289	5.31378	350
Cyp2ab1	0	0.708814	0	523
Cyfip1	5.12586	6.31941	3.63629	181
Cybb	0	0	0.615324	335
Cyb5d2	1.7512	0.380568	1.23065	339
Cx3cl1	40.7785	41.528	37.9196	172
Cwf19l2	58.8947	33.9235	39.661	355
Cwf19l1	7.34573	3.21582	6.34836	340
Cwc25	32.8332	20.9653	21.6844	502
Cul5	3.95185	1.41763	3.97891	498
Cuedc1	1.18211	0	0.615324	343
Cttnbp2	18.3574	17.3321	20.3848	341
Ctso	1.77316	3.87951	4.90123	329
Ctsl	20.7446	15.7641	9.0848	498
Ctsb	52.5669	49.588	45.0618	346
Ctdsp1	0.591053	0	0.615324	337
Ctdnep1	0.996582	0	1.57327	187
Cstf2t	66.1075	43.1037	44.668	344
Cstf2	23.5208	10.8653	9.67844	345
Cst6	0	0	2.09731	489
Csrnp2	0	1.7982	0.615324	348
Csmd3	89.0323	55.4707	58.9622	493
Csf1r	7.37495	5.34226	2.40564	349
Csdc2	8.02334	3.59639	5.5281	335
Crtc3	0.591053	0	0.957947	518
Crispld1	0.591053	0.708814	1.23065	347
Crem	5.31371	7.25658	6.98561	495
Crebl2	0	2.50701	1.30057	354
Crbn	63.3917	72.1921	70.7766	338
Cramp1l	0.591053	0	0.957947	346
Cptp	5.50127	4.46645	4.26455	348
Cpne3	1.18211	1.46995	1.30057	501
Coq9	2.17869	5.08849	4.46866	348
Coq3	10.6882	7.52131	8.93268	352

Commd8	14.983	8.61457	9.94571	342
Col4a1	2.08339	2.70219	1.94962	341
Col26a1	0	1.08938	0.342623	323
Col25a1	4.28404	1.46995	2.53122	357
Col18a1	1.5143	0.708814	1.23065	347
Cnst	2.23373	2.7378	1.02787	505
Cnot6	3.92989	1.46995	4.77566	690
Cnot4	49.2131	32.8282	33.0016	487
Cnih4	14.0419	10.6076	14.0766	344
Cmtm6	3.87641	1.41763	2.67834	342
Cmpk1	29.0228	25.0747	14.8355	501
Clvs1	25.7421	17.2064	13.1831	511
Clptm1	9.4031	7.23954	6.63077	512
Clpb	2.51088	0.380568	1.84597	513
Clmp	1.97487	1.32017	1.30057	183
Clk1	58.6106	53.6397	38.1879	325
Clec1a	2.10535	0.708814	0.685246	359
Clec16a	11.7256	9.74636	14.7733	346
Cldn1	0.332194	0.708814	0	342
Clcn3	45.0854	28.3771	31.0456	354
Clcc1	6.30705	2.02899	4.92006	366
Cklf	2.08339	1.46995	1.23065	656
Ckap5	24.2991	12.8586	11.2159	182
Cipc	11.4136	6.05827	8.06204	335
Cidec	0	0	0.615324	509
Cidea	3.0265	0.708814	0	179
Ciao1	9.33625	5.75872	4.60717	344
Chst2	3.61965	1.4004	2.87384	342
Chst10	0.332194	1.08938	1.57327	187
Chrna3	1.18211	0.708814	0	176
Chpt1	2.67445	2.9399	2.25852	346
Chn1	54.7944	73.4454	109.668	343
Chmp3	20.2926	19.739	19.7208	511
Chmp2b	19.808	18.4462	8.50342	174
ChkbCpt1b	5.13396	1.08938	0.615324	175
Chek1	1.84649	0.708814	0.615324	335
Chchd5	5.06062	3.59639	2.18859	355
Cgnl1	2.97146	0.761137	2.93983	330
Cggbp1	9.66784	5.62538	7.48029	510
Cftr	1.18211	0.708814	0.957947	492
Cfd	0.591053	0.708814	0	341
Cep72	1.6559	2.7378	0	180
Cep70	14.9404	10.988	15.4512	329
Cep350	0.664388	0.380568	1.94962	179
Cep290	14.062	7.75182	15.0089	336
Cep120	6.34914	1.08938	5.06243	503
Cemip	7.20765	4.84214	3.5579	503

Celf3	7.95874	5.49585	4.61173	493
Cebpzos	4.70282	5.29713	3.50154	338
Ceacam2	0	0	0.615324	352
Cdon	0	0.708814	0	342
Cdk2	0.332194	2.55933	0	334
Cdk14	2.76974	1.85052	0.615324	341
Cdh7	9.01037	4.50039	5.74768	183
Cdc6	3.87851	0.708814	0.615324	324
Cdc42se1	5.43549	3.49894	1.23065	487
Cdc42ep4	1.97487	1.46995	0.957947	342
Cdc23	9.22524	8.66543	4.68855	351
Cdan1	0	1.08938	0.342623	346
Cd86	1.47313	0.708814	0	340
Cd53	3.37131	3.14628	1.7204	354
Cd44	4.48576	2.83525	0.957947	339
Cd36	3.61755	3.21582	1.02787	345
Cd34	12.2594	6.16719	7.1813	183
Cd276	0	0	0.615324	346
Cd24a	9.05514	4.13821	0	350
Cd247	0	0	0.615324	183
Cd164	14.1706	2.88758	10.3083	509
Ccr4	0	0	0.615324	349
Ccng1	4.41242	2.7378	3.21646	333
Ccnd1	3.1158	0.761137	0.615324	338
Ccnc	18.8466	9.93058	12.2258	336
Ccdc88a	12.4755	11.1812	8.36847	348
Ccdc77	3.72607	3.44662	2.53122	345
Ccdc38	0	0.708814	0	498
Ccdc32	4.44761	3.1986	2.53122	336
Ccdc177	2.10535	0.708814	0.615324	336
Ccdc169	2.08339	3.26815	7.79643	353
Ccdc157	0.923247	0.380568	2.43994	345
Ccdc14	0.923247	0.380568	1.3343	347
Ccdc120	0	0.708814	0.342623	507
Cbln2	1.5143	0	0.957947	177
Cbll1	1.84649	2.23109	1.57327	354
Cblb	2.82479	2.88758	3.29367	495
Casp8	3.0286	2.55933	2.80392	328
Casd1	39.2485	26.2593	20.0087	370
Casc4	19.4304	13.5621	18.5216	356
Carm1	0.923247	0.380568	0.615324	647
Carf	8.53743	7.04716	9.16299	352
Car12	4.79213	2.73292	7.27441	353
Caprin1	22.2165	10.1442	4.78973	333
Capn5	1.77316	0	0.957947	353
Cand1	4.41242	1.52227	3.46781	350
Camk2n1	43.1062	33.0848	56.3144	343

Calml3	0.332194	0.380568	1.4477	340
Calm3	332.711	250.551	177.395	351
Calm1	788.86	941.641	665.252	322
Cald1	18.9891	10.4477	9.20118	335
Calcrl	10.2031	7.97773	4.25161	337
Calcr	0.591053	1.41763	0.957947	340
Cadm1	40.0789	18.5592	17.7257	340
Cacng5	2.10535	2.60827	1.30057	326
Cacna2d1	4.61624	2.50701	5.96283	503
C77080	2.30707	1.08938	0	339
C330013F16Rik	0	0.708814	0	329
C230091D08Rik	59.2966	40.1427	60.9449	342
C1s2	0	0.380568	0.615324	182
C1qtnf6	0	0.708814	0.615324	338
C1ql3	5.52119	8.11517	8.58609	358
C1qb	2.10535	1.08938	0	187
C1d	39.3152	20.8197	19.1389	499
C130074G19Rik	2.26557	0.708814	0.957947	493
C030039L03Rik	9.37775	2.83525	3.83179	345
Bzw1	67.3054	57.5737	31.3592	344
Bzrap1	41.7053	22.7567	19.208	322
Bub3	14.8261	6.86067	8.93665	333
Btaf1	1.25544	1.01984	0.342623	347
Brsk2	2.10535	2.88758	4.50296	337
Brd3	9.09429	4.4123	1.57327	353
Brcc3	55.7658	52.4995	45.8653	348
Brat1	1.38382	2.40956	2.56495	353
Brap	1.25544	1.32017	0.957947	500
Bnip2	10.0541	6.25698	7.6438	334
Bmpr1a	2.36421	0	1.84597	320
Bmp1	1.5143	0.380568	2.18859	335
Blzf1	51.0429	36.9476	24.646	345
Bloc1s3	0	0	0.615324	178
Bivm	5.72975	3.21582	4.90123	333
Bin3	6.17749	8.46444	3.83595	337
Bin1	12.103	10.626	10.5576	190
Bet1	18.1057	19.2155	13.4414	347
Bdnf	6.68134	3.87951	6.11052	176
Bclaf1	42.9509	25.3452	23.547	336
Bcl11a	11.2564	7.95392	3.83179	504
BC035044	0.591053	0	2.80392	341
BC027231	0.923247	1.08938	0.866667	179
Bbip1	24.0758	9.60615	10.8232	355
Bard1	0.591053	0.708814	0	346
Bag5	9.1294	6.15817	9.58244	347
Bag4	0.332194	0.380568	1.57327	185
Bace1	0.923247	0	0.615324	323

B3gat2	0.332194	0.708814	0.342623	335
B3gat1	1.25544	0	0.615324	341
B230217O12Rik	13.9612	12.8148	12.0954	181
B230216N24Rik	8.07778	4.68577	3.05526	329
Azin1	70.3788	43.489	23.9466	330
AY512931	0	0.708814	0	342
AW554918	7.64587	8.07267	5.44351	335
AU023762	0.923247	0	1.91589	336
AU019823	2.36421	3.21582	0.957947	330
Atxn7l3b	42.472	32.5725	18.4313	347
Atxn3	5.82933	5.68839	4.76719	338
Atrx	28.4266	20.4597	19.5915	326
Atp7a	3.3608	2.46188	1.84597	493
Atp6v0d2	0	0.708814	0	343
Atp6v0a2	4.5429	2.50701	5.24386	390
Atp6ap1	18.4397	16.6618	11.8783	345
Atp2b1	136.725	121.243	113.446	341
Atp1a2	7.49717	8.06177	12.1366	186
Atg14	5.02808	2.73292	7.4361	334
Atf6	10.5926	3.90741	10.9509	494
Asxl2	3.96236	2.48979	0	509
Asic1	4.59427	1.46995	2.53122	662
Asb3	6.88305	8.84501	7.5155	336
Asap3	0.332194	0	0.957947	494
Arxes2	17.5147	8.54806	3.46781	185
Armc8	34.6229	14.8286	16.8583	502
Arl6ip1	43.316	33.3012	30.925	499
Arl6	23.8908	18.3168	15.0154	361
Arl15	12.2345	6.00595	5.73304	526
Arid3a	0.332194	0	0.685246	510
Arhgef9	29.8842	25.0656	28.4023	357
Arhgef6	1.5143	0	0.615324	181
Arhgef33	19.9328	17.7271	12.69	336
Arhgap6	6.50633	1.7982	5.3837	354
Arhgap44	2.69641	0.380568	1.91589	494
Arhgap39	0.332194	0	1.30057	175
Arhgap32	8.46924	14.0195	25.6555	181
Arhgap26	4.15356	2.12644	4.99251	338
Arhgap15	0	1.32017	0	335
Arfrp1	1.91983	1.08938	3.76186	199
Arcn1	76.1825	52.7006	45.1659	341
Arc	1.7512	2.7378	0.615324	334
Arap1	4.7798	0.708814	3.90899	331
Aqp9	0	0	0.615324	497
Aqp4	81.2828	54.6544	30.5408	347
Aplp2	176.589	189.169	165.703	357
Api5	11.0165	7.8698	13.0577	332

Aph1b	1.84649	0	1.23065	504
Ap2m1	56.8615	33.4883	31.8415	183
Ap2a2	2.23373	1.46995	1.57327	177
Ap1s3	0.923247	0	2.71264	357
Ap1g1	4.44761	4.61623	2.16724	321
Ap1b1	49.3081	28.0766	31.7774	338
Anp32a	332.66	271.983	240.412	185
Ano9	0	0	0.615324	350
Ano4	7.33731	11.2187	12.4613	331
Ano3	13.7195	17.5476	46.7923	344
Anks1b	51.6112	50.1886	45.332	513
Anks1	0	0	0.615324	495
Ankrd55	2.43755	1.08938	1.23065	333
Ankrd35	0.591053	0.380568	0.685246	334
Ankrd26	5.24037	3.85509	4.16033	324
Ankrd17	0.664388	1.46995	3.14654	347
Ankrd16	0	0.708814	0.342623	187
Ankra2	2.10535	2.23109	1.64319	330
Ankib1	8.52783	11.236	16.7105	341
Ank3	143.897	62.7702	57.1567	337
Ank2	112.843	74.0183	66.3834	502
Angel1	0	0.708814	1.23065	176
Amot	4.93014	1.08938	2.87384	326
Ammecr1	1.25544	0.708814	0.685246	558
Amd2	12.0488	10.1841	16.2445	342
Amacr	3.45873	1.7982	1.79032	177
Aldh6a1	9.93814	7.95885	9.88363	348
Aldh3a2	4.61624	4.63345	4.78973	353
Alcam	6.0572	2.87035	3.21646	488
Akt3	54.519	38.4746	55.1619	345
Akap17b	1.84649	0.380568	0.957947	495
Akap12	13.0414	2.81803	7.11138	179
Ak3	4.18875	5.44	4.01321	510
Aif1l	1.5143	0	1.84597	506
AI597479	27.6033	12.7128	13.5324	341
AI506816	0.332194	0.380568	0.957947	338
Ahsa2	4.02518	3.27059	1.23065	323
Ahctf1	0	0	1.4477	346
Agtpbp1	43.1832	24.28	15.8288	179
Agpat5	3.69299	1.08938	1.91589	339
Aftph	3.28746	0.761137	2.18859	340
AF529169	1.5143	1.41763	0.615324	189
Aen	3.3608	1.46995	3.63629	323
Adrbk2	7.7083	5.37736	7.7267	526
Adra1a	1.77316	3.82718	3.97891	343
Adipor2	0.923247	1.46995	4.25161	331
Add1	22.2211	14.1637	17.0224	345

Adcyap1r1	3.69299	1.08938	4.35583	337
Adcy6	7.29435	6.28187	2.18859	355
Adcy2	1.58764	1.08938	0.615324	178
Adck4	5.4566	3.21998	3.79635	182
Adarb2	15.229	12.4603	15.8711	535
Adarb1	0.591053	0.380568	1.57327	503
Adar	12.1357	5.77516	8.8847	500
Adamtsl1	0.923247	0.708814	1.94962	344
Adamts6	14.8352	3.59639	3.39788	348
Adamts3	6.4766	3.26815	2.67106	344
Adamts1	1.5143	2.18121	0.342623	184
Adam23	1.84649	0.380568	0.685246	326
Adam17	16.7357	11.4043	8.47309	180
Actr2	125.416	98.8075	98.9516	183
Actr10	6.49531	8.69336	3.76551	348
Acsl4	26.2131	18.1311	15.1069	337
Acp2	27.5121	16.3598	21.4914	349
Aco1	14.3906	8.86005	6.10632	324
Acer3	10.332	3.85509	3.36359	488
Acat1	90.5987	58.8684	33.2916	491
Acap3	2.34225	3.59639	2.18859	516
Acadsb	14.2796	13.5361	13.799	341
Acad8	8.41332	1.08938	3.21646	186
Abr	5.70305	6.81221	9.78224	344
Ablim3	2.51088	2.87035	1.57327	325
Abhd10	9.68199	8.14335	3.74051	511
Abcg1	1.5143	0.380568	1.57327	335
Abce1	36.469	28.8508	15.9737	184
Abcd3	13.6959	7.0074	5.27948	335
Abcd2	13.2071	13.0944	12.036	342
Abcb7	12.3282	10.3941	11.748	508
Abat	65.4173	64.311	55.273	349
Aar2	4.14757	4.11982	2.09731	348
AA543186	0.827951	0	2.09731	181
A930004D18Rik	0.591053	0.708814	0	331
A730020M07Rik	0.591053	0	0.615324	330
A630075F10Rik	0	0.708814	0	337
A430033K04Rik	9.60775	5.87289	7.23787	533
A330033J07Rik	0	0	0.615324	331
9930111J21Rik1	2.84308	3.57917	0.866667	336
9930012K11Rik	0.664388	0	0.685246	339
9630033F20Rik	10.9954	10.6071	8.00489	342
9630028H03Rik	0.591053	0.708814	0	489
9530080O11Rik	1.77316	1.7982	2.33572	338
9530068E07Rik	10.0485	1.7982	4.48615	336
9430083A17Rik	2.08339	0.708814	0.957947	184
9330159F19Rik	15.7996	10.5419	15.6799	528

9230112J17Rik	0	0.380568	1.4477	322
8430431K14Rik	4.13737	3.54407	2.53122	335
6820431F20Rik	72.2277	55.7689	55.5976	328
6530402F18Rik	0.591053	0.708814	0.342623	342
6430548M08Rik	4.7798	3.11837	5.08399	511
6330549D23Rik	0	0	2.09731	335
5930430L01Rik	2.21177	1.72865	4.5131	333
5730457N03Rik	0	0	0.615324	491
5730455P16Rik	14.6846	9.15467	7.22911	340
5430403N17Rik	0	0.708814	0	181
5330426P16Rik	9.42344	3.82718	7.85313	510
4933421O10Rik	0.591053	0	0.615324	337
4933411G06Rik	0	0	1.23065	182
4932438H23Rik	1.77316	0.708814	0	361
4932411E22Rik	2.36421	0.708814	0	173
4931429L15Rik	0	0.708814	0	187
4931414P19Rik	5.20771	5.0006	3.56637	513
4930539E08Rik	0.591053	1.46995	0	368
4930519F09Rik	4.67128	3.87951	1.89909	332
4930515G01Rik	1.5143	1.41763	0.866667	181
4930511M06Rik	0	0	0.615324	500
4930447N08Rik	0.591053	0	1.57327	179
4930442L01Rik	0	0	0.615324	335
4930425O10Rik	0	0	0.615324	497
4632428N05Rik	0	2.23109	1.20929	510
3830406C13Rik	8.02998	9.08871	6.45258	342
3110062M04Rik	0.591053	1.41763	0.615324	342
3110035E14Rik	54.7963	38.6953	62.1142	338
3000002C10Rik	0.332194	0.380568	1.30057	331
2900008C10Rik	1.38382	0	2.18859	179
2810021J22Rik	1.18211	1.46995	2.17179	502
2610301B20Rik	3.46721	3.83787	3.50154	495
2610044O15Rik8	36.2398	33.1254	20.476	335
2610008E11Rik	14.6296	5.39459	3.57931	542
2310061N02Rik	0	0.708814	0	340
2310001H17Rik	0.591053	1.32017	1.84597	181
2210408I21Rik	13.0922	8.89353	10.2413	350
2010012O05Rik	8.77533	4.5321	5.9895	337
1810064F22Rik	0	0	0.615324	193
1700003D09Rik	0.591053	0	0.957947	335
1500011B03Rik	9.86178	4.40647	5.19982	337
1110051M20Rik	27.6745	13.8965	16.7849	184
1110038B12Rik	31.336	10.3412	9.08378	181
1110037F02Rik	3.60821	2.88758	0	508
0610040F04Rik	1.5143	1.41763	1.23065	344

Supplementary Table S2: mRNA targets predicted using miRanda. The Gene names in addition to their

normalized CAGE expression throughout postnatal development in brain and the average miRanda score for each predicted gene target.
