# Supplementary material for: Characterization of piRNAs across postnatal development in mouse brain
Source: Sci Rep. 2016 Apr 26;6:25039. doi: 10.1038/srep25039 (PMC4844963; doi:10.1038/srep25039)
Supplement: Supplementary Table S3 [file srep25039-s3.pdf]

Gene Name	10 dpp	14 dpp	Adult
5830416I19Rik	0	0	0
Ahdc1	12.4984	2.7378	3.90899
Arcn1	76.1825	52.7006	45.1659
B4galt6	0	0	0
B630005N14Rik	1.25544	1.08938	0.342623
Bc1	0	0	0.342623
Camk4	26.2621	14.5017	10.3891
Cep128	3.28746	0.380568	0
Cep97	3.57303	2.98884	4.77509
Comt	1.84649	1.41763	2.18859
Dnal1	22.1745	17.2689	18.5021
Glb1	1.71601	2.17876	0.342623
Gm8234	0	0.380568	0.685246
Gna11	3.17527	1.46995	0.342623
Igf2bp2	0	0	0
Jkamp	8.88074	6.30578	10.1821
Kcnq1	0	0.380568	0
Kcnq1ot1	1.64268	0.761137	1.91589
Klhdc3	5.03656	4.02928	2.53122
Lars2	3.96236	2.17876	2.50986
Lmbrd1	8.98538	11.8051	13.0189
Mc1r	0	0	0
Mrpl19	7.46477	6.81847	5.10924
Nav1	11.6106	3.59639	1.23065
Nfia	33.7869	10.5856	6.99141
Nras	50.6035	38.924	46.8129
Ogfrl1	0.923247	0.761137	0
Pax6	12.5332	2.17876	0.342623
Plxna4	0.332194	0	0
Rapgef4	16.023	14.5468	23.6392
Rbm46os	0	0	0
Rn45s	216.059	131.681	148.229
Rn4.5s	352.341	198.255	213.714
Slx1b	0	1.08938	2.59721
Snrk	8.81341	7.91307	7.36272
Syt11	168.625	160.355	131.069
Thap2	2.43755	1.4004	0
Tmppe	1.71601	2.17876	0.342623
Trip4	16.7145	6.74267	10.1467
Trmt10a	21.5578	9.80688	17.1325
Zxda	0.664388	0.380568	0.342623

Supplementary Table S3: Predicted mRNA targets based on piRNA cleavage signature and their normalized CAGE expression throughout postnatal development in brain
