# Supplementary material for: Characterization of piRNAs across postnatal development in mouse brain
Source: Sci Rep. 2016 Apr 26;6:25039. doi: 10.1038/srep25039 (PMC4844963; doi:10.1038/srep25039)
Supplement: Supplementary Table S4 [file srep25039-s4.pdf]

Repeat Class	Repeat Family	Repeat Subfamily	Chromosome	Start	End	Strand	10 dpp	14 dpp	Adult
SINE	Alu	B1_Mus2	chr14	44062955	44063100	-	0	0	0
Simple_repeat	Simple_repeat	(A)n	chr14	44293009	44293043	+	0.923247	0.380568	1.84597
Simple_repeat	Simple_repeat	(A)n	chr14	44433069	44433104	+	0	0	0
SINE	Alu	B1_Mus2	chr14	44844922	44845065	+	0.591053	0	0
LTR	MaLR	MTA_Mm-int	chr14	44845066	44845846	+	0.591053	0	0
SINE	B2	B2_Mm1a	chr14	45456446	45456640	+	0	0	0
Simple_repeat	Simple_repeat	(A)n	chr14	45456641	45456684	+	0	0	0
LINE	L1	L1Md_T	chr14	45633332	45639965	-	0	0.708814	0
SINE	B2	B2_Mm1a	chr14	46189145	46189333	-	0.332194	0	0
SINE	Alu	B1_Mus2	chr14	46201983	46202128	-	0.591053	0	0
SINE	Alu	B1_Mm	chr10	8402574	8402721	+	0	0	0
Simple_repeat	Simple_repeat	(A)n	chr10	8402722	8402744	+	0.591053	0	0
SINE	Alu	B1_Mus2	chr14	47218365	47218512	-	0	0	0
LTR	MaLR	MTA_Mm	chr14	48243261	48243656	-	0	0	0
SINE	B4	B4	chr14	48631557	48631847	+	0	0	0
LTR	MaLR	MTA_Mm	chr14	49155242	49155635	+	0	0	0
SINE	Alu	B1_Mus2	chr14	49180083	49180220	+	0	0	0
Simple_repeat	Simple_repeat	(TTTA)n	chr14	49418185	49418224	+	0	0	0
SINE	B2	B2_Mm1t	chr14	50604083	50604274	+	0	0	0.342623
Simple_repeat	Simple_repeat	(A)n	chr14	51264803	51264846	+	0	0	0
SINE	Alu	B1_Mus2	chr10	59688638	59688784	-	0	0	0
SINE	Alu	B1_Mm	chr14	51625472	51625614	+	0	0	0
Simple_repeat	Simple_repeat	(A)n	chr14	51625615	51625647	+	0	0	0
LINE	L1	L1_Mus3	chr14	52087084	52093584	+	0	0	0
Simple_repeat	Simple_repeat	(A)n	chr14	52116735	52116775	+	0	0	0
Simple_repeat	Simple_repeat	(A)n	chr14	52118910	52118936	+	0	0	0
SINE	Alu	B1_Mus2	chr14	52592394	52592539	-	0	0	0
SINE	Alu	B1_Mus1	chr14	52691766	52691911	+	0.332194	0	0
Simple_repeat	Simple_repeat	(A)n	chr14	52691912	52691954	+	0	0	0
SINE	Alu	B1_Mus2	chr14	52859736	52859882	-	0	0.708814	0.342623
SINE	Alu	B1_Mus1	chr14	52874067	52874211	-	0	0	0.342623
LTR	MaLR	MTA_Mm	chr14	53044740	53045135	+	0	0	0
LTR	MaLR	MTA_Mm	chr14	53631391	53631786	+	0	0	0
SINE	Alu	B1_Mus2	chr14	53727913	53728061	+	0	0	0
Simple_repeat	Simple_repeat	(A)n	chr14	53728062	53728087	+	0	0	0
LTR	MaLR	MTA_Mm	chr14	53942513	53942908	+	0	0	0
LTR	MaLR	MTA_Mm	chr14	54331202	54331598	+	0	0	0
Simple_repeat	Simple_repeat	(A)n	chr14	54776580	54776616	+	0	0	0
SINE	Alu	B1_Mus1	chr14	54889908	54890055	-	0.332194	0.761137	0.957947
LTR	MaLR	MTA_Mm	chr14	54956750	54957144	-	0	0	0
Low_complexity	Low_complexity	A-rich	chr14	55112093	55112150	+	0	0	0
LTR	MaLR	MTB	chr14	55178677	55179074	-	0	0	0
SINE	Alu	B1_Mm	chr14	55225561	55225707	+	0	0	0
Simple_repeat	Simple_repeat	(A)n	chr14	55225708	55225728	+	0	0	0
Simple_repeat	Simple_repeat	(A)n	chr14	55404825	55404852	+	0	0	0
SINE	Alu	B1_Mm	chr14	56008850	56008978	-	0	0	0
Simple_repeat	Simple_repeat	(A)n	chr14	56109445	56109491	+	0	0	0.615324
SINE	Alu	B1_Mm	chr14	57021154	57021288	+	0	0	0
Simple_repeat	Simple_repeat	(A)n	chr14	57021289	57021314	+	0	0	0
LTR	ERVK	RMER4A	chr14	57280971	57281404	-	0	0.380568	0
SINE	Alu	B1_Mus1	chr14	57281405	57281552	-	0	0.380568	0
Simple_repeat	Simple_repeat	(A)n	chr14	57450005	57450042	+	0	0	0
Simple_repeat	Simple_repeat	(A)n	chr14	58528342	58528371	+	0	0	0
Simple_repeat	Simple_repeat	(A)n	chr14	59671374	59671403	+	0	0	0
Simple_repeat	Simple_repeat	(A)n	chr14	59676380	59676407	+	0	0	0
Simple_repeat	Simple_repeat	(A)n	chr14	59890064	59890100	+	0	0	0.957947
SINE	Alu	B1_Mm	chr14	60922559	60922700	-	0	0	0
Simple_repeat	Simple_repeat	(A)n	chr14	60976143	60976188	+	0	0	0
SINE	Alu	B1_Mus2	chr10	60619009	60619155	+	0	0	0
Low_complexity	Low_complexity	A-rich	chr10	60619156	60619212	+	0	0	0.615324
SINE	B2	B2_Mm1a	chr14	61742582	61742774	-	0	0	0
SINE	B4	B4A	chr14	62120452	62120754	-	0	0	0
SINE	Alu	B1_Mus1	chr14	62542202	62542348	-	0	0	0
SINE	Alu	B1_Mus1	chr14	62818141	62818292	-	0	0	0
LTR	MaLR	MTA_Mm	chr14	62890901	62891296	-	0	0	0
SINE	Alu	B1_Mm	chr14	63248178	63248313	+	0	0.380568	0

Simple_repeat	Simple_repeat	(GAAA)n	chr14	63248314	63248402	+	0.332194	0.380568	0
SINE	Alu	B1_Mm	chr14	64211192	64211337	+	0	0	0
Simple_repeat	Simple_repeat	(A)n	chr14	64211338	64211377	+	0	0	0
SINE	Alu	B1_Mus2	chr14	65264333	65264466	+	0	0	0
Simple_repeat	Simple_repeat	(A)n	chr14	65264467	65264487	+	0	0	0
SINE	B2	B2_Mm1t	chr14	65722060	65722255	+	0	0	0
SINE	B2	B2_Mm1a	chr14	65725949	65726134	-	0	0	0.342623
SINE	Alu	B1_Mm	chr14	65735920	65736065	+	0	0	0
Simple_repeat	Simple_repeat	(A)n	chr14	65736066	65736087	+	0	0	0
LTR	MaLR	MTA_Mm	chr14	65807962	65808357	+	0	0	0
SINE	Alu	B1_Mus1	chr14	66398026	66398172	-	0	0	0
SINE	Alu	B1_Mus2	chr14	66415713	66415857	-	0	0	0
Simple_repeat	Simple_repeat	(A)n	chr14	66643777	66643801	+	0.332194	0	0
LTR	ERVK	RLTR45	chr14	66643802	66644272	+	0.332194	0	0
LTR	MaLR	MTA_Mm	chr14	68083178	68083562	-	0	0	0
SINE	Alu	B1_Mus2	chr14	68216752	68216898	-	0	0	0
SINE	Alu	B1_Mus1	chr14	68565890	68566037	-	0	0	0
SINE	Alu	B1_Mm	chr14	68648354	68648499	+	0	0	0.342623
Low_complexity	Low_complexity	A-rich	chr14	68648500	68648542	+	0	0	0
SINE	Alu	B1_Mm	chr14	68678129	68678273	-	0	0	0
Simple_repeat	Simple_repeat	(A)n	chr14	69152100	69152134	+	0	0	0
Simple_repeat	Simple_repeat	(A)n	chr14	69377617	69377647	+	0	0	0
SINE	Alu	B1_Mm	chr14	69990500	69990641	+	1.18211	0	0.957947
Simple_repeat	Simple_repeat	(A)n	chr14	69990642	69990670	+	0	0	0.957947
SINE	Alu	B1_Mur4	chr14	70766020	70766197	-	0	0	0
Simple_repeat	Simple_repeat	(A)n	chr14	71914209	71914247	+	0	0	0
LINE	L1	L1Md_T	chr14	72184355	72191900	-	0	0	0
SINE	Alu	B1_Mur4	chr14	72333049	72333196	+	0	0	0
Simple_repeat	Simple_repeat	(A)n	chr14	72333197	72333220	+	0.332194	0	0
SINE	Alu	B1_Mm	chr14	72459596	72459741	-	0	0	0
Simple_repeat	Simple_repeat	(A)n	chr14	72549353	72549394	+	0	0	0
Simple_repeat	Simple_repeat	(A)n	chr14	72582849	72582901	+	0	0	0
LTR	MaLR	MTA_Mm	chr14	72774262	72774658	-	0	0	0
SINE	B2	B2_Mm1t	chr14	72844259	72844446	-	0	0	0
LTR	MaLR	MTA_Mm	chr14	73291278	73291672	-	0	0	0
SINE	Alu	B1_Mm	chr14	73405670	73405815	-	0	0	0
LTR	MaLR	MTA_Mm	chr14	74240571	74240965	+	0	0	0
Simple_repeat	Simple_repeat	(A)n	chr14	74658741	74658775	+	0	0	0
Simple_repeat	Simple_repeat	(A)n	chr14	75351422	75351462	+	0	0	0
SINE	Alu	B1_Mur4	chr14	75365503	75365650	-	0	0	0
SINE	Alu	B1_Mm	chr14	76238363	76238510	+	0	0	0
Low_complexity	Low_complexity	A-rich	chr14	76238511	76238557	+	0	0	0
DNA	MER1_type	RCHARR1	chr14	76423579	76424390	-	0	0	0
SINE	Alu	B1_Mm	chr14	76584030	76584176	+	0	0	0
Simple_repeat	Simple_repeat	(A)n	chr14	76584177	76584212	+	0.332194	0	0
SINE	Alu	B1_Mus2	chr14	76630104	76630249	+	0	0	0
Simple_repeat	Simple_repeat	(GAAA)n	chr14	76630250	76630297	+	0	0	0
Simple_repeat	Simple_repeat	(A)n	chr14	77093604	77093633	+	0	0	0
SINE	Alu	B1_Mus2	chr14	77339477	77339614	+	0.591053	0	0
SINE	Alu	B1_Mus2	chr14	78415097	78415241	+	0	0	0
Simple_repeat	Simple_repeat	(A)n	chr14	78415242	78415295	+	0	0	0
SINE	Alu	B1_Mm	chr14	78789667	78789813	+	0	0	0
Simple_repeat	Simple_repeat	(A)n	chr14	78789814	78789839	+	0	0	0
Simple_repeat	Simple_repeat	(A)n	chr14	79713665	79713693	+	0	0	0
SINE	Alu	B1_Mus2	chr10	61979292	61979436	-	0.591053	0	0
LINE	L1	L1Md_T	chr14	80228611	80236116	-	0	0	0
Simple_repeat	Simple_repeat	(A)n	chr14	80659908	80659979	+	0	0	0
scRNA	scRNA	BC1_Mm	chr14	80984419	80984567	-	0	0	0
Simple_repeat	Simple_repeat	(A)n	chr14	81701811	81701858	+	0	0	0
SINE	Alu	B1_Mm	chr10	62068311	62068455	-	0	0	0
SINE	Alu	B1_Mm	chr14	81927179	81927325	-	0	0	0
LINE	L1	L1Md_T	chr14	82190724	82197632	-	0.591053	0	0
SINE	Alu	B1_Mm	chr14	82886887	82887033	+	0	0	0
Simple_repeat	Simple_repeat	(A)n	chr14	82887034	82887059	+	0	0	0
Simple_repeat	Simple_repeat	(A)n	chr14	83388941	83388995	+	0	0	0
SINE	Alu	B1_Mus1	chr14	83421236	83421368	-	0	0	0
SINE	Alu	B1_Mm	chr14	83573066	83573211	+	0	0.708814	0

Simple_repeat	Simple_repeat	(A)n	chr14	83573212	83573242	+	0	0.708814	0
LINE	L1	L1_Mus1	chr14	84333070	84339502	-	0	0	0
Simple_repeat	Simple_repeat	(A)n	chr14	84546868	84546927	+	0.591053	0	0
LINE	L1	L1Md_F2	chr14	84709831	84715027	+	0	0	0
Simple_repeat	Simple_repeat	(A)n	chr14	84834692	84834741	+	0	0	0.342623
SINE	Alu	B1_Mus2	chr14	85312470	85312616	-	0	0	0
Simple_repeat	Simple_repeat	(A)n	chr14	85469753	85469789	+	0	0	0
Simple_repeat	Simple_repeat	(A)n	chr14	85495351	85495387	+	0	0	0
Simple_repeat	Simple_repeat	(A)n	chr14	85679649	85679674	+	0	0	0
SINE	Alu	B1_Mm	chr14	85909745	85909886	+	0	0	0
Simple_repeat	Simple_repeat	(A)n	chr14	85909887	85909920	+	0	0	0
Simple_repeat	Simple_repeat	(A)n	chr14	86545624	86545652	+	0	0	0
Low_complexity	Low_complexity	GA-rich	chr14	86545653	86545829	+	0	0	0
LTR	MaLR	MTB	chr14	87647964	87648361	+	0	0	0
LTR	MaLR	MTA_Mm	chr14	87718375	87718769	+	0	0	0
SINE	Alu	B1_Mm	chr10	62374352	62374490	-	0	0	0
LTR	MaLR	ORR1B1	chr14	88398406	88398728	-	0	0	0
SINE	Alu	B1_Mm	chr14	88622483	88622628	-	0	0	0
Simple_repeat	Simple_repeat	(A)n	chr14	89520173	89520204	+	0	0	0
Simple_repeat	Simple_repeat	(A)n	chr14	89980350	89980389	+	0	0	0
SINE	Alu	B1_Mus1	chr14	90834208	90834354	+	0	0	0
Simple_repeat	Simple_repeat	(A)n	chr14	90834355	90834400	+	0	0	0
Simple_repeat	Simple_repeat	(A)n	chr14	91204171	91204196	+	0	0	0
LTR	MaLR	MTA_Mm	chr14	91688330	91688727	+	0	0	0
SINE	Alu	B1_Mus1	chr14	92378666	92378813	+	0	0	0
Simple_repeat	Simple_repeat	(GAAA)n	chr14	92378815	92378886	+	0	0	0
Simple_repeat	Simple_repeat	(A)n	chr14	92405030	92405070	+	0	0	0
LTR	ERVK	RMER6C	chr14	92546049	92546656	+	0	0	0
SINE	Alu	B1_Mm	chr14	92782547	92782692	+	0	0	0
Simple_repeat	Simple_repeat	(A)n	chr14	92782693	92782715	+	0	0	0
Simple_repeat	Simple_repeat	(A)n	chr14	93016557	93016593	+	0	0	0
Simple_repeat	Simple_repeat	(A)n	chr14	93111829	93111907	+	0	0	0
Other	Other	RMER1B	chr14	95329437	95330393	+	0	0	0
SINE	Alu	B1_Mus2	chr14	95348287	95348431	+	0	0	0
Simple_repeat	Simple_repeat	(A)n	chr14	95348432	95348488	+	0	0	0.342623
LTR	MaLR	MTA_Mm	chr14	97045928	97046323	-	0	0	0
LTR	MaLR	MTA_Mm	chr14	98107339	98107733	+	0	0	0
SINE	Alu	B1_Mur3	chr10	62884591	62884733	-	0	0	0
SINE	Alu	B1_Mus2	chr14	99381824	99381970	+	0	0	0
Simple_repeat	Simple_repeat	(A)n	chr14	99381971	99381994	+	0	0	0
LTR	MaLR	MTA_Mm	chr14	99414897	99415290	-	0	0	0
SINE	Alu	B1_Mus2	chr14	99749395	99749543	-	0	0	0
LTR	MaLR	MLT1D	chr14	10017069	10017110	+	0	0	0
Low_complexity	Low_complexity	A-rich	chr14	10075723	10075731	+	0	0	0
SINE	Alu	B1_Mm	chr14	10095447	10095462	+	0	0	0
Simple_repeat	Simple_repeat	(A)n	chr14	10095462	10095465	+	0	0	0
SINE	Alu	B1_Mus1	chr14	10102155	10102167	-	0	0	0
SINE	B2	B2_Mm1a	chr14	10102481	10102500	+	0	0	0.342623
Simple_repeat	Simple_repeat	(A)n	chr14	10102500	10102503	+	0	0	0.342623
SINE	Alu	B1_Mus2	chr14	10103243	10103256	+	0	0	0
Simple_repeat	Simple_repeat	(A)n	chr14	10103256	10103261	+	0	0	0
Simple_repeat	Simple_repeat	(A)n	chr14	10133167	10133172	+	0	0	0
LINE	L1	L1_Mus3	chr14	10155160	10155750	-	0	0	0
SINE	Alu	B1_Mm	chr14	10178348	10178363	-	0	0	0
LTR	MaLR	MTB	chr14	10253322	10253359	-	0	0	0
Simple_repeat	Simple_repeat	(A)n	chr14	10257111	10257115	+	0	0	0
Simple_repeat	Simple_repeat	(A)n	chr14	10443039	10443043	+	0	0	0
SINE	Alu	PB1	chr14	10549469	10549479	+	0	0	0
Simple_repeat	Simple_repeat	(A)n	chr14	10557706	10557709	+	0	0.708814	1.48199
LTR	MaLR	MTA_Mm	chr14	10559163	10559202	+	0	0	0
SINE	Alu	B1_Mus2	chr14	10623259	10623274	-	0	0	0
SINE	Alu	B1_Mm	chr14	10623854	10623868	-	0	0	0
LTR	MaLR	MTA_Mm	chr14	10628477	10628515	+	0	0	0
LTR	MaLR	MTA_Mm	chr14	10650143	10650182	+	0	0	0
LINE	L1	L1Md_F2	chr14	10669398	10670009	-	0	0	0
SINE	Alu	B1_Mus1	chr14	10847815	10847830	-	0	0	0
SINE	Alu	B1_Mm	chr14	10918652	10918666	+	0	0	0

Simple_repeat	Simple_repeat	(A)n	chr14	10918667	10918670	+	0	0	0
SINE	Alu	B1_Mus2	chr14	10922869	10922884	+	0	0	0
Simple_repeat	Simple_repeat	(A)n	chr14	10922884	10922887	+	0	0	0
SINE	Alu	B1_Mus1	chr14	10923655	10923669	-	2.10535	2.12644	2.80392
LINE	L1	L1Md_F2	chr14	10962569	10963070	+	0	0	0
SINE	Alu	B1_Mus2	chr14	10999873	10999888	-	0	0	0
LTR	MaLR	MTA_Mm	chr14	11060949	11060988	+	0	0	0
LTR	MaLR	MTA_Mm	chr14	11160574	11160613	+	0	0	0
Simple_repeat	Simple_repeat	(A)n	chr10	9011677	9011712	+	0	0	0
Simple_repeat	Simple_repeat	(A)n	chr14	11164912	11164916	+	0	0	0
LINE	L1	L1_Mus1	chr14	11206243	11206848	+	0	0	0
SINE	Alu	B1_Mm	chr14	11213639	11213654	-	0	0	0
Simple_repeat	Simple_repeat	(A)n	chr10	9020387	9020425	+	0	0	0
Simple_repeat	Simple_repeat	(A)n	chr14	11237863	11237867	+	0	0	0
SINE	Alu	B1_Mus1	chr14	11241687	11241701	+	0	0	0
Simple_repeat	Simple_repeat	(A)n	chr14	11241701	11241704	+	0	0	0
SINE	Alu	B1_Mm	chr14	11321486	11321501	-	0	0	0
Simple_repeat	Simple_repeat	(A)n	chr14	11364173	11364177	+	0	0	0
LTR	MaLR	MTA_Mm	chr14	11378648	11378687	+	0	0	0
Simple_repeat	Simple_repeat	(A)n	chr14	11413550	11413554	+	0	0	0
Simple_repeat	Simple_repeat	(A)n	chr14	11440201	11440205	+	0	0	0
Simple_repeat	Simple_repeat	(A)n	chr14	11470015	11470018	+	0	0	0
LTR	MaLR	MTA_Mm	chr14	11492620	11492660	+	0	0	0
Simple_repeat	Simple_repeat	(A)n	chr14	11523098	11523107	+	0	0	0
SINE	Alu	B1_Mur4	chr14	11534407	11534420	+	0	0	0
Simple_repeat	Simple_repeat	(A)n	chr14	11534420	11534423	+	0.332194	0	0
SINE	Alu	B1_Mm	chr10	64495753	64495899	+	0	0	0
Simple_repeat	Simple_repeat	(A)n	chr10	64495900	64495943	+	0	0	0
LINE	L1	L1Md_T	chr10	64845963	64852617	-	0	0	0
SINE	Alu	B1_Mm	chr14	12035128	12035143	-	0	0	0
Simple_repeat	Simple_repeat	(A)n	chr14	12047952	12047955	+	0	0	0
SINE	Alu	B1_Mus2	chr14	12119072	12119086	-	0	0	0
SINE	Alu	B1_Mus1	chr14	12123753	12123766	-	0	0	0
LTR	MaLR	MTA_Mm	chr14	12129336	12129375	+	0	0	0
SINE	Alu	B1_Mus2	chr14	12182666	12182681	-	0	0	0
SINE	Alu	B1_Mm	chr14	12220424	12220438	-	0	0	0
SINE	Alu	B1_Mus2	chr14	12227381	12227396	+	0	0	0
Simple_repeat	Simple_repeat	(A)n	chr14	12227396	12227398	+	0	0	0
LTR	MaLR	MTA_Mm	chr14	12248761	12248800	+	0	0	0
Simple_repeat	Simple_repeat	(A)n	chr14	12289134	12289140	+	0	0	0.957947
SINE	Alu	B1_Mur3	chr14	12292958	12292971	+	0	0	0.615324
Simple_repeat	Simple_repeat	(A)n	chr14	12292971	12292975	+	0	0	0.615324
Simple_repeat	Simple_repeat	(A)n	chr10	65177157	65177211	+	0	0	0
Simple_repeat	Simple_repeat	(A)n	chr10	65207753	65207789	+	0	0	0
Simple_repeat	Simple_repeat	(A)n	chr15	3807997	3808039	+	0	0	0
LINE	L1	L1Md_F3	chr15	4657870	4663803	-	0	0	0
LTR	MaLR	MTA_Mm	chr15	4827160	4827554	+	0.332194	0	0
LTR	MaLR	MTA_Mm	chr15	4828647	4829019	+	0	0	0
LINE	L1	L1Md_F2	chr15	5677950	5683288	-	0	0	0.615324
LTR	MaLR	MTB	chr15	5790441	5790841	+	0	0	0
LTR	MaLR	MTA_Mm	chr15	5875425	5875819	-	0	0	0
LTR	MaLR	MTA_Mm	chr15	5950127	5950522	-	0	0	0
SINE	Alu	B1_Mus2	chr15	6467087	6467233	+	0	0	0
Simple_repeat	Simple_repeat	(A)n	chr15	6467234	6467277	+	0	0	0
LTR	MaLR	MTA_Mm	chr15	7003927	7004319	-	0	0	0
LTR	MaLR	MTA_Mm	chr15	7005413	7005805	-	0	0	0
SINE	Alu	B1_Mm	chr15	8054073	8054219	-	0	0	0
SINE	Alu	B1_Mus1	chr15	8398524	8398667	-	0	0	0
SINE	Alu	B1_Mus2	chr15	8538484	8538629	-	0	0	0
Simple_repeat	Simple_repeat	(A)n	chr15	8552489	8552525	+	0	0	0.342623
SINE	Alu	B1_Mus2	chr15	8667671	8667816	+	0	0	0
Simple_repeat	Simple_repeat	(A)n	chr15	8667817	8667842	+	0	0	0
LTR	MaLR	MTD	chr15	9858443	9858761	-	0	0	0
Simple_repeat	Simple_repeat	(A)n	chr15	9982582	9982638	+	0	0	0
SINE	Alu	B1_Mm	chr15	10044268	10044413	+	0	0	0
SINE	Alu	B1_Mus2	chr15	10375787	10375933	+	0	0	0
Simple_repeat	Simple_repeat	(A)n	chr15	10375934	10375959	+	0	0	0

LTR	MaLR	MTA_Mm	chr15	10839967	10840361	+	0	0	0
LTR	MaLR	MTA_Mm	chr15	11497143	11497536	-	0	0	0
Simple_repeat	Simple_repeat	(A)n	chr15	11960795	11960822	+	0	0	0
SINE	Alu	B1_Mus2	chr15	11993895	11994041	-	0	0	0
SINE	Alu	B1_Mus1	chr15	12132740	12132884	+	0	0	0
Simple_repeat	Simple_repeat	(A)n	chr15	12132896	12132932	+	0	0	0
LTR	ERV1	RLTR23	chr10	66240038	66240386	+	0	0	0
LTR	MaLR	MTC	chr15	12611227	12611700	+	0	0	0
LTR	MaLR	MTA_Mm	chr15	12624811	12625205	+	0	0	0
Simple_repeat	Simple_repeat	(A)n	chr15	14393360	14393405	+	0	0	0
LTR	MaLR	MTA_Mm	chr15	14439838	14440233	+	0	0	0
Simple_repeat	Simple_repeat	(A)n	chr15	14484912	14484938	+	0	0	0
Simple_repeat	Simple_repeat	(TTTA)n	chr15	14532786	14532831	+	0	0	0
Simple_repeat	Simple_repeat	(A)n	chr15	14694583	14694613	+	0	0	0
Simple_repeat	Simple_repeat	(A)n	chr15	14708750	14708775	+	0	0	0
SINE	Alu	B1_Mus1	chr10	66398035	66398181	+	0	0	0
Simple_repeat	Simple_repeat	(A)n	chr10	66398182	66398246	+	0	0	0
SINE	Alu	B1_Mm	chr10	66441809	66441961	+	0	0	0
LINE	L1	L1Md_T	chr15	15832021	15838443	-	0	0.380568	0
LTR	MaLR	MTA_Mm	chr15	15859483	15859876	-	0	0	0
Simple_repeat	Simple_repeat	(A)n	chr15	16019378	16019398	+	0	0	0.342623
SINE	Alu	B1_Mus2	chr15	16076084	16076223	-	0	0	0.342623
LTR	MaLR	MTA_Mm	chr15	16223362	16223756	-	0	0	0
SINE	Alu	B1_Mus2	chr15	17360675	17360820	+	0	0	0
LTR	MaLR	MTA_Mm	chr15	17408821	17409205	+	0	0	0
SINE	Alu	B1_Mus1	chr15	17572178	17572325	-	0	0	0.342623
LTR	MaLR	MTB	chr15	17782107	17782506	+	0	0	0
Simple_repeat	Simple_repeat	(A)n	chr15	17880567	17880673	+	0	0	0
Simple_repeat	Simple_repeat	(A)n	chr15	17976628	17976703	+	0	0	0
LINE	L1	L1Md_T	chr15	18594167	18600499	-	0	0	0
Simple_repeat	Simple_repeat	(A)n	chr15	19194615	19194645	+	0	0	0
LINE	L1	L1Md_F2	chr15	19226885	19232918	-	0	0	0
LINE	L1	L1Md_F2	chr15	19488473	19493557	+	0	0	0
Simple_repeat	Simple_repeat	(A)n	chr15	19863428	19863458	+	0	0	0
SINE	Alu	B1_Mus1	chr15	20052884	20053023	+	0	0	0
Simple_repeat	Simple_repeat	(A)n	chr15	20053024	20053094	+	0	0	0
LTR	MaLR	MTB	chr15	20719902	20720299	-	0	0	0
Simple_repeat	Simple_repeat	(A)n	chr15	20723717	20723784	+	0.591053	2.83525	0
LINE	L1	L1Md_F2	chr15	21839003	21844238	+	0	0	0
LINE	L1	L1Md_F2	chr15	22118695	22125281	-	0	0	0
SINE	Alu	B1_Mm	chr15	22322082	22322229	+	0	0	0
Simple_repeat	Simple_repeat	(A)n	chr15	22322230	22322254	+	0	0	0
SINE	Alu	B1_Mus1	chr15	22692075	22692202	-	0	0	0
Simple_repeat	Simple_repeat	(A)n	chr15	22762211	22762250	+	3.23032	2.43746	2.80392
SINE	Alu	B1_Mus2	chr15	22771370	22771515	-	0.591053	0	0
SINE	Alu	B1_Mus1	chr15	22929537	22929683	+	1.64268	1.85052	1.57327
Simple_repeat	Simple_repeat	(A)n	chr15	22929684	22929765	+	0.591053	1.85052	1.91589
Simple_repeat	Simple_repeat	(A)n	chr15	23708069	23708092	+	0	0	0
SINE	Alu	B1_Mus1	chr15	24976069	24976222	+	0	0	0
Simple_repeat	Simple_repeat	(A)n	chr15	24976223	24976264	+	0	0	0
Simple_repeat	Simple_repeat	(A)n	chr10	67111207	67111243	+	0	0	0
Simple_repeat	Simple_repeat	(A)n	chr15	26218792	26218830	+	0	0	0
LTR	MaLR	MTA_Mm	chr15	26948751	26949146	+	0	0	0
Simple_repeat	Simple_repeat	(A)n	chr15	26954149	26954183	+	0	0	0
LTR	MaLR	MTA_Mm	chr15	27141469	27141865	-	0	0	0
LTR	MaLR	MTA_Mm	chr15	27302761	27303155	+	0	0	0
Simple_repeat	Simple_repeat	(A)n	chr10	67300918	67300955	+	0	0	0
Simple_repeat	Simple_repeat	(A)n	chr15	28846888	28846973	+	0	0	0
SINE	Alu	B1_Mus2	chr15	28860535	28860683	-	0	0	0
SINE	Alu	B1_Mus2	chr15	31860530	31860676	-	0	0	0
LTR	MaLR	MTA_Mm	chr15	32005147	32005543	+	0	0	0
SINE	Alu	B1_Mus2	chr15	34367090	34367193	+	0	0	0
Simple_repeat	Simple_repeat	(A)n	chr15	34367194	34367224	+	0	0	0
Simple_repeat	Simple_repeat	(A)n	chr15	34609414	34609448	+	1.77316	0.708814	0.685246
Simple_repeat	Simple_repeat	(A)n	chr15	34690107	34690178	+	0	0	0
LINE	L1	L1_Mus2	chr15	35899137	35904590	-	0.591053	0	0
SINE	Alu	B1_Mus2	chr15	36567894	36568045	-	0	0	0

Simple_repeat	Simple_repeat	(A)n	chr15	36658916	36658938	+	0	0	0
Simple_repeat	Simple_repeat	(A)n	chr15	36764070	36764130	+	0	0	0
LTR	MaLR	MTA_Mm	chr15	36789268	36789664	-	0	0	0
LTR	MaLR	MTA_Mm	chr15	36790368	36790764	-	0	0	0
SINE	Alu	B1_Mm	chr15	37057208	37057290	-	0	0	0
Simple_repeat	Simple_repeat	(A)n	chr15	38105225	38105252	+	0	0	0
SINE	Alu	B1_Mus2	chr15	38124395	38124540	-	0	0	0
LTR	MaLR	MTA_Mm	chr15	38276168	38276561	+	0	0	0
SINE	Alu	B1_Mur4	chr15	38720895	38721041	+	0.591053	0.708814	0
Simple_repeat	Simple_repeat	(A)n	chr15	38721042	38721063	+	0.591053	1.08938	0
SINE	Alu	B1_Mus1	chr15	38723785	38723925	+	0	1.08938	0
Simple_repeat	Simple_repeat	(CAAA)n	chr15	38723926	38723977	+	0	1.08938	0.615324
SINE	Alu	B1_Mus1	chr15	39596807	39596952	-	0	0	0
scRNA	scRNA	4.5SRNA	chr15	39596953	39597044	-	0	0	0
SINE	B2	B2_Mm2	chr10	68503600	68503788	-	0	0	0
LTR	MaLR	MTA_Mm	chr15	40086067	40086460	-	0	0	0
LTR	MaLR	MTA_Mm	chr15	40114188	40114572	+	0	0	0
Simple_repeat	Simple_repeat	(A)n	chr15	40360695	40360745	+	0	0	0
Simple_repeat	Simple_repeat	(A)n	chr10	68561307	68561347	+	0	0	0
SINE	Alu	B1_Mus1	chr15	40460915	40461055	+	0	0	0
Simple_repeat	Simple_repeat	(A)n	chr15	40461056	40461088	+	0	0	0
LTR	MaLR	MTA_Mm	chr15	40974871	40975266	-	0	0	0
Simple_repeat	Simple_repeat	(A)n	chr10	68648895	68648930	+	0	0	0
LTR	MaLR	MTA_Mm	chr15	43209044	43209437	-	0.591053	0	0
Simple_repeat	Simple_repeat	(A)n	chr10	68786713	68786748	+	0	0	0
SINE	Alu	B1_Mus1	chr15	43703792	43703937	-	0	0	0
LTR	MaLR	MTA_Mm	chr15	43769725	43770119	-	0	0	0
LTR	MaLR	MTA_Mm	chr15	43873844	43874239	-	0	0	0
Simple_repeat	Simple_repeat	(A)n	chr10	68866747	68866786	+	0	0	0
Simple_repeat	Simple_repeat	(A)n	chr15	45222530	45222586	+	0	0	0
Simple_repeat	Simple_repeat	(A)n	chr15	45232684	45232716	+	0	0	0
LINE	L1	L1Md_T	chr15	45415068	45422145	-	0	0	0
Simple_repeat	Simple_repeat	(GAAA)n	chr15	45456999	45457160	+	0	0	0
SINE	Alu	B1_Mm	chr15	46154516	46154662	-	0	0	0
Simple_repeat	Simple_repeat	(A)n	chr15	46338493	46338529	+	0	0	0
SINE	Alu	B1_Mus2	chr15	46548015	46548157	-	0	0	0
Simple_repeat	Simple_repeat	(A)n	chr15	46917830	46917865	+	0	0	0
SINE	Alu	B1_Mus2	chr15	48883032	48883176	-	0	0	0
SINE	Alu	B1_Mus1	chr15	48889679	48889825	-	0	0	0
Simple_repeat	Simple_repeat	(A)n	chr15	49483091	49483123	+	0	0	0
LTR	MaLR	MTA_Mm	chr15	49752109	49752505	-	0	0	0
LTR	MaLR	MTA_Mm	chr15	50109869	50110265	-	0	0	0
Simple_repeat	Simple_repeat	(A)n	chr15	50425071	50425100	+	0	0	0
LTR	MaLR	MTA_Mm	chr15	52040744	52041138	-	0	0	0
SINE	Alu	B1_Mus1	chr15	52041206	52041335	+	0	0	0
LINE	L1	L1Md_T	chr15	52562376	52568805	-	0.591053	0	0
LINE	L1	L1Md_T	chr15	54036273	54042835	-	0	0	0
SINE	Alu	B1_Mus1	chr15	54234439	54234570	-	0	0	0
Simple_repeat	Simple_repeat	(A)n	chr15	55121571	55121650	+	0	0	0.342623
SINE	Alu	B1_Mm	chr10	69685115	69685256	+	0	0	0
Simple_repeat	Simple_repeat	(A)n	chr10	69685257	69685279	+	0	0	0
SINE	B2	B2_Mm1t	chr15	56545699	56545891	+	0	0	0
Simple_repeat	Simple_repeat	(A)n	chr15	56545892	56545916	+	0.664388	0	0
SINE	Alu	B1_Mus1	chr15	56946073	56946219	-	0	0	0
Simple_repeat	Simple_repeat	(A)n	chr15	57520881	57520932	+	0	0	0
SINE	Alu	B1_Mus1	chr15	57803702	57803847	-	0	0	0
Simple_repeat	Simple_repeat	(A)n	chr10	69901714	69901749	+	0	0	0
SINE	Alu	B1_Mus1	chr15	58933180	58933329	+	0	0	0
Simple_repeat	Simple_repeat	(CAAA)n	chr15	58933331	58933384	+	0	0	0
SINE	Alu	B1_Mus1	chr15	59434692	59434839	-	0	0	0
Simple_repeat	Simple_repeat	(A)n	chr15	59492871	59492895	+	0	0	0
SINE	Alu	B1_Mus1	chr15	60073931	60074077	-	0	0	0
SINE	Alu	B1_Mus1	chr15	60390706	60390841	+	0	0	0
Simple_repeat	Simple_repeat	(A)n	chr15	60390842	60390868	+	0	0	0
LTR	MaLR	MTA_Mm	chr15	60490096	60490491	+	0	0	0
LINE	L1	L1Md_F2	chr15	60918898	60924740	-	0	0	0.866667
SINE	Alu	B1_Mm	chr15	61070114	61070245	+	0	0	0

SINE	Alu	B1_Mm	chr15	61474873	61475019	-	0	0	0
SINE	Alu	B1_Mus1	chr15	61482345	61482491	+	0	0	0
Simple_repeat	Simple_repeat	(A)n	chr15	61482492	61482527	+	0	0	0
SINE	Alu	B1_Mm	chr15	61678052	61678183	-	0	0	0
SINE	B2	B2_Mm2	chr15	62084004	62084194	-	0	0	0
LINE	L1	L1Md_T	chr15	62518380	62525329	-	0	0	0
LINE	L1	L1Md_T	chr15	62595637	62601999	-	0	0	0
SINE	Alu	B1_Mus1	chr15	63224714	63224860	-	0	0	0
LTR	MaLR	MTB	chr15	63899778	63900148	-	0.591053	0	0
Simple_repeat	Simple_repeat	(A)n	chr15	64272356	64272396	+	0	0.380568	0
LINE	L1	L1_Mus1	chr15	64434059	64440297	-	0	0	0
Simple_repeat	Simple_repeat	(A)n	chr15	66807917	66807950	+	0	0	0
SINE	Alu	B1_Mm	chr15	67791574	67791720	+	0	0	0
Simple_repeat	Simple_repeat	(A)n	chr15	67791721	67791755	+	0	0	0
LTR	MaLR	MTA_Mm	chr15	67832761	67833162	-	0	0	0
LTR	MaLR	MTA_Mm	chr15	68535637	68536031	+	0	0	0
SINE	Alu	B1_Mus2	chr15	69508447	69508581	+	0	0	0
Simple_repeat	Simple_repeat	(A)n	chr15	69508582	69508610	+	0	0	0
LTR	MaLR	ORR1D2	chr15	70085287	70085634	-	0	0	0
SINE	Alu	B1_Mus2	chr15	70210401	70210547	-	0	0	0
LTR	MaLR	MTA_Mm	chr15	70279789	70280183	-	0	0	0
LTR	MaLR	MTA_Mm	chr15	70720534	70720928	+	0	0	0
LINE	L1	L1_Mus3	chr15	70902819	70908431	+	0	0	0
SINE	Alu	B1_Mus2	chr15	71901244	71901390	+	0	0	0
Simple_repeat	Simple_repeat	(A)n	chr15	72320428	72320463	+	0	0	0
SINE	Alu	B1_Mus1	chr15	73775312	73775458	+	0	0	0
Simple_repeat	Simple_repeat	(A)n	chr15	73775459	73775483	+	0	0	0
Simple_repeat	Simple_repeat	(A)n	chr15	74009693	74009741	+	0	0	0
LTR	MaLR	MTA_Mm	chr10	71331831	71332227	-	0	0	0
SINE	Alu	B1F	chr15	74704995	74705120	+	0	0	0
Simple_repeat	Simple_repeat	(A)n	chr15	74705121	74705145	+	0.591053	0	0
LINE	L1	L1Md_F	chr15	74930032	74934585	-	0	0	0
LTR	MaLR	MTA_Mm	chr15	75085896	75086297	+	0	0	0
SINE	B2	B2_Mm1a	chr10	71447435	71447625	-	0	0	0
SINE	B2	B2_Mm1a	chr15	75704296	75704483	-	0.332194	0	0
SINE	B2	B2_Mm1t	chr15	75705155	75705287	-	0	0	0
SINE	Alu	B1_Mus1	chr15	75705307	75705447	-	0	0	0
SINE	Alu	B1_Mm	chr15	76172913	76173047	-	0	0	0
SINE	Alu	B1_Mm	chr15	76361352	76361498	+	0	0.708814	0.342623
Simple_repeat	Simple_repeat	(A)n	chr15	76361499	76361540	+	0	0.708814	0.342623
SINE	Alu	B1_Mm	chr15	76660238	76660351	+	0	0	0
Simple_repeat	Simple_repeat	(A)n	chr15	76660352	76660395	+	0	0	0
Simple_repeat	Simple_repeat	(A)n	chr15	77153214	77153260	+	0	0	0
SINE	B2	B2_Mm2	chr15	77165556	77165743	+	0	0	0
Simple_repeat	Simple_repeat	(A)n	chr15	77165744	77165770	+	0	0	0
SINE	Alu	B1_Mm	chr15	77507257	77507402	-	0	0	0
SINE	Alu	B1_Mus2	chr15	77693991	77694137	+	0	0	0
SINE	Alu	B1_Mus1	chr15	77697941	77698087	-	0	0	0
LTR	MaLR	MTA_Mm	chr15	77700938	77701304	+	0	0	0
Simple_repeat	Simple_repeat	(A)n	chr15	78646632	78646661	+	0	0	0
SINE	Alu	B1_Mus2	chr15	78751588	78751734	-	0	0	0
Simple_repeat	Simple_repeat	(A)n	chr15	78893721	78893753	+	0	0	0
SINE	B4	B4A	chr15	79159325	79159535	-	0	0	0
SINE	Alu	B1_Mus2	chr15	79246418	79246564	-	0	0	0
SINE	Alu	B1_Mus2	chr15	79286518	79286663	+	0	0	0
Simple_repeat	Simple_repeat	(A)n	chr15	79286664	79286689	+	0	0	0.342623
SINE	Alu	B1_Mm	chr15	79385452	79385597	-	0	0	0
SINE	Alu	B1_Mus2	chr15	79386946	79387087	-	0	0	0
SINE	Alu	B1_Mus1	chr10	71932560	71932702	+	0	0	0
Simple_repeat	Simple_repeat	(A)n	chr10	71932703	71932797	+	0	0	0
LTR	MaLR	MTA_Mm	chr10	72017834	72018228	-	0	0	0
SINE	Alu	B1_Mus2	chr15	80439658	80439794	-	0	0	0
Simple_repeat	Simple_repeat	(A)n	chr15	80446059	80446085	+	0	0	0
Low_complexity	Low_complexity	A-rich	chr10	72084495	72084564	+	0	0.708814	0
SINE	Alu	B1_Mus2	chr10	72137753	72137899	+	6.86594	7.60204	14.3299
Simple_repeat	Simple_repeat	(A)n	chr10	72137900	72137922	+	1.77316	2.12644	2.18859
LTR	MaLR	MTB	chr10	72188701	72189085	+	0	0	0

Simple_repeat	Simple_repeat	(A)n	chr10	72244293	72244325	+	0	0	0
SINE	Alu	B1_Mus2	chr15	81658572	81658718	+	0	2.17876	0.685246
Simple_repeat	Simple_repeat	(A)n	chr15	81794624	81794649	+	0	0	0
SINE	B2	B2_Mm1a	chr15	81973809	81974002	+	0	0	0
Simple_repeat	Simple_repeat	(A)n	chr15	82392497	82392525	+	0	0	0
SINE	Alu	B1_Mm	chr15	82466231	82466377	+	0	0	0
Simple_repeat	Simple_repeat	(A)n	chr15	82466378	82466408	+	0	0	0
SINE	Alu	B1_Mus1	chr15	83014802	83014939	-	0	0	0
SINE	Alu	B1_Mus2	chr15	83428734	83428880	+	0	0	0
Simple_repeat	Simple_repeat	(A)n	chr15	83428881	83428911	+	0	0	0
SINE	Alu	B1_Mus1	chr10	72740036	72740182	+	1.5143	0	0
Simple_repeat	Simple_repeat	(A)n	chr10	72740183	72740208	+	1.5143	0	0
Simple_repeat	Simple_repeat	(A)n	chr10	72744935	72744970	+	1.05163	2.7378	0.615324
Simple_repeat	Simple_repeat	(A)n	chr15	85346790	85346819	+	0	0	0
SINE	B4	B4	chr15	85435549	85435802	-	0.591053	0	0.342623
SINE	Alu	B1_Mm	chr15	86022722	86022868	-	0	0	0
LTR	MaLR	MTA_Mm	chr15	86605478	86605872	-	0	0	0
SINE	Alu	B1_Mus1	chr15	87358153	87358299	+	0	0	0
Simple_repeat	Simple_repeat	(A)n	chr15	87358300	87358330	+	0	0	0
SINE	Alu	B1_Mur2	chr15	88454134	88454281	+	0	0	0
Simple_repeat	Simple_repeat	(A)n	chr15	88454282	88454307	+	0	0	0
SINE	Alu	B1_Mur3	chr15	89041325	89041465	+	0	0	0
Simple_repeat	Simple_repeat	(A)n	chr15	89041466	89041488	+	0	0	0
SINE	Alu	B1_Mus2	chr15	89214462	89214608	-	0	0	0
Simple_repeat	Simple_repeat	(A)n	chr15	89295381	89295458	+	0	0	0.342623
Simple_repeat	Simple_repeat	(A)n	chr15	89500371	89500437	+	0	0	0
SINE	Alu	B1_Mus2	chr15	89505794	89505940	-	0	0	0
LTR	MaLR	MTA_Mm	chr15	89751116	89751512	-	0	0	0
LTR	MaLR	MTB	chr15	90569600	90569992	+	0	0	0
LTR	MaLR	MTA_Mm	chr15	90589570	90589962	+	0	0	0
Simple_repeat	Simple_repeat	(A)n	chr15	91487022	91487069	+	0	0.380568	0.615324
Simple_repeat	Simple_repeat	(A)n	chr15	95447177	95447211	+	0	0	0
SINE	Alu	B1_Mus2	chr15	96238596	96238742	-	0	0	0
SINE	Alu	B1_Mur4	chr15	96505799	96505947	+	0	0	0
Simple_repeat	Simple_repeat	(A)n	chr15	96505948	96505970	+	0	0	0
Simple_repeat	Simple_repeat	(CCAA)n	chr15	96505969	96505998	+	0	0	0
SINE	Alu	B1_Mus2	chr15	96549722	96549868	+	0	0	0
Simple_repeat	Simple_repeat	(A)n	chr15	96549869	96549921	+	0	0	0
SINE	Alu	B1_Mus2	chr15	96912031	96912176	-	0	0	0
LTR	MaLR	MTB	chr15	97440885	97441268	-	0	0	0
SINE	B2	B2_Mm1t	chr15	98014059	98014243	-	0	0	0
Simple_repeat	Simple_repeat	(A)n	chr15	98089719	98089753	+	0	0	0
SINE	Alu	B1_Mm	chr15	98135001	98135146	+	0	0	0
Simple_repeat	Simple_repeat	(A)n	chr15	98135147	98135213	+	0	0	0
Simple_repeat	Simple_repeat	(A)n	chr15	98161455	98161498	+	0	0	0
SINE	Alu	PB1D7	chr15	98286021	98286148	-	0	0	0
Simple_repeat	Simple_repeat	(A)n	chr15	98475680	98475719	+	0	0	0
SINE	Alu	B1_Mus2	chr15	98855711	98855855	-	0	0	0
SINE	Alu	B1_Mus2	chr15	98876443	98876579	-	0	0	0
SINE	Alu	B1_Mus2	chr15	98896462	98896608	-	0	0	0
SINE	Alu	B1_Mm	chr15	98898166	98898315	+	0	0	0
Simple_repeat	Simple_repeat	(A)n	chr15	98898316	98898338	+	0	0	0
SINE	B2	B2_Mm2	chr15	99100231	99100418	-	0	0	0
LTR	MaLR	MTA_Mm	chr15	99387593	99387989	+	0	0	0
SINE	Alu	B1_Mus1	chr15	99782723	99782870	-	0	0	0
SINE	Alu	B1_Mus1	chr15	99862846	99862992	-	0	0	0
SINE	Alu	B1_Mm	chr15	10026898	10026912	+	0	0	0
Simple_repeat	Simple_repeat	(A)n	chr15	10026912	10026915	+	0	0	0
SINE	Alu	B1_Mm	chr15	10056549	10056564	+	0	0	0
Simple_repeat	Simple_repeat	(A)n	chr15	10056564	10056568	+	0	0	0
SINE	Alu	B1_Mus1	chr10	74672793	74672939	-	0	0	0
SINE	Alu	B1_Mur4	chr15	10100171	10100183	+	0	0	0
Low_complexity	Low_complexity	A-rich	chr15	10100183	10100189	+	0	0	0
SINE	Alu	B1_Mm	chr15	10212170	10212185	+	0	0	0
Simple_repeat	Simple_repeat	(CAAAAA)n	chr15	10212186	10212190	+	0	0	0
SINE	B2	B2_Mm1a	chr15	10222182	10222201	+	60.8852	34.5905	36.4764
SINE	Alu	B1_Mm	chr15	10247784	10247799	-	0	0	0

SINE	Alu	B1_Mm	chr15	10265363	10265375	-	0	0	0
SINE	Alu	B1_Mm	chr10	74968654	74968800	+	0	0	0
Simple_repeat	Simple_repeat	(A)n	chr10	74968801	74968836	+	0	0	0
SINE	Alu	B1_Mus1	chr16	4014802	4014951	-	0	0	0
Simple_repeat	Simple_repeat	(A)n	chr16	4235194	4235217	+	0	0	0
SINE	Alu	B1_Mus1	chr16	4448644	4448780	-	0	0	0
Simple_repeat	Simple_repeat	(A)n	chr16	4560096	4560130	+	0	0	0.342623
SINE	Alu	B1_Mm	chr16	4568679	4568825	-	0	0	0
SINE	Alu	B1_Mus2	chr16	4581159	4581304	+	0	0	0
Simple_repeat	Simple_repeat	(A)n	chr16	4581305	4581356	+	0	0	0
SINE	B2	B3	chr16	4870505	4870705	+	0	0	0
Simple_repeat	Simple_repeat	(A)n	chr16	8470347	8470381	+	0	0	0
SINE	Alu	B1_Mus1	chr16	8779667	8779814	-	0	0	0
SINE	Alu	B1_Mus2	chr16	8794616	8794761	-	0	0	0
SINE	B2	B2_Mm1a	chr16	8924187	8924394	+	0	0	0
SINE	Alu	PB1D9	chr10	75494233	75494352	+	0	0	0
SINE	Alu	B1_Mus1	chr16	10054680	10054826	-	0	0	0
Simple_repeat	Simple_repeat	(A)n	chr16	10816042	10816069	+	0	0	0
SINE	Alu	B1_Mus1	chr16	10903764	10903910	-	0	0	0
LTR	MaLR	ORR1A1	chr16	10949002	10949325	+	0	0	0
SINE	Alu	B1_Mus2	chr16	11955939	11956084	+	0	0	0
Simple_repeat	Simple_repeat	(A)n	chr16	11956085	11956107	+	0	0	0
LTR	MaLR	MTA_Mm	chr16	12388043	12388439	+	0	0	0
LTR	MaLR	MTA_Mm	chr16	12969444	12969838	-	0	0	0
SINE	Alu	B1_Mus1	chr16	13043111	13043257	-	0	0	0
SINE	B2	B2_Mm1a	chr16	13056655	13056840	+	0	0	0
Simple_repeat	Simple_repeat	(A)n	chr16	13435821	13435857	+	0	0	0
SINE	Alu	B1_Mm	chr16	13509001	13509146	-	0.332194	0	0
LTR	MaLR	MTA_Mm	chr16	14333143	14333536	+	0	0	0
LTR	MaLR	MTA_Mm	chr16	14534685	14535080	+	0	0	0
LTR	MaLR	MTA_Mm	chr16	15024876	15025255	-	0	0	0
SINE	Alu	B1_Mus2	chr16	15474966	15475111	+	0	0	0
Simple_repeat	Simple_repeat	(A)n	chr16	15475112	15475140	+	0	0	0
LTR	MaLR	MTA_Mm	chr10	76125170	76125563	+	0	0	0
SINE	Alu	B1_Mus1	chr16	15606704	15606849	+	0	0	0
Simple_repeat	Simple_repeat	(A)n	chr16	15606850	15606875	+	0	0	0
SINE	Alu	B1_Mus2	chr16	16856149	16856278	+	0	0	0
Simple_repeat	Simple_repeat	(A)n	chr16	16856279	16856303	+	0	0	0
LTR	ERVK	RMER13A	chr16	16856304	16857114	+	0	0	0
SINE	B2	B2_Mm1a	chr16	17231872	17232064	-	0	0	0
SINE	Alu	PB1D9	chr16	17266473	17266590	-	0	0	0
scRNA	scRNA	BC1_Mm	chr16	17724447	17724579	+	0	0	0
SINE	Alu	B1_Mus2	chr10	76450934	76451075	-	0	0	0
SINE	Alu	B1_Mus2	chr16	18675318	18675463	-	0	0	0.615324
SINE	Alu	B1_Mus1	chr16	18728464	18728595	-	0	0	0
SINE	Alu	B1_Mus2	chr16	18732273	18732417	-	0	0	0
Simple_repeat	Simple_repeat	(A)n	chr16	19127181	19127272	+	0	0.380568	0
SINE	Alu	B1_Mus1	chr10	76632881	76633027	-	0	0	0
LTR	MaLR	MTA_Mm	chr16	19875620	19876019	-	0	0	0
SINE	Alu	B1_Mus1	chr16	20068788	20068935	-	0	0	0
SINE	Alu	B1_Mus1	chr16	20138532	20138679	+	0	0	0
SINE	Alu	B1_Mus1	chr16	20437459	20437573	+	0	0	0
Simple_repeat	Simple_repeat	(A)n	chr16	20437574	20437596	+	0.332194	0	0
SINE	Alu	B1_Mus2	chr16	20495584	20495734	+	0	0	0
Simple_repeat	Simple_repeat	(A)n	chr16	20495735	20495769	+	0	0	0
SINE	Alu	B1_Mus1	chr16	20669264	20669411	-	0	0.380568	0
SINE	Alu	B1_Mus1	chr16	20671094	20671225	-	0	0	0
SINE	Alu	B1_Mm	chr16	21178963	21179109	+	0	0	0
Simple_repeat	Simple_repeat	(A)n	chr16	21369020	21369057	+	0	0	0
SINE	Alu	B1_Mus2	chr16	21697498	21697644	-	0	0	0
LTR	MaLR	MTA_Mm	chr16	21725778	21726165	+	0	0	0
SINE	Alu	B1_Mus2	chr10	76885020	76885164	+	0	0.708814	0
Simple_repeat	Simple_repeat	(A)n	chr10	76885165	76885214	+	0	0	0
SINE	Alu	B1_Mm	chr16	22199752	22199903	+	0	0	0
Simple_repeat	Simple_repeat	(A)n	chr16	22199904	22199926	+	0	0	0
Simple_repeat	Simple_repeat	(CAA)n	chr16	22199925	22199948	+	0	0	0
SINE	Alu	B1_Mus2	chr16	22215337	22215482	-	0	0	0

SINE	Alu	B1_Mus2	chr16	22238762	22238908	-	0	0	0
SINE	Alu	B1_Mm	chr16	22350954	22351102	+	0	0	0
Simple_repeat	Simple_repeat	(A)n	chr16	22351103	22351126	+	0	0	0
SINE	Alu	B1_Mm	chr16	22354254	22354400	-	0	0	0
Simple_repeat	Simple_repeat	(A)n	chr10	76989359	76989403	+	0	0	0.342623
Simple_repeat	Simple_repeat	(A)n	chr16	22758589	22758636	+	0	0	0
Simple_repeat	Simple_repeat	(A)n	chr10	77038004	77038059	+	0	0	0
Simple_repeat	Simple_repeat	(A)n	chr16	23200059	23200098	+	0	0	0
LTR	MaLR	MTA_Mm	chr16	23694555	23694952	-	0	0	0
Simple_repeat	Simple_repeat	(A)n	chr16	24136768	24136797	+	0	0	0
LTR	MaLR	MTA_Mm	chr10	77157696	77158094	-	0	0	0
SINE	Alu	B1_Mus2	chr10	77172550	77172696	-	0	0	0
SINE	Alu	B1_Mm	chr16	25018679	25018825	+	0	0	0.342623
Simple_repeat	Simple_repeat	(A)n	chr16	25018826	25018857	+	0	0	0.342623
SINE	Alu	B1_Mus1	chr16	25022133	25022279	-	0	0	0
SINE	Alu	B1_Mm	chr10	77209614	77209766	+	0	0	0
Simple_repeat	Simple_repeat	(A)n	chr10	77209767	77209793	+	0	0	0
Simple_repeat	Simple_repeat	(A)n	chr16	26814956	26814988	+	0	0	0
Simple_repeat	Simple_repeat	(A)n	chr16	27099973	27100001	+	0.591053	0	0
Simple_repeat	Simple_repeat	(A)n	chr16	27498761	27498789	+	0	0	0
LINE	L1	L1Md_F2	chr16	27691664	27696693	+	0	0.708814	0
SINE	Alu	B1_Mm	chr10	77474328	77474474	+	0	0	0.342623
Simple_repeat	Simple_repeat	(GAAAA)n	chr10	77474475	77474528	+	0.591053	0	0.342623
LTR	MaLR	MTA_Mm	chr16	27999488	27999881	+	0	0	0
LTR	MaLR	MTA_Mm	chr16	28147218	28147601	+	0	0	0
LTR	MaLR	MTA_Mm	chr16	28781778	28782173	+	0	0	0
LTR	MaLR	MTA_Mm	chr16	28783268	28783663	+	0.332194	0	0
SINE	Alu	B1_Mus2	chr16	29574703	29574862	-	0	0	0
SINE	B2	B2_Mm1a	chr16	29765099	29765291	-	0	1.01984	0
SINE	Alu	B1_Mm	chr16	29949683	29949822	+	0	0.708814	0
Simple_repeat	Simple_repeat	(A)n	chr16	29949823	29949846	+	0	0.708814	0
SINE	Alu	B1_Mm	chr16	30117953	30118104	-	0	0	0
SINE	Alu	B1_Mus2	chr16	30167029	30167175	-	0	0	0
Simple_repeat	Simple_repeat	(A)n	chr16	30185999	30186033	+	0	0	0
LTR	MaLR	MTA_Mm	chr16	30199502	30199897	+	0	0	0
LTR	MaLR	MTA_Mm	chr16	30685123	30685494	+	0	0	0
SINE	Alu	B1_Mus2	chr16	30767442	30767588	-	0	0	0
SINE	Alu	B1_Mus2	chr16	30874959	30875102	-	0	0	0
SINE	Alu	B1_Mm	chr10	77825532	77825670	+	0	0	0
Simple_repeat	Simple_repeat	(A)n	chr10	77825671	77825690	+	0.332194	0	0
SINE	Alu	B1_Mus2	chr16	31524608	31524741	+	0	0	0
Simple_repeat	Simple_repeat	(A)n	chr16	31524742	31524768	+	0	0	0
LTR	MaLR	MTC	chr16	31525303	31525651	-	0	0	0
SINE	Alu	B1_Mus2	chr16	31525652	31525798	-	0	0	0
SINE	Alu	B1_Mm	chr16	31625368	31625514	-	0	0	0
Simple_repeat	Simple_repeat	(A)n	chr10	77856501	77856534	+	36.1014	24.1903	20.2875
SINE	B2	B2_Mm1a	chr16	32207136	32207324	+	0	0	0
Simple_repeat	Simple_repeat	(A)n	chr16	32207325	32207348	+	0.591053	1.08938	0.615324
SINE	Alu	B1_Mus2	chr16	32260522	32260667	+	0	0	0
Simple_repeat	Simple_repeat	(A)n	chr16	32260676	32260728	+	0	0	0
LTR	MaLR	MTA_Mm	chr16	32596055	32596450	+	0	0	0
LTR	MaLR	MTA_Mm	chr16	32597542	32597937	+	0	0	0
Simple_repeat	Simple_repeat	(A)n	chr16	32687873	32687904	+	0	0	0
SINE	B2	B3	chr16	32687905	32688086	+	0	0	0
Simple_repeat	Simple_repeat	(A)n	chr10	77994813	77994833	+	0	0	0
Simple_repeat	Simple_repeat	(A)n	chr16	33142032	33142096	+	0	0	0
LTR	MaLR	MTA_Mm	chr16	33156072	33156463	-	0	0	0
SINE	Alu	B1_Mm	chr16	33166622	33166766	+	0	0	0
Simple_repeat	Simple_repeat	(A)n	chr16	33166767	33166793	+	0	0	0
SINE	Alu	B1_Mus2	chr16	33348726	33348872	-	0	0.708814	0
LINE	L1	L1Md_F2	chr10	78099785	78106008	-	0	0	0
SINE	Alu	B1_Mm	chr16	33825027	33825173	+	0	0	0
Simple_repeat	Simple_repeat	(A)n	chr16	33825174	33825196	+	0	0	0
LTR	MaLR	MTA_Mm	chr16	34626440	34626836	+	0	0	0
Simple_repeat	Simple_repeat	(A)n	chr16	34641967	34642025	+	0	0	0
Simple_repeat	Simple_repeat	(T)n	chr16	36096166	36096185	+	0	0	0
SINE	Alu	B1_Mm	chr16	36114423	36114569	-	0	0	0

SINE	Alu	B1_Mus1	chr16	36192594	36192740	-	0	0	0
LINE	L1	L1VL4	chr16	36574461	36577650	-	0	0	0
Simple_repeat	Simple_repeat	(A)n	chr16	37635667	37635706	+	0	0	0
SINE	Alu	B1_Mus2	chr16	38296354	38296498	+	0	0	0
SINE	Alu	B1_Mus2	chr16	38436732	38436880	-	0	0	0
SINE	Alu	B1_Mm	chr16	38557824	38557945	+	0	0	0
Simple_repeat	Simple_repeat	(A)n	chr16	38557946	38557969	+	0	0	0
LINE	L1	L1VL4	chr16	38734842	38739732	-	0	0	0
SINE	Alu	B1_Mus1	chr16	39033036	39033179	-	0	0	0
Simple_repeat	Simple_repeat	(A)n	chr16	39882022	39882085	+	0	0	0
SINE	Alu	B1_Mm	chr16	40461025	40461171	+	7.75265	6.0891	5.88752
Simple_repeat	Simple_repeat	(A)n	chr16	40461172	40461209	+	7.32516	7.17848	5.68474
LINE	L1	L1Md_T	chr16	40660159	40666852	-	0	0	0
Simple_repeat	Simple_repeat	(A)n	chr16	40765710	40765750	+	2.34225	0.380568	2.87384
SINE	Alu	B1_Mus1	chr16	40779703	40779849	+	1.64268	0	0.615324
Simple_repeat	Simple_repeat	(A)n	chr16	40779850	40779898	+	1.97487	0.380568	0
SINE	Alu	B1_Mm	chr16	40788581	40788731	+	1.84649	0.380568	0.615324
Simple_repeat	Simple_repeat	(A)n	chr16	40788732	40788757	+	1.5143	1.08938	0.957947
LTR	MaLR	MTA_Mm	chr16	40950225	40950623	+	1.18211	0.380568	0
Simple_repeat	Simple_repeat	(A)n	chr16	40980920	40980958	+	5.85339	0	1.57327
Simple_repeat	Simple_repeat	(A)n	chr16	41136291	41136331	+	3.98493	3.52684	0.615324
Simple_repeat	Simple_repeat	(A)n	chr16	41253870	41253902	+	4.20861	2.12644	7.18586
Simple_repeat	Simple_repeat	(A)n	chr16	42235820	42235848	+	0	0	0
SINE	Alu	B1_Mm	chr16	44345287	44345433	-	0	0	0
LTR	MaLR	MTA_Mm	chr16	45006088	45006469	+	0	0	0
Simple_repeat	Simple_repeat	(A)n	chr16	45046752	45046781	+	0	0	0
SINE	Alu	PB1D9	chr16	45710508	45710627	-	1.18211	0.708814	1.23065
SINE	Alu	B1_Mus2	chr10	10831361	10831505	+	0	0	0
Simple_repeat	Simple_repeat	(A)n	chr16	46280706	46280754	+	0	0	0
Simple_repeat	Simple_repeat	(A)n	chr16	46514674	46514720	+	0	0	0
LTR	MaLR	MTA_Mm	chr16	47099149	47099544	+	0	0	0
LTR	MaLR	MTA_Mm	chr16	47100637	47101032	+	0.591053	0	0
Simple_repeat	Simple_repeat	(A)n	chr16	47102003	47102031	+	0	0	0
LTR	MaLR	MTA_Mm	chr16	47270600	47270995	+	0	0	0
SINE	Alu	B1_Mus2	chr16	47782367	47782502	+	0	0	0
Simple_repeat	Simple_repeat	(A)n	chr16	47782503	47782525	+	0	0	0
SINE	Alu	B1_Mus2	chr16	48058707	48058855	-	0	0	0
SINE	Alu	B1_Mus1	chr16	48181940	48182093	+	0	0	0
Simple_repeat	Simple_repeat	(A)n	chr16	48304219	48304254	+	0	0	0
LTR	MaLR	MTA_Mm	chr16	48391230	48391625	-	0	0	0
SINE	Alu	B1_Mus1	chr16	48665677	48665824	+	0	0	0
Simple_repeat	Simple_repeat	(A)n	chr16	48665825	48665868	+	0	0	0
Simple_repeat	Simple_repeat	(A)n	chr16	48816786	48816821	+	0	0	0
SINE	Alu	B1_Mus2	chr16	48822778	48822924	-	0.923247	0.708814	0
Simple_repeat	Simple_repeat	(A)n	chr16	48862503	48862547	+	0	0	0
SINE	Alu	B1_Mm	chr16	48900963	48901109	+	0	0	0
SINE	Alu	B1_Mus2	chr10	79626268	79626417	+	0	0	0
Simple_repeat	Simple_repeat	(A)n	chr10	79626418	79626446	+	0	0	0
Simple_repeat	Simple_repeat	(A)n	chr16	49227237	49227259	+	0	0	0
LTR	MaLR	MTA_Mm	chr16	49323930	49324308	-	0	0	0
SINE	B2	B2_Mm1a	chr10	79674245	79674436	+	0	0	0
SINE	Alu	B1_Mus1	chr10	79783710	79783851	+	0	0	0
Simple_repeat	Simple_repeat	(A)n	chr10	79783852	79783874	+	0	0	0
Simple_repeat	Simple_repeat	(A)n	chr16	51207955	51207997	+	0.332194	0	0
LINE	L1	L1Md_F2	chr16	51235920	51241841	-	0	0	0
SINE	Alu	B1_Mm	chr16	51588031	51588172	-	0	0	0
LTR	MaLR	MTA_Mm	chr16	51834667	51835062	+	0.332194	0	0
SINE	Alu	B1_Mm	chr10	79906684	79906830	-	0	0	0
Simple_repeat	Simple_repeat	(A)n	chr16	53153637	53153665	+	6.22949	8.29006	14.1657
SINE	B2	B2_Mm1t	chr16	53271536	53271727	+	0	0	0
Low_complexity	Low_complexity	A-rich	chr16	53271728	53271810	+	0	0	0
Simple_repeat	Simple_repeat	(A)n	chr16	53601351	53601439	+	0	0	0
Simple_repeat	Simple_repeat	(A)n	chr16	53858837	53858878	+	0	0	0.342623
Simple_repeat	Simple_repeat	(A)n	chr16	53913303	53913329	+	0	0	0
SINE	Alu	B1_Mm	chr16	53929746	53929898	+	0	0	0
Simple_repeat	Simple_repeat	(A)n	chr16	54108342	54108383	+	0	0	0
Simple_repeat	Simple_repeat	(A)n	chr16	54724747	54724782	+	0	0	0

LTR	MaLR	MTA_Mm	chr16	54935952	54936267	+	0	0	0
LTR	MaLR	MTA_Mm	chr16	55217169	55217569	+	0	0	0
SINE	Alu	B1_Mus2	chr10	80045747	80045883	+	0	0	0
SINE	Alu	B1_Mus1	chr16	55757854	55758013	-	0	0	0
SINE	Alu	B1_Mus2	chr16	55899587	55899738	-	0	0	0
SINE	Alu	PB1D9	chr16	56057240	56057312	+	0	0	0
SINE	B2	B2_Mm1a	chr10	80124687	80124879	+	0	0	0
LINE	L1	L1_Mus2	chr16	56959159	56965602	+	0	0	0
Simple_repeat	Simple_repeat	(A)n	chr16	57890992	57891019	+	0	0	0
SINE	Alu	B1_Mus1	chr16	59092679	59092830	+	0	0	0
SINE	Alu	B1_Mus1	chr10	80387272	80387402	-	0	0	0
SINE	Alu	B1_Mm	chr16	60965637	60965783	-	0	0	0
SINE	Alu	B1_Mus2	chr16	61059989	61060137	-	0	0	0
LINE	L1	L1Md_F2	chr16	61304654	61310539	-	0	0	0
SINE	Alu	B1_Mus1	chr16	61398582	61398726	-	0	0	0
Simple_repeat	Simple_repeat	(A)n	chr16	61487811	61487861	+	0	0	0
Simple_repeat	Simple_repeat	(A)n	chr16	61967774	61967802	+	0	0	0
LTR	MaLR	MTA_Mm	chr16	62930284	62930677	-	0	0	0
LTR	MaLR	MTB_Mm	chr16	63202862	63203284	+	0	0	0
LINE	L1	L1Md_T	chr16	63257508	63263145	-	0	0	0
Simple_repeat	Simple_repeat	(A)n	chr16	63903888	63903938	+	0	0	0
SINE	Alu	B1_Mus1	chr10	80638365	80638506	-	0	0	0
LTR	MaLR	MTA_Mm	chr16	64283878	64284273	+	0	0	0
SINE	Alu	B1_Mus2	chr16	64846771	64846917	+	0	0	0
Simple_repeat	Simple_repeat	(A)n	chr16	64846918	64846942	+	0	0	0
SINE	Alu	B1F2	chr10	80719791	80719921	-	0	0	0
LINE	L1	L1Md_T	chr16	65745404	65751850	-	0	0	0
SINE	Alu	B1_Mus2	chr16	66045842	66045992	-	0	0	0
SINE	Alu	B1_Mur2	chr16	66046004	66046142	-	0	0	0
Simple_repeat	Simple_repeat	(A)n	chr16	66229406	66229445	+	0	0	0
Simple_repeat	Simple_repeat	(A)n	chr16	66280129	66280154	+	0	0	0
LINE	L1	L1Md_T	chr16	66518330	66523846	-	0.332194	1.08938	0.342623
SINE	B2	B2_Mm1a	chr16	66523847	66524033	-	0.332194	0.380568	0.342623
Simple_repeat	Simple_repeat	(A)n	chr16	67629545	67629581	+	0	0	0
Simple_repeat	Simple_repeat	(A)n	chr16	67652636	67652698	+	0	0	0
SINE	Alu	B1_Mus1	chr16	68173995	68174140	-	0	0	0
SINE	Alu	B1_Mus2	chr16	68374729	68374874	-	0	0	0
Simple_repeat	Simple_repeat	(A)n	chr16	68510223	68510267	+	0	0	0
LINE	L1	L1Md_F3	chr16	68669512	68675713	-	0	0	0
Simple_repeat	Simple_repeat	(A)n	chr16	68777967	68778000	+	0	0.380568	0
Simple_repeat	Simple_repeat	(A)n	chr16	68957962	68958009	+	0	0	0
LTR	MaLR	MTA_Mm	chr16	69315600	69315995	-	0.591053	0.708814	0
SINE	Alu	B1_Mus1	chr16	69360343	69360461	+	0	0	0
Simple_repeat	Simple_repeat	(A)n	chr16	69360462	69360520	+	0	0.380568	0.615324
Simple_repeat	Simple_repeat	(A)n	chr16	69548646	69548673	+	0	0	0
Simple_repeat	Simple_repeat	(A)n	chr16	69600051	69600090	+	0	0	0
Simple_repeat	Simple_repeat	(A)n	chr16	70019154	70019208	+	0	0	0
Simple_repeat	Simple_repeat	(A)n	chr16	70165944	70165973	+	0	0	0
SINE	Alu	B1_Mus1	chr10	81153037	81153184	-	0	0	0
LINE	L1	L1Md_A	chr16	71147905	71154513	-	0	0	0
LTR	MaLR	MTA_Mm	chr10	81187355	81187750	-	0	0.380568	0
SINE	Alu	B1_Mm	chr10	81241637	81241786	+	0	0	0
LINE	L1	L1Md_F2	chr16	72012597	72018594	-	0	0	0
Simple_repeat	Simple_repeat	(A)n	chr10	81241787	81241822	+	0	0	0
SINE	Alu	B1_Mm	chr16	72114204	72114349	-	0	0	0
SINE	Alu	B1_Mus1	chr10	81312132	81312279	-	0	0	0
SINE	Alu	B1_Mus2	chr16	73255135	73255283	-	0	0	0
LTR	MaLR	MTA_Mm	chr10	81346447	81346842	-	0	0.380568	0
SINE	Alu	B1_Mm	chr16	73701525	73701670	+	0	0	0
SINE	Alu	B1_Mm	chr10	81400978	81401127	+	0	0	0
Simple_repeat	Simple_repeat	(A)n	chr10	81401128	81401169	+	0	0	0
SINE	Alu	B1_Mm	chr10	81464491	81464636	-	0	0	0
LTR	MaLR	MTB	chr16	75000744	75001127	+	0	0	0
LTR	MaLR	MTA_Mm	chr10	81479976	81480370	-	0	0	0.615324
SINE	Alu	B1_Mus2	chr16	75223637	75223782	-	1.84649	1.46995	1.23065
Simple_repeat	Simple_repeat	(A)n	chr16	75272037	75272075	+	0	0	0
Simple_repeat	Simple_repeat	(A)n	chr16	76054821	76054857	+	0	0	0

SINE	Alu	B1_Mus2	chr16	76278117	76278263	+	0	0	0
Simple_repeat	Simple_repeat	(A)n	chr16	76278264	76278303	+	0	0	0
Simple_repeat	Simple_repeat	(A)n	chr16	76692842	76692871	+	0	0	0.615324
Simple_repeat	Simple_repeat	(A)n	chr16	78197632	78197674	+	0	0	0
SINE	Alu	B1_Mus2	chr10	81743628	81743774	-	0	0	0
LTR	MaLR	MTA_Mm	chr16	78517945	78518342	+	0	0	0
Simple_repeat	Simple_repeat	(A)n	chr16	78651066	78651103	+	0	0	0
SINE	Alu	B1_Mus1	chr16	78749964	78750111	-	0	0	0
Simple_repeat	Simple_repeat	(A)n	chr16	79494828	79494875	+	0	0	0
LINE	L1	L1Md_F2	chr16	79510837	79516583	-	0	0	0
LTR	MaLR	MTA_Mm	chr16	79529312	79529716	-	0	0	0
Simple_repeat	Simple_repeat	(A)n	chr16	79741577	79741627	+	0	0	0
Simple_repeat	Simple_repeat	(A)n	chr16	80895679	80895704	+	0	0	0
LINE	L1	L1Md_T	chr16	81029627	81036581	-	0	0	0
Simple_repeat	Simple_repeat	(A)n	chr16	81180253	81180294	+	0	0	0
SINE	Alu	B1_Mm	chr16	82101837	82101979	+	0	0	0
Simple_repeat	Simple_repeat	(A)n	chr16	82101980	82102010	+	0	0	0
SINE	Alu	B1_Mm	chr16	83424062	83424208	-	0	0	0
LTR	MaLR	MTA_Mm	chr16	83493148	83493542	+	0	0	0
SINE	Alu	B1_Mus1	chr16	83657045	83657197	-	0	0	0
SINE	Alu	B1_Mm	chr16	83661650	83661796	-	0	0	0
LTR	MaLR	MTA_Mm	chr16	84085084	84085479	-	0	0	0
Simple_repeat	Simple_repeat	(A)n	chr16	84529775	84529801	+	0	0	0
Simple_repeat	Simple_repeat	(TTTA)n	chr10	82293547	82293582	+	0.591053	0	0
LTR	MaLR	MTA_Mm	chr16	84764878	84765271	+	0	0	0
SINE	B2	B2_Mm1a	chr16	84882440	84882631	-	0	0	0.342623
SINE	Alu	B1_Mus1	chr16	85214137	85214284	+	0	0	0
Low_complexity	Low_complexity	A-rich	chr16	85214297	85214404	+	0	0	0
Simple_repeat	Simple_repeat	(A)n	chr16	85804206	85804233	+	0	0	0
LTR	MaLR	MTA_Mm	chr16	85829364	85829758	-	0	0	0
SINE	Alu	B1_Mus2	chr16	86157329	86157475	+	0	0	0
Simple_repeat	Simple_repeat	(A)n	chr16	86157476	86157498	+	0	0	0
LINE	L1	L1_Mus4	chr10	11237726	11243407	-	0	0	0
Simple_repeat	Simple_repeat	(A)n	chr16	87213890	87213919	+	0	0	0
SINE	Alu	B1_Mm	chr16	87674561	87674707	-	0	0	0
SINE	Alu	B1_Mus2	chr16	87687401	87687545	+	0	0	0
Simple_repeat	Simple_repeat	(A)n	chr16	87687546	87687572	+	0	0	0
LINE	L1	L1Md_F2	chr16	88658617	88665017	-	0	0	0
LTR	MaLR	MTA_Mm	chr16	89232428	89232824	-	0	0	0
LTR	MaLR	MTA_Mm	chr16	89453924	89454318	-	0	0	0
Simple_repeat	Simple_repeat	(A)n	chr16	89542494	89542538	+	0	0	0
Simple_repeat	Simple_repeat	(A)n	chr16	89675110	89675136	+	0	0	0
Simple_repeat	Simple_repeat	(A)n	chr16	90504652	90504685	+	0.591053	0	0
LTR	MaLR	MTA_Mm	chr16	90668054	90668460	+	0	0	0.615324
Simple_repeat	Simple_repeat	(A)n	chr16	91097405	91097440	+	0	0	0
SINE	Alu	B1_Mus2	chr10	82801210	82801356	-	0	0	0
SINE	B2	B3	chr16	91311376	91311559	-	0	0	0
SINE	Alu	B1_Mus2	chr10	82818299	82818444	+	0	0	0
Simple_repeat	Simple_repeat	(A)n	chr10	82818445	82818481	+	0	0	0
Simple_repeat	Simple_repeat	(A)n	chr16	91978355	91978391	+	0	0	0
SINE	Alu	B1_Mus1	chr16	92572276	92572405	-	0	0	0
Simple_repeat	Simple_repeat	(A)n	chr10	11330238	11330281	+	1.58764	0.380568	1.84597
LINE	L1	L1Md_F3	chr16	93617191	93622227	+	0.923247	1.41763	0
LTR	MaLR	MTA_Mm	chr16	93673360	93673755	+	0	0	0
Simple_repeat	Simple_repeat	(A)n	chr16	94512622	94512656	+	0	0	0
LTR	MaLR	MTA_Mm	chr16	94573110	94573504	+	0	0	0
SINE	Alu	B1_Mm	chr16	95830797	95830937	-	0	0	0
SINE	Alu	B1_Mus1	chr16	96031795	96031942	+	0	0	0
Simple_repeat	Simple_repeat	(A)n	chr16	96031943	96031977	+	0	0	0
SINE	Alu	B1_Mus2	chr16	96172914	96173058	+	0	0	0
Simple_repeat	Simple_repeat	(A)n	chr16	96173059	96173084	+	0	0	0
SINE	Alu	B1_Mus2	chr16	96324471	96324617	-	0	0	0
Simple_repeat	Simple_repeat	(A)n	chr16	97422049	97422089	+	0	0	0
SINE	Alu	B1_Mus1	chr16	97484485	97484632	+	0	0	0
Simple_repeat	Simple_repeat	(A)n	chr16	97484633	97484660	+	0	0	0.342623
SINE	Alu	B1_Mus1	chr16	98274037	98274177	+	0	0	0
Simple_repeat	Simple_repeat	(A)n	chr16	98274178	98274199	+	0	0	0

SINE	Alu	B1_Mus2	chr17	3089203	3089349	-	0	0	0
LTR	MaLR	MTA_Mm	chr10	83520503	83520897	+	0	0	0
Simple_repeat	Simple_repeat	(A)n	chr17	4106198	4106234	+	0	0	0
SINE	Alu	B1_Mus2	chr17	4709991	4710136	+	0	0	0
Simple_repeat	Simple_repeat	(A)n	chr17	4710137	4710186	+	0	0.380568	0
Simple_repeat	Simple_repeat	(A)n	chr17	4962042	4962078	+	0	0	0
SINE	Alu	B1_Mm	chr17	4983240	4983380	+	0	0	0
Simple_repeat	Simple_repeat	(A)n	chr17	4983381	4983421	+	0	0	0
SINE	Alu	B1_Mus2	chr17	6354163	6354310	-	0.664388	0	0.342623
Simple_repeat	Simple_repeat	(A)n	chr17	6461288	6461326	+	0	0	0
SINE	Alu	B1_Mm	chr17	6494237	6494383	-	0	0	0
SINE	Alu	B1_Mus1	chr17	6539963	6540111	-	0	0	0
SINE	Alu	B1_Mus1	chr17	7049637	7049784	-	0	0	0
SINE	Alu	B1_Mus2	chr17	7640719	7640864	+	0	0	0
Simple_repeat	Simple_repeat	(A)n	chr17	7640865	7640887	+	0	0	0
SINE	Alu	B1_Mus2	chr17	7721554	7721699	+	0	0	0
Simple_repeat	Simple_repeat	(A)n	chr17	7721700	7721740	+	0	0	0
SINE	Alu	B1_Mus1	chr17	7884446	7884592	+	0	0	0
SINE	Alu	B1_Mus1	chr17	8351392	8351538	+	0	0	0
Simple_repeat	Simple_repeat	(A)n	chr17	8351539	8351569	+	0	0	0
Simple_repeat	Simple_repeat	(A)n	chr17	8944414	8944450	+	0	0	0
SINE	Alu	B1_Mm	chr17	10552820	10552966	-	0	0	0
SINE	Alu	B1_Mus2	chr17	12826580	12826708	-	0	0	0
LTR	MaLR	MTA_Mm	chr17	13504469	13504860	-	0	0	0
SINE	Alu	B1_Mus1	chr17	13750221	13750339	-	0	0	0
Simple_repeat	Simple_repeat	(A)n	chr17	14172212	14172241	+	0	0	0
SINE	Alu	B1_Mus2	chr17	15244781	15244927	+	0	0	0
Simple_repeat	Simple_repeat	(A)n	chr17	15244928	15244951	+	0	0	0
SINE	Alu	B1_Mus2	chr17	15277236	15277380	+	0	0	0
Simple_repeat	Simple_repeat	(A)n	chr17	15277381	15277420	+	0	0	0.342623
LTR	MaLR	MTA_Mm	chr17	16124234	16124628	+	0	0	0
LTR	MaLR	MTA_Mm	chr17	16246961	16247357	-	0	0	0
Simple_repeat	Simple_repeat	(A)n	chr17	16380048	16380082	+	0	0	0
Simple_repeat	Simple_repeat	(A)n	chr17	16754679	16754706	+	0	0	0
SINE	Alu	B1_Mus2	chr17	17086791	17086937	-	0	0	0
SINE	Alu	B1_Mus2	chr17	17483557	17483700	+	0	0	0.615324
LTR	MaLR	MTB	chr17	17493623	17494023	-	0	0	0
Simple_repeat	Simple_repeat	(A)n	chr17	17504220	17504244	+	1.18211	0	0
SINE	B2	B2_Mm2	chr17	17616062	17616250	-	0	0	0
SINE	Alu	B1_Mm	chr17	18037257	18037401	+	0	0	0
Simple_repeat	Simple_repeat	(A)n	chr17	18037402	18037431	+	0	0	0
LTR	MaLR	MTA_Mm	chr17	18334696	18335084	-	0	0	0
SINE	Alu	B1_Mus1	chr17	18468903	18469052	+	0	0	0
Low_complexity	Low_complexity	A-rich	chr17	18469053	18469158	+	0	0	0.342623
LINE	L1	L1Md_F2	chr17	18482792	18488033	+	0	0	0
SINE	Alu	B1_Mm	chr17	18505707	18505853	-	0	0	0
SINE	Alu	B1_Mus1	chr17	18535278	18535425	-	0	0	0
LTR	MaLR	MTA_Mm	chr17	18780391	18780769	-	0	0	0
Low_complexity	Low_complexity	A-rich	chr17	19669523	19669690	+	0	0	0
Simple_repeat	Simple_repeat	(A)n	chr17	20054632	20054687	+	0	0	0
LINE	L1	L1Md_F2	chr17	20424258	20430476	-	0	0	0
SINE	Alu	B1_Mus2	chr17	20574865	20575010	-	0	0	0
SINE	Alu	B1_Mm	chr17	20627771	20627917	+	0	0	0
Simple_repeat	Simple_repeat	(A)n	chr17	20627918	20627942	+	0.332194	0	0
Simple_repeat	Simple_repeat	(A)n	chr17	20782608	20782652	+	0	0	0
LINE	L1	L1_Mus2	chr17	21016581	21022547	+	0	0	0
LTR	MaLR	MTA_Mm	chr17	21037184	21037577	-	0	0	0
Simple_repeat	Simple_repeat	(A)n	chr17	21108397	21108446	+	0.923247	1.08938	0.685246
LTR	MaLR	MTA_Mm	chr17	21223768	21224159	-	0	0	0
LTR	MaLR	MTA_Mm	chr17	21374049	21374442	+	0	0	0
SINE	Alu	B1_Mm	chr17	21725912	21726022	+	0	0	0
Simple_repeat	Simple_repeat	(A)n	chr17	21726023	21726047	+	0	0	0
SINE	Alu	B1_Mus1	chr17	21812801	21812945	+	0	0	0
Simple_repeat	Simple_repeat	(A)n	chr17	21983619	21983667	+	0	0	0
SINE	Alu	B1_Mm	chr17	22308708	22308854	+	0	0	0
SINE	Alu	B1_Mus1	chr17	22793320	22793464	+	0	0	0
Simple_repeat	Simple_repeat	(A)n	chr17	22793465	22793511	+	0	0	0

Simple_repeat	Simple_repeat	(A)n	chr17	23668583	23668617	+	0	0	0
Simple_repeat	Simple_repeat	(A)n	chr17	23782854	23782897	+	0	0	0
Simple_repeat	Simple_repeat	(A)n	chr17	23837684	23837713	+	0.591053	0	1.23065
SINE	Alu	B1_Mm	chr17	24395732	24395875	+	0	0	0
Simple_repeat	Simple_repeat	(A)n	chr17	24395876	24395902	+	0	0	0
SINE	Alu	B1_Mus2	chr10	85394623	85394769	+	0	0	0
Simple_repeat	Simple_repeat	(A)n	chr10	85394770	85394797	+	0	0	0
Simple_repeat	Simple_repeat	(A)n	chr17	25848550	25848590	+	0	0	0
SINE	Alu	B1_Mus1	chr17	26108157	26108304	-	0	0	0
SINE	Alu	B1_Mus2	chr17	26270494	26270632	-	0	0	0
LTR	MaLR	MTA_Mm	chr17	26385266	26385660	+	0	0	0
Simple_repeat	Simple_repeat	(A)n	chr17	26697303	26697341	+	0	0	0
LTR	MaLR	MTA_Mm	chr17	26943872	26944267	+	0	0	0
SINE	Alu	B1_Mus1	chr17	27040084	27040224	-	0	0	0
SINE	Alu	B1_Mus1	chr17	27114557	27114705	+	1.77316	0	0.615324
SINE	Alu	B1_Mus1	chr17	27121671	27121818	+	2.82479	1.08938	0.615324
Low_complexity	Low_complexity	A-rich	chr17	27121819	27121875	+	1.18211	1.08938	0
Simple_repeat	Simple_repeat	(TTTA)n	chr17	27974637	27974660	+	0	0	0
SINE	B2	B2_Mm1t	chr17	28527787	28527978	-	1.16015	0	0
SINE	Alu	B1_Mus1	chr17	28528020	28528167	-	0	0	0
Simple_repeat	Simple_repeat	(A)n	chr17	28648978	28649014	+	0	0	0
SINE	Alu	B1_Mm	chr17	28678465	28678613	+	0	0	0
Simple_repeat	Simple_repeat	(A)n	chr17	28678614	28678643	+	0.664388	0	0
Simple_repeat	Simple_repeat	(A)n	chr17	28700253	28700289	+	0	0	0
Simple_repeat	Simple_repeat	(A)n	chr17	28726294	28726380	+	0	0	0
SINE	Alu	B1_Mus1	chr17	29055143	29055288	+	0	0	0
LTR	ERV1	MURVY-LTR	chr17	29055289	29055720	+	0	0	0
SINE	Alu	B1_Mm	chr17	29261151	29261296	+	0	0	0
Simple_repeat	Simple_repeat	(A)n	chr17	29261297	29261332	+	0	0	0
SINE	Alu	B1_Mus2	chr17	29402688	29402836	-	0	0	0
SINE	Alu	B1_Mus1	chr17	29404263	29404410	-	0	0	0
SINE	Alu	B1_Mus1	chr17	29454208	29454353	+	0	0	0
Simple_repeat	Simple_repeat	(A)n	chr17	29454354	29454385	+	0	0	0
SINE	Alu	B1_Mm	chr17	29591028	29591173	+	0	0	0
Simple_repeat	Simple_repeat	(A)n	chr17	29591174	29591196	+	0	0	0
Simple_repeat	Simple_repeat	(A)n	chr17	29622061	29622089	+	0	0	0
SINE	Alu	B1_Mus2	chr17	29643593	29643739	-	0	0	0
SINE	Alu	B1_Mus1	chr17	30124209	30124354	+	0	0	0
Simple_repeat	Simple_repeat	(A)n	chr17	30124355	30124377	+	0	0	0.342623
SINE	Alu	B1_Mus2	chr17	30137774	30137919	+	0	0	0
Simple_repeat	Simple_repeat	(A)n	chr17	30137920	30137956	+	0	0	0
SINE	Alu	B1_Mur1	chr17	30138187	30138324	-	0	0	0
LTR	ERVK	RMER19B	chr17	31149283	31150195	+	0	0	0
SINE	Alu	B1_Mm	chr10	11730916	11731053	+	0	0	0
Simple_repeat	Simple_repeat	(A)n	chr17	31866499	31866539	+	0	0	0
Simple_repeat	Simple_repeat	(A)n	chr10	11731054	11731110	+	0	0	0
SINE	Alu	B1_Mm	chr17	32544965	32545111	-	0	0	0
Simple_repeat	Simple_repeat	(A)n	chr17	32546465	32546502	+	0	0	0
Simple_repeat	Simple_repeat	(A)n	chr17	32670469	32670496	+	0	0	0
SINE	Alu	B1_Mus2	chr17	33174369	33174515	+	0	0	0
LTR	MaLR	MTA_Mm	chr17	33394123	33394503	+	0	0	0
LTR	MaLR	MTA_Mm	chr17	33424071	33424451	-	0	0	0
SINE	Alu	B1_Mus1	chr17	34635113	34635257	+	0	0	0
Simple_repeat	Simple_repeat	(CAAAA)n	chr17	34635260	34635325	+	0	0	0
SINE	Alu	B1_Mus2	chr17	34916715	34916861	+	0	0	0
Simple_repeat	Simple_repeat	(A)n	chr17	34916862	34916886	+	0	0	0
SINE	Alu	B1_Mus1	chr17	35081062	35081207	-	0	0	0
SINE	Alu	B1_Mus2	chr17	35199156	35199302	-	0	0	0
Simple_repeat	Simple_repeat	(A)n	chr10	3918711	3918751	+	0	0	0
SINE	Alu	B1_Mus1	chr17	35342731	35342879	-	0	0	0
Simple_repeat	Simple_repeat	(A)n	chr17	35514972	35515005	+	0	0	0
SINE	Alu	B1_Mus2	chr17	35635555	35635699	+	0	0	0
Simple_repeat	Simple_repeat	(A)n	chr17	35635700	35635725	+	0	0	0
Simple_repeat	Simple_repeat	(A)n	chr10	86749472	86749502	+	0	0	0
SINE	Alu	B1_Mus1	chr17	35750417	35750555	-	0	0	0
LTR	MaLR	MTA_Mm	chr17	35852525	35852914	+	0	0.761137	0
SINE	Alu	B1_Mus1	chr17	35951332	35951466	+	0	0	0

Simple_repeat	Simple_repeat	(A)n	chr17	35951467	35951512	+	0	0	0
LINE	L1	L1Md_F2	chr17	36880706	36886565	+	0	0	0
SINE	Alu	B1_Mm	chr17	37108817	37108962	+	0	0	0
Simple_repeat	Simple_repeat	(A)n	chr17	37108963	37109018	+	0	0	0
SINE	Alu	B1_Mm	chr17	37163046	37163192	+	0	0	0
Simple_repeat	Simple_repeat	(A)n	chr17	37163193	37163219	+	0	0	0
Simple_repeat	Simple_repeat	(A)n	chr17	37300596	37300623	+	0	0	0
Simple_repeat	Simple_repeat	(A)n	chr17	37503935	37503982	+	0	0	0.615324
Simple_repeat	Simple_repeat	(A)n	chr17	37554022	37554056	+	0	0	0
SINE	Alu	B1_Mus1	chr17	37556619	37556766	+	0	0	0
LTR	MaLR	MTA_Mm	chr17	38123301	38123684	-	0	0	0
SINE	Alu	B1_Mus2	chr17	38135577	38135721	-	0	0	0
SINE	Alu	B1_Mm	chr17	38643454	38643596	+	0	0	0
Simple_repeat	Simple_repeat	(A)n	chr10	11826371	11826393	+	0	0	0
SINE	Alu	B1_Mus1	chr10	3921946	3922090	+	0	0	0
Simple_repeat	Simple_repeat	(A)n	chr10	3922091	3922120	+	0	0	0
LINE	L1	L1_Mus1	chr17	40150250	40156284	-	0	0.380568	0
SINE	Alu	B1_Mus1	chr10	87249970	87250083	+	0	0	0
Simple_repeat	Simple_repeat	(A)n	chr10	87250084	87250125	+	0	0	0
Simple_repeat	Simple_repeat	(A)n	chr17	40610338	40610415	+	0	0	0
Simple_repeat	Simple_repeat	(A)n	chr17	41387614	41387659	+	0	0	0.615324
LTR	MaLR	MTA_Mm	chr17	41511802	41512203	+	0	0	0
LINE	L1	L1Md_F2	chr17	41521576	41527076	-	0	0	0
LINE	L1	L1Md_F3	chr17	41546557	41551765	+	0	0	0
SINE	Alu	B1_Mus1	chr17	41696276	41696422	+	0	0	0
Simple_repeat	Simple_repeat	(A)n	chr17	42035332	42035362	+	0	0	0
Simple_repeat	Simple_repeat	(A)n	chr17	42277593	42277623	+	0	0	0
Simple_repeat	Simple_repeat	(A)n	chr17	42289867	42289895	+	0	0	0
LTR	MaLR	MTA_Mm	chr17	42397957	42398350	-	0	0	0
SINE	Alu	B1_Mm	chr17	42920252	42920398	-	0	0	0
SINE	Alu	B1_Mus2	chr17	43044631	43044776	-	0	0	0
LTR	MaLR	MTA_Mm	chr17	45343679	45344060	-	0	0	0
SINE	Alu	B1_Mus1	chr17	45623493	45623604	+	0	0	0
Simple_repeat	Simple_repeat	(GAAA)n	chr17	45623605	45623746	+	0	0	0
SINE	Alu	B1_Mus1	chr17	45713187	45713315	-	0	0	0
SINE	Alu	B1_Mus2	chr17	46498660	46498808	-	0	0	0
SINE	Alu	B1_Mus2	chr17	46999538	46999683	-	2.10535	1.08938	0
SINE	Alu	B1_Mus1	chr17	47243828	47243975	-	0	0	0
Simple_repeat	Simple_repeat	(A)n	chr10	87976211	87976238	+	0	0	0
Simple_repeat	Simple_repeat	(A)n	chr10	88071859	88071897	+	0	0	0
Simple_repeat	Simple_repeat	(A)n	chr17	48243890	48243923	+	0	0	0
SINE	Alu	B1_Mus1	chr17	48475636	48475781	+	0	0	0
Simple_repeat	Simple_repeat	(A)n	chr17	48475782	48475808	+	0	0	0
SINE	Alu	B1_Mm	chr10	88123731	88123877	-	0	0	0
SINE	Alu	B1_Mm	chr10	88128170	88128315	+	0	0	0
Simple_repeat	Simple_repeat	(A)n	chr10	88128316	88128335	+	0	0	0
SINE	Alu	B1_Mus2	chr17	49383362	49383508	-	0	0	0
Simple_repeat	Simple_repeat	(A)n	chr17	49423217	49423258	+	0	0	0
Simple_repeat	Simple_repeat	(A)n	chr17	49423706	49423742	+	0	0	0
SINE	Alu	B1_Mus1	chr17	51380185	51380330	-	0	0	0
Simple_repeat	Simple_repeat	(A)n	chr17	51477181	51477208	+	0	0	0
LTR	MaLR	MTA_Mm	chr17	51778371	51778766	-	0	0	0
LTR	MaLR	MTA_Mm	chr17	51779865	51780260	-	0	0	0
LTR	MaLR	MTA_Mm	chr17	51990508	51990903	+	0.332194	2.12644	0.615324
SINE	Alu	B1_Mus2	chr17	52003306	52003452	+	3.10194	3.97696	3.21646
Simple_repeat	Simple_repeat	(A)n	chr17	52183347	52183379	+	0	0	0
SINE	Alu	B1_Mm	chr17	52188898	52189044	-	0	0	0
SINE	Alu	B1_Mus2	chr17	53693077	53693223	+	0	0	0
Simple_repeat	Simple_repeat	(A)n	chr17	53693224	53693247	+	0	0	0
Simple_repeat	Simple_repeat	(A)n	chr17	53700207	53700276	+	0.332194	0	0
SINE	Alu	B1_Mm	chr17	54171011	54171157	+	0	0	0
Simple_repeat	Simple_repeat	(A)n	chr17	54171158	54171183	+	0	0	0
LINE	L1	L1_Mus4	chr17	54901791	54906250	-	0.332194	0	0
LTR	MaLR	MTA_Mm	chr17	54950684	54951077	+	0	0	0
LINE	L1	Lx	chr17	55722172	55727669	-	0	0	0
SINE	Alu	B1_Mus2	chr17	56364767	56364913	+	0	0	0
Simple_repeat	Simple_repeat	(A)n	chr17	56364914	56364949	+	0	0	0

SINE	Alu	B1_Mus2	chr17	56391296	56391441	-	0	0	0
SINE	Alu	B1_Mm	chr17	56463499	56463646	+	0	0	0
Simple_repeat	Simple_repeat	(A)n	chr17	56463647	56463674	+	0	0	0
SINE	Alu	B1_Mus1	chr17	56662694	56662840	+	0	0	0
Low_complexity	Low_complexity	A-rich	chr17	56662841	56662896	+	0	0	0
SINE	B2	B2_Mm2	chr17	56689079	56689272	-	0	0	0
SINE	Alu	B1_Mm	chr17	56865259	56865405	+	0	0	0
Simple_repeat	Simple_repeat	(A)n	chr17	56865406	56865428	+	0	0	0
Simple_repeat	Simple_repeat	(A)n	chr17	56973859	56973891	+	0	0	0
Simple_repeat	Simple_repeat	(A)n	chr17	57141771	57141792	+	0	0	0
LINE	L1	L1Md_F2	chr10	12050073	12055123	+	0	0	0
SINE	Alu	B1_Mus1	chr17	57269747	57269894	+	0	0	0
Simple_repeat	Simple_repeat	(A)n	chr17	57269895	57269929	+	0	0	0
SINE	Alu	B1_Mus1	chr10	89000301	89000443	-	0	0	0
LTR	MaLR	MTA_Mm	chr17	58933115	58933510	+	0.591053	0	0.615324
LTR	MaLR	MTA_Mm	chr10	89167052	89167447	+	0	0	0
SINE	Alu	B1_Mus2	chr17	60523656	60523804	-	0	0	0
SINE	Alu	B1_Mm	chr17	60548977	60549122	+	0	0	0
SINE	Alu	B1_Mm	chr10	12098767	12098911	-	0	0	0
Low_complexity	Low_complexity	A-rich	chr17	61723210	61723271	+	0	0	0
SINE	Alu	B1_Mm	chr17	61801670	61801808	+	0	0	0
Simple_repeat	Simple_repeat	(A)n	chr17	61801809	61801831	+	0	0	0
SINE	Alu	B1_Mus2	chr17	62357571	62357718	-	0	0	0
Simple_repeat	Simple_repeat	(A)n	chr17	62863329	62863368	+	0	0	0
SINE	Alu	B1_Mus2	chr17	63896669	63896815	-	0	0	0
SINE	Alu	B1_Mm	chr17	63997038	63997173	-	0	0	0
SINE	Alu	B1_Mm	chr17	64083145	64083291	+	0	0	0
Simple_repeat	Simple_repeat	(A)n	chr17	64083292	64083315	+	0	0	0
SINE	Alu	B1_Mus2	chr17	64615435	64615581	+	0	0	0
Simple_repeat	Simple_repeat	(A)n	chr17	64615582	64615607	+	0	0	0
SINE	Alu	B1_Mus1	chr17	64946205	64946352	+	0	0	0
Simple_repeat	Simple_repeat	(A)n	chr17	64946353	64946377	+	0	0	0
Simple_repeat	Simple_repeat	(A)n	chr17	65226967	65227005	+	0	0	0
SINE	B2	B2_Mm1a	chr17	66518990	66519175	+	0	0	0
SINE	Alu	B1_Mm	chr17	66809630	66809777	-	0	0	0
SINE	Alu	B1_Mus2	chr17	66831332	66831478	-	0	0	0
SINE	Alu	B1_Mm	chr17	67735328	67735473	-	0	0	0
Simple_repeat	Simple_repeat	(A)n	chr17	68526394	68526432	+	0	0	0
LTR	MaLR	MTA_Mm	chr17	69318725	69319121	-	0	0	0
Simple_repeat	Simple_repeat	(A)n	chr17	69919837	69919863	+	3.46721	2.98884	0
SINE	Alu	B1_Mm	chr17	70279777	70279923	+	0	0	0
Simple_repeat	Simple_repeat	(A)n	chr17	70506604	70506668	+	1.58764	1.41763	1.30057
SINE	Alu	B1_Mus2	chr17	71190591	71190737	+	0	0	0
Simple_repeat	Simple_repeat	(A)n	chr17	71190738	71190761	+	0	0	0
SINE	Alu	B1_Mur2	chr17	71191754	71191861	+	0	0	0
Simple_repeat	Simple_repeat	(A)n	chr17	71191862	71191887	+	0	0	0
SINE	Alu	B1_Mus2	chr17	71220491	71220627	-	0	0.380568	0
LTR	MaLR	MTA_Mm	chr17	74084609	74085004	+	0	0	0
Simple_repeat	Simple_repeat	(TTTA)n	chr10	90448338	90448358	+	0	0	0
Simple_repeat	Simple_repeat	(A)n	chr17	74484343	74484381	+	0	0	0
SINE	Alu	B1_Mus2	chr17	74724108	74724254	+	0	0	0
Simple_repeat	Simple_repeat	(A)n	chr17	74724255	74724293	+	0	0	0
Simple_repeat	Simple_repeat	(A)n	chr17	74734769	74734797	+	0	0	0
Low_complexity	Low_complexity	GA-rich	chr17	74885304	74885388	+	0	0	0
SINE	Alu	B1_Mus2	chr17	74906920	74907065	+	0	0	0
Simple_repeat	Simple_repeat	(A)n	chr17	74907066	74907107	+	0	0	0.342623
Simple_repeat	Simple_repeat	(A)n	chr17	75104467	75104511	+	1.5143	0.708814	2.06302
SINE	Alu	B1_Mus2	chr17	75264868	75264998	-	0	0	0
SINE	Alu	B1_Mm	chr17	76206011	76206151	-	0	0	0
LTR	MaLR	MTA_Mm	chr17	76759659	76760050	+	0	0	0
SINE	B4	B4	chr17	77334637	77334923	+	0	0	0
LINE	L1	L1Md_F3	chr17	78097627	78102811	+	0	0	0
Simple_repeat	Simple_repeat	(A)n	chr17	78257725	78257765	+	0	0	0
SINE	Alu	B1_Mus1	chr10	90837604	90837739	+	0	0	0
SINE	Alu	B1_Mus2	chr17	80314386	80314529	+	0	0	0
SINE	Alu	B1_Mur3	chr17	80512062	80512181	-	0	0	0
SINE	Alu	B1_Mus2	chr17	81133328	81133457	-	0	0	0

Simple_repeat	Simple_repeat	(A)n	chr17	81247064	81247098	+	0	0	0
SINE	Alu	B1_Mm	chr17	81693260	81693406	+	0	0	0
LTR	MaLR	MTA_Mm	chr17	82788378	82788774	-	0	0	0
LTR	MaLR	MTA_Mm	chr17	82804240	82804633	+	0.332194	0	0
Simple_repeat	Simple_repeat	(A)n	chr17	82870098	82870141	+	0	0	0
SINE	Alu	B1_Mus1	chr17	83632960	83633080	+	0	0	0
Simple_repeat	Simple_repeat	(A)n	chr17	83633081	83633131	+	0	0	0
SINE	Alu	B1_Mm	chr17	83934920	83935068	-	0	0	0
Simple_repeat	Simple_repeat	(A)n	chr17	84081195	84081232	+	0	0	0
LTR	MaLR	MTB	chr17	84084115	84084505	+	0	0	0
SINE	Alu	B1_Mm	chr17	84100570	84100715	+	0	0	0
Simple_repeat	Simple_repeat	(A)n	chr17	84100716	84100764	+	0	0	0
Simple_repeat	Simple_repeat	(A)n	chr10	91316842	91316871	+	0	0	0.615324
LTR	MaLR	MTA_Mm	chr17	84567201	84567597	+	0	0	0
Simple_repeat	Simple_repeat	(A)n	chr17	84582280	84582311	+	0	0	0
SINE	Alu	B1_Mus1	chr10	91427990	91428134	-	0	0	0
SINE	Alu	B1_Mus1	chr17	85099860	85100008	-	0	0	0
Simple_repeat	Simple_repeat	(A)n	chr10	91539514	91539540	+	0	0	0
Simple_repeat	Simple_repeat	(A)n	chr17	85929415	85929441	+	0	0	0
Simple_repeat	Simple_repeat	(A)n	chr17	87328727	87328752	+	0	0	0
SINE	Alu	B1_Mus1	chr17	88015348	88015494	-	0	0	0
SINE	Alu	B1_Mus2	chr17	88061572	88061717	-	0	0	0.342623
SINE	Alu	B1_Mus2	chr17	88358722	88358868	-	0	0	0
SINE	Alu	B1_Mus1	chr17	88363793	88363925	-	0	0	0
SINE	Alu	B1_Mus2	chr17	88478736	88478885	-	0	0	0
LTR	MaLR	MTA_Mm	chr17	88501436	88501831	+	0	0	0
SINE	Alu	B1_Mus1	chr17	88524210	88524343	-	0	0	0
SINE	Alu	B1_Mus2	chr17	88648903	88649049	-	0	0	0
SINE	Alu	B1_Mus1	chr17	88838885	88839033	-	0	0	0
LINE	L1	Lx3B	chr17	89631799	89636902	-	0	0	0
Simple_repeat	Simple_repeat	(A)n	chr17	91987262	91987306	+	0.332194	0	0
SINE	B2	B2_Mm1a	chr17	92711419	92711608	+	0	0	0
Simple_repeat	Simple_repeat	(A)n	chr17	92726095	92726141	+	0	0	0
LINE	L1	L1Md_T	chr17	93095180	93101626	-	0	0	0
SINE	Alu	B1_Mus2	chr10	92389892	92390008	-	0	0	0
Simple_repeat	Simple_repeat	(A)n	chr17	93923399	93923427	+	0	0	0
LINE	L1	L1Md_T	chr17	94022864	94029305	-	0	0	0
LTR	MaLR	MTA_Mm	chr17	94115791	94116179	-	0	0	0
LTR	MaLR	MTB	chr17	94144431	94144815	-	0	0	0
LTR	MaLR	MTA_Mm	chr17	94319055	94319448	+	0	0	0
Simple_repeat	Simple_repeat	(A)n	chr17	94484465	94484501	+	0	0	0
SINE	Alu	B1_Mus1	chr17	95268817	95268962	-	0	0	0
SINE	Alu	B1_Mm	chr18	3089757	3089876	-	0	0	0
LTR	MaLR	MTA_Mm	chr18	3568208	3568602	+	0	0	0
LTR	MaLR	MTA_Mm	chr18	3569700	3570094	+	0.591053	0	0
SINE	Alu	B1_Mus2	chr18	4477735	4477881	+	0	0	0
Simple_repeat	Simple_repeat	(A)n	chr18	4500541	4500571	+	0	0.380568	0
Simple_repeat	Simple_repeat	(A)n	chr18	4981768	4981812	+	0	0	0
SINE	Alu	B1_Mm	chr18	5426557	5426702	-	0	0	0
LTR	MaLR	MTA_Mm	chr18	5804371	5804764	+	0	0	0
SINE	Alu	B1_Mus2	chr18	5822532	5822678	-	0	0	0
LTR	ERV1	MuRRS-int	chr18	6546344	6551000	+	0	0	0
LTR	MaLR	MTA_Mm	chr18	7770449	7770841	-	0	0	0
SINE	Alu	B1_Mus2	chr18	7843342	7843488	+	0	0.380568	0
SINE	Alu	B1_Mm	chr18	7856267	7856410	-	0	0	0
Simple_repeat	Simple_repeat	(A)n	chr18	7936239	7936262	+	0	0	0
LTR	MaLR	MTA_Mm	chr18	8381589	8381983	-	0	0	0
SINE	Alu	B1_Mm	chr18	8608302	8608454	+	0	0	0
Simple_repeat	Simple_repeat	(A)n	chr18	8608455	8608477	+	0	0	0
Simple_repeat	Simple_repeat	(A)n	chr18	8697113	8697158	+	0.591053	1.41763	0.615324
LINE	L1	L1VL2	chr18	8798966	8803655	-	0	0	0
Simple_repeat	Simple_repeat	(A)n	chr18	9003743	9003787	+	0	0	0
LTR	MaLR	MTA_Mm	chr18	9020935	9021330	-	0	0	0
LTR	MaLR	MTB	chr18	9048732	9049127	+	0	0	0
SINE	Alu	B1_Mus2	chr18	9502658	9502804	+	0	0	0
Simple_repeat	Simple_repeat	(A)n	chr18	9502805	9502859	+	0	0	0
Simple_repeat	Simple_repeat	(A)n	chr18	9573108	9573156	+	0	0	0

Simple_repeat	Simple_repeat	(A)n	chr18	10052261	10052284	+	0	0	0
SINE	Alu	B1_Mus2	chr18	10263444	10263586	-	0	0	0
Simple_repeat	Simple_repeat	(GAAA)n	chr18	10615678	10615736	+	0	0	0
SINE	Alu	B1_Mm	chr18	10861769	10861914	+	0	0	0
Simple_repeat	Simple_repeat	(A)n	chr18	10861915	10861938	+	0	0	0
LTR	MaLR	MTA_Mm	chr18	10898002	10898397	-	0	0	0
LINE	L1	L1Md_F2	chr18	11337701	11344102	-	0	0	0
LTR	MaLR	MTA_Mm	chr18	11650337	11650730	-	1.05163	0	0
Simple_repeat	Simple_repeat	(A)n	chr18	11776783	11776804	+	0	0	0
SINE	B2	B2_Mm2	chr10	93283926	93284105	+	0	0	0
LTR	MaLR	MTA_Mm	chr18	12481354	12481748	+	0	0	0
SINE	Alu	B1_Mus2	chr18	13728873	13728994	-	0	0	0
Simple_repeat	Simple_repeat	(A)n	chr18	14482562	14482598	+	0	0	0
SINE	Alu	B1_Mus2	chr18	15479206	15479351	-	0	0	0
LTR	MaLR	MTA_Mm	chr18	15494204	15494598	-	0	0	0
SINE	B4	B4	chr18	16514649	16514897	-	0	0	0
Simple_repeat	Simple_repeat	(T)n	chr18	16606319	16606343	+	0	0	0
LTR	MaLR	MTA_Mm	chr18	16674718	16675112	+	0	0	0
LINE	L1	L1Md_T	chr18	17979385	17986064	-	0	0	0
SINE	Alu	B1_Mm	chr18	18388935	18389081	-	0	0	0
SINE	Alu	B1_Mm	chr18	18723522	18723667	+	0	0	0
Simple_repeat	Simple_repeat	(A)n	chr18	18997476	18997501	+	0	0	0
SINE	Alu	B1_Mus2	chr18	19552050	19552196	-	0	0	0
SINE	Alu	B1_Mm	chr18	19613017	19613163	-	0	0	0
Simple_repeat	Simple_repeat	(A)n	chr18	19983848	19983885	+	0	0	0
Simple_repeat	Simple_repeat	(A)n	chr18	20655836	20655873	+	0	0	0
LINE	L1	MusHAL1	chr18	20662336	20664536	+	0	0	0
Simple_repeat	Simple_repeat	(A)n	chr18	21081785	21081823	+	0	0	0
SINE	Alu	B1_Mm	chr18	21091495	21091618	+	0	0	0
Simple_repeat	Simple_repeat	(A)n	chr18	21091619	21091646	+	0	0	0
SINE	Alu	B1_Mus2	chr18	21203685	21203831	+	0	0	0
Simple_repeat	Simple_repeat	(A)n	chr18	21203832	21203869	+	0	0	0
SINE	Alu	B1_Mus2	chr18	21274250	21274396	+	0	0	0
Simple_repeat	Simple_repeat	(A)n	chr18	21274397	21274430	+	0	0	0
Simple_repeat	Simple_repeat	(A)n	chr18	21643063	21643094	+	0	0	0
Simple_repeat	Simple_repeat	(A)n	chr18	21907660	21907688	+	0	0	0
LINE	L1	L1_Mus3	chr18	22337358	22342617	+	0	0	0
Simple_repeat	Simple_repeat	(A)n	chr18	22455250	22455297	+	0	0	0
SINE	Alu	B1_Mur4	chr10	94079442	94079572	+	0	0	0
Low_complexity	Low_complexity	GA-rich	chr10	94079573	94079657	+	0	0	0
SINE	B2	B2_Mm1a	chr10	94105709	94105901	-	0	0	0
Simple_repeat	Simple_repeat	(A)n	chr18	24074915	24074990	+	0.332194	0	0
SINE	Alu	B1_Mus1	chr18	24281169	24281301	-	0.591053	0	0
SINE	Alu	B1_Mus2	chr18	24354239	24354385	-	0	0	0
SINE	Alu	B1_Mur2	chr18	24462428	24462575	+	0	1.08938	0
Simple_repeat	Simple_repeat	(A)n	chr18	24462576	24462620	+	0	1.08938	0
Simple_repeat	Simple_repeat	(A)n	chr18	24592020	24592083	+	0	0	0
Simple_repeat	Simple_repeat	(A)n	chr18	24597607	24597646	+	0	0	0
LTR	MaLR	MTA_Mm	chr18	25972017	25972411	-	0	0	0
LTR	MaLR	MTA_Mm	chr18	25973506	25973900	-	0	0	0
LTR	MaLR	MTA_Mm	chr18	26707258	26707652	-	0	0	0
LTR	MaLR	MTA_Mm	chr18	26844096	26844478	+	0.591053	1.01984	0
LTR	MaLR	MTA_Mm	chr18	27274716	27275095	-	0	0	0
Simple_repeat	Simple_repeat	(A)n	chr18	27607534	27607565	+	0	0	0
Simple_repeat	Simple_repeat	(A)n	chr18	27807682	27807719	+	0	0	0
Simple_repeat	Simple_repeat	(A)n	chr18	29241465	29241514	+	0	0	0
LTR	MaLR	MTA_Mm	chr18	29271975	29272368	-	0	0	0
SINE	Alu	B1_Mus1	chr18	29322149	29322296	-	0	0	0
Simple_repeat	Simple_repeat	(A)n	chr18	29461790	29461835	+	0	0	0
SINE	Alu	B1_Mus2	chr18	29777561	29777707	+	0	0	0
Simple_repeat	Simple_repeat	(A)n	chr18	29777708	29777745	+	0	0	0
LTR	MaLR	MTA_Mm	chr18	29948978	29949373	-	0	0	0
Simple_repeat	Simple_repeat	(A)n	chr18	30653631	30653665	+	0	0	0
Simple_repeat	Simple_repeat	(A)n	chr18	30766618	30766652	+	0	0	0
LTR	MaLR	MTA_Mm	chr18	31065538	31065932	-	0	0	0
Simple_repeat	Simple_repeat	(A)n	chr18	31107065	31107090	+	0	0	0
SINE	Alu	B1_Mus2	chr18	32457135	32457279	-	0	0	0

Simple_repeat	Simple_repeat	(A)n	chr18	32742638	32742680	+	0	0	0
SINE	Alu	B1_Mus1	chr18	33052291	33052437	-	0	0	0
LTR	MaLR	MTA_Mm	chr18	33548373	33548740	-	0	0	0
Simple_repeat	Simple_repeat	(A)n	chr18	33715092	33715132	+	0	0	0
LTR	MaLR	MTA_Mm	chr18	33738065	33738459	-	0	0	0
SINE	Alu	B1_Mus1	chr10	94919419	94919565	-	0	0	0
LTR	MaLR	MTA_Mm	chr18	34487848	34488243	-	0	0	0
SINE	Alu	B1_Mus2	chr18	34716415	34716561	+	0	0	0
Simple_repeat	Simple_repeat	(A)n	chr18	34716562	34716585	+	0	0	0
LTR	MaLR	MTA_Mm	chr10	12689712	12690083	+	0	0	0
SINE	Alu	B1_Mus2	chr18	34848356	34848484	+	0	0	0
Simple_repeat	Simple_repeat	(A)n	chr18	34848485	34848510	+	0.332194	0	0
SINE	Alu	B1_Mur1	chr18	34935847	34935982	-	0	0	0
SINE	Alu	B1_Mm	chr18	35119173	35119318	+	0	0	0
SINE	Alu	B1_Mus2	chr18	35158065	35158210	+	0	0	0
SINE	B2	B2_Mm1a	chr18	35916925	35917105	+	0	0	0
Simple_repeat	Simple_repeat	(A)n	chr18	35917106	35917132	+	0	0	0.342623
SINE	Alu	B1_Mm	chr10	95085374	95085520	+	0	0	0
Simple_repeat	Simple_repeat	(A)n	chr10	95085521	95085560	+	0	0	0
SINE	Alu	B1_Mm	chr18	36465370	36465512	+	1.18211	0	0
Simple_repeat	Simple_repeat	(A)n	chr18	36465513	36465548	+	0.591053	0	0
SINE	Alu	B1_Mus2	chr18	36553446	36553591	-	0	0	0
LTR	MaLR	MTA_Mm	chr18	36653378	36653772	-	0	0	0
SINE	Alu	B1_Mus2	chr18	36888987	36889133	+	0	0	0
Simple_repeat	Simple_repeat	(A)n	chr18	36889134	36889159	+	0	0	0
LTR	MaLR	MTA_Mm	chr10	95255082	95255476	+	0	0	0
Simple_repeat	Simple_repeat	(A)n	chr18	38184591	38184620	+	0	0	0
Simple_repeat	Simple_repeat	(A)n	chr18	38744664	38744695	+	0	0	0
SINE	Alu	PB1D10	chr18	38988271	38988373	+	0	0	0
Simple_repeat	Simple_repeat	(A)n	chr18	38988374	38988398	+	0	0	0
Simple_repeat	Simple_repeat	(A)n	chr18	39728964	39728992	+	0	0	0
Simple_repeat	Simple_repeat	(TTTA)n	chr18	39733971	39734006	+	0	0	0
Simple_repeat	Simple_repeat	(A)n	chr18	41244228	41244264	+	0	0	0
Simple_repeat	Simple_repeat	(A)n	chr18	41353615	41353650	+	0	0	0
Simple_repeat	Simple_repeat	(A)n	chr18	41654423	41654473	+	0	0	0
SINE	Alu	B1_Mm	chr18	41825925	41826058	-	0.591053	0	0
LTR	MaLR	MTA_Mm	chr18	42161228	42161621	-	0	0	0
SINE	Alu	B1_Mm	chr18	42358387	42358533	-	0	0	0
Simple_repeat	Simple_repeat	(A)n	chr10	95697541	95697579	+	0	0	0
SINE	Alu	B1_Mus2	chr18	42584979	42585115	+	0	0	0
Simple_repeat	Simple_repeat	(A)n	chr18	42585116	42585148	+	0	0	0
Simple_repeat	Simple_repeat	(TTTA)n	chr18	42618184	42618215	+	0	0	0
Simple_repeat	Simple_repeat	(A)n	chr10	95727222	95727260	+	0	0	0
Simple_repeat	Simple_repeat	(A)n	chr18	42753173	42753211	+	0	0	0
LINE	L1	L1Md_T	chr18	43870217	43876428	-	0	0	0
SINE	Alu	B1_Mus1	chr18	43979917	43980064	-	0	0	0
SINE	Alu	B1_Mus1	chr18	44091297	44091438	+	0	0	0
Simple_repeat	Simple_repeat	(A)n	chr18	44091439	44091484	+	0	0	0
Simple_repeat	Simple_repeat	(A)n	chr10	96008661	96008693	+	0	0	0
SINE	Alu	B1_Mm	chr18	46780107	46780252	-	0	0	0
LTR	MaLR	MTA_Mm	chr18	47277938	47278333	+	0	0	0
SINE	Alu	B1_Mm	chr18	47665935	47666087	+	0	0.708814	0
Simple_repeat	Simple_repeat	(A)n	chr18	47666088	47666120	+	0	0	0
LINE	L1	L1Md_T	chr18	48370273	48377565	-	0	0.761137	0
Simple_repeat	Simple_repeat	(A)n	chr18	48466716	48466787	+	0	0	0
LTR	MaLR	MTA_Mm	chr18	48611184	48611578	+	0	0	0
LINE	L1	L1_Mus2	chr18	48746984	48752642	+	0	0	0
SINE	Alu	B1_Mus1	chr18	49243111	49243257	-	0	0	0
Simple_repeat	Simple_repeat	(A)n	chr10	96357538	96357577	+	0	0	0.342623
Simple_repeat	Simple_repeat	(A)n	chr10	96363882	96363918	+	0	0	0
SINE	Alu	B1F2	chr18	50384246	50384368	+	0	0	0
Simple_repeat	Simple_repeat	(A)n	chr18	50384369	50384415	+	0	0	0
LINE	L1	L1Md_T	chr18	50421619	50428042	-	0	0	0
Simple_repeat	Simple_repeat	(A)n	chr18	51059636	51059686	+	0	0	0
Simple_repeat	Simple_repeat	(A)n	chr10	96486012	96486060	+	0	0	0
Simple_repeat	Simple_repeat	(A)n	chr18	52114575	52114614	+	0	0	0
SINE	Alu	B1_Mm	chr18	52735047	52735172	+	0	0	0

Simple_repeat	Simple_repeat	(A)n	chr18	52735173	52735196	+	0	0	0
LTR	MaLR	MTA_Mm	chr18	52769966	52770362	+	0	0	0
LTR	MaLR	MTA_Mm	chr18	52847341	52847733	+	0	0	0
Simple_repeat	Simple_repeat	(A)n	chr18	52917911	52917955	+	0	0	0
SINE	Alu	B1_Mus1	chr10	96653357	96653475	-	0	0	0
SINE	Alu	B1_Mur4	chr18	53321787	53321937	+	0	0	0
Simple_repeat	Simple_repeat	(A)n	chr18	53321938	53321991	+	0	0	0
SINE	Alu	B1_Mus2	chr18	53393377	53393524	-	0	0	0.615324
SINE	Alu	B1_Mus1	chr18	54268311	54268435	-	0	0	0
SINE	Alu	B1_Mm	chr18	54500993	54501138	+	0	0	0
LTR	MaLR	ORR1B2	chr18	54501139	54501487	+	0.332194	0	0
SINE	Alu	B1_Mus1	chr10	96812518	96812665	+	0	0	0
Low_complexity	Low_complexity	A-rich	chr10	96812668	96812776	+	0	0	0
Simple_repeat	Simple_repeat	(A)n	chr18	54899107	54899143	+	0	0	0
Simple_repeat	Simple_repeat	(A)n	chr18	54958509	54958566	+	0	0	0
Simple_repeat	Simple_repeat	(A)n	chr18	54977832	54977857	+	0	0	0
Simple_repeat	Simple_repeat	(A)n	chr10	96895878	96895923	+	0	0	0
Simple_repeat	Simple_repeat	(A)n	chr10	96923244	96923304	+	0	0	0
SINE	Alu	B1_Mm	chr18	55925669	55925822	+	0	0	0
SINE	Alu	B1_Mm	chr18	55991243	55991389	-	0	0	0
Other	Other	RMER1A	chr18	56353455	56354337	+	0	0	0
Simple_repeat	Simple_repeat	(A)n	chr18	56441985	56442018	+	0	0	0
SINE	Alu	B1_Mm	chr18	56775449	56775594	-	0	0	0
SINE	Alu	B1_Mus2	chr18	56836666	56836813	-	0	0	0
SINE	Alu	B1_Mus2	chr18	57088719	57088865	+	0	0	0
LTR	MaLR	MTA_Mm	chr18	57616671	57617066	-	0	0	0
SINE	Alu	B1_Mm	chr18	58123858	58124005	+	0	0	0
Simple_repeat	Simple_repeat	(A)n	chr18	58124006	58124076	+	0	0	0.342623
LTR	MaLR	MTA_Mm	chr18	58666906	58667305	+	0	0	0
SINE	Alu	B1_Mus1	chr18	58917896	58918042	-	0	0	0
SINE	Alu	B1_Mus1	chr18	59297080	59297213	-	0	0	0
Simple_repeat	Simple_repeat	(A)n	chr18	59579556	59579596	+	2.36421	1.41763	0.615324
Simple_repeat	Simple_repeat	(A)n	chr18	59741725	59741771	+	0	0	0
Simple_repeat	Simple_repeat	(A)n	chr18	59897335	59897361	+	0	0	0
LINE	L1	L1Md_T	chr18	60107452	60114739	-	0	0	0
LINE	L1	L1_Mus1	chr18	60321314	60327628	+	0	0	0
Simple_repeat	Simple_repeat	(A)n	chr18	61449375	61449405	+	0	0.708814	0
SINE	Alu	B1_Mus1	chr18	62102137	62102280	-	0	0	0
Simple_repeat	Simple_repeat	(A)n	chr18	62264007	62264043	+	0	0	0
LTR	MaLR	MTB	chr18	62437989	62438387	+	0	0	0
Simple_repeat	Simple_repeat	(A)n	chr10	97693772	97693807	+	0	0	0
Simple_repeat	Simple_repeat	(A)n	chr18	62952345	62952381	+	0	0	0
Simple_repeat	Simple_repeat	(A)n	chr18	64494502	64494540	+	0	0	0
SINE	Alu	B1_Mus1	chr18	65708481	65708626	+	0	0	0
Simple_repeat	Simple_repeat	(A)n	chr18	65708627	65708658	+	0	0	0
SINE	Alu	B1_Mm	chr18	65849648	65849794	-	0	0	0
LTR	MaLR	MTA_Mm	chr18	65947192	65947572	+	0	0	0
Simple_repeat	Simple_repeat	(A)n	chr18	66475516	66475549	+	0	0	0
LTR	MaLR	MTA_Mm	chr18	66884398	66884790	-	0.332194	0	0
Simple_repeat	Simple_repeat	(A)n	chr18	67409855	67409895	+	0	0	0.615324
SINE	Alu	B1_Mus2	chr18	67433700	67433827	+	0	0	0
Simple_repeat	Simple_repeat	(A)n	chr18	67433828	67433859	+	0	0	0
SINE	Alu	B1_Mus2	chr18	67821100	67821240	+	0	0	0
Simple_repeat	Simple_repeat	(A)n	chr18	67821241	67821288	+	0.332194	0	0
SINE	Alu	B1_Mm	chr18	67925778	67925925	+	0	0	0
LTR	MaLR	MTA_Mm	chr18	68073372	68073767	-	0	0	0.342623
LTR	MaLR	MTA_Mm	chr18	68074865	68075260	-	0	0	0
LTR	MaLR	MTB	chr18	69431918	69432301	+	0	0	0
LTR	MaLR	MTA_Mm	chr18	69919004	69919397	-	0	0	0
LTR	MaLR	MTA_Mm	chr18	69920497	69920891	-	0	0	0
SINE	Alu	B1_Mus1	chr18	69958118	69958266	-	0	0	0
LTR	MaLR	MTB	chr18	70135641	70136040	-	0	0	0
SINE	Alu	B1_Mus1	chr18	70696821	70696968	-	0	0	0
LINE	L1	L1Md_T	chr18	70934968	70941530	-	0	0	0
SINE	Alu	B1_Mm	chr18	71041419	71041565	-	0	0	0
LTR	MaLR	MTB	chr18	72762758	72763155	-	0	0	0
Simple_repeat	Simple_repeat	(A)n	chr18	73201707	73201744	+	0	0	0

Simple_repeat	Simple_repeat	(A)n	chr18	73254331	73254363	+	0	0	0
Simple_repeat	Simple_repeat	(A)n	chr18	73265269	73265301	+	0	0	0
LTR	MaLR	MTA_Mm	chr18	73531376	73531768	-	0	0	0
Simple_repeat	Simple_repeat	(A)n	chr18	73756797	73756838	+	0	0	0
SINE	Alu	B1_Mus2	chr18	74268085	74268225	+	0	0	0
Simple_repeat	Simple_repeat	(A)n	chr18	74268226	74268252	+	0	0	0
SINE	Alu	B1_Mus1	chr18	74396249	74396396	+	0	0	0
Simple_repeat	Simple_repeat	(A)n	chr18	74396397	74396434	+	0	0	0
Simple_repeat	Simple_repeat	(A)n	chr18	75448185	75448228	+	0	0	0.615324
LTR	MaLR	MTB	chr18	75578559	75578959	+	0	0	0
LTR	MaLR	MTA_Mm	chr10	99165030	99165410	-	0	0	0
Low_complexity	Low_complexity	A-rich	chr18	77978973	77979078	+	0	0	0
LTR	MaLR	MTA_Mm	chr18	78806194	78806563	+	0	0	0
Simple_repeat	Simple_repeat	(A)n	chr18	78816285	78816311	+	0	0	0
Simple_repeat	Simple_repeat	(A)n	chr18	79716502	79716541	+	0	0.380568	0
SINE	Alu	B1_Mm	chr18	79815913	79816059	-	0	0	0
Simple_repeat	Simple_repeat	(A)n	chr18	80016999	80017031	+	0	0	0
LTR	MaLR	MTB	chr18	80107558	80107955	+	0	0	0
LTR	MaLR	MTA_Mm	chr18	80576457	80576853	+	0	0	0
LTR	MaLR	MTB	chr10	99574978	99575374	+	0	0	0
Simple_repeat	Simple_repeat	(A)n	chr18	81553335	81553374	+	0	0	0.615324
LTR	MaLR	MTA_Mm	chr18	81698536	81698930	+	0	0	0
Simple_repeat	Simple_repeat	(A)n	chr18	82319965	82319994	+	0	0	0
SINE	Alu	B1_Mus1	chr18	83052231	83052372	-	0	0	0
SINE	Alu	B1_Mm	chr18	83259215	83259361	-	0	0	0
Simple_repeat	Simple_repeat	(A)n	chr18	83371820	83371855	+	0	0	0
Simple_repeat	Simple_repeat	(A)n	chr18	83551244	83551275	+	0	0	0
LTR	MaLR	MTA_Mm	chr18	84766836	84767226	-	0	0	0
SINE	Alu	B1_Mus2	chr18	84856809	84856955	+	0	0	0
Simple_repeat	Simple_repeat	(A)n	chr18	84856956	84856979	+	0	0	0
SINE	Alu	B1_Mus2	chr18	84864102	84864248	+	0	0	0
Simple_repeat	Simple_repeat	(A)n	chr18	84864249	84864271	+	0	0	0
SINE	Alu	B1_Mus2	chr18	85239437	85239583	-	0	0	0
LINE	L1	L1Md_T	chr18	85243799	85249965	-	0	0	0
LTR	MaLR	MTB	chr18	85762455	85762854	-	0.591053	0	0
LINE	L1	L1Md_F2	chr18	86052965	86058206	+	0	0	0
Simple_repeat	Simple_repeat	(A)n	chr18	86126430	86126464	+	0	0	0
LINE	L1	L1Md_T	chr10	10013795	10014421	-	0	0	0
SINE	Alu	B1_Mm	chr18	86553986	86554135	-	0	0	0
Simple_repeat	Simple_repeat	(A)n	chr18	86893504	86893535	+	0.591053	0	0
Simple_repeat	Simple_repeat	(A)n	chr18	87120587	87120634	+	0	0	0
Simple_repeat	Simple_repeat	(A)n	chr18	87402035	87402077	+	0	0	0
LINE	L1	L1Md_T	chr18	87946452	87952725	-	0	0	0
LINE	L1	L1Md_F3	chr18	88095741	88100732	+	0	0	0
SINE	Alu	B1_Mus1	chr18	88184852	88184999	+	0	0	0
LTR	ERVK	RLTR40	chr18	88302633	88303354	-	0	0	0
SINE	Alu	B1_Mus1	chr18	88449432	88449582	-	0	0	0
SINE	Alu	B1_Mus1	chr18	88723407	88723553	-	0	0	0
Simple_repeat	Simple_repeat	(A)n	chr18	89939591	89939636	+	0.332194	0.708814	0.615324
LTR	MaLR	MTA_Mm	chr10	10053254	10053294	-	0.591053	0	0
LTR	MaLR	MTA_Mm	chr10	10053400	10053439	-	0	0	0
SINE	Alu	B1_Mus1	chr19	3705813	3705953	-	0	0	0
SINE	Alu	B1_Mm	chr19	3827422	3827568	+	0	0	0
Simple_repeat	Simple_repeat	(A)n	chr19	3827569	3827608	+	0	0	0
SINE	Alu	B1_Mm	chr19	3828136	3828274	+	0	0	0
Simple_repeat	Simple_repeat	(A)n	chr19	3828275	3828297	+	0	0	0
LTR	MaLR	MTA_Mm	chr19	4074319	4074712	-	0	0	0
SINE	Alu	B1_Mm	chr19	4090151	4090297	-	0	0	0
SINE	Alu	B1_Mus1	chr19	4211142	4211289	+	0	0	0
Simple_repeat	Simple_repeat	(A)n	chr19	4211290	4211309	+	0	0	0
Simple_repeat	Simple_repeat	(A)n	chr10	10078193	10078196	+	0	0	0
SINE	Alu	B1_Mus1	chr19	4845064	4845208	+	0	0	0
Simple_repeat	Simple_repeat	(A)n	chr19	4845209	4845240	+	0	0	0
SINE	Alu	B1_Mus2	chr19	5017178	5017324	+	0	0	0
Simple_repeat	Simple_repeat	(A)n	chr19	5017325	5017347	+	0	0	0
SINE	Alu	B1_Mus1	chr19	5077728	5077874	-	0	0	0.615324
SINE	Alu	B1_Mm	chr19	5124031	5124178	+	0	0	0

Simple_repeat	Simple_repeat	(A)n	chr19	5124179	5124226	+	0	0	0
SINE	Alu	B1_Mus1	chr19	5427612	5427730	+	0	0	0.615324
Simple_repeat	Simple_repeat	(A)n	chr19	5427731	5427751	+	0	0	0.615324
Simple_repeat	Simple_repeat	(A)n	chr19	6096511	6096538	+	0	0	0
SINE	Alu	B1_Mm	chr19	6584348	6584468	-	0	0	0
SINE	B2	B2_Mm1a	chr19	6679833	6680024	-	0	0	0
SINE	B2	B2_Mm1a	chr19	7293964	7294154	-	0	0	0
SINE	B2	B2_Mm1a	chr19	7327208	7327400	+	0	0	0
Simple_repeat	Simple_repeat	(A)n	chr19	7327401	7327436	+	0	0	0
SINE	Alu	PB1D9	chr19	8760132	8760229	-	0	0	0
SINE	Alu	B1_Mus2	chr19	8939330	8939476	-	0	0	0.342623
Simple_repeat	Simple_repeat	(A)n	chr19	8944989	8945020	+	0	0	0
LTR	MaLR	MTA_Mm	chr19	10435750	10436143	-	0	0	0
SINE	Alu	B1_Mus1	chr19	10706805	10706950	+	0	0.380568	0
Simple_repeat	Simple_repeat	(A)n	chr19	10706951	10706999	+	0	0	0
SINE	Alu	B1_Mm	chr19	11802292	11802438	+	0	0	0
Simple_repeat	Simple_repeat	(GAAAA)n	chr19	11802439	11802562	+	0	0	0
SINE	Alu	B1_Mus1	chr19	11957739	11957860	-	0	0	0
SINE	Alu	B1_Mus1	chr19	12231754	12231896	+	0	0	0
Simple_repeat	Simple_repeat	(A)n	chr19	12231897	12231916	+	0	0	0
SINE	Alu	B1_Mm	chr19	12796790	12796936	-	0	0	0
SINE	Alu	B1_Mm	chr19	12829338	12829483	+	0	0	0
Simple_repeat	Simple_repeat	(A)n	chr19	12829484	12829534	+	0	0.380568	0
LTR	MaLR	MTA_Mm	chr19	13314864	13315258	+	0	0	0
SINE	Alu	B1_Mus1	chr19	13334458	13334606	-	0	0	0
LTR	MaLR	MTA_Mm	chr19	14174456	14174851	+	0	0	0
SINE	Alu	B1_Mus2	chr19	14717569	14717715	+	0	0	0
Simple_repeat	Simple_repeat	(A)n	chr19	14717716	14717745	+	0	0	0
Simple_repeat	Simple_repeat	(A)n	chr19	14890298	14890347	+	0	0	0
SINE	Alu	B1_Mus1	chr10	10194394	10194409	+	0	0	0
Simple_repeat	Simple_repeat	(A)n	chr10	10194409	10194412	+	0	0	0
SINE	Alu	B1_Mus2	chr19	15003945	15004091	+	0	0	0
Simple_repeat	Simple_repeat	(A)n	chr19	15004099	15004127	+	0	0	0
Simple_repeat	Simple_repeat	(A)n	chr19	15393298	15393324	+	0	0	0
SINE	Alu	B1_Mus2	chr19	15475002	15475153	-	0	0	0
SINE	Alu	B1_Mm	chr19	15605740	15605890	-	0	0	0
SINE	B2	B2_Mm1a	chr19	15959439	15959633	-	0	0	0
SINE	Alu	B1_Mm	chr19	16001482	16001628	+	0	0	0
LTR	MaLR	MTA_Mm	chr19	16087981	16088370	+	0	0	0
LTR	MaLR	MTA_Mm	chr19	18213872	18214265	+	0	0	0
LTR	MaLR	MTA_Mm	chr10	10230185	10230224	+	0	0	0
SINE	Alu	B1_Mus2	chr19	18647040	18647195	+	0	0	0
Simple_repeat	Simple_repeat	(A)n	chr19	18647196	18647234	+	0	0	0
SINE	Alu	B1_Mus1	chr19	18648926	18649073	-	0.332194	0	0
Simple_repeat	Simple_repeat	(A)n	chr10	10233774	10233783	+	0	0	0
SINE	Alu	B1_Mm	chr19	18743477	18743621	-	0	0	0
SINE	Alu	B1_Mus1	chr19	18794272	18794418	-	0	0	0
LTR	MaLR	MTA_Mm	chr19	19593980	19594375	-	0	0	0
LTR	MaLR	MTA_Mm	chr19	20034010	20034405	+	0	0	0
LTR	MaLR	MTA_Mm	chr19	20122207	20122602	+	0	0	0
LTR	MaLR	MTA_Mm	chr19	20261434	20261829	-	0	0	0
SINE	Alu	B1_Mus1	chr19	20304059	20304177	+	0	0	0
Simple_repeat	Simple_repeat	(A)n	chr19	20304178	20304211	+	0	0	0
Simple_repeat	Simple_repeat	(A)n	chr10	10250894	10250896	+	0.332194	0	0
LINE	L1	L1Md_T	chr19	20641820	20648285	-	0	0	0
LTR	MaLR	MTA_Mm	chr19	22039581	22039975	-	0.332194	0	0
LTR	MaLR	MTA_Mm	chr19	22041075	22041469	-	0	0	0
SINE	Alu	B1_Mm	chr19	22164720	22164867	+	0.591053	1.7982	1.23065
Simple_repeat	Simple_repeat	(A)n	chr19	22164868	22164888	+	0.591053	1.08938	1.23065
LTR	MaLR	MTA_Mm	chr19	22204308	22204702	-	0	0	0
LTR	MaLR	MTA_Mm	chr19	23104524	23104920	-	0	0	0
SINE	Alu	B1_Mm	chr19	23133864	23134010	-	0	0	0.342623
SINE	Alu	B1_Mus2	chr19	23134048	23134194	-	0	0	0
Simple_repeat	Simple_repeat	(GAA)n	chr19	23149084	23149140	+	0	0	0
LTR	MaLR	MTA_Mm	chr19	23175485	23175880	+	0	0	0
SINE	Alu	B1_Mus1	chr19	23177712	23177859	-	0	0	0
SINE	Alu	B1_Mus2	chr10	10291654	10291669	+	0	0	0

LTR	MaLR	MTA_Mm	chr10	10298541	10298581	-	0	0	0
LTR	MaLR	MTA_Mm	chr10	10298690	10298729	-	0	0	0
Simple_repeat	Simple_repeat	(A)n	chr10	10306608	10306612	+	0	0	0
LTR	MaLR	MTA_Mm	chr19	25907230	25907629	+	0	0	0
LTR	MaLR	MTEa	chr19	26133047	26133394	-	0	0	0
SINE	Alu	B1_Mus1	chr19	26361369	26361516	+	0	0	0
Simple_repeat	Simple_repeat	(A)n	chr19	26361517	26361549	+	0.332194	0	0
LTR	MaLR	MTA_Mm	chr19	26610794	26611184	-	0	0	0
LTR	MaLR	MTB	chr19	26617862	26618241	+	0	0	0.615324
Simple_repeat	Simple_repeat	(A)n	chr19	26998710	26998747	+	0	0	0
SINE	Alu	B1_Mus1	chr19	27102135	27102283	-	0	0	0.342623
SINE	Alu	B1_Mus2	chr19	27417240	27417386	+	0	0	0
LTR	MaLR	MTA_Mm	chr19	27513852	27514248	-	0	0	0
SINE	Alu	B1_Mm	chr19	27572527	27572674	-	0	0	0
LTR	MaLR	MTB	chr19	28258806	28259201	+	0	0	0
SINE	Alu	B1_Mus2	chr19	28889841	28889987	+	0	0	0
Simple_repeat	Simple_repeat	(A)n	chr19	28889988	28890008	+	0	0	0
SINE	Alu	B1_Mm	chr19	28908738	28908884	+	0	0	0
Simple_repeat	Simple_repeat	(A)n	chr19	28908885	28908910	+	0	0	0
LTR	MaLR	MTB	chr10	10338822	10338860	+	0	0	0
SINE	Alu	B1_Mus2	chr19	29909683	29909829	+	0	0	0
Simple_repeat	Simple_repeat	(A)n	chr19	29909830	29909876	+	0	0	0
Simple_repeat	Simple_repeat	(A)n	chr10	10354169	10354176	+	0	0	0
Simple_repeat	Simple_repeat	(A)n	chr10	10356117	10356123	+	0	0	0
SINE	B2	B2_Mm1t	chr19	32192069	32192256	+	0	0	0
LTR	MaLR	MTA_Mm	chr19	33053204	33053602	-	0	0	0
scRNA	scRNA	4.5SRNA	chr19	33191619	33191711	-	0	0	0
Simple_repeat	Simple_repeat	(A)n	chr19	34525384	34525431	+	0.332194	0	0
SINE	Alu	B1_Mus2	chr19	34870294	34870439	-	0	0	0.342623
SINE	Alu	B1_Mm	chr19	34984337	34984485	+	0	0	0
Simple_repeat	Simple_repeat	(A)n	chr19	34984486	34984524	+	0	0	0
SINE	Alu	B1_Mur2	chr19	35098062	35098207	+	0	0	0
Simple_repeat	Simple_repeat	(A)n	chr19	35779707	35779758	+	0	0	0.615324
LTR	MaLR	MTA_Mm	chr19	35794164	35794557	-	0	0	0
SINE	Alu	B1_Mus1	chr19	35923113	35923258	+	0	0	0
SINE	Alu	B1_Mus2	chr19	36893551	36893697	-	0	0	0
Simple_repeat	Simple_repeat	(A)n	chr19	37449267	37449309	+	0	0.380568	0.615324
SINE	Alu	B1_Mm	chr19	37943755	37943897	+	0	0	0
Simple_repeat	Simple_repeat	(A)n	chr19	37943898	37943918	+	0	0	0
SINE	Alu	B1_Mus2	chr19	37946337	37946482	+	0	0	0
Simple_repeat	Simple_repeat	(GAAA)n	chr19	37946485	37946550	+	0	0	0
SINE	Alu	B1_Mm	chr19	38402108	38402254	-	0.591053	0	0
SINE	Alu	B1_Mm	chr19	38409012	38409158	+	0	0	0
Simple_repeat	Simple_repeat	(A)n	chr19	38409159	38409182	+	0	0	0
SINE	Alu	B1_Mus2	chr19	38496837	38496984	-	0	0	0
Simple_repeat	Simple_repeat	(A)n	chr10	10447293	10447297	+	0	0	0
LINE	L1	Lx2	chr19	40189487	40194397	+	0	0	0
LTR	MaLR	MTA_Mm	chr19	40363599	40363996	+	0	0	0
SINE	Alu	B1_Mus2	chr19	41608543	41608655	+	0	0.708814	0
Simple_repeat	Simple_repeat	(A)n	chr19	41608656	41608682	+	0	0.708814	0
SINE	Alu	B1_Mus2	chr19	41651768	41651900	+	0	0	0.342623
Simple_repeat	Simple_repeat	(A)n	chr19	41651901	41651926	+	0	0.380568	0.342623
SINE	Alu	B1_Mur4	chr19	41897400	41897552	+	0	0	0
Simple_repeat	Simple_repeat	(A)n	chr19	41897553	41897592	+	0	0	0
Simple_repeat	Simple_repeat	(A)n	chr19	41904105	41904152	+	0	0	0
SINE	Alu	B1_Mur3	chr19	41985776	41985909	+	0	0	0
Simple_repeat	Simple_repeat	(A)n	chr19	41985910	41985934	+	0	0	0
SINE	Alu	B1_Mus1	chr19	42231957	42232103	+	0	0	0
Simple_repeat	Simple_repeat	(A)n	chr19	42232104	42232131	+	0.332194	0.380568	0
SINE	Alu	B1_Mm	chr19	43718488	43718631	-	0	0	0
LTR	MaLR	MTA_Mm	chr19	44914848	44915243	-	1.16015	0	0
LTR	MaLR	MTA_Mm	chr19	44916601	44916995	-	0	0	0
SINE	Alu	B1_Mm	chr19	45384107	45384243	-	0	0	0
LTR	MaLR	MTA_Mm	chr19	45788033	45788427	-	0	0	0
SINE	Alu	B1_Mus2	chr19	46124381	46124516	+	0	0	0
Simple_repeat	Simple_repeat	(A)n	chr19	46124517	46124543	+	0	0	0
SINE	Alu	B1_Mm	chr19	46199986	46200132	+	0	0	0

Simple_repeat	Simple_repeat	(A)n	chr19	46200133	46200170	+	0	0	0
SINE	Alu	B1_Mus2	chr19	46422425	46422570	+	0	0	0
Simple_repeat	Simple_repeat	(A)n	chr19	46422571	46422596	+	0	0	0
SINE	Alu	B1_Mus2	chr19	46430761	46430906	-	0	0	0
Simple_repeat	Simple_repeat	(A)n	chr19	48883820	48883866	+	0	0	0
LTR	MaLR	MTA_Mm	chr19	49081293	49081684	-	0	0	0
LTR	ERVK	RMER6A	chr19	49400680	49401465	-	0	0	0
Simple_repeat	Simple_repeat	(A)n	chr19	49570268	49570304	+	0	0	0
Simple_repeat	Simple_repeat	(A)n	chr19	49783242	49783281	+	0	0	0
Simple_repeat	Simple_repeat	(A)n	chr19	51439220	51439287	+	0	0	0
LINE	L1	L1Md_T	chr19	51618350	51624488	-	0	0	0
LTR	MaLR	MTA_Mm	chr19	52967003	52967384	-	0	0	0
SINE	Alu	B1_Mm	chr19	53655435	53655581	-	0	0	0
SINE	Alu	B1_Mus2	chr19	53730937	53731080	+	0	0	0
Simple_repeat	Simple_repeat	(A)n	chr19	53731081	53731104	+	0	0	0
LTR	ERVK	RMER19C	chr19	54134835	54135556	-	0	0	0
SINE	Alu	B1_Mus2	chr19	54152806	54152955	-	0	0	0
Simple_repeat	Simple_repeat	(A)n	chr19	54253620	54253653	+	0	0	0
SINE	Alu	B1_Mus1	chr19	54581927	54582072	+	0	0	0
Simple_repeat	Simple_repeat	(A)n	chr19	54582073	54582096	+	0	0	0
SINE	B4	B4	chr19	54582609	54582942	-	0	0	0
SINE	Alu	B1_Mm	chr19	56592810	56592956	+	0	0	0
Simple_repeat	Simple_repeat	(A)n	chr19	56592957	56593003	+	0	0	0
Simple_repeat	Simple_repeat	(A)n	chr19	57317526	57317556	+	0	0	0
SINE	Alu	B1_Mm	chr19	57484311	57484459	-	0	0	0
SINE	Alu	B1_Mus1	chr19	58668834	58668981	+	0	0	0
SINE	Alu	B1_Mus2	chr19	58672924	58673067	+	0	0	0
Simple_repeat	Simple_repeat	(A)n	chr19	58673068	58673099	+	0	0	0
SINE	Alu	B1_Mm	chr19	58730706	58730852	+	0	0	0
Simple_repeat	Simple_repeat	(A)n	chr19	58730853	58730879	+	0	0	0
SINE	Alu	B1_Mm	chr19	59254812	59254952	+	0	0	0
SINE	Alu	B1_Mus1	chr19	60448860	60449004	-	0	0	0
SINE	Alu	B1_Mm	chr19	60876199	60876335	-	0	0	0
SINE	Alu	B1_Mus2	chr19	60958032	60958180	+	0	0	0
Simple_repeat	Simple_repeat	(A)n	chr19	60958181	60958204	+	0	0	0
LTR	MaLR	MLT1A	chr1	3158390	3158755	+	0	0	0
SINE	Alu	B1_Mus1	chr1	3895094	3895241	+	0	0	0
Low_complexity	Low_complexity	GA-rich	chr1	3895243	3895307	+	0.591053	0	0
LTR	MaLR	MTA_Mm	chr1	4459694	4460087	-	0	0	0
LTR	MaLR	MTA_Mm	chr1	5363027	5363399	+	0	0	0
SINE	Alu	B1_Mm	chr1	5974350	5974496	+	0	0	0
LTR	MaLR	MTA_Mm	chr1	6118540	6118930	+	0	0	0
Simple_repeat	Simple_repeat	(A)n	chr1	6166783	6166808	+	0	0	0
LTR	MaLR	MTA_Mm	chr1	6316887	6317281	-	0	0	0
Simple_repeat	Simple_repeat	(A)n	chr1	7186902	7186942	+	0	0	0
LINE	L1	L1Md_T	chr1	7318763	7325821	-	0	0	0
Simple_repeat	Simple_repeat	(A)n	chr1	7379898	7379924	+	0	0	0
SINE	Alu	B1_Mus1	chr1	7383886	7384024	-	0	0	0
Simple_repeat	Simple_repeat	(A)n	chr1	7384121	7384170	+	0	0	0
Simple_repeat	Simple_repeat	(A)n	chr10	10758043	10758047	+	0	0	0
Simple_repeat	Simple_repeat	(A)n	chr1	7736046	7736092	+	0	0	0
SINE	Alu	B1_Mus2	chr1	8225645	8225791	-	0	0	0
LTR	MaLR	MTA_Mm	chr10	13926283	13926680	+	0	0	1.10507
Simple_repeat	Simple_repeat	(A)n	chr1	10280912	10281032	+	0	0	0
SINE	Alu	B1_Mus2	chr1	10743193	10743339	-	0	0	0
SINE	Alu	B1_Mm	chr1	10869851	10869944	+	0	0	0.342623
Simple_repeat	Simple_repeat	(A)n	chr1	10869945	10869990	+	0	0	0.342623
SINE	Alu	B1_Mus1	chr10	10786471	10786485	+	0	0	0.342623
Simple_repeat	Simple_repeat	(A)n	chr10	10786485	10786489	+	0	0	0.342623
SINE	Alu	B1_Mus1	chr1	12076729	12076875	-	0	0	0.615324
LTR	MaLR	MTA_Mm	chr1	12137289	12137684	+	0	0	0
Simple_repeat	Simple_repeat	(A)n	chr1	12213548	12213577	+	0	0	0
LTR	MaLR	MTB	chr1	12214505	12214876	+	0	0	0
LINE	L1	L1_Mus2	chr1	12350343	12355914	+	0	0	0
LINE	L1	L1Md_F2	chr1	12447775	12453014	+	0	0	0
LTR	MaLR	MTA_Mm	chr1	12507060	12507455	-	0	0	0
LTR	MaLR	MTA_Mm	chr1	13024878	13025261	-	0	0	0

SINE	Alu	B1_Mm	chr1	13438957	13439103	+	0	0	0
LTR	MaLR	MTA_Mm	chr1	13442647	13443045	-	0.332194	0	0
Simple_repeat	Simple_repeat	(A)n	chr1	13579979	13580019	+	0	0.380568	0
SINE	Alu	B1F	chr1	13582238	13582381	-	0	0	0
LTR	MaLR	MTA_Mm	chr1	13701247	13701641	+	0	0	0
LTR	MaLR	MTA_Mm	chr1	13712190	13712583	+	0	0	0
LTR	MaLR	MTA_Mm	chr1	13713683	13714076	+	0	0	0
SINE	Alu	B1_Mm	chr1	14460313	14460459	-	0	0	0
Simple_repeat	Simple_repeat	(A)n	chr1	14808372	14808426	+	0	0	0
Simple_repeat	Simple_repeat	(A)n	chr1	14909142	14909180	+	0	0	0
LTR	MaLR	MTA_Mm	chr1	14911900	14912289	-	0	0	0
Simple_repeat	Simple_repeat	(TTTA)n	chr1	15950048	15950108	+	0	0	0
SINE	Alu	B1_Mus1	chr1	16087059	16087203	+	0	0	0
SINE	Alu	B1_Mm	chr1	16712179	16712324	-	0	0	0
SINE	Alu	B1_Mus1	chr1	16842186	16842333	-	0	0	0
LINE	L1	L1_Mus2	chr1	17128125	17133630	-	0	0	0
SINE	B2	B2_Mm1t	chr1	17224679	17224871	-	0.332194	0	0
Simple_repeat	Simple_repeat	(A)n	chr1	17688764	17688804	+	0	0	0
LTR	MaLR	MTA_Mm	chr1	17701866	17702245	-	0	0	0
LINE	L1	L1Md_T	chr1	17906212	17912668	-	0	0	0
LTR	MaLR	MTA_Mm	chr1	17996654	17997047	-	0	0	0.342623
SINE	Alu	B1_Mus1	chr1	18470778	18470924	+	0	0	0
Simple_repeat	Simple_repeat	(A)n	chr1	18631436	18631484	+	0	0	0
LTR	MaLR	MTA_Mm	chr1	18771358	18771724	+	0	0	0
LINE	L1	L1_Mus1	chr1	18904036	18909923	-	0	0	0
LTR	MaLR	MTA_Mm	chr1	19014247	19014641	+	0	0	0
LINE	L1	L1Md_F	chr1	19261551	19266776	+	0	0	0
SINE	Alu	B1_Mus1	chr10	10871125	10871140	+	0	0	0
Low_complexity	Low_complexity	GA-rich	chr10	10871140	10871149	+	0	0	0
SINE	Alu	B1_Mm	chr1	19532328	19532459	-	0	0	0
LTR	MaLR	MTA_Mm	chr1	22905052	22905445	+	0	0	0
Simple_repeat	Simple_repeat	(A)n	chr1	23046296	23046329	+	0	0	0
Simple_repeat	Simple_repeat	(A)n	chr1	23216073	23216100	+	0	0	0
SINE	Alu	B1_Mus1	chr1	23227188	23227334	+	0	0	0
Simple_repeat	Simple_repeat	(CAAAA)n	chr1	23227335	23227443	+	0	0	0
LTR	MaLR	MTA_Mm	chr1	23628441	23628835	+	0	0	0
LTR	MaLR	MTB	chr1	23724107	23724501	+	0	0	0
Simple_repeat	Simple_repeat	(A)n	chr1	24892423	24892490	+	0	0	0
LTR	ERVK	RLTR25A	chr1	24904866	24905869	-	0	0	0
SINE	Alu	B1_Mm	chr10	14089893	14090035	-	0	0	0
SINE	Alu	B1_Mm	chr1	25067710	25067856	+	0	0	0
Simple_repeat	Simple_repeat	(A)n	chr1	25068897	25068927	+	0	0	0
Simple_repeat	Simple_repeat	(TTTA)n	chr1	25909043	25909065	+	0.591053	0	0
Simple_repeat	Simple_repeat	(A)n	chr10	14094117	14094187	+	0	0	0
LINE	L1	L1_Mus3	chr1	26013440	26019720	-	0	0	0
SINE	Alu	B1_Mm	chr1	26092146	26092292	+	0	0	0
SINE	Alu	B1_Mus1	chr1	26457791	26457938	-	0	0	0
SINE	Alu	B1_Mus2	chr1	26801855	26802000	+	0	0	0.615324
LINE	L1	L1Md_F2	chr1	27047027	27052077	+	0	0	0
Simple_repeat	Simple_repeat	(A)n	chr1	27246077	27246111	+	0.332194	0	0
LTR	MaLR	MTA_Mm	chr10	10955646	10955685	-	7.93678	4.15543	4.86694
LTR	MaLR	MTA_Mm	chr1	27744272	27744665	-	0	0	0
Simple_repeat	Simple_repeat	(A)n	chr1	28557389	28557422	+	0	0	0
LINE	L1	L1Md_F	chr1	28571431	28575921	+	0	0	0
Simple_repeat	Simple_repeat	(A)n	chr1	28685159	28685282	+	0	0	0
SINE	Alu	B1_Mus2	chr1	28861585	28861731	-	0	0	0
Simple_repeat	Simple_repeat	(A)n	chr1	29279945	29279975	+	0	0	0
Simple_repeat	Simple_repeat	(A)n	chr10	10977416	10977419	+	0	0	0
SINE	B2	B3A	chr1	29833803	29834006	-	0	0	0
Simple_repeat	Simple_repeat	(A)n	chr1	29971928	29971964	+	0	0	0
LTR	MaLR	MTA_Mm	chr1	30158936	30159330	-	0	0	0
Simple_repeat	Simple_repeat	(A)n	chr1	30528675	30528710	+	0	0	0
Simple_repeat	Simple_repeat	(A)n	chr1	30532249	30532289	+	0	0	0
LINE	L1	L1Md_T	chr1	30717415	30723900	-	0	0	0
SINE	Alu	B1_Mus2	chr1	30963441	30963587	+	0	0	0
Simple_repeat	Simple_repeat	(A)n	chr1	30963588	30963611	+	0	0	0
SINE	Alu	B1_Mus2	chr1	31669624	31669770	+	0	0	0

Simple_repeat	Simple_repeat	(A)n	chr1	31669771	31669823	+	0	0	0
LTR	MaLR	MTA_Mm	chr1	31827931	31828326	-	0	0	0
LTR	MaLR	MTA_Mm	chr1	31829418	31829813	-	0	0	0
Simple_repeat	Simple_repeat	(A)n	chr1	32036162	32036198	+	0	0	0
LTR	MaLR	MTA_Mm	chr10	11010427	11010466	-	0	0	0
LTR	MaLR	MTA_Mm	chr10	11010576	11010616	-	0	0	0
Simple_repeat	Simple_repeat	(A)n	chr1	32804228	32804251	+	0	0	0
SINE	Alu	B1_Mus2	chr10	11015227	11015241	-	0	0	0
SINE	Alu	B1_Mus2	chr1	33261708	33261847	-	0	0	0
LINE	L1	L1_Mus2	chr1	33399530	33405715	+	0	0	0
Simple_repeat	Simple_repeat	(A)n	chr1	33801796	33801828	+	0	0	0
SINE	Alu	PB1D9	chr1	34458705	34458815	-	0	0	0
Simple_repeat	Simple_repeat	(A)n	chr1	34797641	34797678	+	0	0	0
LTR	MaLR	MTA_Mm	chr1	35232788	35233182	-	0	0	0
LTR	MaLR	MTA_Mm	chr1	35236546	35236942	-	0	0	0
LTR	MaLR	MTA_Mm	chr1	35319340	35319729	+	0	0	0
SINE	Alu	B1_Mus1	chr1	35394174	35394317	+	0	0	0
Simple_repeat	Simple_repeat	(A)n	chr1	35394318	35394346	+	0	0	0
Simple_repeat	Simple_repeat	(A)n	chr1	35756051	35756082	+	0	0	0
SINE	Alu	B1_Mm	chr1	36079100	36079250	+	0	0	0
SINE	B2	B2_Mm2	chr1	36101237	36101424	+	0	0	0
LTR	MaLR	MTB	chr10	11049098	11049138	+	0	0	0
SINE	Alu	B1_Mur3	chr1	37140556	37140708	-	0	0	0
SINE	Alu	B1_Mus2	chr10	11054555	11054570	+	0	0	0
Simple_repeat	Simple_repeat	(A)n	chr10	11054570	11054576	+	0	0	0
SINE	Alu	B1_Mus1	chr1	37468941	37469087	-	0	0	0
SINE	Alu	B1_Mm	chr10	11057331	11057346	+	0	0	0
Simple_repeat	Simple_repeat	(A)n	chr10	11057346	11057350	+	0	0	0
SINE	Alu	B1_Mm	chr10	11059640	11059655	-	0	0	0
LINE	L1	Lx2B	chr1	37993708	38000000	-	0	0	0
LTR	MaLR	MTA_Mm	chr1	38037315	38037708	-	0	0	0
SINE	Alu	B1_Mus2	chr1	38731063	38731208	+	0	0	0
Simple_repeat	Simple_repeat	(A)n	chr1	39672535	39672570	+	0	0	0
LTR	MaLR	MTA_Mm	chr1	40103785	40104179	-	0	0	0
LTR	MaLR	MTA_Mm	chr1	40105275	40105669	-	0	0	0
SINE	Alu	B1_Mm	chr10	11079942	11079956	+	0	0	0
LTR	MaLR	MTA_Mm	chr1	40428697	40429073	+	0	0	0
LTR	MaLR	MTA_Mm	chr1	40609711	40610109	-	0	0	0
Simple_repeat	Simple_repeat	(A)n	chr1	41332932	41333006	+	0	0	0
LINE	L1	L1Md_F2	chr1	41376983	41381722	-	0	0	0
LINE	L1	L1Md_F2	chr10	14294518	14300080	-	0	0	0.342623
SINE	Alu	B1_Mm	chr10	14300081	14300227	-	0	0	0.342623
SINE	Alu	B1_Mm	chr10	11088394	11088408	-	0	0	0
Simple_repeat	Simple_repeat	(A)n	chr1	41867243	41867277	+	0	0	0
SINE	Alu	B1_Mus1	chr1	41919013	41919159	-	0	0	0
SINE	Alu	B1_Mus1	chr1	42343155	42343295	-	0	0	0
Simple_repeat	Simple_repeat	(A)n	chr1	42768187	42768226	+	0	1.08938	0.342623
Simple_repeat	Simple_repeat	(A)n	chr1	42827815	42827843	+	0	0	0
LTR	MaLR	MTA_Mm	chr1	43001033	43001427	+	0	0	0
Simple_repeat	Simple_repeat	(A)n	chr10	11100530	11100533	+	0	0	0
SINE	Alu	B1_Mus1	chr1	44545194	44545341	+	0	0	0
SINE	B2	B2_Mm1a	chr1	44586160	44586352	-	0	0	0
LINE	L1	L1Md_F2	chr1	45136138	45142296	-	0	0	0
Simple_repeat	Simple_repeat	(A)n	chr1	45884874	45884910	+	0.591053	0	0
Simple_repeat	Simple_repeat	(A)n	chr1	45894464	45894513	+	0	0	0
LINE	L1	L1Md_F2	chr1	46552425	46558574	-	0	0	0
SINE	Alu	B1_Mm	chr1	46649209	46649354	-	0	0	0
SINE	B2	B2_Mm1t	chr1	46842767	46842959	+	0	0.380568	0
SINE	Alu	B1_Mus2	chr1	46970938	46971084	+	0	0	0
Simple_repeat	Simple_repeat	(A)n	chr1	46971085	46971125	+	0	0	0
SINE	Alu	B1_Mus1	chr1	47229595	47229743	-	0	0	0.342623
SINE	B2	B2_Mm1a	chr1	47543669	47543857	+	0	0	0
Simple_repeat	Simple_repeat	(A)n	chr1	47543858	47543878	+	0	0	0
Simple_repeat	Simple_repeat	(A)n	chr1	47702515	47702562	+	0	0	0
SINE	Alu	B1_Mus1	chr1	48498396	48498541	+	0	0	0
Simple_repeat	Simple_repeat	(A)n	chr1	48871131	48871184	+	0	0	0
SINE	Alu	B1_Mus1	chr1	49248345	49248492	+	0	0	0

Simple_repeat	Simple_repeat	(A)n	chr1	49248493	49248522	+	0	0	0
SINE	Alu	B1_Mus2	chr10	11148585	11148599	+	0	0	0
Simple_repeat	Simple_repeat	(A)n	chr10	11148599	11148602	+	0	0	0
LTR	MaLR	MTA_Mm	chr1	49316357	49316751	+	0	0	0
SINE	Alu	B1_Mm	chr1	49708529	49708674	-	0	0	0
LTR	MaLR	MTA_Mm	chr1	50028698	50029092	-	0.923247	0.380568	0.342623
LTR	MaLR	MTA_Mm	chr1	50030190	50030584	-	0	0	0
Simple_repeat	Simple_repeat	(A)n	chr1	50344401	50344433	+	0	0	0
LINE	L1	L1Md_F2	chr1	50493795	50499762	-	0	0	0
Simple_repeat	Simple_repeat	(A)n	chr1	50684891	50684929	+	0	0	0
Simple_repeat	Simple_repeat	(A)n	chr1	51253632	51253666	+	0	0.708814	0.342623
SINE	Alu	B1_Mus1	chr10	11167123	11167138	+	0	0	0
LTR	MaLR	MTA_Mm	chr1	52429869	52430264	-	0	0	0
SINE	Alu	B1_Mus1	chr1	52617115	52617262	-	0	0	0
SINE	Alu	B1_Mm	chr1	52895859	52896006	-	0	0	0
LTR	MaLR	MTA_Mm	chr10	11191084	11191124	-	0	0	0
SINE	Alu	B1_Mus2	chr1	53789066	53789211	+	0	0	0
Simple_repeat	Simple_repeat	(A)n	chr1	53789212	53789240	+	0	0	0
SINE	Alu	B1_Mus2	chr1	55176705	55176851	+	0	0.708814	0
Simple_repeat	Simple_repeat	(A)n	chr1	55176852	55176905	+	0	0	0
LTR	MaLR	MTA_Mm	chr1	56087064	56087458	-	0	0	0
LTR	MaLR	MTA_Mm	chr1	56088554	56088948	-	0	0	0
Simple_repeat	Simple_repeat	(A)n	chr1	57038499	57038545	+	0	0	0.342623
LTR	MaLR	MTA_Mm	chr1	57165838	57166232	-	0	0	0
Simple_repeat	Simple_repeat	(A)n	chr1	57690903	57690938	+	0.332194	0	0.342623
SINE	Alu	B1_Mus1	chr10	11232431	11232446	+	0	0	0
Simple_repeat	Simple_repeat	(A)n	chr10	11232447	11232450	+	0	0	0
SINE	Alu	PB1D9	chr1	58437743	58437857	-	0	0	0
SINE	Alu	B1_Mus1	chr1	58688443	58688589	-	0	0	0
SINE	Alu	B1_Mus2	chr1	58695278	58695422	-	0	0	0
SINE	Alu	B1_Mus1	chr1	59778547	59778688	-	0	0	0
Simple_repeat	Simple_repeat	(A)n	chr1	59785820	59785864	+	0	0	0
SINE	Alu	B1_Mm	chr1	60103686	60103828	+	0	0	0
SINE	Alu	B1_Mus2	chr1	60119095	60119241	+	0	0	0
Simple_repeat	Simple_repeat	(A)n	chr1	60119242	60119266	+	0	0	0
SINE	Alu	B1_Mus1	chr1	60210878	60211019	-	0	0	0
SINE	Alu	B1_Mus1	chr1	60234634	60234780	+	0	0.708814	0.342623
Simple_repeat	Simple_repeat	(A)n	chr1	60234781	60234833	+	0	0	0.342623
Simple_repeat	Simple_repeat	(A)n	chr1	61573288	61573325	+	0	0.380568	0
SINE	Alu	B1_Mus1	chr1	63379270	63379417	+	0	0	0
Simple_repeat	Simple_repeat	(A)n	chr1	63379422	63379442	+	0	0	0
SINE	Alu	B1_Mm	chr1	64463532	64463678	-	0	0	0
Simple_repeat	Simple_repeat	(A)n	chr1	64574900	64574949	+	0	0	0
SINE	Alu	B1_Mus2	chr1	64676923	64677068	+	0	0.380568	0
SINE	Alu	B1_Mm	chr1	65004753	65004921	+	0	0	0
SINE	Alu	B1_Mm	chr1	65005722	65005867	-	0	0	0
SINE	Alu	B1_Mm	chr1	65018166	65018313	-	0	0	0
Simple_repeat	Simple_repeat	(A)n	chr10	11309956	11309961	+	0	0	0
SINE	Alu	B1_Mus2	chr10	11312522	11312537	+	0	0	0
Simple_repeat	Simple_repeat	(A)n	chr10	11312537	11312541	+	0	0	0.342623
LTR	MaLR	MTA_Mm	chr1	65989316	65989697	-	0	0	0
SINE	Alu	B1_Mus1	chr1	67479764	67479910	-	0	0	0
LINE	L1	Lx	chr1	68034051	68039844	-	0	0	0
Simple_repeat	Simple_repeat	(A)n	chr10	11343793	11343797	+	0	0	0
Simple_repeat	Simple_repeat	(A)n	chr10	11358143	11358147	+	0	0	0.342623
Simple_repeat	Simple_repeat	(A)n	chr1	71582665	71582702	+	0	0	0
Simple_repeat	Simple_repeat	(A)n	chr1	71586102	71586135	+	0	0	0
Simple_repeat	Simple_repeat	(A)n	chr1	71774368	71774425	+	0	0	0
Simple_repeat	Simple_repeat	(A)n	chr1	72473394	72473422	+	0	0	0
SINE	B2	B2_Mm2	chr1	72750873	72751064	+	0	0	0
SINE	Alu	B1_Mus2	chr1	72847535	72847681	+	0	0	0
Simple_repeat	Simple_repeat	(A)n	chr1	72847682	72847716	+	0	0	0
SINE	Alu	B1_Mus1	chr1	74231092	74231205	+	0	0	0
Simple_repeat	Simple_repeat	(A)n	chr1	74231206	74231229	+	0	0	0
SINE	Alu	B1_Mus2	chr1	74870171	74870315	+	0	0	0
Simple_repeat	Simple_repeat	(A)n	chr1	74870322	74870350	+	0	0	0
LTR	MaLR	MTA_Mm	chr1	75268033	75268429	+	0	0.708814	0.342623

SINE	Alu	B1_Mus2	chr1	75647256	75647392	-	0	0	0
LTR	MaLR	MTA_Mm	chr1	75834615	75835010	-	0	0	0
LTR	MaLR	MTA_Mm	chr1	75888599	75888984	-	0	0	0
Simple_repeat	Simple_repeat	(A)n	chr10	14665430	14665456	+	0	0	0
Simple_repeat	Simple_repeat	(A)n	chr1	75920600	75920637	+	0	0.380568	0
LINE	L1	L1Md_F3	chr1	76375361	76381361	-	0	0	0
LINE	L1	L1VL4	chr1	76513567	76516547	-	0	0	0
SINE	Alu	B1_Mus2	chr1	76838671	76838803	-	0	0	0.615324
Simple_repeat	Simple_repeat	(A)n	chr1	77040601	77040639	+	0	0	0
LTR	MaLR	MTB	chr1	77092600	77092996	+	0	0	0
Simple_repeat	Simple_repeat	(A)n	chr10	11441380	11441383	+	0	0	0
LTR	MaLR	MTA_Mm	chr1	77842130	77842535	+	0	0	0
Simple_repeat	Simple_repeat	(A)n	chr1	77999505	77999543	+	0	0	0
SINE	Alu	B1_Mm	chr1	78245116	78245261	+	0	0	0
Simple_repeat	Simple_repeat	(A)n	chr1	78245262	78245287	+	0	0	0
LTR	MaLR	MTB	chr1	78537264	78537637	+	0	0	0
SINE	Alu	B1_Mm	chr1	78548664	78548810	+	0	0	0
SINE	Alu	B1_Mus1	chr1	78604597	78604724	-	0	0	0
SINE	Alu	B1_Mm	chr10	11451049	11451063	+	0	0	0
Simple_repeat	Simple_repeat	(A)n	chr10	11451064	11451068	+	0	0	0
SINE	Alu	B1_Mm	chr1	78630435	78630581	-	0	0	0
LTR	MaLR	MTA_Mm	chr1	79544362	79544746	+	0.332194	0	0
SINE	Alu	B1_Mus2	chr1	79695462	79695597	+	0	0	0
Simple_repeat	Simple_repeat	(A)n	chr1	79695598	79695626	+	0	0	0
SINE	Alu	B1_Mus1	chr1	80368926	80369071	-	0	0	0
Simple_repeat	Simple_repeat	(A)n	chr1	80371992	80372031	+	0	0	0
Simple_repeat	Simple_repeat	(A)n	chr1	80759732	80759770	+	0	0	0
SINE	Alu	B1_Mur4	chr1	80885014	80885160	-	0	0	0
Simple_repeat	Simple_repeat	(A)n	chr1	81022049	81022082	+	0	0	0
SINE	Alu	B1_Mus2	chr1	81037805	81037949	+	0	0	0
SINE	Alu	B1_Mm	chr1	81802938	81803085	-	0	0	0
Simple_repeat	Simple_repeat	(A)n	chr1	81845800	81845829	+	0	0	0
SINE	Alu	B1_Mus1	chr1	82771555	82771702	-	0	0	0
SINE	Alu	B1_Mus2	chr1	82815080	82815225	+	0	0	0
Simple_repeat	Simple_repeat	(A)n	chr1	82815226	82815247	+	0	0	0
LINE	L1	L1Md_T	chr1	83507613	83513485	-	0.332194	0	0.342623
SINE	Alu	B1_Mus2	chr10	11488503	11488518	-	0	0	0
SINE	Alu	B1_Mus2	chr1	84286579	84286725	+	0	0	0
Simple_repeat	Simple_repeat	(A)n	chr1	84286726	84286754	+	0	0	0
SINE	Alu	B1_Mus2	chr1	85164942	85165087	-	0	0	0
Simple_repeat	Simple_repeat	(A)n	chr1	85329392	85329429	+	1.25544	1.41763	0.342623
SINE	Alu	B1_Mus2	chr1	87392031	87392176	-	0	0	0
SINE	Alu	B1_Mus1	chr1	88328710	88328856	-	0	0	0
SINE	Alu	B1_Mus1	chr1	88342228	88342376	+	0	0	0
Simple_repeat	Simple_repeat	(A)n	chr1	88342377	88342403	+	0.591053	0	0
SINE	Alu	B1_Mus1	chr1	88406560	88406707	+	0	0	0.615324
Low_complexity	Low_complexity	GA-rich	chr1	88406708	88406784	+	0	0	0.615324
SINE	Alu	PB1D9	chr1	88417721	88417826	-	0	0	0
SINE	Alu	B1_Mus2	chr1	89217411	89217557	-	0	0	0
LTR	MaLR	MTA_Mm	chr1	89513727	89514121	+	0	0	0.615324
LTR	MaLR	MTA_Mm	chr1	89515215	89515609	+	0	0	0.685246
LTR	MaLR	MTA_Mm	chr1	89948657	89949051	+	0	0	0
SINE	Alu	B1_Mm	chr1	90958757	90958903	-	0	0	0
SINE	Alu	B1_Mus2	chr1	92316637	92316783	-	0	0	0
SINE	Alu	B1_Mm	chr1	93144220	93144378	-	0	0	0.342623
SINE	Alu	B1_Mm	chr1	93357799	93357945	-	0	0	0
Simple_repeat	Simple_repeat	(A)n	chr1	94092247	94092284	+	0	0	0
SINE	Alu	B1_Mus1	chr1	94329757	94329903	-	0	0	0
SINE	Alu	B1_Mus1	chr1	94330038	94330185	+	0	0	0
Simple_repeat	Simple_repeat	(A)n	chr1	94330186	94330212	+	0	0	0
SINE	Alu	B1_Mur4	chr1	94869199	94869348	-	0	0	0
SINE	Alu	B1_Mus1	chr1	95551990	95552137	-	0	0	0
SINE	Alu	PB1D7	chr1	95576725	95576831	-	0	0	0
Simple_repeat	Simple_repeat	(A)n	chr1	95787570	95787604	+	0	0	0
Simple_repeat	Simple_repeat	(A)n	chr1	96496164	96496219	+	0	0	0
SINE	Alu	B1_Mus2	chr1	96700005	96700150	+	0	0	0
Simple_repeat	Simple_repeat	(A)n	chr1	96700151	96700191	+	0	0	0

LINE	L1	L1Md_F	chr1	97242919	97247255	+	0	0	0.342623
Simple_repeat	Simple_repeat	(A)n	chr1	97360974	97361013	+	0	0	0
LINE	L1	L1Md_T	chr1	97407974	97414683	-	0	0	0
SINE	Alu	B1_Mus1	chr1	97978262	97978402	-	0	0	0
Simple_repeat	Simple_repeat	(A)n	chr1	99476851	99476885	+	0.332194	0	0
Simple_repeat	Simple_repeat	(A)n	chr1	10120414	10120420	+	0	0	0
SINE	Alu	B1_Mus2	chr1	10121164	10121179	-	0	0	0
LINE	L1	L1Md_F	chr1	10126110	10126658	-	0	0	0
Simple_repeat	Simple_repeat	(A)n	chr1	10163186	10163192	+	0	0	0
Simple_repeat	Simple_repeat	(A)n	chr1	10253237	10253241	+	0	0	0.342623
SINE	Alu	B1_Mm	chr1	10280856	10280871	+	0	0	0
Simple_repeat	Simple_repeat	(A)n	chr1	10280871	10280873	+	0	0	0
Simple_repeat	Simple_repeat	(A)n	chr1	10291367	10291376	+	0	0	0
Simple_repeat	Simple_repeat	(A)n	chr1	10292913	10292918	+	0	0	0
SINE	Alu	B1_Mm	chr1	10315447	10315462	+	0	0	0
Simple_repeat	Simple_repeat	(A)n	chr1	10315462	10315464	+	0	0	0
LINE	L1	L1Md_F2	chr1	10393977	10394614	-	0	0	0
SINE	Alu	B1_Mus1	chr1	10396751	10396766	+	0	0	0
Simple_repeat	Simple_repeat	(A)n	chr1	10396766	10396772	+	0	0	0
LINE	L1	L1Md_F3	chr1	10397622	10398130	+	0	0	0.615324
Simple_repeat	Simple_repeat	(A)n	chr1	10478708	10478717	+	0	0	0
SINE	Alu	B1_Mus1	chr1	10481710	10481724	+	0	0	0
Simple_repeat	Simple_repeat	(A)n	chr1	10481724	10481730	+	0	0	0
LTR	MaLR	MTA_Mm	chr10	11662319	11662358	+	0	0	0
Simple_repeat	Simple_repeat	(A)n	chr1	10509100	10509105	+	0	0	0
Simple_repeat	Simple_repeat	(A)n	chr1	10523012	10523015	+	0	0	0
SINE	Alu	B1_Mus1	chr10	11664588	11664601	+	0.332194	0	0
Simple_repeat	Simple_repeat	(A)n	chr10	11664601	11664604	+	0	0	0
SINE	Alu	B1_Mus1	chr1	10564359	10564374	-	0	0	0
SINE	B2	B2_Mm1t	chr10	11675181	11675198	-	0	0	0
SINE	Alu	B1_Mus2	chr10	11675201	11675215	-	0	0	0
SINE	Alu	B1_Mus1	chr1	10708863	10708876	-	0	0	0
Simple_repeat	Simple_repeat	(A)n	chr1	10714107	10714111	+	0	0	0
SINE	Alu	B1_Mus2	chr10	11688358	11688372	-	0	0	0
scRNA	scRNA	4.5SRNA	chr1	10890147	10890156	-	0	1.01984	0
SINE	Alu	B1_Mus2	chr1	10909839	10909854	-	0	0	0
LINE	L1	L1Md_T	chr1	10909929	10910613	-	0	0	0
Simple_repeat	Simple_repeat	(A)n	chr1	10930568	10930572	+	0	0	0
Simple_repeat	Simple_repeat	(A)n	chr1	10973940	10973944	+	0	0	0
LTR	MaLR	MTB	chr10	11694408	11694447	-	0	0	0
Simple_repeat	Simple_repeat	(A)n	chr1	10995290	10995293	+	0	0	0
LINE	L1	L1Md_F2	chr1	11029331	11029905	-	0	0	0
SINE	Alu	B1_Mm	chr1	11060952	11060965	-	0	0	0
Simple_repeat	Simple_repeat	(A)n	chr1	11085281	11085283	+	0	0	0
Simple_repeat	Simple_repeat	(A)n	chr1	11109373	11109377	+	0	0	0
SINE	Alu	B1_Mus2	chr1	11132066	11132081	+	0	0	0
Simple_repeat	Simple_repeat	(A)n	chr1	11132081	11132083	+	0	0	0
LTR	MaLR	MTA_Mm	chr1	11143826	11143866	-	0	0	0
LTR	MaLR	MTA_Mm	chr1	11144024	11144063	-	0	0	0
Simple_repeat	Simple_repeat	(A)n	chr1	11205179	11205186	+	0	0	0
SINE	Alu	B1_Mus1	chr1	11223710	11223724	+	0	0	0
Simple_repeat	Simple_repeat	(A)n	chr1	11238147	11238150	+	0	0	0
SINE	Alu	B1_Mus2	chr1	11295658	11295673	-	0	0	0
SINE	Alu	B1_Mm	chr1	11297351	11297365	-	0	0	0
SINE	Alu	B1_Mus1	chr1	11304918	11304933	-	0	0	0
Simple_repeat	Simple_repeat	(A)n	chr1	11322190	11322197	+	0	0	0
Simple_repeat	Simple_repeat	(GAAA)n	chr1	11323865	11323873	+	0	0	0
LINE	L1	L1_Mus1	chr1	11325896	11326511	-	0	0	0
SINE	Alu	B1_Mus2	chr10	11714975	11714989	+	0	0	0
Simple_repeat	Simple_repeat	(A)n	chr1	11334748	11334753	+	0	0	0
LINE	L1	L1Md_A	chr1	11335729	11336255	+	0	0	0
SINE	Alu	B1_Mus2	chr10	11715763	11715777	-	0	0	0
SINE	Alu	B1_Mm	chr10	11715923	11715938	+	0.332194	0	0
Simple_repeat	Simple_repeat	(A)n	chr10	11715938	11715940	+	0	0	0
SINE	Alu	B1_Mus2	chr1	11391113	11391127	-	0	0	0
SINE	Alu	B1_Mm	chr1	11396307	11396321	-	0	0	0
Simple_repeat	Simple_repeat	(A)n	chr1	11485948	11485954	+	0	0	0

LINE	L1	L1_Mus2	chr1	11488659	11489231	+	0	0	0
LTR	MaLR	MTA_Mm	chr1	11539049	11539089	-	0	0	0
Simple_repeat	Simple_repeat	(A)n	chr1	11576142	11576145	+	0	0	0
LINE	L1	L1Md_F3	chr1	11628967	11629584	-	0	0	0
Simple_repeat	Simple_repeat	(A)n	chr1	11637803	11637809	+	0	0	0
LTR	ERVL	RMER15	chr1	11641217	11641273	+	0	0	0
SINE	Alu	B1_Mus2	chr1	11654815	11654829	+	0	0	0
Simple_repeat	Simple_repeat	(A)n	chr1	11654829	11654833	+	0.332194	0	0
SINE	Alu	B1_Mus1	chr1	11671479	11671493	+	0	0	0
Simple_repeat	Simple_repeat	(A)n	chr1	11671493	11671496	+	0	0	0
SINE	Alu	B1_Mus2	chr1	11679012	11679026	+	0	0	0
Simple_repeat	Simple_repeat	(A)n	chr1	11679026	11679029	+	0	0	0
SINE	Alu	B1_Mus1	chr1	11700889	11700904	-	0	0	0
LINE	L1	Lx	chr1	11745692	11746279	+	0	0	0
Simple_repeat	Simple_repeat	(TTTA)n	chr1	11755369	11755372	+	0	0	0
LTR	MaLR	MTB	chr10	11748531	11748571	-	0	0	0
LTR	MaLR	MTA_Mm	chr1	11848686	11848725	+	0	0	0
SINE	Alu	B1_Mus2	chr1	11864350	11864365	+	0.591053	0.380568	0
Simple_repeat	Simple_repeat	(A)n	chr1	11864365	11864367	+	0.591053	0.380568	0
LTR	MaLR	MTA_Mm	chr1	11870243	11870283	+	0	0	0
SINE	Alu	B1_Mus2	chr1	11872334	11872349	+	0	0.708814	0
Simple_repeat	Simple_repeat	(A)n	chr1	11884649	11884652	+	0	0	0
LINE	L1	L1_Mus1	chr1	11979934	11980445	+	0	0	0
SINE	Alu	B1_Mm	chr1	12051677	12051690	-	0	0	0
Simple_repeat	Simple_repeat	(A)n	chr1	12130500	12130503	+	0	0	0
Simple_repeat	Simple_repeat	(A)n	chr1	12234005	12234009	+	0	0	0
Simple_repeat	Simple_repeat	(A)n	chr1	12332727	12332729	+	0.591053	0	0
LINE	L1	L1Md_F2	chr1	12362083	12362613	+	0	0	0
SINE	Alu	B1_Mm	chr1	12385738	12385751	+	0	0	0.342623
Simple_repeat	Simple_repeat	(A)n	chr1	12385751	12385757	+	0	0	0
SINE	Alu	B1_Mm	chr1	12394364	12394379	-	0	0	0
SINE	Alu	B1_Mus1	chr1	12423698	12423709	+	0	0	0
Simple_repeat	Simple_repeat	(GAAAA)n	chr1	12423709	12423718	+	0	0	0
SINE	Alu	B1_Mm	chr1	12440453	12440468	-	0	0	0
SINE	Alu	B1_Mus2	chr10	11827377	11827391	-	0	0	0
Simple_repeat	Simple_repeat	(A)n	chr1	12449961	12449966	+	0	0	0
LTR	MaLR	MTA_Mm	chr1	12450786	12450825	+	0	0	0.615324
Simple_repeat	Simple_repeat	(A)n	chr1	12595714	12595717	+	0	0	0
SINE	Alu	B1_Mus1	chr1	12680749	12680763	+	0	0	0
SINE	Alu	B1_Mus2	chr1	12739253	12739268	+	0	0	0
Simple_repeat	Simple_repeat	(A)n	chr1	12739268	12739272	+	0	0	0.342623
LTR	MaLR	MTB_Mm	chr10	11868676	11868717	+	0	0	0
LTR	MaLR	MTB	chr10	11873989	11874028	-	0	0	0
LTR	MaLR	MTA_Mm	chr10	4328933	4329328	-	0	0	0
Simple_repeat	Simple_repeat	(A)n	chr10	11877148	11877152	+	0	0	0
LTR	MaLR	MTA_Mm	chr1	13013734	13013773	-	0	0	0
SINE	Alu	B1_Mm	chr1	13032274	13032289	-	0	0	0
LTR	MaLR	MTA_Mm	chr1	13108106	13108145	-	0	0	0
LTR	MaLR	MTA_Mm	chr1	13108255	13108294	-	0	0	0
LTR	MaLR	MTA_Mm	chr1	13232685	13232724	+	0	0	0
SINE	Alu	B1_Mm	chr1	13384750	13384764	-	0	0	0
SINE	Alu	B1_Mus1	chr1	13388301	13388316	-	0	0	0
SINE	Alu	B1_Mus2	chr1	13397016	13397030	+	0	0	0
Simple_repeat	Simple_repeat	(A)n	chr1	13397030	13397038	+	0	0	0
Simple_repeat	Simple_repeat	(A)n	chr1	13404887	13404891	+	0	0	0
SINE	B2	B3	chr1	13538516	13538533	-	0	0	0.342623
SINE	Alu	B1_Mus2	chr1	13556225	13556239	+	0	0	0
LTR	MaLR	ORR1C2	chr1	13556241	13556273	+	0	0	0
SINE	Alu	B1_Mus2	chr1	13594823	13594838	+	0	0	0
Simple_repeat	Simple_repeat	(A)n	chr1	13594838	13594842	+	0	0	0
SINE	B2	B3A	chr1	13605945	13605962	+	0	0	0
LTR	MaLR	MTA_Mm	chr1	13606228	13606267	-	0	0	0
SINE	Alu	B1_Mm	chr1	13614407	13614421	-	0	0	0
SINE	Alu	B1_Mus2	chr1	13642916	13642931	+	0	0	0
Simple_repeat	Simple_repeat	(A)n	chr1	13642931	13642933	+	0	0	0
SINE	Alu	B1_Mus2	chr10	11973053	11973067	+	0	0	0
SINE	Alu	B1_Mus2	chr1	13708823	13708838	-	0	0	0

SINE	Alu	B1_Mm	chr1	13718184	13718198	-	0	0	0
SINE	Alu	B1_Mus2	chr10	11992208	11992222	+	0	0	0
Simple_repeat	Simple_repeat	(A)n	chr10	11992222	11992229	+	0	0	0
SINE	Alu	B1_Mm	chr10	11995171	11995185	-	0	0	0
SINE	Alu	B1_Mm	chr1	13850821	13850836	-	0	0	0
SINE	Alu	B1_Mus2	chr1	13854444	13854459	-	0	0	0
SINE	Alu	B1_Mus1	chr1	13859194	13859208	-	0	0	0
LTR	MaLR	MTA_Mm	chr1	13864657	13864695	+	0	0	0
Simple_repeat	Simple_repeat	(A)n	chr1	13864884	13864888	+	0	0	0
SINE	Alu	B1_Mus1	chr10	11998611	11998626	+	0	0	0
Simple_repeat	Simple_repeat	(A)n	chr10	11998626	11998629	+	0.332194	0	0
Simple_repeat	Simple_repeat	(A)n	chr10	12000377	12000380	+	0	0	0
LINE	L1	L1Md_T	chr1	13923543	13924208	-	0	0	0
SINE	Alu	B1_Mus2	chr10	12004878	12004892	-	0	0	0
Simple_repeat	Simple_repeat	(A)n	chr1	13951629	13951632	+	0	0	0
SINE	Alu	B1_Mus2	chr1	13983178	13983192	+	1.5143	0.708814	0.615324
Simple_repeat	Simple_repeat	(A)n	chr1	13983192	13983194	+	1.25544	0.708814	1.23065
LINE	L1	L1Md_T	chr1	14020199	14020893	-	0	0	0
SINE	Alu	B1_Mus2	chr1	14079966	14079981	-	0	0	0
LTR	MaLR	MTA_Mm	chr10	12017468	12017507	+	0	0	0
Simple_repeat	Simple_repeat	(A)n	chr10	12019818	12019823	+	0	0	0
LTR	MaLR	MTA_Mm	chr1	14146380	14146421	+	0	0	0
Simple_repeat	Simple_repeat	(A)n	chr1	14181060	14181064	+	0	0	0
Simple_repeat	Simple_repeat	(A)n	chr1	14181968	14181973	+	0	0	0
SINE	Alu	B1_Mur4	chr1	14296440	14296453	+	0	0	0
Simple_repeat	Simple_repeat	(A)n	chr1	14296453	14296456	+	0	0	0
SINE	Alu	B1_Mus2	chr1	14328887	14328901	-	0	0	0
LTR	MaLR	MTA_Mm	chr1	14364799	14364839	-	0	0	0
LTR	MaLR	MTA_Mm	chr1	14364948	14364988	-	0	0	0
SINE	Alu	B1_Mus2	chr10	12042584	12042599	+	0	0	0
Low_complexity	Low_complexity	A-rich	chr10	12042599	12042603	+	0	0	0
SINE	Alu	B1_Mus1	chr1	14395316	14395331	-	0	0	0
LINE	L1	L1MA4	chr1	14424562	14425024	-	0	0	0
SINE	Alu	B1_Mm	chr1	14472084	14472099	-	0	0	0
Simple_repeat	Simple_repeat	(A)n	chr1	14496056	14496059	+	0	0	0
LINE	L1	L1Md_F2	chr1	14513118	14513640	+	0	0	0
Simple_repeat	Simple_repeat	(A)n	chr1	14540217	14540221	+	0	0	0
SINE	Alu	B1_Mm	chr1	14607830	14607845	-	0	0	0
SINE	Alu	B1_Mm	chr1	14633201	14633215	-	0	0	0
LTR	MaLR	MTA_Mm	chr1	14655686	14655725	-	0	0	0
LTR	ERVK	MYSERV16_I-int	chr1	14663571	14663778	-	0	0	0
LTR	MaLR	MTA_Mm	chr1	14669456	14669495	+	0	0	0
LTR	MaLR	MTA_Mm	chr10	15516148	15516547	-	0	0	0
SINE	Alu	B1_Mm	chr1	14738447	14738459	-	0	0	0
LTR	MaLR	MTA_Mm	chr1	14743159	14743198	+	0	0	0.342623
LTR	MaLR	MTA_Mm	chr1	14825875	14825915	-	0	0	0.615324
LTR	MaLR	MTA_Mm	chr1	14883835	14883873	-	0	0	0
LTR	MaLR	MTA_Mm	chr10	12084294	12084333	-	0	0	0
Simple_repeat	Simple_repeat	(A)n	chr1	14973345	14973348	+	0	0	0
Simple_repeat	Simple_repeat	(A)n	chr1	14989865	14989868	+	0	0	0
LTR	MaLR	MTA_Mm	chr1	15015908	15015948	-	0	0	0
SINE	Alu	B1_Mus2	chr1	15115082	15115097	+	0	0	0
Simple_repeat	Simple_repeat	(A)n	chr1	15115097	15115106	+	0	0	0
LTR	MaLR	MTA_Mm	chr1	15115192	15115230	-	0	0	0
SINE	Alu	B1_Mus2	chr1	15120184	15120199	+	0	0	0
Simple_repeat	Simple_repeat	(A)n	chr1	15120199	15120202	+	0	0	0
Simple_repeat	Simple_repeat	(A)n	chr1	15155955	15155959	+	0	0	0
SINE	Alu	B1_Mm	chr10	12097639	12097654	+	0	0	0
Simple_repeat	Simple_repeat	(A)n	chr10	12097654	12097657	+	0	0	0
LINE	L1	L1Md_F3	chr1	15184503	15184952	+	0.332194	0	0
Simple_repeat	Simple_repeat	(A)n	chr1	15186650	15186654	+	0	0	0
Simple_repeat	Simple_repeat	(A)n	chr1	15208674	15208679	+	0	0	0
SINE	Alu	B1_Mus2	chr1	15314533	15314547	+	0	0	0
Simple_repeat	Simple_repeat	(A)n	chr1	15314547	15314550	+	0	0	0
SINE	Alu	B1_Mus2	chr1	15338097	15338111	+	0	0	0
Simple_repeat	Simple_repeat	(A)n	chr1	15338111	15338115	+	0	0	0
SINE	Alu	B1_Mus1	chr1	15423440	15423454	+	0	0	0

Simple_repeat	Simple_repeat	(A)n	chr1	15423455	15423457	+	0	0	0
SINE	Alu	B1_Mus2	chr1	15477272	15477287	-	0	0	0
SINE	Alu	B1_Mm	chr1	15518605	15518620	+	0	0	0
SINE	Alu	B1_Mus1	chr1	15521264	15521278	+	0	0	0
Simple_repeat	Simple_repeat	(A)n	chr1	15521279	15521283	+	0	0	0
SINE	Alu	B1_Mus1	chr1	15523166	15523179	-	0	0	0
SINE	Alu	B1_Mm	chr1	15534755	15534770	+	0.591053	0	0
Simple_repeat	Simple_repeat	(A)n	chr1	15534770	15534772	+	0	0	0
SINE	Alu	B1_Mm	chr1	15540089	15540103	-	0	0	0
SINE	Alu	B1_Mus1	chr1	15549467	15549482	-	0	0	0
SINE	Alu	B1_Mm	chr1	15581492	15581507	+	0	0	0
Simple_repeat	Simple_repeat	(A)n	chr1	15581507	15581509	+	0	0	0
LINE	L1	L1Md_T	chr10	15610249	15616584	-	0	0	0
SINE	Alu	B1_Mus1	chr1	15709574	15709588	-	0	0	0
SINE	Alu	B1_Mus1	chr1	15710966	15710980	-	0	0.708814	0
SINE	Alu	B1_Mm	chr1	15736029	15736044	+	0.332194	0	0
Simple_repeat	Simple_repeat	(A)n	chr1	15736044	15736047	+	0.332194	0	0
SINE	B2	B3	chr1	15736047	15736064	+	0.332194	0	0
SINE	Alu	B1_Mm	chr1	15737106	15737121	-	0	0	0
LTR	MaLR	MTA_Mm	chr1	15782503	15782543	-	0	0	0
SINE	Alu	B1_Mus1	chr1	15783568	15783582	-	0	0	0
SINE	Alu	B1_Mus2	chr1	15818059	15818073	+	0	0	0
Simple_repeat	Simple_repeat	(A)n	chr1	15818074	15818076	+	0	0	0
SINE	B2	B2_Mm1a	chr1	15845290	15845309	-	0	0	0
SINE	Alu	B1_Mus2	chr1	15848166	15848180	-	0	0	0
SINE	Alu	B1_Mm	chr1	15873126	15873141	-	0	0	0
SINE	B2	B2_Mm1a	chr1	15887296	15887315	-	0	0	0
SINE	Alu	B1_Mm	chr1	15887628	15887642	+	0	0	0
SINE	Alu	B1_Mus2	chr1	16320040	16320054	+	0	0	0
Simple_repeat	Simple_repeat	(A)n	chr1	16320054	16320056	+	0	0	0
LTR	MaLR	MTA_Mm	chr1	16348587	16348626	-	0	0	0
LTR	MaLR	MTA_Mm	chr1	16359025	16359061	+	0.332194	0	0
SINE	B2	B2_Mm1t	chr1	16381962	16381981	-	0	0	0
SINE	Alu	B1_Mus1	chr1	16384561	16384575	-	0	0	0
Simple_repeat	Simple_repeat	(A)n	chr1	16391583	16391587	+	0	0	0
LTR	MaLR	MTA_Mm	chr1	16603060	16603099	-	0	0	0
SINE	Alu	B1_Mus2	chr1	16644782	16644796	+	0	0	0
Simple_repeat	Simple_repeat	(A)n	chr1	16644797	16644799	+	0	0	0
Simple_repeat	Simple_repeat	(A)n	chr1	16650788	16650792	+	0	0.380568	0.342623
SINE	Alu	B1_Mm	chr1	16719420	16719437	-	0	0	0
SINE	Alu	B1_Mm	chr1	16826711	16826726	+	0	0	0
Simple_repeat	Simple_repeat	(A)n	chr1	16826726	16826728	+	0	0	0
SINE	Alu	B1_Mus1	chr10	12264241	12264255	+	0	0	0
Simple_repeat	Simple_repeat	(A)n	chr10	12264256	12264258	+	0	0	0
Simple_repeat	Simple_repeat	(A)n	chr1	16878946	16878949	+	0	0	0
Simple_repeat	Simple_repeat	(A)n	chr1	16908306	16908308	+	0	0	0
LTR	MaLR	MTA_Mm	chr1	16941142	16941183	+	0	0	0
SINE	Alu	B1_Mus1	chr1	17049117	17049131	+	0	0	0
SINE	Alu	B1_Mus2	chr1	17067491	17067506	-	0	0	0
LTR	MaLR	MTA_Mm	chr1	17098593	17098632	-	0	0	0
LTR	MaLR	MTA_Mm	chr1	17125178	17125217	+	0	0	0
SINE	Alu	B1_Mus1	chr1	17140339	17140353	-	0	0	0
Simple_repeat	Simple_repeat	(A)n	chr1	17224313	17224317	+	0	0	0
SINE	Alu	B1_Mm	chr1	17283154	17283168	+	0	0	0
Simple_repeat	Simple_repeat	(A)n	chr1	17283169	17283171	+	0	0	0
Simple_repeat	Simple_repeat	(A)n	chr1	17293364	17293367	+	0	0	0
Simple_repeat	Simple_repeat	(A)n	chr1	17318020	17318024	+	0	0	0.615324
SINE	Alu	B1_Mus2	chr1	17432604	17432618	-	0	0	0
Simple_repeat	Simple_repeat	(A)n	chr10	12341748	12341752	+	0	0	0
SINE	Alu	B1_Mur4	chr10	12344922	12344936	+	0	0	0
Simple_repeat	Simple_repeat	(A)n	chr10	12344936	12344941	+	0	0	0
Simple_repeat	Simple_repeat	(A)n	chr1	17607958	17607962	+	0	0	0
SINE	Alu	B1_Mus2	chr1	17697111	17697126	+	0	0	0
Simple_repeat	Simple_repeat	(A)n	chr1	17697126	17697129	+	0	0	0
Simple_repeat	Simple_repeat	(A)n	chr1	17874213	17874217	+	0	0	0.342623
SINE	Alu	B1_Mm	chr1	17926703	17926717	+	0	0	0
Simple_repeat	Simple_repeat	(A)n	chr1	17926717	17926720	+	0	0	0

SINE	Alu	B1_Mm	chr1	17926954	17926969	-	0	0	0
SINE	Alu	B1_Mus2	chr10	12383812	12383827	-	0	0	0
LINE	L1	L1Md_T	chr1	17985947	17986592	-	0	0	0
SINE	Alu	B1_Mur3	chr1	18008099	18008114	+	0	0	0
Simple_repeat	Simple_repeat	(A)n	chr1	18008114	18008118	+	0	0	0
SINE	B2	B2_Mm2	chr1	18018847	18018866	+	0	0.708814	0
SINE	Alu	B1_Mm	chr1	18022641	18022657	-	0	0	0
SINE	Alu	B1_Mus1	chr1	18027269	18027283	+	0	0	0
Simple_repeat	Simple_repeat	(A)n	chr1	18027283	18027286	+	0	0	0
Simple_repeat	Simple_repeat	(A)n	chr1	18248458	18248464	+	0	0	0
SINE	Alu	B1_Mus2	chr1	18249198	18249212	-	0	0	0
SINE	Alu	B1_Mm	chr1	18256524	18256538	-	0	0	0
Simple_repeat	Simple_repeat	(CCAA)n	chr1	18320542	18320545	+	0	0	0
Simple_repeat	Simple_repeat	(A)n	chr1	18320545	18320548	+	0	0	0
LTR	MaLR	MTB	chr1	18320558	18320596	+	0	0	0
LTR	MaLR	MTC	chr1	18325040	18325083	+	0	0	0
Simple_repeat	Simple_repeat	(A)n	chr1	18327431	18327433	+	0	0	0
SINE	Alu	B1_Mus1	chr10	12429515	12429529	+	0	0	0
Simple_repeat	Simple_repeat	(A)n	chr10	12429529	12429532	+	0	0	0
SINE	Alu	B1_Mus2	chr1	18356546	18356561	-	0	0	0
SINE	Alu	B1_Mus2	chr1	18369061	18369076	-	0	0	0
SINE	Alu	B1_Mus2	chr1	18400639	18400654	+	0	0	0
Simple_repeat	Simple_repeat	(A)n	chr1	18400654	18400656	+	0	0	0
SINE	Alu	B1_Mm	chr1	18407683	18407699	+	0	0	0
Simple_repeat	Simple_repeat	(A)n	chr1	18407699	18407701	+	0	0	0
Simple_repeat	Simple_repeat	(A)n	chr1	18410166	18410170	+	0	0	0
LTR	MaLR	MTA_Mm	chr1	18417173	18417207	+	0	0	0
SINE	Alu	B1_Mus1	chr1	18421971	18421986	-	0	0	0
LTR	MaLR	MTA_Mm	chr1	18427452	18427490	+	0	0	0
SINE	B2	B2_Mm2	chr1	18448274	18448292	-	0	0	0
LTR	MaLR	MTA_Mm	chr1	18460665	18460704	-	0	0	0
LTR	MaLR	MTB	chr1	18468697	18468735	+	0	0	0
LINE	L1	L1Md_A	chr1	18563364	18563884	+	0	0	0.615324
SINE	Alu	B1_Mm	chr1	18564024	18564039	+	0	0	0
Simple_repeat	Simple_repeat	(A)n	chr1	18564039	18564041	+	0	0	0
LTR	MaLR	MTA_Mm	chr1	18579494	18579533	-	0	0	0
SINE	Alu	B1_Mm	chr1	18582573	18582588	-	0	0	0
LTR	MaLR	MTA_Mm	chr1	18591635	18591672	-	0	0	0
scRNA	scRNA	4.5SRNA	chr1	18628971	18628980	-	0	0	0
Simple_repeat	Simple_repeat	(A)n	chr1	18643865	18643869	+	0	0	0
SINE	Alu	B1_Mus2	chr1	18649547	18649561	+	0	0	0
Simple_repeat	Simple_repeat	(A)n	chr1	18649562	18649568	+	0	0	0
SINE	Alu	B1_Mus2	chr1	18651108	18651123	-	0	0	0
Simple_repeat	Simple_repeat	(A)n	chr10	12460273	12460277	+	0	0	0
SINE	Alu	B1F1	chr1	18687420	18687435	+	0.591053	0	0
Simple_repeat	Simple_repeat	(A)n	chr1	18687435	18687437	+	0.591053	0	0
LTR	MaLR	MTA_Mm	chr1	18766752	18766791	+	0	0	0
SINE	B2	B2_Mm1t	chr1	18862658	18862676	-	0	0	0
LTR	MaLR	MTA_Mm	chr1	18930818	18930858	-	0	0	0
LTR	MaLR	MTA_Mm	chr10	12502629	12502668	+	0	0	0
LTR	MaLR	MTA_Mm	chr1	19078965	19079003	+	0	0	0
Simple_repeat	Simple_repeat	(A)n	chr1	19092659	19092662	+	0	0	0
SINE	Alu	B1_Mus2	chr1	19182386	19182400	+	0	0	0
SINE	Alu	B1_Mus1	chr1	19191874	19191889	-	0	0	0
LTR	MaLR	MTA_Mm	chr1	19245327	19245366	-	0	0	0
LTR	MaLR	MTA_Mm	chr1	19252164	19252203	+	0	0	0
LTR	MaLR	ORR1D2-int	chr1	19296447	19296586	-	0	0	0
SINE	Alu	B1_Mus2	chr1	19348887	19348901	+	0.591053	0	0
Simple_repeat	Simple_repeat	(A)n	chr1	19348901	19348904	+	0	0	0
SINE	Alu	B1_Mus2	chr1	19511831	19511845	+	0	0	0
Simple_repeat	Simple_repeat	(A)n	chr1	19511845	19511848	+	0	0	0
Simple_repeat	Simple_repeat	(A)n	chr1	19542514	19542519	+	0	0	0
Simple_repeat	Simple_repeat	(A)n	chr1	19591308	19591311	+	0	0	0
SINE	B2	B2_Mm1t	chr1	19636896	19636915	+	0	0	0
Simple_repeat	Simple_repeat	(A)n	chr1	19636915	19636919	+	0	0.380568	0
SINE	Alu	B1_Mus2	chr2	3174406	3174524	+	0	0	0
Simple_repeat	Simple_repeat	(A)n	chr2	3174525	3174560	+	0	0	0

SINE	Alu	B1_Mus1	chr2	4910347	4910485	-	0	0	0
SINE	Alu	B1_Mus2	chr2	4941248	4941393	+	0	0	0
Simple_repeat	Simple_repeat	(A)n	chr2	4941394	4941420	+	0	0	0
SINE	Alu	B1_Mus2	chr2	5188456	5188602	-	0	0.761137	0
SINE	ID	ID4_	chr2	5639734	5639813	-	0	0	0
SINE	Alu	B1_Mm	chr10	12605613	12605628	-	0	0	0
LTR	MaLR	MTB	chr10	12613028	12613067	-	0	0	0
LINE	L1	L1Md_F	chr2	7668777	7674169	+	0	0	0
LTR	MaLR	MTA_Mm	chr2	8019490	8019896	+	0	0	0.342623
LTR	MaLR	MTA_Mm	chr2	8094306	8094695	-	0	0	0
LINE	L1	L1Md_T	chr2	8104924	8112296	-	0	0	0
Simple_repeat	Simple_repeat	(A)n	chr2	8137806	8137832	+	0	0	0
Simple_repeat	Simple_repeat	(A)n	chr2	9344965	9345004	+	0	0	0
Simple_repeat	Simple_repeat	(A)n	chr2	9346563	9346599	+	0	0	0
SINE	Alu	B1_Mus2	chr2	9535307	9535432	+	0	0.380568	0
Simple_repeat	Simple_repeat	(A)n	chr2	9535433	9535457	+	0	0.380568	0
Simple_repeat	Simple_repeat	(A)n	chr2	9633930	9633967	+	0	0	0
SINE	Alu	B1_Mus1	chr2	9974454	9974601	-	0	0	0.342623
SINE	Alu	B1_Mm	chr2	10069667	10069812	-	0	0	0
SINE	Alu	B1_Mus2	chr2	10184888	10185030	+	0	0	0
Simple_repeat	Simple_repeat	(A)n	chr2	10185031	10185063	+	0	0	0
SINE	Alu	B1_Mm	chr2	10996014	10996166	-	0	0	0
Simple_repeat	Simple_repeat	(A)n	chr2	11042593	11042632	+	0	0	0
SINE	Alu	B1_Mm	chr2	11536529	11536665	-	0	0	0
LTR	MaLR	MTB	chr2	11658208	11658603	-	0.332194	0	1.10507
LTR	MaLR	MTA_Mm	chr2	12411968	12412365	-	0	0	0.342623
LTR	MaLR	MTA_Mm	chr2	12704276	12704671	-	0	0	0
LTR	MaLR	MTA_Mm	chr2	12792305	12792684	-	0	0	0
LTR	MaLR	MTA_Mm	chr2	12793772	12794151	-	0	0	0
LINE	L1	L1_Mus3	chr2	13838048	13843246	-	0	0	0
SINE	Alu	B1_Mus1	chr2	15183472	15183605	-	0	0	0
LINE	L1	L1_Mus1	chr2	15571270	15577248	-	0	0	0
SINE	Alu	B1_Mus1	chr2	15891210	15891323	-	0	0	0
LINE	L1	L1Md_F3	chr2	15987699	15993003	-	0	0	0
SINE	B2	B2_Mm1a	chr2	15993004	15993194	-	0	0	0
Simple_repeat	Simple_repeat	(A)n	chr10	12682722	12682725	+	0	0	0
LINE	L1	L1Md_T	chr2	17045803	17053054	-	0	0.708814	0.615324
SINE	Alu	B1_Mus2	chr2	17810394	17810540	+	0	0	0
Simple_repeat	Simple_repeat	(A)n	chr2	17810541	17810568	+	0	0	0
SINE	Alu	B1_Mus1	chr2	17835780	17835927	-	0	0	0
Simple_repeat	Simple_repeat	(A)n	chr2	17859018	17859044	+	0	0	0
SINE	B2	B3	chr2	17942916	17943132	+	0	0	0
Low_complexity	Low_complexity	A-rich	chr2	17943133	17943148	+	0	0	0
SINE	Alu	B1_Mus1	chr2	18489599	18489751	-	0	0	0
SINE	Alu	PB1	chr2	18570164	18570294	+	0	0	0
Simple_repeat	Simple_repeat	(A)n	chr2	18570295	18570330	+	0	0	0
LTR	MaLR	MTA_Mm	chr2	19716613	19717007	+	0	0	0.615324
LTR	MaLR	MTA_Mm	chr2	19874272	19874666	+	0	0	0.615324
SINE	Alu	B1_Mus2	chr2	20738694	20738840	-	0	0	0
Simple_repeat	Simple_repeat	(A)n	chr2	20761569	20761616	+	0.332194	0	0.615324
Simple_repeat	Simple_repeat	(A)n	chr2	22609311	22609357	+	0	0	0.342623
SINE	Alu	B1_Mm	chr2	24222450	24222582	+	0	0	0
Simple_repeat	Simple_repeat	(A)n	chr2	24421154	24421184	+	0	0	0
SINE	B2	B2_Mm1a	chr2	25112232	25112423	-	4.2745	4.08066	1.55647
SINE	Alu	B1_Mus2	chr2	25234837	25234983	-	0	0	0
Simple_repeat	Simple_repeat	(A)n	chr2	25308113	25308140	+	0	0	0
SINE	Alu	B1_Mus1	chr2	25408962	25409108	-	0	0	0
SINE	Alu	B1_Mm	chr2	25846423	25846540	-	0	0	0
SINE	Alu	B1_Mus2	chr2	26312691	26312836	-	0	0	0
SINE	Alu	B1_Mm	chr2	26480540	26480686	-	0	0	0
Simple_repeat	Simple_repeat	(A)n	chr2	26642995	26643040	+	0	0	0
SINE	Alu	B1_Mus2	chr2	26886568	26886721	-	0	0	0
SINE	Alu	B1_Mm	chr2	27366863	27367008	+	0	0	0
Simple_repeat	Simple_repeat	(A)n	chr2	27367009	27367035	+	0	0	0
Simple_repeat	Simple_repeat	(A)n	chr2	27432622	27432652	+	0	0	0.342623
Low_complexity	Low_complexity	A-rich	chr2	27471180	27471254	+	0	0	0
LTR	ERVL	MT2B	chr2	27646356	27646921	-	0	0	0

SINE	Alu	B1_Mm	chr2	27646922	27647070	-	0	0	0
SINE	B2	B2_Mm2	chr2	27648007	27648211	-	0	0	0
SINE	Alu	B1_Mm	chr2	27648212	27648358	-	0	0	0
SINE	Alu	B1_Mm	chr2	28298026	28298182	+	0	0	0
Simple_repeat	Simple_repeat	(A)n	chr2	28298183	28298208	+	0	0	0
SINE	Alu	B1_Mur4	chr2	28333744	28333875	-	0	0	0
SINE	B2	B2_Mm2	chr2	28792407	28792561	+	0	0	0
SINE	Alu	B1_Mus1	chr10	12765388	12765403	-	0	0	0
SINE	Alu	B1_Mus2	chr2	28965950	28966095	+	0	0	0
Simple_repeat	Simple_repeat	(A)n	chr2	28966096	28966126	+	0	0	0
SINE	Alu	B1_Mus2	chr2	28970577	28970722	-	0	0	0
SINE	Alu	B1_Mus2	chr2	29045436	29045582	-	0	0	0
Simple_repeat	Simple_repeat	(A)n	chr2	29049719	29049761	+	0	0	0
SINE	Alu	B1_Mus2	chr2	29987658	29987804	-	0	0	0
SINE	Alu	B1_Mus2	chr2	30036357	30036502	-	0	0	0
SINE	Alu	B1_Mus2	chr2	30072376	30072508	+	0	0	0
Simple_repeat	Simple_repeat	(A)n	chr2	30072509	30072536	+	0	0	0
Simple_repeat	Simple_repeat	(A)n	chr2	30219269	30219304	+	0	0	0
SINE	Alu	B1_Mus2	chr2	30584386	30584532	+	0	0	0
SINE	Alu	B1_Mm	chr10	12781178	12781193	-	0	0	0
SINE	Alu	B1_Mus2	chr2	31074800	31074946	+	0	0	0
Simple_repeat	Simple_repeat	(A)n	chr2	31074947	31074970	+	0	0	0
LTR	MaLR	MTA_Mm	chr2	31181852	31182247	-	0	0	0
SINE	Alu	B1_Mus2	chr2	31407169	31407315	-	0	0	0
SINE	Alu	PB1D9	chr2	31484280	31484427	+	0	0	0
Simple_repeat	Simple_repeat	(A)n	chr2	31540855	31540898	+	0	0	0
SINE	Alu	B1_Mus2	chr2	31985852	31985999	-	0	0	0
SINE	Alu	B1_Mm	chr2	32131259	32131405	+	0	0	0
Simple_repeat	Simple_repeat	(A)n	chr2	32131406	32131462	+	0	0	0.342623
LTR	MaLR	MTB_Mm	chr2	32260946	32261348	+	0	0	0
SINE	Alu	B1_Mus2	chr2	32314881	32315027	+	0	0	0
SINE	Alu	B1_Mus2	chr2	32339271	32339417	-	0	0	0
SINE	Alu	B1_Mus2	chr2	32952829	32952975	-	0	0.708814	0
SINE	Alu	B1_Mus2	chr2	33329746	33329892	-	0	0	0
SINE	Alu	B1_Mm	chr10	12799254	12799266	+	0	0	0
Simple_repeat	Simple_repeat	(A)n	chr10	12799266	12799268	+	0.332194	0	0
SINE	Alu	B1_Mus2	chr10	12803614	12803629	-	0	0	0
SINE	Alu	B1_Mus2	chr2	34270572	34270718	+	0	0	0
SINE	B2	B2_Mm1a	chr10	12805311	12805330	-	0	0	0
SINE	Alu	B1_Mus1	chr2	34530110	34530221	-	0.591053	0	0.615324
SINE	Alu	B1_Mus2	chr2	34682611	34682757	-	0	0	0
SINE	Alu	B1_Mus2	chr2	34704026	34704171	-	0	0	0
SINE	Alu	B1_Mus2	chr2	35361679	35361824	-	0	0	0
SINE	Alu	B1_Mus2	chr10	12813408	12813422	+	0	0	0
Simple_repeat	Simple_repeat	(A)n	chr10	12813422	12813425	+	0	0	0
LINE	L1	L1Md_F2	chr2	36318816	36324107	+	0	0	0
LTR	MaLR	MTA_Mm	chr2	36583643	36584036	-	0	0	0
LTR	MaLR	MTA_Mm	chr2	36585133	36585528	-	0	0	0
Simple_repeat	Simple_repeat	(A)n	chr2	36727353	36727380	+	0	0	0
LTR	MaLR	MTA_Mm	chr2	36944566	36944960	-	0	0	0.615324
Simple_repeat	Simple_repeat	(A)n	chr2	37045703	37045754	+	0	0	0
SINE	Alu	B1_Mm	chr2	37590106	37590244	-	0	0	0
SINE	B2	B2_Mm1t	chr2	38190104	38190296	-	0	0	0
LTR	MaLR	MTA_Mm	chr2	38522241	38522637	+	0	0	0
SINE	Alu	B1_Mm	chr2	38849302	38849447	-	0	0	0
LINE	L1	L1Md_T	chr2	39096000	39102538	-	0	0	0
LTR	MaLR	MTA_Mm	chr2	39592935	39593314	-	0	0	0
SINE	Alu	B1_Mus1	chr2	39771964	39772109	-	0	0	0
LINE	L1	L1_Mus1	chr2	40268705	40274952	+	0	0	0
SINE	Alu	B1_Mm	chr2	40290357	40290503	-	0	0	0
SINE	Alu	B1_Mus2	chr2	40308786	40308932	+	0	0	0
Simple_repeat	Simple_repeat	(A)n	chr2	42733048	42733126	+	0	0	0
SINE	Alu	B1_Mus2	chr2	42954132	42954278	-	0	0	0
Simple_repeat	Simple_repeat	(A)n	chr2	43052043	43052082	+	0	0	0.342623
Low_complexity	Low_complexity	A-rich	chr2	44364201	44364253	+	0	0	0
SINE	Alu	B1_Mm	chr2	44773401	44773546	-	0.591053	3.42939	3.41924
Simple_repeat	Simple_repeat	(A)n	chr2	45168811	45168837	+	0	0	0.615324

Simple_repeat	Simple_repeat	(A)n	chr2	45245759	45245799	+	0	0	0
SINE	Alu	B1_Mus2	chr2	45583728	45583873	-	0	0	0
LTR	MaLR	MTA_Mm	chr2	45662432	45662827	+	0	0	0
Simple_repeat	Simple_repeat	(A)n	chr2	45960948	45960987	+	0	0	0
LINE	L1	Lx	chr2	46065352	46071469	+	0	0	0
SINE	Alu	B1_Mus2	chr2	46258723	46258869	-	0	0	0
Simple_repeat	Simple_repeat	(A)n	chr2	46438228	46438291	+	0	0	0
LTR	MaLR	MTA_Mm	chr2	46644108	46644502	+	0	0	0
LINE	L1	L1Md_T	chr2	46978311	46984999	-	0	0	0
Simple_repeat	Simple_repeat	(A)n	chr2	47444296	47444372	+	0	0	0
SINE	Alu	B1_Mus2	chr2	47900533	47900678	-	0	0	0
Simple_repeat	Simple_repeat	(A)n	chr2	48183114	48183141	+	0	0	0
LTR	MaLR	MTA_Mm	chr2	48207153	48207547	-	0	0	0.615324
Simple_repeat	Simple_repeat	(A)n	chr2	48253409	48253447	+	0	0	0
Simple_repeat	Simple_repeat	(A)n	chr10	12915602	12915607	+	0	0	0
Simple_repeat	Simple_repeat	(A)n	chr2	48269214	48269251	+	0	0	0
SINE	Alu	B1_Mus2	chr2	48285677	48285791	+	0	0	0
LTR	MaLR	MTA_Mm	chr2	49852366	49852761	+	0	0	0
LINE	L1	L1_Mus2	chr2	50093544	50098384	+	0	0	0
SINE	Alu	B1_Mus2	chr2	50859728	50859870	+	0	0	0
Simple_repeat	Simple_repeat	(A)n	chr2	50859871	50859916	+	0	0	0
LTR	MaLR	MTA_Mm	chr2	51650234	51650632	+	0	0	0
SINE	Alu	B1F2	chr2	52708737	52708874	+	0	0	0
Simple_repeat	Simple_repeat	(A)n	chr2	52708875	52708898	+	0	0	0
SINE	Alu	B1_Mm	chr10	12970117	12970131	-	0	0	0
SINE	Alu	B1_Mm	chr2	53803224	53803370	+	0	0	0
Simple_repeat	Simple_repeat	(A)n	chr2	53803371	53803394	+	0	0	0
LINE	L1	L1Md_Gf	chr10	12974136	12974622	+	0	0	0
Simple_repeat	Simple_repeat	(A)n	chr2	54279650	54279684	+	0	0	0
SINE	Alu	B1_Mus2	chr2	55522730	55522860	-	0	0	0
Simple_repeat	Simple_repeat	(A)n	chr2	55800233	55800279	+	0	0	0.342623
LINE	L1	L1Md_A	chr2	55902154	55908318	-	0	0	0
LINE	L1	L1Md_F	chr2	56195494	56200714	+	0	0	0
Simple_repeat	Simple_repeat	(A)n	chr2	56372732	56372772	+	0	0	0
Simple_repeat	Simple_repeat	(TTTA)n	chr10	12997844	12997848	+	0	0	0
Simple_repeat	Simple_repeat	(A)n	chr2	56944017	56944051	+	0	0	0
Simple_repeat	Simple_repeat	(A)n	chr2	58115573	58115614	+	0	0	0
SINE	Alu	B1_Mus2	chr2	59206251	59206397	-	0	0	0
SINE	Alu	B1_Mus1	chr11	3148936	3149084	+	0	0	0
Simple_repeat	Simple_repeat	(A)n	chr11	3149085	3149108	+	0	0	0
LTR	MaLR	MTB	chr2	59444099	59444506	-	0	0	0
SINE	Alu	B1_Mm	chr2	60013074	60013219	+	0	0	0
Simple_repeat	Simple_repeat	(A)n	chr2	60013220	60013247	+	0.664388	0	0
SINE	Alu	B1_Mm	chr2	60035799	60035945	-	1.77316	0	0
Simple_repeat	Simple_repeat	(A)n	chr2	60975299	60975334	+	0	0	0
Simple_repeat	Simple_repeat	(A)n	chr2	61065448	61065487	+	0	0	0
Simple_repeat	Simple_repeat	(A)n	chr2	61108608	61108690	+	0.332194	0	0
Simple_repeat	Simple_repeat	(A)n	chr2	61495503	61495539	+	0	0	0
SINE	Alu	B1_Mus2	chr2	61760922	61761066	-	0	0	0
LTR	MaLR	MTA_Mm	chr2	61859480	61859875	+	0	0.708814	0
Simple_repeat	Simple_repeat	(A)n	chr2	64359226	64359254	+	0	0	0
SINE	Alu	B1_Mus1	chr11	3451341	3451487	+	0	0	0
SINE	Alu	B1_Mus1	chr2	64593170	64593319	+	0	0	0
Simple_repeat	Simple_repeat	(A)n	chr2	64593320	64593348	+	0	0	0
SINE	Alu	B1_Mus1	chr2	64876634	64876773	-	0	0	0
SINE	Alu	B1_Mus2	chr11	3487094	3487238	+	0	0	0
SINE	Alu	B1_Mus2	chr2	65152663	65152809	-	0.591053	0	1.23065
Simple_repeat	Simple_repeat	(GAAAA)n	chr11	3487239	3487296	+	0	0	0
SINE	Alu	B1_Mus2	chr11	3514977	3515112	-	0	0	0
Simple_repeat	Simple_repeat	(A)n	chr2	65986615	65986653	+	0	0	0
SINE	Alu	B1_Mus1	chr2	65991120	65991267	-	0	0	0
SINE	Alu	B1_Mus1	chr2	69045129	69045267	+	0	0	0
Simple_repeat	Simple_repeat	(A)n	chr2	69045268	69045317	+	0	0	0
SINE	B2	B2_Mm1a	chr2	69675563	69675750	-	1.8711	0	1.10507
SINE	Alu	B1_Mm	chr2	72117938	72118084	-	0	0	0
SINE	Alu	B1_Mm	chr2	72456529	72456669	-	0	0	0
Simple_repeat	Simple_repeat	(A)n	chr2	72515787	72515829	+	0	0	0.342623

SINE	Alu	B1_Mus1	chr2	72891845	72891982	+	0	0	0
Simple_repeat	Simple_repeat	(A)n	chr2	72891983	72892008	+	0	0	0
Simple_repeat	Simple_repeat	(A)n	chr2	73104912	73104955	+	0	0.380568	0
Simple_repeat	Simple_repeat	(A)n	chr2	73873861	73873937	+	0	0	0
SINE	Alu	B1_Mm	chr2	74022372	74022519	+	0	0	0
Simple_repeat	Simple_repeat	(A)n	chr2	74022520	74022599	+	0	0.380568	0.342623
Simple_repeat	Simple_repeat	(A)n	chr2	74987483	74987553	+	0	0	0
LINE	L1	L1Md_T	chr2	75064941	75071646	-	0	0	0
SINE	Alu	B1_Mus1	chr2	75457827	75457974	+	0	0	0
Simple_repeat	Simple_repeat	(A)n	chr2	75457975	75458000	+	0	0	0
SINE	Alu	B1_Mm	chr2	75509888	75510034	+	0	0.380568	0
Simple_repeat	Simple_repeat	(A)n	chr2	75510035	75510060	+	0	0.380568	0
SINE	B2	B2_Mm2	chr2	75616894	75617078	-	0	0	0
SINE	Alu	B1_Mus1	chr2	76207358	76207505	-	0	0	0
Simple_repeat	Simple_repeat	(A)n	chr11	4502051	4502085	+	0	0	0
Simple_repeat	Simple_repeat	(A)n	chr2	78179694	78179734	+	0	0	0
Simple_repeat	Simple_repeat	(A)n	chr2	78351205	78351235	+	0	0	0
Simple_repeat	Simple_repeat	(A)n	chr2	78706556	78706578	+	0	0	0
SINE	Alu	B1_Mur3	chr2	78848527	78848636	-	0	0	0
Simple_repeat	Simple_repeat	(A)n	chr2	79272503	79272538	+	0	0	0
Simple_repeat	Simple_repeat	(A)n	chr2	79523881	79523928	+	0	0	0
SINE	Alu	B1_Mus1	chr11	4598527	4598675	+	0	0	0
Simple_repeat	Simple_repeat	(A)n	chr11	4598676	4598699	+	0	0	0
Simple_repeat	Simple_repeat	(A)n	chr2	80339974	80340002	+	0	0	0
SINE	Alu	B1_Mus1	chr2	80524282	80524419	-	0	0	0
SINE	Alu	B1_Mus1	chr2	80596000	80596147	-	0	0	0
Simple_repeat	Simple_repeat	(A)n	chr2	81278827	81278879	+	0	0	0
LINE	L1	L1Md_T	chr2	82329221	82336204	-	0	0	0
SINE	Alu	B1_Mus1	chr11	4753427	4753572	+	0	0	0
Simple_repeat	Simple_repeat	(A)n	chr11	4753573	4753596	+	0	0	0
Simple_repeat	Simple_repeat	(A)n	chr11	4760996	4761040	+	0	0	0
LTR	MaLR	MTB	chr2	83536245	83536646	+	0	0	0
LTR	MaLR	MTA_Mm	chr2	83842446	83842829	+	0.332194	0	0
LTR	MaLR	MTA_Mm	chr2	83981174	83981569	+	0	0	0
LTR	MaLR	MTA_Mm	chr2	84005610	84006005	-	0	0	0
SINE	B2	B2_Mm1a	chr2	84421684	84421875	-	0	0	0.615324
SINE	Alu	B1_Mus1	chr2	84424716	84424863	-	0	0	0
SINE	B2	B2_Mm1a	chr2	84673658	84673847	-	0	0	0
SINE	Alu	B1_Mm	chr2	85124221	85124364	+	0	0	0.615324
Simple_repeat	Simple_repeat	(A)n	chr2	85124365	85124416	+	0.332194	0	0.615324
Simple_repeat	Simple_repeat	(A)n	chr2	85406802	85406824	+	0	0	0
SINE	Alu	B1_Mus2	chr2	86047210	86047348	-	0	0	0
SINE	Alu	B1_Mus1	chr2	86198269	86198415	+	0	0	0
Simple_repeat	Simple_repeat	(A)n	chr2	86198416	86198460	+	0	0	0
Simple_repeat	Simple_repeat	(A)n	chr2	86261295	86261330	+	0	0	0
Simple_repeat	Simple_repeat	(A)n	chr2	86445850	86445884	+	0	0	0
Low_complexity	Low_complexity	A-rich	chr2	86594905	86595027	+	0	0	0
SINE	Alu	B1_Mm	chr2	86836376	86836522	-	0	0	0
LTR	MaLR	MTA_Mm	chr2	86901012	86901404	+	0.591053	0	0
SINE	Alu	B1_Mm	chr2	87335028	87335174	-	0	0	0
SINE	Alu	B1_Mus2	chr2	87337192	87337337	-	0	0	0
Simple_repeat	Simple_repeat	(A)n	chr2	87826766	87826818	+	0	0	0
Simple_repeat	Simple_repeat	(A)n	chr2	87929669	87929752	+	0	0	0
LINE	L1	L1Md_F3	chr2	88006664	88012110	+	0	0	0
LINE	L1	L1Md_T	chr2	88545232	88551738	-	0	0	0
SINE	Alu	B1_Mm	chr2	88566862	88567002	+	0	0	0
Simple_repeat	Simple_repeat	(A)n	chr2	88567003	88567031	+	0	0	0.342623
LINE	L1	L1Md_T	chr2	89355875	89359530	-	0	0	0
LINE	L1	L1Md_T	chr2	89733231	89739899	-	0	0	0
LTR	MaLR	MTA_Mm	chr2	90431303	90431690	-	0	0	0.957947
SINE	Alu	B1_Mus1	chr2	90466500	90466647	+	0	0	0
Simple_repeat	Simple_repeat	(A)n	chr2	90466648	90466680	+	0	0	0
SINE	Alu	B1_Mus1	chr2	90582071	90582215	+	0.591053	0	0
Simple_repeat	Simple_repeat	(A)n	chr2	90582216	90582235	+	0.591053	0	0
SINE	Alu	B1_Mus1	chr2	91564369	91564512	-	0	0	0
Simple_repeat	Simple_repeat	(A)n	chr2	91569820	91569872	+	0	0	0
SINE	B2	B2_Mm1a	chr11	5393491	5393681	-	0	0	0

Simple_repeat	Simple_repeat	(A)n	chr2	93438493	93438529	+	0.591053	0	0
LTR	MaLR	MTA_Mm	chr2	93759975	93760370	+	0	0	0
Simple_repeat	Simple_repeat	(A)n	chr2	94103205	94103249	+	0	0.380568	0
SINE	Alu	B1_Mus2	chr11	5599008	5599134	-	0	0	0
SINE	Alu	B1_Mus2	chr2	94568900	94569046	-	0	0	0
LTR	MaLR	MTA_Mm	chr2	94685320	94685708	+	0	0	0
LINE	L1	L1Md_F2	chr2	94929034	94935257	-	0	0	0
Simple_repeat	Simple_repeat	(A)n	chr2	95158669	95158713	+	0	0	0
SINE	Alu	B1_Mus1	chr2	95161043	95161186	+	0	0	0
Simple_repeat	Simple_repeat	(A)n	chr2	95161187	95161215	+	0	0	0
LINE	L1	L1Md_F2	chr2	95684500	95690724	-	0	0	0
SINE	Alu	B1_Mus2	chr2	96148225	96148370	-	0	0	0
LINE	L1	L1Md_T	chr2	96794845	96801163	-	0	0	0
Simple_repeat	Simple_repeat	(A)n	chr2	96885365	96885408	+	0	0	0
Simple_repeat	Simple_repeat	(A)n	chr2	96951977	96952012	+	0	0	0
Simple_repeat	Simple_repeat	(A)n	chr2	97018336	97018377	+	0	0	0
LINE	L1	L1Md_F2	chr2	97095760	97101107	-	0	0	0
Simple_repeat	Simple_repeat	(A)n	chr2	97121797	97121844	+	0	0	0
SINE	Alu	B1_Mus2	chr2	97123424	97123569	+	0	0	0
Simple_repeat	Simple_repeat	(A)n	chr2	97123570	97123592	+	0	0	0
SINE	Alu	B1_Mus1	chr2	98737189	98737336	+	0	0	0
SINE	Alu	B1_Mm	chr2	98771918	98772064	-	0	0	0
SINE	Alu	B1_Mur2	chr2	99023465	99023602	-	0	0	0
Simple_repeat	Simple_repeat	(A)n	chr2	99237849	99237877	+	0	0	0
SINE	Alu	B1_Mus2	chr2	99241251	99241397	+	0	0	0
SINE	B2	B3	chr11	5996739	5996914	-	1.77316	0	0.615324
SINE	Alu	B1_Mm	chr11	5996947	5997092	-	1.77316	0	0.615324
LTR	MaLR	MTA_Mm	chr2	10034703	10034742	+	0	0	0
LTR	MaLR	MTA_Mm	chr2	10034851	10034891	+	0	0	0
LTR	MaLR	MTA_Mm	chr2	10051354	10051393	+	0	0	0
LTR	MaLR	MTA_Mm	chr2	10051502	10051542	+	0	0.380568	0
SINE	Alu	B1_Mus1	chr2	10085343	10085357	+	0	0	0
Simple_repeat	Simple_repeat	(A)n	chr2	10085357	10085359	+	0	0	0
Simple_repeat	Simple_repeat	(A)n	chr2	10093980	10093983	+	0	0	0
LTR	MaLR	MTB	chr2	10176126	10176173	+	0	0	0
SINE	Alu	B1_Mm	chr2	10224328	10224343	+	0	0	0
Simple_repeat	Simple_repeat	(A)n	chr2	10224343	10224345	+	0	0	0
Simple_repeat	Simple_repeat	(TTTA)n	chr11	6262050	6262091	+	0	0	0
SINE	Alu	B1_Mus2	chr11	6279738	6279884	-	0	0	0
Simple_repeat	Simple_repeat	(A)n	chr2	10365180	10365184	+	0	0	0
SINE	Alu	B1_Mus2	chr11	6324646	6324792	-	0.591053	0	0.342623
Simple_repeat	Simple_repeat	(TTTA)n	chr2	10436202	10436210	+	0	0	0
Simple_repeat	Simple_repeat	(GAAAA)n	chr2	10439869	10439875	+	0	0	0
SINE	Alu	B1_Mus2	chr11	6355358	6355503	+	0	0	0
Simple_repeat	Simple_repeat	(A)n	chr11	6355504	6355528	+	0	0	0
SINE	Alu	B1_Mm	chr2	10469850	10469864	-	0	0	0.615324
Simple_repeat	Simple_repeat	(A)n	chr2	10472345	10472352	+	0	0	0
Simple_repeat	Simple_repeat	(A)n	chr2	10605853	10605856	+	0	0	0
Simple_repeat	Simple_repeat	(A)n	chr2	10677446	10677449	+	0	0	0
SINE	Alu	B1_Mus1	chr2	10727745	10727759	+	0.591053	1.32017	0
Simple_repeat	Simple_repeat	(A)n	chr2	10727759	10727763	+	0.591053	1.32017	0.615324
Simple_repeat	Simple_repeat	(A)n	chr2	10812954	10812958	+	0	0	0
LINE	L1	L1Md_F2	chr2	10832956	10833486	+	0	0	0
Simple_repeat	Simple_repeat	(CAAAAA)n	chr2	10877913	10877926	+	0	0	0
LINE	L1	L1Md_F2	chr2	10892637	10893145	+	0	0	0.342623
Simple_repeat	Simple_repeat	(A)n	chr11	6688972	6689024	+	0.591053	0	0
LINE	L1	L1Md_F2	chr2	10940716	10941244	+	0	0	0
SINE	Alu	B1_Mm	chr2	10975618	10975633	+	0	0	0
Simple_repeat	Simple_repeat	(A)n	chr2	10975633	10975635	+	0	0	0
SINE	Alu	B1_Mus2	chr2	11100152	11100166	+	0	0	0
Simple_repeat	Simple_repeat	(A)n	chr2	11100167	11100169	+	0	0	0
Simple_repeat	Simple_repeat	(A)n	chr2	11120432	11120434	+	0	0	0
Simple_repeat	Simple_repeat	(A)n	chr2	11203186	11203189	+	0	0	0
SINE	B2	B2_Mm1a	chr2	11207661	11207680	+	0	0	0
LTR	MaLR	MTA_Mm	chr2	11229116	11229156	+	0	0	0
SINE	Alu	B1_Mus2	chr2	11290268	11290283	+	0	0	0
Simple_repeat	Simple_repeat	(A)n	chr2	11290283	11290285	+	0	0	0

SINE	Alu	B1_Mus1	chr2	11518842	11518858	-	0	0	0
SINE	Alu	B1_Mus1	chr2	11565018	11565033	-	0	0	0
SINE	B2	B2_Mm1a	chr2	11593778	11593797	+	0	0	0
Simple_repeat	Simple_repeat	(A)n	chr2	11593797	11593800	+	0.332194	0	0
SINE	Alu	B1_Mus2	chr2	11601715	11601730	-	0	0	0
SINE	Alu	B1_Mus1	chr2	11605683	11605697	+	0	0	0
Simple_repeat	Simple_repeat	(A)n	chr2	11605697	11605700	+	0	0	0
Simple_repeat	Simple_repeat	(CCAA)n	chr2	11605700	11605702	+	0	0	0
LTR	MaLR	MTA_Mm	chr2	11627579	11627619	+	0	0	0
Simple_repeat	Simple_repeat	(A)n	chr2	11648495	11648499	+	0	0	0
Simple_repeat	Simple_repeat	(A)n	chr2	11664922	11664926	+	0	0	0
Simple_repeat	Simple_repeat	(A)n	chr2	11667158	11667160	+	0	0	0
LTR	MaLR	MTB	chr2	11678037	11678076	-	0	0	0
SINE	Alu	B1_Mus2	chr11	7527922	7528068	-	0	0	0
Simple_repeat	Simple_repeat	(A)n	chr2	11841873	11841877	+	0	0	0
SINE	Alu	B1_Mus2	chr2	11868528	11868543	+	0	0	0
Simple_repeat	Simple_repeat	(A)n	chr2	11868543	11868546	+	0.591053	0	0
SINE	Alu	B1_Mus1	chr2	11893766	11893780	+	0	0	0
Simple_repeat	Simple_repeat	(A)n	chr2	11893780	11893782	+	0	0	0
SINE	Alu	B1_Mus1	chr11	7690661	7690807	-	0	0	0
Simple_repeat	Simple_repeat	(A)n	chr2	11931246	11931250	+	0	0	0
SINE	Alu	B1_Mus2	chr2	11998573	11998586	+	0	0	0
SINE	Alu	B1_Mus2	chr2	12011767	12011782	-	0	0	0
SINE	B2	B2_Mm1t	chr2	12050799	12050818	-	0	0	0
SINE	Alu	B1_Mm	chr2	12130431	12130446	+	0.591053	0	0
Simple_repeat	Simple_repeat	(TTTA)n	chr2	12205936	12205941	+	0	0	0
SINE	Alu	B1_Mus2	chr2	12229035	12229050	+	0	0	0
Simple_repeat	Simple_repeat	(A)n	chr2	12229051	12229062	+	0	0	0
LINE	L1	L1Md_F2	chr2	12238879	12239505	-	0	0	0
SINE	Alu	B1_Mus2	chr2	12248636	12248651	-	0	0	0
SINE	Alu	B1_Mus1	chr2	12364606	12364621	+	0	0	0
Simple_repeat	Simple_repeat	(A)n	chr2	12364623	12364629	+	0	0	0.342623
LTR	MaLR	MTA_Mm	chr2	12396140	12396177	-	0	0	0
Simple_repeat	Simple_repeat	(A)n	chr2	12417551	12417555	+	0	0	0
SINE	Alu	B1_Mm	chr2	12420196	12420211	-	0	0	0
LTR	MaLR	MTA_Mm	chr2	12430002	12430041	+	0	0	0
SINE	Alu	B1_Mus2	chr2	12698296	12698311	+	0	0	0
Simple_repeat	Simple_repeat	(A)n	chr2	12698311	12698314	+	0	0	0
SINE	Alu	B1_Mus2	chr2	12750883	12750897	-	0	0	0
SINE	Alu	B1_Mm	chr2	12851960	12851975	-	0	0	0
SINE	Alu	B1_Mus2	chr2	12863213	12863227	+	0	0	0
Simple_repeat	Simple_repeat	(A)n	chr2	12863228	12863230	+	0	0	0
SINE	Alu	B1_Mus1	chr2	12897171	12897186	-	0	0	0.615324
SINE	Alu	B1_Mm	chr2	12898130	12898142	-	0	0	0
SINE	Alu	B1_Mus1	chr2	12898364	12898378	+	0	0	0
Simple_repeat	Simple_repeat	(A)n	chr2	12898378	12898384	+	0	0	0
Simple_repeat	Simple_repeat	(A)n	chr2	12901325	12901332	+	0	0	0
SINE	Alu	B1_Mm	chr2	12962339	12962353	+	0	0	0
Simple_repeat	Simple_repeat	(A)n	chr2	12962353	12962356	+	0	0	0
SINE	Alu	B1_Mm	chr2	12980841	12980855	-	0	0	0
SINE	Alu	B1_Mus2	chr2	13052506	13052519	+	0	0	0
Simple_repeat	Simple_repeat	(A)n	chr2	13052519	13052521	+	0	0	0
LTR	MaLR	MTA_Mm	chr2	13068516	13068555	+	0.591053	0	0
LTR	MaLR	MTA_Mm	chr2	13094768	13094808	-	0	0	0
LTR	MaLR	MTB	chr2	13140538	13140578	+	0	0	0
SINE	Alu	B1_Mur4	chr2	13199661	13199676	+	0	0	0
Simple_repeat	Simple_repeat	(A)n	chr2	13199676	13199679	+	0	0	0
LTR	MaLR	MTA_Mm	chr2	13224549	13224588	-	0	0	0
Simple_repeat	Simple_repeat	(A)n	chr11	9643899	9643933	+	0	0	0
LTR	MaLR	MTB	chr2	13249320	13249360	-	0	0	0
LINE	L1	L1_Mur1	chr2	13414770	13415271	+	0	0	0
SINE	Alu	B1_Mus2	chr2	13448096	13448110	-	0	0	0
LINE	L1	L1_Mus3	chr11	10018107	10023288	-	0	0	0
Simple_repeat	Simple_repeat	(TTTA)n	chr2	13624163	13624166	+	0	0	0
LTR	MaLR	MTA_Mm	chr2	13634418	13634458	-	0	0	0
SINE	Alu	B1_Mm	chr2	13649157	13649171	+	0	0	0
Simple_repeat	Simple_repeat	(A)n	chr2	13649172	13649175	+	0	0	0

Simple_repeat	Simple_repeat	(A)n	chr2	13690005	13690009	+	0	0	0
SINE	Alu	B1_Mus2	chr2	13737192	13737207	+	0	0	0
SINE	Alu	B1_Mm	chr2	13740380	13740394	+	0	0	0
Simple_repeat	Simple_repeat	(A)n	chr2	13740395	13740400	+	0	0	0
Simple_repeat	Simple_repeat	(A)n	chr2	13748758	13748763	+	0	0	0
SINE	Alu	B1_Mus1	chr2	13811494	13811504	-	0	0	0
Simple_repeat	Simple_repeat	(A)n	chr2	13815568	13815572	+	0	0	0
LTR	MaLR	MTA_Mm	chr11	10291426	10291815	-	0	0	0
Simple_repeat	Simple_repeat	(A)n	chr2	13859401	13859407	+	0	0.708814	0
SINE	Alu	B1_Mus2	chr2	13877274	13877289	-	0	0	0
SINE	B2	B2_Mm1a	chr2	13993125	13993145	+	0	0	0
LTR	MaLR	MTA_Mm	chr11	10540093	10540488	-	0.591053	0	0
SINE	Alu	B1_Mus2	chr11	10550131	10550277	+	0	0	0
Simple_repeat	Simple_repeat	(A)n	chr11	10550278	10550305	+	0	0.708814	0
SINE	Alu	B1_Mus2	chr2	14274505	14274520	-	0	0	0
Simple_repeat	Simple_repeat	(A)n	chr2	14303284	14303288	+	0	0	0
Simple_repeat	Simple_repeat	(A)n	chr2	14315218	14315221	+	0	0	0
LINE	L1	Lx	chr10	17612750	17617503	-	0.591053	1.41763	0.342623
SINE	Alu	B1_Mus2	chr2	14365152	14365166	-	0	0	0
SINE	Alu	B1_Mm	chr2	14402384	14402398	+	0	0	0.342623
Simple_repeat	Simple_repeat	(A)n	chr2	14402398	14402401	+	0	0	0
Simple_repeat	Simple_repeat	(A)n	chr11	10910239	10910272	+	0	0	0
SINE	Alu	B1_Mm	chr2	14416566	14416581	-	0	0	0
SINE	Alu	B1_Mm	chr2	14417469	14417483	-	0	0	0
SINE	Alu	B1_Mus2	chr2	14423528	14423542	-	0	0	0
SINE	Alu	B1_Mus1	chr2	14424160	14424174	-	0	0	0
SINE	Alu	B1_Mus2	chr2	14497841	14497855	-	0	0	0
LTR	MaLR	MTA_Mm	chr2	14558792	14558832	-	0	0	0
SINE	Alu	B1_Mus1	chr2	14661634	14661651	+	0	0	0
Simple_repeat	Simple_repeat	(A)n	chr2	14661651	14661655	+	0	0	0
SINE	Alu	B1_Mus2	chr2	14667110	14667125	-	0	0	0
LTR	MaLR	MTA_Mm	chr2	14761006	14761045	+	0	0	0
SINE	Alu	B1_Mur2	chr2	14779754	14779770	-	0	0	0
SINE	Alu	B1_Mm	chr2	14815542	14815557	+	0	0	0
Simple_repeat	Simple_repeat	(A)n	chr2	14823546	14823550	+	0.332194	0	0.342623
Simple_repeat	Simple_repeat	(A)n	chr2	14846740	14846745	+	0	0	0
Simple_repeat	Simple_repeat	(A)n	chr2	14938288	14938292	+	0	0	0
SINE	Alu	B1_Mus2	chr2	14941085	14941099	-	0	0	0.615324
SINE	Alu	B1_Mm	chr2	15011423	15011437	-	0	0	0
SINE	Alu	B1_Mm	chr2	15012728	15012742	+	0	0	0
Simple_repeat	Simple_repeat	(A)n	chr2	15012742	15012746	+	0	0	0
Simple_repeat	Simple_repeat	(A)n	chr11	11575224	11575262	+	0.332194	0	0
SINE	Alu	B1_Mm	chr2	15019380	15019395	-	0	0	0
SINE	Alu	B1_Mus2	chr2	15021270	15021285	-	0.591053	0	1.23065
SINE	Alu	B1_Mus2	chr2	15031425	15031439	-	0	0	0
SINE	Alu	B1_Mm	chr2	15032348	15032363	-	0.923247	1.08938	0
LINE	L1	L1Md_T	chr2	15032927	15033462	-	0	0	0
SINE	Alu	B1_Mm	chr2	15035541	15035556	-	0	0	0
SINE	Alu	B1_Mm	chr11	11678180	11678329	-	0	0	0
LTR	MaLR	MTA_Mm	chr2	15157071	15157110	+	0	0	0.342623
SINE	Alu	B1_Mm	chr11	11818442	11818587	-	0	0	0
SINE	Alu	B1_Mus2	chr2	15259138	15259151	+	0	0	0
Simple_repeat	Simple_repeat	(A)n	chr2	15259152	15259155	+	0	0	0
SINE	Alu	B1_Mur3	chr2	15310696	15310707	+	0	0	0
Low_complexity	Low_complexity	A-rich	chr2	15310707	15310714	+	0	0	0
Simple_repeat	Simple_repeat	(TTTTA)n	chr2	15341450	15341454	+	0	0	0
Simple_repeat	Simple_repeat	(A)n	chr2	15341501	15341504	+	0	0	0.342623
SINE	Alu	B1_Mus2	chr2	15344158	15344172	+	0	0	0
Simple_repeat	Simple_repeat	(A)n	chr2	15344172	15344175	+	0	0	0
SINE	Alu	B1_Mus2	chr2	15353440	15353455	-	0	0	0
Simple_repeat	Simple_repeat	(A)n	chr2	15392021	15392025	+	0	0	0
Simple_repeat	Simple_repeat	(A)n	chr2	15412958	15412962	+	0	0	0
SINE	Alu	B1_Mus2	chr11	12416163	12416309	-	0	0	0
SINE	Alu	B1_Mm	chr2	15672103	15672118	-	0	0	0
SINE	Alu	B1_Mus1	chr2	15683367	15683381	+	0	0	0
Simple_repeat	Simple_repeat	(A)n	chr2	15683381	15683384	+	0	0	0
SINE	Alu	B1_Mus2	chr2	15742319	15742333	+	0	0	0

Simple_repeat	Simple_repeat	(A)n	chr2	15742333	15742336	+	0	0	0
Simple_repeat	Simple_repeat	(A)n	chr2	15755182	15755188	+	0	0	1.10507
SINE	Alu	B1_Mm	chr2	15779858	15779873	+	0	0	0
Simple_repeat	Simple_repeat	(A)n	chr2	15779873	15779876	+	0	0	0
LTR	MaLR	MTA_Mm	chr11	12807604	12807999	-	0	0	0
LTR	MaLR	MTA_Mm	chr11	12957698	12958090	+	0	0	0.615324
SINE	Alu	B1_Mus1	chr2	15921201	15921216	-	0	0	0
SINE	Alu	B1_Mus2	chr11	13068885	13069030	+	0	0	0
Simple_repeat	Simple_repeat	(A)n	chr11	13069031	13069058	+	0	0	0
SINE	Alu	B1_Mm	chr2	16013381	16013396	-	0	0	0
Simple_repeat	Simple_repeat	(A)n	chr2	16035486	16035490	+	0	0	0
Simple_repeat	Simple_repeat	(A)n	chr2	16036974	16036977	+	0	0	0
SINE	Alu	B1_Mus1	chr2	16040315	16040333	-	0	0	0
LTR	MaLR	MTA_Mm	chr10	3125491	3125864	+	0	0	0
Simple_repeat	Simple_repeat	(A)n	chr2	16105492	16105496	+	0	0	0
SINE	Alu	B1_Mm	chr2	16108704	16108719	+	0	0	0
Simple_repeat	Simple_repeat	(A)n	chr2	16108719	16108725	+	0	0	0
SINE	Alu	B1_Mus2	chr11	13355751	13355897	-	0	0	0
Simple_repeat	Simple_repeat	(A)n	chr2	16261470	16261473	+	0	0	0
SINE	Alu	B1_Mus1	chr11	13454360	13454507	-	0	0	0
SINE	Alu	B1_Mus1	chr2	16344712	16344727	+	0	0	0
Simple_repeat	Simple_repeat	(A)n	chr2	16344727	16344731	+	0	0	0
SINE	Alu	B1_Mus1	chr2	16347211	16347226	+	0	0	0
Simple_repeat	Simple_repeat	(A)n	chr2	16347226	16347229	+	0.332194	0	0
Simple_repeat	Simple_repeat	(A)n	chr11	13591820	13591873	+	0	0	0
SINE	Alu	B1_Mm	chr2	16451653	16451668	-	0	0	0
LTR	MaLR	MTA_Mm	chr11	13941232	13941570	+	0	0	0
SINE	Alu	B1_Mm	chr2	16616066	16616080	-	0	0	0
SINE	Alu	B1_Mus2	chr2	16638083	16638098	-	0	0	0
SINE	Alu	B1_Mur4	chr2	16659387	16659400	-	0	0	0
SINE	Alu	B1_Mm	chr2	16659760	16659775	-	0	0	0
SINE	Alu	B1_Mus2	chr2	16661371	16661385	+	0	0	0
Simple_repeat	Simple_repeat	(A)n	chr2	16702301	16702303	+	0.923247	0	0.615324
SINE	Alu	B1_Mus2	chr2	16722073	16722088	+	0	0	0
Simple_repeat	Simple_repeat	(A)n	chr2	16722088	16722090	+	0	0	0
SINE	Alu	B1_Mus2	chr2	16746072	16746088	-	0	0	0
SINE	Alu	B1_Mus1	chr2	16750737	16750751	-	0	0	0
SINE	Alu	B1_Mur1	chr2	16807432	16807447	-	0	0	0
Simple_repeat	Simple_repeat	(A)n	chr2	16813283	16813285	+	0	0	0
SINE	Alu	B1_Mus2	chr2	16813707	16813721	+	0	0	0
Simple_repeat	Simple_repeat	(A)n	chr2	16813721	16813724	+	0	0	0
LINE	L1	L1_Mus2	chr11	14359886	14365793	-	0	0	0
SINE	B2	B2_Mm1a	chr2	16861489	16861509	-	0	0	0
SINE	Alu	B1_Mm	chr2	16865160	16865173	+	0	0	0
Simple_repeat	Simple_repeat	(A)n	chr2	16865173	16865175	+	0	0	0
SINE	B2	B2_Mm1a	chr2	16873979	16873998	-	0	0.380568	0
SINE	Alu	B1_Mus1	chr2	16878874	16878889	-	0	0	0
Simple_repeat	Simple_repeat	(A)n	chr2	16980293	16980300	+	0	0	0
SINE	Alu	B1_Mus2	chr2	16996857	16996871	-	0	0	0
Simple_repeat	Simple_repeat	(A)n	chr2	17029551	17029554	+	0	0	0
Simple_repeat	Simple_repeat	(A)n	chr11	14699506	14699544	+	0	0	0
LTR	MaLR	MTA_Mm	chr2	17180948	17180988	+	0	0	0
SINE	Alu	B1_Mus2	chr2	17191143	17191158	+	0.664388	0	0
Simple_repeat	Simple_repeat	(A)n	chr2	17191158	17191160	+	0	0	0
Simple_repeat	Simple_repeat	(A)n	chr2	17199403	17199407	+	0	0	0
SINE	Alu	B1_Mus2	chr2	17214545	17214560	-	0	0	0
SINE	Alu	PB1D10	chr2	17248138	17248149	+	0	0	0
Simple_repeat	Simple_repeat	(A)n	chr2	17248150	17248154	+	0	0	0
SINE	B2	B2_Mm1a	chr2	17279389	17279408	+	0	0	0
SINE	B4	ID_B1	chr2	17291539	17291560	-	0	0	0
LTR	MaLR	MTA_Mm	chr2	17370505	17370543	+	0	0	0
Simple_repeat	Simple_repeat	(A)n	chr11	15149916	15149951	+	0	0	0
SINE	Alu	B1_Mm	chr2	17407601	17407615	+	0	0	0
SINE	Alu	B1_Mm	chr2	17458461	17458475	+	0	0	0
Simple_repeat	Simple_repeat	(A)n	chr2	17458475	17458477	+	0	0	0
LTR	MaLR	MTA_Mm	chr2	17473586	17473624	+	0	0	0
SINE	Alu	B1_Mus1	chr2	17488092	17488106	+	0	0	0

Simple_repeat	Simple_repeat	(A)n	chr2	17488106	17488110	+	0	0	0
SINE	Alu	B1_Mus2	chr2	17548966	17548981	-	0	0	0
SINE	Alu	B1_Mus2	chr2	17550934	17550949	+	0.591053	0	0
SINE	Alu	B1_Mus2	chr11	15371734	15371880	+	0	0	0
Simple_repeat	Simple_repeat	(A)n	chr11	15371881	15371903	+	0	0	0
SINE	Alu	B1_Mus1	chr2	17687749	17687763	-	0	0	0.615324
SINE	Alu	B1_Mus2	chr2	17713380	17713394	+	0	0	0
SINE	Alu	B1_Mus2	chr2	17720426	17720441	-	0	0	0
SINE	Alu	B1_Mus2	chr2	17745319	17745334	-	0	0	0
SINE	Alu	B1_Mus2	chr2	17747260	17747274	+	0	0	0
Simple_repeat	Simple_repeat	(A)n	chr2	17747274	17747278	+	0	0	0
SINE	Alu	B1_Mus2	chr2	17753013	17753027	+	0	0	0
SINE	Alu	B1_Mus2	chr2	17758654	17758668	-	0	0	0
Simple_repeat	Simple_repeat	(A)n	chr2	17767373	17767377	+	0	0	0
Simple_repeat	Simple_repeat	(A)n	chr11	15628316	15628362	+	0	0	0
LINE	L1	L1Md_F2	chr2	17888592	17889132	+	0	0	0
Simple_repeat	Simple_repeat	(A)n	chr2	17903889	17903892	+	0	0	0
LTR	MaLR	MTA_Mm	chr2	17905205	17905244	+	0	0	0
Simple_repeat	Simple_repeat	(A)n	chr11	15765076	15765127	+	0	0	0
Simple_repeat	Simple_repeat	(A)n	chr11	15793213	15793251	+	0	0	0
SINE	Alu	B1_Mus2	chr2	18045409	18045423	-	0	0	0
SINE	B2	B2_Mm1t	chr2	18143466	18143486	+	0	0	0
LTR	MaLR	MTA_Mm	chr10	4599525	4599922	-	0	0	0
LTR	MaLR	MTA_Mm	chr10	18002760	18003137	-	0	0	0
LTR	MaLR	MTA_Mm	chr3	3678720	3679115	-	0	0	0
LINE	L1	L1Md_T	chr3	4164192	4170853	-	0	0	0
Simple_repeat	Simple_repeat	(A)n	chr3	4447388	4447436	+	0	0	0
LTR	MaLR	MTA_Mm	chr11	16225227	16225624	-	0	0	0
LINE	L1	L1_Mus1	chr3	6188692	6194794	+	0.591053	0	0
SINE	Alu	B1_Mus2	chr3	6219439	6219585	+	0	0	0
Simple_repeat	Simple_repeat	(A)n	chr3	6219586	6219620	+	0	0	0
LTR	MaLR	MTA_Mm	chr3	6265004	6265398	-	0	0	0
SINE	Alu	B1_Mus2	chr3	6436386	6436531	+	0	0	0
Low_complexity	Low_complexity	A-rich	chr3	6436532	6436624	+	0	0	0
SINE	Alu	B1_Mus2	chr3	6760274	6760418	+	0	0	0
Simple_repeat	Simple_repeat	(A)n	chr3	6760419	6760499	+	0	0	0
Simple_repeat	Simple_repeat	(A)n	chr3	6785486	6785537	+	0	0	0
SINE	Alu	B1_Mus2	chr3	6957704	6957812	+	0	0	0
Simple_repeat	Simple_repeat	(A)n	chr3	6957813	6957844	+	0	0	0
Simple_repeat	Simple_repeat	(A)n	chr3	6959282	6959328	+	0	0	0
Simple_repeat	Simple_repeat	(A)n	chr3	7010425	7010470	+	0	0	0
SINE	Alu	B1_Mm	chr11	16437726	16437869	-	0	0	0
SINE	Alu	B1_Mus1	chr3	8232960	8233105	+	0	0	0
Simple_repeat	Simple_repeat	(A)n	chr3	8233106	8233130	+	0	0	0
SINE	Alu	B1_Mus1	chr3	8245571	8245718	+	0	0	0
Simple_repeat	Simple_repeat	(CAAAC)n	chr3	8245747	8245769	+	0.591053	0	0.615324
Simple_repeat	Simple_repeat	(A)n	chr11	16477999	16478036	+	0	0	0
LINE	L1	L1Md_F2	chr3	8330527	8335703	+	0	0	0
LTR	MaLR	MTA_Mm	chr3	8651898	8652278	+	0	0	0
SINE	Alu	B1_Mus2	chr3	9011506	9011617	+	0	0	0
SINE	Alu	B1_Mur4	chr3	9241405	9241544	+	0	0	0
Simple_repeat	Simple_repeat	(A)n	chr3	9241545	9241603	+	0	0	0
LTR	MaLR	MTA_Mm	chr3	9416687	9417072	-	0	0.380568	0
LTR	MaLR	MTA_Mm	chr3	10073766	10074161	-	0	0	0
Simple_repeat	Simple_repeat	(A)n	chr3	10097278	10097305	+	0	0	0
SINE	Alu	B1_Mus1	chr3	10134119	10134264	-	0	0	0
SINE	Alu	B1_Mus2	chr3	10151987	10152132	-	0	0	0
SINE	Alu	B1_Mus1	chr3	10824527	10824671	+	0	0	0
Simple_repeat	Simple_repeat	(A)n	chr3	10824672	10824703	+	0	0	0
LTR	MaLR	MTA_Mm	chr3	11012719	11013112	+	0	0	0
LINE	L1	L1Md_F2	chr3	11367351	11372700	+	0	0	0
Simple_repeat	Simple_repeat	(A)n	chr3	11615025	11615049	+	0	0	0
Simple_repeat	Simple_repeat	(A)n	chr3	11729266	11729329	+	0	0	0
LTR	MaLR	MTA_Mm	chr3	12113117	12113504	-	0	0	0
LINE	L1	L1Md_F2	chr3	12619618	12625330	+	0	0	0
SINE	Alu	B1_Mus1	chr3	12672844	12672990	+	0	0	0
Simple_repeat	Simple_repeat	(A)n	chr3	12672991	12673014	+	0	0	0

LTR	MaLR	MTA_Mm	chr3	12887285	12887679	-	0	0	0
SINE	Alu	B1_Mus1	chr3	12941557	12941703	+	0	0	0
Simple_repeat	Simple_repeat	(A)n	chr3	13352572	13352607	+	0	0.380568	0
LTR	MaLR	MTA_Mm	chr3	15189734	15190130	+	0	0	0
Simple_repeat	Simple_repeat	(A)n	chr3	15693803	15693843	+	0	0	0
Simple_repeat	Simple_repeat	(A)n	chr3	15752839	15752924	+	0	0	0.615324
LINE	L1	L1Md_T	chr3	15822545	15829064	-	0	0	0
SINE	Alu	B1_Mm	chr3	16043126	16043272	+	0	0	0
LTR	ERVK	RMER13A	chr3	16762314	16763095	-	0	0	0
Simple_repeat	Simple_repeat	(A)n	chr3	16881572	16881639	+	0.827951	0	0
LINE	L1	L1Md_F2	chr3	17413654	17419583	+	0	0	0
LTR	MaLR	MTA_Mm	chr3	17810628	17811014	+	0	0	0
SINE	Alu	B1_Mm	chr3	18262571	18262717	-	0	0	0
LINE	L1	L1_Mus1	chr3	18304422	18310872	+	0	0	0
Low_complexity	Low_complexity	CT-rich	chr3	19314608	19314658	+	0	0	0
LTR	MaLR	MTA_Mm	chr3	19702274	19702657	+	0	0	0
LTR	MaLR	MTA_Mm	chr10	18099028	18099424	-	0	0	0
LTR	MaLR	MTA_Mm	chr3	20196444	20196838	-	0	0	0
SINE	Alu	B1_Mm	chr3	20461059	20461205	-	0	0	0
LTR	MaLR	MTA_Mm	chr3	20688518	20688890	-	0	0	0
SINE	Alu	B1_Mus2	chr3	20805125	20805272	-	0	0	0
SINE	Alu	B1_Mm	chr3	20950943	20951099	+	1.18211	0.708814	0.342623
Simple_repeat	Simple_repeat	(A)n	chr3	20951100	20951123	+	1.18211	0	0
LTR	MaLR	MTEa	chr3	21188701	21189042	+	0	0	0
SINE	B4	B4	chr3	21594385	21594677	+	0	0	0
SINE	Alu	B1_Mus1	chr3	21778822	21778969	+	0	0	0
Simple_repeat	Simple_repeat	(A)n	chr3	21778970	21778993	+	0	0	0
SINE	Alu	B1_Mus2	chr3	21801481	21801627	-	0	0	0
Simple_repeat	Simple_repeat	(A)n	chr3	22120588	22120649	+	0.591053	0.708814	0
LINE	L1	L1Md_A	chr3	22347096	22352613	-	0	0	0
LINE	L1	L1Md_T	chr3	22352630	22359509	-	0.591053	0	0
LINE	L1	L1Md_F2	chr3	22588804	22595023	-	0	0	0
SINE	B2	B2_Mm1t	chr11	17875355	17875546	+	0	0	0
Simple_repeat	Simple_repeat	(A)n	chr11	17875547	17875583	+	0.332194	0	1.4477
LINE	L1	L1Md_T	chr3	22906497	22914001	-	0	0	0
Simple_repeat	Simple_repeat	(A)n	chr3	23742220	23742244	+	0	0	0
LTR	MaLR	MTA_Mm	chr3	23803971	23804364	-	0	0	0
Simple_repeat	Simple_repeat	(A)n	chr3	24179577	24179610	+	0	0	0
Simple_repeat	Simple_repeat	(A)n	chr3	24232475	24232509	+	18.3018	11.2056	11.6282
Simple_repeat	Simple_repeat	(A)n	chr3	24446038	24446100	+	0	0	0
SINE	Alu	B1_Mus2	chr3	24472456	24472602	+	0	0	0
Simple_repeat	Simple_repeat	(A)n	chr3	24472603	24472626	+	0	0	0
LTR	MaLR	MTA_Mm	chr3	24585102	24585496	-	0	0	0
Simple_repeat	Simple_repeat	(A)n	chr3	24808479	24808508	+	0	0	0
SINE	Alu	B1_Mus2	chr3	27755964	27756110	-	0	0	0
Simple_repeat	Simple_repeat	(TTTA)n	chr3	28041748	28041778	+	0	0	0
SINE	Alu	B1_Mus1	chr11	18518995	18519133	-	0	0	0
Simple_repeat	Simple_repeat	(A)n	chr3	28639858	28639896	+	0	0	0
LINE	L1	L1Md_T	chr3	29765054	29771712	-	0	0	0
Simple_repeat	Simple_repeat	(A)n	chr3	29827882	29827902	+	0	0	0
Simple_repeat	Simple_repeat	(A)n	chr3	30413765	30413794	+	0	0	0
LTR	MaLR	MTA_Mm	chr3	30462654	30462977	+	0	0	0
SINE	Alu	B1_Mus1	chr3	30796189	30796326	-	0.591053	0	0.342623
Simple_repeat	Simple_repeat	(A)n	chr3	31509047	31509094	+	0	0	0
LTR	MaLR	MTA_Mm	chr3	31738383	31738778	+	0	0	0
SINE	Alu	B1_Mus2	chr3	32294676	32294812	+	0	0	0
Simple_repeat	Simple_repeat	(A)n	chr3	32294813	32294838	+	0	0	0
SINE	Alu	B1_Mus1	chr3	33487701	33487849	-	0	0	0
SINE	Alu	B1_Mus2	chr3	34617241	34617387	+	0	0	0
SINE	Alu	B1_Mus1	chr3	34940470	34940616	-	0.591053	0	0
Simple_repeat	Simple_repeat	(A)n	chr3	34977271	34977331	+	0	0	0
Simple_repeat	Simple_repeat	(A)n	chr3	36246418	36246449	+	0	0	0
LTR	MaLR	MTA_Mm	chr3	36656824	36657205	+	0	0	0
LTR	MaLR	MTA_Mm	chr3	36658297	36658678	+	0	0	0
LTR	MaLR	MTA_Mm	chr3	37481266	37481671	+	0	0	0.615324
LTR	MaLR	MTA_Mm	chr10	18284560	18284939	+	0	0	0
SINE	Alu	B1_Mus1	chr3	38764013	38764163	-	0	0	0

SINE	Alu	B1_Mus2	chr3	38769792	38769937	-	0	0	0
Simple_repeat	Simple_repeat	(A)n	chr3	39045673	39045699	+	0	0	0
SINE	Alu	B1_Mm	chr3	39303477	39303622	-	0	0	0
Simple_repeat	Simple_repeat	(A)n	chr3	39390451	39390485	+	0	0	0
LTR	MaLR	MTA_Mm	chr3	40861330	40861694	+	0	0	0
LINE	L1	L1Md_F2	chr3	41594751	41599914	+	0	0.708814	0.342623
LTR	MaLR	MTA_Mm	chr3	41655818	41656212	-	0.332194	0	0
SINE	Alu	B1_Mm	chr11	19931275	19931419	-	0	0	0
SINE	Alu	B1_Mus2	chr11	19955126	19955272	-	0	0	0
Simple_repeat	Simple_repeat	(A)n	chr3	42986291	42986334	+	0.591053	0	0
Simple_repeat	Simple_repeat	(A)n	chr3	43098577	43098642	+	0	0	0
Simple_repeat	Simple_repeat	(A)n	chr3	43159367	43159402	+	0	0	0
LTR	MaLR	MTA_Mm	chr3	43515394	43515788	+	0.332194	0.708814	0.866667
Simple_repeat	Simple_repeat	(A)n	chr3	43787145	43787175	+	0	0	0
Simple_repeat	Simple_repeat	(A)n	chr3	44133411	44133438	+	0	0	0
SINE	Alu	B1_Mur3	chr11	20044190	20044337	-	0.591053	0	0
SINE	Alu	B1_Mus2	chr3	44555441	44555595	-	0	0	0
LINE	L1	L1Md_F2	chr3	44730975	44737160	-	0	0	0
Simple_repeat	Simple_repeat	(A)n	chr3	44851511	44851561	+	0	0	0
Simple_repeat	Simple_repeat	(A)n	chr3	44991437	44991478	+	0	0	0
LTR	MaLR	MTA_Mm	chr3	45314976	45315367	-	0	0	0
LTR	MaLR	MTA_Mm	chr3	45321646	45322040	+	0	0	0
Simple_repeat	Simple_repeat	(A)n	chr3	45468770	45468813	+	0	0	0
Simple_repeat	Simple_repeat	(A)n	chr3	46114710	46114744	+	0	0	0
Simple_repeat	Simple_repeat	(A)n	chr3	46172366	46172410	+	0	0	0
SINE	Alu	B1_Mus2	chr3	46254464	46254606	+	0	0	0
Simple_repeat	Simple_repeat	(A)n	chr3	46670281	46670378	+	0.332194	0	0
SINE	Alu	B1_Mus1	chr3	46801879	46802023	+	0	0	0
Simple_repeat	Simple_repeat	(A)n	chr3	46802024	46802046	+	0	0	0
SINE	Alu	B1_Mus1	chr3	47001791	47001937	-	0	0	0
LINE	L1	L1Md_F3	chr3	47105820	47112010	-	0	0	0
Simple_repeat	Simple_repeat	(A)n	chr3	47266569	47266609	+	0	0	0
LTR	MaLR	MTA_Mm	chr3	47453364	47453736	+	0	0	0
LTR	MaLR	MTA_Mm	chr3	47460138	47460533	-	0	0	0
LTR	MaLR	MTA_Mm	chr3	47601653	47602046	-	0	0	0
LTR	MaLR	MTA_Mm	chr3	47603143	47603536	-	0	0	0
LTR	ERV1	RLTR24	chr11	20269083	20269531	+	0	0	0
LTR	MaLR	MTA_Mm	chr3	49129863	49130256	+	0	0	0
LTR	MaLR	MTA_Mm	chr3	49131308	49131701	+	0	0	0
LTR	MaLR	MTA_Mm	chr11	20321657	20322048	+	0	0	0
SINE	Alu	B1_Mus2	chr11	20324806	20324951	+	0	0	0
SINE	Alu	B1_Mus1	chr11	20374898	20375040	+	0	0	0
Simple_repeat	Simple_repeat	(A)n	chr11	20375041	20375068	+	0	0	0
Simple_repeat	Simple_repeat	(A)n	chr3	50653260	50653301	+	0	0	0
SINE	Alu	B1_Mus2	chr11	20408776	20408922	-	0	0	0
SINE	Alu	B1_Mus2	chr3	51928899	51929044	+	0	0	0
Simple_repeat	Simple_repeat	(A)n	chr3	51929045	51929067	+	0	0	0
SINE	Alu	B1_Mus2	chr3	52017487	52017634	+	0	0	0
Simple_repeat	Simple_repeat	(A)n	chr3	52017635	52017671	+	0	0	0
SINE	B2	B2_Mm1a	chr3	52279434	52279625	-	0	0	0
SINE	Alu	B1_Mm	chr3	53165048	53165194	+	0	0	0
LINE	L1	L1Md_T	chr3	53519032	53525883	-	0	0	0
Simple_repeat	Simple_repeat	(A)n	chr3	53603103	53603140	+	0	0	0
SINE	Alu	B1_Mus2	chr3	53619756	53619900	-	0	0	0
SINE	Alu	B1_Mus2	chr3	53786002	53786149	+	0	0	0
Simple_repeat	Simple_repeat	(A)n	chr3	53786150	53786172	+	0	0	0
Low_complexity	Low_complexity	A-rich	chr3	53892577	53892665	+	0	0	0
Simple_repeat	Simple_repeat	(A)n	chr3	53906636	53906685	+	0	0	0
LTR	MaLR	MTA_Mm	chr3	54253940	54254334	+	0	0	0
Simple_repeat	Simple_repeat	(A)n	chr3	54391179	54391213	+	0	0	0
Simple_repeat	Simple_repeat	(CAAAC)n	chr11	20837715	20837742	+	0	0	0
Simple_repeat	Simple_repeat	(A)n	chr3	55991948	55991982	+	0.591053	0	0.615324
Simple_repeat	Simple_repeat	(A)n	chr3	56058482	56058509	+	0	0	0
LTR	MaLR	MTA_Mm	chr3	56129641	56130037	+	0	0	0
LINE	L1	L1Md_F2	chr3	56352275	56357275	-	0	0	0
SINE	Alu	B1_Mus2	chr3	57174380	57174526	+	0	0	0
Simple_repeat	Simple_repeat	(A)n	chr3	57174527	57174569	+	0	0	0.342623

SINE	Alu	B1_Mus2	chr3	57381654	57381800	-	0	0	0
SINE	Alu	B1_Mus2	chr3	57387061	57387207	+	0	0	0
Simple_repeat	Simple_repeat	(A)n	chr3	57387208	57387231	+	0	0	0
LTR	MaLR	MTA_Mm	chr10	4653854	4654253	+	0	0	0
SINE	Alu	B1_Mm	chr3	58279183	58279327	+	0	0	0
LINE	L1	L1Md_F2	chr3	58404445	58409468	+	0.591053	0.708814	0.615324
SINE	Alu	B1_Mus1	chr11	21128285	21128430	-	0	0	0
LTR	MaLR	MTA_Mm	chr3	59510019	59510414	-	0	0	0
LTR	MaLR	MTA_Mm	chr3	59511504	59511895	-	0	0	0
Simple_repeat	Simple_repeat	(TTTA)n	chr10	18507499	18507542	+	0	0	0
LTR	MaLR	MTA_Mm	chr3	59781336	59781731	-	0	0	0
Simple_repeat	Simple_repeat	(A)n	chr3	60541979	60542018	+	0	0	0
LTR	MaLR	MTA_Mm	chr3	61042411	61042805	-	0	0	0
LTR	MaLR	MTA_Mm	chr3	61043900	61044294	-	0	0	0
Simple_repeat	Simple_repeat	(A)n	chr3	61223706	61223748	+	0.591053	0	0
Simple_repeat	Simple_repeat	(A)n	chr3	61472475	61472509	+	0	0	0
LTR	MaLR	MTA_Mm	chr3	62439590	62439985	+	0	0	0.342623
SINE	Alu	B1_Mus2	chr3	62725129	62725271	-	0	0	0
SINE	Alu	B1_Mus2	chr3	62911832	62911978	-	0	0	0
Simple_repeat	Simple_repeat	(A)n	chr3	63666743	63666769	+	0	0	0
Simple_repeat	Simple_repeat	(A)n	chr3	64292471	64292553	+	0	0	0
Simple_repeat	Simple_repeat	(A)n	chr3	64370740	64370783	+	0.332194	0	0
Simple_repeat	Simple_repeat	(A)n	chr3	65385408	65385444	+	0	0	0
SINE	Alu	B1_Mur3	chr3	65822832	65822982	+	0	0	0
Simple_repeat	Simple_repeat	(A)n	chr3	65822983	65823006	+	0	0	0
Simple_repeat	Simple_repeat	(A)n	chr3	66406979	66407005	+	0	0	0
SINE	Alu	B1_Mus2	chr3	67673916	67674063	-	0	0	0
SINE	Alu	B1_Mm	chr3	68517389	68517505	+	0	0	0
Simple_repeat	Simple_repeat	(A)n	chr3	68517506	68517531	+	0	0	0
SINE	Alu	B1_Mus2	chr3	68603190	68603335	+	0	0	0
Simple_repeat	Simple_repeat	(A)n	chr3	68603336	68603362	+	0	0	0
LTR	MaLR	MTA_Mm	chr3	68636270	68636665	+	0	0	0
SINE	Alu	B1_Mur3	chr3	68668723	68668863	-	1.85701	0	0
Simple_repeat	Simple_repeat	(A)n	chr3	68864467	68864493	+	0	0	0
Low_complexity	Low_complexity	A-rich	chr3	69598593	69598712	+	0	0	0
LTR	MaLR	MTA_Mm	chr3	69804905	69805299	+	0	0	0.957947
LTR	MaLR	MTA_Mm	chr11	22205745	22206141	+	0	0	0
LTR	MaLR	MTA_Mm	chr3	70250824	70251217	-	0	0	0
LTR	MaLR	MTA_Mm	chr3	70518923	70519314	-	0	0	0
SINE	Alu	B1_Mus2	chr3	70697709	70697855	-	0	0	0
LTR	MaLR	MTA_Mm	chr11	22339591	22339985	-	0	0	0
Simple_repeat	Simple_repeat	(A)n	chr3	71437018	71437057	+	0	0	0
Simple_repeat	Simple_repeat	(A)n	chr3	71462058	71462086	+	0	0	0
LINE	L1	L1Md_F2	chr3	71481041	71486238	+	0	0	0
LINE	L1	L1Md_F	chr3	71547793	71553709	-	0	0	0
SINE	Alu	B1_Mus1	chr3	72260310	72260457	+	0	0	0
Simple_repeat	Simple_repeat	(A)n	chr3	72260458	72260487	+	0	0	0
SINE	Alu	B1_Mus1	chr3	72309699	72309858	+	0	0	0
Simple_repeat	Simple_repeat	(A)n	chr3	72309859	72309966	+	0	0	0
SINE	Alu	B1_Mm	chr3	72344232	72344381	-	0	0	0
SINE	Alu	B1_Mm	chr11	22423833	22423979	-	0	0	0
Simple_repeat	Simple_repeat	(A)n	chr3	72636435	72636463	+	0	0	0
SINE	Alu	B1_Mm	chr11	22493129	22493275	+	0	0	0
Simple_repeat	Simple_repeat	(A)n	chr11	22509075	22509113	+	0	0	0
SINE	Alu	B1_Mur1	chr11	22572779	22572916	+	0	0	0
Simple_repeat	Simple_repeat	(A)n	chr11	22572917	22572950	+	0	0	0
SINE	Alu	B1_Mus2	chr3	74512229	74512368	+	0	0	0
Simple_repeat	Simple_repeat	(A)n	chr3	74512369	74512409	+	0	0	0
LTR	MaLR	MTA_Mm	chr11	22600151	22600545	-	0	0	0
Simple_repeat	Simple_repeat	(A)n	chr3	74786161	74786211	+	0	0.708814	0
Simple_repeat	Simple_repeat	(A)n	chr3	75042640	75042696	+	0	0	0
SINE	Alu	B1_Mus2	chr3	75296636	75296781	-	0	0	0
Simple_repeat	Simple_repeat	(A)n	chr3	76740180	76740228	+	0	0	0
Simple_repeat	Simple_repeat	(A)n	chr3	76983842	76983885	+	0	0	0
LINE	L1	L1Md_T	chr3	76987954	76994367	-	0	0	0
Simple_repeat	Simple_repeat	(A)n	chr3	77031435	77031477	+	0	0	0
Simple_repeat	Simple_repeat	(A)n	chr3	77079903	77079933	+	0	0	0

LTR	MaLR	MTA_Mm	chr3	77355528	77355926	+	0	0	0
LTR	MaLR	MTA_Mm	chr3	77773004	77773399	+	0	0	0
LTR	MaLR	MTA_Mm	chr3	77924322	77924717	-	0	0	0
Simple_repeat	Simple_repeat	(A)n	chr3	78066947	78066982	+	0	0	0
Simple_repeat	Simple_repeat	(A)n	chr3	78312684	78312723	+	0	0	0
LINE	L1	L1Md_F3	chr3	78370846	78376348	+	0	0	0
Simple_repeat	Simple_repeat	(A)n	chr3	78425518	78425546	+	0	0	0
SINE	Alu	B1_Mus1	chr3	78573176	78573323	-	0	0	0.342623
LTR	MaLR	MTA_Mm	chr3	78955450	78955845	+	0	0	0
SINE	Alu	B1_Mus1	chr3	79759513	79759660	+	0.591053	0	0
Simple_repeat	Simple_repeat	(A)n	chr3	79759661	79759686	+	0.591053	0	0
LTR	MaLR	ORR1B2	chr3	79930865	79931157	-	0	0	0
LINE	L1	L1Md_F2	chr3	79947917	79954042	-	0	0	0
LTR	MaLR	MTA_Mm	chr3	80238168	80238563	-	0	0	0
LTR	MaLR	MTA_Mm	chr3	80325463	80325857	-	0	0	0
LTR	MaLR	MTA_Mm	chr3	80326953	80327347	-	0	0	0.342623
Simple_repeat	Simple_repeat	(A)n	chr3	80741004	80741047	+	0	0	0
Simple_repeat	Simple_repeat	(A)n	chr3	81050701	81050731	+	0.332194	0	0
LINE	L1	L1_Mus4	chr3	81315832	81321762	-	0	0	0
Simple_repeat	Simple_repeat	(A)n	chr3	82298085	82298118	+	0	0	0
LTR	MaLR	MTA_Mm	chr3	82568375	82568768	+	0	0	0
SINE	Alu	B1_Mm	chr3	83020239	83020384	+	0	0	0
Simple_repeat	Simple_repeat	(A)n	chr3	83020385	83020408	+	0.591053	0	0
Simple_repeat	Simple_repeat	(A)n	chr3	83206164	83206234	+	0	0	0.342623
SINE	Alu	B1_Mus2	chr3	84451988	84452133	+	0	0	0
Simple_repeat	Simple_repeat	(A)n	chr3	84452134	84452161	+	0	0	0
Simple_repeat	Simple_repeat	(A)n	chr3	84589292	84589325	+	0	0	0
Simple_repeat	Simple_repeat	(A)n	chr3	85312988	85313026	+	0	0	0
SINE	B4	B4A	chr3	85359870	85360177	-	3.15698	0	0
Simple_repeat	Simple_repeat	(A)n	chr3	85582048	85582101	+	0	0	0
SINE	Alu	B1_Mm	chr3	85745970	85746116	-	0	0	0
SINE	Alu	B1_Mus1	chr3	85790414	85790561	+	1.77316	0	0
Simple_repeat	Simple_repeat	(A)n	chr3	85790562	85790586	+	1.77316	0	0
SINE	Alu	B1_Mus2	chr3	85967221	85967357	-	0	0	0
SINE	Alu	B1_Mus2	chr3	86799109	86799255	+	0	0	0
Simple_repeat	Simple_repeat	(A)n	chr3	86799256	86799284	+	0	0	0
SINE	Alu	B1_Mur4	chr3	87690071	87690217	+	0	0.380568	0.342623
Simple_repeat	Simple_repeat	(A)n	chr3	87690218	87690247	+	0	0.380568	0.342623
SINE	B2	B2_Mm1a	chr3	87761374	87761561	+	0	0	0
SINE	Alu	B1_Mm	chr3	87818182	87818328	-	0	0	0
SINE	B2	B2_Mm1a	chr11	23402039	23402232	+	0	0	0
Simple_repeat	Simple_repeat	(A)n	chr11	23402233	23402262	+	0	0	0.342623
SINE	Alu	B1_Mus2	chr11	23407488	23407634	+	0	0	0
Simple_repeat	Simple_repeat	(A)n	chr11	23407635	23407662	+	0	0	0
LTR	MaLR	MTA_Mm	chr3	88071665	88072046	-	0	0	0
SINE	Alu	B1_Mm	chr11	23416000	23416145	-	0	0	0
SINE	Alu	B1_Mus1	chr3	88282921	88283065	-	0	0	0
SINE	Alu	B1_Mm	chr3	88378663	88378809	-	0	0	0
Simple_repeat	Simple_repeat	(TTTA)n	chr3	88414309	88414339	+	0	0	0
SINE	Alu	B1_Mus2	chr3	88760857	88760989	-	0	0	0
SINE	Alu	B1_Mus1	chr3	88886516	88886660	-	0	0	0
Simple_repeat	Simple_repeat	(A)n	chr3	88916988	88917017	+	0	0	0
SINE	Alu	B1_Mus2	chr3	89004305	89004451	-	0	0	0
SINE	B2	B2_Mm1a	chr3	89259677	89259834	-	0	0	0
SINE	Alu	B1_Mus2	chr3	89290497	89290613	-	0	0	0
SINE	Alu	B1_Mus1	chr3	89496393	89496533	+	0	0	0
Simple_repeat	Simple_repeat	(A)n	chr3	89496534	89496555	+	0	0	0
SINE	Alu	B1_Mm	chr3	89787240	89787386	+	0	0	0
Simple_repeat	Simple_repeat	(A)n	chr3	89787387	89787437	+	0	0	0.342623
SINE	Alu	B1_Mm	chr3	90023725	90023878	-	0	0	0
SINE	Alu	B1_Mus2	chr3	90087665	90087810	+	0	0	0
SINE	Alu	B1_Mm	chr3	90091530	90091679	-	0	0	0
SINE	Alu	B1_Mus2	chr3	90093301	90093446	+	0	0	0
Simple_repeat	Simple_repeat	(A)n	chr3	90093447	90093479	+	0	0	0
SINE	Alu	B1_Mus1	chr3	90251584	90251730	+	0	0	0
Simple_repeat	Simple_repeat	(A)n	chr3	90251731	90251759	+	0	0	0
SINE	Alu	B1_Mus2	chr3	90274991	90275137	+	0	0	0

Simple_repeat	Simple_repeat	(A)n	chr3	90275138	90275169	+	0	0	0
SINE	Alu	B1_Mus2	chr3	90279296	90279448	+	0	0	0
LTR	MaLR	MTA_Mm	chr11	23689081	23689480	+	0	0	0
Simple_repeat	Simple_repeat	(A)n	chr3	90663576	90663611	+	0	0	0
LINE	L1	L1Md_F3	chr3	90971295	90975562	+	0	0	0.342623
SINE	Alu	B1_Mm	chr11	23719512	23719657	-	0	0	0
LINE	L1	L1_Mus2	chr3	91279558	91285114	-	0	0	0
LINE	L1	L1Md_F2	chr3	91459705	91465170	+	0	0	0
LINE	L1	L1Md_F2	chr3	91559343	91564778	+	0	0	0
LINE	L1	L1_Mus1	chr3	91721211	91726913	-	0	0	0
Simple_repeat	Simple_repeat	(A)n	chr3	91808850	91808874	+	0	0	0
Simple_repeat	Simple_repeat	(A)n	chr3	91818084	91818124	+	0	0	0
Simple_repeat	Simple_repeat	(A)n	chr3	92374727	92374763	+	0	0	0
LTR	MaLR	MTA_Mm	chr3	92602954	92603348	-	0	0	0
LTR	MaLR	MTA_Mm	chr3	93104132	93104526	+	0	0	0
SINE	Alu	B1_Mm	chr3	93253193	93253338	+	0	0	0
Simple_repeat	Simple_repeat	(A)n	chr3	93253339	93253362	+	0	0	0
Simple_repeat	Simple_repeat	(A)n	chr11	23906926	23906967	+	0	0	0
SINE	Alu	B1_Mus2	chr3	93287123	93287268	-	0	0	0
SINE	Alu	B1_Mus1	chr3	94086204	94086321	-	0	0	0
SINE	Alu	B1_Mus2	chr3	94395714	94395860	-	0	0	0
SINE	Alu	B1_Mus1	chr3	94412768	94412900	-	0	0	0
SINE	Alu	B1_Mus1	chr3	94526435	94526581	+	0	0	0
Simple_repeat	Simple_repeat	(A)n	chr3	94526582	94526606	+	0	0	0
LTR	MaLR	MTA_Mm	chr3	94861273	94861668	+	0	0	0
SINE	Alu	B1_Mus1	chr3	95112253	95112400	-	0	0	0
SINE	Alu	B1_Mm	chr3	95230785	95230932	-	0	0	0
SINE	Alu	B1_Mur2	chr3	95315534	95315673	+	0	0.380568	0
Simple_repeat	Simple_repeat	(A)n	chr3	95315674	95315699	+	0	0.380568	0
SINE	Alu	B1_Mus1	chr3	95524682	95524830	-	0	0	0
SINE	Alu	B1_Mus1	chr3	95630185	95630328	+	0	0	0
Simple_repeat	Simple_repeat	(A)n	chr3	95630329	95630367	+	0	0	0
SINE	Alu	B1_Mus1	chr3	95764019	95764156	+	0	0	0
Simple_repeat	Simple_repeat	(A)n	chr3	95764157	95764196	+	0	0	0
Simple_repeat	Simple_repeat	(TTTA)n	chr3	96021706	96021757	+	0	0	0
SINE	Alu	B1_Mm	chr3	96356292	96356438	+	0	0	0
Simple_repeat	Simple_repeat	(A)n	chr3	96356439	96356473	+	0	0	0
SINE	B2	B2_Mm1t	chr3	96889878	96890071	+	0	0	0
SINE	Alu	B1_Mus2	chr3	96926642	96926788	+	0	0	0
SINE	Alu	B1_Mm	chr3	96943885	96944030	+	0	0	0
Simple_repeat	Simple_repeat	(A)n	chr3	96944031	96944080	+	0	0	0
Simple_repeat	Simple_repeat	(A)n	chr3	97046455	97046483	+	0	0	0
SINE	Alu	B1_Mus2	chr3	97163963	97164108	-	0	0	0
SINE	Alu	B1_Mm	chr3	97421293	97421407	-	0	0	0
LINE	L1	L1Md_T	chr11	24775660	24782253	-	0	0	0
SINE	Alu	B1_Mm	chr3	97997970	97998115	-	0	0	0
SINE	Alu	B1_Mm	chr3	98146680	98146826	+	0	0	0
Simple_repeat	Simple_repeat	(A)n	chr3	98146827	98146854	+	0	0	0
SINE	Alu	B1_Mus2	chr3	98163720	98163866	+	0	0	0
Simple_repeat	Simple_repeat	(A)n	chr3	98163867	98163888	+	0	0	0
Simple_repeat	Simple_repeat	(A)n	chr11	24851086	24851114	+	0	0.380568	0
Simple_repeat	Simple_repeat	(A)n	chr3	99043110	99043136	+	0	0	0
LINE	L1	L1Md_F	chr3	99170363	99175652	+	0	0	0
LINE	L1	L1Md_F2	chr3	99410060	99416420	-	0	0	0
SINE	Alu	PB1D9	chr3	10051845	10051857	+	0	0	0
Simple_repeat	Simple_repeat	(A)n	chr3	10051857	10051861	+	0	0	0
LTR	MaLR	MTA_Mm	chr3	10056637	10056676	+	0	0	0
LTR	MaLR	MTA_Mm	chr3	10084335	10084372	-	0	0	0
SINE	Alu	B1_Mm	chr3	10183070	10183085	+	0	0	0
Simple_repeat	Simple_repeat	(A)n	chr3	10183085	10183091	+	0	0	0
SINE	Alu	B1_Mus2	chr3	10188406	10188421	-	0	0	0
Simple_repeat	Simple_repeat	(A)n	chr3	10305795	10305798	+	0	0	0
LTR	MaLR	MTA_Mm	chr3	10349632	10349672	-	0	0	0
SINE	Alu	B1_Mm	chr3	10350811	10350825	+	0	0	0
Simple_repeat	Simple_repeat	(A)n	chr3	10350826	10350829	+	0	0	0
Simple_repeat	Simple_repeat	(A)n	chr3	10381285	10381287	+	0.332194	0	0
Simple_repeat	Simple_repeat	(A)n	chr3	10414024	10414027	+	0	0.380568	0

SINE	Alu	B1_Mus2	chr3	10434372	10434387	-	0.332194	0	0
SINE	Alu	B1_Mus2	chr3	10446882	10446897	-	0	0	0
SINE	Alu	PB1D7	chr3	10489134	10489144	+	0	0	0
Simple_repeat	Simple_repeat	(A)n	chr3	10489144	10489147	+	0	0	0
LTR	MaLR	MTA_Mm	chr3	10498754	10498794	-	0	0	0
SINE	Alu	B1_Mur4	chr3	10549318	10549333	+	0	0	0
SINE	Alu	B1_Mus2	chr3	10563959	10563973	-	0	0	0.342623
Simple_repeat	Simple_repeat	(A)n	chr3	10565222	10565225	+	0	0	0
Simple_repeat	Simple_repeat	(A)n	chr3	10573919	10573923	+	0	0	0
LTR	MaLR	MTA_Mm	chr3	10646522	10646561	-	0	0	0
LTR	MaLR	MTA_Mm	chr3	10646667	10646706	-	0	0	0
SINE	Alu	B1_Mus1	chr3	10714247	10714262	-	0.332194	0	0
LTR	MaLR	MTA_Mm	chr11	26141137	26141531	-	0	0	0
SINE	Alu	B1_Mm	chr3	10771508	10771520	-	0	0	0
Simple_repeat	Simple_repeat	(TTTA)n	chr3	10781115	10781119	+	0	0	0
SINE	Alu	B1_Mus2	chr3	10804344	10804359	+	0	0	0
Simple_repeat	Simple_repeat	(A)n	chr3	10804359	10804362	+	0	0	0
SINE	Alu	B1_Mus1	chr3	10819293	10819307	+	0	0	0
Simple_repeat	Simple_repeat	(A)n	chr3	10819307	10819315	+	0	0	0
LTR	MaLR	MTA_Mm	chr3	11026376	11026415	+	0	0	0
LINE	L1	L1Md_F2	chr3	11063086	11063701	-	0	0	0
SINE	Alu	B1_Mus1	chr3	11080280	11080294	+	0	0	0
Simple_repeat	Simple_repeat	(A)n	chr3	11080295	11080297	+	0	0	0
SINE	Alu	B1_Mm	chr3	11089918	11089933	+	0	0	0
Simple_repeat	Simple_repeat	(A)n	chr3	11089933	11089936	+	0	0	0
LTR	MaLR	MTA_Mm	chr3	11091517	11091557	+	0	0	0
LTR	MaLR	MTA_Mm	chr3	11091663	11091702	+	0	0	0
LTR	MaLR	MTA_Mm	chr11	26562613	26562945	-	0	0	0
Simple_repeat	Simple_repeat	(A)n	chr3	11138073	11138078	+	0	0	0
LTR	MaLR	MTA_Mm	chr3	11194867	11194906	+	0	0	0
SINE	Alu	B1_Mm	chr11	26677502	26677648	-	0	0	0
LTR	MaLR	MTA_Mm	chr3	11227044	11227083	+	0	0	0
LINE	L1	L1_Mus2	chr3	11233136	11233587	-	0	0	0
LINE	L1	L1Md_F2	chr3	11233620	11234156	+	0	0	0
LINE	L1	Lx2	chr3	11280539	11281039	+	0	0	0
LTR	MaLR	MTA_Mm	chr3	11283115	11283154	-	0	0	0
SINE	Alu	B1_Mus2	chr11	26755993	26756139	+	0	0	0
Simple_repeat	Simple_repeat	(A)n	chr11	26756140	26756176	+	0	0	0
LTR	MaLR	MTA_Mm	chr3	11407001	11407041	-	0	0	0.615324
Simple_repeat	Simple_repeat	(GAAA)n	chr3	11422482	11422492	+	0	0	0
LTR	MaLR	MTA_Mm	chr3	11496681	11496720	+	0	0	0.342623
LINE	L1	L1Md_T	chr3	11512345	11512881	+	0	0	0
Simple_repeat	Simple_repeat	(A)n	chr3	11520039	11520043	+	0	0	0
Simple_repeat	Simple_repeat	(A)n	chr3	11525787	11525791	+	0	0	0
SINE	Alu	B1_Mus1	chr3	11555645	11555659	-	0	0	0
SINE	Alu	B1_Mus2	chr3	11576718	11576733	+	0	0	0
Simple_repeat	Simple_repeat	(A)n	chr3	11576733	11576736	+	0	0	0
LTR	MaLR	MTA_Mm	chr3	11576899	11576939	+	0	0	0
Simple_repeat	Simple_repeat	(A)n	chr11	27088440	27088482	+	0	0	0
LTR	MaLR	MTA_Mm	chr3	11583851	11583891	+	0	0	0.342623
Simple_repeat	Simple_repeat	(A)n	chr3	11596080	11596083	+	0	0	0
LINE	L1	L1Md_T	chr11	27282597	27289454	-	0	0	0
Simple_repeat	Simple_repeat	(A)n	chr3	11782868	11782872	+	0	0	0
Simple_repeat	Simple_repeat	(A)n	chr3	11783752	11783756	+	0	0	0
Simple_repeat	Simple_repeat	(A)n	chr3	11788897	11788902	+	0	0	0
SINE	Alu	B1_Mur4	chr3	11918661	11918673	-	0.591053	0	0
Simple_repeat	Simple_repeat	(A)n	chr3	11920994	11920997	+	0	0	0
Simple_repeat	Simple_repeat	(A)n	chr3	11938772	11938776	+	0	0	0
Simple_repeat	Simple_repeat	(A)n	chr3	11988289	11988292	+	0	0	0
LTR	MaLR	MTA_Mm	chr3	12003681	12003721	+	0	0	0
Simple_repeat	Simple_repeat	(A)n	chr3	12027021	12027024	+	0	0	0
Simple_repeat	Simple_repeat	(A)n	chr3	12049579	12049582	+	0	0	0
Simple_repeat	Simple_repeat	(A)n	chr3	12053758	12053762	+	0	0	0
Simple_repeat	Simple_repeat	(GAAAA)n	chr3	12059692	12059700	+	0	0	0
Simple_repeat	Simple_repeat	(A)n	chr3	12065403	12065406	+	0	0	0
LTR	MaLR	MTA_Mm	chr3	12089977	12090016	-	0	0	0
SINE	B2	B2_Mm1a	chr3	12126736	12126756	+	0	0	0

LTR	MaLR	MTB	chr10	4726252	4726647	-	0	0	0
Simple_repeat	Simple_repeat	(A)n	chr3	12197672	12197675	+	0	0	0.342623
SINE	Alu	B1_Mus1	chr3	12239103	12239117	-	0	0	0
SINE	B2	B2_Mm1t	chr3	12263111	12263129	-	0	0	0
SINE	Alu	B1_Mus1	chr3	12269834	12269849	-	0	0	0
Simple_repeat	Simple_repeat	(GAAA)n	chr11	27956130	27956231	+	0	0	0
LTR	MaLR	MTA_Mm	chr3	12387264	12387304	+	0	0	0
SINE	Alu	B1_Mus1	chr3	12415655	12415671	+	0	0	0
Simple_repeat	Simple_repeat	(A)n	chr3	12415671	12415673	+	0	0	0
SINE	Alu	B1_Mus2	chr3	12419654	12419669	-	0	0.380568	0
Simple_repeat	Simple_repeat	(GAAA)n	chr3	12500902	12500911	+	0	0	0
LINE	L1	L1Md_F2	chr3	12507730	12508327	-	0	0	0
SINE	Alu	B1_Mus1	chr3	12554561	12554575	+	0	0	0
Simple_repeat	Simple_repeat	(A)n	chr3	12554575	12554578	+	0.332194	0	0
LTR	MaLR	MTA_Mm	chr3	12582853	12582891	-	0	0	0
SINE	Alu	B1_Mus2	chr3	12660619	12660633	+	0	0	0
Simple_repeat	Simple_repeat	(A)n	chr3	12660633	12660636	+	0	0	0
Simple_repeat	Simple_repeat	(A)n	chr3	12684337	12684341	+	0	0	0
SINE	Alu	B1_Mm	chr3	12691754	12691769	+	0	0	0
Simple_repeat	Simple_repeat	(A)n	chr3	12691769	12691771	+	0	0	0
SINE	Alu	B1_Mm	chr3	12692420	12692434	-	2.89812	1.85052	5.26521
Simple_repeat	Simple_repeat	(A)n	chr3	12692824	12692828	+	0.591053	0	0
Simple_repeat	Simple_repeat	(A)n	chr3	12696025	12696028	+	0	0	0
SINE	B2	B2_Mm1a	chr3	12714560	12714579	+	0	0	0
SINE	Alu	B1_Mm	chr3	12722940	12722954	-	0	0	0
Simple_repeat	Simple_repeat	(A)n	chr3	12796103	12796105	+	0	0	0
Simple_repeat	Simple_repeat	(A)n	chr3	12813499	12813504	+	0	0	0
Simple_repeat	Simple_repeat	(A)n	chr11	28610173	28610202	+	0	0	0
SINE	Alu	B1_Mus1	chr3	12827113	12827127	-	0	0	0
SINE	Alu	B1_Mm	chr3	12830543	12830557	-	0	0	0
SINE	Alu	B1_Mus2	chr3	12833169	12833184	+	0	0	0
Simple_repeat	Simple_repeat	(A)n	chr3	12833184	12833188	+	0.591053	0	0
Simple_repeat	Simple_repeat	(A)n	chr3	12844478	12844482	+	0	0	0
SINE	Alu	B1_Mus2	chr3	12868165	12868180	-	0	0	0
SINE	Alu	B1_Mus2	chr3	12871180	12871194	+	0	0	0
Simple_repeat	Simple_repeat	(A)n	chr3	12871195	12871200	+	0	0	0
SINE	Alu	B1_Mm	chr3	12912458	12912472	-	0	0	0
Simple_repeat	Simple_repeat	(A)n	chr10	19229015	19229043	+	0	0	0
LTR	MaLR	MTB	chr3	12947980	12948019	-	0	0	0
Simple_repeat	Simple_repeat	(A)n	chr11	28856085	28856119	+	0	0	0
SINE	Alu	B1_Mus1	chr3	13043430	13043445	+	0.591053	0	0
Simple_repeat	Simple_repeat	(A)n	chr3	13043445	13043450	+	0.591053	0	0
SINE	Alu	B1_Mus2	chr3	13043610	13043624	+	0	0	0
Simple_repeat	Simple_repeat	(A)n	chr3	13043625	13043627	+	0	0	0
Simple_repeat	Simple_repeat	(A)n	chr3	13051636	13051644	+	0	0	0
SINE	Alu	B1_Mus1	chr3	13061362	13061378	-	0	0	0
Simple_repeat	Simple_repeat	(A)n	chr3	13065879	13065884	+	0	0	0.342623
LINE	L1	L1Md_T	chr3	13136962	13137490	+	0	0	0
SINE	Alu	B1_Mm	chr3	13169254	13169268	-	0	0	0
Simple_repeat	Simple_repeat	(A)n	chr3	13193610	13193613	+	0	0	0
SINE	Alu	B1_Mus1	chr3	13231687	13231701	-	0	0	0
SINE	Alu	B1_Mm	chr3	13250226	13250240	-	0	0	0
Simple_repeat	Simple_repeat	(A)n	chr3	13294923	13294929	+	0	0	0
Simple_repeat	Simple_repeat	(A)n	chr3	13322054	13322057	+	0	0	0
Low_complexity	Low_complexity	AT_rich	chr3	13411876	13411880	+	0	0	0
SINE	Alu	B1_Mus2	chr3	13449106	13449121	+	0	0	0
Simple_repeat	Simple_repeat	(A)n	chr3	13449121	13449123	+	0	0	0
Simple_repeat	Simple_repeat	(A)n	chr3	13470858	13470861	+	0	0	0
SINE	Alu	B1_Mm	chr10	19287667	19287813	+	0	0	0
Simple_repeat	Simple_repeat	(A)n	chr10	19287814	19287838	+	0	0	0
SINE	Alu	B1_Mm	chr3	13548560	13548573	-	0	0	0
SINE	Alu	PB1D9	chr3	13620480	13620491	+	0	0	0
Simple_repeat	Simple_repeat	(A)n	chr3	13620491	13620493	+	0	0	0
SINE	Alu	B1_Mus2	chr3	13691754	13691769	-	0	0	0
SINE	Alu	B1_Mm	chr11	29497558	29497704	+	0	0	0
SINE	Alu	B1_Mus1	chr3	13752482	13752494	+	0	0	0
Simple_repeat	Simple_repeat	(A)n	chr3	13752495	13752497	+	0	0	0

Simple_repeat	Simple_repeat	(A)n	chr3	13766074	13766078	+	0	0	0
SINE	B2	B2_Mm1a	chr11	29566743	29566934	+	0	0	0
LTR	MaLR	MTA_Mm	chr3	13875111	13875149	+	0	0	0
SINE	Alu	B1_Mm	chr3	14092979	14092993	-	0	0	0
SINE	Alu	B1_Mus1	chr3	14100428	14100443	+	0	0	0
Low_complexity	Low_complexity	A-rich	chr3	14100443	14100454	+	0	0	0
LTR	MaLR	MTA_Mm	chr3	14184006	14184045	+	0	0	0.615324
Simple_repeat	Simple_repeat	(A)n	chr3	14184241	14184245	+	0	0	0
LTR	MaLR	MTA_Mm	chr3	14272344	14272383	-	0	0	0
Simple_repeat	Simple_repeat	(A)n	chr3	14377402	14377406	+	0	0	0
Simple_repeat	Simple_repeat	(A)n	chr3	14379113	14379117	+	0	0	0
Simple_repeat	Simple_repeat	(A)n	chr3	14398471	14398475	+	0	0	0
SINE	Alu	B1_Mus2	chr3	14431410	14431424	-	0	0	0
Simple_repeat	Simple_repeat	(A)n	chr3	14442605	14442608	+	0	0	0
SINE	Alu	B1_Mus1	chr3	14521735	14521749	+	0	0	0
Simple_repeat	Simple_repeat	(A)n	chr3	14521749	14521752	+	0	0	0
SINE	Alu	B1_Mus2	chr3	14587986	14588000	-	0	0	0
SINE	Alu	B1_Mus2	chr3	14618538	14618553	-	0	0	0
SINE	Alu	B1_Mus1	chr3	14686551	14686566	-	0	0	0
Simple_repeat	Simple_repeat	(A)n	chr3	14833253	14833257	+	0	0	0
Simple_repeat	Simple_repeat	(A)n	chr3	14875745	14875749	+	0	0	0
Simple_repeat	Simple_repeat	(A)n	chr3	14916852	14916857	+	0	0	0
SINE	B2	B2_Mm1t	chr3	14916965	14916984	-	0	0	0
Simple_repeat	Simple_repeat	(A)n	chr3	14917351	14917358	+	0	0	0
Simple_repeat	Simple_repeat	(A)n	chr3	14925801	14925804	+	0	0	0
SINE	Alu	B1_Mus2	chr3	14933073	14933087	-	0	0	0
SINE	Alu	B1_Mm	chr3	15034674	15034689	+	0	0	0
Simple_repeat	Simple_repeat	(A)n	chr3	15175324	15175328	+	0	0	0
LTR	MaLR	MTA_Mm	chr3	15281239	15281278	+	0	0	0
LTR	MaLR	MTA_Mm	chr3	15341545	15341584	+	0	0	0.342623
SINE	B2	B2_Mm1a	chr3	15350215	15350234	+	0	0	0
LTR	MaLR	MTB	chr3	15409632	15409672	+	0	0	0
LTR	MaLR	MTB_Mm	chr3	15409850	15409891	-	0	0	0
LTR	MaLR	MTA_Mm	chr3	15444842	15444882	+	0	0	0
SINE	Alu	B1_Mus1	chr11	31093673	31093821	+	0	0	0
Simple_repeat	Simple_repeat	(A)n	chr11	31093822	31093846	+	0	0	0
SINE	Alu	B1_Mm	chr3	15514032	15514044	+	0	0	0
Simple_repeat	Simple_repeat	(A)n	chr3	15514044	15514048	+	0	0	0
Simple_repeat	Simple_repeat	(A)n	chr3	15523969	15523973	+	0	0	0
Simple_repeat	Simple_repeat	(A)n	chr3	15542001	15542005	+	0	0	0
SINE	Alu	B1_Mm	chr3	15748203	15748218	+	0	0	0
LTR	MaLR	MTA_Mm	chr11	31323251	31323647	+	0	0	0
Simple_repeat	Simple_repeat	(A)n	chr3	15760431	15760434	+	0	0	0
LINE	L1	L1_Mus2	chr3	15826022	15826480	-	0	0	0
LTR	MaLR	MTA_Mm	chr3	15856558	15856590	+	0.591053	0	0
SINE	Alu	B1_Mus2	chr3	15929101	15929116	-	0	0	0
LTR	MaLR	MTA_Mm	chr4	3014062	3014455	-	0	0	0
SINE	Alu	B1_Mus1	chr4	3073799	3073918	-	0	0	0
SINE	Alu	B1_Mus2	chr4	3378663	3378798	-	0	0	0
SINE	Alu	B1_Mus2	chr4	3569715	3569861	-	0	0	0
SINE	Alu	B1_Mus2	chr4	3720228	3720373	-	0	0	0.615324
SINE	Alu	B1_Mus1	chr4	3736676	3736821	+	0.332194	0	0
Simple_repeat	Simple_repeat	(A)n	chr4	3736822	3736855	+	0.332194	0	0.615324
SINE	Alu	B1_Mus2	chr4	3781228	3781373	-	0	0	0
Simple_repeat	Simple_repeat	(A)n	chr4	5350835	5350860	+	0	0	0.342623
LINE	L1	L1VL4	chr4	5938064	5941310	-	0	0	0
SINE	Alu	B1_Mus1	chr4	6164096	6164243	+	0	0	0
Simple_repeat	Simple_repeat	(A)n	chr4	6164244	6164268	+	0	0	0
Simple_repeat	Simple_repeat	(A)n	chr4	6234245	6234298	+	0	0	0
SINE	Alu	B1_Mm	chr4	6398368	6398488	+	0	0	0
Simple_repeat	Simple_repeat	(A)n	chr4	6398489	6398533	+	0	0	0
SINE	Alu	B1_Mus2	chr4	6452102	6452248	-	0	0	0
LTR	MaLR	MTA_Mm	chr4	6478145	6478544	+	0	0	0
LTR	MaLR	MTA_Mm	chr4	6479640	6480039	+	0	0	0
SINE	Alu	B1_Mur1	chr4	7107751	7107889	-	0	0	0
SINE	Alu	B1_Mm	chr4	7146176	7146325	-	0	0	0
Simple_repeat	Simple_repeat	(A)n	chr4	7382583	7382614	+	0	0	0

LTR	MaLR	MTA_Mm	chr4	7446578	7446966	-	0	0	0
Simple_repeat	Simple_repeat	(A)n	chr4	7825165	7825215	+	0	0	0
SINE	Alu	B1_Mm	chr4	7888196	7888329	-	0	0	0
SINE	Alu	B1_Mus2	chr4	8026375	8026523	-	0	0	0
LTR	MaLR	MTA_Mm	chr4	8440128	8440523	-	0	0	0
LTR	MaLR	MTA_Mm	chr4	8441621	8442016	-	0	0	0
Simple_repeat	Simple_repeat	(A)n	chr4	8442877	8442903	+	0	0	0
Simple_repeat	Simple_repeat	(A)n	chr4	8569975	8569997	+	0	0	0
Simple_repeat	Simple_repeat	(A)n	chr4	8611681	8611702	+	0	0	0
SINE	Alu	B1_Mus2	chr11	32024718	32024865	+	0	0	0
Simple_repeat	Simple_repeat	(A)n	chr11	32024866	32024889	+	0	0	0
LTR	MaLR	MTEa	chr4	8896206	8896583	-	0	0	0
SINE	Alu	PB1D9	chr4	9047076	9047193	+	0	0	0
Simple_repeat	Simple_repeat	(A)n	chr4	9047194	9047228	+	0	0	0
SINE	Alu	B1_Mm	chr4	9608727	9608873	+	0	0	0
Simple_repeat	Simple_repeat	(A)n	chr4	9608874	9608897	+	0	0	0
LTR	MaLR	MTA_Mm	chr4	9694928	9695323	-	0	0	0
LINE	L1	L1_Mus1	chr4	10055682	10061390	-	0	0	0
LTR	MaLR	MTA_Mm	chr4	10229581	10229962	-	0	0	0
LTR	MaLR	MTA_Mm	chr4	10231059	10231454	-	0	0	0
Simple_repeat	Simple_repeat	(A)n	chr4	10800300	10800338	+	0	0	0
LTR	MaLR	MTA_Mm	chr4	10885026	10885421	-	0	0	0
LTR	MaLR	MTA_Mm	chr4	10886514	10886909	-	0	0	0
Simple_repeat	Simple_repeat	(A)n	chr4	10915401	10915435	+	0	0	0
SINE	Alu	B1_Mus2	chr4	11397676	11397821	-	0	0	0
SINE	Alu	B1_Mus1	chr4	11669591	11669738	-	0	0	0
Simple_repeat	Simple_repeat	(A)n	chr4	11865134	11865163	+	0	0	0
SINE	Alu	B1_Mm	chr4	11947851	11947993	+	0	0	0
Simple_repeat	Simple_repeat	(A)n	chr4	11947994	11948021	+	0	0	0
Simple_repeat	Simple_repeat	(A)n	chr4	12680998	12681034	+	0	0	0
LINE	L1	L1_Mus2	chr4	12915295	12921364	-	0	0	0
Simple_repeat	Simple_repeat	(A)n	chr4	13361888	13361914	+	0	0	0
Simple_repeat	Simple_repeat	(A)n	chr4	13634284	13634320	+	0	0	0
SINE	Alu	B1_Mm	chr11	32428918	32429065	+	0.591053	0	0
Simple_repeat	Simple_repeat	(A)n	chr11	32429066	32429091	+	0	0	0.342623
LTR	MaLR	MTA_Mm	chr4	14000339	14000734	+	0	0	0
LTR	MaLR	MTA_Mm	chr4	14042330	14042723	+	0	0	0
SINE	Alu	B1_Mus1	chr4	14762929	14763076	+	0	0	0
LTR	MaLR	MTA_Mm	chr4	15136900	15137299	-	0	0	0
Simple_repeat	Simple_repeat	(A)n	chr4	15652389	15652450	+	0	0.708814	0
SINE	Alu	B1_Mm	chr4	16868135	16868276	+	0	0	0
Simple_repeat	Simple_repeat	(A)n	chr4	16868277	16868300	+	0	0	0
Simple_repeat	Simple_repeat	(A)n	chr4	18053266	18053324	+	0.332194	0.708814	0.957947
LTR	MaLR	MTA_Mm	chr4	18185860	18186250	-	0	0	0
LTR	MaLR	MTA_Mm	chr4	18232111	18232499	+	0	0	0
SINE	Alu	B1_Mus2	chr4	18770301	18770447	-	0	0	0
LTR	MaLR	MTA_Mm	chr4	18894286	18894680	+	0	0	0
Simple_repeat	Simple_repeat	(A)n	chr4	19704047	19704074	+	0	0	0
LINE	L1	L1Md_F2	chr4	19710823	19716403	+	0	0	0
Simple_repeat	Simple_repeat	(A)n	chr4	20753732	20753777	+	0	0	0
LTR	MaLR	MTA_Mm	chr4	21143107	21143497	+	0	0	0
LTR	MaLR	MTA_Mm	chr4	21289318	21289709	-	0	0	0
LTR	MaLR	MTA_Mm	chr4	21770608	21771007	+	0	0	0
Simple_repeat	Simple_repeat	(A)n	chr4	22627283	22627304	+	0.591053	0	0
LTR	MaLR	MTA_Mm	chr4	22708514	22708909	-	0	0	0
SINE	Alu	B1_Mm	chr11	33074335	33074480	-	0	0	0
Simple_repeat	Simple_repeat	(A)n	chr4	22761293	22761331	+	0	0	0
LTR	MaLR	MTA_Mm	chr4	22891566	22891960	+	0	0	0
LINE	L1	L1Md_T	chr4	23021702	23028342	-	0	0	0
Low_complexity	Low_complexity	A-rich	chr4	23205350	23205436	+	0	0	0
LTR	MaLR	MTA_Mm	chr4	23299286	23299680	+	0	0	0
LTR	MaLR	MTA_Mm	chr4	23429776	23430171	-	0	0	0
Simple_repeat	Simple_repeat	(A)n	chr4	23532611	23532640	+	0	0	0
Simple_repeat	Simple_repeat	(A)n	chr4	23692776	23692813	+	0	0	0
LINE	L1	L1_Mus1	chr4	23969521	23975351	-	0	0	0
LTR	MaLR	MTA_Mm	chr4	24064943	24065332	-	0	0	0
SINE	Alu	B1_Mus1	chr4	24087560	24087706	+	0.332194	0	0

Simple_repeat	Simple_repeat	(A)n	chr4	24128331	24128376	+	0	0	0
LTR	MaLR	MTA_Mm	chr4	24313510	24313905	+	0	0	0
SINE	Alu	B1_Mus2	chr4	24407120	24407266	+	0	0	0
SINE	Alu	B1_Mm	chr4	25323459	25323603	+	0	0	0
SINE	Alu	B1_Mus1	chr4	25349148	25349286	-	0	0	0
Simple_repeat	Simple_repeat	(A)n	chr4	25478843	25478910	+	0	0	0
LTR	MaLR	MTA_Mm	chr4	26972586	26972976	-	0	0	0
Simple_repeat	Simple_repeat	(A)n	chr4	27022466	27022505	+	0	0	0
Simple_repeat	Simple_repeat	(A)n	chr4	27064647	27064704	+	0	0	0
SINE	Alu	B1_Mus2	chr4	27294488	27294634	+	0	0	0
Simple_repeat	Simple_repeat	(A)n	chr4	27294635	27294697	+	0	0	0
SINE	B2	B2_Mm2	chr4	27514743	27514905	-	0	0	0
Simple_repeat	Simple_repeat	(A)n	chr4	27650342	27650371	+	0	0	0
Simple_repeat	Simple_repeat	(A)n	chr4	28093003	28093036	+	0	0	0
LTR	MaLR	MTA_Mm	chr4	28950004	28950402	+	0	0	0
SINE	Alu	B1_Mm	chr4	29564094	29564230	-	0	0	0
SINE	Alu	B1_Mus1	chr4	29620025	29620171	-	0	0	0
LINE	L1	L1Md_F2	chr4	29795118	29801437	-	0	0	0
SINE	Alu	B1_Mus1	chr4	29840790	29840935	-	0	0	0
Simple_repeat	Simple_repeat	(A)n	chr4	30382638	30382682	+	0	0	0
SINE	B2	B3A	chr4	30828775	30828972	-	0	0	0
Simple_repeat	Simple_repeat	(A)n	chr4	31468380	31468414	+	0	0	0
LTR	MaLR	MTA_Mm	chr4	31783053	31783445	-	0	0	0
SINE	Alu	B1_Mus1	chr4	32049000	32049137	-	0	0	0
SINE	Alu	B1_Mus2	chr4	32158334	32158481	-	0	0	0
Low_complexity	Low_complexity	A-rich	chr4	32742344	32742414	+	0	0	0
SINE	B2	B2_Mm1a	chr4	33063628	33063813	+	0	0	0
SINE	Alu	B1_Mm	chr4	33396534	33396680	+	0	0	0
Simple_repeat	Simple_repeat	(A)n	chr4	33396681	33396708	+	0	0	0
LTR	MaLR	MTA_Mm	chr4	33693507	33693902	+	0	0	0
LTR	MaLR	MTA_Mm	chr4	33708858	33709254	+	0	0	0
SINE	B2	B2_Mm1a	chr4	35113758	35113950	+	0	0	0
SINE	B2	B2_Mm1t	chr4	35123347	35123538	-	0	0	0
SINE	Alu	B1_Mm	chr4	35185531	35185683	+	0	0	0.615324
LTR	MaLR	MTA_Mm	chr4	35553759	35554139	+	0	0	0
LTR	ERVK	RMER6C	chr4	37877886	37878552	-	0	0	0
Simple_repeat	Simple_repeat	(A)n	chr4	37922299	37922335	+	0	0	0
LINE	L1	L1Md_F2	chr4	38161519	38166913	+	0	0	0
SINE	Alu	B1_Mm	chr4	38328185	38328331	+	0	0	0
SINE	Alu	B1_Mus2	chr4	39679015	39679160	+	0	0	0
Simple_repeat	Simple_repeat	(A)n	chr4	39679161	39679188	+	0	0	0
LTR	MaLR	MTA_Mm	chr4	39779380	39779774	+	0	0	0
LTR	MaLR	MTA_Mm	chr4	40316374	40316765	+	0	0	0
SINE	Alu	B1_Mus2	chr4	40596879	40597001	-	0	0	0
Simple_repeat	Simple_repeat	(A)n	chr4	40627381	40627447	+	0	0	0
SINE	Alu	B1_Mur2	chr4	41436960	41437108	+	0.591053	0	0
Simple_repeat	Simple_repeat	(A)n	chr4	41437109	41437132	+	0	0	0
LTR	MaLR	MTA_Mm	chr4	41696687	41697073	+	0	0	0
Simple_repeat	Simple_repeat	(A)n	chr4	42233390	42233422	+	0	0	0
LTR	MaLR	MTB	chr4	42919796	42920185	-	0	0	0
SINE	Alu	B1_Mus1	chr4	43029974	43030120	+	0	0	0
Simple_repeat	Simple_repeat	(A)n	chr4	43030121	43030143	+	0	0	0
SINE	Alu	B1_Mus1	chr4	43475439	43475586	-	0	0	0
SINE	Alu	B1_Mus2	chr4	43622574	43622703	+	0	0	0
Simple_repeat	Simple_repeat	(A)n	chr4	43622704	43622739	+	0	0	0
Simple_repeat	Simple_repeat	(A)n	chr4	43846753	43846791	+	0	0	0
Simple_repeat	Simple_repeat	(A)n	chr4	44011056	44011082	+	0	0	0
SINE	Alu	B1_Mm	chr4	44303118	44303263	-	0	0	0
SINE	B2	B2_Mm1a	chr4	44306747	44306938	-	0	0.380568	0
Simple_repeat	Simple_repeat	(A)n	chr4	44480689	44480722	+	0	0	0
LTR	MaLR	MTA_Mm	chr4	44970626	44971023	-	0	0	0
Simple_repeat	Simple_repeat	(A)n	chr4	44987617	44987649	+	0	0	0
SINE	Alu	B1_Mus1	chr4	45643159	45643305	-	0	0	0
LTR	MaLR	MTA_Mm	chr4	45672824	45673216	-	0	0	0
Simple_repeat	Simple_repeat	(A)n	chr11	35649532	35649582	+	0	0	0
LTR	MaLR	MTA_Mm	chr4	47510223	47510616	+	0	0	0
SINE	Alu	B1_Mus2	chr4	47931424	47931570	-	0	0	0

SINE	Alu	B1_Mm	chr4	48517505	48517651	+	0	0	0
SINE	Alu	B1_Mus1	chr4	48540202	48540352	+	0	0	0
Simple_repeat	Simple_repeat	(A)n	chr4	48540353	48540377	+	0	0	0
Simple_repeat	Simple_repeat	(A)n	chr4	49615940	49615966	+	0	0.708814	0
SINE	Alu	B1_Mus1	chr4	49642383	49642530	+	0	0	0
LTR	MaLR	MTA_Mm	chr4	49931280	49931674	+	0	0	0
LINE	L1	L1_Mus1	chr4	50310116	50315638	-	0	0	0
SINE	Alu	B1_Mm	chr4	51103091	51103237	-	0	0	0
Simple_repeat	Simple_repeat	(A)n	chr4	51275579	51275621	+	0	0	0
SINE	Alu	B1_Mus1	chr4	51513640	51513787	+	0	0	0
Simple_repeat	Simple_repeat	(A)n	chr4	51513788	51513872	+	0	0	0
SINE	Alu	B1_Mus1	chr4	51773017	51773164	-	0	0	0
SINE	Alu	B1_Mm	chr4	52777304	52777450	-	0	0	0
LINE	L1	L1Md_T	chr4	53399667	53405975	-	0	0	0
Simple_repeat	Simple_repeat	(A)n	chr4	53410395	53410426	+	0	0	0
SINE	Alu	B1_Mm	chr4	53952595	53952740	-	0.332194	0	0
LTR	MaLR	MTA_Mm	chr4	53978052	53978440	-	0	0	0
SINE	Alu	B1_Mus1	chr4	53984327	53984474	+	0	0	0
Simple_repeat	Simple_repeat	(A)n	chr4	53984475	53984499	+	0	0	0
Simple_repeat	Simple_repeat	(A)n	chr4	54040906	54040950	+	0	0	0
LINE	L1	L1Md_T	chr4	54683065	54689756	-	0	0	0
Simple_repeat	Simple_repeat	(A)n	chr4	54940717	54940745	+	0	0	0
Simple_repeat	Simple_repeat	(A)n	chr4	54940837	54940867	+	0	0	0
SINE	Alu	B1_Mus2	chr4	55249561	55249707	+	0	0	0
Simple_repeat	Simple_repeat	(A)n	chr4	55249708	55249747	+	0	0	0
SINE	Alu	B1_Mm	chr4	55320534	55320678	+	0	0	0
Simple_repeat	Simple_repeat	(A)n	chr4	55355937	55355977	+	0	0	0
LTR	MaLR	MTB_Mm	chr4	55422717	55423113	+	0	0	0
SINE	Alu	B1_Mm	chr4	55482170	55482317	+	0	0	0
Simple_repeat	Simple_repeat	(A)n	chr4	55482318	55482348	+	0	0	0
SINE	Alu	B1_Mm	chr4	57843339	57843485	-	0	0	0
LINE	L1	L1Md_T	chr4	58024858	58031068	-	0	0	0
SINE	Alu	B1_Mm	chr4	59144276	59144422	-	0	0	0
Simple_repeat	Simple_repeat	(A)n	chr4	59275417	59275484	+	0	0	0
SINE	Alu	B1_Mm	chr4	59584603	59584746	-	0	0	0
Simple_repeat	Simple_repeat	(A)n	chr4	61946739	61946775	+	0	0	0
SINE	Alu	B1_Mus2	chr4	62183082	62183219	+	0	0	0
Simple_repeat	Simple_repeat	(A)n	chr4	62183220	62183243	+	0	0	0
SINE	B2	B2_Mm1t	chr11	37686231	37686420	-	0	0	0.615324
Simple_repeat	Simple_repeat	(A)n	chr11	37707494	37707530	+	0	0	0
Simple_repeat	Simple_repeat	(A)n	chr4	63483229	63483282	+	0	0	0.342623
LTR	MaLR	MTA_Mm	chr4	64632789	64633184	-	0	0	0
SINE	Alu	B1_Mm	chr11	37913545	37913690	+	0	0	0
LINE	L1	L1Md_T	chr4	66292354	66299296	-	0	0	0
SINE	Alu	B1_Mus2	chr4	66511754	66511923	+	0	0	0
Simple_repeat	Simple_repeat	(A)n	chr4	66534598	66534642	+	0	0	0
SINE	Alu	B1_Mus1	chr4	66796993	66797134	+	0	0	0
Simple_repeat	Simple_repeat	(A)n	chr4	66797135	66797179	+	0	0	0
Simple_repeat	Simple_repeat	(A)n	chr4	67086673	67086700	+	0	0	0
SINE	Alu	B1_Mm	chr4	67236896	67237041	+	0	0	0
Simple_repeat	Simple_repeat	(A)n	chr4	67624523	67624549	+	0	0	0
Simple_repeat	Simple_repeat	(A)n	chr4	67768927	67768969	+	0	0	0
SINE	Alu	B1_Mm	chr4	67798823	67798969	+	0	0	0
Simple_repeat	Simple_repeat	(A)n	chr4	67798970	67799010	+	0	0	0
Simple_repeat	Simple_repeat	(A)n	chr4	68173074	68173115	+	0.332194	0	0
SINE	Alu	B1_Mus1	chr4	68274007	68274153	-	0	0	0
SINE	Alu	B1_Mus2	chr4	68348928	68349073	+	0	0.380568	0
LTR	MaLR	MTA_Mm	chr4	68934271	68934665	-	0	0	0.615324
LTR	MaLR	MTA_Mm	chr4	69104219	69104613	-	0	0	0
Simple_repeat	Simple_repeat	(A)n	chr4	69489105	69489135	+	0.332194	0	0
LINE	L1	L1Md_F2	chr4	70385447	70390478	+	0	0	0
SINE	Alu	B1_Mm	chr4	70630602	70630747	+	0	0	0
Simple_repeat	Simple_repeat	(A)n	chr4	70630748	70630777	+	0	0	0
LTR	MaLR	MTA_Mm	chr4	70648761	70649156	+	0	0	0
Simple_repeat	Simple_repeat	(A)n	chr4	72162917	72162965	+	0	0	0
Simple_repeat	Simple_repeat	(A)n	chr4	72249869	72249916	+	0	0	0
LTR	ERVK	RLTR13D6	chr4	72380999	72381762	-	0	0	0

SINE	Alu	B1_Mus2	chr4	73068672	73068815	-	0	0	0
Simple_repeat	Simple_repeat	(A)n	chr4	73115255	73115293	+	0	0	0
LTR	MaLR	MTA_Mm	chr4	73127559	73127953	-	0	0	0
SINE	Alu	B1_Mus1	chr4	73855309	73855455	+	0.591053	0.708814	0.342623
Simple_repeat	Simple_repeat	(A)n	chr4	74161359	74161402	+	0	0	0
Simple_repeat	Simple_repeat	(A)n	chr4	74750548	74750578	+	0	0	0.342623
LTR	MaLR	MTA_Mm	chr4	74963922	74964318	-	0	0	0
Simple_repeat	Simple_repeat	(A)n	chr4	74968815	74968862	+	0	0	0
SINE	Alu	B1_Mus1	chr4	75070524	75070670	+	0	0	0
Simple_repeat	Simple_repeat	(A)n	chr4	75070671	75070700	+	0	0	0
Simple_repeat	Simple_repeat	(A)n	chr11	38943030	38943060	+	0	0	0
Simple_repeat	Simple_repeat	(A)n	chr11	38949656	38949682	+	0	0	0
SINE	Alu	B1_Mm	chr11	39035338	39035485	-	0	0	0
LINE	L1	L1Md_F3	chr4	78081594	78087717	-	0	0	0
LINE	L1	Lx3A	chr4	78236009	78242084	-	0	0	0
LTR	MaLR	MTA_Mm	chr4	78330325	78330721	+	0	0	0
LTR	MaLR	MTA_Mm	chr11	39208683	39209077	-	0	0	0.615324
LTR	MaLR	MTA_Mm	chr4	78494880	78495276	+	0	0	0
SINE	Alu	B1_Mus1	chr4	78528151	78528298	-	0	0	0
Simple_repeat	Simple_repeat	(A)n	chr4	78617374	78617399	+	0	0	0
LINE	L1	L1Md_F2	chr4	78621922	78626952	-	0	0	0
LINE	L1	L1Md_F2	chr4	79161176	79166486	-	0	0	0
SINE	Alu	B1_Mus1	chr4	79283352	79283479	-	0	0	0
LINE	L1	L1_Mus3	chr4	79307124	79312528	-	0	0	0
SINE	Alu	B1_Mus2	chr11	39380534	39380679	-	0.332194	0	0
Simple_repeat	Simple_repeat	(A)n	chr4	80191916	80191954	+	0	0	0.342623
Simple_repeat	Simple_repeat	(A)n	chr4	81332754	81332801	+	0	0	0
Simple_repeat	Simple_repeat	(A)n	chr4	81374077	81374112	+	0	0	0
SINE	Alu	B1_Mus1	chr11	39508462	39508605	+	0	0	0
Simple_repeat	Simple_repeat	(A)n	chr11	39508606	39508642	+	0	0	0
LINE	L1	L1Md_T	chr4	81833098	81839570	-	0	0	0
Simple_repeat	Simple_repeat	(A)n	chr4	82333728	82333758	+	0	0	0
Simple_repeat	Simple_repeat	(A)n	chr4	83590327	83590356	+	0	0	0
SINE	Alu	B1_Mm	chr4	84476717	84476862	-	0	0	0
LINE	L1	L1Md_T	chr4	85053278	85059536	-	0.332194	0	0
SINE	Alu	B1_Mm	chr4	85141714	85141860	+	0	0.708814	0
Simple_repeat	Simple_repeat	(A)n	chr4	85141861	85141887	+	0	0	0
Simple_repeat	Simple_repeat	(A)n	chr4	85250840	85250918	+	0.591053	0.380568	0
Simple_repeat	Simple_repeat	(A)n	chr4	86372602	86372627	+	0	0	0
SINE	Alu	B1_Mm	chr4	86498394	86498532	-	1.58764	0.380568	1.84597
SINE	B2	B2_Mm2	chr4	86509973	86510151	+	0	0.708814	0
SINE	Alu	B1_Mus2	chr11	40105350	40105497	-	0	0	0
Simple_repeat	Simple_repeat	(A)n	chr4	87107878	87107914	+	0	0	0
Simple_repeat	Simple_repeat	(A)n	chr4	87260185	87260232	+	0	0	0
Simple_repeat	Simple_repeat	(A)n	chr4	87330405	87330427	+	0	0	0
LTR	MaLR	MTA_Mm	chr4	88656837	88657232	+	0	0	0
LINE	L1	L1Md_T	chr4	89381050	89386227	+	0	0	0
LTR	MaLR	MTA_Mm	chr4	90034151	90034546	+	0	0	0
LTR	MaLR	MTD	chr4	90047842	90048269	+	0	0	0
SINE	Alu	B1_Mm	chr4	90135846	90135992	+	0	0	0
LINE	L1	L1Md_T	chr4	90220445	90226938	-	0	0	0
SINE	Alu	B1_Mm	chr4	90683429	90683575	-	0	0	0
Simple_repeat	Simple_repeat	(A)n	chr11	40453841	40453884	+	0	0	0
LTR	MaLR	MTA_Mm	chr11	40456214	40456608	+	0	0	0
Simple_repeat	Simple_repeat	(A)n	chr4	91067056	91067095	+	0.332194	0	0
LINE	L1	L1_Mus3	chr4	92302151	92308336	-	0	0	0
LTR	MaLR	MTA_Mm	chr4	92417264	92417658	+	0	0	0
LTR	MaLR	MTA_Mm	chr4	92418758	92419152	+	0	0	0
Simple_repeat	Simple_repeat	(A)n	chr4	92858997	92859021	+	0	0	0
SINE	Alu	B1_Mus2	chr4	93238234	93238380	-	0	0	0
SINE	Alu	B1F2	chr11	40788706	40788827	-	0	0	0
SINE	Alu	B1_Mm	chr4	96367563	96367710	+	0	0	0
Simple_repeat	Simple_repeat	(A)n	chr4	96367711	96367760	+	0	0	0
SINE	Alu	B1_Mus2	chr4	97064985	97065133	+	0	0	0
Simple_repeat	Simple_repeat	(A)n	chr4	98515058	98515077	+	0	0	0
SINE	Alu	B1_Mus2	chr4	98519091	98519237	+	0	0	0
Simple_repeat	Simple_repeat	(A)n	chr4	98519238	98519283	+	0	0	0.342623

SINE	Alu	B1_Mm	chr4	98519284	98519428	+	0	0	0.342623
Simple_repeat	Simple_repeat	(A)n	chr4	98519429	98519457	+	0	0	0.342623
LTR	MaLR	MTB	chr4	98547444	98547837	-	0	0	0
Simple_repeat	Simple_repeat	(A)n	chr4	98590274	98590310	+	0	0	0
Simple_repeat	Simple_repeat	(A)n	chr4	99106879	99106904	+	0	0	0
LTR	MaLR	MTA_Mm	chr4	99157705	99158100	+	0.332194	0	0
Simple_repeat	Simple_repeat	(TTTA)n	chr4	99314056	99314094	+	0	0	0
SINE	Alu	B1_Mm	chr11	41477511	41477634	+	0	0	0
Simple_repeat	Simple_repeat	(A)n	chr4	10097471	10097474	+	0.332194	0	0
SINE	Alu	B1_Mus1	chr11	41564180	41564327	+	0	0	0
Simple_repeat	Simple_repeat	(A)n	chr11	41564328	41564354	+	0	0	0
SINE	Alu	B1_Mus1	chr4	10155174	10155189	+	0	0	0
SINE	Alu	B1_Mm	chr4	10160411	10160425	+	0	0	0
Simple_repeat	Simple_repeat	(A)n	chr4	10160425	10160429	+	0	0	0
SINE	Alu	B1_Mm	chr4	10163097	10163112	+	0	0	0
Simple_repeat	Simple_repeat	(A)n	chr4	10163112	10163117	+	0	0	0
Simple_repeat	Simple_repeat	(A)n	chr4	10190176	10190178	+	1.18211	0.708814	3.05526
Simple_repeat	Simple_repeat	(A)n	chr4	10236818	10236821	+	0	0	0
Simple_repeat	Simple_repeat	(A)n	chr4	10318152	10318162	+	0	0	0
Simple_repeat	Simple_repeat	(A)n	chr4	10338550	10338553	+	3.28746	4.63345	1.82461
SINE	Alu	B1_Mus1	chr4	10347268	10347283	-	0	0	0
Simple_repeat	Simple_repeat	(A)n	chr4	10376897	10376901	+	0	0	0
SINE	Alu	B1_Mus2	chr4	10494199	10494213	+	0	0	0
Simple_repeat	Simple_repeat	(A)n	chr4	10494214	10494217	+	0	0	0.342623
LINE	L1	L1Md_T	chr4	10496511	10497198	-	0	0	0
Simple_repeat	Simple_repeat	(A)n	chr4	10512663	10512668	+	0	0	0
LTR	MaLR	MTA_Mm	chr11	42032811	42033210	-	0	0	0
Simple_repeat	Simple_repeat	(A)n	chr4	10581240	10581246	+	0	0	0
SINE	Alu	B1_Mus1	chr4	10640109	10640124	-	0	0	0
SINE	Alu	B1_Mus1	chr4	10650798	10650813	+	0	0	0
Simple_repeat	Simple_repeat	(A)n	chr4	10650813	10650816	+	0	0	0
SINE	B2	B2_Mm1a	chr4	10689370	10689389	+	0	0	0
Simple_repeat	Simple_repeat	(A)n	chr11	42218746	42218773	+	0	0	0
SINE	Alu	B1_Mus2	chr4	10760834	10760849	-	0	0	0
LTR	MaLR	MTA_Mm	chr4	10809347	10809386	-	0	0	0
LTR	MaLR	MTA_Mm	chr4	10809494	10809534	-	0	0	0
SINE	Alu	B1_Mus1	chr4	10848155	10848169	-	0	0	0
SINE	Alu	B1_Mur2	chr4	10887559	10887573	-	0	0	0
SINE	Alu	B1_Mus2	chr4	10928865	10928880	+	0	0	0
Simple_repeat	Simple_repeat	(GGAA)n	chr4	10928882	10928889	+	0	0	0
SINE	Alu	B1_Mm	chr4	10932102	10932116	+	0	0	0
SINE	Alu	B1_Mm	chr4	10934438	10934453	-	0	0	0
SINE	Alu	B1_Mm	chr4	10963966	10963980	-	0	0	0
SINE	Alu	B1_Mus1	chr11	42710611	42710766	-	0	0	0
SINE	Alu	B1_Mus2	chr4	11138382	11138396	+	0.332194	0	0
Simple_repeat	Simple_repeat	(A)n	chr4	11138396	11138399	+	0.332194	0	0
LTR	MaLR	MTA_Mm	chr11	42881138	42881530	+	0	0	0
SINE	Alu	B1_Mm	chr4	11199244	11199258	-	0	0	0
Simple_repeat	Simple_repeat	(A)n	chr11	42941225	42941291	+	0.332194	0	0
Simple_repeat	Simple_repeat	(A)n	chr4	11296611	11296616	+	0	0	0
SINE	Alu	B1_Mm	chr4	11310856	11310871	-	0	0	0
SINE	Alu	B1_Mus2	chr4	11459379	11459393	+	0	0	0
Simple_repeat	Simple_repeat	(GAAA)n	chr4	11459394	11459400	+	0	0	0.342623
SINE	Alu	B1_Mus2	chr4	11467069	11467083	-	0	0	0
Simple_repeat	Simple_repeat	(A)n	chr4	11471636	11471639	+	0	0	0
SINE	Alu	B1_Mus1	chr4	11572746	11572761	-	0	0	0
SINE	Alu	B1_Mus2	chr4	11585993	11586007	-	0	0	0
Simple_repeat	Simple_repeat	(A)n	chr11	43247141	43247176	+	0	0	0.342623
LTR	MaLR	MTB	chr4	11622249	11622288	-	0	0	0
SINE	Alu	B1_Mus1	chr4	11635481	11635496	+	0	0	0
Simple_repeat	Simple_repeat	(A)n	chr4	11635496	11635502	+	0	0	0
SINE	Alu	B1_Mus2	chr4	11651061	11651075	+	0	0	0
Simple_repeat	Simple_repeat	(A)n	chr4	11651075	11651078	+	0	0	0
SINE	Alu	B1_Mur4	chr4	11668220	11668239	-	0	0	0
SINE	Alu	B1_Mm	chr4	11681757	11681772	+	0	0	0
Simple_repeat	Simple_repeat	(A)n	chr4	11681772	11681774	+	0	0	0.342623
SINE	Alu	B1_Mus1	chr4	11747182	11747194	-	0	0	0

SINE	B2	B2_Mm1a	chr4	11797335	11797353	-	0	3.53776	0.342623
SINE	Alu	B1_Mus2	chr4	11811229	11811243	-	0	0	0
SINE	Alu	B1_Mus2	chr4	11820609	11820623	-	0	0	0
Simple_repeat	Simple_repeat	(A)n	chr4	11842816	11842819	+	0	0	0
SINE	Alu	B1_Mur4	chr4	11854638	11854653	+	0	0	0
SINE	Alu	B1_Mus2	chr4	11858212	11858226	+	0	0	0
LINE	L1	L1VL4	chr4	11861820	11862301	+	0	0	0
SINE	Alu	B1_Mus2	chr4	11865739	11865753	-	0	0	0
SINE	B2	B2_Mm1a	chr4	11866664	11866683	-	0	0	0
Simple_repeat	Simple_repeat	(A)n	chr4	11872163	11872167	+	0	0	0
SINE	Alu	B1_Mus2	chr4	11917548	11917563	-	0	0	0
SINE	Alu	B1_Mm	chr4	11931234	11931246	-	0	0	0
Simple_repeat	Simple_repeat	(A)n	chr4	11942627	11942632	+	1.84649	1.08938	2.18859
Simple_repeat	Simple_repeat	(GAAA)n	chr4	11986925	11986938	+	0	0	0
Simple_repeat	Simple_repeat	(A)n	chr4	12003052	12003056	+	0	0	0
SINE	B2	B3	chr11	43652429	43652623	-	0	0	0
SINE	Alu	B1_Mus2	chr4	12102621	12102635	+	0	0	0
Simple_repeat	Simple_repeat	(A)n	chr4	12118806	12118811	+	0	0	0
SINE	Alu	B1_Mus1	chr4	12144484	12144498	+	0	0	0
Simple_repeat	Simple_repeat	(A)n	chr4	12144498	12144501	+	0	0	0
SINE	Alu	B1_Mus1	chr4	12150437	12150452	+	0	0	0
SINE	Alu	B1_Mus1	chr4	12189735	12189750	+	0	0	0
SINE	Alu	B1_Mus1	chr4	12215899	12215914	+	0	0	0
SINE	Alu	B1_Mur3	chr4	12249746	12249761	-	0	0	0
Simple_repeat	Simple_repeat	(A)n	chr4	12253641	12253644	+	1.18211	0.761137	1.84597
Low_complexity	Low_complexity	GA-rich	chr4	12266564	12266569	+	0.664388	0	0.342623
Simple_repeat	Simple_repeat	(A)n	chr4	12276018	12276020	+	0	0	0
SINE	Alu	B1_Mus2	chr4	12295562	12295576	+	0	0	0
SINE	Alu	B1_Mus1	chr4	12353292	12353306	+	0	0	0
Low_complexity	Low_complexity	A-rich	chr4	12353306	12353310	+	0	0	0
SINE	Alu	B1_Mus1	chr4	12380741	12380755	-	0	0	0
LTR	MaLR	MTB	chr4	12401446	12401485	-	0	0	0.342623
LTR	MaLR	MTA_Mm	chr4	12417267	12417307	+	0	0	0
SINE	Alu	B1_Mm	chr4	12425627	12425642	-	0	0	0.615324
SINE	Alu	B1_Mm	chr4	12441739	12441753	-	0	0	0
SINE	Alu	B1_Mm	chr4	12441788	12441803	-	0	0	0
SINE	Alu	B1_Mus2	chr4	12454139	12454153	+	0	0	0
Simple_repeat	Simple_repeat	(GAA)n	chr4	12454154	12454160	+	0	0.380568	0.342623
SINE	Alu	B1_Mus2	chr4	12476037	12476052	+	0	0	0
Simple_repeat	Simple_repeat	(A)n	chr4	12476052	12476054	+	0	0	0
SINE	Alu	B1_Mus2	chr4	12562846	12562861	+	0	0	0.615324
Simple_repeat	Simple_repeat	(A)n	chr4	12562861	12562863	+	0	0	0.615324
SINE	Alu	B1_Mm	chr4	12567219	12567233	+	0	0	0
LTR	MaLR	MTC	chr4	12567234	12567271	+	0	0	0
SINE	Alu	B1_Mus2	chr4	12665441	12665456	+	0	0	0
Simple_repeat	Simple_repeat	(A)n	chr4	12665456	12665458	+	0	0	0.342623
SINE	Alu	B1_Mm	chr4	12675283	12675298	+	0	0	0
Simple_repeat	Simple_repeat	(A)n	chr4	12675298	12675301	+	0	0	0
Simple_repeat	Simple_repeat	(A)n	chr11	44431278	44431356	+	11.4927	4.9617	3.02097
SINE	Alu	B1_Mm	chr4	12714791	12714806	-	0	0	0
Simple_repeat	Simple_repeat	(A)n	chr4	12732805	12732807	+	0	0	0
LTR	MaLR	MTA_Mm	chr4	12758660	12758699	-	0	0	0
LTR	MaLR	MTA_Mm	chr4	12860569	12860608	-	0	0	0
Simple_repeat	Simple_repeat	(A)n	chr4	12865038	12865042	+	0	0	0
SINE	Alu	B1_Mus2	chr4	12866295	12866310	+	0	0	0
Simple_repeat	Simple_repeat	(A)n	chr4	12866310	12866312	+	0	0	0
Simple_repeat	Simple_repeat	(A)n	chr4	12894921	12894923	+	0	0	0
SINE	Alu	B1_Mus2	chr4	12918458	12918472	+	0.591053	0	0.615324
SINE	Alu	B1_Mus2	chr4	12922345	12922357	+	0	0	0
Simple_repeat	Simple_repeat	(A)n	chr4	12922357	12922359	+	0	0	0
SINE	Alu	B1_Mm	chr4	12922454	12922469	-	0	0	0
SINE	B2	B2_Mm1t	chr4	12947950	12947969	-	0	0	0
SINE	Alu	B1_Mus2	chr4	12961790	12961805	+	0	0	0
Simple_repeat	Simple_repeat	(A)n	chr4	12961805	12961808	+	0	0	0
Simple_repeat	Simple_repeat	(TTTA)n	chr4	12998041	12998043	+	0	0	0
SINE	Alu	B1_Mus1	chr4	13019337	13019350	+	0	0	0.342623
Simple_repeat	Simple_repeat	(A)n	chr4	13019350	13019353	+	0	0	0.342623

SINE	Alu	B1_Mm	chr4	13019794	13019807	-	0	0	0
Simple_repeat	Simple_repeat	(A)n	chr4	13021836	13021839	+	0	0	0
SINE	Alu	B1_Mus1	chr4	13185256	13185271	-	0	0	0.342623
SINE	B2	B2_Mm1a	chr4	13186228	13186247	-	0	0	0
SINE	Alu	B1_Mm	chr4	13190312	13190327	+	0	0	0
Simple_repeat	Simple_repeat	(A)n	chr4	13190327	13190333	+	0	0	0
SINE	Alu	B1_Mus1	chr4	13203830	13203844	-	0	0	0
SINE	Alu	B1_Mus2	chr4	13214013	13214027	+	0	0	0
Simple_repeat	Simple_repeat	(A)n	chr4	13214027	13214030	+	0	0	0
Simple_repeat	Simple_repeat	(A)n	chr4	13235980	13235983	+	0	0	0
SINE	Alu	B1_Mus1	chr4	13278284	13278299	+	0	0	0
Simple_repeat	Simple_repeat	(A)n	chr4	13278299	13278301	+	0	0	0
SINE	Alu	B1_Mus2	chr4	13282016	13282030	-	0	0	0
SINE	Alu	B1_Mus2	chr4	13283652	13283667	+	0	0	0
LTR	MaLR	MTA_Mm	chr4	13310631	13310671	+	0.332194	0	0
Simple_repeat	Simple_repeat	(A)n	chr4	13315238	13315240	+	0	0	0
SINE	Alu	B1_Mus2	chr4	13334713	13334728	+	0	0	0
SINE	Alu	B1_Mus2	chr4	13347823	13347838	-	0	0	0
Simple_repeat	Simple_repeat	(A)n	chr4	13449506	13449509	+	0.591053	0	0
SINE	Alu	B1_Mus1	chr4	13475466	13475480	+	0	0	0
Simple_repeat	Simple_repeat	(A)n	chr11	45778527	45778599	+	0	0	0
Simple_repeat	Simple_repeat	(A)n	chr10	20892610	20892653	+	0	0	0
SINE	Alu	B1_Mm	chr4	13518764	13518777	+	0	0	0
Simple_repeat	Simple_repeat	(A)n	chr4	13518777	13518779	+	0	0	0
SINE	Alu	B1_Mus2	chr4	13522419	13522433	-	0	0	0
SINE	Alu	B1_Mus2	chr4	13543471	13543486	+	0	0	0
Simple_repeat	Simple_repeat	(A)n	chr4	13543486	13543488	+	0	0	0
SINE	Alu	B1_Mus2	chr4	13566235	13566250	-	0	0	0
Simple_repeat	Simple_repeat	(A)n	chr4	13568690	13568693	+	0	0	0
Simple_repeat	Simple_repeat	(A)n	chr4	13575277	13575279	+	0	0	0
SINE	Alu	B1_Mm	chr4	13583391	13583406	-	0	0	0
Simple_repeat	Simple_repeat	(TTTA)n	chr4	13616149	13616153	+	0	0	0
SINE	Alu	B1_Mus2	chr4	13644442	13644457	-	0	0	0
Simple_repeat	Simple_repeat	(A)n	chr4	13645941	13645944	+	0	0	0
Simple_repeat	Simple_repeat	(A)n	chr4	13681386	13681389	+	0	0	0
SINE	Alu	B1_Mm	chr4	13714529	13714544	-	0	0	0
SINE	Alu	B1_Mus2	chr4	13810602	13810617	+	0	0	0
Simple_repeat	Simple_repeat	(A)n	chr4	13810617	13810620	+	0	0	0
SINE	Alu	B1_Mm	chr11	46204625	46204770	-	0	0	0
Simple_repeat	Simple_repeat	(A)n	chr4	13869777	13869783	+	0	0	0
SINE	Alu	B1_Mus2	chr4	14023774	14023789	-	0	0	0
Simple_repeat	Simple_repeat	(A)n	chr4	14045550	14045553	+	0	0	0
Low_complexity	Low_complexity	AT_rich	chr4	14070153	14070156	+	0	0	0
SINE	Alu	B1_Mus2	chr4	14111045	14111059	-	0	0	0
SINE	Alu	B1_Mm	chr4	14112007	14112022	+	0	0	0
Simple_repeat	Simple_repeat	(A)n	chr4	14122455	14122458	+	0	0	0
SINE	B2	B2_Mm1a	chr4	14128252	14128270	+	0	0	0
Low_complexity	Low_complexity	GA-rich	chr11	46608545	46608701	+	0	0	0
Simple_repeat	Simple_repeat	(A)n	chr4	14235316	14235318	+	0	0	0
SINE	Alu	B1_Mus2	chr11	46675490	46675636	-	0	0	0
SINE	Alu	B1_Mus1	chr4	14289478	14289492	+	0	0	0
Low_complexity	Low_complexity	A-rich	chr4	14289492	14289500	+	0	0	0
SINE	Alu	B1_Mm	chr4	14296447	14296459	-	0	0	0
SINE	B2	B3A	chr4	14297047	14297066	+	0	0	0
Simple_repeat	Simple_repeat	(A)n	chr4	14297066	14297069	+	0	0	0
SINE	Alu	B1_Mus1	chr4	14298369	14298383	-	0	0	0
SINE	Alu	B1_Mus2	chr11	46714729	46714873	-	0	0	0
LTR	MaLR	MTA_Mm	chr4	14308738	14308778	+	0	0	0
SINE	Alu	B1_Mus2	chr4	14351704	14351718	+	0	0	0
Simple_repeat	Simple_repeat	(A)n	chr4	14351718	14351720	+	0	0	0
SINE	Alu	B1_Mus1	chr4	14363840	14363855	-	0	0	0
SINE	Alu	B1_Mm	chr4	14480038	14480053	-	0	0	0
SINE	Alu	B1_Mm	chr4	14491621	14491635	-	0	0	0
SINE	Alu	B1_Mus2	chr4	14499704	14499718	+	0	0	0
Simple_repeat	Simple_repeat	(A)n	chr4	14499718	14499721	+	0	0	0
SINE	B2	B2_Mm1t	chr4	14507140	14507159	-	0	0	0
SINE	Alu	B1_Mus2	chr4	14515358	14515373	-	0	0	0.615324

LTR	MaLR	MTA_Mm	chr4	14519889	14519928	-	0	0	0
SINE	Alu	B1_Mm	chr4	14557942	14557956	+	0	0	0
Simple_repeat	Simple_repeat	(A)n	chr4	14557956	14557959	+	0	0	0
SINE	Alu	B1_Mm	chr4	14559931	14559945	-	0	0	0
SINE	Alu	B1_Mus2	chr4	14571808	14571823	-	0	0	0
SINE	Alu	B1_Mm	chr4	14622539	14622554	+	0	0	0
SINE	Alu	B1_Mus2	chr4	14623026	14623041	-	0	0	0
SINE	Alu	B1_Mm	chr4	14696813	14696828	-	0	0	0
SINE	Alu	B1_Mm	chr4	14702899	14702913	-	0.591053	0	0
Simple_repeat	Simple_repeat	(A)n	chr4	14719106	14719108	+	0	0	0
SINE	Alu	B1_Mus2	chr4	14743778	14743792	+	0	0	0
Simple_repeat	Simple_repeat	(A)n	chr4	14743792	14743794	+	0	0	0
SINE	Alu	B1_Mus1	chr4	14744297	14744310	+	0	0	0
Simple_repeat	Simple_repeat	(A)n	chr4	14744310	14744313	+	0	0	0
SINE	Alu	B1_Mus2	chr4	14796433	14796447	-	2.94048	0.708814	1.55647
SINE	Alu	B1_Mus2	chr4	14800258	14800273	-	0	0	0
SINE	Alu	B1_Mus2	chr4	14804683	14804697	-	0	0	0
SINE	Alu	B1_Mus2	chr4	14854172	14854187	-	0	0	0
Simple_repeat	Simple_repeat	(A)n	chr4	14887935	14887939	+	0	0	0.342623
SINE	Alu	B1_Mus1	chr4	14908048	14908058	-	0	0	0
SINE	Alu	B1_Mus1	chr4	14911661	14911674	+	0	0	0
Simple_repeat	Simple_repeat	(A)n	chr4	14911674	14911677	+	0	0.380568	0
SINE	Alu	B1_Mus1	chr4	14933185	14933198	+	0	0	0
Simple_repeat	Simple_repeat	(A)n	chr4	14933198	14933201	+	0	0	0.342623
SINE	Alu	B1_Mus2	chr4	14965126	14965141	+	0	0	0
Low_complexity	Low_complexity	A-rich	chr4	14965141	14965147	+	0	0	0
SINE	Alu	B1_Mus1	chr4	15026595	15026608	+	0	0	0.342623
SINE	B2	B2_Mm1a	chr4	15033196	15033215	-	0	0	0
SINE	Alu	B1_Mus1	chr4	15169781	15169795	-	0	0	0
SINE	Alu	B1_Mm	chr4	15254341	15254356	-	0	0	0
LTR	MaLR	MTB	chr4	15258388	15258427	+	0	0	0
LINE	L1	L1Md_F2	chr11	48012487	48017719	+	0	0	0
Simple_repeat	Simple_repeat	(A)n	chr4	15409431	15409434	+	0	0	0
LTR	ERVL	MLT2D	chr4	15409434	15409464	+	0	0	0
SINE	Alu	B1_Mus2	chr4	15429603	15429618	+	0	0	0
Simple_repeat	Simple_repeat	(A)n	chr4	15429618	15429620	+	0	0	0
SINE	Alu	B1_Mus2	chr4	15514013	15514028	-	0	0	0
LTR	ERVL	RMER15	chr4	15518379	15518412	-	0	0	0
SINE	Alu	B1_Mus1	chr4	15518413	15518428	-	0	0	0
Simple_repeat	Simple_repeat	(TTTA)n	chr4	15521076	15521079	+	0	0.708814	0
SINE	Alu	B1_Mus1	chr4	15544074	15544088	+	0	0	0
Simple_repeat	Simple_repeat	(A)n	chr4	15544088	15544091	+	0	0	0
SINE	Alu	B1_Mm	chr4	15551334	15551349	+	0	0	0
Simple_repeat	Simple_repeat	(A)n	chr4	15551349	15551352	+	0.332194	0	0
SINE	Alu	B1_Mus2	chr4	15558705	15558719	-	0	0	0
Simple_repeat	Simple_repeat	(A)n	chr5	3118537	3118593	+	0	0	0
LINE	L1	L1Md_A	chr5	3175316	3180450	-	0	0	0
SINE	Alu	B1_Mus2	chr5	3538007	3538156	-	0	0	0
LTR	MaLR	MTA_Mm	chr5	3650172	3650566	-	0	0	0.342623
LTR	MaLR	MTA_Mm	chr5	3918469	3918847	-	0	0	0
SINE	Alu	B1_Mus2	chr11	48465741	48465887	+	0	0	1.23065
SINE	Alu	B1_Mus2	chr5	5500825	5500969	-	0.591053	0	0
LTR	MaLR	MTA_Mm	chr5	5903165	5903558	+	0	0	0
LTR	MaLR	MTA_Mm	chr5	5904644	5905037	+	0	0	0
SINE	B4	B4A	chr5	6177374	6177638	-	0.591053	0	0
SINE	Alu	B1_Mus2	chr5	6571122	6571268	-	0.332194	0.380568	0
SINE	Alu	B1_Mus2	chr11	48600270	48600415	+	0	0	0
Simple_repeat	Simple_repeat	(A)n	chr11	48600416	48600442	+	0	0	0
Simple_repeat	Simple_repeat	(A)n	chr11	48612907	48612942	+	0	0	0
LINE	L1	Lx8	chr5	7400952	7405667	-	0	0	0
Simple_repeat	Simple_repeat	(TTTA)n	chr5	7495409	7495444	+	0	0	0
SINE	Alu	B1_Mm	chr5	7549707	7549853	-	0	0	0
Simple_repeat	Simple_repeat	(A)n	chr5	7584265	7584307	+	0	0	0
Simple_repeat	Simple_repeat	(A)n	chr5	7845535	7845614	+	0	0	0
SINE	Alu	B1_Mus1	chr5	8794566	8794686	+	0	0	0
Simple_repeat	Simple_repeat	(A)n	chr5	8794687	8794714	+	0	0	0
Low_complexity	Low_complexity	A-rich	chr5	9242552	9242580	+	0	0	0

SINE	Alu	B1_Mm	chr5	10702482	10702630	+	0	0	0
Simple_repeat	Simple_repeat	(A)n	chr5	10702631	10702666	+	0	0	0
Simple_repeat	Simple_repeat	(A)n	chr5	11177436	11177465	+	0	0	0
SINE	B2	B2_Mm2	chr10	21251148	21251337	-	0	0	0
Simple_repeat	Simple_repeat	(A)n	chr5	11995027	11995059	+	0	0	0
Simple_repeat	Simple_repeat	(A)n	chr5	12358935	12358968	+	0	0	0
LTR	MaLR	MTA_Mm	chr5	12800522	12800913	+	0	0	0
LTR	MaLR	MTA_Mm	chr5	12802009	12802424	+	0	0	0
SINE	Alu	B1_Mus1	chr5	13088007	13088148	+	0	0	0
Simple_repeat	Simple_repeat	(A)n	chr5	13088149	13088187	+	0	0	0
LTR	MaLR	MTA_Mm	chr5	13890440	13890833	-	0	0	0
LTR	MaLR	MTA_Mm	chr5	14443586	14443980	-	0	0	0
Low_complexity	Low_complexity	A-rich	chr5	14915889	14915981	+	0	0	0
Simple_repeat	Simple_repeat	(A)n	chr5	15252668	15252706	+	0	0.380568	0
SINE	Alu	B1_Mus2	chr5	15897110	15897256	+	0	0	0
Simple_repeat	Simple_repeat	(A)n	chr5	15897257	15897324	+	0	0	0
Low_complexity	Low_complexity	A-rich	chr5	15965484	15965549	+	0	0	0
Simple_repeat	Simple_repeat	(A)n	chr5	16186837	16186881	+	0	0	0
LINE	L1	L1Md_T	chr5	16538848	16545477	-	0	0	0
Simple_repeat	Simple_repeat	(A)n	chr5	16670882	16670932	+	0	0	0
LINE	L1	L1Md_T	chr5	16744089	16753072	-	0	0	0
SINE	Alu	B1_Mus1	chr5	16806219	16806369	+	0	0	0
Simple_repeat	Simple_repeat	(A)n	chr5	16806370	16806391	+	0	0	0
SINE	Alu	B1_Mus1	chr5	16975257	16975403	+	0	0	0
LTR	MaLR	MTA_Mm	chr5	18546832	18547226	+	0	0	0
SINE	Alu	B1_Mm	chr5	20483029	20483175	+	0	0	0
Simple_repeat	Simple_repeat	(A)n	chr5	20483176	20483215	+	0	0	0
SINE	B2	B2_Mm2	chr5	20487001	20487189	+	0	0	0
SINE	B2	B2_Mm1a	chr5	20578171	20578359	-	0	0	0
Simple_repeat	Simple_repeat	(A)n	chr5	22081438	22081467	+	0	0	0
SINE	Alu	B1_Mus2	chr5	22120974	22121122	+	0	0	0.615324
Simple_repeat	Simple_repeat	(A)n	chr5	22121123	22121149	+	0	0	0.615324
Simple_repeat	Simple_repeat	(A)n	chr5	22177576	22177613	+	0	0.708814	0
Simple_repeat	Simple_repeat	(A)n	chr5	22237107	22237148	+	0	0	0.342623
Simple_repeat	Simple_repeat	(A)n	chr5	22795153	22795198	+	0	0	0
SINE	Alu	B1_Mus1	chr5	23230505	23230645	-	0	0	0
Simple_repeat	Simple_repeat	(A)n	chr5	23805549	23805587	+	0	0	0
SINE	B2	B2_Mm2	chr5	23815082	23815270	+	0	0	0.342623
SINE	Alu	B1_Mus2	chr5	24174715	24174860	+	0	0	0
Simple_repeat	Simple_repeat	(A)n	chr5	24174861	24174884	+	0	0	0
LTR	MaLR	MTA_Mm	chr5	24665602	24665996	+	0	0	0
SINE	Alu	B1_Mus1	chr11	50063738	50063884	-	0	0	0
SINE	Alu	B1_Mus2	chr5	24774817	24774957	+	0.591053	0	0.615324
Simple_repeat	Simple_repeat	(A)n	chr5	24774958	24774982	+	0.591053	0	0.615324
SINE	Alu	B1_Mm	chr5	25037318	25037463	-	0	0	0
SINE	Alu	B1_Mm	chr5	25167090	25167235	-	0	0	0
SINE	Alu	B1_Mur3	chr11	50157166	50157321	+	0	0	0
Simple_repeat	Simple_repeat	(A)n	chr11	50157322	50157347	+	0	0	0
Simple_repeat	Simple_repeat	(A)n	chr5	27123309	27123354	+	0	0	0
SINE	B2	B2_Mm1t	chr10	21433994	21434186	-	0	0	0
LTR	MaLR	MTA_Mm	chr5	29280719	29281114	-	0	0	0
Simple_repeat	Simple_repeat	(A)n	chr5	29724076	29724120	+	0	0	0
SINE	Alu	PB1D10	chr5	29795441	29795551	-	0	0	0
SINE	Alu	B1_Mm	chr5	29850258	29850404	-	0	0	0
SINE	Alu	B1_Mm	chr5	30149851	30149997	-	0	0	0
SINE	Alu	B1_Mur2	chr5	30192346	30192496	-	0	0	0
Simple_repeat	Simple_repeat	(A)n	chr5	30207683	30207717	+	0	0	0
Low_complexity	Low_complexity	A-rich	chr5	30270786	30270872	+	0	0	0
SINE	Alu	B1_Mus1	chr5	31012576	31012714	+	0.332194	0	0
Simple_repeat	Simple_repeat	(A)n	chr5	31012715	31012755	+	0.332194	0	0
SINE	Alu	B1_Mur1	chr5	31301749	31301890	+	0	0	0
Simple_repeat	Simple_repeat	(A)n	chr5	31301891	31301916	+	0	0	0
SINE	Alu	B1_Mm	chr5	31381858	31382004	+	0	0	0
Simple_repeat	Simple_repeat	(A)n	chr5	31382005	31382036	+	0	0	0
SINE	Alu	B1_Mm	chr5	31403907	31404056	+	0	0	0
Simple_repeat	Simple_repeat	(A)n	chr5	31404057	31404081	+	0	0	0
SINE	Alu	B1_Mus1	chr5	31408801	31408948	+	0	0	0

Simple_repeat	Simple_repeat	(A)n	chr5	31408949	31408990	+	0	0	0
SINE	Alu	B1_Mm	chr5	31535153	31535299	+	0	0	0
Simple_repeat	Simple_repeat	(A)n	chr5	32723704	32723740	+	0	0.380568	0
SINE	Alu	B1_Mus2	chr5	33129085	33129231	+	0	0	0
Simple_repeat	Simple_repeat	(A)n	chr5	33129232	33129263	+	0	0	0
SINE	Alu	B1_Mm	chr5	33201993	33202138	+	0	0	0
SINE	Alu	B1_Mus2	chr5	33658233	33658379	-	0	0	0
SINE	B2	B3A	chr5	33676978	33677173	+	0	0	0
Simple_repeat	Simple_repeat	(A)n	chr5	33677174	33677195	+	0	0.380568	0
SINE	Alu	B1_Mm	chr5	34060226	34060372	+	0	0	0.615324
Simple_repeat	Simple_repeat	(A)n	chr5	34060373	34060401	+	0	0.761137	0.615324
Simple_repeat	Simple_repeat	(A)n	chr5	34319091	34319128	+	0	0	0
SINE	B4	ID_B1	chr5	35431991	35432214	-	0	0	0
Simple_repeat	Simple_repeat	(A)n	chr5	35641675	35641715	+	0	0	0
LTR	MaLR	MTA_Mm	chr5	36357696	36358089	+	0	0	0
SINE	Alu	B1_Mm	chr5	37155726	37155872	+	0	0	0
Simple_repeat	Simple_repeat	(A)n	chr5	37155873	37155915	+	0	0	0
SINE	Alu	B1_Mm	chr5	37437229	37437375	-	0	0	0
Simple_repeat	Simple_repeat	(A)n	chr5	38108750	38108788	+	0	0	0
SINE	Alu	B1_Mm	chr5	38301451	38301599	+	0	0	0
Simple_repeat	Simple_repeat	(A)n	chr5	38301600	38301631	+	0	0	0
Simple_repeat	Simple_repeat	(A)n	chr5	39024086	39024113	+	0	0	0
LTR	MaLR	MTA_Mm	chr5	39826534	39826929	+	0	0	0
Simple_repeat	Simple_repeat	(A)n	chr5	40743604	40743632	+	0	0	0
LTR	MaLR	MTA_Mm	chr5	40973251	40973649	-	0	0	0
LTR	MaLR	MTA_Mm	chr5	40974740	40975138	-	0	0	0
LTR	MaLR	MTA_Mm	chr5	41330001	41330395	+	0	0	0
Simple_repeat	Simple_repeat	(A)n	chr5	41336037	41336071	+	0	0	0
LTR	MaLR	MTB	chr5	41403951	41404344	-	0	0	0
SINE	Alu	B1_Mm	chr5	42297793	42297939	+	0	0	0
Simple_repeat	Simple_repeat	(A)n	chr5	42297940	42297980	+	0	0	0.342623
LINE	L1	L1Md_T	chr5	42391767	42397052	-	0	0	0
LTR	MaLR	MTB	chr5	43182415	43182787	-	0	0	0
LTR	ERVK	RMER19B	chr5	44049531	44050478	+	0	0	0
SINE	Alu	B1_Mm	chr11	51477816	51477938	-	0	0	0
SINE	Alu	B1_Mus1	chr5	44305968	44306114	-	0	0	0
Simple_repeat	Simple_repeat	(A)n	chr5	44560452	44560490	+	0	0	0
LTR	MaLR	MTA_Mm	chr5	45499344	45499738	-	0	0	0
SINE	B2	B2_Mm1t	chr5	45719065	45719255	-	0	0	0
Simple_repeat	Simple_repeat	(A)n	chr5	45728257	45728310	+	0	0	0
SINE	Alu	B1_Mm	chr5	46296351	46296496	+	0	0	0
Simple_repeat	Simple_repeat	(A)n	chr5	46296497	46296573	+	0	0	0
scRNA	scRNA	4.5SRNA	chr5	46498263	46498356	-	0	0	0
Simple_repeat	Simple_repeat	(A)n	chr5	46726531	46726560	+	0	0	0
SINE	Alu	B1_Mm	chr5	47050350	47050495	+	0	0	1.10507
Simple_repeat	Simple_repeat	(A)n	chr5	47050496	47050521	+	0	0	1.10507
SINE	Alu	B1_Mm	chr11	51614093	51614238	+	0	0	0
Simple_repeat	Simple_repeat	(A)n	chr11	51614239	51614264	+	0	0	0
Simple_repeat	Simple_repeat	(A)n	chr5	48212764	48212792	+	0	0	0
Simple_repeat	Simple_repeat	(A)n	chr5	49726187	49726226	+	0	0	0
Simple_repeat	Simple_repeat	(A)n	chr5	50065257	50065304	+	0	0	0
LINE	L1	L1Md_F2	chr5	50466858	50471342	+	0	0.708814	0
Simple_repeat	Simple_repeat	(A)n	chr5	50548444	50548473	+	0	0	0
Simple_repeat	Simple_repeat	(A)n	chr5	50571143	50571185	+	0	0	0
SINE	Alu	B1_Mus1	chr5	50583500	50583646	+	0	0	0
Simple_repeat	Simple_repeat	(A)n	chr5	50583647	50583673	+	0	0	0
Simple_repeat	Simple_repeat	(A)n	chr5	51064755	51064791	+	0	0	0
LTR	MaLR	MTA_Mm	chr5	52041748	52042143	-	1.18211	0	0.957947
Simple_repeat	Simple_repeat	(A)n	chr5	52263344	52263380	+	0	0	0
LTR	MaLR	MTA_Mm	chr5	52333307	52333703	+	0	0	0
SINE	Alu	B1_Mus1	chr5	53109935	53110070	-	0	0	0
SINE	Alu	B1_Mus1	chr5	53473877	53474024	-	0	0	0
SINE	Alu	B1_Mm	chr5	53879646	53879790	+	0	0	0
Simple_repeat	Simple_repeat	(A)n	chr5	53879791	53879814	+	0	0	0
SINE	Alu	B1_Mus1	chr5	54071430	54071567	-	0	0	0
Simple_repeat	Simple_repeat	(A)n	chr5	55487170	55487224	+	0	0	0
SINE	Alu	B1_Mus2	chr5	55647202	55647344	+	0	0	0

SINE	Alu	B1_Mus1	chr5	55907400	55907547	+	0	0	0
SINE	Alu	B1_Mus2	chr5	56098912	56099057	+	0	0	0
Simple_repeat	Simple_repeat	(A)n	chr5	56146741	56146790	+	0	0	0
Simple_repeat	Simple_repeat	(A)n	chr5	56200637	56200666	+	0	0	0
LINE	L1	L1Md_T	chr5	56228525	56235445	-	0	0	0
SINE	Alu	B1_Mm	chr5	56252636	56252750	-	0	0	0
SINE	Alu	B1_Mus2	chr5	56256680	56256825	+	0	0	0
Simple_repeat	Simple_repeat	(A)n	chr5	56256826	56256847	+	0	0	0
Simple_repeat	Simple_repeat	(A)n	chr5	56622432	56622468	+	0	0	0
SINE	Alu	B1_Mus2	chr5	56749775	56749901	+	0	0	0
Simple_repeat	Simple_repeat	(A)n	chr5	56749902	56749930	+	0	0	0
SINE	Alu	B1_Mur4	chr5	56879996	56880145	-	0	0	0
SINE	Alu	B1_Mm	chr5	57419783	57419934	+	0	0	0
Simple_repeat	Simple_repeat	(A)n	chr5	57419935	57419969	+	0	0	0
LINE	L1	L1Md_F2	chr5	57636519	57641682	+	0	0	0
Simple_repeat	Simple_repeat	(A)n	chr11	52247261	52247300	+	0	0	0
SINE	Alu	B1_Mus1	chr11	52263217	52263353	-	0	0	0
SINE	Alu	B1_Mm	chr5	59075751	59075897	-	0	0	0
LINE	L1	L1_Mus2	chr5	59478292	59482906	-	0	0	0
Simple_repeat	Simple_repeat	(A)n	chr5	59815412	59815453	+	0	0	0
SINE	Alu	B1_Mus2	chr5	60222719	60222866	+	0	0	0
Simple_repeat	Simple_repeat	(A)n	chr5	60222867	60222891	+	0	0	0
Simple_repeat	Simple_repeat	(A)n	chr5	60339531	60339580	+	0	0	0
Low_complexity	Low_complexity	A-rich	chr5	60799577	60799644	+	0	0	0
LINE	L1	L1Md_T	chr5	60941757	60948495	-	0	0	0
LTR	MaLR	MTA_Mm	chr5	61032907	61033302	+	0	0	0
LTR	MaLR	MTA_Mm	chr5	61073357	61073752	+	0	0	0
SINE	Alu	B1_Mm	chr5	61154611	61154737	-	0	0	0
SINE	Alu	B1_Mus1	chr5	62370353	62370497	-	0	0	0
Simple_repeat	Simple_repeat	(A)n	chr5	62641838	62641878	+	0	0	0
Simple_repeat	Simple_repeat	(A)n	chr5	62661189	62661232	+	0	0	0
LTR	MaLR	MTA_Mm	chr5	62858265	62858614	-	0	0	0
Simple_repeat	Simple_repeat	(A)n	chr5	63529151	63529192	+	0	0	0
Simple_repeat	Simple_repeat	(A)n	chr5	63949103	63949129	+	0	0	0
Simple_repeat	Simple_repeat	(A)n	chr5	64035374	64035404	+	0	0	0
SINE	Alu	B1_Mus1	chr5	64363734	64363881	+	0	0	0
SINE	Alu	B1_Mus2	chr5	65093923	65094048	-	0	0	0
SINE	Alu	B1_Mus2	chr5	65325893	65326039	+	0	0	0
SINE	Alu	B1_Mus1	chr5	66091919	66092066	-	0	0	0
Simple_repeat	Simple_repeat	(TTTA)n	chr5	66110272	66110295	+	0	0	0
SINE	Alu	B1_Mus2	chr5	66288482	66288627	+	0	0	0
Simple_repeat	Simple_repeat	(A)n	chr5	66288628	66288654	+	0	0	0
SINE	Alu	B1_Mus2	chr5	66345681	66345839	+	0	0	0
Simple_repeat	Simple_repeat	(A)n	chr5	66345840	66345865	+	0	0	0
Simple_repeat	Simple_repeat	(A)n	chr5	66348009	66348044	+	0	0	0
LTR	MaLR	MTB	chr5	67518841	67519242	+	0	0	0
SINE	Alu	B1_Mus2	chr11	53120850	53120994	-	0	0	0
SINE	B2	B2_Mm1t	chr11	53126638	53126823	-	0	0	0
Simple_repeat	Simple_repeat	(A)n	chr5	68394262	68394322	+	0	0	0
SINE	Alu	B1_Mus1	chr11	53132342	53132487	-	0	0	0
LTR	MaLR	MTA_Mm	chr5	68617578	68617972	+	0	0	0
LTR	MaLR	MTA_Mm	chr5	69043634	69044028	-	0	0	0
LINE	L1	L1_Mus3	chr5	70478162	70484368	-	0	0	0
LINE	L1	L1Md_F2	chr5	70906915	70912884	-	0	0	0
LTR	MaLR	MTA_Mm	chr5	70961348	70961743	+	0	0	0
SINE	Alu	B1_Mus1	chr11	53240693	53240847	-	0	0	0
SINE	Alu	B1_Mus1	chr5	71111707	71111854	-	0	0	0
Simple_repeat	Simple_repeat	(A)n	chr5	71954550	71954584	+	0	0	0
Simple_repeat	Simple_repeat	(GAA)n	chr5	73270814	73270864	+	0	0.380568	0.342623
SINE	Alu	B1_Mus2	chr5	73296706	73296840	-	0	0	0
SINE	Alu	B1_Mm	chr5	73757110	73757255	+	0	0	0
Simple_repeat	Simple_repeat	(A)n	chr5	73757256	73757297	+	0	0	0
LTR	MaLR	MTA_Mm	chr5	73777254	73777648	-	0	0	0
SINE	Alu	B1_Mm	chr5	74279284	74279430	+	0	0	0
LTR	MaLR	MTB	chr5	74351973	74352371	+	0	0	0.342623
Simple_repeat	Simple_repeat	(A)n	chr5	74468844	74468866	+	0	0	0
LTR	MaLR	MTB	chr5	75332738	75333130	+	0	0	0

LTR	MaLR	MTB	chr5	75765626	75766019	+	0	0	0
Simple_repeat	Simple_repeat	(A)n	chr5	76390995	76391036	+	0	0	0
Simple_repeat	Simple_repeat	(TTTA)n	chr5	76610227	76610267	+	0	0	0
SINE	Alu	B1_Mm	chr5	77400549	77400705	+	0	0	0
Simple_repeat	Simple_repeat	(A)n	chr5	77400706	77400729	+	0	0	0
LTR	MaLR	MTA_Mm	chr5	77554999	77555396	+	0	0	0
SINE	Alu	B1_Mur1	chr5	77666037	77666179	+	0	0	0
Simple_repeat	Simple_repeat	(A)n	chr5	77666180	77666203	+	0	0	0
SINE	Alu	B1_Mm	chr5	77720339	77720468	+	0	0	0
Simple_repeat	Simple_repeat	(A)n	chr5	77720469	77720492	+	0	0	0
LTR	MaLR	MTB	chr5	78069434	78069833	+	0	0	0
Simple_repeat	Simple_repeat	(A)n	chr5	79530078	79530137	+	0	0	0
LINE	L1	L1Md_F2	chr5	79629285	79634730	-	0	0	0
Simple_repeat	Simple_repeat	(A)n	chr5	79976441	79976537	+	0	0	0
LTR	MaLR	MTA_Mm	chr5	80180014	80180391	-	0	0	0
Simple_repeat	Simple_repeat	(A)n	chr5	80317087	80317131	+	0.591053	0	0.342623
Simple_repeat	Simple_repeat	(A)n	chr5	80699241	80699277	+	0	0	0
SINE	Alu	B1_Mus2	chr5	80929421	80929567	-	0	0	0
Simple_repeat	Simple_repeat	(A)n	chr5	80971609	80971652	+	0	0	0
Simple_repeat	Simple_repeat	(A)n	chr5	81028523	81028547	+	0	0	0
SINE	Alu	B1_Mus2	chr5	82457878	82458024	+	0	0	0
Simple_repeat	Simple_repeat	(A)n	chr5	82458025	82458064	+	0	0	0
SINE	Alu	B1_Mus1	chr5	82531052	82531198	+	0	0	0
Simple_repeat	Simple_repeat	(A)n	chr5	82531199	82531287	+	0	0	0
Simple_repeat	Simple_repeat	(A)n	chr5	82643972	82644013	+	0	0	0
SINE	Alu	B1_Mm	chr5	82768806	82768936	-	0	0	0
Simple_repeat	Simple_repeat	(A)n	chr5	82769985	82770019	+	0	0	0
SINE	Alu	B1_Mus2	chr5	82921272	82921417	+	0	0	0
Simple_repeat	Simple_repeat	(A)n	chr5	82921418	82921478	+	0	0	0
Simple_repeat	Simple_repeat	(A)n	chr5	83186090	83186129	+	0	0	0
Simple_repeat	Simple_repeat	(CAAAC)n	chr5	83348503	83348564	+	0	0	0
Simple_repeat	Simple_repeat	(A)n	chr5	83348565	83348588	+	0	0	0
Simple_repeat	Simple_repeat	(A)n	chr5	83354749	83354775	+	0	0	0
LTR	MaLR	MTA_Mm	chr5	83416919	83417314	-	0	0	0
Simple_repeat	Simple_repeat	(A)n	chr5	83603042	83603078	+	0	0	0
SINE	Alu	B1_Mur3	chr5	83622793	83622932	+	0	0	0
Low_complexity	Low_complexity	A-rich	chr5	83622933	83623037	+	0	0	0
Simple_repeat	Simple_repeat	(A)n	chr5	83818988	83819044	+	0	0	0
LTR	MaLR	MTB	chr5	84888623	84889016	-	0	0	0
Simple_repeat	Simple_repeat	(A)n	chr5	85645045	85645082	+	0	0	0
Simple_repeat	Simple_repeat	(A)n	chr5	85685292	85685323	+	0	0	0
Simple_repeat	Simple_repeat	(A)n	chr5	85726294	85726335	+	0	0	0
Simple_repeat	Simple_repeat	(A)n	chr11	54516067	54516091	+	0.923247	0.380568	0
LTR	MaLR	MTA_Mm	chr5	89714621	89715015	+	0	0	0
Simple_repeat	Simple_repeat	(A)n	chr5	90023587	90023623	+	0	0	0
SINE	Alu	B1_Mus2	chr11	54610982	54611127	-	0	0	0
Simple_repeat	Simple_repeat	(A)n	chr5	90357653	90357703	+	0	0	0
Low_complexity	Low_complexity	A-rich	chr5	90610415	90610542	+	0	0	0
Simple_repeat	Simple_repeat	(A)n	chr5	90617267	90617311	+	0	0	0
SINE	Alu	B1_Mus1	chr11	54646456	54646596	+	0	0	0
Simple_repeat	Simple_repeat	(A)n	chr11	54646597	54646619	+	0	0	0
LTR	MaLR	MTA_Mm	chr5	92157649	92158043	-	0	0	0
SINE	Alu	B1_Mm	chr5	92407018	92407161	+	0	0	0
Simple_repeat	Simple_repeat	(A)n	chr5	92407162	92407195	+	0	0	0
LTR	MaLR	MTA_Mm	chr5	93505586	93505980	-	0	0	0.615324
SINE	Alu	B1_Mus1	chr5	93508945	93509084	-	0	0	0
SINE	Alu	B1_Mm	chr5	93783060	93783206	+	0	0	0
SINE	Alu	PB1D9	chr5	93912102	93912210	+	0	0	0
Simple_repeat	Simple_repeat	(A)n	chr5	93912211	93912248	+	0	0	0
Simple_repeat	Simple_repeat	(CAAG)n	chr5	93912247	93912267	+	0	0	0
SINE	Alu	B1_Mus2	chr5	93926219	93926354	-	0	0	0
SINE	Alu	B1_Mus2	chr5	94269164	94269299	+	0	0	0
SINE	Alu	PB1D9	chr5	94283295	94283403	-	0	0	0
SINE	Alu	B1_Mus1	chr5	94363164	94363309	+	0	0	0
Simple_repeat	Simple_repeat	(A)n	chr5	94363310	94363351	+	0	0	0
SINE	Alu	B1_Mus2	chr5	94635088	94635224	+	0.332194	0	0
SINE	Alu	B1_Mus2	chr5	94951518	94951664	+	0	0	0.342623

SINE	Alu	B1_Mus2	chr5	95290737	95290873	+	0	0	0
Simple_repeat	Simple_repeat	(A)n	chr5	95310346	95310382	+	0	0	0
SINE	Alu	B1_Mus2	chr11	54909120	54909265	+	0	0	0
Simple_repeat	Simple_repeat	(A)n	chr11	54909266	54909287	+	0	0	0
SINE	Alu	B1_Mus1	chr5	95954363	95954510	+	0	0	0
Simple_repeat	Simple_repeat	(A)n	chr5	95954511	95954552	+	0	0	0
SINE	Alu	B1_Mus1	chr5	96028980	96029125	+	0	0	0
Simple_repeat	Simple_repeat	(A)n	chr5	96029126	96029163	+	0	0	0
Simple_repeat	Simple_repeat	(A)n	chr5	96428828	96428867	+	0	0	0
Simple_repeat	Simple_repeat	(A)n	chr5	98608362	98608397	+	0	0	0
Simple_repeat	Simple_repeat	(TTTA)n	chr5	98733725	98733775	+	0	0	0
LINE	L1	L1Md_T	chr5	99338414	99344953	-	0	0	0
Simple_repeat	Simple_repeat	(A)n	chr5	99555181	99555208	+	0	0	0
Simple_repeat	Simple_repeat	(A)n	chr5	10015896	10015901	+	0	0	0
LTR	MaLR	MTA_Mm	chr5	10027225	10027264	+	0	0	0
LTR	MaLR	MTA_Mm	chr5	10036511	10036550	+	0.591053	0	0
LTR	MaLR	MTA_Mm	chr10	22138463	22138857	-	0	0	0
SINE	Alu	B1_Mm	chr5	10118768	10118782	+	0	0	0
Simple_repeat	Simple_repeat	(A)n	chr5	10118782	10118785	+	0	0	0
Simple_repeat	Simple_repeat	(A)n	chr5	10153720	10153723	+	0	0	0
Simple_repeat	Simple_repeat	(A)n	chr5	10186717	10186721	+	0	0.380568	0
Simple_repeat	Simple_repeat	(A)n	chr5	10211826	10211833	+	0	0	0
SINE	Alu	B1_Mus1	chr5	10217810	10217824	+	0	0	0
LINE	L1	L1Md_T	chr5	10261982	10262645	-	0	0	0
SINE	Alu	B1_Mm	chr5	10364210	10364224	-	0	0	0
Simple_repeat	Simple_repeat	(A)n	chr5	10379763	10379766	+	0	0	0.615324
SINE	Alu	B1_Mus2	chr5	10414354	10414369	-	0	0	0
Simple_repeat	Simple_repeat	(A)n	chr5	10415779	10415783	+	0	0	0
SINE	Alu	B1_Mus2	chr5	10417251	10417266	-	0	0	0
SINE	Alu	B1_Mus2	chr5	10513426	10513441	-	0	0	0
SINE	Alu	B1_Mm	chr5	10519679	10519692	-	0	0	0
SINE	Alu	B1_Mm	chr5	10582260	10582275	-	0	0	0
SINE	Alu	B1_Mm	chr5	10610757	10610771	+	0	0	0
Simple_repeat	Simple_repeat	(A)n	chr5	10610771	10610775	+	0	0	0
Simple_repeat	Simple_repeat	(A)n	chr5	10611493	10611496	+	0	0	0
SINE	B2	B2_Mm1t	chr5	10629386	10629405	+	0	0	0
Simple_repeat	Simple_repeat	(A)n	chr5	10629406	10629407	+	0	0	0
SINE	B2	B2_Mm1t	chr5	10629407	10629426	+	0	0	0
Simple_repeat	Simple_repeat	(A)n	chr5	10629426	10629427	+	0	0	0
Simple_repeat	Simple_repeat	(A)n	chr5	10640448	10640451	+	0	0	0
LTR	MaLR	MTA_Mm	chr5	10668780	10668819	+	0	0	0
LTR	MaLR	MTB	chr10	22199858	22200191	+	0	0	0
Simple_repeat	Simple_repeat	(A)n	chr5	10725838	10725843	+	0	0	0
SINE	Alu	B1_Mus1	chr5	10795538	10795551	-	0	0	0
SINE	Alu	B1_Mus2	chr5	10813602	10813616	+	0	0	0
Simple_repeat	Simple_repeat	(GAAAA)n	chr5	10813616	10813620	+	0	0	0
SINE	Alu	PB1D9	chr5	10831453	10831464	+	0	0	0
Simple_repeat	Simple_repeat	(A)n	chr5	10831464	10831471	+	0	0	0
SINE	Alu	B1_Mus2	chr5	10843055	10843070	+	0	0	0.342623
Simple_repeat	Simple_repeat	(A)n	chr5	10843070	10843077	+	0	0	0.342623
Simple_repeat	Simple_repeat	(A)n	chr5	10888388	10888392	+	0	0	0
SINE	Alu	B1_Mus2	chr5	10911801	10911816	-	0	0	0
LTR	MaLR	MTA_Mm	chr5	11001030	11001070	+	0	0	0
SINE	Alu	B1_Mus1	chr5	11003603	11003618	+	0	0	0
Simple_repeat	Simple_repeat	(A)n	chr5	11003618	11003622	+	0	0	0
Simple_repeat	Simple_repeat	(A)n	chr5	11017650	11017652	+	0	0	0
SINE	B2	B2_Mm1a	chr5	11090510	11090529	-	0	0	0
LTR	MaLR	MTA_Mm	chr5	11094239	11094278	-	0	0	0
SINE	Alu	B1_Mus1	chr5	11174058	11174072	-	0	0	0
LTR	MaLR	MTA_Mm	chr5	11189168	11189207	-	0	0	0
SINE	Alu	B1_Mm	chr11	56285979	56286125	+	0	0	0
Simple_repeat	Simple_repeat	(A)n	chr11	56286126	56286166	+	0.332194	0.380568	0
SINE	Alu	B1_Mus2	chr5	11264159	11264174	-	0	0	0
SINE	Alu	B1_Mus1	chr5	11493345	11493360	-	0	0	0
SINE	Alu	B1_Mur4	chr5	11500571	11500586	+	0	0	0
SINE	Alu	B1_Mm	chr5	11501286	11501300	+	0	0	0
SINE	Alu	B1_Mus2	chr5	11515496	11515510	+	0	0	0

LTR	ERVK	RMER4A	chr5	11533828	11533865	-	0	0	0
SINE	Alu	B1_Mm	chr5	11533867	11533882	-	0	0	0
SINE	Alu	B1_Mm	chr5	11544114	11544127	+	0	0	0
Simple_repeat	Simple_repeat	(A)n	chr5	11544127	11544132	+	0	0	0
Simple_repeat	Simple_repeat	(A)n	chr5	11545899	11545906	+	0	0	0
LINE	L1	L1Md_T	chr11	56613952	56620865	-	0	0	0
SINE	Alu	B1_Mus2	chr5	11575244	11575258	+	0	1.41763	0.615324
Simple_repeat	Simple_repeat	(A)n	chr5	11575258	11575261	+	0	0.708814	0
SINE	Alu	B1_Mus2	chr5	11628057	11628070	-	0	0	0
LTR	MaLR	MTA_Mm	chr11	56753799	56754193	+	0	0	0
SINE	Alu	B1_Mm	chr5	11648397	11648412	+	0	0	0
Simple_repeat	Simple_repeat	(A)n	chr5	11648412	11648414	+	0	0	0
Simple_repeat	Simple_repeat	(A)n	chr5	11653803	11653806	+	0	0	0
Simple_repeat	Simple_repeat	(A)n	chr5	11676275	11676278	+	0	0	0
Simple_repeat	Simple_repeat	(A)n	chr11	56815458	56815499	+	0	0	0
Simple_repeat	Simple_repeat	(A)n	chr5	11714543	11714546	+	0	0	0
SINE	Alu	B1_Mus1	chr5	11725848	11725863	+	0	0	0
Simple_repeat	Simple_repeat	(A)n	chr5	11725863	11725871	+	0	0	0
Simple_repeat	Simple_repeat	(A)n	chr5	11748150	11748154	+	0	0	0
SINE	Alu	B1_Mus2	chr10	22329454	22329609	-	0.591053	0	0
Simple_repeat	Simple_repeat	(A)n	chr5	11893180	11893183	+	0	0	0
SINE	Alu	B1_Mus2	chr5	12010313	12010327	+	0	0	0
SINE	B2	B3	chr5	12054387	12054406	+	0	0	0
SINE	B2	B2_Mm2	chr5	12055451	12055470	-	0	0	0
LTR	MaLR	MTA_Mm	chr11	57289302	57289695	-	0	0	0
LTR	MaLR	MTA_Mm	chr11	57382178	57382563	-	0	0	0
SINE	Alu	PB1D9	chr11	57395550	57395642	-	0.332194	0	0
SINE	Alu	B1_Mus2	chr5	12195798	12195812	+	0	0	0
SINE	Alu	B1_Mus1	chr5	12201038	12201053	+	0	0	0
SINE	Alu	B1_Mus1	chr5	12254008	12254022	-	0	0	0
SINE	Alu	B1_Mm	chr5	12254544	12254559	-	0	0	0
SINE	Alu	B1_Mus1	chr5	12259009	12259024	-	0	0	0
Simple_repeat	Simple_repeat	(A)n	chr5	12264242	12264245	+	0	0	0
SINE	Alu	B1_Mm	chr5	12265637	12265652	+	0	0	0
Simple_repeat	Simple_repeat	(A)n	chr5	12265652	12265655	+	0.332194	0	0
SINE	Alu	B1_Mus2	chr11	57603254	57603399	-	0	0	0
SINE	B2	B2_Mm1a	chr5	12351133	12351152	-	0	0	0
SINE	Alu	B1_Mm	chr11	57639185	57639330	-	0.332194	0	0
LTR	MaLR	MTB	chr5	12417442	12417478	-	0	0	0
SINE	Alu	B1_Mus2	chr5	12450752	12450766	-	0	0	0
SINE	Alu	B1_Mus1	chr5	12480938	12480953	+	0	0	0
Simple_repeat	Simple_repeat	(A)n	chr5	12480953	12480957	+	0	0	0
SINE	Alu	B1_Mm	chr5	12537998	12538012	+	0	0	0
SINE	Alu	B1_Mus2	chr5	12544245	12544260	-	0	0	0
LTR	MaLR	MTA_Mm	chr5	12567835	12567875	-	0	0	0
Simple_repeat	Simple_repeat	(A)n	chr5	12582225	12582229	+	0	0	0
SINE	Alu	B1_Mus2	chr5	12583795	12583809	-	0	0	0
LTR	MaLR	MTA_Mm	chr5	12585652	12585691	+	0	0	0
SINE	Alu	B1_Mm	chr11	57792362	57792507	-	0	0	0
Simple_repeat	Simple_repeat	(A)n	chr11	57799091	57799121	+	0	0	0
SINE	Alu	B1_Mur4	chr5	12601079	12601088	+	0	0	0
Simple_repeat	Simple_repeat	(GAAA)n	chr5	12601088	12601093	+	0	0	0
Simple_repeat	Simple_repeat	(A)n	chr5	12673716	12673719	+	0	0	0
Simple_repeat	Simple_repeat	(A)n	chr5	12718284	12718287	+	0	0	0
Simple_repeat	Simple_repeat	(A)n	chr5	12737537	12737542	+	0	0	0
LTR	MaLR	MTB	chr5	12740200	12740238	+	0	0	0
Simple_repeat	Simple_repeat	(A)n	chr5	12760821	12760824	+	0	0	0
SINE	Alu	B1_Mm	chr11	57994097	57994242	-	0	0	0
Simple_repeat	Simple_repeat	(A)n	chr5	12895650	12895653	+	0	0	0
LTR	MaLR	MTA_Mm	chr5	12917873	12917911	+	0	0	0
SINE	Alu	B1_Mus1	chr5	12958554	12958568	+	0	0	0
Simple_repeat	Simple_repeat	(A)n	chr5	12958568	12958572	+	0	0	0
LTR	MaLR	MTA_Mm	chr5	12974722	12974761	-	0	0	0
SINE	Alu	B1_Mm	chr5	12987624	12987637	+	0	0	0
Simple_repeat	Simple_repeat	(A)n	chr5	12987637	12987640	+	0	0	0
LTR	MaLR	MTA_Mm	chr5	13007832	13007871	-	0	0	0
SINE	Alu	B1_Mm	chr5	13063677	13063692	+	0	0	0

Simple_repeat	Simple_repeat	(A)n	chr5	13063692	13063695	+	0.332194	0	0
SINE	Alu	B1_Mus1	chr11	58208775	58208921	+	0	0	0
Simple_repeat	Simple_repeat	(A)n	chr11	58208922	58208946	+	0	0	0
SINE	B4	ID_B1	chr5	13190230	13190251	-	0	0	0
LTR	MaLR	MTA_Mm	chr5	13308131	13308171	-	0	0	0
SINE	Alu	PB1D10	chr10	22573493	22573609	-	0	0	0
SINE	Alu	B1_Mm	chr11	58675838	58675982	+	0	0	0.342623
Simple_repeat	Simple_repeat	(A)n	chr11	58675983	58676005	+	0	0	0.342623
Simple_repeat	Simple_repeat	(A)n	chr5	13395145	13395151	+	5.90476	1.41763	2.53122
SINE	Alu	B1_Mus2	chr5	13481937	13481952	+	0	0	0
SINE	Alu	B1_Mus2	chr5	13504615	13504629	+	0	0	0
Simple_repeat	Simple_repeat	(A)n	chr5	13504629	13504632	+	0	0	0
SINE	Alu	B1_Mm	chr11	59047127	59047278	-	0	0	0
SINE	Alu	B1_Mus1	chr5	13587703	13587718	-	0	0	0
SINE	Alu	B1_Mus1	chr5	13615815	13615829	+	0.332194	0	0
Simple_repeat	Simple_repeat	(A)n	chr5	13615830	13615832	+	0.332194	0	0
SINE	Alu	B1_Mm	chr5	13643059	13643073	+	0	0	0
Simple_repeat	Simple_repeat	(A)n	chr5	13643073	13643076	+	0	0	0
SINE	B4	ID_B1	chr5	13664905	13664924	-	0.591053	0	0
SINE	Alu	B1_Mm	chr5	13709832	13709847	-	0	0	0
SINE	Alu	B1_Mus1	chr11	59287185	59287332	-	0	0	0
SINE	Alu	B1_Mm	chr5	13753219	13753234	-	0	0	0
Simple_repeat	Simple_repeat	(A)n	chr11	59337230	59337265	+	0	0	0
SINE	Alu	B1_Mm	chr5	13792024	13792039	+	0	0	0
Simple_repeat	Simple_repeat	(A)n	chr5	13818650	13818654	+	0	0	0
SINE	Alu	B1_Mm	chr5	13898524	13898538	-	0	0	0
SINE	Alu	B1_Mm	chr5	13966478	13966492	+	0	0	0
Simple_repeat	Simple_repeat	(A)n	chr5	13966492	13966494	+	0	0	0
Simple_repeat	Simple_repeat	(TAA)n	chr5	13966494	13966497	+	0	0	0
SINE	Alu	PB1D10	chr5	14031586	14031596	-	0	0	0
SINE	B2	B2_Mm1a	chr11	59714965	59715160	-	0.591053	0.380568	0
SINE	Alu	B1_Mus1	chr11	59720335	59720437	-	0	0	0
SINE	Alu	B1_Mm	chr5	14087719	14087731	+	0	0	0
Low_complexity	Low_complexity	A-rich	chr5	14087731	14087738	+	0	0	0
SINE	Alu	B1_Mus2	chr5	14092443	14092458	+	0	0	0
Simple_repeat	Simple_repeat	(A)n	chr5	14092458	14092461	+	0	0	0
SINE	Alu	B1_Mm	chr5	14092663	14092677	+	0	0	0
Simple_repeat	Simple_repeat	(A)n	chr5	14092677	14092679	+	0	0	0
SINE	Alu	B1_Mus2	chr5	14109164	14109178	+	0	0.708814	0
Simple_repeat	Simple_repeat	(A)n	chr5	14109178	14109183	+	0.332194	0.380568	0.342623
Simple_repeat	Simple_repeat	(A)n	chr5	14283082	14283087	+	0	0	0.342623
SINE	B4	ID_B1	chr5	14317004	14317024	-	0	0	0
SINE	Alu	B1_Mus1	chr5	14362156	14362170	-	0	0.380568	0
SINE	Alu	B1_Mus1	chr5	14388808	14388823	-	0	0	0
SINE	Alu	B1_Mus2	chr5	14483514	14483528	+	0	0	0.615324
Simple_repeat	Simple_repeat	(A)n	chr5	14483528	14483538	+	0	0	0.615324
SINE	Alu	B1_Mus1	chr5	14519740	14519754	+	0	0	0
SINE	Alu	B1_Mus2	chr5	14574046	14574061	+	0	0	0
Simple_repeat	Simple_repeat	(A)n	chr5	14574061	14574064	+	0	0	0
Simple_repeat	Simple_repeat	(A)n	chr5	14605469	14605472	+	0.591053	0	0
Simple_repeat	Simple_repeat	(A)n	chr5	14625742	14625746	+	0	0	0.615324
SINE	Alu	B1_Mus2	chr5	14701206	14701220	-	1.18211	0.708814	1.23065
SINE	Alu	B1_Mus1	chr5	14719111	14719126	-	0	0	0
Simple_repeat	Simple_repeat	(A)n	chr11	60450611	60450650	+	0	0	0.342623
SINE	Alu	B1_Mus1	chr5	14738861	14738876	+	0	0	0
Simple_repeat	Simple_repeat	(A)n	chr5	14738876	14738878	+	0	0	0
SINE	Alu	B1_Mm	chr5	14753276	14753290	-	0	0	0
LTR	MaLR	MTB	chr5	14754267	14754307	-	0	0	0.615324
SINE	Alu	B1_Mus1	chr5	14763900	14763914	-	0	0	0
Simple_repeat	Simple_repeat	(A)n	chr5	14777545	14777549	+	0	0	0
SINE	Alu	B1_Mus1	chr5	14779089	14779104	-	0	0	0
SINE	Alu	B1_Mm	chr5	14813664	14813679	-	0	0	0
SINE	Alu	B1_Mur4	chr5	14836583	14836597	-	0	0	0
LTR	MaLR	MTA_Mm	chr5	14967052	14967091	-	0	0	0
SINE	Alu	B1_Mm	chr5	14974125	14974139	+	0	0	0
Simple_repeat	Simple_repeat	(A)n	chr5	14974139	14974142	+	0	0	0
SINE	Alu	B1_Mus1	chr5	14975644	14975658	-	0	0	0.615324

LTR	MaLR	MTB	chr5	14984086	14984126	+	0	0	0
SINE	Alu	B1_Mus2	chr11	60782836	60782982	-	0	0	0
SINE	Alu	B1_Mus2	chr5	15171229	15171243	+	0	0	0
Simple_repeat	Simple_repeat	(A)n	chr5	15171244	15171246	+	0	0	0
SINE	Alu	B1_Mm	chr5	15171584	15171599	-	0	0	0
SINE	Alu	B1_Mm	chr5	15228304	15228318	+	0	0	0
Simple_repeat	Simple_repeat	(A)n	chr5	15228318	15228320	+	0	0	0
LINE	L1	L1_Mus4	chr5	15241732	15242262	-	0	0	0
Simple_repeat	Simple_repeat	(A)n	chr5	15246493	15246497	+	0	0	0.342623
LTR	MaLR	MTA_Mm	chr5	15250322	15250362	-	0	0	0
LTR	MaLR	MTA_Mm	chr5	15250471	15250511	-	0	0	0
LTR	MaLR	MTA_Mm	chr6	3398148	3398543	+	0	0	0
LTR	MaLR	MTA_Mm	chr6	3399641	3400036	+	0	0	0
Simple_repeat	Simple_repeat	(A)n	chr6	4388908	4388935	+	0	0	0
LTR	MaLR	MTA_Mm	chr6	5402618	5403012	+	0	0	0
SINE	Alu	PB1D9	chr6	5508419	5508519	-	0	0	0
SINE	Alu	B1_Mus2	chr6	5634784	5634929	-	0	0	0
LTR	MaLR	MTA_Mm	chr6	5635993	5636388	+	0	0	0
SINE	Alu	B1_Mm	chr6	6298543	6298690	-	0.591053	0	0
SINE	Alu	B1_Mus1	chr6	6668116	6668263	+	0	0	0
Simple_repeat	Simple_repeat	(A)n	chr6	6668264	6668290	+	0	0	0
SINE	Alu	B1_Mus2	chr6	7223290	7223435	+	0	0	0
Simple_repeat	Simple_repeat	(A)n	chr6	7223436	7223472	+	0	0	0
Simple_repeat	Simple_repeat	(A)n	chr6	7256755	7256830	+	0	0	0
Simple_repeat	Simple_repeat	(A)n	chr6	7689470	7689501	+	0	0	0
LTR	ERV1	RLTR24	chr6	7703849	7704360	+	0	0	0
SINE	Alu	B1_Mus1	chr6	7713447	7713594	-	0	0	0
Simple_repeat	Simple_repeat	(A)n	chr11	61328999	61329058	+	1.18211	0	1.84597
Simple_repeat	Simple_repeat	(A)n	chr6	9228117	9228174	+	0	0	0
SINE	Alu	B1_Mus2	chr6	9282774	9282915	-	0	0	0
SINE	Alu	B1_Mus1	chr11	61405169	61405302	+	0	0	0
Simple_repeat	Simple_repeat	(A)n	chr11	61405303	61405335	+	0	0	0
Simple_repeat	Simple_repeat	(A)n	chr6	9543303	9543341	+	0	0	0
LTR	MaLR	MTA_Mm	chr6	10749687	10750081	-	0	0	0
LINE	L1	L1Md_F2	chr6	10754546	10759533	+	0	0	0
LTR	MaLR	MTA_Mm	chr6	11003273	11003668	-	0	0	0
Simple_repeat	Simple_repeat	(A)n	chr6	11142889	11142916	+	0	0	0
LTR	ERVK	BGLII	chr6	11293183	11293638	-	0	0	0
LINE	L1	L1Md_T	chr6	11692603	11699447	-	0	0	0
SINE	Alu	B1_Mus2	chr6	12086938	12087084	+	0.591053	0	0.615324
Simple_repeat	Simple_repeat	(A)n	chr6	12087085	12087107	+	0	0	0
LINE	L1	L1Md_F2	chr6	12173286	12179332	-	0	0	0
Simple_repeat	Simple_repeat	(A)n	chr6	13349775	13349815	+	0	0	0
LTR	MaLR	MTA_Mm	chr6	13431456	13431847	-	0	0	0
LTR	MaLR	MTA_Mm	chr6	13668024	13668422	-	0	0	0
Simple_repeat	Simple_repeat	(A)n	chr6	14191269	14191296	+	0	0	0
Simple_repeat	Simple_repeat	(A)n	chr6	14440562	14440598	+	0	0	0
Simple_repeat	Simple_repeat	(A)n	chr6	15474851	15474889	+	0.591053	0	0
Simple_repeat	Simple_repeat	(A)n	chr6	15505807	15505833	+	0	0	0
SINE	Alu	B1_Mm	chr11	61755420	61755565	-	0	0	0
SINE	Alu	B1_Mus2	chr6	15846488	15846605	-	0	0	0
Simple_repeat	Simple_repeat	(A)n	chr6	16281944	16281972	+	0	0	0
Simple_repeat	Simple_repeat	(A)n	chr6	16983599	16983641	+	0	0.708814	0.615324
Simple_repeat	Simple_repeat	(A)n	chr6	17536925	17536951	+	0	0	0
SINE	Alu	B1_Mus1	chr6	17655804	17655947	+	0	0	0
SINE	Alu	B1_Mus2	chr6	17694394	17694540	-	0	0	0
LINE	L1	L1Md_T	chr6	18112286	18119018	-	0	0	0
Simple_repeat	Simple_repeat	(A)n	chr6	18483469	18483506	+	0	0	0
LINE	L1	L1Md_F2	chr6	18957307	18962717	-	0	0	0
LTR	MaLR	MTA_Mm	chr6	19357503	19357896	-	0	0	0
LINE	L1	L1Md_F	chr6	19616859	19621345	-	0	0	0
Simple_repeat	Simple_repeat	(A)n	chr6	19837747	19837775	+	0	0	0
SINE	Alu	B1_Mus2	chr6	19847553	19847698	-	0	0	0
Simple_repeat	Simple_repeat	(A)n	chr6	19893176	19893228	+	0	0	0
LINE	L1	L1Md_F3	chr6	19927306	19933485	-	0	0	0
LTR	MaLR	MTA_Mm	chr6	20225638	20226031	+	0	0	0
LINE	L1	L1_Mus3	chr6	20417553	20422686	+	0	0	0

LINE	L1	L1Md_F2	chr6	20557760	20562957	+	0	0	0
LINE	L1	L1Md_F2	chr6	20578973	20585158	+	0	0	0
Simple_repeat	Simple_repeat	(A)n	chr6	21806341	21806369	+	0	0	0
LTR	MaLR	MTA_Mm	chr6	21882690	21883084	+	0	0	0
LTR	MaLR	MTA_Mm	chr6	22713729	22714124	-	0	0	0
SINE	Alu	B1_Mus2	chr6	25552394	25552540	+	0	0	0
Simple_repeat	Simple_repeat	(A)n	chr6	25552541	25552563	+	0	0	0
SINE	Alu	B1_Mus2	chr6	26241579	26241726	-	0	0	0
SINE	Alu	B1_Mus1	chr6	26343175	26343322	-	0	0	0
LTR	MaLR	MTA_Mm	chr6	26546873	26547268	+	0	0	0
Simple_repeat	Simple_repeat	(A)n	chr11	62387588	62387626	+	0	0	0
SINE	Alu	B1_Mus2	chr6	26767094	26767242	+	0	0	0
Simple_repeat	Simple_repeat	(A)n	chr6	26767243	26767264	+	0	0	0
LINE	L1	L1Md_T	chr6	26824939	26831447	-	0	0	0
SINE	Alu	B1_Mus1	chr6	26896625	26896771	+	0	0	0
Simple_repeat	Simple_repeat	(A)n	chr6	26896772	26896811	+	0	0	0
SINE	Alu	B1_Mm	chr11	62413844	62413989	+	0.591053	0	0
Simple_repeat	Simple_repeat	(A)n	chr11	62413990	62414012	+	0.923247	0	0
SINE	Alu	B1_Mm	chr6	28258854	28258999	-	0	0	0.342623
Simple_repeat	Simple_repeat	(A)n	chr6	28881069	28881095	+	0	0	0
SINE	Alu	B1_Mm	chr6	29167671	29167819	+	0	0.380568	0.342623
Simple_repeat	Simple_repeat	(A)n	chr6	29379569	29379608	+	0	0	0
SINE	Alu	B1_Mm	chr6	29575307	29575453	-	0	0	0
SINE	Alu	B1_Mus1	chr6	29670647	29670793	+	0	0	0
Simple_repeat	Simple_repeat	(A)n	chr6	29670794	29670816	+	0	0	0
SINE	Alu	B1_Mus2	chr6	29990728	29990874	-	0	0	0
SINE	B2	B2_Mm1a	chr6	29992086	29992277	+	0	0	0
SINE	Alu	B1_Mur3	chr6	30106239	30106379	+	0	0	0
SINE	Alu	B1_Mus1	chr6	30155395	30155540	-	0	0	0
SINE	Alu	B1_Mus2	chr6	30273183	30273322	-	0	0	0
SINE	Alu	B1_Mus2	chr6	30307131	30307257	+	0	0	0
Simple_repeat	Simple_repeat	(A)n	chr6	30307258	30307280	+	0	0	0
SINE	Alu	B1_Mus2	chr6	31462125	31462270	+	4.41242	2.17876	3.07662
Simple_repeat	Simple_repeat	(A)n	chr6	31462271	31462300	+	5.07681	2.17876	2.87384
Simple_repeat	Simple_repeat	(A)n	chr6	31890244	31890278	+	0	0	0
LTR	MaLR	MTA_Mm	chr6	32719156	32719547	+	0	0	0
SINE	Alu	B1_Mm	chr11	63142516	63142664	-	0	0	0
SINE	Alu	B1_Mus2	chr11	63170006	63170150	-	0	0	0
LINE	L1	L1Md_T	chr6	35289469	35296397	-	0	0	0
SINE	Alu	B1_Mus1	chr6	35873431	35873557	+	0	0	0
Simple_repeat	Simple_repeat	(A)n	chr6	35873558	35873594	+	0	0	0
Simple_repeat	Simple_repeat	(A)n	chr6	35944656	35944705	+	0	0	0
SINE	Alu	B1_Mus1	chr11	63321623	63321747	-	0	0	0
SINE	Alu	B1_Mus1	chr6	36481254	36481404	+	0	0	0
Simple_repeat	Simple_repeat	(A)n	chr6	36481405	36481446	+	0	0	0
LTR	MaLR	MTA_Mm	chr6	36502702	36503095	+	0	0	0
SINE	Alu	B1_Mm	chr6	36580608	36580752	+	0	0	0
Low_complexity	Low_complexity	A-rich	chr6	36580753	36580923	+	0	0	0
LTR	MaLR	MTB	chr6	37545469	37545856	+	0	0	0
LTR	MaLR	MTA_Mm	chr6	37549963	37550355	+	0	0	0
LTR	MaLR	MTA_Mm	chr6	37666145	37666536	-	0	0	0
SINE	Alu	B1_Mus2	chr6	37710966	37711112	+	0	0	0
Simple_repeat	Simple_repeat	(A)n	chr6	37711113	37711153	+	0	0	0.342623
SINE	Alu	B1_Mus2	chr6	37743689	37743835	+	0	0	0
Simple_repeat	Simple_repeat	(A)n	chr6	37743836	37743894	+	0.332194	0	0
LTR	MaLR	MTA_Mm	chr6	37772082	37772477	-	0	0	0
SINE	Alu	B1_Mus2	chr6	38256162	38256308	+	0	0	0
Simple_repeat	Simple_repeat	(A)n	chr6	38256309	38256332	+	0	0	0
SINE	Alu	B1_Mus2	chr6	38257914	38258060	-	0	0	0
Simple_repeat	Simple_repeat	(A)n	chr6	38307100	38307126	+	0	0	0
SINE	Alu	B1_Mus2	chr6	38382693	38382815	+	0	0	0
Simple_repeat	Simple_repeat	(A)n	chr6	38382816	38382841	+	0	0	0
SINE	Alu	B1_Mus2	chr6	38463934	38464084	-	0	0	0
SINE	Alu	B1_Mus2	chr6	38478814	38478962	+	0	0.708814	0
Simple_repeat	Simple_repeat	(A)n	chr6	38478963	38478987	+	0	0	0
Simple_repeat	Simple_repeat	(A)n	chr6	39700292	39700365	+	0	0	0
LTR	MaLR	MTA_Mm	chr6	39764714	39765109	+	0	0	0

LTR	MaLR	MTA_Mm	chr6	39785203	39785597	+	0	0	0
LTR	MaLR	MTB	chr6	40208450	40208849	-	0	0	0
LTR	MaLR	MTA_Mm	chr6	40272308	40272690	+	0	0	0
SINE	Alu	B1_Mm	chr6	41293714	41293859	-	0	0	0
SINE	Alu	B1_Mm	chr6	42364540	42364685	-	0	0	0
LTR	MaLR	MTA_Mm	chr6	42367216	42367592	-	0	0	0
LTR	MaLR	MTA_Mm	chr6	42387774	42388167	+	0	0	0
LTR	MaLR	MTA_Mm	chr6	42389260	42389653	+	0	0	0
LTR	MaLR	MTA_Mm	chr6	42663259	42663657	+	0	0	0
SINE	Alu	B1_Mus2	chr6	42825413	42825559	+	0	0	0
Simple_repeat	Simple_repeat	(A)n	chr6	42825560	42825599	+	0	0	0
SINE	Alu	B1_Mm	chr6	43986930	43987067	-	0	0	0
SINE	Alu	B1_Mus2	chr6	44277313	44277459	+	0	0	0
LTR	MaLR	MTA_Mm	chr6	44413856	44414256	-	0	0	0
LINE	L1	L1Md_F2	chr6	44503082	44509533	-	0	0	0
LTR	MaLR	MTA_Mm	chr11	64502426	64502809	+	0	0	0
LTR	MaLR	MTA_Mm	chr6	47263151	47263546	-	0	0	0
LTR	MaLR	MTA_Mm	chr6	47301197	47301592	-	0	0	0
SINE	Alu	B1_Mm	chr6	47336067	47336212	-	0	0	0
SINE	Alu	B1_Mus1	chr6	47366480	47366625	-	0	0	0
scRNA	scRNA	4.5SRNA	chr6	47600761	47600854	-	0.332194	1.32017	0.342623
scRNA	scRNA	4.5SRNA	chr6	47698497	47698590	-	3.13357	0	2.32725
scRNA	scRNA	4.5SRNA	chr6	47707665	47707758	-	0	0	0
scRNA	scRNA	4.5SRNA	chr6	47713263	47713356	-	0.332194	0.380568	4.24948
scRNA	scRNA	4.5SRNA	chr6	47717586	47717660	-	0	0	0
SINE	Alu	PB1D10	chr6	47717592	47717687	-	0	0	0
scRNA	scRNA	4.5SRNA	chr6	47721993	47722067	-	0	0	0
SINE	Alu	PB1D10	chr6	47721999	47722094	-	0	0	0
scRNA	scRNA	4.5SRNA	chr6	47728245	47728338	-	5.81523	3.69765	3.56423
SINE	Alu	B1_Mus2	chr6	47864837	47864982	-	0	0	0
Simple_repeat	Simple_repeat	(A)n	chr6	48239128	48239167	+	0	0	0
Simple_repeat	Simple_repeat	(A)n	chr6	48305289	48305332	+	0	0	0
SINE	Alu	B1_Mm	chr6	48624566	48624710	+	0	0	0
Simple_repeat	Simple_repeat	(A)n	chr6	48624711	48624738	+	0	0	0
Simple_repeat	Simple_repeat	(A)n	chr11	64766419	64766454	+	0	0	0
SINE	Alu	B1_Mus1	chr6	50149669	50149807	-	0	0	0
SINE	Alu	B1_Mur4	chr6	50695445	50695603	+	0	0	0
Simple_repeat	Simple_repeat	(GAAA)n	chr6	50695604	50695666	+	0	0	0
Simple_repeat	Simple_repeat	(A)n	chr6	50980689	50980715	+	0	0	0
SINE	Alu	B1_Mus1	chr6	51448730	51448876	-	0	0	0
SINE	Alu	B1_Mm	chr6	51603437	51603594	+	0	0	0
Simple_repeat	Simple_repeat	(A)n	chr6	51603595	51603616	+	0	0	0
SINE	Alu	B1_Mur2	chr6	52018187	52018324	+	0	0	0
Simple_repeat	Simple_repeat	(A)n	chr6	52018325	52018358	+	0	0	0
SINE	Alu	B1_Mur1	chr6	52059063	52059207	-	0	0	0
SINE	Alu	B1_Mm	chr6	52084386	52084531	+	0	0	0
Simple_repeat	Simple_repeat	(A)n	chr6	52084532	52084566	+	0	0	0
SINE	Alu	B1_Mus1	chr6	52355513	52355661	-	0	0	0
SINE	Alu	B1_Mus1	chr6	52402920	52403067	+	0	0	0
Simple_repeat	Simple_repeat	(A)n	chr6	52403068	52403091	+	0.332194	0	0
SINE	Alu	B1_Mm	chr11	65158937	65159082	-	0	0	0
SINE	Alu	B1_Mm	chr6	52627790	52627936	-	0	0	0
LTR	MaLR	MTA_Mm	chr6	53414214	53414596	+	0	0	0
LTR	MaLR	MTA_Mm	chr6	53415694	53416076	+	0.332194	0.708814	0
Simple_repeat	Simple_repeat	(A)n	chr6	53488980	53489016	+	0	0	0
Simple_repeat	Simple_repeat	(A)n	chr6	53715270	53715295	+	0	0	0
SINE	Alu	B1_Mus1	chr6	54679987	54680134	-	0	0	0
SINE	Alu	B1_Mus2	chr6	55568502	55568647	+	0	0	0
Simple_repeat	Simple_repeat	(A)n	chr6	55568648	55568697	+	0	0	0
SINE	Alu	B1_Mus2	chr6	56664136	56664281	+	0	0	0
Simple_repeat	Simple_repeat	(A)n	chr6	56664282	56664304	+	0	0	0
Simple_repeat	Simple_repeat	(A)n	chr6	57162240	57162281	+	0	0	0
SINE	Alu	B1_Mm	chr6	57197913	57198058	-	0	0	0
SINE	Alu	B1_Mm	chr6	57516260	57516406	+	0	0	0
SINE	Alu	B1_Mus1	chr6	59708695	59708841	+	0	0	0
Simple_repeat	Simple_repeat	(A)n	chr6	59708842	59708887	+	0	0	0
Simple_repeat	Simple_repeat	(A)n	chr6	60128013	60128050	+	0	0	0

Simple_repeat	Simple_repeat	(A)n	chr6	60201082	60201147	+	0	0	0
SINE	Alu	B1_Mus2	chr10	23489873	23490019	-	0	0	0
Simple_repeat	Simple_repeat	(A)n	chr6	60943833	60943868	+	0	0	0
SINE	Alu	B1_Mus2	chr6	62423244	62423395	-	0	0	0
Simple_repeat	Simple_repeat	(A)n	chr6	62551059	62551098	+	0	0	0
Simple_repeat	Simple_repeat	(A)n	chr10	23510118	23510150	+	0	0	0
Simple_repeat	Simple_repeat	(A)n	chr6	64681543	64681580	+	0	0	0
LINE	L1	L1Md_T	chr6	65152421	65158900	-	0	0	0
SINE	Alu	B1_Mm	chr6	65158929	65159064	+	0	0	0
Simple_repeat	Simple_repeat	(A)n	chr6	65159065	65159093	+	0	0	0
Simple_repeat	Simple_repeat	(A)n	chr6	65253368	65253400	+	0	0	0
Simple_repeat	Simple_repeat	(A)n	chr6	65254918	65254956	+	0.332194	0	0
LINE	L1	L1VL4	chr6	66078485	66083685	-	0	0	0
LINE	L1	L1VL4	chr6	66191313	66194086	+	0	0	0
LINE	L1	L1Md_T	chr6	66408595	66414529	-	0	0	0
SINE	Alu	B1_Mus1	chr6	67209230	67209383	+	0	0	0
Simple_repeat	Simple_repeat	(A)n	chr6	67209384	67209421	+	0	0	0
Simple_repeat	Simple_repeat	(A)n	chr6	67451058	67451096	+	0	0	0
Simple_repeat	Simple_repeat	(A)n	chr6	67487505	67487544	+	0	0	0
LINE	L1	L1Md_T	chr6	67526312	67533627	-	0	0	0
LINE	L1	L1Md_F3	chr6	67544935	67550133	+	0	0	0
SINE	Alu	B1_Mus2	chr6	67581328	67581474	+	0	0	0
LTR	MaLR	MTA_Mm	chr6	67884450	67884845	-	0	0	0
LINE	L1	L1Md_F2	chr6	68223311	68230134	-	0	0	0
Simple_repeat	Simple_repeat	(A)n	chr6	68357727	68357774	+	0	0	0
LINE	L1	L1Md_F2	chr6	68362603	68367970	+	0	0	0
LTR	MaLR	MTA_Mm	chr6	68711205	68711597	+	0	0	0
SINE	Alu	B1_Mus2	chr6	69993251	69993397	+	0	0	0
Simple_repeat	Simple_repeat	(A)n	chr6	69993398	69993438	+	0	0	0
SINE	Alu	B1_Mm	chr6	70244172	70244317	-	0	0	0
Simple_repeat	Simple_repeat	(A)n	chr6	70760433	70760471	+	0	0	0
LTR	MaLR	MTA_Mm	chr10	23550230	23550625	-	0.332194	0	0
LTR	MaLR	MTA_Mm	chr10	23551723	23552118	-	0	0	0
SINE	Alu	B1_Mur2	chr6	70997461	70997603	-	0	0	0
SINE	Alu	B1_Mus2	chr6	71443325	71443470	-	0.591053	0	0
Simple_repeat	Simple_repeat	(A)n	chr6	71780856	71780908	+	0	0	0
SINE	Alu	B1_Mus2	chr6	72401690	72401835	+	0	0	0
Simple_repeat	Simple_repeat	(A)n	chr6	72790988	72791025	+	0	0	0
Simple_repeat	Simple_repeat	(A)n	chr6	74392703	74392737	+	0	0	0
Simple_repeat	Simple_repeat	(A)n	chr6	74636848	74636895	+	0	0	0
SINE	Alu	B1_Mus2	chr6	74716404	74716548	-	0	0	0
Simple_repeat	Simple_repeat	(A)n	chr6	74881963	74882009	+	0	0	0
LINE	L1	L1Md_F2	chr6	75082287	75087307	-	0	0	0
SINE	Alu	B1_Mus1	chr6	75212977	75213122	+	0	0	0
Simple_repeat	Simple_repeat	(A)n	chr6	75213123	75213152	+	0	0	0
SINE	Alu	B1_Mus1	chr6	75538495	75538642	-	0	0	0
Simple_repeat	Simple_repeat	(A)n	chr6	75773684	75773734	+	0	0	0
SINE	Alu	B1_Mus1	chr6	75926108	75926233	-	0	0	0
LTR	MaLR	MTA_Mm	chr6	75965375	75965756	+	0	0	0
SINE	Alu	B1_Mm	chr6	76096400	76096537	-	0	0	0
SINE	Alu	B1_Mm	chr6	76360814	76360958	-	0	0	0
SINE	Alu	B1_Mus1	chr6	77947778	77947902	-	0	0	0
SINE	Alu	B1_Mm	chr6	78036656	78036801	+	0	0.380568	0
Simple_repeat	Simple_repeat	(A)n	chr6	78036802	78036844	+	0	0	0
LTR	MaLR	MTA_Mm	chr10	23627364	23627758	+	0	0	0
LINE	L1	L1Md_T	chr6	78826365	78832760	-	0	0	0
Simple_repeat	Simple_repeat	(A)n	chr6	78991474	78991510	+	0	0	0.342623
Simple_repeat	Simple_repeat	(A)n	chr6	79083948	79083989	+	0	0	0
Simple_repeat	Simple_repeat	(A)n	chr6	79084052	79084088	+	0	0	0
SINE	Alu	B1_Mm	chr6	79523318	79523464	+	0	0	0
SINE	Alu	B1_Mus2	chr6	80167517	80167663	+	0	0	0
Simple_repeat	Simple_repeat	(A)n	chr6	80167664	80167713	+	0	0	0
Simple_repeat	Simple_repeat	(TTTA)n	chr6	80341790	80341820	+	0	0	0
SINE	Alu	B1_Mus2	chr6	81175839	81175984	+	0	0	0
Simple_repeat	Simple_repeat	(A)n	chr6	81175985	81176013	+	0	0	0
LTR	MaLR	MTA_Mm	chr6	81440105	81440498	+	0	0	0
Simple_repeat	Simple_repeat	(A)n	chr6	81705715	81705746	+	0	0	0

Simple_repeat	Simple_repeat	(A)n	chr6	82566330	82566359	+	0	0	0
LTR	MaLR	MTA_Mm	chr6	82614424	82614819	-	0	0	0
SINE	Alu	B1_Mus1	chr6	82618936	82619083	-	0	0	0
Simple_repeat	Simple_repeat	(A)n	chr6	83061853	83061901	+	0.923247	0.380568	0.342623
SINE	Alu	B1_Mm	chr6	83740795	83740941	-	0	0	0
SINE	Alu	B1_Mm	chr6	83754833	83754978	-	0	0	0
SINE	Alu	B1_Mur3	chr6	83802926	83803080	+	0	0	0
Simple_repeat	Simple_repeat	(A)n	chr6	83803081	83803108	+	0	0	0
SINE	Alu	B1_Mm	chr6	83810485	83810631	+	0	0	0
SINE	Alu	B1_Mus1	chr6	85341636	85341782	-	0	0	0
SINE	B2	B2_Mm1t	chr10	23711359	23711550	-	0	0	0
SINE	Alu	B1_Mus2	chr6	86252188	86252340	-	0	0	0
SINE	Alu	B1_Mus1	chr6	86471987	86472113	+	0	0	0
SINE	Alu	B1_Mm	chr6	86780428	86780574	-	0	0	0
Simple_repeat	Simple_repeat	(A)n	chr11	68463611	68463665	+	0	0	0
SINE	Alu	PB1D9	chr6	87892250	87892338	+	0	0	0.957947
Simple_repeat	Simple_repeat	(A)n	chr6	87892339	87892362	+	0	0	0.957947
SINE	Alu	B1_Mus1	chr6	87990743	87990890	+	0	0	0
Simple_repeat	Simple_repeat	(A)n	chr6	87990891	87990917	+	0	0	0
SINE	Alu	B1_Mus2	chr6	88020690	88020835	-	0	0	0
SINE	Alu	B1_Mm	chr6	88061717	88061862	-	0	0	0
SINE	Alu	B1_Mm	chr6	88129619	88129765	+	0	0	0
Simple_repeat	Simple_repeat	(A)n	chr6	88547940	88547974	+	0	0	0
SINE	Alu	B1_Mus2	chr6	89042905	89043037	-	0	0	0
SINE	Alu	B1_Mus1	chr6	89815369	89815515	+	0	0	0
Simple_repeat	Simple_repeat	(A)n	chr6	89815516	89815545	+	0	0	0
LINE	L1	L1Md_T	chr6	89977512	89985872	-	0	0	0
SINE	Alu	B1_Mm	chr6	90135267	90135414	+	0	0	0
SINE	Alu	B1_Mus2	chr6	90466671	90466817	-	0	0	0
SINE	Alu	B1_Mur4	chr6	91255915	91256064	+	0	0	0
Simple_repeat	Simple_repeat	(A)n	chr6	91256065	91256126	+	0	0	0
SINE	Alu	B1_Mur2	chr6	91497608	91497747	-	0	0	0
SINE	B2	B2_Mm1a	chr6	91614161	91614353	+	0	0	0
Simple_repeat	Simple_repeat	(A)n	chr6	91614354	91614376	+	0	0	0
SINE	Alu	B1_Mus1	chr6	92185718	92185855	-	0	0	0
SINE	Alu	B1_Mus2	chr6	92201391	92201537	-	0	0	0
Simple_repeat	Simple_repeat	(TTTA)n	chr10	23804521	23804555	+	0	0	0
Simple_repeat	Simple_repeat	(A)n	chr6	92678055	92678091	+	0	0	0
Simple_repeat	Simple_repeat	(A)n	chr6	92681757	92681811	+	0	0	0
SINE	Alu	B1_Mm	chr6	93186804	93186956	+	0	0.708814	1.23065
Simple_repeat	Simple_repeat	(A)n	chr6	93186957	93186982	+	0	0.708814	1.23065
SINE	B2	B2_Mm1a	chr6	93502380	93502572	+	0	0	0
Simple_repeat	Simple_repeat	(A)n	chr6	93502573	93502604	+	0	0.380568	0.957947
Simple_repeat	Simple_repeat	(A)n	chr11	68892677	68892714	+	0.827951	0	0
SINE	Alu	B1_Mus2	chr6	94945418	94945564	-	0	0	0
SINE	Alu	B1_Mus1	chr11	69109954	69110096	-	0.591053	0.708814	0
Simple_repeat	Simple_repeat	(A)n	chr6	98012029	98012066	+	0	0	0
LTR	MaLR	MTA_Mm	chr6	98175900	98176294	-	0	0	0
LTR	MaLR	MTA_Mm	chr6	98177393	98177787	-	0	0	0
LTR	MaLR	MTA_Mm	chr6	98621425	98621825	-	0	0	0
SINE	Alu	B1_Mm	chr6	98846898	98847044	-	0	0	0
SINE	Alu	B1_Mm	chr6	98858129	98858273	-	0	0	0
SINE	Alu	B1_Mm	chr6	98863383	98863508	-	0	0.380568	0.342623
SINE	B2	B2_Mm1a	chr6	98863539	98863731	+	0	0	0
Simple_repeat	Simple_repeat	(A)n	chr6	98863732	98863756	+	0	0.380568	0.342623
SINE	Alu	B1_Mm	chr6	99422805	99422947	-	1.18211	0.761137	0
SINE	Alu	B1_Mus2	chr6	10030843	10030855	-	0	0	0
Simple_repeat	Simple_repeat	(A)n	chr6	10030918	10030923	+	0.332194	0	0
SINE	Alu	B1_Mm	chr6	10031023	10031035	-	0	0	0
Simple_repeat	Simple_repeat	(A)n	chr6	10037433	10037436	+	0	0	0
Simple_repeat	Simple_repeat	(A)n	chr11	69445885	69445909	+	0	0	0
LTR	MaLR	MTA_Mm	chr6	10278848	10278887	+	0	0	0
SINE	Alu	B1_Mm	chr6	10292689	10292703	+	0	0	0
Simple_repeat	Simple_repeat	(A)n	chr6	10292704	10292706	+	0	0	0
Simple_repeat	Simple_repeat	(A)n	chr6	10302296	10302300	+	0	0	0
LTR	MaLR	MTA_Mm	chr6	10309917	10309955	+	0	0	0
LTR	MaLR	MTA_Mm	chr6	10310064	10310103	+	0	0	0

SINE	Alu	B1_Mus2	chr6	10368402	10368417	-	0	0	0
LINE	L1	L1Md_T	chr6	10420707	10421441	-	0	0	0
Simple_repeat	Simple_repeat	(A)n	chr6	10436064	10436066	+	0	0	0
LINE	L1	L1_Mus2	chr6	10486022	10486490	-	0	0	0
LINE	L1	L1Md_A	chr6	10502225	10502864	-	0	0	0
Simple_repeat	Simple_repeat	(A)n	chr6	10544963	10544967	+	0	0	0
SINE	Alu	B1_Mus1	chr6	10758054	10758069	+	0	0	0
Simple_repeat	Simple_repeat	(A)n	chr6	10758069	10758073	+	0	0	0
LINE	L1	L1Md_T	chr6	10762080	10762768	-	0	0	0
Simple_repeat	Simple_repeat	(A)n	chr6	10792671	10792674	+	0	0	0
Simple_repeat	Simple_repeat	(A)n	chr6	10794434	10794437	+	0	0	0
SINE	Alu	B1_Mus1	chr6	10862940	10862954	+	0	0	0
Simple_repeat	Simple_repeat	(A)n	chr6	10862955	10862957	+	0	0	0
SINE	Alu	B1_Mm	chr6	10934899	10934908	+	0	0	0
Simple_repeat	Simple_repeat	(A)n	chr6	10934908	10934911	+	0	0	0
SINE	Alu	B1_Mm	chr6	10981118	10981133	+	0	0	0
Simple_repeat	Simple_repeat	(A)n	chr6	10981133	10981136	+	0	0	0
SINE	Alu	B1_Mus1	chr6	10989704	10989720	+	0	0	0
Simple_repeat	Simple_repeat	(A)n	chr6	10989720	10989727	+	0	0	0
SINE	Alu	B1_Mur1	chr6	11058576	11058589	-	4.00689	1.41763	3.07662
SINE	Alu	B1_Mm	chr6	11222162	11222177	+	0	0	0
Simple_repeat	Simple_repeat	(A)n	chr6	11222177	11222180	+	0	0	0
LTR	MaLR	MTA_Mm	chr6	11248245	11248284	-	0	0	0
SINE	Alu	B1_Mus2	chr10	24044488	24044632	+	0	0	0
SINE	Alu	B1_Mm	chr6	11297670	11297685	-	0	0	0
Simple_repeat	Simple_repeat	(GAA)n	chr10	24044633	24044730	+	0	0	0.342623
Simple_repeat	Simple_repeat	(A)n	chr6	11315641	11315644	+	0	0	0
Simple_repeat	Simple_repeat	(A)n	chr6	11338872	11338875	+	0	0	0
Simple_repeat	Simple_repeat	(A)n	chr11	70245551	70245585	+	0	0	0
SINE	Alu	B1_Mus2	chr6	11358601	11358616	+	0	0	0
Simple_repeat	Simple_repeat	(A)n	chr6	11358616	11358619	+	0	0	0
SINE	Alu	B1_Mus1	chr6	11366335	11366350	+	0	0	0
Simple_repeat	Simple_repeat	(A)n	chr6	11366350	11366353	+	0	0	0
LTR	ERVL	RLTR28	chr6	11434381	11434435	-	0	0	0
SINE	Alu	B1_Mus2	chr11	70337152	70337298	-	0	0	0
Simple_repeat	Simple_repeat	(TTTA)n	chr6	11499446	11499449	+	0	0	0
Simple_repeat	Simple_repeat	(A)n	chr6	11526309	11526312	+	0	0	0
SINE	Alu	B1_Mus1	chr6	11544149	11544164	-	0	0	0
SINE	Alu	B1_Mm	chr6	11670711	11670724	+	0	0	0
Simple_repeat	Simple_repeat	(A)n	chr6	11670724	11670726	+	0	0	0
SINE	Alu	B1_Mus2	chr6	11678840	11678854	-	0	0	0
Low_complexity	Low_complexity	AT_rich	chr6	11704431	11704435	+	0	0	0
LTR	MaLR	MTA_Mm	chr6	11770437	11770476	-	0	0	0
SINE	Alu	B1_Mus2	chr6	11777948	11777963	-	0	0	0
LTR	MaLR	MTE2b	chr6	11778729	11778760	-	0	0	0
SINE	Alu	B1_Mm	chr11	70566596	70566742	-	0	0	0
LINE	L1	L1Md_T	chr6	11815915	11816580	-	0	0	0
SINE	Alu	B1_Mus2	chr11	70652637	70652782	+	0	0	0
Simple_repeat	Simple_repeat	(A)n	chr11	70652783	70652840	+	0	0	0
SINE	Alu	B1_Mus1	chr6	12053471	12053486	+	0	0	0
SINE	Alu	B1_Mus2	chr6	12053989	12054004	+	0	0	0
Simple_repeat	Simple_repeat	(A)n	chr6	12054004	12054006	+	0	0	0
SINE	Alu	B1_Mus2	chr6	12078380	12078394	+	0	0	0
Simple_repeat	Simple_repeat	(A)n	chr6	12078394	12078397	+	0	0	0
SINE	Alu	B1_Mus1	chr6	12275385	12275397	-	0	0	0
LINE	L1	L1_Mus3	chr6	12385252	12385662	-	0	0	0
SINE	Alu	B1_Mus2	chr6	12434529	12434543	-	0	0	0.615324
LTR	MaLR	MTC	chr6	12443900	12443937	-	0	0	0
SINE	Alu	B1_Mus2	chr6	12453980	12453994	-	0	0	0
SINE	Alu	B1_Mm	chr6	12489692	12489706	+	0	0.380568	0
Simple_repeat	Simple_repeat	(A)n	chr6	12489706	12489708	+	0	0.380568	0
SINE	Alu	B1_Mus2	chr6	12515480	12515495	-	0	0	0
SINE	Alu	B1_Mus1	chr6	12524812	12524826	-	0	0	0
SINE	Alu	B1_Mus1	chr6	12538431	12538444	-	0	0	0
SINE	Alu	B1_Mus2	chr6	12539659	12539673	+	0	0	0
Simple_repeat	Simple_repeat	(A)n	chr6	12539673	12539676	+	0	0	0
SINE	Alu	B1_Mus2	chr6	12692578	12692593	-	0	0.708814	0

SINE	Alu	B1_Mm	chr6	12707422	12707437	+	0	0.708814	0
Simple_repeat	Simple_repeat	(A)n	chr6	12720783	12720785	+	0	0	0
SINE	B2	B3	chr6	12747352	12747373	+	0	0	0
Simple_repeat	Simple_repeat	(A)n	chr6	12747373	12747377	+	0	0	0
SINE	Alu	B1_Mm	chr6	12781264	12781279	+	0	0	0
Simple_repeat	Simple_repeat	(A)n	chr6	12781279	12781281	+	0	0	0
LTR	MaLR	MTA_Mm	chr6	12929249	12929288	+	0	0	0
SINE	Alu	B1_Mur4	chr6	12948073	12948088	+	0	0	0
Low_complexity	Low_complexity	A-rich	chr6	12948088	12948093	+	0	0	0
SINE	Alu	B1_Mus2	chr6	12951819	12951833	+	0	0	0
Simple_repeat	Simple_repeat	(A)n	chr6	12951833	12951836	+	0	0	0
Low_complexity	Low_complexity	A-rich	chr6	12963208	12963217	+	0	0	0
SINE	Alu	B1_Mus2	chr6	13104694	13104709	+	0	0	0
Simple_repeat	Simple_repeat	(A)n	chr6	13144071	13144074	+	0	0	0
Simple_repeat	Simple_repeat	(A)n	chr6	13153949	13153953	+	0	0	0
SINE	Alu	B1_Mus2	chr6	13241693	13241708	-	0	0	0
Simple_repeat	Simple_repeat	(A)n	chr6	13254354	13254357	+	0	0	0
LINE	L1	L1Md_T	chr6	13256515	13257073	-	0	0	0
Simple_repeat	Simple_repeat	(A)n	chr6	13284758	13284761	+	0	0	0
SINE	Alu	B1_Mm	chr6	13324141	13324156	+	0	0	0
Simple_repeat	Simple_repeat	(A)n	chr6	13324156	13324163	+	0	0	0
Simple_repeat	Simple_repeat	(TTTA)n	chr6	13388129	13388134	+	0	0	0
SINE	Alu	B1_Mm	chr6	13474556	13474570	-	0	0	0
Simple_repeat	Simple_repeat	(A)n	chr6	13492149	13492152	+	0	0	0
SINE	Alu	PB1D10	chr6	13521964	13521975	-	0	0	0
SINE	Alu	B1_Mm	chr6	13615474	13615486	+	0	0	0
Simple_repeat	Simple_repeat	(A)n	chr6	13615486	13615489	+	0	0	0
SINE	B2	B2_Mm1a	chr6	13631667	13631686	+	0	0.708814	0
LTR	MaLR	MTA_Mm	chr6	13820846	13820885	-	0	0	0
Simple_repeat	Simple_repeat	(A)n	chr6	13872260	13872263	+	0	0	0
Simple_repeat	Simple_repeat	(A)n	chr6	13905718	13905721	+	0	0	0
SINE	Alu	PB1D7	chr6	14304901	14304913	+	0	0	0
Simple_repeat	Simple_repeat	(A)n	chr6	14304913	14304918	+	0	0	0
SINE	Alu	B1_Mm	chr6	14309042	14309056	+	0	0	0
Simple_repeat	Simple_repeat	(A)n	chr6	14309056	14309059	+	0	0	0
SINE	Alu	B1_Mus2	chr6	14309980	14309995	+	0	0	0
Low_complexity	Low_complexity	A-rich	chr6	14309995	14310003	+	0	0	0
SINE	Alu	B1_Mus1	chr6	14311420	14311435	-	0	0	0
SINE	Alu	B1_Mur3	chr6	14456418	14456433	-	0	0	0
SINE	Alu	B1_Mur4	chr11	72966069	72966205	-	0	0	0
Simple_repeat	Simple_repeat	(A)n	chr6	14564588	14564590	+	0	0	0
LTR	MaLR	MTB	chr6	14594302	14594342	-	0.332194	0	0
SINE	Alu	B1_Mus1	chr6	14663575	14663587	-	0.332194	0	0
SINE	Alu	B1_Mus1	chr11	73141557	73141703	-	0	0	0
LTR	MaLR	MTA_Mm	chr6	14861150	14861189	-	0	0	0
LTR	MaLR	MTA_Mm	chr6	14861298	14861337	-	0	0	0
Simple_repeat	Simple_repeat	(A)n	chr6	14889687	14889691	+	0.591053	0	0
SINE	Alu	B1_Mus1	chr6	14892199	14892214	+	0	0	0
Simple_repeat	Simple_repeat	(A)n	chr6	14892214	14892216	+	0	0	0
SINE	Alu	B1_Mm	chr6	14922941	14922955	-	0	0	0
SINE	B2	B2_Mm1t	chr6	14932651	14932670	+	0	0	0
Simple_repeat	Simple_repeat	(A)n	chr6	14932671	14932673	+	0	0	0
SINE	Alu	B1_Mus1	chr7	3199359	3199500	-	0	0	0
SINE	Alu	B1_Mus2	chr7	3205507	3205653	-	0	0	0
SINE	Alu	B1_Mus2	chr7	3211949	3212068	-	0	0	0
SINE	Alu	B1_Mus1	chr7	3273079	3273220	+	0	0	0
Simple_repeat	Simple_repeat	(A)n	chr7	3273221	3273243	+	0	0	0
LTR	MaLR	MTA_Mm	chr7	3710429	3710825	+	0.332194	0	0
LTR	MaLR	MTA_Mm	chr7	3770490	3770884	+	0	0	0
LINE	L1	L1Md_T	chr7	4137820	4144724	-	0	0	0.342623
LTR	MaLR	MTA_Mm	chr7	4933547	4933886	-	0	0	0
SINE	Alu	B1_Mm	chr7	4971391	4971537	+	0.332194	0	0
Simple_repeat	Simple_repeat	(A)n	chr7	4971538	4971560	+	0.332194	0	0
LINE	L1	L1_Mus3	chr7	5371037	5376288	-	0	0	0
SINE	Alu	B1_Mus2	chr7	6118645	6118791	+	0	0	0.615324
Simple_repeat	Simple_repeat	(A)n	chr7	6296486	6296514	+	0	0	0
SINE	Alu	B1_Mm	chr11	74172177	74172323	-	0	0	0

SINE	Alu	B1_Mm	chr7	7009411	7009543	-	0	0	0
Simple_repeat	Simple_repeat	(A)n	chr7	7458415	7458451	+	0	0	0
Simple_repeat	Simple_repeat	(A)n	chr7	8002903	8002941	+	0	0	0
LINE	L1	L1Md_F2	chr7	9051349	9056456	-	0	0	0
LINE	L1	L1Md_F2	chr7	9342690	9347717	+	0	0	0
LINE	L1	L1Md_F2	chr7	9709170	9714277	-	0	0	0
LTR	MaLR	MTA_Mm-int	chr7	10789210	10790414	+	0	0	0
SINE	Alu	B1_Mus1	chr11	74420400	74420547	+	0	0	0
Simple_repeat	Simple_repeat	(A)n	chr11	74420548	74420569	+	0.332194	0	0
LTR	MaLR	MTA_Mm	chr11	74421947	74422327	-	0	0	0
Simple_repeat	Simple_repeat	(A)n	chr7	11492226	11492278	+	0	0	0
Simple_repeat	Simple_repeat	(A)n	chr7	11897855	11897893	+	0	0	0
SINE	Alu	B1_Mus1	chr7	13505882	13506029	-	0.591053	0	0.615324
SINE	Alu	B1_Mus2	chr7	13537061	13537207	-	0.591053	0	0
SINE	Alu	B1_Mm	chr7	13654283	13654429	-	0	0	0
SINE	Alu	B1_Mur4	chr11	74569355	74569494	-	0	0	0
LINE	L1	L1Md_A	chr7	14001064	14007285	-	0.332194	0	0
SINE	Alu	B1_Mm	chr10	24560456	24560600	-	0	0	0
LTR	MaLR	MTA_Mm	chr7	15221308	15221703	-	0.591053	0	0
LTR	MaLR	MTA_Mm	chr7	15222799	15223194	-	0	0	0
Simple_repeat	Simple_repeat	(A)n	chr7	15550974	15551023	+	0	0	0
Simple_repeat	Simple_repeat	(A)n	chr7	16652918	16652958	+	0	0	0
SINE	Alu	B1_Mur4	chr7	17346033	17346175	+	0	0	0.615324
Simple_repeat	Simple_repeat	(A)n	chr7	17346176	17346205	+	0	0	0
LTR	MaLR	MTB	chr7	17511449	17511846	-	0	0	0
SINE	Alu	B1_Mus2	chr7	17541457	17541580	-	0	0	0
LTR	MaLR	MTA_Mm	chr7	18373381	18373776	+	0	0	0
SINE	Alu	B1_Mm	chr7	19339092	19339247	+	0	0	0
Simple_repeat	Simple_repeat	(TTTA)n	chr7	19500050	19500079	+	0	0	0
SINE	Alu	B1_Mus2	chr7	19833492	19833636	+	1.18211	0	1.23065
SINE	Alu	B1_Mm	chr11	74988037	74988189	+	3.0286	1.08938	0.957947
Simple_repeat	Simple_repeat	(A)n	chr11	74988190	74988240	+	3.28746	0.708814	0.685246
Simple_repeat	Simple_repeat	(TTTA)n	chr7	20522015	20522044	+	0	0	0
LINE	L1	L1Md_F2	chr7	20794688	20799926	+	0	0	0.342623
SINE	Alu	B1_Mm	chr11	75109298	75109449	-	0	0	0
LINE	L1	L1Md_F2	chr7	21267808	21273041	+	0	0	0
LINE	L1	L1Md_F2	chr7	21467477	21472711	+	0	0	0
LINE	L1	L1Md_F2	chr7	21672872	21678098	+	0	0	0
LINE	L1	L1Md_F2	chr7	22310342	22315572	+	0	0	0
LINE	L1	L1Md_F2	chr7	22793149	22798379	+	0	0	0
LINE	L1	L1Md_F2	chr7	22990015	22995258	+	0	0	0
LINE	L1	L1Md_F2	chr7	23456574	23461810	+	0	0	0
LTR	MaLR	MTA_Mm	chr7	24557633	24558027	+	0	0	0
Simple_repeat	Simple_repeat	(A)n	chr7	25680898	25680918	+	0	0	0
SINE	Alu	B1_Mm	chr7	26158697	26158843	+	0	0	0
Simple_repeat	Simple_repeat	(A)n	chr7	26158844	26158877	+	0	0	0
Simple_repeat	Simple_repeat	(A)n	chr7	26402309	26402342	+	0	0	0
SINE	Alu	B1_Mus2	chr7	26462393	26462539	+	0	0	0
Simple_repeat	Simple_repeat	(A)n	chr7	26462540	26462583	+	0	0.708814	0
Simple_repeat	Simple_repeat	(A)n	chr11	75524658	75524687	+	0	0	0
SINE	Alu	B1_Mus2	chr11	75530419	75530547	-	0	0	0
SINE	Alu	B1_Mus2	chr7	27935981	27936138	+	0	0	0
SINE	Alu	B1_Mus2	chr7	28372272	28372409	+	0	0	0
Simple_repeat	Simple_repeat	(A)n	chr7	28372410	28372432	+	0	0	0
SINE	Alu	B1_Mm	chr7	28434546	28434692	-	0	0	0
SINE	Alu	B1_Mus1	chr7	28512030	28512174	+	0	0	0
SINE	Alu	B1_Mus2	chr7	28590471	28590617	-	0	0	0
SINE	Alu	B1_Mus2	chr7	28670184	28670330	-	0	0	0
SINE	Alu	B1_Mus1	chr7	28802318	28802463	-	0	0	0
LTR	MaLR	MTB_Mm	chr7	29060036	29060441	+	0.591053	0.708814	0
SINE	Alu	B1_Mm	chr7	29618160	29618307	-	0	0	0
SINE	Alu	B1_Mus1	chr7	29653004	29653150	+	0	0	0
Simple_repeat	Simple_repeat	(A)n	chr7	29653151	29653199	+	0	0	0
SINE	Alu	B1_Mm	chr7	29665026	29665171	+	0	0	0.342623
Simple_repeat	Simple_repeat	(A)n	chr7	29665172	29665197	+	0	0	0.342623
SINE	Alu	B1_Mus1	chr7	30546327	30546473	+	0	0	0
Simple_repeat	Simple_repeat	(A)n	chr7	30752202	30752227	+	0	0	0

SINE	Alu	B1_Mus2	chr7	31170101	31170247	+	0	0	0
Simple_repeat	Simple_repeat	(A)n	chr7	31170248	31170274	+	0	0	0
SINE	Alu	B1_Mus2	chr7	31594575	31594720	+	0	0	0
Simple_repeat	Simple_repeat	(A)n	chr7	31594721	31594748	+	0	0	0
LTR	MaLR	MTA_Mm	chr7	31780211	31780600	-	0	0	0
LTR	MaLR	MTA_Mm	chr7	32771122	32771514	+	0	0	0
LINE	L1	L1Md_A	chr7	32908219	32914554	-	0	0	0
Simple_repeat	Simple_repeat	(A)n	chr7	33062185	33062237	+	0	0	0
Simple_repeat	Simple_repeat	(A)n	chr7	33089312	33089337	+	0	0	0
LINE	L1	L1_Mus1	chr7	33380908	33387196	-	0	0	0
LTR	MaLR	MTA_Mm	chr7	33503462	33503854	+	0	0	0
LTR	MaLR	MTA_Mm	chr7	33730357	33730749	+	0	0	0
LINE	L1	L1Md_F2	chr7	34321163	34326357	+	0	0	0
LINE	L1	L1Md_T	chr7	34734480	34741392	-	0	0	0
SINE	Alu	B1_Mus2	chr7	34924902	34925048	+	0	0	0
SINE	Alu	B1_Mus1	chr7	35413993	35414138	-	0	0	0
SINE	Alu	B1_Mus1	chr7	35446467	35446621	+	0	0	0
Simple_repeat	Simple_repeat	(A)n	chr7	35446622	35446665	+	0	0	0
SINE	Alu	B1_Mm	chr7	35642870	35643016	-	0	0	0
Simple_repeat	Simple_repeat	(A)n	chr7	35695657	35695691	+	0	0	0
SINE	Alu	B1_Mm	chr7	36015181	36015327	+	0	0	0
Simple_repeat	Simple_repeat	(A)n	chr7	36015328	36015393	+	0	0	0
SINE	B2	B2_Mm1a	chr7	36111336	36111528	-	0	0	0
SINE	Alu	B1_Mus1	chr7	36113264	36113410	-	0	0	0
SINE	Alu	B1_Mus2	chr7	36554612	36554758	+	0	0	0
Simple_repeat	Simple_repeat	(A)n	chr7	36554759	36554782	+	0	0	0
scRNA	scRNA	BC1_Mm	chr7	37472719	37472839	+	0	0	0
SINE	Alu	B1_Mus1	chr7	37630675	37630807	+	0	0	0
Simple_repeat	Simple_repeat	(A)n	chr7	37630808	37630831	+	0	0	0
Simple_repeat	Simple_repeat	(A)n	chr7	37926735	37926773	+	0	0	0
Simple_repeat	Simple_repeat	(A)n	chr7	38109583	38109610	+	0	0	0
Simple_repeat	Simple_repeat	(A)n	chr7	38651313	38651348	+	0	0	0
SINE	Alu	B1_Mm	chr7	39038123	39038269	+	0	0	0
Simple_repeat	Simple_repeat	(A)n	chr7	39038270	39038295	+	0	0	0
Simple_repeat	Simple_repeat	(A)n	chr7	47353807	47353845	+	0	0	0
SINE	Alu	B1_Mus2	chr7	47674143	47674288	+	0	0	0
Low_complexity	Low_complexity	GA-rich	chr7	47674289	47674371	+	0	0	0
Simple_repeat	Simple_repeat	(A)n	chr7	47790412	47790449	+	0	0	0
Simple_repeat	Simple_repeat	(A)n	chr7	48008895	48008933	+	0	0	0
SINE	B2	B3A	chr7	48040351	48040540	+	0	0	0
SINE	B2	B2_Mm1a	chr7	48345754	48345957	-	0	0	0
Simple_repeat	Simple_repeat	(A)n	chr7	48603883	48603917	+	0	0	0
Simple_repeat	Simple_repeat	(A)n	chr7	48609966	48610013	+	0	0	0
Simple_repeat	Simple_repeat	(A)n	chr7	48809135	48809157	+	0	0	0
SINE	Alu	B1_Mm	chr7	48832503	48832649	+	0	0	0
Simple_repeat	Simple_repeat	(A)n	chr7	48832650	48832683	+	0	0	0
LTR	ERVK	RLTR18B	chr7	48877616	48878136	-	0	0	0
LTR	MaLR	MTA_Mm	chr7	48914761	48915138	-	0	0	0
LTR	MaLR	MTA_Mm	chr7	48917451	48917823	-	0	0	0
SINE	Alu	B1_Mm	chr11	76746128	76746274	-	0	0	0
LINE	L1	L1Md_F2	chr7	49483419	49488637	+	0	0	0
SINE	Alu	B1_Mus2	chr7	49733650	49733789	+	0	0	0
Simple_repeat	Simple_repeat	(A)n	chr7	49733790	49733814	+	0	0	0
SINE	Alu	B1_Mm	chr7	49845941	49846086	+	0	0	0
Simple_repeat	Simple_repeat	(A)n	chr7	49846087	49846155	+	0	0	0
SINE	Alu	B1_Mus2	chr7	50266515	50266663	+	0.591053	0	0.342623
Simple_repeat	Simple_repeat	(A)n	chr7	50485898	50485934	+	0	0	0.342623
SINE	Alu	B1_Mm	chr7	50517710	50517855	+	0	0	0
SINE	Alu	B1_Mm	chr7	50530063	50530176	-	0	0	0
SINE	Alu	B1_Mm	chr7	50531852	50531997	-	0	0	0
SINE	Alu	B1_Mm	chr7	50575459	50575604	-	0	0	0
SINE	B2	B2_Mm1t	chr7	50811580	50811772	+	0	0	0
Simple_repeat	Simple_repeat	(A)n	chr7	50811773	50811794	+	0	0.708814	0
SINE	B2	B2_Mm1a	chr7	50811795	50811987	+	0	0.708814	0
Simple_repeat	Simple_repeat	(A)n	chr7	50811988	50812024	+	0	0.708814	0
SINE	Alu	B1_Mus1	chr7	50953689	50953811	+	0	0	0
SINE	Alu	B1_Mus2	chr7	51550181	51550326	+	0	0	0

Simple_repeat	Simple_repeat	(A)n	chr7	51550327	51550354	+	0	0	0.342623
SINE	Alu	B1_Mus2	chr7	51737373	51737519	-	0.591053	0.708814	0.615324
SINE	Alu	B1_Mm	chr7	51935182	51935328	-	0	0	0
Simple_repeat	Simple_repeat	(A)n	chr7	52361560	52361590	+	0	0	0
SINE	Alu	B1_Mm	chr7	52365118	52365243	+	0	0	0
Simple_repeat	Simple_repeat	(GAAA)n	chr7	52365245	52365336	+	0	0	0
SINE	Alu	B1_Mm	chr7	52629898	52630034	-	0.923247	0.708814	0.615324
SINE	Alu	B1_Mus2	chr7	52656209	52656354	-	0	0	0
Simple_repeat	Simple_repeat	(A)n	chr7	52867463	52867504	+	0	0	0.342623
SINE	Alu	B1_Mus2	chr7	53019133	53019277	+	0	0	0
SINE	Alu	B1_Mus2	chr7	53037139	53037287	+	0	0	0
Simple_repeat	Simple_repeat	(A)n	chr7	53037288	53037321	+	0	0	0
SINE	Alu	B1_Mus2	chr7	54084984	54085130	+	0	0	0
Simple_repeat	Simple_repeat	(A)n	chr7	54085131	54085157	+	0	0	0
Simple_repeat	Simple_repeat	(A)n	chr7	54136462	54136498	+	0	0	0
SINE	Alu	B1_Mus1	chr7	55311486	55311629	-	0	0	0
LINE	L1	L1Md_T	chr7	55553241	55560561	-	0	0	0
Simple_repeat	Simple_repeat	(A)n	chr7	55885556	55885591	+	0.591053	0	0
Simple_repeat	Simple_repeat	(A)n	chr7	55972511	55972538	+	0	0	0
SINE	Alu	B1_Mm	chr7	56159738	56159884	+	0	0	0
Simple_repeat	Simple_repeat	(A)n	chr7	56159885	56159947	+	0	0	0
SINE	Alu	B1_Mus2	chr7	56915897	56916026	+	0	0	0
Simple_repeat	Simple_repeat	(A)n	chr7	56916027	56916077	+	0	0	0
Simple_repeat	Simple_repeat	(A)n	chr7	57229915	57229942	+	0.923247	0	0
LTR	MaLR	MTA_Mm	chr7	58256476	58256871	+	0	0	0
Simple_repeat	Simple_repeat	(A)n	chr7	58262586	58262622	+	0	0	0
LTR	MaLR	MTA_Mm	chr7	58350268	58350654	-	0	0	0
SINE	Alu	B1_Mus2	chr7	58415080	58415226	-	0	0	0
SINE	Alu	B1_Mus1	chr11	77435384	77435526	+	0	0	0
Simple_repeat	Simple_repeat	(A)n	chr11	77435527	77435582	+	0	0.380568	0
Simple_repeat	Simple_repeat	(A)n	chr7	58931904	58931937	+	0	0	0
SINE	Alu	B1_Mus1	chr11	77461710	77461847	+	0	0	0
Simple_repeat	Simple_repeat	(A)n	chr11	77461848	77461884	+	0	0	0
Simple_repeat	Simple_repeat	(A)n	chr7	59339564	59339606	+	0	0	0
SINE	Alu	B1_Mm	chr11	77483870	77484015	-	0	0	0
Simple_repeat	Simple_repeat	(A)n	chr7	60213788	60213814	+	0	0	0
SINE	Alu	B1_Mus2	chr7	60379635	60379779	-	0	0	0
LINE	L1	L1Md_F3	chr7	60463731	60469337	-	0	0	0
Simple_repeat	Simple_repeat	(A)n	chr7	61562308	61562356	+	0	0	0
Simple_repeat	Simple_repeat	(A)n	chr7	62786236	62786277	+	0	0	0
SINE	Alu	B1_Mus2	chr11	77692794	77692941	+	0	0	0
Simple_repeat	Simple_repeat	(A)n	chr11	77692942	77692967	+	0	0	0
LINE	L1	L1Md_T	chr7	65490466	65497395	-	0	0	0
SINE	Alu	B1_Mus2	chr7	65537904	65538050	-	0	0	0
SINE	Alu	B1_Mus2	chr7	65722804	65722950	+	0	0	0
LINE	L1	L1Md_F2	chr7	67059151	67063852	+	0	0	0
Simple_repeat	Simple_repeat	(A)n	chr7	67308826	67308859	+	0	0	0
LTR	MaLR	MTA_Mm	chr7	67328544	67328934	-	0	0	0.615324
SINE	Alu	B1_Mus2	chr7	68186190	68186336	+	0	0	0
Simple_repeat	Simple_repeat	(A)n	chr7	68186337	68186426	+	0	0	0
LTR	MaLR	MTA_Mm	chr7	68290388	68290783	+	0	0	0
SINE	Alu	B1_Mm	chr7	68370988	68371116	+	0	0	0
Simple_repeat	Simple_repeat	(A)n	chr7	68371117	68371157	+	0	0	0
Simple_repeat	Simple_repeat	(A)n	chr7	69384160	69384204	+	0	0	0
SINE	Alu	B1_Mus2	chr7	69801203	69801349	+	0	0	0
LTR	MaLR	MTA_Mm	chr7	70403602	70403996	+	0	0	0
Simple_repeat	Simple_repeat	(A)n	chr7	70587937	70587977	+	0	0	0
Simple_repeat	Simple_repeat	(A)n	chr7	71567907	71567949	+	0	0	0
SINE	Alu	B1_Mus1	chr7	72553500	72553646	-	0.591053	0	0
SINE	Alu	B1_Mus2	chr7	72650239	72650384	+	0	0	0
Simple_repeat	Simple_repeat	(A)n	chr7	72650385	72650419	+	0	0	0
Simple_repeat	Simple_repeat	(A)n	chr7	73712584	73712628	+	0.591053	0	0
SINE	Alu	B1_Mus1	chr7	75967124	75967272	-	0	0	0
Simple_repeat	Simple_repeat	(A)n	chr7	76132139	76132183	+	0	0	0
Simple_repeat	Simple_repeat	(A)n	chr7	76563883	76563910	+	0	0	0
Simple_repeat	Simple_repeat	(A)n	chr7	77274209	77274251	+	0	0	0
SINE	Alu	B1_Mm	chr11	78530349	78530495	-	0	0	0

SINE	Alu	B1_Mus2	chr11	78533606 78533752 +	0	0	0
SINE	Alu	B1_Mus1	chr11	78569518 78569664 +	0	0	0
Simple_repeat	Simple_repeat	(A)n	chr11	78569665 78569692 +	0	0	0
Simple_repeat	Simple_repeat	(A)n	chr7	78289934 78289985 +	0	0	0
LTR	MaLR	MTB_Mm	chr11	78590213 78590540 +	0	0	0
SINE	Alu	B1_Mm	chr7	78714528 78714674 +	0	0	0
Simple_repeat	Simple_repeat	(A)n	chr7	78714675 78714704 +	0	0	0
LTR	MaLR	MTA_Mm	chr7	79699433 79699827 +	0	0	0
Simple_repeat	Simple_repeat	(A)n	chr7	79947443 79947482 +	0	0	0
LTR	MaLR	MTA_Mm	chr7	79961838 79962218 -	0	0	0
LTR	MaLR	MTA_Mm	chr7	79963318 79963712 -	0	0	0
Simple_repeat	Simple_repeat	(A)n	chr7	80567428 80567463 +	4.2269	0.380568	0.685246
SINE	Alu	B1_Mus1	chr7	80764656 80764802 +	0	0	0
Simple_repeat	Simple_repeat	(A)n	chr7	80764803 80764843 +	0	0	0
SINE	Alu	B1_Mus1	chr7	82947411 82947556 -	0	0	0
Simple_repeat	Simple_repeat	(A)n	chr7	82949904 82949940 +	0	0	0
LTR	MaLR	MTA_Mm	chr7	82977112 82977507 +	0	0	0
Simple_repeat	Simple_repeat	(A)n	chr7	83034405 83034440 +	0	0	0
LTR	MaLR	MTB	chr7	83311886 83312283 -	0	0	0
Simple_repeat	Simple_repeat	(A)n	chr7	83358255 83358282 +	0	0	0
SINE	Alu	B1_Mus1	chr7	84422635 84422772 -	0	0	0
SINE	Alu	B1_Mus2	chr7	84435361 84435507 +	0	0	0
Simple_repeat	Simple_repeat	(A)n	chr7	84435508 84435567 +	0	0	0
LINE	L1	L1Md_F	chr7	84500001 84505115 -	0	0	0
LINE	L1	L1Md_F2	chr7	84601290 84607812 -	0	0	0
LTR	MaLR	MTA_Mm	chr7	84956283 84956677 +	0	0	0
SINE	Alu	B1_Mus2	chr7	85222544 85222690 -	0	0	0
SINE	Alu	B1_Mus2	chr7	86322761 86322899 +	0	0	0
Simple_repeat	Simple_repeat	(A)n	chr7	86322900 86322931 +	0	0	0
SINE	ID	ID4_	chr7	86765187 86765272 -	0	0	0
Simple_repeat	Simple_repeat	(A)n	chr7	86878177 86878208 +	0	0	0
SINE	Alu	B1_Mus2	chr7	86997322 86997466 +	0	0	0
Simple_repeat	Simple_repeat	(A)n	chr7	86997467 86997502 +	0	0	0
SINE	Alu	B1_Mm	chr7	87126379 87126528 -	0	0	0
Simple_repeat	Simple_repeat	(A)n	chr7	87127533 87127554 +	0	0	0
SINE	Alu	B1_Mm	chr7	87163954 87164100 -	0.591053	0	0
Simple_repeat	Simple_repeat	(A)n	chr7	87958328 87958364 +	0.332194	0	0
SINE	Alu	B1_Mus1	chr7	87990219 87990357 +	0	0	0
Simple_repeat	Simple_repeat	(A)n	chr7	87990363 87990384 +	0	0	0
SINE	Alu	PB1D9	chr7	88193853 88193969 +	0	0	0
Simple_repeat	Simple_repeat	(A)n	chr7	88193970 88194001 +	0	0	0
SINE	Alu	B1_Mus1	chr7	88251184 88251321 -	0	0	0
SINE	Alu	B1_Mus1	chr7	89155465 89155627 +	0	0	0
Simple_repeat	Simple_repeat	(A)n	chr7	89155628 89155652 +	0	0	0
LTR	MaLR	MTA_Mm	chr7	90535309 90535703 +	0	0	0
SINE	Alu	B1_Mm	chr7	91039299 91039444 +	0.332194	0	0
Simple_repeat	Simple_repeat	(A)n	chr7	91039445 91039472 +	0.664388	0	0
SINE	B2	B2_Mm2	chr11	79529388 79529574 -	0	0	0
SINE	Alu	B1_Mus2	chr11	79535240 79535392 +	0.827951	0	0
Simple_repeat	Simple_repeat	(A)n	chr11	79535393 79535416 +	0.827951	0	0
LINE	L1	L1Md_F2	chr7	91640058 91646344 -	0	0	0
SINE	Alu	B1_Mus2	chr7	91948197 91948344 +	0	0	0
Simple_repeat	Simple_repeat	(A)n	chr7	91948345 91948374 +	0	0	0.615324
Simple_repeat	Simple_repeat	(A)n	chr7	91950339 91950372 +	0	0	0
LINE	L1	L1_Mus1	chr7	92472529 92478269 -	0	0	0
LINE	L1	L1_Mm	chr7	92504588 92509196 +	0	0	0
LTR	ERVK	MMERVK10C-int	chr7	92990535 92997858 -	0	0	0
LINE	L1	L1Md_T	chr7	93645972 93652411 -	0	0	0
SINE	Alu	B1_Mus1	chr7	94349957 94350104 +	0	0	0
Simple_repeat	Simple_repeat	(A)n	chr7	94350105 94350128 +	0	0	0
SINE	Alu	B1_Mus2	chr7	94668643 94668789 +	0	0	0
Simple_repeat	Simple_repeat	(A)n	chr7	94668790 94668831 +	0.332194	0.380568	0.342623
SINE	Alu	B1_Mus1	chr7	95801776 95801923 -	0	0	0
SINE	Alu	B1_Mm	chr7	95860639 95860784 +	0	0	0
Simple_repeat	Simple_repeat	(A)n	chr7	95860785 95860826 +	0	0	0
SINE	Alu	B1_Mus2	chr11	79791562 79791708 -	0	0	0
SINE	Alu	B1_Mus1	chr7	96011138 96011285 -	0	0.708814	0

Simple_repeat	Simple_repeat	(A)n	chr7	96120264	96120305	+	0	0	0
SINE	Alu	B1_Mus1	chr11	79800821	79800967	-	0	0	0
Simple_repeat	Simple_repeat	(A)n	chr7	96488438	96488481	+	0	0	0
Simple_repeat	Simple_repeat	(A)n	chr7	96589926	96589973	+	0	0	0
SINE	Alu	B1_Mus1	chr7	97097571	97097707	+	0	0	0
Simple_repeat	Simple_repeat	(A)n	chr7	97097708	97097731	+	0	0	0
SINE	B2	B2_Mm2	chr7	97370204	97370390	-	0	0	0
SINE	Alu	B1_Mm	chr7	97664080	97664231	+	0	0	0
LTR	MaLR	MTA_Mm	chr7	97861306	97861693	+	0	0	0
LTR	MaLR	MTB_Mm	chr7	99682320	99682699	-	0	0	0
SINE	B2	B2_Mm1a	chr11	79961662	79961855	+	0	0	0
Simple_repeat	Simple_repeat	(A)n	chr11	79961856	79961877	+	0	0	0
SINE	Alu	B1_Mm	chr7	99822363	99822508	+	0	0	0
LTR	MaLR	MTA_Mm	chr7	10031210	10031249	+	0	0.380568	0
SINE	Alu	B1_Mus2	chr11	80014448	80014593	-	0	0	0
LTR	MaLR	MTA_Mm	chr7	10083790	10083829	+	0	0	0
SINE	Alu	B1_Mm	chr7	10129095	10129110	-	0	0	0
Simple_repeat	Simple_repeat	(A)n	chr7	10132065	10132070	+	0.591053	0	0
SINE	Alu	B1_Mus2	chr7	10138948	10138962	-	0	0	0
SINE	Alu	B1_Mm	chr7	10145612	10145627	+	0.332194	0	0
Simple_repeat	Simple_repeat	(A)n	chr7	10145627	10145629	+	0	0.380568	0
Simple_repeat	Simple_repeat	(A)n	chr7	10184477	10184480	+	0.332194	0	0
LTR	MaLR	MTA_Mm	chr7	10212013	10212053	-	0	0	0
Simple_repeat	Simple_repeat	(A)n	chr7	10247196	10247200	+	0.591053	0	0
SINE	Alu	B1_Mus1	chr7	10262413	10262427	+	0	0	0
Simple_repeat	Simple_repeat	(A)n	chr7	10262428	10262430	+	0	0	0
SINE	Alu	B1_Mm	chr7	10294261	10294275	-	0.591053	0	0
Simple_repeat	Simple_repeat	(A)n	chr7	10298374	10298379	+	0	0	0
SINE	B4	ID_B1	chr10	25445135	25445348	-	0	0	0
Simple_repeat	Simple_repeat	(A)n	chr7	10322820	10322823	+	0	0	0
SINE	Alu	B1_Mus2	chr11	80231157	80231302	+	3.31962	0	1.57327
Simple_repeat	Simple_repeat	(A)n	chr11	80231303	80231328	+	2.39637	0	0.957947
SINE	Alu	B1_Mus1	chr7	10491230	10491244	+	0	0	0
Simple_repeat	Simple_repeat	(A)n	chr7	10491245	10491250	+	0	0	0
SINE	Alu	B1_Mus1	chr7	10494614	10494626	+	0	0	0.615324
Simple_repeat	Simple_repeat	(A)n	chr7	10494626	10494628	+	0	0	0.615324
LTR	MaLR	MTA_Mm	chr7	10535795	10535835	+	0	0	0
LTR	MaLR	MTA_Mm	chr7	10535945	10535984	+	0	0	0
SINE	Alu	B1_Mus1	chr7	10552352	10552366	+	0	0	0
Simple_repeat	Simple_repeat	(A)n	chr7	10552366	10552368	+	0	0	0
Simple_repeat	Simple_repeat	(A)n	chr7	10581386	10581389	+	0	0	0
Simple_repeat	Simple_repeat	(A)n	chr7	10584102	10584106	+	0	0	0
SINE	Alu	B1_Mm	chr7	10664218	10664233	-	0	0	0
SINE	Alu	B1_Mus1	chr7	10664413	10664428	-	0	0	0
SINE	Alu	B1_Mm	chr7	10664708	10664722	+	0	0	0
Simple_repeat	Simple_repeat	(A)n	chr7	10664722	10664725	+	0	0	0
scRNA	scRNA	4.5SRNA	chr7	10665232	10665242	+	0	0	0
SINE	Alu	B1_Mm	chr7	10665242	10665256	+	0	0	0
Simple_repeat	Simple_repeat	(A)n	chr7	10665457	10665461	+	0	0	0
Simple_repeat	Simple_repeat	(A)n	chr7	10768237	10768240	+	0	0	0
Simple_repeat	Simple_repeat	(TTTA)n	chr7	10783401	10783405	+	0	0	0
SINE	Alu	B1_Mus2	chr7	10785256	10785271	+	0	0	0
Simple_repeat	Simple_repeat	(A)n	chr7	10785271	10785273	+	0	0	0
SINE	Alu	B1_Mus1	chr7	10801448	10801463	-	0	0	0
Simple_repeat	Simple_repeat	(A)n	chr7	10821144	10821147	+	0	0	0
Simple_repeat	Simple_repeat	(A)n	chr7	10903340	10903344	+	0	0	0
Simple_repeat	Simple_repeat	(A)n	chr7	10999554	10999561	+	0	0	0
SINE	Alu	B1_Mus1	chr7	11037810	11037822	-	0	0	0
Simple_repeat	Simple_repeat	(A)n	chr7	11075697	11075702	+	0	0	0
LTR	MaLR	MTA_Mm	chr7	11109880	11109919	+	0	0	0
SINE	Alu	B1_Mus2	chr7	11189768	11189782	-	0	0	0
SINE	Alu	B1_Mus2	chr7	11193028	11193042	+	0	0	0
Simple_repeat	Simple_repeat	(GAAA)n	chr7	11193044	11193053	+	0	0	0
Simple_repeat	Simple_repeat	(A)n	chr7	11238219	11238223	+	0	0	0
LINE	L1	L1_Mus2	chr7	11319969	11320565	+	0	0	0
LINE	L1	L1_Mus2	chr7	11344155	11344752	+	0	0	0
LINE	L1	L1Md_F2	chr7	11350100	11350615	+	0	0	0

SINE	Alu	B1_Mus1	chr7	11383414	11383429	-	0	0	0
SINE	Alu	B1_Mm	chr7	11389750	11389764	+	0	0	0
Simple_repeat	Simple_repeat	(A)n	chr7	11389764	11389771	+	0.332194	0	0
SINE	Alu	B1_Mm	chr7	11393291	11393306	+	0	0	0
Simple_repeat	Simple_repeat	(A)n	chr7	11393306	11393308	+	0	0	0
Simple_repeat	Simple_repeat	(A)n	chr7	11545426	11545431	+	0	0	0
Simple_repeat	Simple_repeat	(A)n	chr7	11636434	11636438	+	0	0	0
SINE	Alu	PB1D10	chr7	11677144	11677155	+	0	0	0
SINE	B4	RSINE1	chr7	11677156	11677171	+	0	0	0
SINE	Alu	B1_Mus2	chr7	11679517	11679531	+	0	0	0
Simple_repeat	Simple_repeat	(TTTA)n	chr7	11977436	11977440	+	0.591053	0	0
SINE	Alu	B1_Mus2	chr7	11981833	11981847	+	0	0	0
Simple_repeat	Simple_repeat	(A)n	chr7	11981847	11981850	+	0	0	0
Simple_repeat	Simple_repeat	(A)n	chr7	12006431	12006434	+	0	0	0
SINE	Alu	B1_Mm	chr7	12045890	12045905	+	0	0	0
Simple_repeat	Simple_repeat	(A)n	chr7	12045905	12045907	+	0	0	0
SINE	B2	B3A	chr7	12126971	12126988	+	0	0	0
SINE	Alu	B1_Mm	chr7	12213935	12213949	-	0	0	0
Simple_repeat	Simple_repeat	(A)n	chr7	12255675	12255678	+	0	0	0
SINE	B2	B2_Mm1t	chr7	12336216	12336235	+	0	0	0
Simple_repeat	Simple_repeat	(A)n	chr7	12336235	12336238	+	0.332194	0	0.685246
Simple_repeat	Simple_repeat	(A)n	chr7	12375033	12375035	+	0	0	0
SINE	Alu	B1_Mm	chr7	12410065	12410079	+	0	0	0
Simple_repeat	Simple_repeat	(A)n	chr7	12410079	12410084	+	0	0	0
SINE	Alu	B1_Mm	chr7	12421433	12421448	-	0	0	0
LTR	MaLR	MTA_Mm	chr11	82282149	82282543	-	0	0	0
LTR	MaLR	MTE2b	chr7	12493021	12493055	+	0	0	0
SINE	Alu	B1_Mm	chr7	12517368	12517383	+	0	0	0
LTR	MaLR	MTD	chr7	12517383	12517419	+	0	0	0
Simple_repeat	Simple_repeat	(A)n	chr7	12542299	12542301	+	0	0	0
SINE	Alu	B1_Mm	chr11	82515748	82515902	+	0	0	0
Simple_repeat	Simple_repeat	(A)n	chr11	82515903	82515933	+	0	0	0
Simple_repeat	Simple_repeat	(A)n	chr7	12707879	12707883	+	0	0	0
SINE	Alu	B1_Mm	chr7	12712337	12712351	+	0	0	0
Simple_repeat	Simple_repeat	(A)n	chr7	12712351	12712354	+	0	0	0
Simple_repeat	Simple_repeat	(A)n	chr7	12726911	12726915	+	0	0	0
Simple_repeat	Simple_repeat	(A)n	chr7	12769478	12769481	+	0	0	0
LTR	MaLR	MTA_Mm	chr7	12867178	12867218	-	0	0	0
SINE	Alu	B1_Mus1	chr7	12896657	12896672	+	0	0	0
SINE	Alu	B1_Mus2	chr7	12897278	12897293	-	0	0	0
LTR	MaLR	MTB	chr11	82858321	82858724	-	0	0	0
SINE	Alu	B1_Mus2	chr7	12992084	12992098	+	0	0	0
Simple_repeat	Simple_repeat	(A)n	chr7	12992098	12992104	+	0.332194	0	0
SINE	Alu	B1_Mus1	chr7	13019661	13019675	+	0	0	0
Simple_repeat	Simple_repeat	(A)n	chr7	13019677	13019683	+	0	0	0
Simple_repeat	Simple_repeat	(A)n	chr7	13254096	13254098	+	0	0	0
SINE	Alu	B1_Mm	chr7	13347232	13347246	+	0.332194	0	0
Simple_repeat	Simple_repeat	(A)n	chr7	13347246	13347251	+	0.332194	0	0
SINE	Alu	B1_Mus1	chr7	13349114	13349129	-	0	0	0
SINE	Alu	B1_Mm	chr7	13354631	13354646	-	0	0	0
Simple_repeat	Simple_repeat	(A)n	chr7	13354963	13354966	+	0	0.380568	0
Simple_repeat	Simple_repeat	(A)n	chr7	13368111	13368114	+	0	0	0
SINE	B2	B2_Mm1a	chr7	13377386	13377405	+	0	0	0
SINE	Alu	B1_Mus1	chr7	13405601	13405616	+	0	0	0
Simple_repeat	Simple_repeat	(A)n	chr7	13405616	13405620	+	0	0	0
SINE	Alu	PB1D9	chr7	13420134	13420146	-	0	0	0
SINE	Alu	B1_Mus1	chr7	13421143	13421157	-	0	0	0
Simple_repeat	Simple_repeat	(A)n	chr7	13428903	13428908	+	0	0	0
LTR	MaLR	MTA_Mm	chr7	13451271	13451311	+	0	0	0
LTR	MaLR	MTA_Mm	chr7	13451407	13451447	+	0	0	0.342623
SINE	Alu	B1_Mm	chr7	13456061	13456075	+	0	0	0
SINE	Alu	B1_Mus1	chr7	13464077	13464089	+	0	0	0
Simple_repeat	Simple_repeat	(A)n	chr7	13464089	13464091	+	0	0	0
Simple_repeat	Simple_repeat	(TTTA)n	chr7	13467081	13467085	+	0.591053	0.708814	0
SINE	Alu	B1_Mus2	chr7	13479306	13479322	-	0	0	1.23065
Simple_repeat	Simple_repeat	(A)n	chr7	13487171	13487176	+	0.332194	0	0
Simple_repeat	Simple_repeat	(TTTA)n	chr7	13488398	13488400	+	0	0	0

SINE	B2	B3A	chr7	13513258	13513277	-	0	0	0
SINE	Alu	B1_Mus1	chr7	13546532	13546547	+	0	0	0
Simple_repeat	Simple_repeat	(A)n	chr7	13546547	13546549	+	0	0	0
Simple_repeat	Simple_repeat	(A)n	chr7	13548458	13548461	+	0	0	1.23065
SINE	Alu	B1_Mus2	chr7	13563330	13563345	+	0	0	0.342623
SINE	Alu	B1_Mus1	chr7	13570244	13570258	-	0	0	0
Simple_repeat	Simple_repeat	(A)n	chr10	25897460	25897506	+	0	0	0
SINE	B2	B2_Mm1a	chr7	13753343	13753364	+	0	0	0
Simple_repeat	Simple_repeat	(A)n	chr7	13753364	13753370	+	0.664388	0	0
Simple_repeat	Simple_repeat	(A)n	chr7	13865643	13865646	+	0	0	0
SINE	Alu	B1_Mm	chr11	83891508	83891654	+	0	0	0
Simple_repeat	Simple_repeat	(A)n	chr11	83891655	83891700	+	0	0	0
SINE	Alu	B1_Mus1	chr7	14013711	14013726	+	4.74462	2.50701	3.29367
Simple_repeat	Simple_repeat	(A)n	chr7	14013726	14013729	+	1.91983	2.50701	3.02097
SINE	Alu	B1_Mm	chr11	83961408	83961553	+	0	0	0.957947
Simple_repeat	Simple_repeat	(A)n	chr11	83961554	83961588	+	0	0	0.957947
SINE	Alu	B1_Mur2	chr11	83984105	83984248	-	0	0	0
Simple_repeat	Simple_repeat	(A)n	chr7	14178322	14178325	+	0	0	0
SINE	Alu	B1_Mus2	chr7	14185174	14185189	+	0	0	0
Low_complexity	Low_complexity	A-rich	chr7	14297447	14297456	+	0	0	0
SINE	Alu	B1_Mur1	chr7	14345894	14345909	+	0	0	0
Simple_repeat	Simple_repeat	(A)n	chr7	14345909	14345913	+	0	0	0
Simple_repeat	Simple_repeat	(A)n	chr7	14359849	14359853	+	0	0	0
Simple_repeat	Simple_repeat	(A)n	chr7	14399906	14399910	+	0	0	0.342623
SINE	Alu	B1_Mus2	chr7	14518143	14518158	+	0	0	0
Simple_repeat	Simple_repeat	(A)n	chr7	14518158	14518162	+	0	0	0
SINE	Alu	B1_Mm	chr7	14610962	14610976	+	0	0	0
Simple_repeat	Simple_repeat	(A)n	chr7	14683406	14683409	+	0	0	0
LTR	MaLR	MTA_Mm	chr7	14695618	14695658	+	0	0	0
Simple_repeat	Simple_repeat	(A)n	chr7	14707763	14707766	+	0	0	0
Simple_repeat	Simple_repeat	(A)n	chr7	14727285	14727289	+	0	0	0
Simple_repeat	Simple_repeat	(A)n	chr7	14798175	14798178	+	0	0	0
SINE	Alu	B1_Mus1	chr7	14819275	14819288	+	0	0	0
Simple_repeat	Simple_repeat	(A)n	chr7	14819288	14819292	+	0	0	0
SINE	Alu	B1_Mus1	chr7	14836667	14836681	-	0	0	0
SINE	B2	B2_Mm1t	chr7	14884514	14884533	-	0	0	0.342623
SINE	Alu	B1_Mus1	chr7	14979876	14979890	+	0	0	0
Simple_repeat	Simple_repeat	(A)n	chr7	14979890	14979894	+	0	0	0
Simple_repeat	Simple_repeat	(A)n	chr7	14985665	14985673	+	0	0	0
Simple_repeat	Simple_repeat	(A)n	chr7	15069511	15069513	+	0	0	0
SINE	Alu	B1_Mm	chr7	15078810	15078824	+	0	0	0
SINE	Alu	B1_Mm	chr7	15161772	15161786	-	0	0	0
SINE	Alu	B1_Mm	chr11	84689127	84689260	+	0	0	0
Simple_repeat	Simple_repeat	(A)n	chr11	84689261	84689308	+	0	0	0
Low_complexity	Low_complexity	GA-rich	chr7	15201787	15201799	+	0	0	0
SINE	Alu	B1_Mus2	chr11	84769140	84769286	+	0	0	0
Simple_repeat	Simple_repeat	(A)n	chr11	84769287	84769309	+	0	0.380568	0
SINE	Alu	B1_Mus1	chr8	3560946	3561092	-	0	0	0
LTR	MaLR	MTA_Mm	chr8	3861211	3861583	+	0	0	0
SINE	Alu	B1_Mm	chr8	4133219	4133360	-	0	0	0
LTR	MaLR	MTA_Mm	chr8	4422710	4423104	-	0	0	0
Simple_repeat	Simple_repeat	(A)n	chr8	5119157	5119187	+	0	0	0.342623
SINE	Alu	B1_Mus1	chr8	5637088	5637232	+	0	0	0
Low_complexity	Low_complexity	GA-rich	chr8	5637233	5637368	+	0	0	0
SINE	Alu	B1_Mm	chr8	5885533	5885679	-	0	0	0
SINE	Alu	B1_Mus2	chr8	6169310	6169455	+	0	0	0
Simple_repeat	Simple_repeat	(A)n	chr8	6169456	6169475	+	0	0	0
LTR	MaLR	MTA_Mm	chr8	6259817	6260202	-	0	0.708814	0
Simple_repeat	Simple_repeat	(A)n	chr8	6297219	6297259	+	0	0	0
SINE	Alu	B1_Mus1	chr8	6436951	6437104	+	0	0	0
Simple_repeat	Simple_repeat	(A)n	chr8	6460216	6460253	+	0	0	0
Simple_repeat	Simple_repeat	(A)n	chr8	6644418	6644457	+	0	0	0
Simple_repeat	Simple_repeat	(A)n	chr8	6857748	6857768	+	0	0	0
LTR	MaLR	MTB	chr8	7001471	7001867	-	0	0	0
Simple_repeat	Simple_repeat	(A)n	chr8	7135552	7135577	+	0	0	0
Simple_repeat	Simple_repeat	(A)n	chr8	7492555	7492591	+	0	0	0
SINE	Alu	B1_Mus1	chr11	85055835	85055982	-	0	0	0

Simple_repeat	Simple_repeat	(A)n	chr8	7914883	7914945	+	0	0	0
LINE	L1	L1Md_F2	chr8	8066791	8073201	-	0	0	0.615324
Simple_repeat	Simple_repeat	(A)n	chr8	8314801	8314850	+	0	0	0
SINE	Alu	B1_Mus2	chr8	8614893	8615039	+	0	0	0
Simple_repeat	Simple_repeat	(A)n	chr8	8615040	8615076	+	0	0	0
SINE	Alu	B1_Mus2	chr8	8701644	8701790	-	0	0	0
Simple_repeat	Simple_repeat	(TTTA)n	chr8	8723275	8723297	+	0	0	0
LTR	MaLR	MTA_Mm	chr8	8816865	8817255	-	0	0	0
Simple_repeat	Simple_repeat	(A)n	chr8	8826065	8826112	+	0	0	0
SINE	Alu	B1_Mus2	chr11	85115720	85115860	-	0	0	0
SINE	Alu	B1_Mus2	chr8	9778463	9778609	+	0	0	0
Simple_repeat	Simple_repeat	(A)n	chr8	9778610	9778636	+	0	0	0
SINE	Alu	B1_Mm	chr11	85161428	85161569	-	0	0	0
LTR	MaLR	MTB	chr8	10799864	10800255	-	0	0	0
SINE	Alu	B1_Mus2	chr8	10969701	10969847	-	0	0	0
SINE	Alu	B1_Mm	chr8	11495289	11495433	-	0	0	0
SINE	Alu	B1_Mus2	chr8	12266863	12266998	+	0	0	0
Simple_repeat	Simple_repeat	(A)n	chr8	12495752	12495797	+	0	0	0
Simple_repeat	Simple_repeat	(A)n	chr8	12673499	12673532	+	0	0	0.615324
SINE	Alu	B1_Mus1	chr8	13690256	13690401	+	0	0	0
Low_complexity	Low_complexity	A-rich	chr8	13690402	13690472	+	0	0	0
SINE	Alu	B1_Mus2	chr8	13855473	13855619	+	0	0	0
Simple_repeat	Simple_repeat	(A)n	chr8	13855620	13855655	+	0	0	0
LTR	MaLR	MTA_Mm	chr8	15176455	15176846	+	0	0.708814	0
LTR	MaLR	MTA_Mm	chr8	15198515	15198910	+	0	0	0
Simple_repeat	Simple_repeat	(A)n	chr8	15449660	15449687	+	0	0	0
Simple_repeat	Simple_repeat	(A)n	chr8	15557450	15557479	+	0	0	0
SINE	ID	ID2	chr8	17560192	17560276	-	0	0	0
LTR	MaLR	MTA_Mm	chr8	17630582	17630961	+	0	0	0
SINE	Alu	B1_Mus2	chr8	18138356	18138493	+	0	0	0
Simple_repeat	Simple_repeat	(A)n	chr8	18326938	18326974	+	0	0	0
Simple_repeat	Simple_repeat	(A)n	chr8	18894133	18894175	+	0	0	0
LTR	MaLR	MTA_Mm	chr8	19539381	19539776	+	0	0	0.342623
SINE	Alu	B1_Mus1	chr8	22942384	22942530	-	0	0	0
SINE	Alu	B1_Mm	chr8	23025264	23025409	-	0	0	0
SINE	Alu	B1_Mus2	chr8	23301528	23301674	+	0	0	0
LTR	MaLR	MTA_Mm	chr8	25438769	25439166	+	0	0	0
Simple_repeat	Simple_repeat	(A)n	chr8	25493432	25493472	+	0	0	0
LTR	MaLR	MTA_Mm	chr8	25518738	25519129	+	0	0	0
SINE	Alu	B1_Mur4	chr8	25556609	25556730	-	0	0	0
LTR	MaLR	MTB	chr8	25611755	25612148	-	0	0	0
Simple_repeat	Simple_repeat	(A)n	chr8	26574112	26574140	+	0	0	0
Simple_repeat	Simple_repeat	(A)n	chr8	26581100	26581128	+	0	0	0
LTR	MaLR	MTA_Mm	chr8	27053880	27054275	+	0	0	0
SINE	Alu	B1_Mus1	chr11	86256819	86256954	-	0	0	0
SINE	Alu	B1_Mus2	chr8	27735629	27735779	+	0	0	0
Simple_repeat	Simple_repeat	(A)n	chr8	27735780	27735811	+	0	0	0
LTR	MaLR	MTA_Mm	chr8	27898119	27898511	-	0	0	0
Low_complexity	Low_complexity	AT_rich	chr8	28320437	28320468	+	0	0	0
LTR	MaLR	MTA_Mm	chr8	28355228	28355622	+	0	0	0
SINE	Alu	B1_Mus2	chr8	28636372	28636521	+	0	0	0
Simple_repeat	Simple_repeat	(A)n	chr8	29265964	29266013	+	0	0	0
LTR	MaLR	MTA_Mm	chr8	29659436	29659815	-	0	0	0
Simple_repeat	Simple_repeat	(A)n	chr8	30607396	30607432	+	0	0	0
SINE	Alu	B1_Mus1	chr8	30981921	30982041	-	0	0	0
LTR	MaLR	MTA_Mm	chr8	30988457	30988850	+	0	0	0
SINE	Alu	B1_Mm	chr8	31064433	31064579	+	0	0	0
Simple_repeat	Simple_repeat	(A)n	chr8	31522711	31522739	+	0	0	0
LTR	MaLR	MTA_Mm	chr8	31885947	31886327	+	0.591053	0	0
LTR	MaLR	MTA_Mm	chr8	32059179	32059548	+	0	0	0
LTR	MaLR	MTA_Mm	chr8	32095674	32096065	+	0	0	0
Simple_repeat	Simple_repeat	(A)n	chr8	33115317	33115358	+	0	0	0
LTR	MaLR	MTA_Mm	chr8	33543311	33543698	+	0.591053	0	0
Simple_repeat	Simple_repeat	(A)n	chr8	34090225	34090256	+	0	0	0
SINE	Alu	B1_Mus1	chr8	34268624	34268770	+	0	0	0
SINE	Alu	B1_Mus1	chr8	34332837	34332964	+	0	0	0
Simple_repeat	Simple_repeat	(A)n	chr8	34332965	34332985	+	0	0	0

SINE	Alu	B1_Mus2	chr8	34560826	34560972	+	0	0	0
Simple_repeat	Simple_repeat	(A)n	chr8	34560973	34561017	+	0	0	0
Simple_repeat	Simple_repeat	(A)n	chr8	35043079	35043115	+	0	0	0
SINE	Alu	B1_Mus2	chr8	35109423	35109569	-	0	0	0
SINE	Alu	B1_Mus1	chr8	35214861	35215010	-	0	0	0
Simple_repeat	Simple_repeat	(A)n	chr8	35422964	35422993	+	0	0	0
SINE	Alu	B1_Mus2	chr8	36130404	36130552	-	0	0	0
Simple_repeat	Simple_repeat	(A)n	chr8	36271709	36271760	+	0	0	0
Simple_repeat	Simple_repeat	(A)n	chr8	37103832	37103873	+	0	0	0
Simple_repeat	Simple_repeat	(A)n	chr8	39751601	39751633	+	0	0	0
SINE	Alu	B1_Mm	chr8	39872774	39872920	+	0	0	0
Simple_repeat	Simple_repeat	(A)n	chr8	39872921	39872944	+	0	0	0
LTR	MaLR	MTA_Mm	chr8	40476505	40476894	+	0	0	0
Simple_repeat	Simple_repeat	(A)n	chr8	41867776	41867810	+	0	0	0
LTR	MaLR	MTA_Mm	chr8	42271490	42271877	-	0	0	0
SINE	Alu	B1_Mus1	chr8	42543775	42543923	+	0	0	0
Low_complexity	Low_complexity	A-rich	chr8	42543935	42543967	+	0	0	0
LINE	L1	L1Md_F2	chr8	42587006	42592293	-	0	0	0
LTR	MaLR	MTA_Mm	chr8	43716956	43717349	-	0	0	0
LTR	MaLR	MTA_Mm	chr8	43718429	43718822	-	0	0	0
SINE	Alu	B1_Mm	chr8	44287862	44288006	+	0	0	0
Simple_repeat	Simple_repeat	(A)n	chr8	44288007	44288032	+	0	0	0
SINE	Alu	B1_Mm	chr8	44426680	44426826	+	0	0	0
Simple_repeat	Simple_repeat	(A)n	chr8	44426827	44426854	+	0	0	0
SINE	Alu	B1_Mus1	chr8	44823905	44824052	+	0	0	0
Simple_repeat	Simple_repeat	(A)n	chr8	44824053	44824078	+	0	0	0
SINE	Alu	B1_Mus2	chr8	44876437	44876583	+	0	0	0
Simple_repeat	Simple_repeat	(A)n	chr8	44876584	44876717	+	0	0	0
LTR	MaLR	MTA_Mm	chr8	45089812	45090186	+	0	0	0
LINE	L1	L1Md_F2	chr8	45379230	45385341	-	0	0	0
Simple_repeat	Simple_repeat	(A)n	chr8	45536722	45536766	+	0	0	0
Simple_repeat	Simple_repeat	(A)n	chr8	46549539	46549565	+	0	0	0
SINE	Alu	B1_Mm	chr8	48251306	48251452	+	0	0	0
Simple_repeat	Simple_repeat	(A)n	chr8	48251453	48251483	+	0	0	0
SINE	Alu	B1_Mus1	chr8	48543668	48543814	-	0	0	0
SINE	Alu	B1_Mus1	chr8	48648542	48648688	-	0	0	0
SINE	Alu	B1_Mus1	chr8	48726136	48726253	+	0	0	0
SINE	Alu	B1_Mus2	chr8	48783816	48783960	-	0	0	0
SINE	Alu	B1_Mus1	chr8	49100980	49101115	+	0	0	0
Simple_repeat	Simple_repeat	(A)n	chr8	49101116	49101138	+	0	0.380568	0
LTR	MaLR	MTB	chr8	49241649	49242047	-	0	0.708814	0.615324
Simple_repeat	Simple_repeat	(A)n	chr8	49796393	49796431	+	0	0	0
LTR	ERVK	RMER6D	chr8	50515401	50516187	+	0	0	0
Simple_repeat	Simple_repeat	(A)n	chr8	50926890	50926918	+	0	0	0
LTR	MaLR	MTA_Mm	chr8	51359765	51360156	+	0	0	0
Simple_repeat	Simple_repeat	(A)n	chr8	51412196	51412222	+	0	0	0
LTR	MaLR	MTA_Mm	chr8	51443621	51444013	+	0	0	0
SINE	Alu	B1_Mm	chr8	51512988	51513133	+	0	0	0
Simple_repeat	Simple_repeat	(A)n	chr8	51513134	51513166	+	0	0	0
SINE	Alu	B1_Mus2	chr8	51824363	51824509	-	1.18211	0	0.615324
LINE	L1	L1Md_F2	chr8	52060475	52065552	+	0	0	1.10507
LTR	MaLR	ORR1D1-int	chr8	52429191	52430442	-	0	0	0
Simple_repeat	Simple_repeat	(A)n	chr8	53006005	53006047	+	0	0	0
Simple_repeat	Simple_repeat	(A)n	chr8	53046760	53046803	+	0	0	0
SINE	Alu	B1_Mus1	chr8	53464191	53464337	-	0	0	0
Simple_repeat	Simple_repeat	(A)n	chr8	53900981	53901037	+	0	0	0
Simple_repeat	Simple_repeat	(A)n	chr8	54506778	54506817	+	0	0.380568	0.957947
Simple_repeat	Simple_repeat	(GAAAA)n	chr8	54520446	54520619	+	0	0	0
Low_complexity	Low_complexity	A-rich	chr8	54563175	54563287	+	0	0	0
Simple_repeat	Simple_repeat	(A)n	chr8	55016041	55016077	+	0	0	0
Simple_repeat	Simple_repeat	(A)n	chr8	55091961	55091988	+	0	0	0
SINE	Alu	B1_Mm	chr8	55277062	55277207	-	0	0	0
Simple_repeat	Simple_repeat	(A)n	chr8	58922436	58922482	+	0	0	0
SINE	Alu	B1_Mus1	chr8	59104641	59104785	+	0	0	0
Simple_repeat	Simple_repeat	(A)n	chr8	59104786	59104809	+	0	0	0
SINE	Alu	B1_Mus2	chr11	88055784	88055917	+	0	0	0
Simple_repeat	Simple_repeat	(A)n	chr11	88055918	88055941	+	0	0	0

LTR	MaLR	MTA_Mm	chr8	59524362	59524757	-	0	0	0.342623
LTR	MaLR	MTA_Mm	chr8	59756679	59757060	+	0	0	0
SINE	Alu	B1_Mus2	chr8	59882899	59883024	+	0	0	0
Simple_repeat	Simple_repeat	(A)n	chr8	59883025	59883074	+	0	0	0
SINE	Alu	B1_Mur2	chr8	59926214	59926359	+	0	0	0
Low_complexity	Low_complexity	A-rich	chr8	59926362	59926426	+	0	0	0
Simple_repeat	Simple_repeat	(A)n	chr8	61420247	61420328	+	0.591053	0	0
SINE	B4	ID_B1	chr8	61585647	61585860	+	0	0	0
Simple_repeat	Simple_repeat	(A)n	chr8	61585861	61585885	+	0	0	0
Simple_repeat	Simple_repeat	(A)n	chr10	26491840	26491882	+	0	0	0
Simple_repeat	Simple_repeat	(A)n	chr8	62066868	62066902	+	0	0	0
LINE	L1	L1Md_F2	chr8	62339122	62344820	+	0	0	0
Simple_repeat	Simple_repeat	(A)n	chr8	63172433	63172474	+	0	0	0
LTR	MaLR	MTA_Mm	chr8	63186018	63186413	-	0	0	0
SINE	Alu	B1_Mus1	chr8	63288522	63288668	+	0	0	0
Simple_repeat	Simple_repeat	(A)n	chr8	63288669	63288707	+	0	0	0
SINE	Alu	B1_Mm	chr8	63359483	63359628	-	0	0	0
SINE	Alu	B1_Mm	chr8	63683984	63684115	+	0	0	0
Simple_repeat	Simple_repeat	(GAAAA)n	chr8	63684116	63684183	+	0	0	0
LTR	MaLR	MTB	chr8	63934211	63934606	-	0	0	0
LINE	L1	L1Md_F2	chr8	64649394	64655284	-	0	0	0.342623
SINE	Alu	B1_Mus2	chr8	64899392	64899540	-	0	0	0
SINE	Alu	B1_Mm	chr11	88630165	88630311	-	0	0	0
SINE	Alu	B1_Mus1	chr8	65331953	65332099	+	0	0	0
LTR	MaLR	MTA_Mm	chr8	65409137	65409532	+	0	0	0
LTR	MaLR	MTA_Mm	chr8	65943005	65943398	+	0	0	0
LINE	L1	L1_Mus3	chr8	66079146	66085069	-	0	0	0
Simple_repeat	Simple_repeat	(A)n	chr8	66093597	66093623	+	0	0	0
SINE	Alu	B1_Mm	chr11	88739080	88739225	+	0	0	0.342623
Simple_repeat	Simple_repeat	(A)n	chr11	88739226	88739256	+	0	0	0.342623
SINE	Alu	B1_Mus2	chr8	67423729	67423876	+	0	0	0
Simple_repeat	Simple_repeat	(A)n	chr8	67423877	67423933	+	0	0	0
LINE	L1	L1Md_F2	chr8	67755269	67761636	-	0	0	0.615324
LINE	L1	L1Md_F2	chr8	69045686	69050636	+	0	0	0
Simple_repeat	Simple_repeat	(A)n	chr8	69131143	69131177	+	0	0	0
SINE	B2	B2_Mm1t	chr8	69237567	69237757	+	0	0	0
SINE	Alu	B1_Mus2	chr8	69586444	69586590	+	0	0	0
Simple_repeat	Simple_repeat	(A)n	chr8	69586591	69586615	+	0	0	0
Simple_repeat	Simple_repeat	(A)n	chr8	69889516	69889537	+	0	0	0
SINE	Alu	B1_Mus2	chr8	70456423	70456570	+	0	0	0
SINE	Alu	B1_Mus1	chr8	70875587	70875734	-	0.591053	0	0.342623
SINE	Alu	B1_Mus2	chr8	71387446	71387591	-	0	0	0
SINE	Alu	B1_Mus1	chr8	71553053	71553199	+	0	0	0
Simple_repeat	Simple_repeat	(A)n	chr8	71553200	71553255	+	0	0	0
LTR	MaLR	MTA_Mm	chr8	72209932	72210329	+	0	0	0
SINE	B2	B2_Mm1t	chr8	72212760	72212944	-	0	0	0
SINE	Alu	B1_Mm	chr11	89021077	89021222	+	0	0	0
Simple_repeat	Simple_repeat	(A)n	chr11	89021223	89021244	+	0	0	0
SINE	Alu	B1_Mus1	chr8	73391442	73391589	-	0	0	0
SINE	Alu	B1_Mus2	chr8	73405805	73405951	+	0	0	0
SINE	Alu	B1_Mm	chr8	73763998	73764144	+	0	0	0
Simple_repeat	Simple_repeat	(A)n	chr8	73764145	73764174	+	0	0	0
SINE	B2	B2_Mm2	chr8	74017526	74017714	-	0	0	0
SINE	Alu	B1_Mus1	chr8	74056019	74056158	-	0	0	0
LTR	MaLR	MTA_Mm	chr8	74444007	74444401	-	0	0	0
SINE	Alu	B1_Mm	chr8	74628346	74628495	+	0	0	0
Simple_repeat	Simple_repeat	(A)n	chr8	74628496	74628547	+	0	0.380568	0
SINE	Alu	B1_Mm	chr8	74710869	74711016	-	0	0	0
LINE	L1	L1Md_F2	chr8	76029152	76035614	-	0	0	0
LTR	MaLR	MTA_Mm	chr8	76367664	76368059	+	0	0	0
LINE	L1	Lx	chr8	76683556	76687980	-	0	0	0
LINE	L1	L1Md_F2	chr8	77033597	77039284	-	0.332194	0	0.342623
SINE	Alu	B1_Mm	chr8	77730886	77731031	-	0	0	0
Simple_repeat	Simple_repeat	(A)n	chr8	77759633	77759659	+	0	0	0
LTR	MaLR	MTA_Mm	chr11	89619604	89619999	+	0	0	0
LINE	L1	L1Md_F3	chr8	78687786	78693976	-	0	0	0
LINE	L1	L1_Mus1	chr8	78759451	78765556	-	0	0	0

Simple_repeat	Simple_repeat	(A)n	chr8	78949564	78949592	+	0	0	0
SINE	Alu	B1_Mm	chr8	80159529	80159675	-	0	0	0
Simple_repeat	Simple_repeat	(A)n	chr8	80614288	80614323	+	0.591053	0	0
SINE	Alu	B1_Mm	chr8	80693450	80693596	+	0	0	0
SINE	Alu	B1_Mm	chr8	82002684	82002829	-	0	0	0
LINE	L1	Lx	chr8	82331942	82338062	-	0	0	0
SINE	Alu	B1_Mus2	chr8	82898210	82898347	-	0	0	0
SINE	Alu	B1_Mur4	chr8	83271368	83271515	+	0	0	0
Simple_repeat	Simple_repeat	(A)n	chr8	83271516	83271562	+	0	0	0
SINE	Alu	B1_Mus2	chr8	83281797	83281943	-	0.591053	0.708814	0
LTR	MaLR	MTA_Mm	chr11	90164611	90165009	+	0	0	0
Simple_repeat	Simple_repeat	(A)n	chr8	85175047	85175114	+	0	0	0
SINE	Alu	B1_Mm	chr8	86641105	86641236	+	0	0	0
Simple_repeat	Simple_repeat	(A)n	chr8	86641237	86641263	+	0	0	0
SINE	Alu	B1_Mus2	chr8	86802241	86802389	-	0	0	0
SINE	Alu	B1_Mm	chr11	90583153	90583299	-	0	0	0
SINE	Alu	B1_Mus2	chr11	90619878	90620024	-	0	0	0
SINE	Alu	B1_Mus2	chr8	87419075	87419220	-	0	0	0
SINE	Alu	B1_Mus1	chr8	87461716	87461862	-	0	0.708814	0
LTR	MaLR	MTA_Mm	chr8	87628089	87628469	+	0	0	0
SINE	Alu	B1_Mm	chr8	87660541	87660687	-	0	0	0
LTR	ERV1	RMER21A	chr8	87744729	87745752	+	0	0	0
SINE	Alu	B1_Mus1	chr8	87979208	87979349	-	0	0	0
SINE	Alu	B1_Mm	chr8	88013735	88013873	+	0	0	0
Simple_repeat	Simple_repeat	(A)n	chr8	88013874	88013897	+	0	0	0
LTR	MaLR	MTA_Mm	chr11	90778234	90778629	+	0	0	0
SINE	Alu	B1_Mm	chr8	90494380	90494529	+	0	0	0
Simple_repeat	Simple_repeat	(A)n	chr8	90494530	90494551	+	0	0	0
Simple_repeat	Simple_repeat	(A)n	chr8	90508017	90508051	+	0	0	0
Simple_repeat	Simple_repeat	(A)n	chr11	91001680	91001712	+	0	0	0
SINE	Alu	B1_Mm	chr8	90703388	90703516	+	0	0	0.615324
Simple_repeat	Simple_repeat	(A)n	chr8	90703517	90703540	+	0	0	0.957947
SINE	Alu	B1_Mm	chr8	90720235	90720381	+	0	0	0
Simple_repeat	Simple_repeat	(A)n	chr8	90720382	90720422	+	0	0	0
SINE	Alu	B1_Mm	chr8	90791429	90791546	+	0	0	0
SINE	Alu	PB1D7	chr8	90854945	90855058	-	2.69641	0	1.57327
SINE	Alu	PB1D10	chr8	92718421	92718508	+	0	0	0
Simple_repeat	Simple_repeat	(A)n	chr8	92718509	92718535	+	0	0	0
Simple_repeat	Simple_repeat	(A)n	chr8	95078434	95078473	+	0	0	0
LTR	MaLR	MTA_Mm	chr8	96257382	96257776	+	0	0	0
SINE	Alu	B1_Mus2	chr8	97221630	97221775	-	0	0	0
SINE	Alu	B1_Mm	chr8	98378650	98378796	+	0	0	0
Simple_repeat	Simple_repeat	(A)n	chr8	98378797	98378822	+	0	0	0
SINE	Alu	B1_Mus1	chr8	98380193	98380339	-	0	0	0
LTR	MaLR	MTD	chr8	98923439	98923851	-	0	0	0
LTR	MaLR	MTA_Mm	chr8	99478308	99478703	-	0	0	0
SINE	Alu	B1_Mus2	chr8	99811309	99811454	-	0	0	0
Simple_repeat	Simple_repeat	(A)n	chr8	99885489	99885510	+	0	0	0
LINE	L1	L1Md_T	chr8	10006921	10007584	-	0	0	0
LTR	MaLR	MTA_Mm	chr8	10118281	10118320	+	0	0	0
SINE	Alu	B1_Mus1	chr8	10121508	10121521	-	0	0	0
Simple_repeat	Simple_repeat	(A)n	chr8	10216197	10216201	+	0.332194	0	0
Simple_repeat	Simple_repeat	(A)n	chr8	10299117	10299120	+	0	0	0
Simple_repeat	Simple_repeat	(A)n	chr8	10331432	10331436	+	0.332194	0	0
Simple_repeat	Simple_repeat	(A)n	chr8	10342302	10342308	+	0	0	0
SINE	Alu	B1_Mm	chr8	10366987	10367002	+	0	0	0
Simple_repeat	Simple_repeat	(A)n	chr8	10367002	10367005	+	0	0	0
Simple_repeat	Simple_repeat	(A)n	chr8	10387771	10387775	+	0	0	0
LTR	MaLR	MTA_Mm	chr8	10434075	10434115	+	0	0	0
LINE	L1	L1Md_T	chr8	10466535	10467248	-	0	0	0
LINE	L1	L1Md_F2	chr8	10484053	10484626	+	0	0	0
Simple_repeat	Simple_repeat	(A)n	chr8	10542351	10542356	+	0	0	0
Simple_repeat	Simple_repeat	(A)n	chr8	10652139	10652142	+	0	0	0
SINE	Alu	B1_Mm	chr8	10715091	10715106	+	0	0	0
Simple_repeat	Simple_repeat	(A)n	chr8	10715106	10715113	+	0	0	0
Simple_repeat	Simple_repeat	(A)n	chr8	10762592	10762595	+	0	0	0
SINE	Alu	B1_Mus2	chr8	10895255	10895269	-	0	0	0

SINE	Alu	B1_Mus1	chr8	10910849	10910863	-	0	0	0
SINE	Alu	B1_Mm	chr8	10937630	10937644	+	0	0	0
Simple_repeat	Simple_repeat	(A)n	chr8	10937645	10937647	+	0.923247	0	0
SINE	Alu	B1_Mm	chr8	10963553	10963567	-	0	0	0
SINE	Alu	B1_Mus2	chr8	10974110	10974125	+	0	0	0
Simple_repeat	Simple_repeat	(A)n	chr8	10994992	10994995	+	0	0	0
SINE	B2	B2_Mm1t	chr8	11011517	11011536	+	0	0	0
SINE	Alu	PB1D10	chr8	11012684	11012695	-	0	0	0
SINE	Alu	B1_Mm	chr8	11024829	11024843	+	0	0	0
Simple_repeat	Simple_repeat	(A)n	chr8	11024843	11024846	+	0	0	0
SINE	Alu	B1_Mm	chr8	11032623	11032636	-	0	0	0
SINE	Alu	B1_Mus2	chr8	11037079	11037094	-	0	0	0
Simple_repeat	Simple_repeat	(A)n	chr8	11059637	11059641	+	0	0	0
SINE	Alu	B1_Mus2	chr8	11112737	11112751	-	0	0	0
SINE	Alu	B1_Mus2	chr8	11175831	11175846	-	1.77316	0	0
SINE	Alu	B1_Mur4	chr8	11181913	11181927	+	0	0	0.615324
SINE	Alu	B1_Mus2	chr8	11211317	11211332	+	0	0	0
Simple_repeat	Simple_repeat	(A)n	chr8	11211332	11211335	+	0	0	0.342623
SINE	Alu	B1_Mus2	chr8	11253238	11253253	-	0	0	0
Simple_repeat	Simple_repeat	(A)n	chr8	11271872	11271874	+	0	0	0
Simple_repeat	Simple_repeat	(TTTA)n	chr8	11416345	11416348	+	0	0	0
SINE	Alu	B1_Mus2	chr8	11446222	11446237	+	0	0	0
Simple_repeat	Simple_repeat	(A)n	chr8	11446237	11446245	+	0	0	0
SINE	Alu	B1_Mus2	chr8	11459308	11459323	+	0	0	0
Low_complexity	Low_complexity	A-rich	chr8	11459323	11459329	+	0	0	0
SINE	Alu	B1_Mm	chr8	11485893	11485907	-	10.9481	12.0857	16.4115
Simple_repeat	Simple_repeat	(A)n	chr8	11502283	11502294	+	0	0	0
Simple_repeat	Simple_repeat	(A)n	chr8	11503074	11503079	+	0	0	0
Simple_repeat	Simple_repeat	(A)n	chr8	11640966	11640970	+	0	0	0
LTR	MaLR	MTA_Mm	chr8	11814801	11814840	+	0	0	0
Simple_repeat	Simple_repeat	(A)n	chr8	11869772	11869775	+	0	0	0
SINE	Alu	B1_Mm	chr8	11877369	11877384	+	0	0	0
Simple_repeat	Simple_repeat	(GAA)n	chr8	11877384	11877392	+	0	0	0
Simple_repeat	Simple_repeat	(A)n	chr8	11887135	11887138	+	0	0	0
Simple_repeat	Simple_repeat	(A)n	chr8	11939972	11939976	+	0	0	0
LTR	MaLR	MTB	chr8	12041555	12041595	+	0	0	0
SINE	Alu	B1_Mus2	chr11	94255060	94255211	-	0	0	0
Simple_repeat	Simple_repeat	(A)n	chr8	12187645	12187648	+	0	0	0
Simple_repeat	Simple_repeat	(A)n	chr8	12240632	12240635	+	0	0	0
SINE	Alu	B1_Mus2	chr8	12243119	12243133	+	0	0	0
Simple_repeat	Simple_repeat	(A)n	chr8	12243133	12243136	+	0	0	0
LTR	MaLR	MTA_Mm	chr8	12257773	12257812	-	0	0	0
LTR	MaLR	MTA_Mm	chr8	12259379	12259418	+	0	0	0
SINE	B2	B2_Mm1t	chr8	12276899	12276918	-	0	0	0
SINE	Alu	B1_Mus2	chr8	12341458	12341472	-	0	0	0
SINE	Alu	B1_Mus2	chr8	12395968	12395982	-	0	0	0
SINE	Alu	B1_Mm	chr8	12497589	12497603	-	0	0	0
SINE	B4	ID_B1	chr11	94543115	94543335	-	0	0	0
SINE	Alu	B1_Mm	chr8	12508733	12508747	+	0	0	0
Simple_repeat	Simple_repeat	(A)n	chr8	12508748	12508750	+	0	0	0
SINE	Alu	B1_Mus2	chr8	12557504	12557519	+	0	0	0
Simple_repeat	Simple_repeat	(A)n	chr8	12557519	12557522	+	0	0	0
SINE	Alu	B1_Mus2	chr11	94596615	94596762	+	0	0	0
Simple_repeat	Simple_repeat	(A)n	chr11	94596763	94596788	+	0	0	0
LTR	MaLR	MTB	chr8	12615026	12615066	-	0	0	0
SINE	Alu	B1_Mus2	chr8	12639395	12639409	-	0	0	0
SINE	Alu	B1_Mus2	chr8	12639417	12639432	+	0	0	0
Simple_repeat	Simple_repeat	(A)n	chr8	12639432	12639434	+	0	0	0
LTR	MaLR	MTA_Mm	chr8	12707709	12707748	+	0	0	0
SINE	Alu	B1_Mus1	chr8	12756930	12756944	-	0	0	0.615324
SINE	Alu	B1_Mm	chr11	94733525	94733665	-	0	0	0.342623
Simple_repeat	Simple_repeat	(A)n	chr8	12790073	12790077	+	0.332194	0	0
Simple_repeat	Simple_repeat	(A)n	chr8	12810565	12810569	+	0	0	0
LTR	MaLR	MTA_Mm	chr8	12842368	12842407	-	0	0	0
Simple_repeat	Simple_repeat	(A)n	chr8	12866146	12866149	+	0	0	0
LTR	MaLR	MTA_Mm	chr8	12868505	12868545	-	0	0	0.342623
SINE	Alu	B1_Mus1	chr8	12943801	12943815	+	0	0	0

Simple_repeat	Simple_repeat	(A)n	chr8	12943815	12943818	+	0	0	0
SINE	Alu	B1_Mus2	chr8	12950230	12950245	+	0	0	0
Simple_repeat	Simple_repeat	(A)n	chr8	12950245	12950257	+	0	0	0
SINE	Alu	B1_Mus1	chr8	13029316	13029329	+	0	0	0
Simple_repeat	Simple_repeat	(A)n	chr8	13029329	13029337	+	0	0	0
LINE	L1	L1Md_F2	chr8	13039370	13039838	+	0	0	0
LTR	MaLR	MTB	chr8	13059788	13059828	+	0	0	0
Simple_repeat	Simple_repeat	(A)n	chr8	13118066	13118069	+	0	0	0
SINE	Alu	B1_Mus2	chr11	95035331	95035477	+	0	0	0
Simple_repeat	Simple_repeat	(A)n	chr11	95035478	95035500	+	0	0	0
LTR	MaLR	MTA_Mm	chr9	3291564	3291953	-	0	0	0
SINE	Alu	B1_Mus2	chr11	95112300	95112445	+	0	0	0
Simple_repeat	Simple_repeat	(A)n	chr11	95112446	95112473	+	0	0	0
Simple_repeat	Simple_repeat	(A)n	chr9	5039482	5039521	+	0	0	0
Simple_repeat	Simple_repeat	(A)n	chr9	5127787	5127809	+	0	0	0
LTR	MaLR	MTA_Mm	chr9	5166358	5166752	+	0	0	0
SINE	Alu	B1_Mus1	chr9	5189843	5189977	-	0	0	0
SINE	Alu	B1_Mus1	chr9	5245575	5245712	+	0	0	0
Simple_repeat	Simple_repeat	(A)n	chr9	5245713	5245754	+	0	0	0
SINE	Alu	B1_Mus2	chr9	5491705	5491849	+	0	0	0.342623
SINE	Alu	B1_Mm	chr9	5577244	5577390	-	0	0	0
SINE	Alu	B1_Mus2	chr9	5645142	5645288	+	0	0	0
Simple_repeat	Simple_repeat	(GAAA)n	chr9	5645289	5645349	+	0	0	0
SINE	Alu	B1_Mus2	chr9	5848659	5848805	-	0	0	0
Simple_repeat	Simple_repeat	(A)n	chr10	27462752	27462806	+	0	0	0
LTR	MaLR	MTB	chr9	7255216	7255613	+	0	0	0
LTR	MaLR	MTA_Mm	chr9	7267408	7267803	+	0	0	0
LTR	MaLR	MTA_Mm	chr9	7268900	7269295	+	0.332194	0	0.615324
Simple_repeat	Simple_repeat	(A)n	chr9	7593443	7593483	+	0	0	0
Simple_repeat	Simple_repeat	(A)n	chr9	7684180	7684228	+	0	0	0
SINE	Alu	B1_Mus1	chr9	7899052	7899198	+	0	0	0
Simple_repeat	Simple_repeat	(A)n	chr9	7899199	7899257	+	0	0	0
SINE	Alu	B1_Mus1	chr9	7911626	7911743	-	0	0	0
LTR	MaLR	MTA_Mm	chr9	8508040	8508435	+	0	0	0
SINE	Alu	B1_Mm	chr9	9277938	9278084	-	0	0	0
SINE	Alu	B1_Mus2	chr11	95468586	95468734	+	0	0	0
Simple_repeat	Simple_repeat	(A)n	chr11	95468735	95468771	+	0	0	0
LTR	MaLR	MTA_Mm	chr9	9361024	9361419	+	0.591053	0	0
SINE	Alu	B1_Mm	chr11	95499213	95499358	-	0	0	0
LTR	MaLR	MTA_Mm	chr9	11419025	11419419	+	0	0	0
LTR	ERVK	RLTR45	chr9	11513613	11514038	-	0.332194	0	0
LINE	L1	L1Md_F3	chr9	12533593	12539976	+	0	0	0
Simple_repeat	Simple_repeat	(A)n	chr9	12880296	12880328	+	0	0	0
Simple_repeat	Simple_repeat	(A)n	chr9	12951421	12951462	+	0	0	0
SINE	Alu	B1_Mm	chr9	13138414	13138560	+	0	0	0
SINE	Alu	B1_Mus2	chr9	13331449	13331594	+	0.923247	0	0
Simple_repeat	Simple_repeat	(A)n	chr9	13331595	13331618	+	0.591053	0	0
SINE	Alu	B1_Mus2	chr9	13515354	13515499	+	0	0.708814	0
Simple_repeat	Simple_repeat	(A)n	chr9	13515500	13515525	+	0	0.708814	0
SINE	Alu	B1_Mus1	chr9	14432424	14432571	+	0	0	0
LINE	L1	L1Md_A	chr10	27532354	27539767	-	0	0	0
SINE	Alu	B1_Mus2	chr9	14684447	14684593	+	0	0.380568	0
Simple_repeat	Simple_repeat	(A)n	chr9	14684594	14684618	+	0	0	0
Simple_repeat	Simple_repeat	(A)n	chr9	14762485	14762517	+	0	0	0
SINE	Alu	B1_Mus1	chr11	95789649	95789796	-	0	0	0
LTR	MaLR	MTA_Mm	chr9	16786370	16786764	+	0	0	0
SINE	Alu	B1_Mus2	chr9	17779615	17779761	-	0	0	0
LINE	L1	L1_Mus1	chr9	17782484	17787747	+	0	0	0
SINE	Alu	B1_Mus2	chr9	18334960	18335083	-	0	0	0
SINE	Alu	B1_Mus2	chr9	18908476	18908621	-	0	0	0
SINE	Alu	B1_Mus1	chr9	19230144	19230250	-	0	0	0
SINE	Alu	B1_Mm	chr9	19673863	19674008	-	0	0	0
LTR	MaLR	MTA_Mm	chr9	19839545	19839927	+	0	0	0
Simple_repeat	Simple_repeat	(A)n	chr9	20440314	20440349	+	0	0	0
LTR	ERV1	RLTR23	chr9	20689507	20689895	-	0	0	0
SINE	Alu	B1_Mm	chr9	20689919	20690065	-	0	0	0
Simple_repeat	Simple_repeat	(A)n	chr11	96045752	96045792	+	0	0	0

SINE	Alu	B1_Mus2	chr9	20964695	20964848	-	0	0	0.615324
Simple_repeat	Simple_repeat	(A)n	chr9	21170237	21170286	+	0	0	0
SINE	Alu	B1_Mm	chr9	21401698	21401844	+	0	0	0
Simple_repeat	Simple_repeat	(A)n	chr9	21401845	21401879	+	0	0	0
Simple_repeat	Simple_repeat	(A)n	chr11	96141884	96141922	+	0	0	0
SINE	Alu	B1_Mm	chr9	21665077	21665223	-	0	0	0
Simple_repeat	Simple_repeat	(A)n	chr9	21897352	21897387	+	6.07381	3.52684	6.05776
SINE	Alu	B1_Mm	chr9	21945636	21945781	+	0	0	0
Simple_repeat	Simple_repeat	(A)n	chr9	21945782	21945806	+	0	0	0
SINE	Alu	B1_Mus2	chr9	22076163	22076309	+	0	0	0
Simple_repeat	Simple_repeat	(A)n	chr9	22076310	22076335	+	0	0	0
SINE	Alu	B1_Mus2	chr9	22119795	22119945	+	0	0	0
Simple_repeat	Simple_repeat	(A)n	chr9	22119946	22119970	+	0	0	0
LTR	MaLR	MTA_Mm	chr9	22963723	22964118	-	0	0	0
SINE	Alu	B1_Mus1	chr9	23305963	23306101	+	0	0	0
SINE	Alu	B1_Mus2	chr9	23382872	23383018	+	0	0	0
Simple_repeat	Simple_repeat	(A)n	chr9	23383019	23383091	+	0	0	0
LTR	MaLR	MTA_Mm	chr9	23394251	23394649	+	0	0.380568	0
SINE	Alu	B1_Mur3	chr9	23494365	23494502	-	0	0	0
LTR	MaLR	MTA_Mm	chr9	23871584	23871980	+	0	0	0
Simple_repeat	Simple_repeat	(A)n	chr9	24212587	24212624	+	0	0	0
SINE	Alu	B1_Mur4	chr9	24641035	24641219	+	0	0	0
Simple_repeat	Simple_repeat	(A)n	chr9	24641220	24641249	+	0	0	0
SINE	Alu	B1_Mus1	chr9	24730533	24730679	+	0	0	0
Simple_repeat	Simple_repeat	(A)n	chr9	24730680	24730733	+	0	0	0
LTR	MaLR	MTA_Mm	chr9	24733785	24734181	-	0	0	0
Simple_repeat	Simple_repeat	(A)n	chr9	24847570	24847607	+	0	0	0
SINE	B2	B2_Mm1t	chr9	25674550	25674746	+	0	0	0
Low_complexity	Low_complexity	AT_rich	chr9	25674747	25674767	+	0	0	0
SINE	Alu	B1_Mus1	chr9	25821210	25821355	-	0	0	0
LTR	MaLR	MTA_Mm	chr9	25889214	25889606	+	0	0	0
LTR	MaLR	MTA_Mm	chr9	25890701	25891094	+	0	0	0.342623
LINE	L1	L1Md_F2	chr9	26149394	26155666	-	0	0	0
Simple_repeat	Simple_repeat	(A)n	chr9	26289114	26289152	+	0	0	0
SINE	Alu	B1_Mus2	chr9	26964459	26964604	-	0	0	0
SINE	Alu	B1_Mm	chr11	96778318	96778455	+	0.591053	0	0
Simple_repeat	Simple_repeat	(A)n	chr11	96778456	96778479	+	0.923247	0.708814	0
Simple_repeat	Simple_repeat	(A)n	chr9	30321323	30321368	+	0	0	0
SINE	Alu	B1_Mur4	chr10	27737512	27737662	+	0	0	0
Simple_repeat	Simple_repeat	(GAAA)n	chr10	27737663	27737740	+	0	0	0
SINE	Alu	B1_Mus2	chr9	30815700	30815846	+	0	0	0
Simple_repeat	Simple_repeat	(A)n	chr9	30815847	30815880	+	0	0	0
SINE	Alu	B1_Mus2	chr9	31098915	31099060	+	0	0	0
Simple_repeat	Simple_repeat	(A)n	chr9	31099061	31099086	+	0	0	0
SINE	Alu	B1_Mus1	chr9	31363750	31363897	+	0	0	0.342623
Simple_repeat	Simple_repeat	(A)n	chr9	31363898	31363920	+	0	0	0
SINE	Alu	B1_Mus2	chr9	31893915	31894060	+	6.21866	3.21582	5.24386
Simple_repeat	Simple_repeat	(A)n	chr9	31894061	31894089	+	3.39388	1.08938	3.14654
SINE	Alu	B1_Mus2	chr9	32659305	32659447	+	0	0	0
Simple_repeat	Simple_repeat	(A)n	chr9	32659448	32659471	+	0	0	0
Simple_repeat	Simple_repeat	(A)n	chr9	32919547	32919580	+	0	0	0
LTR	MaLR	MTA_Mm	chr9	33241160	33241553	-	0	0	0
Simple_repeat	Simple_repeat	(A)n	chr9	35630443	35630469	+	0	0	0
Simple_repeat	Simple_repeat	(A)n	chr11	97149068	97149138	+	0	0	0.615324
SINE	B2	B2_Mm1a	chr11	97218889	97219079	+	0	0	0
Simple_repeat	Simple_repeat	(A)n	chr11	97219080	97219100	+	0	0	0
SINE	Alu	B1_Mus1	chr9	37288206	37288359	-	0.332194	0	0
SINE	Alu	B1_Mus1	chr9	38147881	38148018	-	0	0	0
SINE	Alu	B1_Mus1	chr9	38284092	38284238	+	0	0	0
Simple_repeat	Simple_repeat	(A)n	chr9	38284239	38284271	+	0	0	0
Simple_repeat	Simple_repeat	(A)n	chr9	38313931	38314001	+	0	0	0
Simple_repeat	Simple_repeat	(A)n	chr9	39221060	39221117	+	0	0	0
SINE	Alu	B1_Mus1	chr9	39323842	39323989	-	0	0	0
SINE	Alu	B1_Mus2	chr9	39481381	39481529	-	0	0	0
SINE	Alu	B1_Mm	chr9	39660495	39660640	-	0	0	0
SINE	Alu	B1_Mm	chr9	39993078	39993223	-	0	0	0.615324
Simple_repeat	Simple_repeat	(A)n	chr9	39998710	39998746	+	0	0	0

Simple_repeat	Simple_repeat	(TTTA)n	chr9	40030457	40030496	+	0	0	0
SINE	Alu	B1_Mus2	chr9	40400353	40400498	+	0	0	0
Simple_repeat	Simple_repeat	(A)n	chr9	40400499	40400532	+	0	0	0
DNA	MER1_type	RCHARR1	chr9	41957231	41958174	+	0	0	0
LTR	MaLR	MTA_Mm	chr9	42767936	42768331	+	0.591053	0	0
SINE	Alu	B1_Mm	chr9	42927531	42927676	+	0	0	0
Simple_repeat	Simple_repeat	(A)n	chr9	42927677	42927711	+	0.332194	0	0
SINE	Alu	B1_Mus2	chr9	43385020	43385150	+	0	0	0
Simple_repeat	Simple_repeat	(A)n	chr9	43385151	43385218	+	0	0	0
Simple_repeat	Simple_repeat	(A)n	chr9	43658833	43658878	+	0	0	0
SINE	Alu	B1_Mus2	chr9	43683869	43684014	+	0	0	0
Simple_repeat	Simple_repeat	(A)n	chr9	43684015	43684061	+	0	0	0
SINE	Alu	B1_Mm	chr9	43713452	43713598	+	0	0	0
Simple_repeat	Simple_repeat	(A)n	chr9	43713599	43713653	+	0	0	0
SINE	Alu	B1_Mus2	chr11	97656665	97656805	+	0	0	0
Simple_repeat	Simple_repeat	(A)n	chr11	97656806	97656835	+	0	0	0
SINE	Alu	B1_Mm	chr11	97701056	97701190	+	0	0	0
Simple_repeat	Simple_repeat	(A)n	chr11	97701191	97701235	+	0	0	0
SINE	Alu	B1_Mus1	chr9	44133527	44133674	-	0	0	0
SINE	Alu	B1_Mm	chr9	44227857	44227986	+	0	0	0
SINE	Alu	B1_Mm	chr9	44266842	44266988	-	0	0	0
SINE	Alu	B1_Mus1	chr9	44348512	44348659	-	0	0	0
SINE	Alu	B1_Mus1	chr11	97748173	97748318	+	0	0	0
Simple_repeat	Simple_repeat	(A)n	chr11	97748319	97748350	+	0	0	0
SINE	Alu	B1_Mus2	chr9	45645966	45646114	-	0	0	0
SINE	B2	B2_Mm1a	chr9	45767447	45767639	+	0	0	0
Simple_repeat	Simple_repeat	(A)n	chr9	45767640	45767666	+	0	0.708814	0
SINE	Alu	B1_Mm	chr9	46144661	46144806	-	0	0	0
SINE	Alu	B1_Mus1	chr9	46349981	46350128	-	0	0	0
Simple_repeat	Simple_repeat	(A)n	chr9	46369485	46369526	+	0	0	0.615324
Simple_repeat	Simple_repeat	(A)n	chr9	48296496	48296535	+	0	0	0
LTR	MaLR	MTA_Mm	chr9	48692351	48692745	-	0	0	0
LTR	MaLR	MTA_Mm	chr9	48734513	48734913	+	0	0	0
LTR	MaLR	MTA_Mm	chr9	48738494	48738892	-	0	0	0
SINE	Alu	B1_Mm	chr9	48965994	48966130	+	0	0	0
Simple_repeat	Simple_repeat	(A)n	chr9	48966131	48966155	+	0	0	0
SINE	B4	B4	chr9	48984039	48984350	-	0	0	0
LTR	MaLR	MTA_Mm	chr9	49242116	49242510	+	0	0	0
Simple_repeat	Simple_repeat	(A)n	chr9	49809174	49809208	+	0	0	0
Simple_repeat	Simple_repeat	(A)n	chr9	50115259	50115294	+	0	0	0
SINE	B2	B2_Mm1a	chr9	50208684	50208876	-	0	0	0
SINE	B2	B2_Mm1t	chr9	50437487	50437679	+	0	0.380568	0.615324
SINE	Alu	B1_Mm	chr9	50849961	50850100	+	0	0	0
Simple_repeat	Simple_repeat	(A)n	chr9	50850101	50850123	+	0	0	0
Simple_repeat	Simple_repeat	(A)n	chr9	51342700	51342741	+	0	0	0
SINE	Alu	B1_Mur3	chr9	51720923	51721063	+	0	0	0
SINE	Alu	B1_Mm	chr9	52146399	52146545	-	0	0	0
Simple_repeat	Simple_repeat	(A)n	chr9	52260007	52260057	+	0	0	0
Simple_repeat	Simple_repeat	(A)n	chr9	53592235	53592268	+	0	0	0
LTR	MaLR	MTB	chr9	53607181	53607529	+	0	0	0
SINE	Alu	B1_Mm	chr9	54687197	54687343	+	0	0	0
Simple_repeat	Simple_repeat	(A)n	chr9	54687344	54687375	+	0	0	0
Simple_repeat	Simple_repeat	(A)n	chr11	98458939	98458976	+	0	0	0
LTR	ERV1	RMER5	chr9	54955204	54955685	-	0	0	0
SINE	B2	B2_Mm1t	chr9	54958893	54959082	-	0	0	0
SINE	B2	B2_Mm1t	chr9	54964508	54964702	+	0	0	0
SINE	Alu	B1_Mus1	chr9	54991821	54991968	-	0	0	0
SINE	Alu	B1_Mus1	chr9	55921402	55921548	+	0	0	0.615324
LTR	ERVK	RMER17C	chr9	55921549	55921910	+	0	0	0.615324
SINE	Alu	B1_Mm	chr9	56415397	56415542	+	0	0	0
SINE	B2	B2_Mm1a	chr9	56590269	56590461	-	0.591053	0	0
SINE	Alu	B1_Mm	chr9	56651763	56651908	+	0	0	0
Simple_repeat	Simple_repeat	(A)n	chr9	56651909	56651934	+	0	0	0
SINE	Alu	B1_Mus2	chr9	56832323	56832468	+	1.77316	0.380568	1.23065
Simple_repeat	Simple_repeat	(A)n	chr9	56832469	56832511	+	0.591053	0.380568	1.57327
SINE	B2	B2_Mm1a	chr9	57496781	57496984	+	0	0	0
SINE	Alu	B1_Mus2	chr9	57555431	57555577	-	0	0	0

SINE	Alu	B1_Mm	chr9	57618469	57618614	-	0.332194	0	0
SINE	Alu	B1_Mus2	chr9	57818537	57818694	+	0	0	0
Simple_repeat	Simple_repeat	(A)n	chr9	57818695	57818717	+	0	0	0
SINE	Alu	B1_Mur2	chr9	58057786	58057927	+	0.591053	0	0
SINE	B2	B2_Mm1a	chr9	58428496	58428689	-	0	0	0
SINE	Alu	B1_Mus2	chr9	59359654	59359801	+	0	0	0
Simple_repeat	Simple_repeat	(A)n	chr9	59359802	59359823	+	0.591053	0	0
SINE	B2	B3A	chr9	59359824	59359979	+	0.591053	0	0
SINE	Alu	B1_Mus1	chr9	59376565	59376710	-	0	0	0.342623
SINE	Alu	PB1D9	chr9	59414309	59414425	-	0	0	0
SINE	Alu	B1_Mm	chr11	98889294	98889420	+	0	0	0.342623
Simple_repeat	Simple_repeat	(A)n	chr11	98889421	98889446	+	0	0	0.342623
Simple_repeat	Simple_repeat	(TTTA)n	chr9	60399934	60399955	+	0	0	0
SINE	Alu	B1_Mur4	chr11	98957602	98957740	-	0	0	0
SINE	Alu	B1_Mus1	chr9	60888404	60888547	+	0	0	0
Simple_repeat	Simple_repeat	(A)n	chr9	60888548	60888595	+	0	0	0
Simple_repeat	Simple_repeat	(A)n	chr9	61544106	61544171	+	0	0	0
Simple_repeat	Simple_repeat	(A)n	chr9	61587495	61587532	+	0	0	0
LTR	MaLR	MTA_Mm	chr9	61599381	61599779	+	0	0	0
SINE	B2	B2_Mm1a	chr9	61721718	61721908	+	0	0	0
Simple_repeat	Simple_repeat	(CAAAA)n	chr9	61721909	61721926	+	0.332194	0.380568	0
SINE	Alu	B1_Mus1	chr9	61763731	61763878	-	0	0	0
SINE	Alu	B1_Mus2	chr9	62058379	62058524	+	0	0	0
SINE	Alu	B1_Mus2	chr9	62158118	62158264	+	0	0	0
LTR	MaLR	MTA_Mm	chr9	62470928	62471323	+	0	0	0
SINE	Alu	B1_Mus1	chr9	62935639	62935774	+	0	0	0
Simple_repeat	Simple_repeat	(A)n	chr9	62935775	62935799	+	0	0	0
SINE	Alu	B1_Mm	chr9	63001853	63001978	+	0	0	0
SINE	Alu	B1_Mus2	chr11	99200152	99200298	+	0	0	0
Simple_repeat	Simple_repeat	(A)n	chr11	99200299	99200320	+	0	0	0
SINE	Alu	B1_Mus1	chr9	63698734	63698881	-	0	0	0
Simple_repeat	Simple_repeat	(A)n	chr11	99259477	99259503	+	0	0	0
SINE	B4	ID_B1	chr9	63954152	63954368	+	0	0	0
SINE	Alu	B1_Mus2	chr9	64112392	64112537	-	0	0	0
SINE	Alu	B1_Mus2	chr9	64196817	64196961	+	0	0	0
Simple_repeat	Simple_repeat	(A)n	chr9	64196962	64196992	+	0	0	0
SINE	Alu	B1_Mus1	chr9	64209943	64210095	-	0	0	0
Simple_repeat	Simple_repeat	(A)n	chr11	99326369	99326409	+	0	0	0
Simple_repeat	Simple_repeat	(A)n	chr9	64643968	64644055	+	0	0	0
Simple_repeat	Simple_repeat	(T)n	chr11	99419732	99419786	+	0	0.380568	0
SINE	Alu	B1_Mus1	chr9	65035204	65035351	-	0	0	0
Simple_repeat	Simple_repeat	(TTTA)n	chr9	65094002	65094043	+	0	0	0
Simple_repeat	Simple_repeat	(A)n	chr9	65186181	65186232	+	0	0	0
Simple_repeat	Simple_repeat	(A)n	chr9	65434355	65434378	+	0	0	0.342623
SINE	Alu	B1_Mus2	chr9	65473444	65473587	-	0	0	0
SINE	Alu	B1_Mus2	chr9	66150414	66150554	-	0	0	0
LTR	MaLR	MTA_Mm	chr11	99760709	99761105	+	0	0	0
SINE	Alu	B1_Mm	chr9	66846291	66846435	-	0	0	0
SINE	B2	B2_Mm1a	chr9	66898098	66898288	+	0	0	0
Simple_repeat	Simple_repeat	(A)n	chr9	66898289	66898324	+	1.18211	0.380568	0
Simple_repeat	Simple_repeat	(A)n	chr9	66957409	66957438	+	0	0	0
LTR	MaLR	MTB	chr9	67013850	67014152	+	0	0	0
SINE	Alu	B1_Mus2	chr11	99829937	99830083	+	0	0	0
Simple_repeat	Simple_repeat	(A)n	chr11	99830084	99830109	+	0	0	0
SINE	B4	B4	chr9	68021105	68021344	-	0	0.380568	0
SINE	B2	B2_Mm1t	chr9	68021354	68021543	-	0	0	0
LTR	MaLR	MTB	chr9	69550849	69551254	+	0	0	0
SINE	Alu	B1_Mus2	chr9	69859642	69859793	-	0	0	0
SINE	B2	B2_Mm1a	chr9	69871683	69871867	-	0	0	0
SINE	Alu	B1_Mm	chr9	69934500	69934644	-	0	0	0
LTR	MaLR	MTB	chr9	72118897	72119341	+	0	0	0
Simple_repeat	Simple_repeat	(A)n	chr9	72241773	72241817	+	0	0	0
SINE	Alu	B1_Mus1	chr9	72354561	72354707	-	0	0	0
SINE	Alu	B1_Mus1	chr9	72472519	72472666	-	0	0	0
SINE	Alu	B1_Mus2	chr11	10040477	10040491	-	0	0	0
SINE	Alu	B1_Mm	chr9	73293988	73294134	+	0	0	0
Simple_repeat	Simple_repeat	(A)n	chr9	73314792	73314824	+	0.591053	0	0.342623

LTR	MaLR	MTA_Mm	chr9	73927013	73927410	-	0	0	0
SINE	Alu	B1_Mm	chr9	74444053	74444196	+	0	0	0
Simple_repeat	Simple_repeat	(A)n	chr9	74763531	74763564	+	0.591053	0	0
SINE	Alu	B1_Mus1	chr9	75268415	75268569	-	0	0	0
Simple_repeat	Simple_repeat	(A)n	chr9	75620680	75620716	+	0	0	0
LTR	MaLR	MTA_Mm	chr9	77595262	77595659	-	0	0	0
SINE	Alu	B1_Mm	chr11	10081022	10081036	+	0	0	0
Simple_repeat	Simple_repeat	(A)n	chr11	10086472	10086479	+	0	0	0
Simple_repeat	Simple_repeat	(A)n	chr9	79866111	79866133	+	0	0	0
LINE	L1	L1Md_F2	chr9	81235045	81241032	-	0.591053	0.708814	0.615324
LTR	MaLR	MTA_Mm	chr9	81260388	81260783	-	0.591053	1.41763	1.23065
LTR	MaLR	MTA_Mm	chr9	81568796	81569153	-	0	0	0
SINE	Alu	B1_Mur3	chr11	10100067	10100082	-	0	0	0
SINE	Alu	B1_Mus1	chr11	10100464	10100478	+	0	0	0
Simple_repeat	Simple_repeat	(A)n	chr11	10100478	10100481	+	0	0.761137	0
SINE	Alu	B1_Mus2	chr9	82043569	82043714	+	0.591053	0.708814	0.615324
Low_complexity	Low_complexity	A-rich	chr9	82043715	82043814	+	0.591053	0.708814	0.615324
SINE	Alu	B1_Mm	chr9	82202120	82202266	-	0	0	0
Simple_repeat	Simple_repeat	(A)n	chr9	82266385	82266419	+	0	0	0
Simple_repeat	Simple_repeat	(A)n	chr9	82532544	82532569	+	0	0	0
SINE	Alu	B1_Mus2	chr9	82751554	82751699	-	0	0	0
SINE	Alu	B1_Mus1	chr9	83496508	83496655	+	0.591053	0.708814	0
Simple_repeat	Simple_repeat	(A)n	chr9	83523699	83523733	+	0.591053	0	0
SINE	Alu	B1_Mus1	chr9	83526443	83526573	-	0	0	0
Simple_repeat	Simple_repeat	(A)n	chr9	84174911	84174938	+	0	0	0
Simple_repeat	Simple_repeat	(A)n	chr9	84279596	84279625	+	0	0	0
LINE	L1	Lx3_Mus	chr9	84308217	84312517	-	0	0	0
LTR	MaLR	MTA_Mm	chr9	85015323	85015707	+	0	0	0
LTR	MaLR	MTA_Mm	chr9	85087100	85087491	-	0	0	0
SINE	Alu	B1_Mus1	chr9	85206814	85206966	+	0	0	0
Simple_repeat	Simple_repeat	(GAA)n	chr9	85206968	85207069	+	0	0	0
Simple_repeat	Simple_repeat	(A)n	chr9	85298142	85298174	+	0	0	0
Simple_repeat	Simple_repeat	(A)n	chr9	85485563	85485591	+	0	0	0
SINE	Alu	B1_Mm	chr9	85540958	85541105	+	0	0	0
Simple_repeat	Simple_repeat	(A)n	chr9	85541106	85541125	+	0	0	0
LTR	MaLR	MTA_Mm	chr9	85569376	85569770	-	0	0	0
Simple_repeat	Simple_repeat	(A)n	chr9	85727125	85727160	+	0	0	0
LTR	MaLR	MTA_Mm	chr9	85980437	85980832	+	0	0	0
LTR	MaLR	MTA_Mm	chr9	86029465	86029858	-	0	0	0
LTR	MaLR	MTA_Mm	chr9	86030957	86031350	-	0	0	0
LTR	MaLR	MTA_Mm	chr9	87250535	87250928	+	0	0	0
Simple_repeat	Simple_repeat	(A)n	chr9	87297754	87297780	+	0	0	0
SINE	B4	B4	chr9	87323697	87323945	-	0	0	0
LINE	L1	Lx	chr9	88196359	88201999	+	0	0	0
SINE	Alu	B1_Mm	chr9	88435354	88435497	-	0	0	0
SINE	Alu	B1_Mus2	chr9	88976380	88976524	+	0	0	0
SINE	Alu	B1_Mm	chr9	88994319	88994468	+	0	0	0
Simple_repeat	Simple_repeat	(A)n	chr9	88994469	88994499	+	0	0	0
LTR	MaLR	MTA_Mm	chr9	89201810	89202207	+	0.591053	0	0.615324
LTR	MaLR	MTA_Mm	chr9	89247070	89247457	-	0	0	0
LTR	MaLR	MTA_Mm	chr9	89255768	89256155	-	0	0	0
SINE	Alu	B1_Mus2	chr9	89260866	89261014	-	0	0	0.615324
SINE	Alu	B1_Mm	chr9	89942533	89942678	+	0	0	0
Low_complexity	Low_complexity	A-rich	chr9	89942679	89942763	+	0	0	0
SINE	Alu	B1_Mus2	chr9	89978268	89978404	-	1.18211	0.761137	0
SINE	Alu	B1_Mus2	chr9	90042441	90042586	+	2.26557	1.01984	1.97174
Simple_repeat	Simple_repeat	(A)n	chr9	90042587	90042615	+	2.26557	1.01984	1.97174
SINE	Alu	B1_Mus2	chr9	90463090	90463236	-	0	0	0
SINE	B2	B2_Mm1t	chr11	10157381	10157400	-	0	0	0
Simple_repeat	Simple_repeat	(A)n	chr9	92720575	92720616	+	0	0	0
Simple_repeat	Simple_repeat	(A)n	chr9	93038574	93038649	+	0	0	0
Simple_repeat	Simple_repeat	(A)n	chr9	93336904	93336929	+	0	0	0
LINE	L1	L1Md_F2	chr9	94019344	94023371	-	0	0	0
LTR	MaLR	MTA_Mm	chr9	94512606	94512994	-	0	0	0
SINE	Alu	B1_Mus2	chr9	95351793	95351939	+	0	0	0
Simple_repeat	Simple_repeat	(A)n	chr9	95351940	95351962	+	0	0	0
SINE	B2	B2_Mm1a	chr9	96268057	96268246	-	2.06356	0	0

SINE	Alu	B1_Mm	chr9	96288649	96288795	+	0	0	0
Simple_repeat	Simple_repeat	(A)n	chr9	96288796	96288821	+	0	0	0
Simple_repeat	Simple_repeat	(A)n	chr9	96718666	96718695	+	0	0.708814	0
SINE	Alu	B1_Mus2	chr11	10189010	10189025	-	0	0	0
SINE	Alu	B1_Mm	chr9	97055340	97055487	+	0	0	0
SINE	Alu	B1_Mur3	chr11	10191731	10191745	+	0	0	0
SINE	Alu	B1_Mus1	chr9	98827422	98827572	-	0.591053	0	0
Simple_repeat	Simple_repeat	(A)n	chr9	98832029	98832065	+	0.332194	0	0
LTR	MaLR	MTA_Mm	chr9	99121751	99122135	+	0	0	0
SINE	B2	B2_Mm2	chr9	99492689	99492862	-	0	0	0
LTR	MaLR	MTA_Mm	chr9	99578774	99579168	-	0	0	0
SINE	Alu	B1_Mus2	chr9	99584245	99584393	+	0	0	0
Simple_repeat	Simple_repeat	(A)n	chr9	99584394	99584429	+	0	0	0
SINE	Alu	B1_Mus1	chr11	10214340	10214355	+	0	0	0
Simple_repeat	Simple_repeat	(A)n	chr11	10214355	10214358	+	0	0	0
SINE	Alu	B1_Mus2	chr9	10047297	10047311	-	0	0	0
SINE	Alu	B1_Mus2	chr9	10053328	10053343	-	0	0	0
SINE	Alu	B1_Mm	chr11	10216314	10216328	+	0	0	0
Simple_repeat	Simple_repeat	(A)n	chr11	10216328	10216332	+	0	0	0
Simple_repeat	Simple_repeat	(A)n	chr9	10115842	10115846	+	0	0	0
Simple_repeat	Simple_repeat	(A)n	chr11	10220343	10220345	+	0	0	0
SINE	Alu	B1_Mm	chr11	10225156	10225170	+	0	0	0
Simple_repeat	Simple_repeat	(A)n	chr11	10225170	10225175	+	0	0	0
SINE	Alu	B1_Mm	chr9	10269506	10269520	-	0	0	0
SINE	Alu	B1_Mus1	chr9	10300548	10300564	-	0.591053	0	0
LTR	MaLR	MTD	chr9	10300685	10300728	+	0	0	0
SINE	Alu	B1_Mus2	chr11	10242123	10242137	-	0	0	0
SINE	Alu	B1_Mm	chr11	10243705	10243720	+	0	0	0
Simple_repeat	Simple_repeat	(A)n	chr11	10244890	10244892	+	0.591053	0	0.615324
Simple_repeat	Simple_repeat	(A)n	chr11	10245994	10245998	+	0.332194	0	0
LTR	MaLR	MTA_Mm	chr9	10546139	10546178	-	0	0	0
LTR	MaLR	MTA_Mm	chr9	10547731	10547770	+	0	0	0
SINE	Alu	B1_Mm	chr9	10574039	10574053	-	0	0	0
SINE	B2	B2_Mm1a	chr9	10600794	10600813	+	0	0	0.342623
Simple_repeat	Simple_repeat	(A)n	chr9	10600813	10600815	+	0	0	0
SINE	Alu	B1_Mus2	chr9	10603770	10603784	+	0	0	0
Low_complexity	Low_complexity	A-rich	chr9	10603784	10603789	+	0	0	0
SINE	Alu	B1_Mm	chr9	10612264	10612278	+	0	0	0
LTR	MaLR	MTB	chr9	10625466	10625504	+	0	0	0
SINE	Alu	B1_Mus1	chr10	28724109	28724256	-	0	0	0
SINE	Alu	B1_Mm	chr9	10820404	10820417	-	0	0	1.23065
SINE	Alu	B1_Mus2	chr9	10824805	10824819	+	0	0	0.615324
SINE	Alu	B1_Mm	chr9	10840646	10840661	-	0	0	0
SINE	Alu	B1_Mus2	chr9	10856167	10856181	+	0	0	0
Simple_repeat	Simple_repeat	(A)n	chr9	10856181	10856186	+	0	0	0
LINE	L1	L1Md_A	chr9	10914750	10915457	-	0.923247	0	0
SINE	Alu	B1_Mus2	chr9	10961284	10961299	-	0	0	0
Simple_repeat	Simple_repeat	(A)n	chr9	10977767	10977771	+	0	0	0
SINE	Alu	B1_Mus1	chr9	10981836	10981851	+	0	0	0
Simple_repeat	Simple_repeat	(A)n	chr9	10981851	10981853	+	0	0	0
SINE	Alu	B1_Mus2	chr11	10288580	10288593	-	0	0	0
SINE	Alu	B1_Mus2	chr9	11043111	11043125	+	0	0	0
Simple_repeat	Simple_repeat	(A)n	chr9	11043125	11043128	+	0	0	0
SINE	B2	B2_Mm1t	chr9	11069903	11069921	-	0	0	0
SINE	Alu	B1_Mus1	chr9	11142407	11142422	-	0	0	0
Simple_repeat	Simple_repeat	(CCAA)n	chr9	11174160	11174163	+	0	0	0
Simple_repeat	Simple_repeat	(A)n	chr9	11174163	11174167	+	0	0	0
LTR	MaLR	MTA_Mm	chr9	11290739	11290778	+	0.591053	0	0
LTR	ERVL	RMER15	chr11	10315778	10315837	+	0	0	0
Simple_repeat	Simple_repeat	(A)n	chr9	11363485	11363488	+	0	0	0
Simple_repeat	Simple_repeat	(A)n	chr9	11396215	11396217	+	0	0	0
Simple_repeat	Simple_repeat	(A)n	chr9	11418057	11418061	+	0	0	0
scRNA	scRNA	4.5SRNA	chr9	11458051	11458060	-	40.5997	21.5213	21.4602
SINE	B2	B2_Mm1a	chr11	10333390	10333410	+	0	0	0
Simple_repeat	Simple_repeat	(A)n	chr11	10333410	10333412	+	0.332194	0	0
SINE	Alu	B1_Mus1	chr9	11571403	11571417	+	0	0	0
Simple_repeat	Simple_repeat	(A)n	chr9	11571417	11571427	+	0	0	0

Simple_repeat	Simple_repeat	(A)n	chr9	11577988	11577992	+	0	0	0
SINE	Alu	B1_Mus1	chr9	11599436	11599451	-	0	0	0
SINE	Alu	B1_Mm	chr10	28845289	28845435	+	0	0	0
LTR	ERVK	RLTR15	chr9	11647872	11647900	-	0	0	0
SINE	Alu	B1_Mus2	chr11	10362220	10362235	-	0	0	0
Simple_repeat	Simple_repeat	(A)n	chr9	11786902	11786905	+	0	0	0
SINE	Alu	B1_Mus2	chr9	11937524	11937538	-	0	0	0
Simple_repeat	Simple_repeat	(A)n	chr9	12038848	12038851	+	0	0.708814	0
SINE	Alu	B1_Mm	chr9	12044404	12044418	-	0	0	0
SINE	Alu	B1_Mus2	chr9	12066168	12066182	+	0	0	0
SINE	B2	B2_Mm1a	chr9	12144150	12144169	-	0	0	0
Simple_repeat	Simple_repeat	(A)n	chr9	12146979	12146983	+	0	0	0
SINE	B2	B2_Mm2	chr9	12252102	12252117	+	2.06418	1.08938	0
Simple_repeat	Simple_repeat	(A)n	chr9	12255155	12255161	+	1.25544	0.761137	0.957947
SINE	B2	B2_Mm1a	chr9	12306655	12306674	+	0	0	0
LTR	MaLR	MTA_Mm	chr9	12313089	12313128	+	0	0	0
SINE	Alu	B1_Mus1	chr9	12313857	12313872	-	0	0	0
SINE	Alu	B1_Mus2	chr9	12338370	12338385	+	0.332194	0	0.615324
Simple_repeat	Simple_repeat	(A)n	chr9	12338385	12338387	+	0.332194	0	0.615324
SINE	Alu	B1_Mm	chrX	3770627	3770772	-	0	0	0
LINE	L1	L1Md_T	chrX	3836687	3843352	-	0	0	0
SINE	Alu	B1_Mus2	chrX	5655774	5655918	+	0	0	0
Simple_repeat	Simple_repeat	(GAAA)n	chrX	5655919	5656005	+	0	0	0
LTR	MaLR	MTB_Mm	chrX	6933336	6933747	-	0	0	0
SINE	Alu	B1_Mm	chrX	7115294	7115440	-	0	0	0
SINE	Alu	B1_Mus2	chrX	7269834	7269957	-	0	0	0
Simple_repeat	Simple_repeat	(TTTA)n	chrX	7292459	7292485	+	0	0	0
SINE	Alu	B1_Mus1	chrX	7526856	7527003	-	0	0	0
SINE	Alu	B1_Mus1	chrX	7570994	7571146	+	0	0	0
LTR	MaLR	ORR1C2	chrX	7571162	7571512	+	0	0	0
LTR	MaLR	MTA_Mm	chrX	7614098	7614490	-	0	0	0
SINE	Alu	B1_Mm	chrX	7668042	7668188	+	0	0	0
Simple_repeat	Simple_repeat	(A)n	chrX	7668189	7668213	+	0	0	0
SINE	Alu	B1_Mur2	chrX	7669740	7669887	+	0	0	0
Low_complexity	Low_complexity	A-rich	chrX	7669888	7669957	+	0	0	0
SINE	Alu	B1_Mm	chrX	7761042	7761190	-	1.84649	1.41763	0.615324
LTR	MaLR	MTA_Mm	chrX	9134901	9135296	+	0	0	0
SINE	Alu	B1_Mm	chr10	5748221	5748349	-	0	0	0
LTR	MaLR	MTA_Mm	chrX	9904355	9904750	-	0	0	0.615324
SINE	B2	B2_Mm1t	chrX	10204472	10204672	-	0	0	0
SINE	Alu	B1_Mus1	chrX	10383269	10383405	+	0	0	0
Simple_repeat	Simple_repeat	(A)n	chrX	10383406	10383438	+	0	0	0
SINE	Alu	B1_Mm	chrX	10407100	10407221	-	0	0	0
LINE	L1	Lx6	chrX	10503178	10509625	-	0	0	0
Simple_repeat	Simple_repeat	(A)n	chrX	10817999	10818034	+	0	0	0
LTR	MaLR	MTA_Mm	chrX	11605626	11606020	+	0	0	0
SINE	B4	RSINE1	chrX	11786086	11786243	-	0	0	0
SINE	Alu	B1_Mus2	chrX	11964377	11964499	+	0	0	0
Simple_repeat	Simple_repeat	(A)n	chrX	11964500	11964524	+	0	0	0
SINE	B4	ID_B1	chrX	12136314	12136515	+	0	0	0
Simple_repeat	Simple_repeat	(A)n	chrX	12136516	12136539	+	0	0	0
SINE	Alu	B1_Mm	chrX	12146322	12146467	-	0	0	0
Low_complexity	Low_complexity	GA-rich	chrX	12406096	12406264	+	0	0	0.685246
SINE	Alu	B1_Mus1	chrX	12588910	12589057	+	0	0.380568	0
Simple_repeat	Simple_repeat	(A)n	chrX	12589058	12589077	+	0	0.380568	0
SINE	Alu	B1_Mus1	chrX	12642007	12642153	-	0	0	0
SINE	Alu	B1_Mus1	chrX	12848109	12848260	+	0	0	0
Simple_repeat	Simple_repeat	(A)n	chrX	12848261	12848293	+	0	0	0
SINE	Alu	B1_Mur1	chrX	13434375	13434541	+	0	0	0.615324
Simple_repeat	Simple_repeat	(A)n	chrX	13434542	13434567	+	0	0	0
SINE	Alu	B1_Mm	chrX	13473255	13473401	-	0	0	0
Simple_repeat	Simple_repeat	(A)n	chrX	13867880	13867925	+	0	0	0
LINE	L1	L1Md_F2	chrX	13978907	13984325	+	0	0	0
LINE	L1	L1_Mus3	chrX	14107344	14113488	-	0	0	0
SINE	Alu	B1_Mm	chrX	14128954	14129099	-	0	0	0
LINE	L1	L1_Mus1	chrX	14442771	14447679	-	0	0	0
LTR	MaLR	MTA_Mm	chrX	14602431	14602825	+	0	0	0

Simple_repeat	Simple_repeat	(A)n	chrX	15062422 15062462 +	0	0	0
SINE	Alu	B1_Mm	chrX	17007823 17007952 +	0.591053	0	0
Simple_repeat	Simple_repeat	(A)n	chrX	17007953 17007981 +	0.591053	0	0
SINE	Alu	PB1D9	chrX	17547820 17547917 +	0	0	0
Simple_repeat	Simple_repeat	(A)n	chrX	17547918 17547937 +	0	0	0
LINE	L1	MusHAL1	chrX	18073917 18078596 -	0	0	0
LINE	L1	L1Md_T	chrX	18763899 18770362 -	0	0	0
SINE	Alu	B1F	chrX	18813069 18813172 +	0	0	0
SINE	Alu	B1_Mus1	chrX	19051267 19051414 +	0	0	0
Simple_repeat	Simple_repeat	(A)n	chrX	19051415 19051445 +	0	0	0
Simple_repeat	Simple_repeat	(A)n	chrX	19086841 19086884 +	0	0	0
LTR	MaLR	MTA_Mm	chrX	19117062 19117460 +	0	0	0
SINE	Alu	B1_Mus2	chrX	19391514 19391659 +	0	0	0
Simple_repeat	Simple_repeat	(A)n	chrX	19391660 19391684 +	0	0	0
SINE	Alu	B1_Mm	chrX	19929425 19929582 -	0	0	0
SINE	Alu	B1_Mus1	chrX	20158965 20159104 -	0	0	0
SINE	Alu	B1_Mus1	chrX	20164798 20164932 +	0	0	0
Simple_repeat	Simple_repeat	(A)n	chrX	20164933 20164962 +	0	0	0
SINE	Alu	B1_Mus2	chrX	20337050 20337196 -	0	0	0
SINE	Alu	B1_Mm	chrX	20363814 20363938 +	0	0	0
Simple_repeat	Simple_repeat	(A)n	chrX	20363939 20363986 +	0	0	0
SINE	Alu	B1_Mus1	chrX	20573732 20573879 -	0	0.708814	0
SINE	Alu	B1_Mm	chrX	20990285 20990431 +	0.591053	0	0
SINE	Alu	B1_Mm	chr11	10592422 10592435 +	0.591053	0	0
Simple_repeat	Simple_repeat	(A)n	chr11	10592435 10592437 +	0	0	0
SINE	B2	B2_Mm1t	chrX	21049478 21049674 +	0	0.708814	0
SINE	Alu	PB1D9	chrX	21178408 21178529 +	0	0	0.615324
Simple_repeat	Simple_repeat	(A)n	chrX	21518832 21518870 +	0	0	0
LTR	MaLR	MTA_Mm	chrX	22422718 22423114 +	0	0	0
Simple_repeat	Simple_repeat	(A)n	chrX	22671925 22671959 +	0	0	0
LINE	L1	L1Md_F2	chrX	23425380 23431778 -	0	0	0
LINE	L1	L1_Mus1	chrX	24200568 24205958 -	0	0	0
LINE	L1	L1_Mus1	chrX	24307301 24313511 +	0	0	0
SINE	Alu	B1_Mur3	chrX	24615743 24615888 +	0	0	0
Simple_repeat	Simple_repeat	(A)n	chrX	24615889 24615921 +	0	0	0
LINE	L1	L1_Mus1	chrX	24947154 24952958 +	0	0	0
Simple_repeat	Simple_repeat	(A)n	chrX	25145525 25145565 +	0	0	0
LINE	L1	L1_Mus1	chrX	25222976 25228779 +	0	0	0
LINE	L1	L1Md_F	chrX	25510300 25516367 -	0	0	0
Simple_repeat	Simple_repeat	(A)n	chrX	26547155 26547196 +	0	0	0
Simple_repeat	Simple_repeat	(A)n	chrX	27315557 27315611 +	0	0	0
LINE	L1	L1Md_F3	chrX	27397079 27402517 +	0	0	0
SINE	Alu	B1_Mur3	chrX	27483384 27483529 -	0	0	0
LINE	L1	L1Md_F2	chrX	27492770 27497960 +	0	0	0
LINE	L1	L1Md_F3	chrX	28397342 28402766 +	0	0	0
SINE	Alu	B1_Mur3	chrX	28483635 28483780 -	0	0	0
LINE	L1	L1Md_F2	chrX	28493021 28498206 +	0	0	0
SINE	Alu	B1_Mur3	chrX	29246009 29246154 +	0	0	0
Simple_repeat	Simple_repeat	(A)n	chrX	29246155 29246181 +	0	0	0
SINE	Alu	B1_Mur3	chrX	29581740 29581885 +	0	0	0
Simple_repeat	Simple_repeat	(A)n	chrX	29581886 29581925 +	0	0	0
LINE	L1	L1_Mus1	chrX	29591169 29597144 -	0	0	0
SINE	Alu	B1_Mur3	chrX	29837398 29837543 -	0	0	0
SINE	Alu	B1_Mus2	chrX	29839263 29839409 -	0	0	0
LINE	L1	L1Md_F2	chrX	29846793 29852177 +	0	0	0
Simple_repeat	Simple_repeat	(A)n	chrX	30009446 30009488 +	0	0	0
SINE	Alu	B1_Mm	chrX	30620851 30620996 -	0	0	0
SINE	Alu	B1_Mm	chrX	31723097 31723242 +	0	0	0
LINE	L1	L1Md_F2	chrX	32366460 32372830 -	0	0	0
SINE	Alu	B1_Mus2	chrX	32380214 32380360 +	0	0	0
Simple_repeat	Simple_repeat	(A)n	chrX	32380361 32380383 +	0	0.380568	0
SINE	Alu	B1_Mur3	chrX	32382080 32382225 +	0	0	0
Simple_repeat	Simple_repeat	(A)n	chrX	32382226 32382253 +	0	0	0
LINE	L1	L1_Mus1	chrX	32987990 32994830 -	0	0	0
LTR	MaLR	MTA_Mm	chrX	33286170 33286576 +	0	0	0
SINE	Alu	B1_Mm	chr11	10658035 10658049 +	0	0	0
Simple_repeat	Simple_repeat	(A)n	chr11	10658049 10658051 +	0	0	0

LTR	MaLR	MTA_Mm	chrX	34078088	34078483	+	0	0	0
SINE	Alu	B1_Mus1	chrX	34343866	34343994	-	0	0	0
SINE	ID	ID4	chrX	35225096	35225167	-	0	0	0
SINE	Alu	B1_Mus1	chrX	35941676	35941878	+	0	0	0
LINE	L1	L1_Mus2	chrX	36094670	36100852	-	0	0	0
LINE	L1	L1_Mus2	chrX	37142544	37147876	-	0	0	0.342623
LINE	L1	L1_Mus1	chrX	38126202	38130691	-	0	0	0
Simple_repeat	Simple_repeat	(A)n	chrX	38357175	38357224	+	0	0	0
LTR	MaLR	MTA_Mm	chrX	38598062	38598457	-	0	0	0
LINE	L1	L1Md_T	chrX	39052200	39059066	-	0	0	0
SINE	Alu	B1_Mm	chr11	10687144	10687158	-	0	0	0
SINE	Alu	B1_Mus1	chr11	10687548	10687562	-	0	0	0
LINE	L1	L1Md_T	chrX	39342191	39348979	-	0	0	0
SINE	Alu	B1_Mus1	chrX	39396760	39396910	+	0	0	0
Simple_repeat	Simple_repeat	(A)n	chrX	39396911	39396934	+	0	0	0
Simple_repeat	Simple_repeat	(A)n	chrX	39709152	39709197	+	0	0	0
SINE	Alu	B1_Mm	chr11	10702019	10702034	-	0	0	0
LTR	MaLR	MTA_Mm	chrX	41802739	41803120	-	0	0	0
Simple_repeat	Simple_repeat	(A)n	chrX	42599160	42599199	+	0	0	0
LINE	L1	L1Md_F2	chrX	42925892	42931989	-	0	0	0
Simple_repeat	Simple_repeat	(A)n	chrX	43109274	43109317	+	0	0	0
SINE	Alu	B1_Mus2	chrX	43856437	43856582	+	0	0	0
SINE	Alu	B1_Mus2	chrX	43888567	43888711	+	0	0	0
LTR	MaLR	MTA_Mm	chrX	44329039	44329429	+	0	0	0
LTR	MaLR	MTA_Mm	chrX	44337179	44337574	+	0	0	0
SINE	Alu	B1_Mus1	chrX	44975345	44975470	-	0	0	0
SINE	Alu	B1_Mus2	chrX	45678722	45678867	-	0	0	0
SINE	Alu	B1_Mus2	chrX	45689743	45689895	-	0	0	0
LTR	MaLR	MTA_Mm	chrX	46080407	46080794	-	0	0	0
LINE	L1	L1Md_T	chrX	46961262	46967207	-	0	0	0
SINE	Alu	B1_Mus2	chrX	47251474	47251615	+	0	0	0
Simple_repeat	Simple_repeat	(A)n	chrX	47251616	47251638	+	0	0	0
SINE	Alu	B1_Mus1	chrX	47261607	47261754	-	0	0	0
SINE	Alu	B1_Mm	chr11	10736596	10736611	-	0	0	0
LTR	MaLR	MTA_Mm	chrX	49043878	49044271	-	0	0	0
SINE	Alu	B1_Mus1	chrX	49204450	49204596	-	0	0	0
LTR	MaLR	MTA_Mm	chrX	49288228	49288623	+	0	0	0
SINE	Alu	B1_Mus2	chrX	49617011	49617156	-	0	0	0
SINE	Alu	B1_Mus2	chrX	50205098	50205244	+	0	0	0
Simple_repeat	Simple_repeat	(A)n	chrX	50264906	50264934	+	0.332194	0	0
LTR	MaLR	MTA_Mm	chrX	50386753	50387148	+	0	0	0
SINE	Alu	B1F	chrX	50737008	50737155	-	0	0	0
LINE	L1	L1Md_T	chrX	50961676	50968149	-	0	0	0
SINE	Alu	PB1D7	chrX	51027592	51027696	+	0	0	0
Simple_repeat	Simple_repeat	(A)n	chrX	51027697	51027742	+	0	0	0
SINE	Alu	B1_Mm	chr11	10759158	10759172	+	0	0	0
Simple_repeat	Simple_repeat	(A)n	chr11	10759172	10759175	+	0	0	0
LTR	MaLR	MTA_Mm	chrX	53135811	53136205	-	0	0	0
LTR	MaLR	MTA_Mm	chrX	53588379	53588776	+	0	0	0
SINE	Alu	B1_Mus1	chr10	29583105	29583238	-	0	0	0
SINE	Alu	PB1D9	chrX	54102827	54102929	-	0	0	0
LTR	MaLR	MTA_Mm	chrX	54813850	54814245	+	0	0	0
Simple_repeat	Simple_repeat	(A)n	chrX	55319184	55319219	+	0	0	0
LTR	MaLR	MTA_Mm	chrX	55473941	55474336	+	0	0	0
Simple_repeat	Simple_repeat	(A)n	chrX	56176249	56176281	+	0	0	0
LTR	MaLR	MTA_Mm	chrX	56477498	56477880	+	0	0	0
LINE	L1	L1Md_T	chrX	56713325	56719726	-	0	0	0
LTR	MaLR	MTA_Mm	chrX	57179252	57179646	-	0	0	0
LTR	MaLR	MTA_Mm	chrX	57436692	57437081	-	0	0	0.615324
LTR	MaLR	MTA_Mm	chrX	57438158	57438503	-	0	0	0
SINE	Alu	B1_Mm	chrX	57866645	57866781	-	0	0	0
LINE	L1	L1Md_T	chrX	58512383	58519652	-	0	0	0
LINE	L1	L1Md_T	chrX	59215611	59222799	-	0.332194	0	0
Simple_repeat	Simple_repeat	(A)n	chrX	59876571	59876616	+	0	0	0
LTR	MaLR	MTA_Mm	chrX	59965929	59966323	-	0	0	0
LINE	L1	L1Md_F	chrX	59972643	59977359	+	0	0	0
LTR	MaLR	MTA_Mm	chrX	60230004	60230399	+	0	0	0

SINE	Alu	B1_Mus2	chrX	60427427	60427572	-	0	0	0
Simple_repeat	Simple_repeat	(A)n	chrX	60813562	60813605	+	0	0	0
Simple_repeat	Simple_repeat	(A)n	chrX	60837569	60837604	+	0	0	0
SINE	Alu	B1_Mus2	chrX	61237437	61237559	+	0	0	0.615324
Simple_repeat	Simple_repeat	(A)n	chrX	61237560	61237602	+	0	0	0
SINE	Alu	B1_Mm	chr10	29692060	29692191	+	0	0	0
Simple_repeat	Simple_repeat	(A)n	chr10	29692192	29692215	+	0	0	0
LINE	L1	L1_Mus1	chrX	61717733	61724157	-	0	0	0
LINE	L1	L1Md_T	chrX	61879182	61886544	-	0	0	0
SINE	Alu	B1_Mur3	chr11	10872521	10872536	+	0.332194	0	0
Simple_repeat	Simple_repeat	(A)n	chr11	10872536	10872539	+	0.332194	0	0
SINE	Alu	B1_Mur4	chr11	10873767	10873781	-	0	0	0
SINE	Alu	B1_Mm	chrX	63079770	63079915	+	0	0	0
Simple_repeat	Simple_repeat	(A)n	chrX	63079916	63079944	+	0	0	0
LINE	L1	L1_Mus3	chrX	63131420	63135933	-	0	0	0
Simple_repeat	Simple_repeat	(A)n	chrX	63755098	63755134	+	0	0	0
SINE	Alu	B1_Mus1	chrX	63928201	63928351	+	0	0	0
Simple_repeat	Simple_repeat	(A)n	chrX	63928352	63928374	+	0	0	0
Simple_repeat	Simple_repeat	(A)n	chrX	64128922	64128985	+	0	0	0
LTR	ERVK	RMER19A	chrX	64297207	64297972	-	0	0	0
LTR	MaLR	MTA_Mm	chrX	64680136	64680531	+	0	0	0
Simple_repeat	Simple_repeat	(A)n	chrX	64715038	64715072	+	0	0	0
LINE	L1	L1_Mus3	chrX	64734841	64741556	-	0	0	0
Simple_repeat	Simple_repeat	(A)n	chrX	64856642	64856675	+	0	0	0
SINE	Alu	B1_Mus2	chrX	64861314	64861460	+	0	0	0
Simple_repeat	Simple_repeat	(A)n	chrX	64861461	64861486	+	0	0	0
LINE	L1	L1Md_F2	chrX	65145337	65151454	-	0.591053	0	0
SINE	Alu	B1_Mus2	chr11	10890723	10890738	+	0	0	0
Simple_repeat	Simple_repeat	(A)n	chr11	10890738	10890740	+	0	0	0
SINE	Alu	B1_Mus2	chrX	65977044	65977190	-	0	0	0
Simple_repeat	Simple_repeat	(A)n	chrX	66259233	66259270	+	0	0	0.615324
LINE	L1	L1Md_F2	chrX	66524249	66530303	-	0	0	0
LTR	MaLR	MTA_Mm	chrX	67662882	67663277	+	0	0	0
LTR	MaLR	MTA_Mm	chrX	67783413	67783808	-	0	0	0
LTR	MaLR	MTA_Mm	chrX	67808526	67808920	+	0.332194	0	0
SINE	Alu	B1_Mus2	chrX	68767453	68767599	+	0	0	0
Simple_repeat	Simple_repeat	(A)n	chrX	68767600	68767693	+	0	0	0
SINE	Alu	B1_Mus1	chrX	68784359	68784493	-	0	0	0
SINE	Alu	B1_Mm	chrX	68828398	68828544	+	0	0	0
Simple_repeat	Simple_repeat	(A)n	chrX	68828545	68828604	+	0	0	0
LTR	MaLR	MTA_Mm	chrX	68881198	68881585	+	0	0	0
SINE	Alu	B1_Mm	chr11	10916289	10916303	+	0	0	0
Simple_repeat	Simple_repeat	(A)n	chr11	10916303	10916306	+	0	0	0
Simple_repeat	Simple_repeat	(A)n	chrX	69274495	69274534	+	0	0	0
Simple_repeat	Simple_repeat	(A)n	chrX	70285603	70285632	+	0	0	0
Simple_repeat	Simple_repeat	(A)n	chrX	70308217	70308258	+	0	0	0
SINE	Alu	B1_Mus2	chrX	70996175	70996320	+	0	0	0
Simple_repeat	Simple_repeat	(A)n	chrX	70996321	70996344	+	0	0	0
SINE	Alu	B1_Mus2	chrX	71508204	71508350	-	0	0	0
SINE	Alu	B1_Mus1	chrX	71537784	71537937	+	1.18211	0.380568	1.23065
Simple_repeat	Simple_repeat	(A)n	chrX	71537938	71537973	+	1.18211	0.380568	0.615324
SINE	Alu	B1_Mm	chrX	71808214	71808360	-	0	0	0
SINE	Alu	B1_Mus1	chrX	72024375	72024496	+	0	0	0
Simple_repeat	Simple_repeat	(A)n	chrX	72024497	72024519	+	0	0	0
Simple_repeat	Simple_repeat	(A)n	chrX	72720343	72720378	+	0	0	0
SINE	Alu	B1_Mm	chrX	72951365	72951511	+	0	0	0
LINE	L1	L1Md_F	chrX	73500002	73504695	-	0	0	0
LTR	MaLR	MTA_Mm	chrX	73880287	73880681	-	0	0	0
LTR	MaLR	MTA_Mm	chrX	75061481	75061879	-	0	0	0
SINE	Alu	B1_Mus1	chrX	75178080	75178227	+	0	0	0
Low_complexity	Low_complexity	A-rich	chrX	75178228	75178289	+	0	0	0
Simple_repeat	Simple_repeat	(A)n	chrX	75438289	75438323	+	0	0	0
Simple_repeat	Simple_repeat	(A)n	chrX	75583441	75583477	+	0	0	0
LTR	MaLR	MTA_Mm	chrX	76045662	76046056	+	0	0.380568	0
LTR	MaLR	MTA_Mm	chrX	76229316	76229699	-	0	0	0
LTR	MaLR	MTA_Mm	chrX	76231748	76232142	-	0	0	0
SINE	Alu	B1_Mus2	chrX	76253395	76253539	+	0	0	0

Simple_repeat	Simple_repeat	(GAAA)n	chrX	76253540	76253605	+	0	0	0
SINE	Alu	B1_Mus1	chrX	76461738	76461884	-	0	0	0
SINE	Alu	B1_Mus2	chrX	76651152	76651298	-	0	0	0
LTR	MaLR	MTA_Mm	chrX	76843725	76844122	+	0	0	0
SINE	Alu	B1_Mus1	chrX	76951577	76951724	-	0	0	0
SINE	Alu	B1_Mus1	chrX	77024945	77025092	-	0	0	0
Simple_repeat	Simple_repeat	(A)n	chrX	77642740	77642796	+	0	0	0
LTR	MaLR	MTA_Mm	chrX	77953157	77953551	-	0	0	0
LINE	L1	L1Md_T	chrX	78204440	78212164	-	0	0	0
LINE	L1	L1Md_F2	chrX	78423161	78429108	-	0	0	0
LTR	MaLR	MTA_Mm	chrX	78548413	78548803	-	0	0	0
SINE	Alu	B1_Mm	chr10	29869878	29870023	-	0	0	0
LTR	MaLR	MTA_Mm	chrX	78652855	78653250	+	0	0	0
SINE	Alu	B1_Mm	chrX	78986725	78986871	+	0	0	0
LTR	MaLR	MTA_Mm	chrX	79158298	79158693	+	0	0	0.615324
Simple_repeat	Simple_repeat	(A)n	chrX	79247284	79247320	+	0	0	0
LTR	MaLR	MTA_Mm	chrX	79374472	79374867	+	0	0	0
LTR	MaLR	MTA_Mm	chrX	80023819	80024213	-	0	0	0
SINE	Alu	B1_Mm	chrX	80140508	80140654	+	2.10535	2.50701	2.4613
Simple_repeat	Simple_repeat	(A)n	chrX	80140655	80140675	+	2.10535	1.41763	2.4613
LTR	MaLR	MTB	chrX	80144189	80144567	+	0.591053	0	1.91589
LINE	L1	Lx3_Mus	chr10	29889781	29895175	-	0	0	0.342623
LTR	MaLR	MTA_Mm	chrX	83205945	83206339	-	0	0	0
SINE	Alu	B1_Mm	chrX	83331486	83331627	+	0	0	0
SINE	Alu	B1_Mus1	chrX	83505415	83505560	+	0	0	0
Simple_repeat	Simple_repeat	(A)n	chrX	85403345	85403392	+	0	0	0
LINE	L1	L1_Mus1	chrX	85801837	85806940	+	0	0	0
LTR	MaLR	MTA_Mm	chrX	85936259	85936653	-	0	0	0
SINE	Alu	B1_Mm	chrX	85960298	85960443	-	0	0	0
Simple_repeat	Simple_repeat	(A)n	chrX	86362277	86362314	+	0	0	0
SINE	Alu	B1_Mus1	chrX	86635814	86635960	-	0	0.708814	0
SINE	Alu	B1_Mus2	chrX	86680092	86680238	+	0	0	0
Simple_repeat	Simple_repeat	(A)n	chrX	86680239	86680301	+	0	0	0
SINE	Alu	B1_Mus1	chrX	87031589	87031735	-	0	0	0
SINE	Alu	B1_Mm	chrX	87038185	87038330	-	0	0	0
Simple_repeat	Simple_repeat	(A)n	chrX	87433908	87433951	+	0	0	0
Simple_repeat	Simple_repeat	(A)n	chrX	87442213	87442248	+	0	0	0
SINE	Alu	B1_Mus1	chrX	88463756	88463902	+	0	0	0
Simple_repeat	Simple_repeat	(A)n	chrX	88463903	88463928	+	0	0	0
LTR	MaLR	MTA_Mm	chrX	88603630	88604027	+	0	0	0
SINE	Alu	B1_Mus2	chrX	88750534	88750626	+	0	0	0
Simple_repeat	Simple_repeat	(A)n	chrX	88750627	88750661	+	0	0	0
Simple_repeat	Simple_repeat	(A)n	chr11	11075235	11075239	+	0	0	0
SINE	Alu	B1_Mus2	chrX	90392811	90392957	-	0	0	0
SINE	Alu	B1_Mus2	chrX	91288957	91289103	-	0	0	0
Simple_repeat	Simple_repeat	(A)n	chrX	91309011	91309050	+	0	0.380568	0
SINE	Alu	PB1D7	chrX	91386993	91387110	-	0	0	0
SINE	Alu	B1_Mur3	chrX	91396885	91397031	-	0	0	0
SINE	Alu	B1_Mus2	chrX	91459263	91459409	-	0	0	0
SINE	Alu	B1_Mur1	chrX	91535037	91535176	-	0	0	0
SINE	Alu	B1_Mus1	chrX	91904744	91904890	+	0	0	0
Simple_repeat	Simple_repeat	(A)n	chr11	11111208	11111210	+	0	0	0
SINE	Alu	B1_Mus1	chrX	92833285	92833431	+	0	0	0
Simple_repeat	Simple_repeat	(A)n	chrX	92833432	92833473	+	0	0	0
Simple_repeat	Simple_repeat	(A)n	chr11	11120949	11120952	+	0	0	0
SINE	Alu	B1_Mus1	chrX	93184887	93185033	+	0	0	0
Simple_repeat	Simple_repeat	(A)n	chrX	93185034	93185081	+	0	0	0
LTR	MaLR	MTA_Mm	chrX	93940612	93941006	-	0	0	0
LTR	MaLR	MTA_Mm	chrX	93982441	93982836	+	0	0	0
LINE	L1	L1VL4	chrX	94975429	94978333	-	0	0	0
LINE	L1	L1Md_T	chrX	95164412	95171295	-	0	0	0
LTR	MaLR	MTA_Mm	chrX	95731169	95731563	+	0	0	0
Simple_repeat	Simple_repeat	(A)n	chrX	96666216	96666258	+	0	0.380568	0.342623
LINE	L1	L1Md_T	chrX	97954332	97960794	-	0	0	0
SINE	Alu	B1_Mm	chrX	98549137	98549289	+	0	0	0
Simple_repeat	Simple_repeat	(A)n	chrX	98549290	98549314	+	0	0	0
SINE	Alu	B1_Mus1	chrX	98678722	98678869	+	0.591053	0	0

Simple_repeat	Simple_repeat	(A)n	chrX	98678870	98678898	+	0.591053	0	0
SINE	Alu	B1_Mus2	chrX	98721081	98721227	-	0	0	0
SINE	Alu	B1_Mus1	chrX	98727146	98727293	-	0	0	0
SINE	Alu	B1_Mus2	chrX	98826874	98827020	-	0	0	0
SINE	Alu	B1F	chrX	99000096	99000245	-	0	0	0
SINE	Alu	B1_Mm	chrX	99356474	99356620	-	0	0	0
SINE	Alu	B1_Mm	chrX	10046250	10046264	-	0	0	0
Simple_repeat	Simple_repeat	(A)n	chrX	10047744	10047748	+	0	0	0
SINE	Alu	B1_Mus2	chrX	10052782	10052797	+	0	0	0
Simple_repeat	Simple_repeat	(A)n	chrX	10052797	10052799	+	0	0	0
SINE	Alu	B1_Mm	chrX	10054123	10054135	+	0	0	0
Simple_repeat	Simple_repeat	(A)n	chrX	10060565	10060568	+	0	0	0
SINE	Alu	B1_Mm	chrX	10070424	10070439	+	1.05163	0.380568	0
Low_complexity	Low_complexity	A-rich	chrX	10070439	10070449	+	0	0.380568	0
SINE	Alu	B1_Mus2	chrX	10075420	10075434	-	0.591053	0	0
LTR	MaLR	MTA_Mm	chr11	11215109	11215148	-	0	0	0
SINE	Alu	B1_Mm	chr11	11215765	11215777	+	0	0	0
Simple_repeat	Simple_repeat	(A)n	chr11	11215777	11215781	+	0	0.380568	0
SINE	Alu	B1_Mus2	chrX	10143576	10143591	+	0	0	0
Simple_repeat	Simple_repeat	(A)n	chrX	10143591	10143594	+	0	0	0
SINE	Alu	B1_Mus2	chrX	10166401	10166416	-	0	0	0
SINE	Alu	B1_Mus2	chrX	10187851	10187866	-	0	0	0
Simple_repeat	Simple_repeat	(A)n	chrX	10221520	10221523	+	0	0	0
LINE	L1	L1Md_F2	chrX	10245206	10245725	+	0	0	0
LTR	MaLR	MTA_Mm	chrX	10264049	10264088	-	0	0	0
Simple_repeat	Simple_repeat	(A)n	chrX	10285325	10285333	+	0	0	0
SINE	Alu	B1_Mus2	chrX	10338026	10338041	-	0	0	0
LINE	L1	L1_Mus2	chrX	10354308	10354887	+	0	0	0
SINE	B2	B2_Mm2	chrX	10411166	10411185	-	0	0	0
LTR	MaLR	MTA_Mm	chrX	10493383	10493422	-	0	0	0
SINE	Alu	B1_Mm	chrX	10520670	10520685	-	0	0	0
Simple_repeat	Simple_repeat	(A)n	chrX	10527906	10527910	+	0	0	0
Simple_repeat	Simple_repeat	(A)n	chrX	10542883	10542887	+	0	0	0
Simple_repeat	Simple_repeat	(A)n	chrX	10548623	10548629	+	0	0	0
LTR	MaLR	MTB	chrX	10554752	10554796	-	0	0	0
LTR	MaLR	MTA_Mm	chrX	10665685	10665724	-	0	0	0
SINE	Alu	B1_Mm	chrX	10670172	10670189	-	0	0	0
Simple_repeat	Simple_repeat	(A)n	chrX	10712103	10712105	+	0	0	0
SINE	Alu	B1_Mm	chrX	10745892	10745906	-	0	0	0
SINE	Alu	B1_Mm	chrX	10836966	10836981	+	0	0	0
Simple_repeat	Simple_repeat	(A)n	chrX	10836981	10836983	+	0	0	0
Simple_repeat	Simple_repeat	(TTTA)n	chrX	10882031	10882035	+	0	0	0
LTR	MaLR	MTA_Mm	chrX	10931748	10931788	+	0	0	0
Simple_repeat	Simple_repeat	(GAA)n	chr11	11289099	11289111	+	0	0	0
LINE	L1	L1_Mus1	chrX	10983472	10984064	-	0	0	0
Simple_repeat	Simple_repeat	(A)n	chrX	11101654	11101658	+	0	0	0
LINE	L1	L1_Mus1	chrX	11127549	11128171	+	0	0	0
LTR	MaLR	MTA_Mm	chrX	11130627	11130666	-	0	0	0
LINE	L1	L1Md_T	chrX	11137429	11138163	-	0	0	0
LINE	L1	L1VL4	chrX	11196169	11196664	-	0	0	0
Simple_repeat	Simple_repeat	(A)n	chrX	11381326	11381330	+	0	0	0
SINE	Alu	B1_Mm	chrX	11390320	11390335	-	0	0	0
LINE	L1	L1Md_F2	chrX	11402905	11403545	-	0	0	0
Simple_repeat	Simple_repeat	(A)n	chrX	11422895	11422902	+	0	0	0
Simple_repeat	Simple_repeat	(A)n	chrX	11517966	11517969	+	0	0	0
LINE	L1	L1_Mus2	chrX	11554416	11554876	-	0	0	0
LTR	MaLR	MTA_Mm	chrX	11575523	11575563	+	0	0	0
LINE	L1	L1Md_F2	chrX	11586980	11587588	-	0	0	0
Simple_repeat	Simple_repeat	(A)n	chrX	11607090	11607093	+	0	0	0
SINE	Alu	B1_Mm	chr11	11348949	11348963	+	1.5143	0	0.615324
SINE	Alu	B1_Mm	chrX	11713125	11713140	-	0	0	0
Simple_repeat	Simple_repeat	(A)n	chrX	11715772	11715775	+	0	0	0
LINE	L1	L1Md_T	chrX	11807278	11807926	-	0	0	0
Simple_repeat	Simple_repeat	(A)n	chrX	11822297	11822302	+	0.332194	0	0
SINE	Alu	B1_Mus1	chrX	11846744	11846759	+	0	0	0
Simple_repeat	Simple_repeat	(A)n	chrX	11846759	11846762	+	0	0	0
Simple_repeat	Simple_repeat	(A)n	chrX	11863382	11863386	+	0	0	0

SINE	Alu	B1_Mm	chr11	11361402	11361416	+	0	0	0
Simple_repeat	Simple_repeat	(A)n	chr11	11361416	11361418	+	0	0	0
Simple_repeat	Simple_repeat	(A)n	chrX	11902958	11902962	+	0	0	0
Simple_repeat	Simple_repeat	(A)n	chr10	30343343	30343377	+	0	0	0.342623
SINE	Alu	B1_Mus1	chrX	11928538	11928553	+	0	0	0
Simple_repeat	Simple_repeat	(A)n	chrX	11928553	11928560	+	0	0	0
LINE	L1	L1Md_F2	chrX	11934612	11935158	+	0	0	0
SINE	Alu	B1_Mus1	chrX	11938701	11938717	+	0	0	0
Low_complexity	Low_complexity	AT_rich	chrX	11938717	11938720	+	0	0	0
SINE	Alu	B1_Mus2	chr11	11363572	11363586	+	0	0	0
Simple_repeat	Simple_repeat	(A)n	chr11	11363586	11363589	+	0	0	0
SINE	Alu	B1_Mus1	chrX	11993797	11993812	+	0	0	0
Simple_repeat	Simple_repeat	(A)n	chrX	11993812	11993816	+	0	0	0
Simple_repeat	Simple_repeat	(A)n	chrX	12016307	12016309	+	0	0	0
Simple_repeat	Simple_repeat	(GGAA)n	chrX	12016309	12016319	+	0	0	0
Low_complexity	Low_complexity	GA-rich	chrX	12018698	12018707	+	0	0	0
LTR	MaLR	MTA_Mm	chr10	30353161	30353551	-	0	0	0
SINE	Alu	B1_Mus1	chrX	12044259	12044274	-	0	0	0
Simple_repeat	Simple_repeat	(GAAA)n	chrX	12059983	12059997	+	0	0	0
Low_complexity	Low_complexity	A-rich	chrX	12060564	12060572	+	0.591053	0	0
SINE	Alu	B1_Mus1	chrX	12067209	12067223	-	0	0	0.615324
SINE	Alu	B1_Mm	chrX	12074235	12074249	+	0	0	0
Simple_repeat	Simple_repeat	(A)n	chrX	12074249	12074254	+	0	0	0
SINE	Alu	B1_Mus1	chrX	12082075	12082090	+	0	0	0
Simple_repeat	Simple_repeat	(GAAA)n	chrX	12082090	12082103	+	0	0	0
Low_complexity	Low_complexity	A-rich	chrX	12083258	12083266	+	0	0	0
Simple_repeat	Simple_repeat	(A)n	chrX	12084894	12084897	+	0.332194	0.380568	0
Simple_repeat	Simple_repeat	(A)n	chrX	12090357	12090362	+	0	0	0
Simple_repeat	Simple_repeat	(GAAA)n	chrX	12104027	12104040	+	0	0	0
Simple_repeat	Simple_repeat	(A)n	chrX	12105187	12105191	+	0	0	0
SINE	Alu	B1_Mus1	chrX	12132437	12132451	+	0	0	0
Low_complexity	Low_complexity	GA-rich	chrX	12132451	12132465	+	0	0	0
SINE	Alu	B1_Mus1	chrX	12133787	12133801	+	0	0	0
Simple_repeat	Simple_repeat	(A)n	chrX	12133802	12133804	+	0	0	0
SINE	Alu	B1_Mus2	chrX	12142364	12142379	-	0	0	0
SINE	Alu	B1_Mus1	chrX	12161183	12161198	-	0	0	0
Simple_repeat	Simple_repeat	(GAAA)n	chrX	12185339	12185352	+	0	0	0
Low_complexity	Low_complexity	A-rich	chrX	12186535	12186544	+	0	0	0
Simple_repeat	Simple_repeat	(GAAA)n	chrX	12197314	12197327	+	0	0	0
Simple_repeat	Simple_repeat	(A)n	chrX	12198245	12198250	+	0	0	0
SINE	Alu	B1_Mus2	chrX	12203903	12203918	+	0	0	0
Low_complexity	Low_complexity	A-rich	chrX	12203918	12203926	+	0	0	0
Simple_repeat	Simple_repeat	(A)n	chrX	12207733	12207736	+	0	0	0
Simple_repeat	Simple_repeat	(A)n	chrX	12258899	12258903	+	0	0	0
SINE	Alu	B1_Mm	chrX	12269768	12269783	-	0.332194	0	0.615324
LTR	MaLR	MTA_Mm	chrX	12303050	12303090	-	0	0	0
LINE	L1	L1_Mus3	chrX	12311133	12311724	-	0	0	0
SINE	Alu	B1_Mm	chrX	12364054	12364068	+	0	0	0
Simple_repeat	Simple_repeat	(A)n	chrX	12364068	12364071	+	0	0	0
Simple_repeat	Simple_repeat	(A)n	chrX	12367979	12367982	+	0	0	0
LTR	ERVK	RMER17D2	chrX	12376591	12376682	-	0	0	0
LINE	L1	L1_Mus2	chrX	12377677	12378252	-	0	0	0
LTR	MaLR	MTA_Mm	chrX	12380100	12380139	-	0	0	0
LTR	MaLR	MTA_Mm	chrX	12428413	12428452	-	0	0	0
LINE	L1	L1Md_F2	chrX	12439704	12440227	+	0	0	0
SINE	Alu	B1_Mus2	chr11	11409151	11409165	-	0	0	0
SINE	Alu	B1_Mus1	chrX	12536123	12536137	-	0	0	0
Simple_repeat	Simple_repeat	(A)n	chrX	12602919	12602924	+	0.591053	0	0
SINE	Alu	B1_Mus2	chrX	12705050	12705064	+	0	0	0
Simple_repeat	Simple_repeat	(A)n	chrX	12719865	12719869	+	0	0	0
SINE	Alu	B1_Mm	chrX	12751221	12751236	+	0	0	0
Simple_repeat	Simple_repeat	(TTTA)n	chrX	12833580	12833586	+	0	0	0
SINE	Alu	B1_Mm	chrX	12846020	12846034	+	0	0	0
Simple_repeat	Simple_repeat	(A)n	chrX	12846034	12846038	+	0	0	0
SINE	Alu	B1_Mm	chrX	12851886	12851900	-	0	0	0
Simple_repeat	Simple_repeat	(A)n	chrX	12859188	12859192	+	0	0	0
Simple_repeat	Simple_repeat	(A)n	chrX	12948744	12948748	+	0	0	0

SINE	Alu	B1_Mus2	chrX	12954976	12954991	+	0	0	0
Simple_repeat	Simple_repeat	(A)n	chrX	12954991	12954995	+	0	0	0
Simple_repeat	Simple_repeat	(A)n	chrX	12972357	12972360	+	0	0	0
SINE	Alu	B1F1	chr11	11446734	11446748	-	0.332194	0	0
SINE	B2	B2_Mm1a	chr11	11446778	11446797	-	0	0	0
LINE	L1	L1Md_F	chrX	13056866	13057404	-	0	0	0
Simple_repeat	Simple_repeat	(A)n	chr11	11452898	11452901	+	0	0	0
LTR	MaLR	MTA_Mm	chrX	13092913	13092953	-	0	0	0
SINE	Alu	B1_Mus1	chr11	11453608	11453622	+	0	0	0
Simple_repeat	Simple_repeat	(A)n	chr11	11453622	11453625	+	0	0	0
Simple_repeat	Simple_repeat	(TTTA)n	chrX	13101170	13101172	+	0	0	0
LTR	MaLR	MTA_Mm	chrX	13138925	13138965	-	0	0	0
LTR	MaLR	MTB	chrX	13239302	13239340	-	0	0	0
LTR	MaLR	MTB	chrX	13280012	13280050	-	0	0	0
SINE	Alu	B1_Mus1	chrX	13280873	13280887	+	0	0	0
Simple_repeat	Simple_repeat	(A)n	chrX	13280887	13280889	+	0	0	0
SINE	Alu	B1_Mm	chrX	13320716	13320731	+	0	0	0
Simple_repeat	Simple_repeat	(A)n	chrX	13320731	13320734	+	0	0	0
SINE	Alu	B1_Mus2	chrX	13322542	13322557	+	0	0	0
Simple_repeat	Simple_repeat	(A)n	chrX	13322557	13322560	+	0	0	0
SINE	Alu	B1_Mus1	chr11	11470984	11470999	-	0	0	0
SINE	Alu	B1_Mus2	chrX	13374475	13374490	+	0	0	0
Simple_repeat	Simple_repeat	(A)n	chrX	13374490	13374496	+	0	0	0
SINE	Alu	B1_Mus1	chrX	13592512	13592527	-	0	0	0.615324
Simple_repeat	Simple_repeat	(A)n	chrX	13733528	13733536	+	0	0	0
LINE	L1	L1Md_T	chrX	13825530	13826156	-	0	0	0
SINE	Alu	B1_Mus2	chrX	13828480	13828495	-	0	0	0
LINE	L1	L1Md_F2	chrX	13846608	13847226	-	0	0	0
SINE	Alu	B1_Mus2	chrX	13882910	13882925	-	0	0	0
SINE	Alu	B1_Mus1	chr10	30548091	30548236	+	0	0	0
Simple_repeat	Simple_repeat	(A)n	chr10	30548237	30548272	+	0	0.708814	0
SINE	Alu	B1_Mus2	chrX	13949225	13949240	+	1.05163	0	0
Simple_repeat	Simple_repeat	(A)n	chrX	13949240	13949246	+	0	0	0.685246
Simple_repeat	Simple_repeat	(A)n	chrX	13967094	13967100	+	0.591053	0	0
SINE	Alu	B1_Mus2	chrX	14071482	14071497	-	0	0	0
Low_complexity	Low_complexity	AT_rich	chrX	14072383	14072386	+	0	0	0
LTR	MaLR	MTA_Mm	chrX	14073316	14073355	-	0	0	0
SINE	Alu	B1_Mus2	chrX	14164145	14164159	+	0	0	0
Simple_repeat	Simple_repeat	(A)n	chrX	14164159	14164165	+	0	0	0
SINE	Alu	PB1D10	chrX	14204665	14204675	-	0.591053	0	0
LINE	L1	L1Md_F2	chrX	14258308	14258827	+	0	0	0
LTR	MaLR	MTD	chrX	14263548	14263591	-	0.591053	0	0
SINE	Alu	B1_Mm	chr11	11541286	11541301	+	0	0	0
Simple_repeat	Simple_repeat	(A)n	chr11	11541301	11541303	+	0	0	0
SINE	Alu	B1_Mm	chr11	11541879	11541893	+	0	0	0
LINE	L1	L1Md_F2	chrX	14318003	14318646	-	0	0	0
Simple_repeat	Simple_repeat	(A)n	chrX	14387704	14387710	+	1.49234	1.08938	2.29225
LTR	MaLR	MTA_Mm	chrX	14388900	14388939	+	0	0	0
LTR	MaLR	MTA_Mm	chrX	14401103	14401141	+	0	0	0
LTR	MaLR	MTA_Mm	chrX	14435337	14435376	-	0	0	0
Simple_repeat	Simple_repeat	(A)n	chrX	14435530	14435534	+	0	0	0
LTR	MaLR	MTA_Mm	chrX	14453421	14453460	-	0	0	0
LTR	MaLR	MTA_Mm	chrX	14502244	14502283	-	0	0	0
LTR	MaLR	MTA_Mm	chrX	14521425	14521464	-	0	0	0
LTR	MaLR	MTA_Mm	chrX	14543466	14543505	-	0	0	0
LTR	MaLR	MTA_Mm	chrX	14580602	14580641	-	0	0	0
Simple_repeat	Simple_repeat	(A)n	chrX	14601306	14601313	+	0	0	0
SINE	B2	B2_Mm2	chr11	11558649	11558666	-	0	0	0
LTR	MaLR	MTA_Mm	chrX	14614186	14614225	-	0	0	0
SINE	B2	B3A	chr11	11558667	11558685	-	0	0	0
LTR	MaLR	MTA_Mm	chrX	14650921	14650960	-	0	0	0
SINE	Alu	B1_Mm	chrX	14731590	14731605	-	0	0	0
Low_complexity	Low_complexity	A-rich	chrX	14757958	14757969	+	0	0	0
SINE	Alu	B1_Mus2	chr11	11574620	11574634	-	0	0	0
SINE	Alu	B1_Mus1	chrX	14854515	14854529	-	0	0.708814	0
SINE	Alu	B1_Mus2	chrX	14872220	14872234	+	0	0	0
SINE	Alu	B1_Mus1	chr11	11587730	11587744	+	0	0	0

Simple_repeat	Simple_repeat	(A)n	chr11	11587744	11587746	+	0	0	0
SINE	Alu	B1_Mus1	chr11	11588010	11588025	-	0	0	0
SINE	Alu	B1_Mus1	chrX	14961957	14961971	+	0	0	0
Simple_repeat	Simple_repeat	(A)n	chrX	14961972	14961974	+	0	0	0
SINE	Alu	B1_Mus1	chr10	30692962	30693116	-	0	0	0
SINE	Alu	B1_Mus1	chrX	14999472	14999487	+	0	0	0
Simple_repeat	Simple_repeat	(A)n	chrX	14999487	14999489	+	0	0	0
SINE	Alu	B1_Mus1	chr11	11595726	11595740	-	0	0	0
LTR	MaLR	MTB	chrX	15023086	15023126	+	0	0	0
Simple_repeat	Simple_repeat	(A)n	chrX	15125927	15125931	+	0	0	0
SINE	Alu	B1_Mus1	chrX	15220092	15220105	-	0	0	0.342623
SINE	Alu	B1_Mus1	chr11	11610218	11610232	+	0	0	0
Simple_repeat	Simple_repeat	(A)n	chr11	11610232	11610236	+	0	0	0
Simple_repeat	Simple_repeat	(A)n	chrX	15349261	15349267	+	0	0	0
SINE	Alu	B1_Mm	chrX	15449087	15449102	+	0	0	0
LINE	L1	L1Md_F2	chrX	15468543	15469046	+	0.591053	0	0
Simple_repeat	Simple_repeat	(A)n	chrX	15502188	15502192	+	0	0	0
SINE	Alu	B1_Mus1	chr11	11632991	11633004	-	0	0	0
LINE	L1	L1Md_F2	chrX	15659235	15659773	+	0	0	0
SINE	Alu	B1_Mm	chrX	15659860	15659875	-	0	0	0
SINE	Alu	B1_Mus1	chr11	11636532	11636547	-	0.591053	0	0
SINE	Alu	B1_Mm	chr11	11637809	11637824	-	0	0	0
LINE	L1	L1Md_T	chrX	15749425	15750000	-	0.332194	0	0
SINE	Alu	B1_Mm	chr11	11649491	11649506	+	0	0	0
LTR	MaLR	MTA_Mm	chr11	11649663	11649701	-	0	0	0
SINE	Alu	B1_Mus2	chr11	11649722	11649736	-	0	0	0
SINE	Alu	B1_Mus2	chrX	15865757	15865772	-	0	0	0
SINE	Alu	B1_Mus2	chrX	15926851	15926866	-	0	0	0
LTR	MaLR	MTA_Mm	chr11	11660440	11660479	+	0	0	0
LINE	L1	L1_Mus1	chrX	15951394	15951901	-	0	0	0
SINE	Alu	B1_Mm	chr11	11663996	11664010	+	0	0	0
Simple_repeat	Simple_repeat	(A)n	chr11	11664010	11664013	+	0	0	0
SINE	Alu	B1_Mus1	chrX	15977820	15977834	-	0	0	0
scRNA	scRNA	BC1_Mm	chrX	16055790	16055802	-	0	0	0
SINE	Alu	B1_Mus2	chrX	16087286	16087300	+	0	0	0
Simple_repeat	Simple_repeat	(A)n	chrX	16087301	16087303	+	0	0	0
SINE	Alu	B1_Mus1	chrX	16128536	16128551	+	0	0	0
Simple_repeat	Simple_repeat	(A)n	chrX	16128551	16128554	+	0	0	0
SINE	Alu	B1_Mm	chr11	11677128	11677143	+	0	0	0
Simple_repeat	Simple_repeat	(A)n	chr11	11677143	11677145	+	0	0	0
Simple_repeat	Simple_repeat	(A)n	chrX	16189651	16189654	+	0	0	0
SINE	Alu	B1_Mm	chrX	16196589	16196604	-	0	0	0
Simple_repeat	Simple_repeat	(A)n	chrX	16197557	16197561	+	0	0	0
SINE	Alu	B1_Mur3	chrX	16256984	16257002	+	0	0	0
SINE	Alu	B1_Mm	chr11	11692252	11692267	-	0	0	0
SINE	Alu	B1_Mur3	chr11	11702582	11702596	-	0	0	0
SINE	Alu	B1_Mus1	chrX	16589731	16589747	+	0	0	0
Simple_repeat	Simple_repeat	(A)n	chrX	16589747	16589753	+	0	0	0
LINE	L1	L1_Mus2	chrX	16596766	16597288	+	0.332194	0	0
SINE	Alu	B1_Mus2	chr11	11744371	11744386	+	0	0	0
Simple_repeat	Simple_repeat	(A)n	chr11	11744386	11744388	+	0	0	0
SINE	Alu	B1_Mus1	chr11	11751316	11751327	-	0	0	0
SINE	Alu	B1_Mm	chr11	11783899	11783912	+	0	0	0
Simple_repeat	Simple_repeat	(A)n	chr11	11783912	11783918	+	0	0	0
SINE	Alu	B1_Mm	chr11	11824201	11824216	+	0	0	0
Simple_repeat	Simple_repeat	(A)n	chr11	11824216	11824220	+	0	0	0
SINE	Alu	B1_Mus1	chr11	11832857	11832871	+	0	0	0
LTR	ERVK	RLTR16	chr11	11832872	11832915	+	0	0	0
LTR	MaLR	MTA_Mm	chr11	11833041	11833081	-	0	0	0
LTR	MaLR	MTB	chr11	11887292	11887331	-	0	0	0
SINE	B2	B2_Mm1a	chr11	12001506	12001525	-	0.591053	0	0
Simple_repeat	Simple_repeat	(A)n	chr11	12016920	12016927	+	0	0	0
SINE	Alu	B1_Mur4	chr10	5887709	5887856	-	0	0	0
SINE	Alu	B1_Mus2	chr11	12129228	12129243	-	0	0	0
LINE	L1	L1Md_A	chrY	805303	810787	-	0	0	0
LINE	L1	L1Md_A	chrY	885815	891300	-	0	0	0
LTR	MaLR	MTA_Mm	chrY	990967	991380	+	0	0	0

LINE	L1	L1Md_T	chrY	1102872	1109956	-	0	0	0
Simple_repeat	Simple_repeat	(A)n	chrY	1253690	1253732	+	0	0	0
Simple_repeat	Simple_repeat	(A)n	chrY	1994334	1994367	+	0	0	0
SINE	Alu	B1_Mus1	chrY	2007789	2007927	-	0	0	0
LINE	L1	L1Md_A	chrY	2537057	2541534	+	0.664388	0.380568	0.342623
LTR	MaLR	MTA_Mm	chr12	3233234	3233623	-	1.18211	0.708814	0
Simple_repeat	Simple_repeat	(A)n	chr12	3347811	3347859	+	0	0	0
SINE	Alu	B1_Mus1	chr12	3511646	3511792	+	0.591053	0	0
Simple_repeat	Simple_repeat	(A)n	chr12	3511793	3511833	+	0	0	0
SINE	Alu	B1_Mm	chr12	3530796	3530942	-	0	0	0
SINE	Alu	B1_Mus1	chr12	3800718	3800862	+	0	0	0
Simple_repeat	Simple_repeat	(A)n	chr12	3800863	3800890	+	0	0	0
LTR	MaLR	MTA_Mm	chr12	4549728	4550121	+	0	0	0
Simple_repeat	Simple_repeat	(A)n	chr12	4561680	4561713	+	0.332194	0	0
SINE	Alu	B1_Mus1	chr12	5647165	5647299	+	0	1.01984	0.615324
Simple_repeat	Simple_repeat	(A)n	chr12	5647300	5647336	+	0	1.01984	0
SINE	Alu	B1_Mus2	chr12	5765325	5765470	-	0	0	0
SINE	Alu	B1_Mus2	chr12	6144701	6144847	-	0	0	0
SINE	Alu	B1_Mus1	chr12	6169239	6169386	+	0	0	0.342623
Simple_repeat	Simple_repeat	(A)n	chr12	6169387	6169413	+	0	0	0.342623
LTR	MaLR	MTA_Mm	chr12	6508038	6508429	-	0	0	0
LTR	MaLR	MTA_Mm	chr12	6567743	6568124	-	0	0	0
LTR	MaLR	MTA_Mm	chr12	6641419	6641811	+	0	0	0
LINE	L1	L1Md_F2	chr12	6650652	6655660	+	0	0	0
LTR	MaLR	MTA_Mm	chr12	6804138	6804531	+	0	0	0
SINE	Alu	B1_Mus2	chr12	7517420	7517567	-	0	0	0
Simple_repeat	Simple_repeat	(A)n	chr12	8490422	8490457	+	0	0	0
LTR	MaLR	MTB	chr12	8529916	8530386	+	0	0	0
SINE	Alu	B1_Mm	chr12	8625459	8625604	+	0	0	0
Simple_repeat	Simple_repeat	(A)n	chr12	8625605	8625629	+	0	0	0
LTR	MaLR	MTA_Mm	chr12	8635000	8635397	-	0.332194	0.380568	0.615324
SINE	Alu	B1_Mus1	chr12	8902711	8902857	+	0	0	0
Simple_repeat	Simple_repeat	(A)n	chr12	8902858	8902887	+	0	0	0
SINE	Alu	B1_Mur1	chr12	8951826	8951972	+	0	0	0
Simple_repeat	Simple_repeat	(A)n	chr12	8951973	8951997	+	0	0	0
LTR	MaLR	MTA_Mm	chr12	9088668	9089063	+	0	0	0
Simple_repeat	Simple_repeat	(A)n	chr12	10023552	10023592	+	0	0	0
LTR	MaLR	MTA_Mm	chr12	10267196	10267593	+	0	0	0
SINE	Alu	B1_Mus2	chr12	10350818	10350963	+	0	0	0
SINE	Alu	B1_Mus1	chr12	10408818	10408965	-	0	0	0
Simple_repeat	Simple_repeat	(A)n	chr12	11528674	11528700	+	0	0	0
Simple_repeat	Simple_repeat	(A)n	chr12	12470827	12470854	+	0	0	0
LTR	MaLR	MTA_Mm	chr12	12673467	12673861	-	0	0	0
SINE	Alu	B1_Mm	chr12	12720986	12721108	-	0	0	0
SINE	Alu	B1_Mm	chr12	12831961	12832106	+	0	0	0
Simple_repeat	Simple_repeat	(A)n	chr12	12832107	12832136	+	0	0	0
SINE	Alu	B1_Mm	chr12	12865689	12865835	+	0	0	0
Simple_repeat	Simple_repeat	(A)n	chr12	12865836	12865860	+	0	0	0
SINE	Alu	B1_Mus1	chr12	12910170	12910324	-	0	0	0
LTR	MaLR	MTA_Mm	chr12	13069297	13069691	+	0	0	0
Simple_repeat	Simple_repeat	(A)n	chr12	14153912	14153951	+	0.332194	0.708814	0.342623
SINE	Alu	B1_Mm	chr12	14591894	14592039	-	0	0	0
SINE	Alu	B1_Mus2	chr12	15124881	15125024	-	0	0	0
LINE	L1	L1Md_F2	chr12	15672344	15676981	-	0	0	0
SINE	Alu	B1_Mus2	chr12	17520134	17520279	+	0	0	0
Simple_repeat	Simple_repeat	(A)n	chr12	17520280	17520317	+	1.05163	0	0
LTR	MaLR	MTA_Mm	chr10	33429352	33429747	-	0	0	0
SINE	Alu	B1_Mm	chr12	19054679	19054824	+	0	0	0
Simple_repeat	Simple_repeat	(A)n	chr12	19054825	19054846	+	0	0	0
SINE	Alu	B1_Mus2	chr12	20833632	20833777	-	0	0	0
SINE	Alu	B1_Mus2	chr12	21392697	21392841	+	0	0	0
Simple_repeat	Simple_repeat	(A)n	chr12	21392842	21392881	+	0	0	0.342623
SINE	Alu	B1_Mm	chr12	21523893	21524010	+	0	0	0
SINE	Alu	B1_Mm	chr12	22319833	22319978	-	0	0	0
Simple_repeat	Simple_repeat	(A)n	chr12	23068473	23068502	+	0	0	0
SINE	Alu	B1_Mus2	chr12	23432127	23432275	-	0	0	0
SINE	Alu	B1_Mus2	chr12	23644845	23644991	+	0	0	0

Simple_repeat	Simple_repeat	(A)n	chr12	23644992	23645031	+	0	0	0
SINE	Alu	B1_Mm	chr12	23782861	23783006	-	0	0	0
SINE	Alu	B1_Mus2	chr12	23868499	23868645	-	0	0	0
SINE	Alu	B1_Mm	chr12	24107656	24107801	-	0	0	0.342623
SINE	Alu	B1_Mus2	chr12	24597763	24597911	+	0	0	0
Simple_repeat	Simple_repeat	(A)n	chr12	24597912	24597934	+	0	0	0
SINE	Alu	B1_Mus2	chr12	25316877	25317024	-	0	0	0
LTR	MaLR	MTA_Mm	chr12	25451960	25452354	-	0	0	0.615324
LTR	MaLR	MTA_Mm	chr12	26484494	26484879	+	0.591053	0	0
Simple_repeat	Simple_repeat	(A)n	chr10	34537130	34537163	+	0	0	0
Simple_repeat	Simple_repeat	(A)n	chr10	34587040	34587080	+	0	0	0
LTR	MaLR	MTA_Mm	chr12	27633293	27633686	-	0	0	0
SINE	Alu	B1_Mm	chr10	34654164	34654310	-	0	0	0
LTR	MaLR	MTA_Mm	chr12	28309116	28309497	+	0	0	0
LTR	MaLR	MTA_Mm	chr10	34774847	34775222	-	0	0	0
LTR	MaLR	MTA_Mm	chr12	28997443	28997839	-	0	0	0
Simple_repeat	Simple_repeat	(A)n	chr12	29943129	29943162	+	0	0	0
LTR	MaLR	MTA_Mm	chr12	30083705	30084100	-	0	0	0
LTR	MaLR	MTA_Mm	chr12	30085196	30085591	-	0	0	0
Simple_repeat	Simple_repeat	(A)n	chr12	31283657	31283743	+	0	0	0
LTR	MaLR	MTA_Mm	chr12	31380953	31381350	-	0	0	0
SINE	Alu	B1_Mus2	chr10	35003976	35004122	-	0	0	0
SINE	Alu	B1_Mus2	chr12	32102993	32103139	+	0	0	0.342623
Simple_repeat	Simple_repeat	(A)n	chr12	32103140	32103163	+	0	0	0.342623
Simple_repeat	Simple_repeat	(A)n	chr12	33194645	33194703	+	0.591053	0	0
LTR	MaLR	MTA_Mm	chr12	33497715	33498114	-	0	0	0
LTR	MaLR	MTA_Mm	chr10	35317848	35318240	-	0	0	0
SINE	Alu	B1_Mm	chr12	34279377	34279527	-	0	0	0
LINE	L1	L1Md_F2	chr10	35332766	35339246	-	0	0	0
LTR	MaLR	MTA_Mm	chr12	34634155	34634547	+	0	0	0
LTR	MaLR	MTA_Mm	chr12	34706725	34707118	-	0	0	0
SINE	Alu	B1_Mm	chr10	6082720	6082866	+	0	0	0
Simple_repeat	Simple_repeat	(A)n	chr10	6082867	6082936	+	0	0	0
SINE	Alu	B1_Mus1	chr12	35627536	35627683	+	0	0	0
Simple_repeat	Simple_repeat	(A)n	chr10	35536181	35536232	+	0	0	0
SINE	Alu	B1_Mus2	chr10	35555519	35555664	-	0	0	0
SINE	Alu	B1_Mus2	chr12	37100994	37101137	+	0	0	0
Simple_repeat	Simple_repeat	(A)n	chr12	37101138	37101166	+	0	0	0.342623
LTR	MaLR	MTA_Mm	chr12	37527071	37527465	-	0	0	0
SINE	Alu	B1_Mus1	chr12	37646652	37646799	-	0	0	0
SINE	Alu	B1_Mm	chr12	37663351	37663514	+	0	0	0
Simple_repeat	Simple_repeat	(A)n	chr12	37663515	37663560	+	0	0	0
SINE	Alu	B1_Mus2	chr12	37697506	37697652	-	0	0	0
Simple_repeat	Simple_repeat	(TTTA)n	chr10	35681421	35681471	+	0	0	0
Simple_repeat	Simple_repeat	(A)n	chr10	35826981	35827015	+	0	0	0
Simple_repeat	Simple_repeat	(A)n	chr12	39483572	39483606	+	0	0	0
SINE	Alu	B1_Mm	chr12	40026568	40026713	+	0	0	0
Simple_repeat	Simple_repeat	(A)n	chr12	40026714	40026738	+	0	0	0
LTR	MaLR	MTA_Mm	chr12	40059374	40059752	-	0	0	0
LTR	MaLR	MTA_Mm	chr12	40362042	40362437	-	0	0	0
SINE	Alu	B1_Mus1	chr10	6107133	6107269	-	0	0	0
LINE	L1	L1Md_T	chr12	44825237	44831659	-	0	0	0
SINE	Alu	B1_Mm	chr12	45321350	45321496	-	0	0	0
LTR	MaLR	MTA_Mm	chr12	45936339	45936737	+	0	0	0
LTR	MaLR	MTB	chr12	46203408	46203806	+	0	0	0
SINE	Alu	B1_Mus2	chr12	46561988	46562134	+	0	0	0
Simple_repeat	Simple_repeat	(A)n	chr12	46562135	46562164	+	0	0	0
SINE	Alu	B1_Mus2	chr12	46743803	46743950	+	0	0	0
Simple_repeat	Simple_repeat	(A)n	chr12	46743951	46743979	+	0	0	0
SINE	B2	B2_Mm1a	chr12	48387467	48387656	-	0.332194	0	0
Simple_repeat	Simple_repeat	(A)n	chr12	48577748	48577792	+	0	0	0
SINE	Alu	B1_Mus1	chr10	6126524	6126667	+	0	0	0
Simple_repeat	Simple_repeat	(A)n	chr10	6126668	6126713	+	0	0	0
SINE	Alu	B1_Mm	chr12	48975926	48976057	-	0	0	0
Simple_repeat	Simple_repeat	(A)n	chr12	49215230	49215267	+	0	0	0
SINE	Alu	B1_Mus2	chr12	49333765	49333892	-	0	0	0
SINE	Alu	B1_Mus1	chr10	36804181	36804324	-	0	0	0

LTR	MaLR	MTA_Mm	chr12	49591008	49591402	-	0	0	0
SINE	Alu	B1_Mm	chr10	36816790	36816917	-	0	0	0
LINE	L1	Lx	chr12	50017821	50022093	+	0	0	0
LTR	MaLR	MTA_Mm	chr12	50224067	50224435	+	0	0	0
LINE	L1	L1Md_F2	chr12	50536723	50543087	-	0	0	0
SINE	Alu	B1_Mus1	chr12	50900820	50900969	-	0	0	0
SINE	Alu	B1_Mus2	chr12	50920296	50920442	-	0	0	0
Simple_repeat	Simple_repeat	(A)n	chr12	51011420	51011463	+	0	0	0
LTR	MaLR	MTA_Mm	chr12	51176537	51176932	-	0	0	0
Simple_repeat	Simple_repeat	(A)n	chr12	52362320	52362385	+	0	0	0
LTR	MaLR	MTA_Mm	chr12	52621033	52621424	-	0	0	0
Simple_repeat	Simple_repeat	(A)n	chr12	53180721	53180756	+	0	0	0.342623
SINE	Alu	B1_Mus1	chr12	53679436	53679598	+	0.923247	3.34916	2.18859
Simple_repeat	Simple_repeat	(A)n	chr12	53719814	53719843	+	0	0	0
Simple_repeat	Simple_repeat	(A)n	chr12	53738902	53738950	+	0	0	0
LINE	L1	L1Md_F2	chr10	37226800	37231846	+	0	0	0
Simple_repeat	Simple_repeat	(A)n	chr12	55392544	55392589	+	0	0	0
SINE	Alu	B1_Mus2	chr12	55406737	55406882	-	0	0	0
LTR	MaLR	MTA_Mm	chr12	55649987	55650381	-	0	0	0
SINE	Alu	B1_Mm	chr12	55682251	55682395	-	0	0	0.615324
SINE	Alu	B1_Mus2	chr12	55801494	55801640	-	0	0	0
LTR	MaLR	MTA_Mm	chr10	37378634	37379028	-	0	0	0
SINE	Alu	B1_Mus2	chr12	55900580	55900709	-	0	0.708814	0
SINE	Alu	B1_Mus2	chr12	55969479	55969625	+	0	0	0
Simple_repeat	Simple_repeat	(A)n	chr12	55969626	55969728	+	0	0	0
SINE	Alu	B1_Mus1	chr12	55988233	55988403	+	0	0	0
Simple_repeat	Simple_repeat	(A)n	chr12	55988404	55988436	+	0	0	0
SINE	Alu	B1_Mm	chr12	56219474	56219620	+	0.591053	0.380568	0.342623
Simple_repeat	Simple_repeat	(A)n	chr12	56219621	56219647	+	0	0.380568	0.342623
LTR	ERV1	RMER21A	chr12	56223649	56224525	+	0	0	0
LTR	ERV1	RMER21A	chr12	56373233	56373972	+	0	0	0
SINE	Alu	B1_Mm	chr12	57338326	57338472	+	0	0	0
Simple_repeat	Simple_repeat	(A)n	chr12	57338473	57338499	+	0	0	0
LTR	MaLR	MTA_Mm	chr12	59393250	59393645	+	0.591053	0	0
Simple_repeat	Simple_repeat	(A)n	chr12	60020970	60020998	+	0	0	0
SINE	Alu	B1_Mur3	chr12	60058234	60058380	+	0	0	0
Simple_repeat	Simple_repeat	(A)n	chr12	60058381	60058409	+	0	0	0
LINE	L1	L1Md_T	chr10	37977914	37984384	-	0	0	0
SINE	B4	ID_B1	chr12	60755680	60755897	-	0	0	0
SINE	Alu	B1_Mm	chr12	61790995	61791134	-	0	0	0
LTR	ERVK	RMER6C	chr12	61917368	61918147	-	0	0	0
SINE	Alu	B1_Mus1	chr12	63144502	63144647	-	0	0	0
Simple_repeat	Simple_repeat	(A)n	chr12	63364314	63364377	+	0	0	0
Simple_repeat	Simple_repeat	(A)n	chr12	63585768	63585805	+	0	0	0
SINE	Alu	B1_Mus2	chr12	64051846	64051991	+	0	0	0
Simple_repeat	Simple_repeat	(A)n	chr12	64051992	64052028	+	0	0	0
Simple_repeat	Simple_repeat	(A)n	chr12	64118647	64118687	+	0	0	0
Simple_repeat	Simple_repeat	(A)n	chr10	38405400	38405427	+	0	0.380568	0
SINE	Alu	B1_Mus1	chr12	64488745	64488890	-	0	0	0
SINE	Alu	B1_Mus1	chr12	64924861	64924999	+	0	0	0
Simple_repeat	Simple_repeat	(A)n	chr12	64925000	64925023	+	0	0	0
Simple_repeat	Simple_repeat	(A)n	chr12	65108165	65108202	+	0	0	0
LTR	MaLR	MTA_Mm	chr12	65166688	65167082	+	0	0	0
SINE	Alu	B1_Mus2	chr12	65712491	65712640	+	0	0	0
Simple_repeat	Simple_repeat	(A)n	chr12	65712641	65712669	+	0	0	0
SINE	Alu	B1_Mus2	chr12	66353088	66353224	-	0	0	0
LTR	MaLR	MTA_Mm	chr12	66607137	66607532	+	0	0	0
SINE	Alu	B1_Mm	chr12	67175772	67175918	-	0	0	0
Simple_repeat	Simple_repeat	(TTTA)n	chr12	67184073	67184112	+	0	0	0
SINE	Alu	B1_Mus1	chr12	67285574	67285711	-	0	0	0
Simple_repeat	Simple_repeat	(A)n	chr12	67458147	67458183	+	0	0	0
SINE	Alu	B1_Mm	chr12	67545878	67545979	-	0	0	0
Simple_repeat	Simple_repeat	(A)n	chr12	68433604	68433646	+	0	0	0.342623
Simple_repeat	Simple_repeat	(A)n	chr12	68499219	68499253	+	0	0	0
Simple_repeat	Simple_repeat	(A)n	chr12	68612327	68612374	+	81.3778	38.4938	50.0266
SINE	Alu	B1_Mm	chr12	68756631	68756774	-	0.827951	0	0
SINE	Alu	B1_Mm	chr12	68868852	68868993	-	0	0	0

SINE	Alu	B1_Mm	chr12	69267045	69267191	+	0	0	0
Low_complexity	Low_complexity	A-rich	chr12	69267192	69267270	+	0	0	0
SINE	Alu	B1_Mm	chr12	69362713	69362858	-	0	0	0
Simple_repeat	Simple_repeat	(A)n	chr12	69560081	69560130	+	0	0	0
SINE	Alu	B1_Mus2	chr12	69687776	69687922	+	0	0	0
Simple_repeat	Simple_repeat	(A)n	chr12	69687923	69687945	+	0	0	0
Simple_repeat	Simple_repeat	(A)n	chr12	69706019	69706058	+	0	0	0
SINE	B2	B2_Mm2	chr12	70050666	70050838	+	0	0	0
SINE	Alu	B1_Mus2	chr12	70145998	70146128	-	0	0	0
SINE	Alu	B1_Mus1	chr12	70339733	70339879	+	0.591053	0	0
SINE	Alu	B1_Mus2	chr12	70564578	70564714	-	0	0	0
SINE	Alu	PB1D7	chr12	70671932	70672056	+	0	0	0
Simple_repeat	Simple_repeat	(A)n	chr12	70672057	70672077	+	0	0	0
Simple_repeat	Simple_repeat	(A)n	chr12	71888551	71888588	+	0	0	0
Simple_repeat	Simple_repeat	(A)n	chr12	71901009	71901037	+	0	0	0
SINE	Alu	B1_Mus2	chr12	72075174	72075320	-	0	0	0.957947
Simple_repeat	Simple_repeat	(A)n	chr12	72398712	72398739	+	0	0	0
SINE	Alu	B1_Mus2	chr12	73709365	73709509	+	0	0	0
Simple_repeat	Simple_repeat	(A)n	chr12	73709510	73709539	+	0	0	0
Simple_repeat	Simple_repeat	(A)n	chr12	73722735	73722762	+	0	0	0
Simple_repeat	Simple_repeat	(A)n	chr12	73730032	73730072	+	0	0	0
SINE	Alu	B1_Mus2	chr12	73749656	73749802	+	0	0	0
Simple_repeat	Simple_repeat	(A)n	chr12	73749803	73749842	+	0	0	0
LTR	MaLR	MTB	chr12	73778348	73778758	+	0	0	0
SINE	Alu	B1_Mus2	chr12	73976684	73976830	+	0	0	0
Simple_repeat	Simple_repeat	(A)n	chr12	73976831	73976865	+	0	0	0
Simple_repeat	Simple_repeat	(A)n	chr12	74039857	74039905	+	0	0	0
SINE	Alu	B1_Mur4	chr12	74189359	74189496	-	0	0	0
Simple_repeat	Simple_repeat	(A)n	chr12	74638590	74638614	+	0	0	0
SINE	Alu	B1_Mm	chr12	75060381	75060526	+	0	0	0
Simple_repeat	Simple_repeat	(A)n	chr12	75060527	75060553	+	0	0	0
Simple_repeat	Simple_repeat	(A)n	chr12	75681540	75681590	+	0.332194	0	0
LTR	MaLR	MTA_Mm	chr12	76309258	76309653	+	0	0	0
scRNA	scRNA	BC1_Mm	chr12	76796967	76797095	+	0	0	0
SINE	Alu	B1_Mus2	chr12	77439098	77439245	+	0	0	0
Simple_repeat	Simple_repeat	(A)n	chr12	77439246	77439277	+	0	0	0
SINE	Alu	B1_Mus2	chr12	77444918	77445064	-	0	0	0.615324
SINE	Alu	B1_Mm	chr12	78074473	78074618	+	0	0	0
Simple_repeat	Simple_repeat	(A)n	chr12	78074619	78074644	+	0	0	0
LTR	MaLR	ORR1B2	chr12	78187941	78188222	-	0	0	0
SINE	Alu	B1_Mus1	chr12	78188241	78188386	-	0	0	0
LTR	MaLR	MTA_Mm	chr12	78247223	78247614	-	0	0	0
Simple_repeat	Simple_repeat	(A)n	chr12	78890202	78890266	+	0.332194	0	0
SINE	Alu	B1_Mus1	chr10	39903332	39903478	+	0	0	0
Simple_repeat	Simple_repeat	(A)n	chr10	39903479	39903502	+	0	0	0
SINE	Alu	B1_Mus2	chr12	79268579	79268725	-	0	0	0
SINE	Alu	B1_Mus2	chr12	80984639	80984748	+	0	0	0
Simple_repeat	Simple_repeat	(A)n	chr12	80984749	80984774	+	0	0	0
Simple_repeat	Simple_repeat	(A)n	chr12	81032358	81032382	+	0	0	0
SINE	B2	B2_Mm2	chr12	81544171	81544357	-	0	0	0
LTR	MaLR	MTA_Mm	chr12	81730585	81730963	-	0	0	0
SINE	Alu	B1_Mus2	chr12	81829544	81829689	-	0	0	0
SINE	Alu	B1_Mus1	chr12	81987851	81987994	-	0	0	0
SINE	Alu	B1_Mus1	chr12	82658880	82659027	-	0	0	0
SINE	Alu	B1_Mm	chr12	82806992	82807120	+	0	0	0
Simple_repeat	Simple_repeat	(A)n	chr10	40264457	40264539	+	0	0	0
SINE	Alu	B1_Mm	chr12	84838543	84838692	-	0	0	0
SINE	Alu	B1_Mus2	chr12	85289350	85289496	-	0	0	0
LTR	MaLR	MTA_Mm	chr12	85390451	85390846	-	0	0	0.342623
SINE	Alu	B1_Mus2	chr12	85572555	85572693	-	0	0	0
SINE	B2	B2_Mm2	chr12	85992682	85992855	-	0	0	0
SINE	Alu	B1_Mm	chr12	86336312	86336457	-	0	0	0.615324
Simple_repeat	Simple_repeat	(A)n	chr12	86621868	86621903	+	0.591053	0.708814	0
SINE	Alu	B1_Mus1	chr12	86718253	86718407	-	0	0	0.342623
SINE	Alu	B1_Mus2	chr12	87063532	87063678	-	0	0	0
SINE	Alu	B1_Mus2	chr12	87077606	87077751	-	0	0	0.342623
Simple_repeat	Simple_repeat	(A)n	chr12	87918789	87918830	+	0	0	0

SINE	Alu	B1_Mus1	chr12	88011031	88011178	-	0	0	0
SINE	Alu	B1_Mus2	chr12	88123916	88124056	+	0	0	0
Simple_repeat	Simple_repeat	(A)n	chr12	88124057	88124085	+	0	0	0
SINE	Alu	B1_Mus1	chr12	88187774	88187901	+	0	0	0.342623
Simple_repeat	Simple_repeat	(A)n	chr12	88187902	88187946	+	0	0	0
SINE	Alu	B1_Mm	chr12	88307650	88307796	+	0.591053	0	0
Simple_repeat	Simple_repeat	(A)n	chr12	88307797	88307822	+	0	0	0
SINE	Alu	B1F1	chr12	88625419	88625556	-	0	0	0
SINE	Alu	B1_Mur4	chr12	88738426	88738571	-	0	0	0
Simple_repeat	Simple_repeat	(A)n	chr12	88761967	88761996	+	0	0	0
SINE	Alu	B1_Mus1	chr12	91753335	91753482	-	0	0	0
SINE	Alu	B1_Mm	chr10	41186346	41186491	-	0	0	0
Simple_repeat	Simple_repeat	(A)n	chr12	93116168	93116208	+	0	0	0
SINE	Alu	B1_Mus2	chr12	93509024	93509171	-	0	0	0
SINE	Alu	B1_Mm	chr12	93545359	93545505	-	0	0	0
Low_complexity	Low_complexity	A-rich	chr12	94504054	94504143	+	0	0	0
SINE	Alu	B1_Mus2	chr12	94656113	94656259	+	0	0	0
Simple_repeat	Simple_repeat	(A)n	chr12	95045784	95045821	+	0	0	0
Simple_repeat	Simple_repeat	(A)n	chr12	95238418	95238454	+	0	0	0
SINE	Alu	B1_Mus1	chr12	95416229	95416374	-	0	0	0
Simple_repeat	Simple_repeat	(A)n	chr12	95466711	95466860	+	0	0	0
Simple_repeat	Simple_repeat	(A)n	chr12	95482469	95482520	+	0	0	0
LTR	MaLR	MTA_Mm	chr12	95486837	95487231	-	0	0	0
SINE	Alu	B1_Mm	chr12	95803657	95803803	-	0	0	0
LINE	L1	L1Md_T	chr12	95855838	95861110	-	0	0	0
SINE	Alu	B1_Mus2	chr12	96312581	96312729	-	0	0	0
Simple_repeat	Simple_repeat	(A)n	chr12	96572291	96572404	+	0	0	0
Simple_repeat	Simple_repeat	(A)n	chr12	96705033	96705078	+	0	0	0
Simple_repeat	Simple_repeat	(A)n	chr12	96921996	96922037	+	0	0	0
SINE	Alu	B1_Mm	chr12	97775062	97775198	-	0	0	0
SINE	Alu	B1_Mm	chr12	97999753	97999899	+	0	0	0
Simple_repeat	Simple_repeat	(A)n	chr12	97999900	97999951	+	0	0	0
SINE	Alu	B1_Mus2	chr10	41800083	41800233	+	0	0	0
Simple_repeat	Simple_repeat	(A)n	chr10	41800234	41800258	+	0	0	0
SINE	B2	B3	chr12	10041043	10041064	+	0	0	0
Simple_repeat	Simple_repeat	(A)n	chr12	10041064	10041066	+	0	0	0
LTR	MaLR	MTA_Mm	chr12	10088902	10088941	+	0	0	0
SINE	B2	B2_Mm2	chr12	10131287	10131306	+	0	0	0.342623
SINE	Alu	B1_Mm	chr10	42004353	42004499	+	0	0	0
SINE	Alu	B1_Mm	chr12	10236374	10236389	+	0	0	0
SINE	Alu	PB1D10	chr12	10255283	10255295	-	0	0	0
SINE	Alu	B1_Mus1	chr12	10369535	10369550	-	0	0	0
SINE	Alu	B1_Mus2	chr12	10373900	10373914	-	0	0	0
SINE	Alu	B1_Mus2	chr12	10395966	10395980	+	0	0	0
Simple_repeat	Simple_repeat	(A)n	chr12	10395980	10395983	+	0	0	0
LTR	MaLR	MTA_Mm	chr12	10473400	10473439	+	0	0	0
LTR	MaLR	MTB_Mm	chr12	10526007	10526047	-	0	0	0
LTR	MaLR	MTA_Mm	chr12	10557599	10557639	-	0	0	0
LTR	MaLR	MTA_Mm	chr10	6708535	6708928	+	0	0	0.342623
Low_complexity	Low_complexity	A-rich	chr10	42273881	42273978	+	0	0	0
Simple_repeat	Simple_repeat	(A)n	chr12	10663251	10663254	+	0	0	0
Simple_repeat	Simple_repeat	(A)n	chr12	10776668	10776672	+	0	0	0
LTR	MaLR	MTA_Mm	chr12	10854833	10854872	+	0	0	0
SINE	Alu	B1_Mus1	chr10	42469695	42469842	-	0	0	0
SINE	Alu	B1_Mus1	chr12	10996834	10996849	-	0	0	0
SINE	Alu	B1_Mus2	chr10	42573059	42573205	-	0	0	0
SINE	Alu	B1_Mus2	chr12	11036162	11036177	+	0	0	0
Simple_repeat	Simple_repeat	(A)n	chr12	11036177	11036180	+	0	0	0
SINE	Alu	B1_Mus1	chr12	11070832	11070847	+	0	0	0
SINE	Alu	B1_Mm	chr12	11123549	11123564	+	0	0	0
Simple_repeat	Simple_repeat	(A)n	chr10	42695503	42695544	+	0	0	0
Simple_repeat	Simple_repeat	(A)n	chr12	11236798	11236803	+	0	0	0
Simple_repeat	Simple_repeat	(A)n	chr12	11272222	11272226	+	0	0	0
SINE	Alu	B1_Mm	chr12	11275066	11275080	-	0	0	0
SINE	Alu	B1_Mus2	chr12	11289974	11289989	-	0	0	0
SINE	Alu	B1_Mus1	chr12	11293723	11293737	+	0	0	0
Simple_repeat	Simple_repeat	(A)n	chr12	11293738	11293740	+	0	0	0

Simple_repeat	Simple_repeat	(A)n	chr10	42932702	42932736	+	0	0	0.615324
Simple_repeat	Simple_repeat	(A)n	chr10	42934984	42935027	+	0.591053	0	0
Simple_repeat	Simple_repeat	(A)n	chr12	11514587	11514592	+	0	0	0
Simple_repeat	Simple_repeat	(A)n	chr12	11516137	11516139	+	0	0	0
Simple_repeat	Simple_repeat	(A)n	chr12	11980921	11980924	+	0	0	0
Simple_repeat	Simple_repeat	(A)n	chr12	11985890	11985894	+	0	0	0
SINE	Alu	B1_Mm	chr10	43307937	43308085	+	0	0	0
SINE	Alu	B1_Mm	chr12	12031891	12031905	-	0	0	0
Simple_repeat	Simple_repeat	(A)n	chr12	12050274	12050279	+	0	0.380568	0
scRNA	scRNA	4.5SRNA	chr12	12056363	12056372	-	0	0	0
LTR	MaLR	MTA_Mm	chr10	6875805	6876192	+	0	0	0
SINE	Alu	B1_Mus2	chr13	3074972	3075118	+	0	0	0
Simple_repeat	Simple_repeat	(A)n	chr13	3075119	3075142	+	0	0	0
SINE	Alu	B1_Mm	chr13	3256411	3256547	-	0	0	0
SINE	Alu	B1_Mus2	chr13	3393746	3393892	+	0	0	0
LTR	MaLR	MTA_Mm	chr10	43421735	43422129	-	0	0	0
SINE	Alu	B1_Mus2	chr13	3629431	3629572	-	0	0	0
SINE	Alu	B1_Mus2	chr13	3631498	3631640	+	0	0	0
Simple_repeat	Simple_repeat	(A)n	chr13	3631641	3631685	+	0	0	0
SINE	Alu	B1_Mus2	chr13	3872925	3873070	-	0	0	0
SINE	Alu	B1_Mm	chr13	4171004	4171150	-	0	0	0
SINE	Alu	B1_Mus2	chr13	4225773	4225919	+	0	0	0
Simple_repeat	Simple_repeat	(A)n	chr13	4225920	4225946	+	0	0	0
LTR	MaLR	MTA_Mm	chr13	4962817	4963210	-	0	0	0
Simple_repeat	Simple_repeat	(A)n	chr10	43547026	43547085	+	0	0	0
LINE	L1	L1Md_T	chr13	5091424	5098088	-	0	0	0
Simple_repeat	Simple_repeat	(A)n	chr10	43571875	43571910	+	0	0	0
SINE	Alu	B1_Mus2	chr13	5471973	5472086	-	0	0	0
LTR	MaLR	MTA_Mm	chr10	43572860	43573255	+	0	0	0
SINE	Alu	B1_Mus2	chr13	5724000	5724145	-	0	0	0
SINE	Alu	B1_Mus1	chr13	6168741	6168882	+	0	0	0
Simple_repeat	Simple_repeat	(A)n	chr13	6168883	6168925	+	0	0	0
Simple_repeat	Simple_repeat	(A)n	chr13	6210177	6210219	+	0	0	0
LTR	MaLR	MTA_Mm	chr13	6536099	6536492	+	0	0	0
Simple_repeat	Simple_repeat	(A)n	chr13	6763760	6763796	+	0	0	0
SINE	Alu	B1_Mus2	chr13	7142048	7142193	+	0	0	0
Simple_repeat	Simple_repeat	(A)n	chr13	7142194	7142220	+	0.591053	0	0
Simple_repeat	Simple_repeat	(A)n	chr13	7338389	7338425	+	0	0	0
Simple_repeat	Simple_repeat	(A)n	chr13	7338573	7338597	+	0	0	0
SINE	Alu	B1_Mm	chr13	7349497	7349642	+	0	0	0
Low_complexity	Low_complexity	GA-rich	chr13	7349643	7349780	+	0	0	0
SINE	Alu	B1_Mm	chr13	7581745	7581889	+	0	0	0
Simple_repeat	Simple_repeat	(A)n	chr13	7581890	7581913	+	0	0	0
Simple_repeat	Simple_repeat	(A)n	chr13	7852506	7852537	+	0	0	0
LTR	MaLR	MTA_Mm-int	chr13	7908604	7909614	-	0	0	0
LTR	MaLR	MTA_Mm	chr13	8066391	8066774	-	0	0	0
Simple_repeat	Simple_repeat	(A)n	chr13	8116770	8116796	+	0	0	0
SINE	Alu	B1_Mm	chr10	43766869	43767014	-	0	0	0
Simple_repeat	Simple_repeat	(A)n	chr13	8776222	8776256	+	0	0	0
LTR	MaLR	MTA_Mm	chr13	8789329	8789723	+	0	0	0
SINE	Alu	B1_Mm	chr13	8925622	8925767	+	0	0	0
SINE	Alu	B1_Mus2	chr13	8996981	8997126	+	0	0	0
Simple_repeat	Simple_repeat	(A)n	chr13	8997127	8997159	+	0	0	0
LTR	MaLR	MTB	chr13	9065833	9066225	-	0	0	0.615324
SINE	Alu	B1_Mm	chr13	9807401	9807541	+	0	0	0
Simple_repeat	Simple_repeat	(A)n	chr13	9807542	9807571	+	0	0	0
LINE	L1	L1Md_T	chr13	9831267	9837911	-	0	0	0
SINE	Alu	B1_Mm	chr13	11241806	11241952	+	0	0	0
Simple_repeat	Simple_repeat	(A)n	chr13	11241953	11241984	+	0	0	0
SINE	Alu	B1_Mm	chr13	12782709	12782855	+	0	0	0
Simple_repeat	Simple_repeat	(A)n	chr13	12782856	12782878	+	0	0	0
SINE	Alu	B1_Mus2	chr10	44207316	44207481	-	0	0	0
LINE	L1	Lx	chr13	13145962	13152191	+	0	0	0.615324
SINE	Alu	B1_Mus1	chr13	13284392	13284535	+	0	0	0
Low_complexity	Low_complexity	A-rich	chr13	13284536	13284614	+	0	0	0
SINE	Alu	B1_Mus2	chr13	13448003	13448154	+	0	0	0
Simple_repeat	Simple_repeat	(A)n	chr13	13448155	13448183	+	0	0	0

SINE	Alu	B1_Mus2	chr13	13517876	13518022	+	0	0	0
Simple_repeat	Simple_repeat	(A)n	chr13	13518023	13518047	+	0	0	0
LTR	MaLR	MTA_Mm	chr13	13654197	13654590	-	0	0	0
SINE	Alu	B1_Mus1	chr13	14133886	14134033	-	0	0	0.615324
SINE	Alu	B1_Mm	chr13	14139606	14139752	+	0	0	0
Simple_repeat	Simple_repeat	(A)n	chr13	14139753	14139783	+	0	0	0.342623
Other	Other	RMER1B	chr13	15249376	15250045	-	0	0	0
Simple_repeat	Simple_repeat	(A)n	chr13	16072236	16072273	+	0	0	0
Simple_repeat	Simple_repeat	(A)n	chr13	16095834	16095870	+	0	0	0
LINE	L1	L1Md_T	chr13	16477070	16483894	-	0.827951	0	0
LTR	ERV1	RLTR23	chr13	16579226	16579713	+	0	0	0
Simple_repeat	Simple_repeat	(A)n	chr13	16872498	16872527	+	0	0	0
LTR	MaLR	MTA_Mm	chr13	17907577	17907973	-	0	0	0
SINE	Alu	B1_Mus2	chr13	18193336	18193482	-	0.591053	0	0
SINE	Alu	PB1D10	chr13	18483657	18483755	-	0	0	0
Simple_repeat	Simple_repeat	(A)n	chr13	18590276	18590324	+	0	0	0
LTR	MaLR	MTA_Mm	chr13	18611277	18611671	-	0	0	0
SINE	Alu	B1_Mus2	chr10	44771632	44771778	+	0	0	0
Simple_repeat	Simple_repeat	(A)n	chr10	44771779	44771817	+	0	0	0
Simple_repeat	Simple_repeat	(A)n	chr13	18707432	18707468	+	0	0	0
SINE	Alu	B1_Mus1	chr13	19961324	19961469	+	0	0	0
Simple_repeat	Simple_repeat	(A)n	chr13	20044640	20044704	+	0	0	0
SINE	Alu	B1_Mus1	chr10	44948957	44949101	-	0	0	0
Simple_repeat	Simple_repeat	(A)n	chr13	22084589	22084618	+	0	0	0
SINE	Alu	B1_Mm	chr13	22124297	22124443	-	0	0	0
LTR	MaLR	MTA_Mm	chr13	23274139	23274534	+	0	0	0
Simple_repeat	Simple_repeat	(A)n	chr13	23500658	23500694	+	0	0	0
Simple_repeat	Simple_repeat	(A)n	chr13	23822094	23822148	+	0	0	0
SINE	Alu	B1_Mm	chr13	25642029	25642186	-	0	0	0
LTR	MaLR	MTA_Mm	chr13	25951168	25951561	-	0	0	0
LINE	L1	L1Md_T	chr13	26331199	26337871	-	0	0	0
LTR	MaLR	MTA_Mm	chr10	45467741	45468131	+	0	0	0
SINE	Alu	B1_Mus2	chr13	26859637	26859783	+	0	0	0
Simple_repeat	Simple_repeat	(A)n	chr13	26859784	26859824	+	0	0	0
LTR	MaLR	MTA_Mm	chr13	27349371	27349764	-	0	0	0
LINE	L1	Lx	chr13	27839811	27845872	+	0	0	0
SINE	Alu	B1_Mus1	chr13	27994561	27994707	-	0	0	0
LINE	L1	L1VL1	chr13	28194016	28199205	+	0	0	0
SINE	B2	B2_Mm2	chr13	28504805	28504986	+	0	0	0
Simple_repeat	Simple_repeat	(A)n	chr13	28504987	28505021	+	0	0	0
LTR	MaLR	MTA_Mm	chr10	45615831	45616225	+	0	0	0
SINE	B2	B2_Mm2	chr10	45666579	45666765	+	0	0	0
Other	Other	RMER1B	chr10	45666766	45668066	+	0	0	0
SINE	Alu	B1_Mm	chr13	29033515	29033661	+	0	0	0
Simple_repeat	Simple_repeat	(A)n	chr13	29033662	29033695	+	0	0	0
Simple_repeat	Simple_repeat	(A)n	chr13	29172487	29172534	+	0	0	0
SINE	Alu	B1_Mus2	chr13	29955738	29955889	-	0	0	0
Simple_repeat	Simple_repeat	(A)n	chr13	30187207	30187236	+	0	0	0
Simple_repeat	Simple_repeat	(A)n	chr13	31481522	31481557	+	0	0	0
Simple_repeat	Simple_repeat	(A)n	chr13	31596039	31596074	+	0	0	0
SINE	Alu	B1_Mus1	chr13	31599178	31599324	+	0	0	0
Simple_repeat	Simple_repeat	(A)n	chr13	31599325	31599392	+	0	0	0
SINE	Alu	B1_Mm	chr13	33362437	33362580	+	0	0	0
SINE	Alu	B1_Mm	chr13	33437000	33437143	+	0	0	0
SINE	Alu	B1_Mm	chr13	33506913	33507056	+	0	0	0
SINE	Alu	B1_Mus2	chr13	33722760	33722911	-	0	0	0
LTR	MaLR	MTA_Mm	chr13	33919288	33919683	-	0	0	0
SINE	Alu	B1_Mus2	chr13	34447082	34447209	+	0	0	0
Simple_repeat	Simple_repeat	(A)n	chr13	34447210	34447263	+	0	0	0
SINE	Alu	B1_Mm	chr10	7170108	7170254	+	0	0	0
Simple_repeat	Simple_repeat	(A)n	chr10	7170255	7170288	+	0	0	0
LTR	MaLR	MTA_Mm	chr13	35492480	35492876	-	0	0	0
LINE	L1	L1_Mus1	chr13	35681586	35687320	+	0	0	0
Simple_repeat	Simple_repeat	(A)n	chr13	35738854	35738890	+	0	0	0
Simple_repeat	Simple_repeat	(A)n	chr13	36720144	36720172	+	0	0	0
Simple_repeat	Simple_repeat	(A)n	chr13	37214783	37214809	+	0	0	0
Simple_repeat	Simple_repeat	(A)n	chr13	37401895	37401937	+	0	0	0

Simple_repeat	Simple_repeat	(A)n	chr10	46582278	46582345	+	0	0	0
SINE	Alu	B1_Mur3	chr13	37540223	37540346	+	0	0	0
SINE	Alu	B1_Mm	chr13	37828491	37828628	+	0	0	0
SINE	B2	B2_Mm1t	chr13	37828645	37828805	+	0	0	0
SINE	Alu	B1_Mur3	chr13	37867562	37867710	+	0	0	0
Simple_repeat	Simple_repeat	(A)n	chr13	37867711	37867733	+	0	0	0
SINE	Alu	B1_Mus2	chr13	38165439	38165586	+	0	0	0
Simple_repeat	Simple_repeat	(A)n	chr13	38165587	38165616	+	0	0	0
Simple_repeat	Simple_repeat	(A)n	chr13	38178197	38178227	+	0	0	0
Simple_repeat	Simple_repeat	(A)n	chr13	39447365	39447418	+	0	0	0
Simple_repeat	Simple_repeat	(A)n	chr13	39778342	39778370	+	0	0	0
LTR	MaLR	MTB	chr13	39893579	39893977	-	0	0	0
Simple_repeat	Simple_repeat	(A)n	chr13	40041626	40041652	+	0	0	0
Simple_repeat	Simple_repeat	(A)n	chr13	40436088	40436123	+	0	0	0
LTR	MaLR	MTA_Mm	chr13	40627378	40627771	-	0	0	0
Simple_repeat	Simple_repeat	(A)n	chr13	40771528	40771560	+	0	0	0
SINE	Alu	B1_Mus1	chr10	46989513	46989651	+	0	0	0
Low_complexity	Low_complexity	GA-rich	chr10	46989652	46989737	+	0	0	0
SINE	Alu	B1_Mus1	chr13	40899383	40899523	-	0	0	0
SINE	Alu	B1_Mus2	chr13	41073739	41073885	-	0	0	0
SINE	Alu	B1_Mus2	chr13	41238278	41238415	+	0	0	0
Simple_repeat	Simple_repeat	(A)n	chr13	41238416	41238439	+	0	0	0
SINE	Alu	B1_Mus2	chr10	47076019	47076166	-	0	0	0
SINE	Alu	B1_Mus1	chr10	47087340	47087486	-	0	0	0
Simple_repeat	Simple_repeat	(A)n	chr10	47105411	47105430	+	0	0	0
LTR	MaLR	MTA_Mm	chr13	42427750	42428138	+	0	0	0
Simple_repeat	Simple_repeat	(A)n	chr13	42487162	42487208	+	0	0	0
Simple_repeat	Simple_repeat	(A)n	chr13	42754315	42754363	+	0	0	0
LTR	MaLR	MTA_Mm	chr13	42762425	42762819	+	0	0	0
Simple_repeat	Simple_repeat	(A)n	chr13	44157084	44157135	+	0	0	0
Simple_repeat	Simple_repeat	(A)n	chr13	44227824	44227865	+	0.332194	0	0
SINE	Alu	B1_Mm	chr13	44467305	44467448	-	0	0	0
Simple_repeat	Simple_repeat	(A)n	chr13	44579491	44579522	+	0	0	0
SINE	Alu	B1_Mm	chr13	44596213	44596361	+	0	0	0
Simple_repeat	Simple_repeat	(A)n	chr13	44596362	44596446	+	0	0	0
SINE	Alu	B1_Mus2	chr13	44640990	44641135	-	0	0	0
LTR	MaLR	MTA_Mm	chr13	44669765	44670160	-	0	0	0
SINE	Alu	B1_Mus1	chr13	44710800	44710946	-	0	0	0
Simple_repeat	Simple_repeat	(A)n	chr13	44746982	44747018	+	0	0	0
Simple_repeat	Simple_repeat	(A)n	chr10	47511353	47511384	+	0	0	0
Simple_repeat	Simple_repeat	(A)n	chr10	47517039	47517065	+	0	0	0
Simple_repeat	Simple_repeat	(A)n	chr13	45115589	45115617	+	0	0.708814	0
Simple_repeat	Simple_repeat	(A)n	chr10	47672008	47672069	+	0	0	0
Simple_repeat	Simple_repeat	(A)n	chr10	47778583	47778637	+	0	0	0
SINE	Alu	B1_Mus2	chr13	47561767	47561904	-	0	0	0
Simple_repeat	Simple_repeat	(A)n	chr13	47993440	47993493	+	0	0.708814	0.957947
SINE	Alu	B1_Mm	chr13	48886106	48886252	+	0	0	0
SINE	Alu	B1_Mus2	chr13	50147655	50147779	+	0	0	0
Low_complexity	Low_complexity	A-rich	chr13	50147780	50147871	+	0	0	0
SINE	Alu	B1_Mus1	chr13	50470512	50470658	+	0	0	0
Simple_repeat	Simple_repeat	(A)n	chr13	50470659	50470723	+	0	0	0
SINE	Alu	B1_Mus1	chr13	51000123	51000273	-	0	0	0
SINE	Alu	B1_Mm	chr13	51162146	51162290	-	0	0	0
SINE	Alu	PB1D10	chr13	51252538	51252637	-	0.591053	0	0
Simple_repeat	Simple_repeat	(A)n	chr10	48380475	48380511	+	0	0	0
SINE	Alu	B1_Mm	chr10	48418109	48418243	+	0	0	0
Simple_repeat	Simple_repeat	(A)n	chr10	48418244	48418266	+	0	0	0
SINE	Alu	B1_Mus1	chr13	52031096	52031242	-	0	0	0
Simple_repeat	Simple_repeat	(TTTA)n	chr13	52428439	52428474	+	0	0	0
SINE	Alu	B1_Mus2	chr13	52462170	52462301	-	0	0	0
LTR	MaLR	MTB	chr13	52589609	52590009	-	0	0	0
SINE	Alu	B1_Mus1	chr13	52640744	52640890	-	0	0	0
Simple_repeat	Simple_repeat	(A)n	chr13	53078076	53078115	+	0	0	0
Simple_repeat	Simple_repeat	(A)n	chr13	53105107	53105145	+	0	0	0
SINE	Alu	B1_Mus2	chr13	53172344	53172490	+	0	0	0
SINE	Alu	B1_Mm	chr13	53198455	53198600	+	0	0	0
Simple_repeat	Simple_repeat	(A)n	chr13	53198601	53198632	+	0	0	0

SINE	Alu	B1_Mus2	chr13	54553896	54554042	-	0.923247	0.708814	1.30057
SINE	Alu	B1_Mm	chr13	54570805	54570951	+	0	0	0.615324
Simple_repeat	Simple_repeat	(A)n	chr13	54570952	54570997	+	0	0	0
SINE	Alu	B1_Mus2	chr13	54673779	54673925	+	0	0	0
Simple_repeat	Simple_repeat	(A)n	chr13	54673926	54673961	+	0	0	0
LINE	L1	L1Md_F2	chr10	48785219	48790547	+	0	0	0
SINE	Alu	B1_Mm	chr13	54770762	54770909	+	0	0	0
SINE	Alu	B1_Mus2	chr13	54773316	54773460	-	0	0	0
SINE	Alu	B1_Mm	chr13	55274929	55275069	-	0	0	0
SINE	Alu	B1_Mus2	chr13	55277071	55277216	+	0	0	0
Simple_repeat	Simple_repeat	(A)n	chr13	55277217	55277243	+	0	0	0
Simple_repeat	Simple_repeat	(A)n	chr13	55282446	55282469	+	0	0	0.342623
SINE	Alu	B1_Mus1	chr13	55544392	55544533	-	0	0	0
SINE	Alu	B1_Mm	chr13	55678232	55678377	+	0	1.01984	0
Simple_repeat	Simple_repeat	(A)n	chr13	55678378	55678403	+	0	1.01984	0
SINE	Alu	PB1D10	chr13	56347080	56347195	-	0	0	0
Simple_repeat	Simple_repeat	(A)n	chr13	56438131	56438172	+	0	0	0
SINE	Alu	B1_Mus2	chr13	56856130	56856276	-	0	0	0
SINE	Alu	B1_Mus2	chr13	57235429	57235547	-	0	0	0
LTR	MaLR	MTA_Mm	chr13	57461942	57462333	+	0	0.380568	0
SINE	B2	B2_Mm1t	chr13	58273325	58273516	+	0	0	0
SINE	Alu	B1_Mus1	chr13	58716863	58717008	+	0	0	0
Simple_repeat	Simple_repeat	(A)n	chr13	58717009	58717041	+	0	0	0
SINE	Alu	B1_Mus1	chr13	59479206	59479353	-	0	0	0
Simple_repeat	Simple_repeat	(A)n	chr13	59524902	59524935	+	0	0	0
LTR	MaLR	MTA_Mm	chr13	59824610	59825005	-	0	0	0
LTR	ERV1	MER89	chr13	60623834	60624307	+	0	0	0
SINE	Alu	B1_Mus1	chr13	60691714	60691861	-	0	0	0
SINE	Alu	B1_Mus1	chr13	61564169	61564313	-	0	0	0
LINE	L1	L1Md_F2	chr13	61617493	61622559	+	0	0	0
SINE	Alu	B1_Mus2	chr13	61815325	61815471	-	0	0	0
SINE	Alu	B1_Mm	chr13	61850194	61850339	-	0	0	0
SINE	Alu	B1_Mus2	chr13	61981422	61981567	+	0	0	0
SINE	Alu	B1_Mus2	chr13	62073027	62073173	+	0	0	0
Simple_repeat	Simple_repeat	(A)n	chr13	62073174	62073211	+	0	0	0
SINE	Alu	B1_Mus1	chr13	62096694	62096831	-	0	0	0
SINE	Alu	B1_Mus2	chr13	62196835	62196981	+	0	0	0
Simple_repeat	Simple_repeat	(A)n	chr13	62196982	62197013	+	0	0	0
SINE	Alu	B1_Mm	chr10	49734284	49734427	+	0	0	0
Simple_repeat	Simple_repeat	(A)n	chr10	49734428	49734474	+	0.591053	0	0
SINE	Alu	B1_Mus2	chr13	62593966	62594112	+	0	0	0
SINE	Alu	B1_Mm	chr13	62615786	62615931	+	0	0	0
Simple_repeat	Simple_repeat	(A)n	chr13	62615932	62615983	+	0	0	0
SINE	Alu	B1_Mm	chr13	62737425	62737571	-	0	0	1.23065
SINE	Alu	B1_Mm	chr13	62808719	62808864	+	0	0	0
Simple_repeat	Simple_repeat	(A)n	chr13	62808865	62808889	+	0	0	0
SINE	Alu	B1_Mus2	chr13	62835842	62835988	+	0	0	0
Simple_repeat	Simple_repeat	(A)n	chr13	62835989	62836010	+	0	0	0
SINE	Alu	B1_Mus2	chr13	63030794	63030939	-	0	0	0
SINE	Alu	B1_Mus2	chr13	64389268	64389429	-	0	0	0
LTR	MaLR	MTA_Mm	chr13	64832090	64832485	-	0	0	0
Simple_repeat	Simple_repeat	(A)n	chr10	50060516	50060550	+	0.591053	0	0
SINE	Alu	B1_Mus2	chr13	65221347	65221485	+	0	0	0
Simple_repeat	Simple_repeat	(A)n	chr13	65221486	65221509	+	0	0	0.615324
LTR	MaLR	MTA_Mm	chr13	65563228	65563622	-	0	0	0
SINE	Alu	B1_Mm	chr13	65720724	65720869	-	0	0	0
SINE	Alu	B1_Mus2	chr10	50160768	50160914	+	0	0	0
SINE	Alu	B1_Mus2	chr13	66087117	66087263	+	0	0	0.615324
LTR	MaLR	MTA_Mm	chr13	66581009	66581403	+	0	0	0
LTR	MaLR	MTA_Mm	chr13	66712376	66712769	-	0	0	0
SINE	Alu	B1_Mus1	chr13	67655089	67655235	+	0	0	0
Simple_repeat	Simple_repeat	(GAAA)n	chr13	67655236	67655313	+	0	0	0
SINE	Alu	B1_Mus2	chr13	67715461	67715607	-	0	0	0
LTR	MaLR	MTB	chr13	69359070	69359466	-	0	0	0
SINE	B2	B2_Mm2	chr13	69623586	69623773	+	0	0	0
LTR	MaLR	MTA_Mm	chr13	69943316	69943714	+	0	0	0
LTR	MaLR	MTB_Mm	chr13	70141061	70141441	-	0.332194	0	0

SINE	Alu	B1_Mus1	chr13	70708555	70708702	+	0	0	0
Simple_repeat	Simple_repeat	(A)n	chr13	70708703	70708746	+	0	0	0
LTR	MaLR	MTA_Mm	chr13	71123828	71124222	+	0	0	0
SINE	Alu	B1_Mm	chr13	71171973	71172119	+	0	0	0
Simple_repeat	Simple_repeat	(A)n	chr13	71172120	71172143	+	0.332194	0	0
Simple_repeat	Simple_repeat	(A)n	chr13	71413475	71413503	+	0	0	0
Simple_repeat	Simple_repeat	(A)n	chr13	71635564	71635592	+	0	0	0
Simple_repeat	Simple_repeat	(A)n	chr13	71806124	71806160	+	0	0	0
Simple_repeat	Simple_repeat	(A)n	chr10	50747265	50747300	+	0	0	0
Simple_repeat	Simple_repeat	(A)n	chr13	72431764	72431804	+	0	0	0
SINE	Alu	B1_Mm	chr13	72592812	72592947	+	0	0	0
Simple_repeat	Simple_repeat	(A)n	chr13	72592950	72592976	+	0	0.380568	0
LTR	MaLR	MTA_Mm	chr13	72914149	72914544	-	0	0	0
SINE	Alu	B1_Mus2	chr13	73053550	73053695	+	0	0	0
Simple_repeat	Simple_repeat	(A)n	chr13	73168501	73168537	+	0	0	0
SINE	Alu	B1_Mus2	chr13	73561803	73561949	-	0	0	0
Simple_repeat	Simple_repeat	(A)n	chr13	74037466	74037504	+	0	0	0
LTR	MaLR	MTA_Mm	chr13	74773048	74773442	+	0	0	0
Simple_repeat	Simple_repeat	(A)n	chr13	75002148	75002180	+	0.332194	0	0
SINE	B2	B2_Mm1a	chr13	75498002	75498193	+	0	0	0
LINE	L1	L1_Mus4	chr13	75628623	75634697	+	0	0	0
SINE	Alu	B1_Mm	chr10	51196566	51196700	-	0	0	0
SINE	Alu	B1_Mm	chr13	76335597	76335743	+	0	0	0
Simple_repeat	Simple_repeat	(A)n	chr13	76335744	76335768	+	0	0	0
LINE	L1	Lx2	chr13	78377855	78383139	-	0	0	0
LINE	L1	L1Md_A	chr13	78437960	78442838	+	0	0	0
LINE	L1	L1_Mus2	chr13	78558013	78563950	+	0	0	0
Simple_repeat	Simple_repeat	(A)n	chr13	78611711	78611760	+	0	0	0
SINE	Alu	B1_Mur2	chr13	79398939	79399079	-	0	0	0
Simple_repeat	Simple_repeat	(A)n	chr13	79631496	79631538	+	0	0.380568	0
SINE	Alu	B1_Mm	chr13	79693013	79693159	+	0	0	0
Simple_repeat	Simple_repeat	(A)n	chr13	79693160	79693183	+	0	0	0
Simple_repeat	Simple_repeat	(A)n	chr13	79922006	79922032	+	0	0	0
LTR	MaLR	MTA_Mm	chr13	79995709	79996103	+	0	0	0
Simple_repeat	Simple_repeat	(CAAAAA)n	chr13	80532706	80532810	+	0	0	0
LTR	MaLR	MTA_Mm	chr13	82293486	82293877	+	0	0	0
SINE	B2	B2_Mm1t	chr13	83055656	83055847	+	0	0	0
Simple_repeat	Simple_repeat	(A)n	chr13	83055848	83055882	+	0.664388	0.380568	0
SINE	Alu	B1_Mm	chr13	83070981	83071127	-	0	0	0
Simple_repeat	Simple_repeat	(A)n	chr13	83372778	83372806	+	0	0	0
Simple_repeat	Simple_repeat	(A)n	chr13	83518060	83518104	+	0	0	0
LTR	ERVK	RMER19B	chr13	83840303	83841215	-	0.591053	0	0
LINE	L1	L1Md_T	chr13	84623034	84630285	-	0	0	0
Simple_repeat	Simple_repeat	(A)n	chr13	85232746	85232780	+	0	0	0
SINE	Alu	B1_Mus1	chr13	85289023	85289167	-	0	0	0
SINE	Alu	B1_Mur4	chr13	85718064	85718210	+	0	0	0
Simple_repeat	Simple_repeat	(A)n	chr13	85718211	85718238	+	0	0	0
SINE	Alu	B1_Mus2	chr13	85892462	85892600	-	0	0	0
SINE	Alu	B1_Mm	chr13	86103579	86103724	+	0	0	0
Simple_repeat	Simple_repeat	(A)n	chr13	86103725	86103770	+	0	0	0
SINE	Alu	B1_Mus2	chr13	86313869	86314013	-	0	0	0
LTR	MaLR	ORR1B2-int	chr13	86445516	86446832	+	0	0	0
SINE	Alu	B1_Mm	chr13	86905562	86905708	-	0	0	0
SINE	Alu	B1_Mm	chr13	87628756	87628896	+	0	0	0
Simple_repeat	Simple_repeat	(A)n	chr13	87628897	87628934	+	0	0	0
Simple_repeat	Simple_repeat	(A)n	chr13	87896976	87897022	+	0	0	0
LTR	MaLR	MTA_Mm	chr13	87998283	87998677	-	0	0	0
SINE	Alu	B1_Mus2	chr13	88224379	88224525	+	0	0	0
SINE	Alu	B1_Mus2	chr13	88351362	88351508	+	0	0	0
Simple_repeat	Simple_repeat	(A)n	chr13	88351509	88351589	+	0	0	0
Simple_repeat	Simple_repeat	(A)n	chr13	88363431	88363457	+	0	0	0
SINE	Alu	B1_Mm	chr13	88431079	88431225	+	0	0	0
LTR	MaLR	MTA_Mm	chr13	88784776	88785170	-	0	0	0
Simple_repeat	Simple_repeat	(A)n	chr10	52250094	52250134	+	0.664388	0	1.02787
Simple_repeat	Simple_repeat	(A)n	chr13	90868424	90868458	+	0	0	0
LTR	MaLR	MTA_Mm	chr13	90940306	90940704	-	0	0	0
SINE	Alu	B1_Mus1	chr13	91535293	91535442	+	0	0	0

Simple_repeat	Simple_repeat	(A)n	chr13	91535443	91535469	+	0	0	0
SINE	B2	B2_Mm2	chr13	91934904	91935096	-	0	0	0
Simple_repeat	Simple_repeat	(A)n	chr13	92904032	92904072	+	0	0	0
SINE	B2	B2_Mm1a	chr13	93179717	93179908	+	0	0	0
Simple_repeat	Simple_repeat	(A)n	chr13	93179909	93179942	+	0.664388	0	0.342623
SINE	Alu	B1_Mus2	chr13	94007260	94007405	-	0	0	0
SINE	Alu	B1_Mus2	chr13	94320870	94321015	+	0	0	0.342623
Simple_repeat	Simple_repeat	(A)n	chr13	94321016	94321049	+	0	0	0
SINE	Alu	B1_Mus1	chr13	94364208	94364354	+	0	0	0
Simple_repeat	Simple_repeat	(A)n	chr13	94364355	94364390	+	0	0.380568	0
SINE	Alu	B1_Mus1	chr13	95111699	95111844	+	0	0	0
Simple_repeat	Simple_repeat	(A)n	chr13	95111845	95111883	+	0	0	0
Simple_repeat	Simple_repeat	(A)n	chr13	96686650	96686688	+	0	0	0
LTR	MaLR	MTA_Mm	chr13	96976470	96976865	-	0	0	0
LTR	MaLR	MTA_Mm	chr13	97479721	97480106	-	0	0	0
SINE	Alu	B1_Mm	chr13	97520114	97520251	-	0	0	0
LTR	MaLR	MTA_Mm	chr13	97553052	97553433	-	0	0	0
SINE	Alu	B1_Mm	chr13	98144829	98144974	-	0	0	0
SINE	Alu	B1_Mus2	chr13	99046571	99046717	+	0	0	0
Simple_repeat	Simple_repeat	(A)n	chr13	99046718	99046740	+	0	0	0
LTR	MaLR	MTA_Mm	chr10	53174693	53175069	+	0	0	0
SINE	Alu	B1_Mus1	chr13	99116714	99116869	+	0	0	0
SINE	Alu	B1_Mus1	chr10	53178343	53178488	+	0	0	0
Simple_repeat	Simple_repeat	(A)n	chr10	53178489	53178518	+	0	0	0
SINE	Alu	B1_Mus2	chr13	99598967	99599114	+	0	0	0
Simple_repeat	Simple_repeat	(A)n	chr13	99599115	99599143	+	0	0	0
SINE	Alu	B1_Mus2	chr13	99699179	99699325	-	0	0	0
SINE	Alu	B1_Mm	chr13	99750249	99750394	+	0	0	0
SINE	Alu	B1_Mus2	chr13	10116095	10116109	-	0	0	0
Simple_repeat	Simple_repeat	(A)n	chr13	10123716	10123719	+	0	0	0
SINE	Alu	B1_Mus2	chr13	10161799	10161813	-	0	0	0
LTR	MaLR	MTA_Mm	chr13	10163368	10163407	-	0	0	0.342623
LINE	L1	L1Md_F2	chr10	53549275	53555455	-	0	0	0
LTR	MaLR	MTA_Mm	chr13	10211032	10211071	+	0	0	0
SINE	Alu	B1_Mus1	chr13	10423011	10423021	+	0	0	0
Simple_repeat	Simple_repeat	(A)n	chr13	10423021	10423025	+	0	0	0
SINE	Alu	B1_Mm	chr13	10423176	10423191	+	0	0	0
Simple_repeat	Simple_repeat	(A)n	chr13	10423191	10423196	+	0	0	0
SINE	Alu	B1_Mus2	chr13	10480008	10480023	-	0	0	0
SINE	Alu	B1_Mus1	chr10	7822651	7822780	+	0	0	0
Simple_repeat	Simple_repeat	(A)n	chr13	10608937	10608942	+	0	0	0
Simple_repeat	Simple_repeat	(A)n	chr13	10651802	10651805	+	0	0	0
LTR	MaLR	MTA_Mm	chr13	10727185	10727224	-	0	0	0
LTR	MaLR	MTA_Mm	chr13	10729853	10729892	-	0	0	0
SINE	Alu	B1_Mm	chr13	10787496	10787510	-	0	0	0
SINE	Alu	B1_Mus1	chr13	10827701	10827716	+	0	0	0
Simple_repeat	Simple_repeat	(A)n	chr13	10827716	10827723	+	0	0	0
Simple_repeat	Simple_repeat	(A)n	chr13	10829685	10829690	+	0	0	0
LINE	L1	L1Md_T	chr13	10929342	10930007	-	0	0	0
SINE	Alu	PB1D7	chr10	54360832	54360953	-	0	0.708814	0
SINE	Alu	B1_Mus2	chr10	54470339	54470487	+	0	0	0
Simple_repeat	Simple_repeat	(A)n	chr13	11109186	11109190	+	0	0	0
Simple_repeat	Simple_repeat	(A)n	chr13	11132979	11132983	+	0	0	0.342623
Simple_repeat	Simple_repeat	(A)n	chr10	54558813	54558842	+	0	0	0
LTR	MaLR	MTB	chr13	11160828	11160868	-	0	0	0
Simple_repeat	Simple_repeat	(A)n	chr13	11215978	11215980	+	0	0	0
LTR	MaLR	MTA_Mm	chr13	11219905	11219945	+	0	0	0
SINE	Alu	B1_Mm	chr13	11228450	11228464	+	0	0	0
Simple_repeat	Simple_repeat	(A)n	chr13	11228464	11228467	+	0	0	0
LTR	MaLR	MTA_Mm	chr13	11230415	11230454	+	0	0	0
LTR	MaLR	MTA_Mm	chr13	11230564	11230604	+	1.18211	0	0
Simple_repeat	Simple_repeat	(A)n	chr13	11231660	11231666	+	0	0	0
SINE	Alu	B1_Mm	chr13	11232909	11232924	+	0	0	0.342623
Simple_repeat	Simple_repeat	(A)n	chr13	11232924	11232926	+	0	0	0.342623
SINE	Alu	B1_Mus2	chr13	11252844	11252858	+	0	0	0.342623
Simple_repeat	Simple_repeat	(A)n	chr13	11252858	11252860	+	0.332194	0	0
Simple_repeat	Simple_repeat	(A)n	chr13	11284881	11284885	+	0	0	0

Simple_repeat	Simple_repeat	(A)n	chr13	11289059	11289062	+	0	0	0
LTR	MaLR	MTB	chr13	11307347	11307387	+	0	0	0
Simple_repeat	Simple_repeat	(A)n	chr13	11321759	11321764	+	0	0.380568	0
LTR	MaLR	MTA_Mm	chr13	11414840	11414880	+	0	0	0
LTR	MaLR	MTA_Mm	chr13	11423443	11423483	+	0	0	0
LTR	MaLR	MTA_Mm	chr13	11430184	11430223	+	0	0	0
Simple_repeat	Simple_repeat	(A)n	chr13	11457805	11457807	+	0	0	0
SINE	B2	B3	chr13	11523869	11523888	-	0	0	0
LTR	MaLR	MTB	chr13	11526604	11526644	+	0	0	0
LTR	MaLR	MTA_Mm	chr10	55147670	55148064	-	0	0	0
Simple_repeat	Simple_repeat	(A)n	chr13	11679166	11679170	+	0.591053	0.380568	0.957947
LTR	MaLR	MTA_Mm	chr13	11759153	11759193	-	0	0	0
LTR	MaLR	MTA_Mm	chr13	11925603	11925642	-	0	0	0
LINE	L1	L1Md_F2	chr13	11978540	11979067	+	0	0	0
Simple_repeat	Simple_repeat	(A)n	chr14	3019467	3019492	+	0	0	0
SINE	Alu	B1_Mus1	chr14	4271863	4272009	-	0	0	0
Simple_repeat	Simple_repeat	(A)n	chr14	5223751	5223776	+	0	0	0
Simple_repeat	Simple_repeat	(A)n	chr14	5360784	5360819	+	0	0	0.342623
SINE	Alu	B1_Mus1	chr14	5402442	5402589	+	0	0	0
Low_complexity	Low_complexity	A-rich	chr14	5402590	5402639	+	0	0	0
Simple_repeat	Simple_repeat	(A)n	chr14	6408820	6408845	+	0	0	0
Simple_repeat	Simple_repeat	(A)n	chr14	6545857	6545890	+	0	0	0.342623
SINE	Alu	B1_Mus1	chr14	6587705	6587852	+	0	0	0
Low_complexity	Low_complexity	A-rich	chr14	6587853	6587903	+	0	0	0.342623
Simple_repeat	Simple_repeat	(A)n	chr14	7165543	7165613	+	0	0	0
SINE	B2	B2_Mm1a	chr14	8516825	8517021	+	0	0	0
SINE	Alu	B1_Mm	chr10	56003776	56003921	+	0	0	0
SINE	Alu	B1_Mus2	chr14	8594154	8594300	-	0	0	0
LTR	MaLR	MTA_Mm	chr14	8599962	8600343	-	0	0	0
LTR	MaLR	MTA_Mm	chr14	8646969	8647364	+	0	0	0
SINE	Alu	B1_Mm	chr14	9021558	9021703	+	0	0	0
Simple_repeat	Simple_repeat	(A)n	chr14	9021704	9021758	+	0	0	0
LTR	MaLR	MTA_Mm	chr14	9850607	9851000	+	0	0	0
LTR	MaLR	MTA_Mm	chr14	9852100	9852493	+	0	0	0.342623
SINE	Alu	B1_Mus2	chr10	56113910	56114056	-	0	0	0
SINE	Alu	B1_Mus2	chr10	56145755	56145908	+	0	0	0
Simple_repeat	Simple_repeat	(A)n	chr10	56145909	56145936	+	0	0	0
SINE	Alu	B1_Mur2	chr10	56193647	56193784	-	0	0	0
SINE	Alu	B1_Mm	chr14	12125289	12125435	+	0	0	0
Simple_repeat	Simple_repeat	(A)n	chr14	12125436	12125461	+	0	0	0
SINE	Alu	B1_Mm	chr10	56267522	56267668	+	0	0	0
Simple_repeat	Simple_repeat	(A)n	chr10	56267669	56267694	+	0	0	0
LTR	MaLR	MTA_Mm	chr14	13143360	13143751	-	0.591053	0	0
Simple_repeat	Simple_repeat	(A)n	chr10	56300529	56300585	+	0	0	0
LINE	L1	L1Md_F2	chr10	56338075	56344228	-	0	0	0
Simple_repeat	Simple_repeat	(A)n	chr14	14485581	14485607	+	0	0	0
SINE	Alu	B1_Mus1	chr14	14732433	14732576	+	0	0	0
Simple_repeat	Simple_repeat	(A)n	chr14	14732577	14732609	+	0	0	0
SINE	Alu	B1_Mm	chr14	14827144	14827289	-	0	0	0
LINE	L1	L1_Mus1	chr14	15087175	15091630	-	0	0.380568	0
LINE	L1	L1_Mus2	chr14	15188554	15193433	+	0	0	0
LINE	L1	L1_Mus2	chr14	15417219	15423484	-	0	0.708814	0
Simple_repeat	Simple_repeat	(A)n	chr14	15508711	15508739	+	0	0	0.615324
LTR	MaLR	MTA_Mm	chr14	15647598	15647992	+	0	0	0
Simple_repeat	Simple_repeat	(A)n	chr14	15897940	15897986	+	0	0	0
SINE	Alu	B1_Mus1	chr14	16030748	16030895	+	0	0	0
Simple_repeat	Simple_repeat	(A)n	chr14	16030896	16030943	+	0	0	0
LINE	L1	L1_Mus3	chr14	16108848	16113632	+	0	0	0
LINE	L1	L1_Mus1	chr14	17183355	17187733	-	0	0	0
LTR	MaLR	MTA_Mm	chr14	17969965	17970351	-	0.591053	0.708814	0
Simple_repeat	Simple_repeat	(A)n	chr14	18221184	18221221	+	0	0	0
LTR	MaLR	MTA_Mm	chr14	18415648	18416040	-	0.591053	0	0.615324
LTR	MaLR	MTA_Mm	chr14	18486297	18486696	+	0	0	0
Simple_repeat	Simple_repeat	(A)n	chr10	56759332	56759366	+	0.591053	0	0
SINE	Alu	B1_Mus2	chr14	19097662	19097800	+	0	0	0
Simple_repeat	Simple_repeat	(A)n	chr14	19097801	19097848	+	0	0	0
SINE	Alu	B1_Mm	chr14	20023156	20023292	+	0	0	0

Simple_repeat	Simple_repeat	(A)n	chr14	20023293	20023320	+	0	0	0
LTR	MaLR	MTA_Mm	chr14	20053001	20053395	-	0	0	0
Simple_repeat	Simple_repeat	(A)n	chr14	20305130	20305184	+	0	0	0
SINE	Alu	B1_Mus1	chr14	20685450	20685603	+	0	0	0
Simple_repeat	Simple_repeat	(A)n	chr14	20685604	20685627	+	0	0	0
SINE	Alu	B1_Mus1	chr14	20844841	20844962	+	0	0	0
Simple_repeat	Simple_repeat	(GAAA)n	chr14	20844963	20845013	+	0	0	0
Simple_repeat	Simple_repeat	(A)n	chr14	20931794	20931824	+	0	0	0
SINE	Alu	B1_Mm	chr14	20955724	20955860	+	0	0	0
Simple_repeat	Simple_repeat	(A)n	chr14	20955861	20955883	+	0	0	0
Simple_repeat	Simple_repeat	(A)n	chr14	21300380	21300409	+	0	0	0
Simple_repeat	Simple_repeat	(A)n	chr14	21450957	21451005	+	0	0	0
SINE	Alu	B1_Mus1	chr14	21688914	21689060	-	0.591053	0	0
Simple_repeat	Simple_repeat	(A)n	chr14	21713466	21713492	+	0	0	0
SINE	B2	B2_Mm1a	chr14	21715851	21716043	+	0	0	0
Simple_repeat	Simple_repeat	(A)n	chr14	21716044	21716069	+	1.38382	0.708814	0.685246
LTR	MaLR	MTA_Mm	chr14	23995720	23996113	+	0	0	0
SINE	Alu	B1_Mm	chr14	26952216	26952361	-	0	0	0
SINE	Alu	B1_Mus2	chr14	26952950	26953096	+	0	0	0
Simple_repeat	Simple_repeat	(A)n	chr14	26953097	26953120	+	0	0.380568	0
SINE	Alu	B1_Mm	chr14	27091865	27092010	-	0	0	0
SINE	Alu	B1_Mus2	chr14	27092599	27092745	+	0	0	0
Simple_repeat	Simple_repeat	(A)n	chr14	27092746	27092769	+	0	0	0
SINE	Alu	B1_Mus1	chr10	57774628	57774775	-	0	0	0
SINE	Alu	B1_Mus2	chr14	28223983	28224129	+	0	0.708814	0
Simple_repeat	Simple_repeat	(A)n	chr14	28224130	28224158	+	0	0.708814	0
LTR	MaLR	MTA_Mm	chr14	28363619	28364014	-	0.591053	0	0
SINE	Alu	B1_Mus2	chr14	28401938	28402082	-	0	0	0
Simple_repeat	Simple_repeat	(A)n	chr14	29610337	29610441	+	0	0	0
SINE	Alu	B1_Mus2	chr14	30745642	30745783	+	0	0	0
Simple_repeat	Simple_repeat	(A)n	chr14	30745784	30745828	+	0	0	0
SINE	Alu	B1_Mus1	chr14	30775571	30775716	-	0	0	0
SINE	Alu	B1_Mus2	chr14	31936139	31936284	-	0	0	0
LTR	MaLR	MTB	chr10	58181127	58181525	+	0	0	0
LTR	MaLR	MTA_Mm	chr14	32143313	32143708	-	0.332194	0	0
LTR	MaLR	MTA_Mm	chr14	32398327	32398725	+	0	0	0
Simple_repeat	Simple_repeat	(A)n	chr14	32496883	32496912	+	0	0	0
SINE	Alu	B1_Mus1	chr14	32648480	32648623	-	0	0	0
SINE	Alu	B1_Mus2	chr14	32935988	32936134	+	0	0	0
Simple_repeat	Simple_repeat	(A)n	chr14	32936135	32936157	+	0	0	0
SINE	Alu	B1_Mm	chr14	33555429	33555576	+	0	0	0
Simple_repeat	Simple_repeat	(A)n	chr14	33555577	33555670	+	0.332194	0	0
Simple_repeat	Simple_repeat	(A)n	chr14	34184460	34184495	+	0	0	0
Simple_repeat	Simple_repeat	(A)n	chr14	34296510	34296538	+	0	0	0.342623
Simple_repeat	Simple_repeat	(A)n	chr14	34563721	34563759	+	0	0	0
LTR	MaLR	MTB	chr14	34671328	34671725	-	0	0	0
Simple_repeat	Simple_repeat	(A)n	chr14	34765411	34765445	+	0	0	0
SINE	Alu	B1_Mus2	chr14	35484377	35484526	+	0	0	0
Simple_repeat	Simple_repeat	(A)n	chr14	35484527	35484570	+	0	0	0
LINE	L1	L1_Mus2	chr14	37040240	37046087	-	0	0	0
LINE	L1	L1Md_F2	chr14	37052070	37058253	-	0	0	0
Simple_repeat	Simple_repeat	(A)n	chr14	37100296	37100324	+	0	0	0
SINE	Alu	B1_Mus2	chr14	37389033	37389179	-	0	0	0
Simple_repeat	Simple_repeat	(A)n	chr14	38074507	38074555	+	0	0	0
LTR	MaLR	MTA_Mm	chr14	38271599	38271994	-	0.332194	0	0
Simple_repeat	Simple_repeat	(A)n	chr14	38300956	38300999	+	0	0	0.342623
Simple_repeat	Simple_repeat	(A)n	chr14	38323203	38323240	+	0	0	0
SINE	Alu	B1_Mus2	chr14	38938544	38938659	-	0	0	0
LINE	L1	L1Md_F2	chr14	40529522	40535385	-	0	0	0
SINE	Alu	B1_Mus2	chr14	40783900	40784045	-	0	0	0
SINE	Alu	B1_Mm	chr14	41186299	41186450	+	0	0	0
SINE	Alu	B1_Mus2	chr14	42329045	42329190	-	0	0	0

Supplementary Table S4: Predicted repeat targets based on piRNA cleavage signature in addition to their class; family; subfamily; genomic coordinates and normalized CAGE expression throughout postnatal development in brain
